# Human splice factors contribute to latent HIV infection in primary cell models and blood CD4^+^ T cells from ART-treated individuals

**DOI:** 10.1371/journal.ppat.1009060

**Published:** 2020-11-30

**Authors:** Sara Moron-Lopez, Sushama Telwatte, Indra Sarabia, Emilie Battivelli, Mauricio Montano, Amanda B. Macedo, Dvir Aran, Atul J. Butte, R. Brad Jones, Alberto Bosque, Eric Verdin, Warner C. Greene, Joseph K. Wong, Steven A. Yukl

**Affiliations:** 1 University of California San Francisco, San Francisco, California, United States of America; 2 San Francisco VA Medical Center, San Francisco, California, United States of America; 3 George Washington University, Washington DC, United States of America; 4 Buck Institute, Novato, California, United States of America; 5 Gladstone Institutes, San Francisco, California, United States of America; 6 Infectious Diseases Division, Weill Cornell Medicine, New York City, New York, United States of America; Vaccine Research Center, UNITED STATES

## Abstract

It is unclear what mechanisms govern latent HIV infection *in vivo* or in primary cell models. To investigate these questions, we compared the HIV and cellular transcription profile in three primary cell models and peripheral CD4^+^ T cells from HIV-infected ART-suppressed individuals using RT-ddPCR and RNA-seq. All primary cell models recapitulated the block to HIV multiple splicing seen in cells from ART-suppressed individuals, suggesting that this may be a key feature of HIV latency in primary CD4^+^ T cells. Blocks to HIV transcriptional initiation and elongation were observed more variably among models. A common set of 234 cellular genes, including members of the minor spliceosome pathway, was differentially expressed between unstimulated and activated cells from primary cell models and ART-suppressed individuals, suggesting these genes may play a role in the blocks to HIV transcription and splicing underlying latent infection. These genes may represent new targets for therapies designed to reactivate or silence latently-infected cells.

## Author summary

The ability of HIV to establish a reversibly silent (latent) infection in CD4+ T cells is widely regarded as the main barrier to curing HIV, but it is unclear what mechanisms govern latent HIV infection. To investigate these questions, we compared the HIV and cellular transcription profile in three primary cell models of HIV latency and cells from the blood of HIV-infected individuals. A reversible block to HIV RNA splicing was common to all primary cell models and cells from HIV-infected individuals, suggesting that this may be a key mechanism of latent HIV infection. Blocks at other stages of HIV transcription were observed more variably among models. Differential expression of 234 human genes, including members of the minor spliceosome pathway, distinguished latently-infected from reactivated (non-latently infected) CD4+ T cells in primary cell models and HIV-infected individuals. These genes may play a role in the blocks to HIV transcription and splicing underlying latent infection and may represent new targets for therapies aimed at curing HIV.

## Introduction

The ability of HIV to establish latent infection in CD4^+^ T cells is regarded as the main barrier to curing HIV, but despite two decades of research, it is unclear what mechanisms govern latent HIV infection *in vivo* [1]. Multiple mechanisms may contribute to latency and may differ by cell and tissue type. We recently demonstrated that a series of blocks to HIV transcriptional elongation, completion, and splicing are the main mechanisms that reversibly inhibit HIV expression in peripheral blood mononuclear cells (PBMC) and CD4^+^ T cells from the blood of ART-suppressed individuals [2], whereas gut CD4^+^ T cells showed a much stronger block to HIV transcriptional initiation [3]. However, these studies could not determine whether the same mechanisms operate in the rare subset of cells with infectious proviruses, or which human cellular factors govern the blocks to different stages of HIV transcription. There is no known method to answer these questions using CD4+ T cells from HIV-infected individuals because there are no known phenotypic markers to distinguish latently-infected from uninfected cells or cells infected with defective or non-inducible proviruses without T cell activation, which reverses latency. Therefore, *in vitro* models have been developed to facilitate the study of HIV latency.

The earliest models consisted of latently-infected clonal cell lines, which have advantages such as cost, ease of use, infection frequency, and known HIV sequence and integration site. However, we recently observed that the blocks to HIV expression differ between these cell lines and CD4^+^ T cells from ART-suppressed individuals, possibly because of their transformed, continuously proliferating state, single or limited integration sites, and (in some cell lines) defects in Tat, TAR, or Nef [4]. In an attempt to overcome these limitations, multiple laboratories have developed primary cell latency models based on *in vitro* infection of cells from uninfected donors [5]. The various primary cell models differ in many important aspects, including the source of T cells, cell state upon infection, virus used for infection, methods used to establish and/or select for latent infection, and cell populations analyzed [5]. While widely considered to be more “physiologic” than cell lines, it is unclear what mechanisms govern latency in each of these primary cell models, or the degree to which they recapitulate latency *in vivo*.

To investigate these questions, we compared the blocks to HIV expression in three primary cell models (Table 1) to that of peripheral CD4^+^ T cells from HIV-infected ART-suppressed individuals [2,6–9]. We have referred to each primary cell model by the virus used or the cell state upon infection. The three models were chosen to include variation in: 1) source of T cells (peripheral total CD4+ T cells or central memory CD4+ T cells, total tissue [tonsil] cells); 2) activation status upon infection (activated or non-activated); 3) virus used for infection (replication-incompetent laboratory virus [HIV_GKO_], replication-competent laboratory virus with reporters [_mCherry-Luc_NL4.3], replication-competent wild type laboratory virus [_wt_NL4.3], or infectious viruses isolated from ART-suppressed individuals using Quantitative Viral Outgrowth Assays [QVOA]); and (iv) cell populations analyzed (sorted latently- and productively-infected, versus mix of uninfected and latently-infected cells that can be reactivated with αCD3/αCD28).

**Table 1:** Properties of the primary cell models of HIV-1 latency used in this study.

Model	Source of T-cells	Cell type upon infection	Virus/vector	Replication competence	ARVs in culture	Cell populations analyzed	Readout	Time between infection and readout
Dual-reporter virus [9]	Peripheral total CD4^+^ T cells	αCD3/αCD28 activated *in vitro*	HIV_GKO_ (ΔNef-csGFP-EF1α-mKO2)	Incompetent	NA	Latent (GFP^-^KO2^+^) Productive (GFP^+^KO2^+^) DN (GFP^-^KO2^-^)	HIV-1 transcription profile [2]	Day 3, 5 and 10
Resting-cell [7]	Peripheral total CD4^+^ T cells	Non-activated *in vitro*	_mCherry-Luc_NL4.3 (ΔNef-mCherry-Luc)	Competent	SQV (after infection) + RAL (after reactivation)	Non-stimulated αCD3/CD28 stimulated	Luciferase HIV-1 transcription profile [2]	Day 5 and 7 (48 hours after stimulation)
	Tonsil total CD4^+^ T cells							Day 7 (48 hours after stimulation)
Wild-type virus [8]	Peripheral naïve CD4^+^ T cells	T_CM_ cytokine-polarized αCD3/αCD28 activated *in vitro*	_wt_NL4.3	Competent	RAL + NFV (day6 after infection)	Non-stimulated αCD3/CD28 stimulated	% p24^+^ cells HIV-1 transcription profile [2]	Day 10 and 12 (48 hours after stimulation)
					NFV + AMD (day6 after infection)			
			QVOA isolate					

T_CM_, T central memory cells; wt, wild type; SQV, saquinavir; RAL, raltegravir; NFV, nelfinavir; DN, Double Negative.

The “Wild-type” virus model is a modification of the original Bosque/Planelles model of latency [6,8,10], which establishes viral latency in cytokine-polarized peripheral central memory CD4^+^ T cells (T_CM_) that have been differentiated from peripheral naïve CD4+ T cells using TGF-β and monoclonal antibodies to IL-4 and IL-12. Activated T_CM_ cells are infected using spinoculation with replication-competent laboratory viruses, such as wild type NL4.3 (_wt_NL4.3), or (in this work) latent viruses isolated from ART-treated individuals using QVOA. Three days after infection, cells are crowded to increase cell-to-cell HIV transmission for an additional 3 days, and then antiretroviral drugs (ARVs) are added to stop spreading infection. After 4 days with ARVs, CD4^+^ T cells are sorted and the frequency of latently-infected cells can be measured by activation followed by flow cytometry to detect intracellular Gag (p24) protein. This model allows the generation of 1–10% latently-infected cells.

The “Resting-cell” model [7,11,12] establishes HIV infection by spinoculation of non-activated total peripheral CD4^+^ T cells or tonsil cells. This model uses a replication-competent laboratory virus (_mCherry-Luc_NL4.3) containing two reporters in the *nef* reading frame (mCherry and Luciferase) that are used to monitor for productive infection. After 5 day-culture in the presence of a protease inhibitor, at which time there is minimal constitutive expression of the reporters, cells can be activated or treated with latency reversing agents (LRAs) and induction of HIV expression can be quantified by mCherry expression and/or Luciferase activity. This assay permits the generation of 5–10% latently-infected cells.

The “Dual-reporter” virus model [9] establishes HIV infection by spinoculation of αCD3/αCD28 activated total peripheral CD4^+^ T cells. This model uses a replication-incompetent virus (HIV_GKO_) derived from NL4.3 in which two fluorescent reporters have been inserted into the *nef* reading frame: csGFP (transcribed from the viral Long Terminal Repeat [LTR] promoter, and used as a reporter for HIV expression) and mKO2 (inserted with a separate, constitutively-active EF1a promoter, and used to identify cells containing the provirus). After 5 day-culture, flow cytometry is used to sort and quantify cells that are productively-infected (defined as csGFP^+^mKO2^+^), latently-infected (csGFP^-^mKO2^+^), and double negative (csGFP^-^mKO2^-^, mix of uninfected cells and latently-infected cells that have lost expression of mKO2). This model is fundamentally different from the other two models in that it allows the establishment and analysis of pure latently- and productively-infected populations without the need for an additional stimulation step.

Given the methodologic differences between these primary cell models and the fact that they show varying response to LRAs [13], we hypothesized that different mechanisms may govern HIV latency in each model. To measure the reversible blocks to HIV transcription in each model, we quantified the levels of different HIV RNA regions representing progression through different blocks to HIV transcription in latently-infected and activated (Resting-cell, Wild-type models) or productively-infected (Dual-reporter model) cells from each model and compared them to unstimulated or *ex vivo* activated blood cells from ART-suppressed individuals. Though differences were observed between each model and cells from ART-suppressed individuals, all models recapitulated the reversible block to HIV multiple splicing observed in cells from ART-suppressed individuals. To investigate the human cellular factors involved in the blocks to HIV transcription and splicing, RNA-seq was used to compare the genes that were differentially expressed between latently-infected and activated cell populations in the Resting-cell model, Wild-type model, and CD4^+^ T cells from ART-suppressed individuals. We found a common set of 234 genes, including a subset of genes involved in splicing, that were differentially expressed between latent and re-activated cells from both models and CD4^+^ T cells from ART-suppressed individuals.

## Results

**Fig 1:**
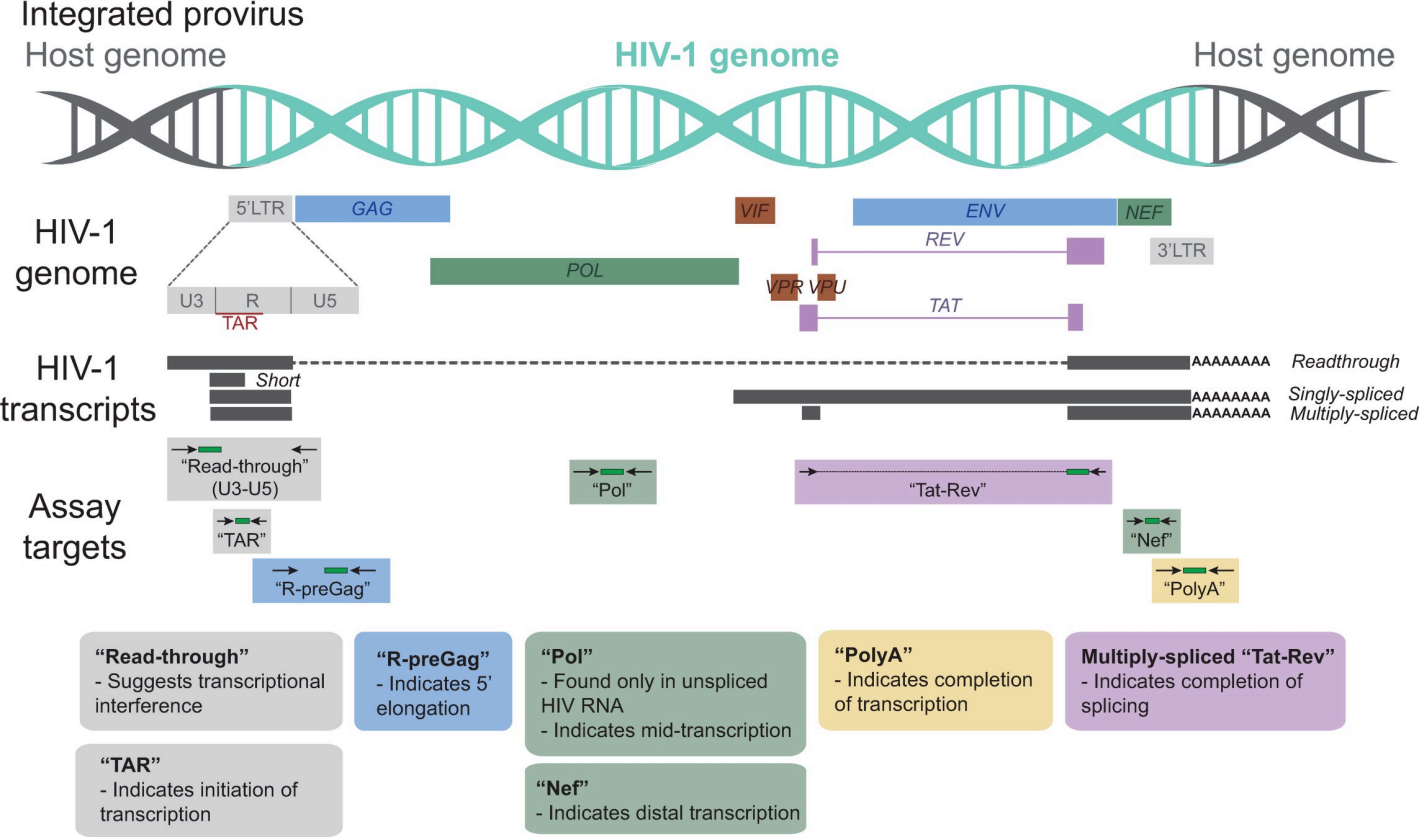
HIV genome and the targets for HIV transcription profiling assays. This schematic shows the genetic organization of proviral HIV DNA and the HIV “transcription profiling” assays targeting specific HIV RNA sequence regions that provide insight into blocks to transcription.

To measure the degree to which HIV expression is blocked by mechanisms operating at different stages of transcription, we used a panel of RT-ddPCR assays (Fig 1) to quantify HIV RNA regions suggesting transcriptional interference (read-through) and HIV transcriptional initiation (TAR), 5’ elongation (R-pre-Gag), mid transcription (Pol; also indicates unspliced), distal transcription (Nef), polyadenylation (U3-polyA), and multiply-splicing (Tat-Rev) in latently-infected and productively-infected or activated (αCD3/αCD28) cells from each primary cell model (Table 1) and peripheral CD4^+^ T cells from ART-suppressed individuals (n = 14; S1 Fig). For each model, cells from 2–5 donors were used for infection.

### In the Wild-type model, HIV latency is regulated mostly by blocks to HIV transcription initiation and multiple splicing

**Fig 2:**
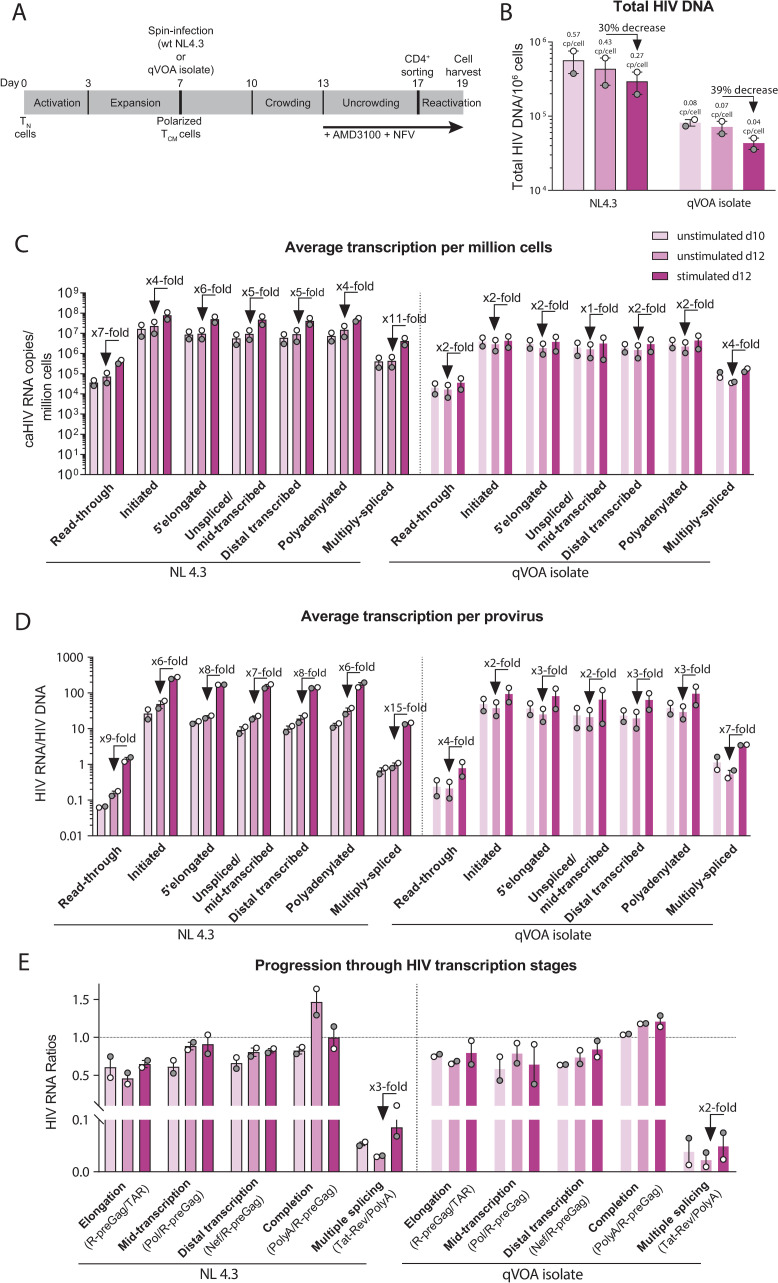
The Wild-type model: HIV latency is regulated by blocks to initiation, multiple splicing, and possibly elongation. (A) Diagram of the model, (B) total HIV DNA, (C) level of HIV transcripts per million cells, (D) level of HIV transcripts per provirus, (E) progression through HIV transcription stages. Individual values per donor (dots), and median and range (bars) are shown. Unstimulated cells (days 10 and 12 post-infection) are shown in light colors and stimulated cells (day 12) in dark color.

The latest iteration of the Wild-type model (Fig 2A) uses _wt_NL4.3 HIV or viruses isolated from QVOA to infect polarized CD4^+^ T central memory (T_CM_) cells from HIV-negative donors and adds AMD3100 and nelfinavir (NFV) at day 6 after infection (2 donors for each virus). Total HIV DNA levels were quantified in unstimulated cells at day 10 or 12 after infection, and in stimulated (activated) cells at day 12 (Fig 2B), using ddPCR. As inferred from HIV DNA levels, the median infection frequencies were 57% at d10, 43% at d12, and 29% after stimulation at d12 when using _wt_NL4.3, versus 8% at d10, 7% at d12 and 4% after stimulation when using virus from QVOA. Therefore, the total HIV DNA decreased over time in culture (d12/d10 = 0.75 with _wt_NL4.3 and 0.87 with QVOA isolate) and decreased after activation (d12 stimulated/unstimulated = 0.70 with _wt_NL4. and 0.61 with QVOA), suggesting a cytopathic effect on latently-infected cells that is enhanced after activation.

Levels of different HIV RNA regions (Fig 1) were measured by RT-ddPCR and expressed as copies per million cells (Fig 2C). To account for differences in infection frequency, levels of each HIV RNA were divided by the total HIV DNA to express the average level of each transcript per provirus (Fig 2D) in each cell population. HIV RNA levels per million cells and per provirus of all HIV transcripts were similar in unstimulated cells from d10 and d12 (Fig 2C and 2D). Compared to unstimulated cells from d12, activated cells showed successively higher levels of initiated, 5’elongated, and multiply-spliced HIV transcripts (6-, 8- and 15-fold higher with _wt_NL4.3, vs. 2-, 3- and 7-fold higher with QVOA virus, respectively). To measure the progression through each block to HIV transcription, ratios of one HIV RNA region to another were used to calculate the degree of 5’elongation (5’elongated/initiated), mid transcription (Pol/5’elongated), completion (polyadenylated/5’elongated) and multiple splicing (multiply-spliced/polyadenylated). As measured by the ratio of one HIV RNA region to another, we observed a 1.4-fold (_wt_NL4.3) or 1.2-fold (QVOA isolate) increase in elongation and a 3-fold (_wt_NL4.3) or 2-fold (QVOA isolate) increase in multiple splicing when comparing stimulated and unstimulated cells at d12 (Fig 2E). These results suggest that in the Wild-type model, HIV latency is regulated mostly by reversible blocks to initiation, multiple splicing, and possibly elongation.

We also analyzed the original version of the Wild-type model, which uses the _wt_NL4.3 HIV but adds raltegravir (RAL) and nelfinavir (NFV) at day 6 after infection (n = 3 donors; S2A Fig). The median total HIV DNA was 5.4×10^6^ copies/million unstimulated cells at day 10, 3.2×10^6^ copies/million unstimulated cells at day 12, and 2.6×10^6^ copies/million stimulated cells at day 12, indicating an average of 2.6–5.4 copies of HIV DNA per cell (S2B Fig). To determine whether the HIV DNA corresponds to integrated or unintegrated forms, we also measured the integrated HIV DNA [14]. The median integrated HIV DNA was 1.5×10^6^ copies/million unstimulated cells at day 10, 1.5×10^6^ copies/million unstimulated cells at day 12, and 1.3×10^6^ copies/million stimulated cells at day 12, indicating an average of 1.3–1.5 integrated proviruses per cell The excess of total over integrated HIV DNA suggests that much of the HIV DNA consists of unintegrated forms (73% of total HIV DNA in unstimulated cells at day 10, 31% in unstimulated cells at day 12, and 53% in stimulated cells at day 12), likely due to the use of the integrase inhibitor raltegravir. The total HIV DNA decreased more over time in culture (d12/d10 = 0.30) than the integrated HIV DNA (d12/d10 = 0.89), suggesting loss of unintegrated HIV DNA with cell division or greater cytopathic effects in cells with more unintegrated HIV DNA (S2B Fig). In contrast, activation caused similar small decreases in total and integrated HIV DNA (d12 stimulated/unstimulated = 0.89 and 0.87, respectively).

The levels of HIV transcripts per provirus (normalized to total HIV DNA) were lower in unstimulated cells at d12 than at d10, while activation caused successive 13-, 14-, 8-, 19-, 10-, and 186-fold increases in initiated, 5’ elongated, unspliced, distal transcribed, polyadenylated, and multiply-spliced transcripts, respectively (S2C Fig). As measured by the ratios of one HIV RNA to another, which are independent of HIV DNA quantification, activated cells showed higher levels of multiple splicing (17-fold higher than unstimulated cells) and possibly distal transcription (1.3-fold higher). Overall, these results suggest the use of an integrase inhibitor in the Wild-type model leads to accumulation of unintegrated forms of DNA and changes the regulation of HIV transcription, with apparent blocks to initiation and multiple splicing but not elongation.

### In the Resting-cell model, HIV latency is regulated mostly by a block to multiple splicing in peripheral CD4^+^ T cells and blocks to transcription initiation and multiple splicing in tonsil cells

**Fig 3:**
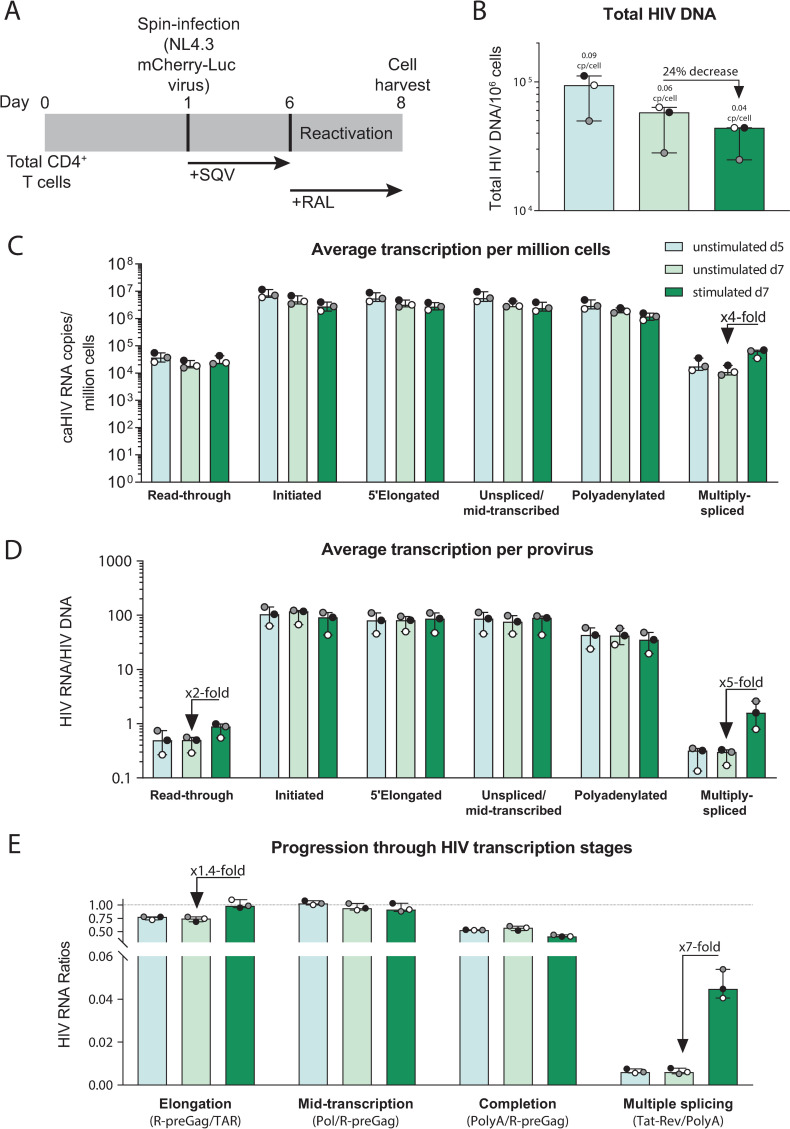
The Resting-cell model: HIV latency is regulated mostly by a block to multiple splicing. (A) Diagram of the model, (B) total HIV DNA, (C) level of HIV transcripts per million cells, (D) level of HIV transcripts per provirus, (E) progression through HIV transcription stages. Individual values per donor (dots), and median and range (bars) are shown. Unstimulated cells (days 5 and 7 post-infection) are shown in light colors and stimulated cells (day 7) in dark color.

The Resting-cell model (Fig 3A) uses the _Cherry-Luc_NL4.3 HIV [ΔNef-mCherry-Luc] virus to quantify productive HIV infection by mCherry expression or luciferase activity. After infection of donor CD4^+^ T cells from blood, total HIV DNA levels were quantified in unstimulated cells at day 5 or 7 after infection, and in stimulated (activated using αCD3/αCD28) cells at day 7 (Fig 3B). The median infection frequency (assuming ≤1 provirus/cell) was 9% at d5, 6% at d7, and 4% after stimulation at d7. Therefore, HIV DNA decreased over time in culture (d7/d5 = 0.57) and after activation (d7 stimulated/unstimulated = 0.76), suggesting a cytopathic effect in latently-infected peripheral CD4^+^ T cells that increases after activation. HIV RNA levels per million cells (Fig 3C) and per provirus (HIV RNA/HIV DNA; Fig 3D) of all HIV transcripts were similar in unstimulated cells at d5 and d7. Compared to the unstimulated (latently-infected) cells at d7, the stimulated (activated) cells differed only in having higher levels per provirus of multiply-spliced HIV transcripts. As measured by ratios of one RNA region to another, the activated cells showed 1.4-fold higher levels of HIV transcriptional elongation and 7-fold higher levels of multiple splicing when compared to the unstimulated cells at d7 (Fig 3E). These results suggest that in peripheral CD4^+^ T cells infected using the Resting-cell model, HIV latency is regulated mostly by a reversible block to multiple splicing.

To investigate whether the mechanisms that regulate HIV latency differ between donor cells from the blood and tissues, we generated the Resting-cell model by infecting CD4^+^ T cells from tonsils from two donors (S3 Fig). Compared to the unstimulated tonsil cells from day 7, the stimulated (activated) cells showed successively higher levels per million cells of initiated HIV transcripts (3-fold higher) and multiply-spliced HIV transcripts (7-fold higher; S3A Fig), suggesting that latency may be governed by blocks to initiation and multiple splicing in tonsil cells from this model. As measured by the ratio of multiply-spliced/polyadenylated HIV RNA, the activated cells showed 3-fold higher levels of HIV multiple splicing than the unstimulated cells at d7 (S3B Fig). These results suggest that in the Resting-cell model, the mechanisms of latency may vary based on the tissue source of the CD4^+^ T cells, with a block to HIV splicing in cells from both blood and tonsil, but an additional block to HIV transcriptional initiation in cells from the tonsil.

While we washed the cells multiple times after infection, it is possible that surface-bound virions persist and that their genomic RNA contributes to the measured cell-associated HIV RNA, which could obscure blocks to HIV transcription initiation, elongation, or completion. The presence of surface bound virions might be more likely in the resting cell model than the other two models, since the resting cell model involves a shorter duration of culture after infection and does not incorporate a subsequent sorting step. To assess the effect of surface bound virions in the resting cell model, we performed this model with and without addition of pronase digestion (which should help remove surface virions) after spinoculation of peripheral CD4+ T cells from 3 additional donors (S4 Fig). Compared to the non-pronase treated cells, the pronase treated cells showed lower total HIV DNA per million cells, suggesting that the pronase treatment reduced the number of infected cells. The pronase treated cells also showed lower levels of HIV transcription per million cells and per provirus (S4C and S4D Fig), suggesting that the pronase treatment may have eliminated some bound virions and/or reduced HIV transcriptional initiation. However, when comparing the progression through HIV transcription stages, the levels of elongation, mid-transcription, completion, and multiple splicing did not appear to differ between pronase treated and non-treated cells (S4E Fig), suggesting that any pronase-mediated reduction of bound virions did not reveal new blocks at these other stages of HIV transcription.

### In the Dual-reporter model, HIV latency is regulated by donor-specific blocks to initiation, elongation, and multiple splicing

**Fig 4:**
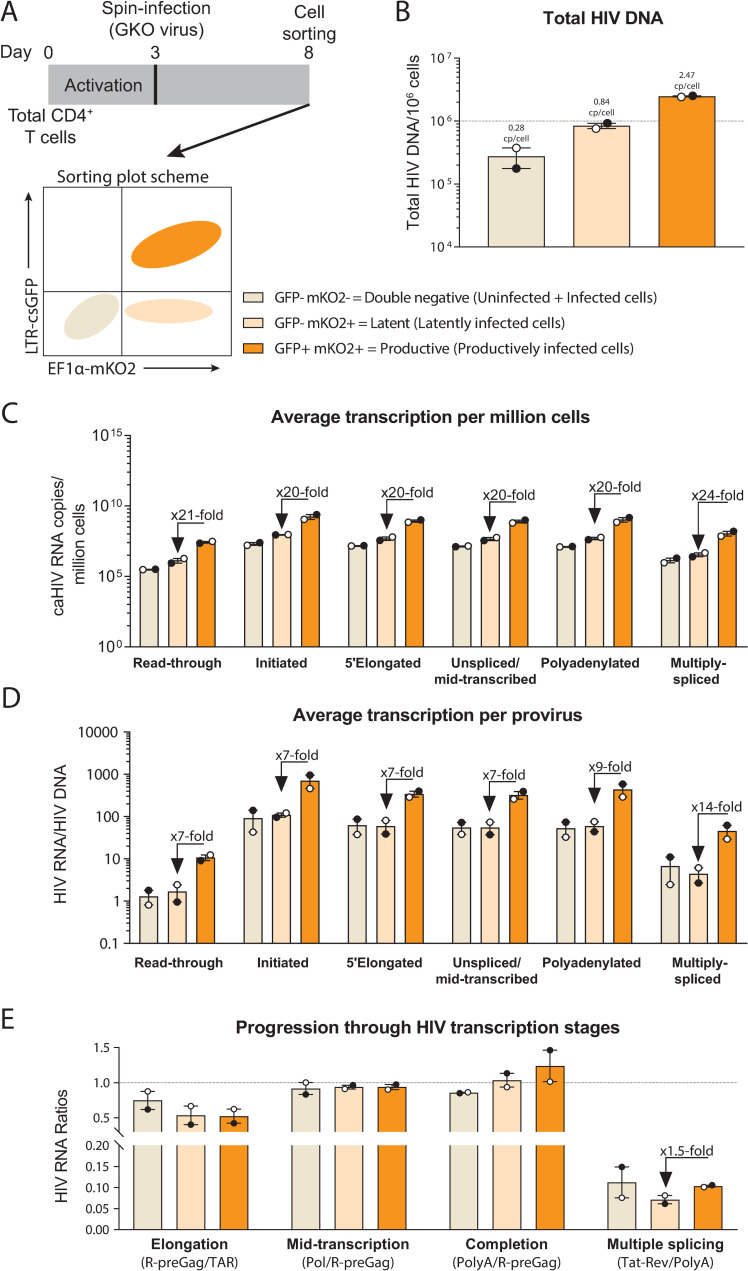
The Dual-reporter model: HIV latency is regulated by blocks to initiation and multiple splicing. (A) Diagram of the model, (B) total HIV DNA, (C) level of HIV transcripts per million cells, (D) level of HIV transcripts per provirus, (E) progression through HIV transcription stages. Individual values per donor (dots), and median and range (bars) are shown. Double negative and latent cells are shown in light colors and productive cells in dark color.

The Dual-reporter model (Fig 4A) uses the HIV_GKO_ [ΔNef-csGFP-EF1α-mKO2] dual reporter virus and allows for sorting of latently-infected [GFP^-^mKO2^+^], productively-infected [GFP^+^mKO2^+^], and double negative [GFP^-^mKO2^-^] (mix of infected and uninfected) populations. Total HIV DNA levels were measured in each cell population (Fig 4B). The double negative population showed a median of 2.8×10^5^ HIV DNA copies/million cells, suggesting an infection frequency of 28%. The latently-infected population harbored a median of 8.4×10^5^ HIV DNA copies/million cells, suggesting that as expected, almost every cell harbors a provirus. In contrast, the productively-infected population showed a median of 2.5×10^6^ HIV DNA copies/million cells, suggesting that the average productively-infected cell harbors more than one provirus. To confirm these results and determine whether we were detecting unintegrated forms of HIV DNA, we also measured the integrated HIV DNA using a published *Alu* qPCR assay [14]. The integrated HIV DNA was 2.3×10^6^ copies/million cells in the productively-infected population, confirming that the productively-infected cells from these donors harbor more than one integrated provirus per cell.

HIV RNA levels per million cells and per provirus of each HIV transcript were similar in the double negative and latently-infected populations (Fig 4C and 4D). Compared to the latently-infected cells, the productively-infected cells showed successively higher levels of initiated (TAR) and multiply-spliced (Tat-Rev) HIV transcripts per provirus (7- and 14-fold higher, respectively), suggesting that blocks to HIV transcriptional initiation and multiple splicing contribute to latency in this model. As measured by the ratio of multiply-spliced/polyadenylated HIV transcripts, HIV multiple splicing was 1.5-fold higher in the productive compared to the latent cells (Fig 4E). Together, these results suggest that HIV latency in the Dual-reporter model is regulated by blocks to initiation and multiple splicing.

To determine whether these mechanisms vary with time after infection in the Dual-reporter model, we quantified the levels of HIV transcripts and the progression through HIV transcription stages 3 or 10 days after infection in one donor (S5A and S5B Fig). The double negative and latent populations showed similar level per provirus of each HIV transcript. Compared to the latently-infected cells, the productively-infected cells showed successively higher levels of initiated and 5’elongated transcripts (5- and 8-fold higher at d3, and 7- and 13-fold higher at d10, respectively), suggesting differences in initiation and elongation in the cells from this particular donor. When the progression through HIV transcriptional stages was measured by ratios of one HIV RNA to another (S5C and S5D Fig), the productively-infected cells showed 2-fold higher levels of elongation when compared to the latently-infected cells. These results suggest there might be time- or donor-specific mechanisms that regulate HIV latency in this model, but overall, the differences in HIV transcriptional initiation were maintained across donors and time points. Therefore, HIV latency in the Dual-reporter model is regulated mostly by a block to initiation, with additional blocks to elongation and/or multiple splicing in a donor- or time-dependent manner.

### The reversible block to multiple splicing is a common feature of all three models and peripheral CD4^+^ T cells from ART-suppressed individuals

**Fig 5:**
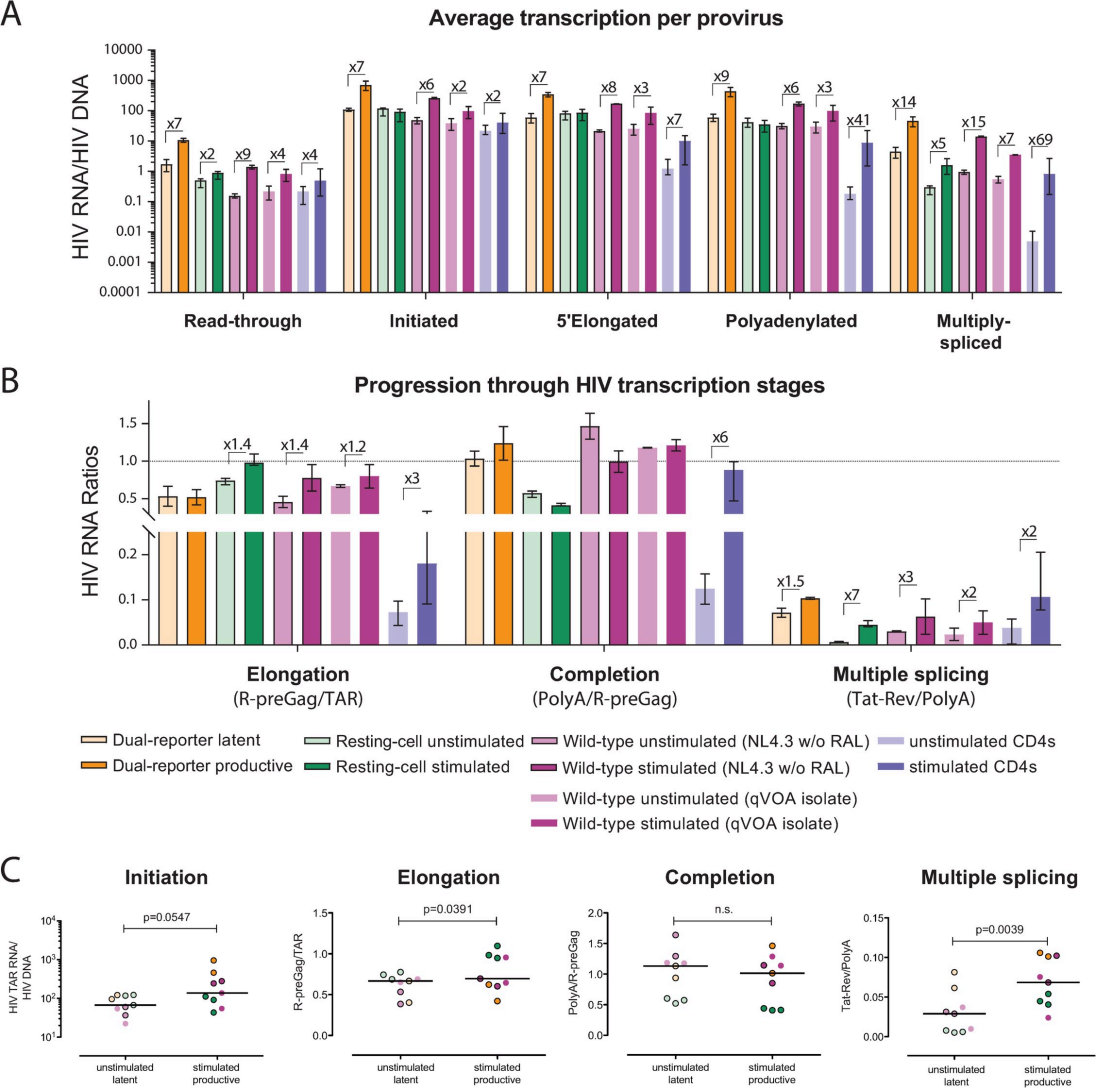
Primary cell HIV latency models recapitulate the block to multiple splicing observed in CD4^+^ T cells from ART-suppressed individuals. Comparison of (A) Level of HIV transcripts per provirus, and (B) progression through HIV transcription stages in cells from the Dual-reporter (orange), the Resting-cell (green) and the Wild-type (purple) primary cell models, and cells from ART-suppressed individuals (blue), in latently- (light color) and productively-infected or stimulated cells (dark color). Median and range (bars) are shown. (C) Comparison of HIV transcriptional initiation, elongation, completion, and multiple splicing in the latent (unstimulated) and productive (stimulated) populations in all donors from the three main models shown in Figs 2–4. Bars indicate the median; P-values were calculated using the Wilcoxon signed rank test.

To evaluate how the mechanisms that regulate HIV transcription in each model correspond to those that operate *in vivo*, we compared the results from the main experiments performed in each model and peripheral CD4^+^ T cells from HIV-infected ART-suppressed individuals (n = 14; Fig 5). Compared to unstimulated CD4+ T cells from ART-suppressed individuals, latently-infected or unstimulated cells from each model showed higher baseline levels of HIV initiated, 5’elongated, polyadenylated, and multiply-spliced transcripts per provirus (p<0.05 for all transcripts and models; Mann-Whitney test).

In cells from ART-suppressed individuals, activation caused successive increases in initiated, 5’elongated, polyadenylated, and multiply-spliced HIV RNA per provirus (2-, 7–41- and 69-fold increases), suggesting reversible blocks to initiation, elongation, completion, and multiple splicing (Fig 5A). In the Dual-reporter model, we observed successive increases in initiated and multiply-spliced HIV RNA per provirus (7- and 14-fold increases) in the productive compared to the latent cells, suggesting blocks to initiation and multiple splicing. In the Resting-cell model, activation primarily increased the multiply-spliced HIV RNA per provirus (5-fold), suggesting a reversible block to multiple splicing. In the Wild-type model, activation caused successive increases in initiated, 5’elongated, and multiply-spliced HIV RNA per provirus (6-, 8- and 15-fold increase using _wt_NL4.3, and 2-, 3- and 7-fold increase using QVOA isolate), suggesting reversible blocks to initiation, multiple-splicing, and possibly elongation.

As measured by HIV RNA ratios, we observed 3-, 6- and 2- fold increases in elongation, completion, and multiple splicing in peripheral CD4^+^ T cells from ART-suppressed individuals, while we observed a 1.5-fold increase in multiple splicing in the Dual-reporter model, 1.4- and 7-fold increases in elongation and multiple splicing in the Resting-cell model, and 1.4- (_wt_NL4.3) or 1.2- (QVOA isolate) fold increases in elongation and 3- (_wt_NL4.3) or 2-fold (QVOA) increases in multiple splicing in the Wild-type model (Fig 5B). Hence, all three primary cell HIV latency models differ from each other and from CD4^+^ T cells from ART-suppressed individuals in the degree to which HIV transcription differs between the latently-infected/unstimulated and productively-infected/activated cells, and therefore, in the likely mechanisms that govern HIV latency (S1 Fig).

When compared across the donors for all 3 primary cell models shown in Figs 2–4, HIV multiple-splicing (multiply-spliced/polyadenylated HIV RNA) was significantly higher in the stimulated or productively-infected cells compared to the unstimulated or latently-infected cells (P = 0.0039; Wilcoxon-signed rank test; Fig 5C). HIV transcriptional elongation (elongated/initiated HIV RNA) was also higher in the stimulated or productively-infected cells (P = 0.039), although the difference in medians was small. When we included all the donors used in the main models plus additional experiments shown in the supplementary figures, the productively-infected cells showed significantly higher levels of initiation, elongation, and multiple-splicing (S6 Fig). However, the largest and most consistent difference across all models (12 of 13 total donors) and HIV-infected individuals was that the stimulated or productively-infected cells had higher levels of HIV multiple splicing, suggesting that a reversible block to HIV multiple splicing is a conserved mechanism of latent HIV infection in primary CD4^+^ T cells.

### snRNAs and snRNPs involved in the minor spliceosome pathway may play a role in reversing the block to HIV multiple splicing

To identify human cellular genes that may regulate the blocks to HIV transcription and splicing, we compared the transcriptome (bulk RNA-Seq) of unstimulated and stimulated peripheral CD4^+^ T cells from the Resting-cell model (2 donors, 17–22×10^6^ reads/sample), the Wild-type model (2 donors, 13–19×10^6^ reads/sample), and CD4^+^ T cells from the blood of 2 ART-suppressed individuals (12–19×10^6^ reads/sample). The dual reporter model was not included in this analysis because of the fundamental ways in which it differs from these other models or cells from ART-suppressed individuals, including the fact that it generates pure populations of infected cells and that the productively infected cells constitutively express viral proteins in the absence of an extra stimulation step.

In the Resting-cell model, in which HIV latency is regulated mostly by a reversible block to splicing, the latently-infected and activated populations differed in expression of 694 cellular genes (FDR<0.05), including 17 cytokines or growth factors, 36 transcription factors, 10 cell differentiation markers, 16 protein kinases, 14 long-non-coding RNAs (lncRNAs), and 20 factors involved in RNA metabolism (S1 Table). Of the genes involved in RNA metabolism, 17 correspond to genes related to mRNA processing and/or splicing: 9 small nuclear RNAs (RNU1-122P, RNU4ATAC, RNVU1-7, RNU6-789P, RNU1-27P, RNU6-606P, RNU6-808P, RNU6-761P, and RNU4ATAC11P), 4 mRNA processing factors (MAGOH, PPWD1, CCNL2 and PAPOLA), 2 small nuclear ribonucleoproteins (snRNPs) (SNRNP25 and SNRPD2), 1 helicase (DDX17), and 1 nuclease/transcription factor that encodes for the uncharacterized protein C3orf67. Of those 17 genes, 4 were related to the minor mRNA splicing pathway (3 up-regulated: RNAU4ATAC [7.8-log_2_fold increase], SNRNP25 [3.7-log_2_fold increase] and SNRPD2 [1.2-log_2_fold increase], and 1 down-regulated: RNU4ATAC11P [-1.8-log_2_fold decrease]), suggesting that the minor spliceosome pathway may play a role in reversing the block to HIV multiple splicing.

In the Wild-type model, in which HIV latency is regulated mostly by reversible blocks to initiation and splicing, the latently-infected and activated populations differed in expression of 8914 cellular genes (FDR<0.05), including 66 cytokines or growth factors, 450 transcription factors, 111 cell differentiation markers, 187 protein kinases, 165 lncRNAs, and 422 factors involved in RNA metabolism (S2 Table). In peripheral CD4^+^ T cells from ART-suppressed individuals, the unstimulated and activated cells differed in expression of 9699 cellular genes (FDR<0.05), including 98 cytokines or growth factors, 477 transcription factors, 140 cell differentiation markers, 194 protein kinases, 195 lncRNAs, and 440 factors involved in RNA metabolism (S3 Table).

**Fig 6:**
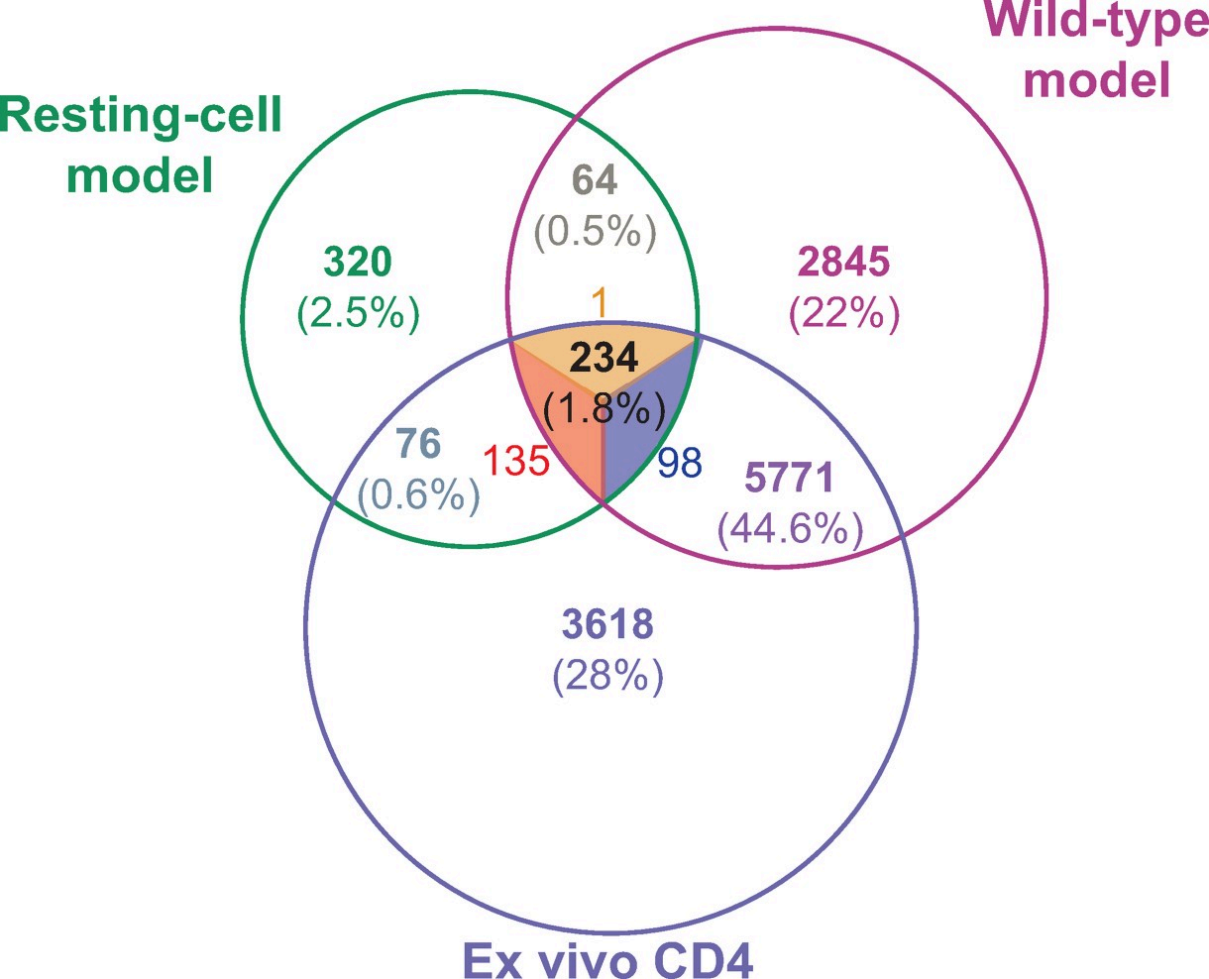
Transcriptome analysis of the Resting-cell model, Wild-type model, and cells from ART-suppressed individuals. Venn diagram showing the number of differentially expressed genes between unstimulated and stimulated peripheral CD4^+^ T cells from the Resting-cell model, the Wild-type model (infection using _wt_NL4.3 HIV and no raltegravir), and ART-treated individuals (2 donors/model). Up-regulated genes are shown in red, down-regulated in blue, and discordant genes in orange.

To determine similarities between the models and HIV infection *in vivo*, we assessed for overlap in the differentially expressed genes (FDR<0.05) from the Resting-cell and Wild-type models and cells from ART-suppressed individuals. A total of 234 genes were differentially expressed in all three comparisons: 135 up-regulated and 98 down-regulated in all three, and 1 up-regulated in the Wild-type model and ART-treated individuals but down-regulated in the Resting-cell model (Fig 6, S4 Table). Of those 234 genes, 7 correspond to cytokines and growth factors, 17 to transcription factors, 5 to cell differentiation markers, 6 to lncRNAs, and 10 to factors involved in RNA metabolism. Six genes had a previously-described interaction with HIV, of which 3 were also related to the metabolism of RNA (SEC13, PSMD14 and NUP155) (S4 Table). Two of the 10 genes involved in RNA metabolism correspond to snRNPs. Both are related to the minor spliceosome pathway (SNRNP25 and SNRPD2) and both were up-regulated in stimulated cells independently of the model, suggesting that the minor spliceosome pathway might be involved in the reversible block to HIV splicing. Only 76 differentially expressed genes were common to both the Resting-cell model and CD4^+^ T cells from ART-suppressed individuals, while 5771 differentially expressed genes were common to the Wild-type model and ART-suppressed individuals.

## Discussion

This study is the first to characterize the mechanisms that govern HIV latency in several widely used primary cell models and compare them to the mechanisms that reversibly inhibit HIV expression in peripheral CD4^+^ T cells from ART-suppressed individuals. Moreover, this study represents the first RNA-seq characterization of the Resting-cell primary cell HIV latency model. All three primary cell models differed from each other and from cells from ART-suppressed individuals. However, a common feature of all three models and *in vivo* infected cells was a reversible block to HIV multiple splicing, suggesting that this is a conserved mechanism of latent HIV infection in primary CD4^+^ T cells. The RNA-Seq results from the Resting-cell model, in which HIV latency in peripheral CD4+ T cells is regulated mostly by a reversible block to splicing, showed that snRNAs and snRNPs involved in the minor spliceosome pathway may play a role in reversing the block to HIV multiple splicing.

The Wild-type model has the advantage that it uses unmodified, infectious viruses and has been implemented with both laboratory viruses and viruses derived from HIV-infected individuals. Using either laboratory viruses or patient isolates, HIV latency in the Wild-type model appears to be regulated by blocks to HIV transcription initiation, multiple splicing, and possibly elongation. In terms of the reversible blocks to HIV transcription, this model appears to be closest to peripheral cells from HIV-infected ART-suppressed individuals [2], perhaps due to the use of unmodified viruses. Nonetheless, when this model was implemented using an integrase inhibitor (per the original protocol), we detected more than one HIV DNA molecule per cell but approximately one copy of integrated HIV DNA per cell, suggesting the accumulation of unintegrated forms of HIV DNA. When used with the integrase inhibitor, we also observed a greater reversibility in the blocks to initiation, elongation, distal transcription, and multiple splicing. As previously reported [15], HIV can be transcribed from unintegrated and integrated forms of HIV DNA, so it is possible that these unintegrated forms of HIV DNA may affect the HIV transcription profile and subsequent experiments testing LRAs or latency-silencing agents.

The Resting-cell model allows for HIV infection without activation, incorporates two markers for productive infection, and has been used with blood as well as tissue (tonsil) cells. In the Resting-cell model using peripheral CD4 T cells, HIV latency appears to be regulated mostly by a block to HIV multiple splicing. Multiply-spliced HIV RNA is used to express the HIV Tat protein, which provides positive feedback to increase HIV transcriptional initiation and elongation, and Tat is likely essential for reactivation from latency. Multiple splicing is the last stage in the HIV transcription process, and multiply-spliced HIV transcripts have been proposed as a surrogate marker of productive infection [16–18]. Therefore, the Resting-cell model might be extremely useful to investigate the regulation of HIV splicing and screen for novel LRAs that can increase multiply-spliced HIV RNA and effectively reverse HIV latency *in vivo*. However, when the Resting-cell model was performed using CD4^+^ T cells from tonsils, we observed a block to HIV transcriptional initiation as well as multiple splicing. These data accord with our recent publication showing lower HIV transcriptional initiation in gut biopsies and CD4^+^ T cells from gut compared to blood of ART-suppressed individuals, and they suggest that intrinsic cellular factors related to the tissue of origin may influence the mechanisms that govern HIV latency [3].

The Dual-reporter model, which uses two separate reporters for the presence of the provirus and HIV expression, has the advantage that it allows isolation of pure populations of productively- and latently-infected cells [9]. When donor CD4^+^ T cells were infected in the Dual-reporter laboratory according to the standard protocol, latently- and productively-infected cells differed in the extent of HIV transcriptional initiation and multiple splicing, suggesting regulation of latency at these two stages of transcription. Productively-infected cells from both donors appeared to harbor an average of two proviruses per cell, which may contribute to higher levels of LTR-driven transcription [15] and splicing in the productively-infected cells. In addition, it is possible that different mechanisms govern expression of the two proviruses. No reversible block was observed to HIV transcriptional elongation or completion, in contrast to what is observed in peripheral CD4^+^ T cells infected *in vivo* [2,3]. It should be noted that transcription from the constitutively-active EF1α promoter may lead to polyadenylated HIV transcripts that do not contain the 5’elongated or mid-transcribed (Pol) regions, which complicates interpretation of the HIV transcription profile and can mask a block to completion but not elongation. At the same time, levels of the 5’elongated, Pol, and U3-polyA transcripts were generally similar, suggesting no significant excess of transcripts from the EF1α promoter. Moreover, a block to elongation was observed in cells from a different donor that were analyzed at different time point, suggesting possible donor or time-specific differences in this model.

Overall, all three primary cell models showed more HIV transcription initiation, elongation, and completion than peripheral cells from ART-suppressed individuals. These results might be explained by different reasons. First, all models are performed over short time courses in which HIV latency is evaluated 7 to 12 days after infection, so these models may resemble early HIV infection *in vivo* more closely than chronic, ART-treated infection. Second, all models infect the cells using spinoculation, which has been reported to enhance HIV infection primarily by sedimenting virions and increasing virion binding to the cell surface [12,19,20]. However, spinoculation increases CD4 and CXCR4 expression, and might increase T cell activation [21], conferring a cellular status more favorable to transcribe HIV. Third, both the Dual-reporter and Wild-type models activate the cells prior to infection and/or culture the cells in the presence of IL-2, and these activation-induced changes might not have resolved by the time the cells were analyzed. Finally, most HIV-infected cells from ART-suppressed individuals contain defective proviruses, and some of these defects may reduce HIV transcriptional initiation (LTRs, Tat), elongation (Tat), or splicing (splice sites, Rev).

Nonetheless, our results suggest that these models can be used to search for cellular genes and test compounds that affect HIV transcription initiation (Dual-reporter model, Wild-type model, and Resting-cell model using tonsil cells) and multiple splicing (all models). To identify cellular genes that may be involved in the regulation of HIV multiple splicing, we compared the cellular transcriptome between unstimulated and stimulated cells from the Resting-cell model, the Wild-type model, and CD4^+^ T cells from ART-suppressed individuals. In all three, unstimulated and stimulated cells differed in expression of a common set of 234 genes, of which 39 encode for cytokines, growth factors, transcription factors, protein kinases or cell differentiation markers, 6 for lncRNAs, 10 for metabolism of RNA factors, and 6 for proteins with known interaction with HIV (3 of which govern metabolism of RNA).

Of the cell “differentiation” markers, programmed cell death 1 (PDCD1 or PD-1) was up-regulated in stimulated cells from the Resting-cell and Wild-type models and ART-suppressed individuals, corroborating previous findings that showed up-regulation of PD-1 in productively HIV-infected cells [22]. We also observed up-regulation of the cluster of differentiation 200 (CD200), a type-1 membrane glycoprotein belonging to the immunoglobulin superfamily, suggesting that this protein may be a marker of productive HIV infection. Both PD-1 and CD200 are expressed in T follicular helper cells (Tfh), which play complex immunological and immunopathogenic roles in HIV infection and constitute one of the major cell subsets for establishment and maintenance of the HIV reservoir [23–25]. Regarding lncRNAs, linc00158 and linc02416 were up-regulated in stimulated cells, while linc00205, linc01138, linc02328 and linc02362 were down-regulated. Prior studies have shown an association between the expression of lncRNAs and HIV infection [26–32]. However, the lncRNAs that we identified have not previously been associated with HIV infection, so further investigation may help to decipher how these lncRNAs regulate HIV latency at the posttranscriptional level.

Of the genes involved in metabolism of RNA, poly(A) polymerase alpha (PAPOLA) was down-regulated, while most other factors were upregulated in stimulated cells from the two models and ART-suppressed individuals, including mago homolog exon junction complex subunit (MAGOH), NIN1 (RPN12) binding protein 1 homolog (NOB1), nucleoporin 155 (NUP155), proteasome 26S subunit non-ATPase 14 (PSMD14), RNA variant U1 small nuclear 7 (RNVU1-7), SEC13 homolog nuclear pore and COPII coat complex component (SEC13), small nuclear ribonucleoprotein U11/U12 subunit 25 (SNRNP25), small nuclear ribonucleoprotein D2 polypeptide (SNRPD2), and tRNA methyltransferase 6 (TRMT6). PAPOLA polyadenylates mRNA, so the down-regulation in transcription of PAPOLA does not explain the increase in polyadenylated HIV transcripts observed in the *ex vivo* stimulated CD4^+^ T cells, which might be driven by an increase in PAPOLA protein activity due to Vpr [33] or cellular proteins. MAGOH interacts with the nonsense-mediated mRNA decay, which promotes nuclear export and translation of unspliced HIV RNA [34,35], while NUP155 and SEC13 are components of the nuclear pores and are necessary for HIV replication after nuclear entry but prior to viral integration [36], so these proteins might also be involved in HIV RNA import, export and/or processing. RNVU1-7, SNRNP25 and SNRPD2 are involved in the spliceosome machinery, and specifically both snRNPs play a role in the minor spliceosome pathway, also known as U12-dependent splicing. U12-type introns represent less than 1% of all introns in human cells [37,38]; however, they are found in genes carrying out essential cellular functions (e.g. DNA replication and repair, transcription, RNA processing and translation) and their removal can be a rate limiting step in the splicing of pre-mRNAs containing both U2- and U12-type introns [39].

It should be noted that the RNA-seq was performed on mixed populations of infected and uninfected cells, and T cell activation can change the expression of many cellular genes, so further studies are necessary to determine the contribution of the differentially expressed genes to HIV expression or latency. However, the RT-ddPCR data showed a reversible block to splicing in the three primary cell models and cells from ART-suppressed individuals, and HIV does not encode any of its own splice factors, so the block to HIV splicing must be driven by human splice factors that are differentially expressed by activation. The RNA seq data reveals a subset of human splice factors that are differentially expressed by activation in two primary cell models of HIV latency as well as cells from HIV-infected individuals, providing strong circumstantial evidence that these factors contribute to the block to HIV splicing. To our knowledge, these findings are the first to associate the minor spliceosome pathway with a direct or indirect regulation of HIV multiple splicing. Nonetheless, further characterization of the specific function of these genes and the role of the minor spliceosome pathway in the regulation of HIV splicing should be analyzed in future studies, such as those that employ techniques to downregulate (CRISP-Cas, siRNA) or upregulate (transfection) gene expression in primary cell models or cells from HIV-infected individuals.

Other limitations of this study should be acknowledged. A common limitation of all *in vitro* infection models is the possibility of excess virions in the supernatant or bound to the cell surface, especially after spinoculation. If present, the genomic RNA in these virions could confound interpretation of the HIV transcription profile, causing the appearance of higher initiation (more TAR RNA per provirus) and less block to elongation or completion. To reduce the number of bound virions, cells were thoroughly washed at least three times after spinoculation and again before harvesting the cells. In addition, both the dual reporter and the wild type models include a sorting step preceded by additional washes, which would be expected to further decrease virions bound to the cells. However, surface bound virions may be more likely in the resting cell model. The observation that pronase treatment reduced the levels of each transcript per provirus in this model suggests that either the pronase removed some surface bound virions or it reduced HIV transcriptional initiation. At the same time, the progression through subsequent stages of HIV transcription (as assessed by ratios of one HIV RNA to another) did not appear to differ between the pronase treated and untreated cells. More importantly, the major findings were based on comparing the differences in levels of each HIV transcript between cells from the same infection that were subsequently sorted based on reporter proteins (dual reporter model) or aliquoted for activation or no activation. While we cannot exclude that any of these cells had bound virions, we anticipate that the number of bound virions would be similar between the aliquots that were subsequently activated or unstimulated, or those that were subsequently sorted based on expression of marker proteins. Finally, multiply-spliced HIV RNA is rarely packaged in virions, so bound virions should not contribute to differences in multiply-spliced HIV RNA between latent/unstimulated and productive/stimulated cells, which was the basis for the finding of the block to HIV splicing.

A second limitation of all primary cell models is that cells from different donors could behave differently. Each primary cell model was enacted in the laboratory that published the model using their usual source of donors and procedures for cell isolation, infection, and culture. Differences between donors or sources of donors could impact the findings for each model or the comparisons between models. However, when different donors were analyzed using the same model and methods, we generally saw remarkable concordance in the levels of HIV transcripts and ratios between all 2–3 donors. Within the same model, if we made relatively small changes in the methods (differing ARVs in the wild type model, or cell types in the resting model), we again saw consistency between donors but started to see differences between iterations of the same model performed with different methods. These findings suggest that the differences between models or iterations are driven more by differences in the methods rather than the donors. Finally, it should be pointed out that a total of 13 different donors were studied across all cell types and models/iterations, and despite any variability between donors or donor pools, the block to HIV splicing was observed across models and in 12 of the 13 total donors.

Additional caveats should be acknowledged. While the resting cell model and wild type model utilize infectious viruses, it is possible that proviral mutations occur during production of the viral stock or after infection of the donor cells [40]. This process may be more likely in the wild type model, which allows cell-to-cell spread of infection and a longer duration of culture after infection, and it may be one reason why the blocks to HIV expression in this model more closely resemble those in cells from ART-suppressed individuals. All three of the primary cell models studied here also utilize spinoculation, which may be important to increase infection frequency but may make them less similar to HIV infection *in vivo*. Other protocols for HIV infection, which do not use spin-infection, should be studied. However, in the Wild-type model, most of the latent infection happens during the cell-to-cell transmission, which may also explain why this model appears more similar to CD4^+^ T cells from ART-suppressed individuals. Moreover, all these models evaluate HIV infection after a short time period (5–12 days after infection) [7–9], which may mimic short-term HIV latency establishment but may not be representative of the mechanisms that regulate HIV latency in cells from chronically ART-suppressed individuals. Hence, future studies should investigate how the HIV transcription profile differs in cells from viremic HIV-infected individuals and at different time points after ART initiation. Moreover, in the time course performed in the Dual-reporter model, we observed that the blocks to HIV transcription at day 3 and 10 may differ from the ones at day 5. Therefore, the HIV transcription profile should also be compared in cells from all these models cultured for different amounts of time.

While we studied multiple variations of three different primary cell models, other primary cell models have been described and should be studied using the techniques described here [13,41–44]. At the same time, the HIV transcription profiling approach does not inform about blocks to HIV RNA export or translation, which are other proposed mechanisms of latency, so other techniques are necessary to determine whether these post-transcriptional mechanisms contribute to latency. Likewise, an inherent limitation of RNA-seq is that the actual protein expression might also be influenced by post-transcriptional processes, RNA stability, translational regulation, and post-translational modification of proteins [45,46]. Thus, RNA expression does not infer levels of protein generated or functionality, which can limit our interpretation that the up-regulation of certain snRNPs may affect HIV RNA multiple splicing. However, this limitation does not apply to RNAs that are not translated into protein, such as the snRNAs or lncRNAs.

Despite these limitations, our results provide valuable characterization of the mechanisms that regulate HIV latency in primary cell latency models and show that all these models mimic the reversible block to multiple splicing observed in cells from ART-suppressed individuals. These data suggest that multiple splicing is a conserved mechanism of latent HIV infection in primary CD4^+^ T cells and that latency reversal will require therapies that can efficiently increase HIV splicing. We also identified 234 genes whose differential expression is shared across two models and cells from ART-suppressed individuals, including factors involved in human transcription and splicing. These genes deserve further study for their contribution to HIV latency and may represent new targets for therapies designed to reactivate or silence latently-infected cells.

## Materials and methods

### Ethics statement

Studies involving human peripheral blood mononuclear cells were conducted at the following institutions, and approved by the respective Internal Review Boards (IRB), as indicated:

University of California San Francisco (UCSF) and Gladstone Institute of Virology. HIV DNA and RNA levels from ART-suppressed individuals were measured over the course of a prior study that was approved by the IRB of UCSF (the Committee on Human Research, CHR; approval #10–01561) and the San Francisco VA. The study participants included 14 HIV-infected ART-suppressed adults (median age = 51; median CD4 count = 611 cells/mm^3^; median years of suppression = 5) who were recruited prospectively and sequentially from the San Francisco VA. All participants provided written informed consent. CD4^+^ T cell pellets were isolated using negative selection from blood and either frozen immediately or after activation for 2 days with αCD3/αCD28 and antiretrovirals. The primary cell models were implemented using blood from unidentified blood donors (leukoreduction filters purchased from the blood bank) or de-identified tonsil tissue from tonsillectomies. The UCSF CHR considered this latter research exempt from further protocol review and approval.

Buck Institute: Blood was obtained from unidentified donors via the nonprofit Vitalant Organization. The Marin General Hospital Institutional Review Board (IRB) considered these samples exempt for further protocol review and approval.

George Washington University: Blood was obtained from unidentified blood donors (leukoreduction filters purchased from the blood bank). The Institutional Review Board (IRB) considered these samples exempt for further protocol review and approval.

### Primary cell models of HIV latency

Latently-infected and productively-infected or activated CD4^+^ T cells from each model were prepared by the laboratories that developed these models according to previously-published protocols [7–9].

### Wild-type virus model, cytokine-polarized CM CD4^+^ T cells [8]

PBMC were isolated from blood from healthy donors. T_CM_ and latently infected T_CM_ were generated as previously described [6,8,10,47]. Briefly, naïve CD4 T cells were isolated from healthy donors using the EasySep Human Naïve CD4^+^ T cell isolation kit (Stemcell Technologies). After isolation, naïve cells were plated at a density of 0.5×10^6^ cells per ml of RPMI (supplemented with 10% FBS, L-glutamine and Penicillin/Streptomycin) and activated with 12.5 μl of αCD3/αCD28-coated beads (Human T-Activator CD3/CD28 for T Cell Expansion and Activation Dynabeads, DynaI/Invitrogen, Carlsbad, CA) in the presence of 10 ng/ml of TGF-β1, 2 μg/ml of anti-Human IL-12 and 1 μg/ml of anti-Human IL-4 (Peprotech, Rocky Hill, NJ). Activation was performed in 96-well round plates with 100 μl per well to ensure homogeneous activation. After activation, cells were resuspended and Dynabeads were removed using a Magnetic Particle Concentrator (Dyna MPC-L, Invitrogen). Activated cells were kept at 1×10^6^ cells per ml in complete medium with 30 IU/ml of IL-2. Media and IL-2 were replaced at day 4 and 5. To generate T_CM_, media and IL-2 were replaced at day 7, 10 and 13. Cell density was maintained at 1×10^6^. To generate latently-infected T_CM_, cells were infected at day 7 using NL4-3 or a viral isolate from a positive well from a quantitative viral outgrowth assay (QVOA). One fifth of the culture was left uninfected as a control. One fifth of the culture was infected with an MOI of 0.3 by spinoculation at 2,900 rpm (1,741g) for 2 h at 37°C in 5 mL round-bottom polystyrene tubes. After infection, cells were mixed with the other three fifths of uninfected cells at 1×10^6^ cells per ml in complete medium with 30 IU/ml of IL-2. At day 10, media and IL-2 were replaced and the cells were cultured in 96 well round plates at 1×10^6^ cells per ml with 100 μl per well to ensure cell-to-cell transmission. At day 13, cells were transferred to flasks, media and IL-2 were replaced, and 100 nM of AMD-3100 or 1 μM of Raltegravir and 0.5 μM of Nelfinavir were added to the cultures to prevent viral replication. At day 17, CD4 positive cells were enriched using Dynabeads CD4 Positive Isolation Kit (Invitrogen), the percentage of p24^+^ cells was quantified by flow cytometry, and 100,000 CD4 T cells were sorted, washed with PBS and immediately frozen. Half of the remaining CD4 T cells were stimulated with αCD3/αCD28-coated beads (1 bead per cell) for 48 h, and the percentage of p24^+^ cells was quantified by flow cytometry in stimulated and unstimulated cells; 100,000 cells of each type were sorted, washed with PBS, and immediately frozen.

### Resting-cell model, peripheral and tonsil total CD4^+^ T cells [7]

Healthy PBMC were isolated from leukoreduction system chambers (Trima Accel System, Terumo BCT, Inc.) by Ficoll-Hypaque density gradient centrifugation or from tonsils. Total CD4^+^ T cells were immediately isolated by negative selection using EasySep Human CD4^+^ T Cell Enrichment Kit (Stemcell Technologies). Isolated CD4^+^ T cells were plated at a density of 1×10^6^ cells per well in a 96-well v-bottom plate at a volume of 200 μL RPMI containing 10% FCS. Cells were spinoculated with 100 ng (p24Gag) of NL4-3-Luciferase at 1,200g for 2 h at 37°C in 96-well v-bottom plates. After spinoculation, cells were washed with RPMI and 10% FCS, resuspended at a cell density of 1×10^6^ cells/mL in RPMI and 10% FCS containing 5 mM saquinavir mesylate (Sigma-Aldrich), and incubated at 37°C for 72 h. Infected cells were then plated at 1×10^6^ cells/well in a 96-well U-bottom plate in 200 mL RPMI and 10% FCS containing 30 mM raltegravir (Santa Cruz Biotechnology), directly incubated at 37°C for 48 h, or immediately activated with αCD3/CD28-coated beads (Human T-Activator CD3/CD28 for T Cell Expansion and Activation Dynabeads, Dynal/Invitrogen) (ratio 1:1), and incubated at 37°C for 48 h. To analyze these samples, cells were washed with PBS, counted, and immediately frozen for subsequent HIV DNA and RNA quantification, or lysed in Glo Lysis Buffer (Promega). Luciferase activity was quantified using a PekinElmer VICTOR3 Luminometer after mixing 50 mL of lysate with 50 mL of substrate (Luciferase Assay System, Promega). Cell viability was >80% in all blood cells and >50% in all tonsil cells, as determined by trypan blue manual counting or automatic counting using the TC20 automated cell counter (Bio-Rad).

### Pronase treatment in the resting cell model

To assess for the presence of surface bound virions in the resting cell model, this model was repeated with and without addition of pronase (to remove surface bound virions) after spinoculation of peripheral CD4+ T cells from three additional donors. The resting cell model was performed as above except that after spinoculation, cells were treated with pronase (400 μg/ml) or no pronase, incubated for 30 min at 4°C, washed with RPMI and 10% FCS, and then resuspended and incubated as described above.

### Dual-reporter virus model, activated total CD4^+^ T cells [9]

CD4^+^ T cells were extracted from PBMC from leukoreduction system chambers (Blood Centers of the Pacific, San Francisco, CA, and Stanford Blood Center) by Ficoll-Hypaque density gradient centrifugation (GE Healthcare Life Sciences, Chicago, IL). Primary CD4^+^ T cells were purified by negative selection using the RosetteSep Human CD4^+^ T Cell Enrichment Cocktail (StemCell Technologies, Canada) and cultured in RPMI 1640 medium supplemented with 10% FBS, L-glutamine (2 mM), penicillin (50 U/ml), streptomycin (50 mg/ml), and IL-2 (20 to 100 U/ml) (37°C, 5% CO2). Purified CD4^+^ T cells were stimulated with αCD3/αCD28 activating beads (Thermofisher, Waltham, MA) at a concentration of 0.5 bead/cell in the presence of 20–100 U/ml IL-2 (PeproTech, Rocky Hill, NJ) for three days. All cells were spinoculated with HIV_GKO_ at a concentration of 300 ng of p24 per 1×10^6^ cells for 2 h at 1,200g at 37°C in 96-well v-bottom plates without activation beads. Spin-infected primary CD4^+^ T cells were maintained in complete RPMI media supplemented with IL-2 (20–100 U/ml) and FACS sorted into double negative (DN, GFP^-^mKO2^-^), latently infected (GFP^-^mKO2^+^) and productively infected (GFP^+^mKO2^+^) at 3–10 days post-infection.

### Quantification of total and integrated HIV DNA

Nucleic acids from frozen cells from each primary cell model and from ART-suppressed individuals were extracted using TRI Reagent [2]. Copies of the human TERT (telomere reverse transcriptase) gene were measured in the extracted DNA using ddPCR [2] and used to determine the total cell equivalents in the extracted nucleic acids. Total HIV DNA (R-U5-pre-Gag region) was quantified in duplicate by ddPCR, as described previously [2]. Integrated HIV DNA was quantified in duplicate using *Alu*-qPCR, as previously described [14]. Total and integrated HIV DNA were expressed as copies/million cells using the cell equivalents as measured by TERT. Levels of total and integrated HIV DNA per million cells were also divided by one million to express the average copies per cell, or estimated infection frequency.

### Quantification of HIV RNA

Cell-associated HIV transcripts (Read-through, initiated [TAR], 5’elongated [R-U5-pre-Gag], unspliced/mid-transcribed [Pol], distal-transcribed [Nef], polyadenylated [PolyA] and multiply-spliced [Tat-Rev]) were quantified in duplicate using reverse transcription droplet digital PCR, as previously described [2]. An exception was the Wild-type model with _wt_NL4.3, for which the reverse primer of the PolyA assay was changed to 5’ttttttttttttttttttttttttttgagt3’ to match a mutation detected in the provirus. Levels of each HIV RNA transcript were expressed as copies per million cells using the cell equivalents in the total nucleic acid, as measured by TERT. The degree of HIV transcriptional initiation was expressed by dividing the level of initiated (TAR) HIV transcripts by the total HIV DNA (both in copies per million cells). Given the large differences in the fraction of infected cells between models (~4% to 100%) and HIV-infected individuals (<0.1%), and the presence of superinfection in some models, levels of the other HIV RNA regions were also normalized to levels of total HIV DNA (both expressed as copies per million cells) to express the average levels of each transcript per provirus (HIV RNA/HIV DNA).

Levels of each HIV transcript per million cells and per provirus were compared between latent (unstimulated) and productive (stimulated) populations. Because an increase in HIV transcriptional initiation would be expected to increase elongated HIV transcripts (and all downstream HIV RNA regions) even in the absence of any change in elongation, differences in levels of each downstream HIV RNA were evaluated with regard to the difference in HIV transcriptional initiation. In addition, the rate of progression through different stages of HIV transcription (or blocks to HIV transcription) was expressed by calculating the ratio of each RNA region to the preceding or upstream region. For example, HIV transcriptional elongation was measured by the ratio of 5’elongated to initiated HIV transcripts, which expresses the fraction of initiated transcripts that get elongated. Likewise, ratios of HIV RNA regions were used to measure the degree of mid transcription (Pol/5’elongated), completion (polyadenylated/5’elongated), and multiple splicing (multiply-spliced/polyadenylated).

### Statistical analysis of HIV RNA levels

Statistical comparisons between the latent (unstimulated) and productive (stimulated) populations within any given model were limited due to the relatively small numbers of donors. Given the variance in measuring ratios of HIV levels, within-model differences between the latent and productive populations in HIV transcriptional initiation (initiated HIV transcripts/HIV DNA) and progression through subsequent stages of HIV transcription (as measured by the ratio of one HIV RNA to another) were deemed more significant when the ratio was ≥1.5-fold higher in the productive cells and the latent cells showed evidence of a baseline block to transcription (RNA ratio considerably <1).

For each model, levels of HIV transcriptional initiation, elongation, polyadenylation, and multiple splicing in the latent or unstimulated cells were compared to unstimulated cells from ART-suppressed HIV-infected individuals using the Mann-Whitney test. Differences in HIV transcriptional initiation, elongation, completion, and splicing between the latent (unstimulated) and productive (stimulated) populations were also compared across all donors from all models using the Wilcoxon signed rank test. These analyses were performed using GraphPad Prism 5.0.

### Total RNA-Seq analysis from the unstimulated and stimulated cells from the Resting-cell model, Wild-type model, and CD4^+^ T cells from ART-suppressed individuals

To investigate the human cellular factors that may be involved in the blocks to HIV expression, RNA-seq was performed on aliquots of RNA from unstimulated and stimulated peripheral CD4^+^ T cells from the Resting-cell model (n = 2 donors), the Wild-type model (infection using _wt_NL4.3, n = 2 donors), and 2 ART-suppressed individuals. The donors and HIV-infected individuals were chosen based on the quantity of RNA remaining after measurement of HIV RNA levels by RT-ddPCR. The dual reporter model was excluded from analysis because of fundamental differences from the other systems, particularly with regard to the lack of an extra activation step. RNA quantity and quality were measured by NanoDrop ND1000 (Thermo Fisher) and also by Agilent 2100 Bioanalyzer (Agilent) to confirm RNA Integrity Number (RIN)>7. If necessary, samples from a given donor were further purified using the RNeasy MinElute kit (Qiagen) and reanalyzed to confirm RIN>7.

We analyzed 0.5-1x10^6^ cells per condition and model, and we recovered 0.3-1ug RNA per condition and model. The number of cells and the amount of RNA recovered was similar among models. Total RNA-seq was performed by the UCSF Functional Genomics Core Facility using the TruSeq Stranded Total RNA kit (Illumina) according to the manufacturer’s instructions. Total curated and normalized RNA-Seq results were analyzed using BioJupies [48] and R packages data analysis, and gene set enrichment analysis (GSEA) and Reactome databases.

## Supporting information

S1 Table List of statistically significant differentially expressed genes between unstimulated and stimulated peripheral CD4^+^ T cells from the Resting-cell model. Genes are ranked by the average log_2_ fold change (FC).
(PDF)

S2 Table List of statistically significant differentially expressed genes between unstimulated and stimulated peripheral CD4^+^ T cells from the Wild-type model. Genes are ranked by the average log_2_ fold change (FC).
(PDF)

S3 Table List of statistically significant differentially expressed genes between unstimulated and stimulated peripheral CD4^+^ T cells from ART-suppressed individuals. Genes are ranked by the average log_2_ fold change (FC).
(PDF)

S4 Table List of statistically significant differentially expressed genes (FDR<0.05) in stimulated vs. unstimulated peripheral CD4+ T cells from the Resting-cell model, the Wild-type model (wtNL4.3) and CD4+ T cells from ART-suppressed individuals. Genes are ranked alphabetically and the average log2 fold change (FC) is shown for each model.
(PDF)

S1 Fig Progression through HIV transcription stages. This schematic representation shows relative levels of HIV transcription initiation, elongation, completion, and multiple splicing quantified in latently/unstimulated and productively/stimulated HIV infected cells from the Dual-reporter, Resting-cell, and Wild-type primary cell HIV latency models and from CD4^+^ T cells from HIV-infected ART-suppressed individuals. The scale depicts the maximal block to transcription (red) to no transcriptional block (green), and the blue arrow indicates the comparative progression through each stage of HIV transcription.
(TIF)

S2 Fig The Wild-type virus model using integrase inhibitors. (A) Diagram of the model, (B) total HIV DNA, (C) level of HIV transcripts million cells, (D) level of HIV transcripts per provirus, (E) progression through HIV transcription stages. Individual values per donor (dots), and median and range (bars) are shown. Unstimulated cells at days 10 and 12 post-infection are shown in light colors and stimulated cells at day 12 in dark color.
(TIF)

S3 Fig The Resting-cell model in tonsil-CD4^+^ T cells. (A) Level of HIV transcripts per million cells, (B) progression through HIV transcription stages. Individual values per donor (dots), and median and range (bars) are shown. Unstimulated cells are shown in light color and stimulated cells in dark color.
(TIF)

S4 Fig Effect of bound virions on the Resting-cell model. (A) Diagram of the model, (B) total HIV DNA, (C) level of HIV transcripts per million cells, (D) level of HIV transcripts per provirus, (E) progression through HIV transcription stages. Individual values per donor (dots), and median and range (bars) are shown. Non-pronase treated cells are shown in light color and pronase treated cells in dark color.
(TIF)

S5 Fig The Dual-reporter virus model time course. (A-B) Level of HIV transcripts per million cells at days 3 and 10 after infection, (C-D) Level of HIV transcripts per provirus at days 3 and 10 after infection, (E-F) progression through HIV transcription stages at days 3 and 10 after infection. Individual values per donor (dot) are shown. Double negative and latent cells are shown in light colors and productive cells in dark color.
(TIF)

S6 Fig Comparison of HIV transcriptional initiation, elongation, completion, and multiple splicing across all donors. Levels of HIV transcriptional initiation, elongation, completion, and multiple splicing are shown in the latent (unstimulated) and productive (stimulated) populations from all donors used in the three main models (Figs 2–4) plus supplementary experiments (S2–S4 Figs). Bars indicate the median; P-values were calculated using the Wilcoxon signed rank test.
(TIF)
