# Supplementary material for: Human splice factors contribute to latent HIV infection in primary cell models and blood CD4+ T cells from ART-treated individuals
Source: PLoS Pathog. 2020 Nov 30;16(11):e1009060. doi: 10.1371/journal.ppat.1009060 (PMC7728277; doi:10.1371/journal.ppat.1009060)
Supplement: S1 Table — Genes are ranked by the average log2 fold change (FC). (PDF) [file ppat.1009060.s001.pdf]

Table S1. List of statistically significant differentially expressed genes between unstimulated and stimulated peripheral CD4+ T cells from the Resting-cell model. Genes are ranked by the average log2 fold change (FC).

Gene	FC (log2)	p value	FDR
IFNG	14.06	1.07E-06	0.0165
ZBTB32	12.37	4.66E-04	0.0420
AP003472.1	12.27	1.01E-04	0.0213
CCL4L2	11.10	2.38E-04	0.0307
IL9	10.86	2.76E-04	0.0323
MELK	10.75	1.62E-05	0.0165
P4HA2	10.03	2.14E-05	0.0165
ARFGEF3	9.95	1.86E-04	0.0275
CCL3L3	9.81	6.99E-06	0.0165
MND1	9.80	3.69E-05	0.0165
LINC02416	9.77	1.52E-04	0.0254
CENPM	9.66	8.28E-05	0.0188
CCDC150	9.65	2.43E-05	0.0165
BATF3	9.65	8.52E-06	0.0165
TK1	9.63	4.58E-04	0.0420
RN7SL471P	9.63	2.31E-04	0.0303
NIPAL1	9.62	7.63E-06	0.0165
GALNT18	9.61	7.52E-06	0.0165
FAM57A	9.59	9.25E-06	0.0165
IL13	9.22	1.52E-05	0.0165
IL21	9.14	2.79E-05	0.0165
C9orf172	9.14	2.66E-05	0.0165
AC017002.3	9.04	1.19E-05	0.0165
EMP1	9.03	1.20E-05	0.0165
TREM1	8.86	1.56E-04	0.0259
CDCA2	8.78	8.07E-05	0.0188
FSD1	8.73	3.21E-04	0.0355
C2orf72	8.69	4.53E-04	0.0420
C4orf47	8.64	8.89E-05	0.0197
Z83847.1	8.63	1.49E-05	0.0165
UPK1A-AS1	8.62	3.02E-05	0.0165
HIST1H4A	8.59	3.39E-05	0.0165
PDGFA	8.59	4.53E-04	0.0420
AC078819.1	8.55	1.15E-05	0.0165
SPAG4	8.52	1.10E-04	0.0219
RAC3	8.46	5.29E-05	0.0165
SPC25	8.46	3.55E-05	0.0165
AL158201.1	8.43	3.44E-05	0.0165
RNU1-122P	8.42	2.95E-05	0.0165
BNIP3P1	8.42	3.64E-04	0.0378
DEPDC4	8.42	4.07E-05	0.0165
GJC1	8.41	2.32E-04	0.0303

IGHV1OR15-3	8.40	1.90E-04	0.0276
FAM71B	8.36	1.52E-05	0.0165
PBK	8.30	1.59E-05	0.0165
PDZD4	8.30	1.47E-05	0.0165
AOX1	8.29	2.03E-05	0.0165
CYB5R2	8.24	5.19E-04	0.0443
MAK	8.19	5.33E-04	0.0452
RASAL1	8.16	1.70E-05	0.0165
PLEKHN1	8.15	2.55E-04	0.0311
BSPRY	8.12	3.35E-04	0.0363
PALD1	8.03	1.83E-04	0.0274
GALR2	8.02	2.45E-04	0.0308
TERT	8.01	1.84E-05	0.0165
AL445524.1	7.97	3.99E-04	0.0388
HOXB9	7.95	1.61E-05	0.0165
SLC17A9	7.95	3.89E-04	0.0385
SMOX	7.92	3.27E-04	0.0359
SPTBN2	7.89	1.70E-05	0.0165
C3orf14	7.88	3.93E-05	0.0165
RAI14	7.86	2.82E-04	0.0327
AP000769.1	7.85	5.03E-04	0.0435
RNU4ATAC	7.83	2.74E-05	0.0165
NCS1	7.81	3.41E-04	0.0367
ARRDC4	7.79	1.65E-04	0.0262
AC104261.1	7.77	2.83E-05	0.0165
PLXNB1	7.76	4.73E-05	0.0165
LRTOMT	7.72	1.58E-05	0.0165
STAP2	7.72	2.56E-04	0.0312
PGAM1P2	7.71	6.08E-05	0.0167
TSKU	7.68	4.71E-04	0.0420
AC130324.3	7.67	3.10E-05	0.0165
SECTM1	7.67	6.70E-05	0.0173
RCAN2	7.62	6.28E-05	0.0169
ARHGEF37	7.60	6.49E-05	0.0170
SPR	7.59	2.03E-05	0.0165
B3GNT4	7.57	3.71E-05	0.0165
PHBP3	7.57	1.80E-04	0.0274
AC110373.1	7.56	2.00E-04	0.0280
PSRC1	7.56	1.97E-04	0.0279
C3orf67	7.54	4.62E-04	0.0420
AL049836.1	7.53	3.43E-04	0.0368
FBXO43	7.47	1.87E-05	0.0165
SYNGR3	7.47	1.12E-04	0.0219
LPIN3	7.46	7.10E-05	0.0178
AL109615.3	7.42	4.49E-05	0.0165
CFAP58	7.34	2.50E-04	0.0309
NALT1	7.32	3.24E-04	0.0358

TTC24	7.28	4.33E-05	0.0165
SCDP1	7.27	5.54E-04	0.0463
BX842568.2	7.27	1.05E-04	0.0216
GVQW2	7.25	7.19E-05	0.0178
AC005702.3	7.25	2.03E-05	0.0165
LINC01160	7.17	2.54E-04	0.0311
AL117336.2	7.17	3.54E-04	0.0373
ALDOAP2	7.15	2.68E-04	0.0318
AC114803.1	7.10	3.82E-04	0.0384
CDC20P1	7.08	2.63E-04	0.0316
LINC01686	7.07	3.20E-04	0.0355
ESR2	7.07	1.36E-04	0.0239
MAP3K20-AS1	7.04	3.83E-04	0.0384
AC112721.2	7.02	5.18E-04	0.0443
IL1A	7.01	2.45E-05	0.0165
TM4SF1	6.99	6.03E-05	0.0167
NIPAL4	6.98	2.41E-05	0.0165
CCDC153	6.97	2.93E-04	0.0333
DFNA5	6.96	1.03E-04	0.0213
SLC22A1	6.94	4.64E-04	0.0420
ARVCF	6.93	3.92E-05	0.0165
QPRT	6.90	2.84E-04	0.0329
CTAGE6	6.90	7.14E-05	0.0178
GNG4	6.87	2.58E-05	0.0165
ATP2A1	6.85	7.45E-05	0.0181
RHPN2	6.84	2.63E-04	0.0316
RNVU1-7	6.83	2.80E-04	0.0327
BX539320.1	6.83	4.77E-05	0.0165
RRM2P3	6.83	2.44E-05	0.0165
ZSCAN10	6.83	2.44E-05	0.0165
LINC00158	6.83	4.10E-04	0.0395
AL109914.1	6.82	3.84E-04	0.0384
DHRS9	6.80	1.59E-04	0.0260
CDC42EP2	6.79	1.24E-04	0.0228
LYPLA2P1	6.78	5.08E-04	0.0437
AC105074.1	6.78	2.75E-05	0.0165
KIAA1549	6.77	1.24E-04	0.0228
TWIST1	6.73	3.83E-04	0.0384
CNGA1	6.71	1.12E-04	0.0219
AC016700.3	6.66	3.28E-05	0.0165
AC007608.4	6.66	6.22E-04	0.0497
C11orf74	6.62	3.83E-04	0.0384
GLP1R	6.61	3.23E-05	0.0165
SLC25A5P5	6.60	4.46E-04	0.0420
AC091132.4	6.59	2.82E-05	0.0165
NRN1	6.57	2.58E-05	0.0165
PQLC2	6.56	2.61E-05	0.0165

RAC1P4	6.54	1.92E-04	0.0277
AC012085.1	6.52	1.47E-04	0.0248
PITX3	6.51	3.73E-04	0.0380
AC243829.4	6.50	4.32E-05	0.0165
PCDH1	6.49	3.69E-04	0.0379
AC091607.1	6.49	3.87E-05	0.0165
MIR3153	6.45	2.44E-04	0.0308
AC016735.2	6.44	2.14E-04	0.0292
IQANK1	6.40	1.10E-04	0.0219
VPS37D	6.37	1.83E-04	0.0274
AC008083.2	6.34	2.93E-05	0.0165
BX284668.2	6.33	1.98E-04	0.0280
ZFHX2	6.30	4.06E-05	0.0165
AC074351.1	6.30	2.73E-05	0.0165
ECEL1	6.29	2.23E-04	0.0300
DDX11-AS1	6.25	4.00E-04	0.0388
C2CD4D	6.24	2.78E-05	0.0165
TEAD3	6.21	3.58E-04	0.0374
RTN2	6.19	3.26E-05	0.0165
JPH1	6.16	3.02E-04	0.0341
AC017076.1	6.15	8.86E-05	0.0197
OR7E31P	6.15	1.71E-04	0.0267
GMPR	6.14	3.26E-04	0.0358
PEX11G	6.13	1.83E-04	0.0274
AL592164.1	6.12	6.97E-05	0.0177
AC135178.5	6.11	5.48E-05	0.0165
CAPSL	6.11	2.92E-05	0.0165
MCMDC2	6.11	2.92E-05	0.0165
RASL11A	6.10	3.54E-04	0.0373
CDKL2	6.09	7.09E-05	0.0178
LPP-AS2	6.08	4.55E-05	0.0165
TUBBP6	6.07	4.38E-04	0.0416
ZNF385C	6.06	3.82E-04	0.0384
MIR586	6.05	2.09E-04	0.0287
C20orf96	6.01	1.43E-04	0.0245
RIPK4	6.00	2.41E-04	0.0308
THAP12P1	5.99	7.50E-05	0.0181
PRG2	5.97	5.11E-04	0.0437
MAP6D1	5.94	7.75E-05	0.0185
TMEM145	5.94	3.17E-05	0.0165
TNPO1P2	5.91	1.88E-04	0.0276
FAM109B	5.91	3.21E-05	0.0165
MET	5.88	1.45E-04	0.0247
AC021224.1	5.88	2.59E-04	0.0313
NRM	5.87	4.00E-04	0.0388
C1orf194	5.87	4.18E-04	0.0400
OXTR	5.86	3.31E-05	0.0165

CHST3	5.85	4.97E-05	0.0165
MIR3161	5.85	4.97E-05	0.0165
NPPC	5.83	1.71E-04	0.0267
PTPRU	5.80	5.42E-05	0.0165
UQCRHP2	5.78	1.95E-04	0.0279
LINC01399	5.76	4.74E-04	0.0421
AC087072.1	5.75	6.31E-05	0.0169
IL22	5.74	2.23E-04	0.0300
DISP3	5.73	6.01E-05	0.0167
RNU6-789P	5.72	4.72E-04	0.0420
CRABP2	5.71	2.27E-04	0.0303
TMEM256	5.70	3.99E-04	0.0388
AC069120.2	5.70	2.16E-04	0.0293
AC136604.2	5.69	1.81E-04	0.0274
MIR604	5.68	2.47E-04	0.0309
SLC2A8	5.68	3.25E-04	0.0358
NAMPT	5.67	2.04E-06	0.0165
GNG8	5.65	3.46E-05	0.0165
AC092017.1	5.62	4.79E-04	0.0422
AC018371.2	5.60	1.59E-04	0.0260
ZEB2-AS1	5.57	5.55E-04	0.0463
AL391832.3	5.56	2.49E-04	0.0309
AC009118.2	5.56	3.78E-05	0.0165
CEL	5.53	1.88E-04	0.0276
TNFRSF4	5.49	5.72E-04	0.0471
BCL2L15	5.47	1.00E-04	0.0213
Z73417.1	5.45	1.09E-04	0.0219
GGT2	5.45	3.79E-05	0.0165
CBSL	5.42	3.85E-05	0.0165
PYGO1	5.42	3.85E-05	0.0165
ZMYND12	5.42	3.85E-05	0.0165
FMO5	5.41	3.57E-04	0.0374
SFN	5.37	4.04E-05	0.0165
AC107954.1	5.35	2.02E-04	0.0281
AC018761.2	5.33	5.63E-05	0.0165
HSPE1P4	5.33	5.63E-05	0.0165
MAP1LC3A	5.33	5.63E-05	0.0165
ACTN3	5.30	4.88E-05	0.0165
HSF2BP	5.30	4.21E-05	0.0165
KRT18P6	5.25	5.38E-04	0.0454
RASD1	5.24	1.69E-04	0.0266
ETNPPL	5.11	1.43E-04	0.0245
NPM1P38	5.09	3.70E-04	0.0379
AL157871.4	5.08	4.19E-05	0.0165
NPM1P31	5.08	8.19E-05	0.0188
PHBP9	5.07	5.80E-04	0.0476
PCDHGB5	4.98	2.68E-04	0.0318

RPA2P2	4.93	6.37E-05	0.0169
GAPDH	4.90	1.41E-04	0.0245
DUSP5	4.87	6.06E-05	0.0167
SOCS3	4.74	4.68E-05	0.0165
CDC6	4.69	4.86E-04	0.0427
ENO1	4.69	2.50E-04	0.0309
TPX2	4.57	8.33E-06	0.0165
AC233266.2	4.57	1.32E-04	0.0234
USP27X-AS1	4.49	3.38E-04	0.0364
AL049842.1	4.37	8.05E-05	0.0188
PDCD1	4.19	2.29E-04	0.0303
TTK	4.15	4.31E-04	0.0410
CORO1B	4.14	5.54E-05	0.0165
SDC4	3.90	9.80E-05	0.0211
TUBA1B	3.89	2.52E-04	0.0309
SNORD3B-2	3.70	2.41E-04	0.0308
BRCA1	3.70	2.27E-04	0.0303
BATF	3.70	1.02E-04	0.0213
SNRNP25	3.69	4.75E-04	0.0421
DCLRE1B	3.67	1.23E-04	0.0228
PRELID2	3.66	2.51E-04	0.0309
SYTL3	3.65	3.95E-04	0.0387
KIF15	3.56	3.86E-04	0.0384
RNU1-27P	3.53	2.00E-04	0.0280
TBX21	3.51	3.09E-04	0.0347
TUBAP2	3.50	4.73E-04	0.0420
PGK1	3.41	1.91E-04	0.0276
RAD51AP1	3.35	4.63E-04	0.0420
KIF11	3.32	4.59E-04	0.0420
DHFR	3.20	5.30E-04	0.0450
GNPDA1	3.18	1.99E-04	0.0280
HIST1H4F	3.14	2.34E-04	0.0304
SRGN	3.12	1.63E-04	0.0260
TPM4	3.11	1.22E-04	0.0228
FANCI	3.10	1.30E-05	0.0165
AC015720.2	3.10	2.91E-04	0.0333
STARD4	3.06	1.11E-04	0.0219
SHMT2	3.05	2.43E-04	0.0308
CENPN	3.04	6.27E-04	0.0499
XRCC3	2.99	4.79E-04	0.0422
CHAF1B	2.98	5.48E-04	0.0459
GDPGP1	2.95	4.51E-04	0.0420
TONSL	2.89	2.93E-04	0.0333
EFEMP2	2.87	1.21E-04	0.0228
IL23R	2.75	3.48E-04	0.0370
CLIC1	2.74	1.22E-05	0.0165
TFDP1	2.72	5.55E-05	0.0165

WDR76	2.65	4.10E-04	0.0395
MTHFD2	2.65	3.07E-04	0.0345
CD200	2.60	3.52E-04	0.0372
ARID5A	2.60	7.61E-06	0.0165
UBALD2	2.46	3.50E-04	0.0371
RANBP1	2.44	2.14E-04	0.0292
AL050331.3	2.43	3.37E-04	0.0363
FAM206BP	2.43	3.37E-04	0.0363
MFSD2B	2.43	3.37E-04	0.0363
TRIM60P14	2.43	3.37E-04	0.0363
MDH2	2.37	6.33E-06	0.0165
LIMA1	2.36	3.96E-05	0.0165
PIM3	2.33	6.21E-05	0.0169
CALM3	2.26	1.92E-04	0.0277
FLOT1	2.25	8.49E-05	0.0190
ACTG1	2.25	4.54E-04	0.0420
IL24	2.24	3.48E-04	0.0370
ARSD	2.18	5.59E-04	0.0463
MFSD10	2.17	1.62E-04	0.0260
NCAPD2	1.98	5.61E-05	0.0165
RAB27A	1.97	2.41E-04	0.0308
MRPL47	1.96	5.05E-04	0.0436
RBPJ	1.92	4.09E-04	0.0395
LMAN1	1.87	1.75E-04	0.0269
YEATS2	1.86	4.57E-04	0.0420
ZNF367	1.85	4.99E-04	0.0434
NOB1	1.78	6.16E-04	0.0495
AP3M2	1.74	2.88E-04	0.0331
EIF4H	1.73	2.89E-05	0.0165
SGO2	1.66	5.62E-04	0.0465
ACTN4	1.66	2.36E-04	0.0305
ARF6	1.66	2.87E-04	0.0331
TRMT6	1.54	5.46E-04	0.0458
DOT1L	1.54	4.40E-04	0.0417
CD99	1.52	1.25E-04	0.0228
TMEM63B	1.51	5.54E-04	0.0463
SNHG16	1.50	6.35E-05	0.0169
GNG5	1.46	6.04E-05	0.0167
TXLNA	1.46	5.90E-04	0.0482
JOSD1	1.45	4.18E-04	0.0400
RPSAP15	1.45	7.60E-05	0.0182
DSN1	1.45	1.96E-04	0.0279
NUP155	1.42	5.52E-05	0.0165
LCP1	1.39	5.66E-05	0.0165
PSMD14	1.39	1.32E-04	0.0234
PDAP1	1.38	4.29E-04	0.0408
PTPN11	1.38	2.56E-04	0.0312

KLHL6	1.35	6.04E-04	0.0488
BRI3BP	1.34	6.25E-04	0.0499
NLE1	1.34	2.09E-04	0.0287
STAT6	1.33	1.01E-04	0.0213
ANKLE2	1.31	1.55E-04	0.0258
PDE4D	1.27	1.98E-04	0.0280
NGLY1	1.26	1.75E-05	0.0165
GPN1	1.25	2.09E-04	0.0287
RANGAP1	1.25	1.75E-04	0.0269
CBR1	1.24	9.95E-05	0.0213
ODF2	1.23	2.51E-04	0.0309
SNRPD2	1.22	2.33E-04	0.0303
PAXIP1	1.15	2.57E-04	0.0312
THAP4	1.15	7.79E-05	0.0185
MAGOH	1.06	1.60E-04	0.0260
SLC25A39	1.06	5.26E-04	0.0448
LSM12	1.05	2.77E-04	0.0323
SEC13	1.05	1.20E-04	0.0228
GARS	1.01	2.69E-04	0.0318
RMDN1	0.94	5.99E-05	0.0167
TRIR	0.86	5.56E-04	0.0463
RBM42	0.66	6.17E-04	0.0495
MORF4L1P1	0.62	5.45E-04	0.0458
PCNT	0.58	2.88E-04	0.0331
GALM	0.57	1.81E-04	0.0274
RAB1A	0.56	1.57E-04	0.0259
GSPT1	0.45	3.72E-04	0.0380
RAP1B	-0.55	2.39E-04	0.0308
MRPS10	-0.58	5.10E-04	0.0437
LEMD3	-0.62	3.49E-04	0.0370
RSF1	-0.65	1.23E-04	0.0228
FNIP1	-0.67	5.91E-04	0.0482
DNAJB14	-0.70	5.43E-04	0.0457
GPATCH2L	-0.73	4.68E-04	0.0420
HIPK3	-0.73	2.29E-04	0.0303
PAPOLA	-0.75	2.49E-04	0.0309
ZNF814	-0.77	1.26E-04	0.0228
MORC2	-0.81	4.80E-04	0.0422
ELK4	-0.89	1.87E-05	0.0165
FYCO1	-0.91	4.70E-04	0.0420
PPWD1	-0.92	4.52E-04	0.0420
ZEB1	-0.94	4.63E-05	0.0165
PHF3	-0.94	2.97E-04	0.0336
MSL2	-0.96	3.91E-04	0.0385
STYX	-0.98	1.73E-04	0.0269
CCDC88C	-0.98	7.31E-05	0.0179
SAFB2	-1.00	1.91E-04	0.0276

RNF115	-1.02	2.70E-04	0.0319
MIA3	-1.06	5.21E-05	0.0165
ANKMY2	-1.06	3.32E-04	0.0362
RCSD1	-1.09	4.92E-04	0.0428
ZRANB1	-1.11	3.68E-04	0.0379
FBXW7	-1.11	3.94E-05	0.0165
PIKFYVE	-1.11	5.57E-04	0.0463
MFN1	-1.12	3.97E-04	0.0388
IFT172	-1.12	1.76E-04	0.0269
DDX17	-1.13	1.96E-04	0.0279
TRMT2B	-1.14	1.71E-04	0.0267
ERAP2	-1.15	1.11E-04	0.0219
CCM2	-1.16	4.91E-04	0.0428
RPS6KA3	-1.21	3.13E-04	0.0350
MBTPS1	-1.23	4.76E-04	0.0421
RBM12B	-1.26	2.45E-04	0.0308
ZNF333	-1.30	2.49E-04	0.0309
BIN2	-1.31	4.61E-04	0.0420
FMR1	-1.31	4.53E-04	0.0420
CCNT2	-1.32	6.17E-04	0.0495
CNTRL	-1.33	4.88E-05	0.0165
SOAT1	-1.33	7.53E-05	0.0181
STXBP5	-1.35	2.32E-04	0.0303
STAG3L5P	-1.36	3.76E-04	0.0383
PURA	-1.38	4.55E-05	0.0165
CENPC	-1.39	8.01E-06	0.0165
SLC15A2	-1.40	5.86E-04	0.0480
ZNF793	-1.40	2.20E-04	0.0298
ZNF792	-1.41	3.80E-05	0.0165
ZNF227	-1.43	1.12E-04	0.0219
ASAH1	-1.43	5.41E-04	0.0456
GIMAP4	-1.46	1.23E-04	0.0228
TMEM116	-1.47	7.24E-05	0.0179
IKBKE	-1.53	4.53E-04	0.0420
TRAJ23	-1.57	4.02E-05	0.0165
WDR73	-1.58	8.11E-05	0.0188
PSMA3-AS1	-1.60	1.77E-04	0.0270
AL513548.3	-1.62	4.58E-04	0.0420
CCNL2	-1.63	1.82E-04	0.0274
SSBP2	-1.64	6.14E-04	0.0494
THRA	-1.64	3.15E-05	0.0165
MED21	-1.70	3.90E-05	0.0165
MAN1C1	-1.71	4.76E-04	0.0421
MTHFD2L	-1.72	3.59E-04	0.0374
BRWD1	-1.75	9.23E-05	0.0202
RNU4ATAC11P	-1.77	5.58E-04	0.0463
ZNF548	-1.78	4.91E-04	0.0428

AL356801.1	-1.80	1.18E-04	0.0225
ITGA10	-1.80	2.27E-04	0.0303
REV1	-1.80	5.10E-04	0.0437
RNF216	-1.85	4.67E-04	0.0420
TULP4	-1.85	1.52E-04	0.0254
ZNF235	-1.86	8.25E-05	0.0188
AC108010.1	-1.88	1.30E-04	0.0232
TGFBR3	-1.92	1.69E-04	0.0266
SNHG14	-1.92	1.14E-04	0.0221
LINC01138	-1.93	4.71E-04	0.0420
AC135983.2	-1.98	3.05E-04	0.0344
MGAT4A	-2.00	3.34E-05	0.0165
MAP3K1	-2.03	2.39E-05	0.0165
L3MBTL3	-2.11	3.87E-04	0.0385
ZNF75D	-2.21	4.72E-04	0.0420
L3HYPDH	-2.21	1.63E-04	0.0260
GLIPR1	-2.22	1.02E-04	0.0213
UTRN	-2.23	2.00E-06	0.0165
SMCR8	-2.24	1.75E-05	0.0165
MCEE	-2.26	4.66E-04	0.0420
IRS2	-2.27	3.79E-04	0.0384
PARP8	-2.33	4.72E-04	0.0420
LEF1-AS1	-2.38	3.84E-04	0.0384
TRBV12-2	-2.38	3.85E-04	0.0384
ZNF44	-2.39	1.01E-04	0.0213
ITM2B	-2.54	2.66E-04	0.0318
ZNF767P	-2.59	6.26E-04	0.0499
SORL1	-2.60	7.57E-05	0.0182
TC2N	-2.66	1.16E-05	0.0165
RASSF3	-2.71	6.01E-04	0.0487
AL392172.1	-2.74	1.74E-04	0.0269
AMY2B	-2.77	4.72E-04	0.0420
ZNF879	-2.80	1.37E-04	0.0239
STK38	-2.80	1.03E-04	0.0213
IPCEF1	-2.81	2.03E-04	0.0282
AP001767.4	-2.82	1.58E-04	0.0260
SCML4	-2.84	3.11E-04	0.0349
PLCL1	-2.89	1.11E-04	0.0219
AC232271.1	-2.90	1.42E-04	0.0245
FYB1	-2.99	4.60E-04	0.0420
SAMD9	-3.02	6.34E-05	0.0169
KLF3	-3.03	1.17E-04	0.0225
KIAA1551	-3.04	1.77E-04	0.0270
GIMAP1	-3.08	1.82E-04	0.0274
GIMAP7	-3.12	1.39E-04	0.0243
GBP3	-3.13	3.20E-04	0.0355
SLC16A10	-3.17	4.21E-04	0.0401

TRANK1	-3.17	2.61E-04	0.0315
PRR13	-3.19	5.60E-05	0.0165
ZNF181	-3.30	2.06E-04	0.0285
EVI2B	-3.52	5.62E-05	0.0165
AL353194.1	-3.57	2.37E-04	0.0307
ZNF559	-3.60	4.53E-05	0.0165
ATM	-3.72	2.33E-04	0.0303
AL133375.1	-3.87	6.87E-05	0.0176
LINC02078	-4.04	5.02E-04	0.0434
AC005899.5	-4.36	5.54E-05	0.0165
ZNRF2P2	-4.63	4.99E-04	0.0434
SOWAHD	-4.64	1.18E-04	0.0225
AP000911.3	-4.73	4.72E-05	0.0165
Z95114.3	-4.82	1.19E-04	0.0228
EDAR	-4.83	2.75E-05	0.0165
AC079921.1	-4.83	1.27E-04	0.0229
NPM1P33	-4.90	1.24E-04	0.0228
KLHDC1	-4.97	6.11E-04	0.0492
COL9A3	-4.99	6.18E-04	0.0495
AC253576.2	-5.08	3.69E-04	0.0379
RN7SL840P	-5.10	2.07E-04	0.0286
AC005899.1	-5.22	2.17E-04	0.0294
RN7SL508P	-5.33	1.44E-04	0.0245
BANK1	-5.34	5.91E-04	0.0482
AL928654.4	-5.36	3.44E-04	0.0368
RN7SL242P	-5.36	1.99E-04	0.0280
AL159163.1	-5.42	5.32E-05	0.0165
AL391497.1	-5.56	5.56E-05	0.0165
OSGIN1	-5.58	5.76E-05	0.0166
MIR4459	-5.59	3.34E-04	0.0363
AC073389.3	-5.61	5.04E-05	0.0165
AC008967.1	-5.62	3.47E-05	0.0165
AC007292.1	-5.63	5.97E-05	0.0167
AK4P4	-5.65	3.43E-05	0.0165
PWAR1	-5.66	3.69E-04	0.0379
SLC7A7	-5.66	3.69E-04	0.0379
RNY1P16	-5.66	4.51E-05	0.0165
CACNA1E	-5.68	3.91E-05	0.0165
OR13C5	-5.74	3.40E-05	0.0165
AC108002.1	-5.75	6.10E-04	0.0492
ESRRAP1	-5.75	5.09E-04	0.0437
AP000255.1	-5.78	4.72E-04	0.0420
SLC45A2	-5.78	4.72E-04	0.0420
DNM1	-5.80	3.19E-04	0.0355
AC090517.2	-5.80	1.06E-04	0.0216
LEXM	-5.85	3.26E-04	0.0358
IL12A	-5.87	3.60E-04	0.0375

AL357793.1	-5.88	6.55E-05	0.0170
MTND2P13	-5.89	8.03E-05	0.0188
AL121983.2	-5.97	1.40E-04	0.0243
AC012358.3	-6.01	1.59E-04	0.0260
CDKN2B-AS1	-6.03	6.77E-05	0.0174
EIF4EBP2P2	-6.07	2.66E-04	0.0318
AC018511.2	-6.07	3.10E-05	0.0165
SRCIN1	-6.07	1.08E-04	0.0219
SNORD38C	-6.08	4.00E-05	0.0165
BEND7P1	-6.13	4.20E-05	0.0165
AC007881.3	-6.14	1.14E-04	0.0222
ADAMTS7P1	-6.16	1.66E-04	0.0263
LIN7A	-6.17	1.63E-04	0.0260
RN7SL569P	-6.18	4.44E-05	0.0165
AC022395.1	-6.19	6.41E-05	0.0169
ARHGAP26-IT1	-6.19	6.41E-05	0.0169
TRHDE-AS1	-6.19	5.17E-05	0.0165
AGGF1P2	-6.20	5.01E-04	0.0434
P2RX6	-6.25	9.19E-05	0.0202
DM1-AS	-6.27	4.72E-04	0.0420
AL354793.1	-6.27	2.78E-05	0.0165
AC011446.1	-6.28	4.15E-04	0.0398
RHCE	-6.29	2.44E-04	0.0308
RN7SL688P	-6.29	2.44E-04	0.0308
CLU	-6.30	2.77E-04	0.0323
SLC16A13	-6.37	1.52E-04	0.0254
IGLV3-12	-6.38	3.81E-04	0.0384
HPGDS	-6.41	2.91E-04	0.0333
RASGRF2-AS1	-6.44	3.58E-04	0.0374
SV2B	-6.44	2.86E-04	0.0330
BX537318.2	-6.47	5.80E-04	0.0476
WNT2B	-6.48	3.96E-05	0.0165
CABYR	-6.48	2.48E-04	0.0309
TTC39C-AS1	-6.50	4.21E-04	0.0401
AL139022.1	-6.53	8.22E-05	0.0188
AL591721.1	-6.53	8.22E-05	0.0188
EEPD1	-6.54	4.88E-04	0.0427
AL117344.1	-6.54	4.05E-04	0.0391
UBR5-AS1	-6.55	2.76E-05	0.0165
AL138762.1	-6.56	4.27E-05	0.0165
AC246785.2	-6.57	3.59E-05	0.0165
AL139124.1	-6.61	8.35E-05	0.0188
RNASEH2B-AS1	-6.61	2.82E-04	0.0327
RNU6-761P	-6.61	1.09E-04	0.0219
TFEC	-6.61	1.09E-04	0.0219
BRWD1-AS2	-6.63	3.55E-05	0.0165
AC092747.2	-6.67	7.36E-05	0.0180

AL358933.1	-6.67	2.96E-04	0.0336
AP001372.4	-6.69	1.25E-04	0.0228
LINC00205	-6.72	6.04E-04	0.0488
AC007773.1	-6.72	3.04E-05	0.0165
FAM204BP	-6.73	3.94E-04	0.0387
AC010754.1	-6.74	4.70E-04	0.0420
AC007637.1	-6.74	5.72E-05	0.0166
CDC42-IT1	-6.77	1.86E-04	0.0275
CPEB2-AS1	-6.77	4.06E-05	0.0165
AL356020.1	-6.79	1.28E-04	0.0231
BTG1P1	-6.80	5.17E-05	0.0165
LRRK2	-6.80	4.24E-05	0.0165
AC243654.2	-6.80	5.91E-04	0.0482
MMP2	-6.81	3.34E-05	0.0165
AC110792.1	-6.81	2.24E-04	0.0300
AC022098.2	-6.82	2.94E-04	0.0334
AL137779.2	-6.84	2.66E-05	0.0165
ARHGEF40	-6.84	1.62E-04	0.0260
CTD-3080P12.3	-6.86	2.41E-04	0.0308
DNM1P46	-6.87	2.12E-05	0.0165
RNU6-808P	-6.88	9.42E-05	0.0205
MIR4644	-6.88	3.59E-04	0.0374
C1GALT1P1	-6.89	8.44E-05	0.0190
AC104447.1	-6.89	1.61E-04	0.0260
LINC01504	-6.89	5.34E-04	0.0452
AC002091.1	-6.91	5.97E-04	0.0485
MPEG1	-6.93	1.96E-04	0.0279
AC097527.1	-6.93	1.90E-04	0.0276
AC133555.1	-6.93	3.86E-04	0.0384
NPIPP1	-6.95	4.92E-05	0.0165
AC012291.1	-6.96	4.26E-05	0.0165
AL109917.1	-6.97	2.08E-05	0.0165
AC008555.1	-6.98	3.74E-04	0.0381
RNU6-606P	-7.00	4.47E-05	0.0165
AC079360.1	-7.01	9.44E-05	0.0205
AC110053.1	-7.03	3.30E-05	0.0165
AC010245.2	-7.04	9.47E-05	0.0205
AC016168.2	-7.05	5.02E-05	0.0165
GPR156	-7.06	1.25E-04	0.0228
AC104966.1	-7.08	2.16E-05	0.0165
NPM1P49	-7.09	2.04E-05	0.0165
AC138409.1	-7.11	3.29E-04	0.0360
MIR99AHG	-7.11	5.64E-04	0.0466
AC004908.3	-7.11	9.11E-05	0.0201
AP003356.1	-7.12	1.03E-04	0.0213
IGSF6	-7.14	5.80E-05	0.0166
WDR86-AS1	-7.14	4.87E-04	0.0427

AL136368.1	-7.16	1.30E-04	0.0232
AL606491.1	-7.16	1.30E-04	0.0232
IGLV2-14	-7.17	5.81E-05	0.0166
AC011816.1	-7.18	4.66E-04	0.0420
AL358472.1	-7.19	3.91E-04	0.0385
C11orf94	-7.21	1.74E-04	0.0269
AC005480.1	-7.22	1.86E-04	0.0275
AC116096.1	-7.23	4.83E-05	0.0165
CCSER1	-7.24	4.65E-04	0.0420
RPL23AP72	-7.24	4.65E-04	0.0420
AL031274.1	-7.25	2.72E-04	0.0320
PRKG1	-7.26	2.65E-05	0.0165
LILRB4	-7.26	5.40E-05	0.0165
RPSAP16	-7.27	3.43E-04	0.0368
ZNF582-AS1	-7.30	4.21E-05	0.0165
ZGLP1	-7.30	9.90E-05	0.0213
BCO2	-7.30	1.86E-05	0.0165
AC025171.4	-7.32	2.74E-04	0.0322
MIR505	-7.34	2.69E-04	0.0318
AC127070.3	-7.34	2.12E-04	0.0290
AP001157.1	-7.34	1.63E-04	0.0260
POU5F1P5	-7.36	3.88E-04	0.0385
IGHA1	-7.36	1.77E-05	0.0165
AC005306.1	-7.39	2.20E-05	0.0165
ACE	-7.39	2.64E-05	0.0165
PFN2	-7.40	2.29E-04	0.0303
AL162274.3	-7.43	2.63E-04	0.0316
SLC23A3	-7.45	6.01E-04	0.0487
AL390066.1	-7.45	3.92E-04	0.0386
KCNA2	-7.46	1.86E-04	0.0275
RN7SL753P	-7.47	2.36E-05	0.0165
CFL1P1	-7.48	3.86E-05	0.0165
AC007780.1	-7.55	3.68E-04	0.0379
CHMP1B2P	-7.55	6.54E-05	0.0170
AP003035.1	-7.61	5.34E-04	0.0452
AC010627.1	-7.61	1.05E-04	0.0216
SYNPO2	-7.63	4.03E-04	0.0391
AC018845.3	-7.63	4.39E-05	0.0165
CR2	-7.64	1.32E-04	0.0234
PSMD10P1	-7.64	1.24E-04	0.0228
TMEM191A	-7.65	5.85E-05	0.0167
STAB1	-7.69	5.14E-04	0.0440
AC018868.2	-7.70	6.27E-05	0.0169
GPR146	-7.71	3.52E-05	0.0165
AC020659.1	-7.75	2.46E-04	0.0308
AC022079.1	-7.80	1.16E-04	0.0224
AC234782.4	-7.84	4.54E-04	0.0420

AC018926.3	-7.94	3.46E-04	0.0369
POT1-AS1	-7.97	1.36E-04	0.0239
SLC14A1	-8.00	5.51E-05	0.0165
CPA3	-8.00	4.61E-04	0.0420
TSC22D1-AS1	-8.01	1.03E-04	0.0213
FAM234A	-8.02	2.19E-05	0.0165
KLRB1	-8.03	4.03E-05	0.0165
LINC01719	-8.04	1.44E-04	0.0245
AC027682.4	-8.10	1.26E-04	0.0229
LIPT2	-8.13	1.34E-05	0.0165
CASP1P2	-8.21	1.46E-05	0.0165
PLXDC2	-8.21	1.88E-04	0.0276
LINC01624	-8.22	2.19E-05	0.0165
AP002365.1	-8.35	1.95E-04	0.0279
AC023090.2	-8.50	1.91E-05	0.0165
AC241520.1	-8.51	6.94E-05	0.0177
AC008014.1	-8.57	3.25E-05	0.0165
FGF9	-8.60	4.81E-05	0.0165
SLC22A23	-8.60	7.20E-05	0.0178
AC099494.3	-8.63	2.03E-04	0.0282
AC011726.3	-8.65	4.71E-05	0.0165
AC046136.1	-8.67	4.00E-04	0.0388
WNT7A	-8.87	2.18E-05	0.0165
PCDH9	-8.90	4.19E-05	0.0165
C12orf42	-8.92	3.15E-04	0.0351
PPP2R2B	-8.95	1.52E-04	0.0254
ACTN1-AS1	-8.97	3.75E-05	0.0165
KRT1	-8.98	2.32E-04	0.0303
LINC02362	-9.12	1.55E-05	0.0165
LINC02328	-9.12	1.53E-05	0.0165
AL670729.1	-9.13	1.66E-04	0.0263
FAM19A1	-9.25	8.30E-05	0.0188
MYO16	-9.32	5.65E-04	0.0466
KLF3-AS1	-9.34	1.47E-04	0.0248
LINC00824	-9.50	6.85E-06	0.0165
GPA33	-9.61	1.74E-05	0.0165
OCM	-9.64	2.60E-04	0.0315
DSC1	-9.70	5.58E-04	0.0463
B3GALT2	-9.73	4.83E-05	0.0165
CR1	-9.75	7.93E-05	0.0187
HCG27	-9.97	2.01E-05	0.0165
