# Supplementary material for: Human splice factors contribute to latent HIV infection in primary cell models and blood CD4+ T cells from ART-treated individuals
Source: PLoS Pathog. 2020 Nov 30;16(11):e1009060. doi: 10.1371/journal.ppat.1009060 (PMC7728277; doi:10.1371/journal.ppat.1009060)
Supplement: S2 Table — Genes are ranked by the average log2 fold change (FC). (PDF) [file ppat.1009060.s002.pdf]

Table S2. List of statistically significant differentially expressed genes between unstimulated and stimulated peripheral CD4+ T cells from the Wild-type model. Genes are ranked by the average log2 fold change (FC).

Gene	FC (log2)	p value	FDR
IL2	13.79	8.56E-07	0.0014
ZBED2	11.11	5.20E-05	0.0027
MYOF	11.04	1.28E-05	0.0018
AP003472.1	10.48	2.68E-06	0.0014
G0S2	10.22	9.28E-07	0.0014
FABP5P7	9.56	1.23E-05	0.0018
ENST000003232744	9.43	2.84E-04	0.0045
NR4A3	9.29	1.15E-04	0.0033
HMGB1P17	9.24	5.19E-06	0.0016
IRF8	9.02	8.93E-06	0.0016
ENST000002568760	9.02	1.47E-02	0.0370
ENST00000318981	8.93	5.76E-06	0.0016
ADM	8.91	3.60E-06	0.0015
ENST000003150501	8.66	5.57E-06	0.0016
GZMB	8.61	1.05E-03	0.0079
IL1A	8.56	4.06E-05	0.0024
IGHM	8.54	1.51E-04	0.0036
CDC45	8.53	1.09E-06	0.0014
ENST000002636302	8.45	5.83E-05	0.0028
ENST000003127830	8.44	1.25E-05	0.0018
ENST000002288433	8.41	6.36E-06	0.0016
EGR2	8.38	3.36E-05	0.0023
IL24	8.33	4.88E-05	0.0026
LINC00158	8.32	2.63E-05	0.0021
TUBB6	8.31	1.24E-04	0.0035
LINC02341	8.31	1.45E-06	0.0014
GALNT18	8.25	1.97E-05	0.0020
IGSF3	8.18	2.01E-04	0.0040
MYO1B	8.15	8.61E-04	0.0072
DHFRP1	8.12	8.74E-06	0.0016
LINC02051	8.11	3.37E-05	0.0023
CTTN	8.11	8.07E-06	0.0016
ENST000003082782	8.10	3.68E-05	0.0023
ENST000003083610	8.07	5.31E-05	0.0027
ENST000003109550	8.07	2.74E-06	0.0014
CCL3	8.00	8.39E-04	0.0071
CCL4	7.93	2.11E-03	0.0113
ENST000003018432	7.90	2.28E-06	0.0014
MT3	7.87	1.79E-05	0.0020
ENST000003237600	7.86	5.34E-04	0.0058
ENST000003141631	7.85	1.50E-05	0.0019
ENST000002587810	7.85	1.06E-03	0.0079
ENST000002743760	7.79	1.74E-05	0.0020
ENST000002590431	7.78	4.42E-03	0.0172
SPR	7.78	1.57E-05	0.0019

ENST000003566570	7.76	9.91E-05	0.0032
UBE2C	7.72	2.11E-04	0.0041
DGKI	7.71	1.50E-05	0.0019
IL21-AS1	7.71	1.91E-03	0.0107
SLC27A2	7.67	2.49E-04	0.0043
DDIT4L	7.66	3.82E-05	0.0023
EXO1	7.64	7.60E-07	0.0014
DOK5	7.58	5.35E-05	0.0027
SETBP1	7.49	2.38E-04	0.0042
PBK	7.48	1.38E-06	0.0014
ENST000003331371	7.48	2.27E-05	0.0020
GPR87	7.48	2.47E-03	0.0124
ENST000003157112	7.45	5.03E-03	0.0185
RRM2	7.40	2.62E-06	0.0014
ENST000002681381	7.37	4.97E-03	0.0184
TNFRSF18	7.37	3.49E-03	0.0149
LPL	7.35	2.70E-04	0.0044
CCL20	7.33	3.19E-05	0.0023
APOBEC3B	7.33	2.50E-05	0.0021
ZBTB32	7.32	3.01E-05	0.0022
HILPDA	7.31	4.92E-07	0.0014
HIST1H3G	7.30	2.50E-06	0.0014
TNFRSF8	7.27	5.98E-04	0.0060
KIF20A	7.27	1.41E-05	0.0018
CDCA5	7.26	6.08E-06	0.0016
DLGAP5	7.26	1.79E-06	0.0014
ENST000003435280	7.24	3.92E-03	0.0160
MCM10	7.24	2.20E-05	0.0020
ENST000002721980	7.21	4.19E-05	0.0024
IL21	7.10	1.44E-03	0.0092
ENST000002613770	7.06	3.40E-03	0.0147
ENST000002954971	7.03	5.50E-04	0.0058
LINC01281	7.02	1.92E-06	0.0014
DSCC1	7.02	4.07E-05	0.0024
ENST000003298752	6.95	1.99E-02	0.0460
BIRC5	6.95	5.31E-07	0.0014
ENST00000409111	6.95	8.20E-05	0.0030
OXCT2	6.94	4.63E-05	0.0025
MND1	6.93	2.33E-06	0.0014
GEM	6.93	4.82E-04	0.0055
EBI3	6.93	1.25E-03	0.0086
ENST000003051882	6.92	3.11E-05	0.0022
ENST000002500562	6.92	4.31E-05	0.0024
MYBL2	6.82	3.74E-05	0.0023
FAM72D	6.81	2.67E-06	0.0014
TMCC2	6.81	1.13E-04	0.0033
ENST000003173681	6.77	2.89E-04	0.0045
CAVIN3	6.76	5.76E-06	0.0016
ENST000002868351	6.76	9.02E-05	0.0031
AK4P1	6.76	8.93E-06	0.0016

MFSD2A	6.74	8.26E-05	0.0030
HMSD	6.73	1.35E-04	0.0035
TMPOP2	6.72	6.53E-05	0.0029
ENST000002269510	6.71	4.52E-04	0.0054
AK4	6.70	5.56E-07	0.0014
LAG3	6.69	1.47E-03	0.0093
ENST000002994980	6.69	4.55E-03	0.0175
CSF2	6.69	6.78E-05	0.0029
PLA2G4A	6.68	8.00E-05	0.0030
RAD54L	6.65	8.15E-06	0.0016
TEAD4	6.65	6.26E-05	0.0028
ECSCR	6.56	8.19E-05	0.0030
ANGPTL4	6.55	5.03E-06	0.0016
BATF3	6.55	2.99E-03	0.0137
EME1	6.54	1.57E-05	0.0019
PHEX	6.54	1.29E-04	0.0035
C2orf72	6.54	7.98E-05	0.0030
SLC8A3	6.53	1.66E-04	0.0038
PTPRF	6.53	9.62E-06	0.0017
HMMR	6.53	3.26E-05	0.0023
IL4I1	6.51	1.47E-04	0.0036
HIST1H3B	6.50	1.40E-06	0.0014
ENST000002623153	6.50	1.07E-03	0.0080
PDCD1	6.50	1.23E-05	0.0018
SDC4	6.49	6.00E-04	0.0060
TRIP13	6.48	6.41E-05	0.0028
CLDN1	6.48	7.94E-05	0.0030
ZACN	6.46	8.14E-05	0.0030
ENST000002292650	6.45	1.26E-04	0.0035
HIST2H3A	6.43	9.61E-06	0.0017
HIST2H3C	6.43	9.61E-06	0.0017
ENST000003283540	6.42	1.47E-02	0.0370
KCNK5	6.42	3.07E-06	0.0014
FAM72C	6.41	1.24E-03	0.0085
ENST000003016341	6.38	4.22E-05	0.0024
B4GALT2	6.38	7.78E-06	0.0016
INSM1	6.38	8.23E-04	0.0070
C17orf58	6.37	2.03E-04	0.0040
ENST000003576370	6.37	1.28E-03	0.0087
HIST1H2AB	6.36	9.70E-05	0.0031
AIM2	6.34	2.25E-03	0.0117
B3GNT5	6.34	6.60E-05	0.0029
ENST000002938722	6.33	4.55E-04	0.0054
SPRY1	6.30	3.44E-04	0.0048
AC007283.1	6.30	1.13E-04	0.0033
SLC41A2	6.29	1.86E-05	0.0020
ENST000003370002	6.29	1.04E-04	0.0033
MAOA	6.29	2.28E-03	0.0118
GCSHP5	6.28	1.36E-04	0.0035
HIST1H3C	6.28	1.39E-04	0.0036

IL1R2	6.26	2.37E-04	0.0042
AC112777.1	6.26	1.14E-04	0.0033
GLT1D1	6.26	2.08E-04	0.0040
FAM71B	6.24	1.33E-04	0.0035
CEP55	6.23	6.34E-07	0.0014
ENST000002584943	6.22	2.53E-04	0.0043
TNFRSF4	6.21	1.76E-04	0.0039
ENST000002280271	6.21	2.11E-04	0.0041
HSD11B1	6.21	7.35E-05	0.0029
AL139300.1	6.20	1.36E-02	0.0352
HIST1H2BM	6.19	8.38E-05	0.0030
AC010655.2	6.19	3.84E-03	0.0158
ERCC6L	6.19	1.99E-06	0.0014
CCNB2	6.18	7.04E-06	0.0016
DEPDC1	6.17	3.10E-06	0.0014
ENST000002168400	6.17	1.99E-04	0.0040
HIST1H3F	6.16	8.05E-06	0.0016
KIF2C	6.14	9.87E-07	0.0014
AC011893.1	6.14	2.96E-03	0.0136
TEDC2	6.12	2.96E-04	0.0046
OXCT2P1	6.12	3.08E-04	0.0046
AL034430.1	6.12	1.14E-02	0.0312
NTRK2	6.10	1.98E-04	0.0040
SHF	6.10	1.10E-03	0.0080
ENST000003282572	6.10	1.35E-02	0.0349
AC112721.2	6.09	1.59E-03	0.0097
LIF	6.09	5.51E-04	0.0058
POLQ	6.08	3.71E-06	0.0015
ENST000003120870	6.08	2.88E-04	0.0045
ENST000002478331	6.05	7.19E-04	0.0065
TYMS	6.05	1.47E-04	0.0036
DTL	6.05	1.86E-05	0.0020
CCNA2	6.03	1.12E-05	0.0018
GNA15	6.02	1.46E-03	0.0093
AL391832.3	6.01	3.87E-04	0.0050
UHRF1	6.01	5.61E-06	0.0016
ENST000002463140	6.01	3.84E-04	0.0050
HIST1H2AJ	6.00	3.61E-05	0.0023
ENST000002962890	6.00	7.36E-03	0.0234
BARX1	5.99	1.65E-04	0.0037
ENST000003578574	5.97	2.92E-04	0.0045
ENST000003073772	5.97	5.69E-04	0.0059
LHFPL6	5.96	2.59E-03	0.0127
ITPKA	5.96	1.66E-04	0.0037
SHCBP1	5.95	1.30E-05	0.0018
ENST000003405560	5.94	6.97E-03	0.0228
IFNG	5.91	6.86E-05	0.0029
HIST1H1B	5.90	3.04E-06	0.0014
ENST000002972580	5.90	1.03E-04	0.0033
AP003472.2	5.90	3.25E-04	0.0047

CCNB1	5.89	2.59E-06	0.0014
DDIAS	5.88	1.36E-04	0.0035
HIST1H2AH	5.88	1.00E-06	0.0014
AC010655.4	5.88	1.76E-03	0.0102
ZNF887P	5.87	1.94E-04	0.0039
NCAPG	5.87	8.42E-07	0.0014
PTGFRN	5.84	5.75E-05	0.0028
NCAPH	5.84	2.64E-04	0.0044
FAM72A	5.83	2.47E-06	0.0014
ENST000002611921	5.82	3.46E-03	0.0148
HIST1H2AL	5.82	5.47E-05	0.0027
GOLM1	5.82	2.98E-04	0.0046
AP000424.1	5.82	3.34E-04	0.0047
GJB2	5.82	6.87E-04	0.0064
ENST000003791630	5.81	2.29E-04	0.0042
TMEM200B	5.78	1.22E-03	0.0084
ENST000003285960	5.78	1.86E-04	0.0039
ENST000002988380	5.77	1.82E-02	0.0432
AC114803.1	5.77	2.86E-04	0.0045
ESPL1	5.77	2.22E-03	0.0117
HIST1H2AI	5.77	4.22E-05	0.0024
RNU1-120P	5.76	7.25E-04	0.0066
AC006329.1	5.76	7.05E-03	0.0229
E2F8	5.75	7.53E-06	0.0016
RBFOX2	5.74	5.76E-03	0.0202
CDC20	5.74	3.37E-03	0.0146
ATP9A	5.73	3.29E-04	0.0047
CASKIN1	5.73	6.26E-04	0.0062
CALD1	5.71	1.32E-05	0.0018
ENST000002603831	5.71	1.35E-02	0.0350
CDK1	5.70	1.51E-06	0.0014
AC010655.3	5.69	3.75E-04	0.0049
LINC02156	5.69	2.81E-04	0.0045
FAM72B	5.67	1.41E-04	0.0036
MELK	5.67	3.05E-06	0.0014
ENST000002790361	5.66	6.62E-03	0.0220
MKI67	5.66	3.36E-05	0.0023
SULF2	5.65	1.45E-04	0.0036
AL035604.1	5.64	2.59E-04	0.0044
ENST000002951710	5.64	2.65E-04	0.0044
TMPRSS3	5.64	1.55E-05	0.0019
CA2	5.64	4.65E-04	0.0054
IQGAP3	5.63	6.59E-04	0.0063
NCS1	5.63	2.50E-03	0.0124
ENST000003083170	5.61	6.77E-03	0.0223
CENPA	5.61	3.94E-06	0.0015
F12	5.61	2.86E-04	0.0045
ENST000002623002	5.59	4.12E-04	0.0051
ENST000002633600	5.58	7.83E-05	0.0030
HIST1H2BB	5.58	1.19E-05	0.0018

VWA1	5.58	6.41E-04	0.0062
CDC25C	5.58	4.33E-05	0.0024
AC084262.1	5.57	2.93E-04	0.0045
DPCD	5.57	1.07E-03	0.0079
KIF15	5.55	7.38E-06	0.0016
AC084026.1	5.55	4.35E-04	0.0053
CENPW	5.55	9.02E-06	0.0016
GZMH	5.52	8.48E-03	0.0257
RAD51AP1	5.51	2.45E-06	0.0014
IGHV1-3	5.51	3.82E-03	0.0157
STC1	5.50	1.54E-03	0.0096
PPFIA4	5.50	3.30E-06	0.0015
TP53I11	5.49	4.21E-04	0.0052
HIST2H2AA3	5.49	1.03E-03	0.0078
NEK2	5.48	4.54E-05	0.0025
ENST000003294330	5.47	5.11E-04	0.0056
HIST1H2BO	5.46	1.77E-04	0.0039
AL391832.2	5.45	7.91E-05	0.0030
QPCT	5.45	1.34E-03	0.0088
KIFC1	5.43	5.32E-05	0.0027
MIR155HG	5.43	2.74E-06	0.0014
LINC01686	5.42	1.18E-03	0.0083
ENST000003158910	5.42	1.74E-03	0.0102
CDKN3	5.41	6.07E-04	0.0061
CDCA8	5.41	1.19E-05	0.0018
CDC6	5.41	7.40E-05	0.0029
KIF23	5.40	1.20E-06	0.0014
KIF14	5.40	4.08E-04	0.0051
CTHRC1	5.37	4.05E-04	0.0051
HIST1H2BJ	5.37	6.36E-06	0.0016
GINS2	5.36	5.86E-06	0.0016
AC006011.1	5.35	1.12E-03	0.0081
SMIM10	5.35	8.29E-04	0.0071
HJURP	5.34	3.57E-06	0.0015
CXCL8	5.33	3.01E-03	0.0137
PTMS	5.31	5.32E-04	0.0058
TESC	5.30	9.13E-03	0.0269
MCM2	5.29	7.25E-06	0.0016
ENST000003094811	5.28	4.41E-04	0.0053
MYH10	5.28	2.37E-06	0.0014
ZNF697	5.28	3.53E-03	0.0150
STC2	5.27	9.36E-03	0.0273
TOP2A	5.27	1.51E-06	0.0014
EVI5	5.26	6.85E-05	0.0029
RECQL4	5.25	2.30E-05	0.0020
CDCA2	5.24	3.03E-05	0.0022
ZWINT	5.22	3.40E-04	0.0048
AP002852.1	5.21	1.05E-05	0.0017
FRMD5	5.21	8.75E-04	0.0072
RF00017.13	5.20	7.09E-05	0.0029

RF00017.14	5.20	7.09E-05	0.0029
RF00017.21	5.20	7.09E-05	0.0029
RF00017.22	5.20	7.09E-05	0.0029
RN7SL471P	5.20	7.09E-05	0.0029
ENST00000537344	5.19	1.89E-03	0.0106
TTK	5.19	2.24E-05	0.0020
AUNIP	5.19	3.63E-05	0.0023
NAMPTP1	5.18	5.41E-04	0.0058
ANK2	5.17	1.76E-04	0.0039
RNVU1-6	5.17	7.73E-05	0.0030
RXRA	5.17	3.94E-04	0.0050
ZNF704	5.17	2.32E-04	0.0042
ANLN	5.16	3.67E-05	0.0023
ENST000002846373	5.15	1.73E-03	0.0102
P4HA2	5.14	1.99E-04	0.0040
EPB41L4B	5.14	7.60E-04	0.0067
HIST1H3J	5.14	1.50E-05	0.0019
VDR	5.13	2.83E-06	0.0014
CCND1	5.13	5.71E-03	0.0200
CGREF1	5.12	6.75E-05	0.0029
TROAP	5.12	4.85E-05	0.0026
HPDL	5.10	3.99E-05	0.0024
TRPC3	5.10	2.96E-03	0.0136
ARG2	5.10	1.76E-04	0.0039
RHOV	5.10	7.16E-04	0.0065
ENST000002650561	5.09	2.43E-05	0.0021
ENST000002998215	5.09	2.22E-02	0.0498
AC007240.1	5.09	2.69E-04	0.0044
GSDME	5.09	2.49E-03	0.0124
ENST000002621245	5.08	4.06E-03	0.0163
SLC43A3	5.08	4.75E-05	0.0026
CKAP2L	5.08	2.17E-03	0.0115
COCH	5.08	1.29E-03	0.0087
GGH	5.07	7.09E-06	0.0016
CCDC74B	5.06	5.03E-03	0.0185
FBXO43	5.05	2.99E-05	0.0022
SEMA7A	5.05	1.08E-04	0.0033
FAM81A	5.05	1.08E-03	0.0080
ORC1	5.05	2.33E-06	0.0014
ENST000002572610	5.04	1.55E-02	0.0384
ICAM5	5.04	1.10E-03	0.0080
ENST000002875980	5.04	1.71E-05	0.0020
LMNA	5.04	5.31E-04	0.0058
LINC02389	5.03	7.12E-04	0.0065
AURKA	5.03	3.38E-06	0.0015
SLAMF7	5.01	5.91E-05	0.0028
SLC45A3	5.00	8.03E-04	0.0070
ENST000002436620	4.99	6.45E-05	0.0028
IL23R	4.98	1.36E-05	0.0018
MIR210HG	4.97	2.99E-04	0.0046

SORD2P	4.96	6.29E-04	0.0062
ENST000003408481	4.95	8.37E-04	0.0071
RNU1-122P	4.95	8.80E-03	0.0263
ENST000003004030	4.94	1.70E-05	0.0020
AKAP12	4.94	2.02E-03	0.0110
CDC25A	4.94	2.42E-05	0.0021
DUPD1	4.93	7.65E-04	0.0068
FTH1P22	4.92	1.88E-05	0.0020
MAK	4.92	1.83E-03	0.0104
KIF24	4.92	2.23E-05	0.0020
PPP1R26	4.91	1.51E-03	0.0094
RNU2-63P	4.91	1.18E-04	0.0034
AP000919.2	4.90	8.47E-04	0.0071
ENST000002680491	4.90	1.81E-02	0.0428
ZNF860	4.90	1.06E-03	0.0079
KIF4A	4.89	6.29E-06	0.0016
JPH1	4.89	1.36E-03	0.0089
DPYSL4	4.88	7.76E-04	0.0068
LINC00475	4.88	1.31E-03	0.0087
CLSPN	4.88	5.54E-06	0.0016
TNFRSF9	4.88	1.76E-04	0.0039
FOS	4.87	1.12E-03	0.0081
SLC16A9	4.87	1.54E-03	0.0095
NAMPT	4.87	5.19E-06	0.0016
PLTP	4.87	1.22E-03	0.0084
DIAPH3	4.87	1.49E-04	0.0036
PCLAF	4.87	1.06E-05	0.0017
GPR176	4.86	8.75E-04	0.0072
SKA1	4.86	2.08E-05	0.0020
CA9	4.86	8.84E-04	0.0073
FOSL1	4.86	8.70E-04	0.0072
MIR5087	4.86	5.98E-03	0.0206
POC1A	4.86	1.04E-04	0.0033
MELTF	4.85	1.88E-03	0.0106
PROC	4.85	1.32E-03	0.0088
VAV2	4.84	6.76E-04	0.0064
CLEC17A	4.84	1.19E-04	0.0034
FASLG	4.84	1.03E-03	0.0078
UBXN10	4.83	1.01E-03	0.0078
NT5DC2	4.82	2.42E-04	0.0042
CD200	4.81	4.12E-04	0.0051
CENPU	4.81	5.79E-05	0.0028
SIPA1L2	4.81	2.33E-05	0.0021
DEPDC1B	4.80	4.63E-06	0.0016
NR4A1	4.80	1.49E-02	0.0373
SPC24	4.80	4.05E-06	0.0016
ENST000003092760	4.79	1.04E-03	0.0078
IL5	4.79	1.04E-03	0.0078
CDCA3	4.78	9.68E-05	0.0031
GSTA4	4.78	5.16E-04	0.0057

TUBA1B	4.76	1.98E-06	0.0014
HASPIN	4.76	6.28E-06	0.0016
FSCN1	4.75	7.87E-05	0.0030
BEND5	4.74	1.37E-04	0.0035
DUSP4	4.74	3.96E-04	0.0051
AL021578.1	4.74	1.01E-03	0.0078
AC068446.2	4.74	1.07E-03	0.0080
CAMKV	4.74	1.07E-03	0.0080
DUSP2	4.73	1.75E-05	0.0020
TK1	4.72	3.68E-03	0.0154
EPOP	4.72	3.15E-04	0.0046
ASF1B	4.71	2.65E-04	0.0044
OSBPL10	4.71	1.50E-04	0.0036
ABCA1	4.71	7.33E-03	0.0234
ANGPTL6	4.71	3.26E-05	0.0023
IER3	4.70	6.91E-06	0.0016
AL645608.8	4.70	1.29E-03	0.0087
SLC28A3	4.70	1.29E-03	0.0087
TNFSF11	4.69	2.19E-04	0.0041
NTRK1	4.69	1.92E-03	0.0107
WNT10A	4.68	1.67E-02	0.0405
CTNNAL1	4.68	2.40E-05	0.0021
B4GALT6	4.68	5.61E-04	0.0059
EGR3	4.67	2.70E-03	0.0130
GNG12	4.67	1.14E-03	0.0082
HIST1H1T	4.67	1.14E-03	0.0082
ZNF560	4.67	1.14E-03	0.0082
JADE3	4.66	9.76E-05	0.0031
AL031846.1	4.65	1.51E-03	0.0094
CD9	4.65	4.34E-05	0.0024
LOXL3	4.64	2.26E-04	0.0041
CABLES1	4.64	1.74E-04	0.0038
FGF2	4.63	4.88E-04	0.0055
DNAJC12	4.63	1.13E-04	0.0033
ENST000003461410	4.63	1.45E-02	0.0368
LINC01480	4.62	4.50E-05	0.0025
BRCA1	4.60	2.58E-06	0.0014
KNL1	4.60	5.28E-06	0.0016
UACA	4.59	3.38E-04	0.0048
CDK14	4.59	3.82E-03	0.0157
CAMK1	4.59	5.58E-06	0.0016
ENST000003024500	4.59	2.30E-05	0.0020
DOCK1	4.59	6.93E-03	0.0227
PIF1	4.59	6.68E-06	0.0016
ENST000002997985	4.58	1.36E-03	0.0089
KIAA1211	4.58	1.36E-03	0.0089
SPHK1	4.58	6.64E-04	0.0063
C16orf45	4.57	2.76E-04	0.0044
PHKA1	4.57	9.80E-04	0.0077
HDC	4.56	2.98E-04	0.0046

AP001189.5	4.56	1.43E-02	0.0363
C4orf47	4.54	5.49E-04	0.0058
AC007032.1	4.54	1.12E-02	0.0308
HIST1H4I	4.54	7.22E-06	0.0016
HIST1H2BL	4.54	1.05E-05	0.0017
RNVU1-4	4.54	2.58E-03	0.0127
RF00003.22	4.53	3.74E-05	0.0023
NEK6	4.53	2.47E-03	0.0124
FOXP4-AS1	4.53	1.42E-03	0.0091
HIST1H1A	4.52	8.55E-05	0.0030
ENST000003020031	4.52	1.20E-02	0.0322
DDIT4	4.52	2.73E-04	0.0044
ASPM	4.52	4.64E-05	0.0025
CENPS-CORT	4.51	1.01E-02	0.0288
LINC00877	4.51	1.01E-02	0.0288
SNORA21B	4.51	1.45E-03	0.0093
ENST000003364301	4.51	1.70E-02	0.0411
ENST000002909430	4.50	3.86E-03	0.0158
ENST000002840060	4.50	1.09E-02	0.0303
HIST2H4A	4.49	2.30E-06	0.0014
HIST1H4A	4.49	8.06E-06	0.0016
CKB	4.48	2.57E-04	0.0043
RGS16	4.48	2.45E-03	0.0123
NUF2	4.47	2.91E-06	0.0014
AL445524.1	4.46	6.26E-04	0.0062
SPC25	4.46	2.61E-05	0.0021
ENST000003107751	4.44	2.21E-04	0.0041
ENST000003352091	4.44	1.39E-05	0.0018
RIMKLA	4.43	1.22E-05	0.0018
HIST3H2BA	4.43	2.43E-04	0.0042
ERICD	4.42	5.52E-03	0.0196
WASF1	4.41	1.07E-03	0.0080
AP000781.2	4.40	4.32E-03	0.0169
AC004816.1	4.40	8.78E-03	0.0263
SNORA24	4.40	2.49E-03	0.0124
SPAG5	4.38	6.15E-06	0.0016
SMC1B	4.38	3.89E-03	0.0159
SPAG1	4.37	1.06E-03	0.0079
RNVU1-3	4.37	3.62E-04	0.0049
LDHA	4.37	3.09E-06	0.0014
TFRC	4.36	1.33E-05	0.0018
CENPI	4.35	4.11E-05	0.0024
ARHGEF39	4.35	3.57E-05	0.0023
AC116366.2	4.35	2.51E-03	0.0125
PFKFB4	4.34	8.65E-05	0.0030
ENST000003228861	4.34	5.50E-05	0.0027
TMPRSS6	4.33	8.16E-05	0.0030
MPZL2	4.33	1.33E-05	0.0018
UNC13B	4.33	2.13E-03	0.0114
CPM	4.33	3.95E-04	0.0050

PTTG1	4.32	1.97E-05	0.0020
RF00012.19	4.32	1.88E-04	0.0039
ENST000002214130	4.32	1.01E-03	0.0078
NME1-NME2	4.32	9.22E-04	0.0075
ENST000003476170	4.31	8.28E-03	0.0253
MPP2	4.31	4.19E-03	0.0166
RNU1-72P	4.31	2.37E-03	0.0121
CU633904.1	4.31	1.48E-04	0.0036
LINC01132	4.30	1.13E-03	0.0081
RIBC2	4.30	3.49E-04	0.0048
AL357992.1	4.29	7.82E-04	0.0069
B4GALNT1	4.29	5.15E-04	0.0057
ENST000002902770	4.28	1.91E-02	0.0447
RAD51	4.28	1.60E-05	0.0019
RPL39L	4.27	8.77E-05	0.0031
RNU1-130P	4.27	6.47E-03	0.0217
LTA	4.27	3.08E-05	0.0022
CENPF	4.26	2.55E-05	0.0021
SYNGR3	4.26	3.42E-04	0.0048
FANCL	4.25	1.27E-05	0.0018
JAM3	4.22	1.10E-04	0.0033
SCCPDH	4.22	7.22E-05	0.0029
CKS2	4.22	3.93E-06	0.0015
TP53I3	4.22	2.32E-04	0.0042
SCD	4.21	3.59E-05	0.0023
ENST000003351830	4.20	1.63E-05	0.0019
LAMB3	4.19	5.39E-04	0.0058
FEN1	4.19	8.67E-06	0.0016
ANKRD37	4.18	2.54E-05	0.0021
SPAG4	4.18	3.33E-03	0.0145
CCDC150	4.17	2.46E-05	0.0021
ENST00000567345	4.17	2.11E-02	0.0481
COL6A3	4.17	4.32E-03	0.0169
SNORD3B-1	4.16	3.67E-03	0.0153
GTSE1	4.16	1.66E-05	0.0019
CIT	4.15	9.03E-06	0.0016
E2F7	4.15	1.15E-03	0.0082
COLGALT2	4.14	1.31E-03	0.0087
ITPRIPL2	4.14	4.96E-04	0.0056
CENPM	4.14	1.74E-04	0.0038
FAM131B	4.14	8.26E-03	0.0253
EMP1	4.13	2.08E-03	0.0112
ENST000002606052	4.13	5.14E-03	0.0188
SPTBN4	4.13	7.54E-03	0.0238
CRIM1	4.12	6.83E-04	0.0064
TYMSOS	4.12	9.83E-03	0.0283
ENST000003132852	4.12	1.14E-05	0.0018
RTKN	4.11	1.77E-02	0.0422
ETV4	4.11	4.51E-04	0.0054
EGR1	4.10	8.40E-04	0.0071

DYRK3	4.10	9.08E-05	0.0031
HSD11B1-AS1	4.10	1.20E-04	0.0034
AC099552.1	4.10	2.70E-03	0.0130
NDRG4	4.08	1.73E-03	0.0102
CKS1B	4.08	5.48E-06	0.0016
COX20P2	4.07	1.12E-02	0.0309
SKA3	4.05	5.36E-06	0.0016
IL13	4.05	7.58E-03	0.0239
CHRNA6	4.05	2.43E-03	0.0123
FLT1	4.05	8.31E-04	0.0071
ESCO2	4.04	6.45E-05	0.0028
NKG7	4.03	7.42E-03	0.0236
PARD3	4.02	5.82E-04	0.0060
CU633906.1	4.02	1.64E-04	0.0037
RAC3	4.01	1.25E-02	0.0332
PKMYT1	4.01	8.46E-05	0.0030
ARFGEF3	4.00	2.18E-05	0.0020
FAM111B	3.98	7.81E-06	0.0016
SLC29A1	3.98	4.27E-04	0.0052
AC084026.2	3.98	3.66E-03	0.0153
BUB1B	3.98	1.09E-02	0.0302
C17orf53	3.98	3.36E-04	0.0047
TPI1	3.97	1.19E-05	0.0018
PGAM1	3.97	1.00E-05	0.0017
DNAH11	3.97	1.28E-05	0.0018
IGFBP2	3.97	1.42E-03	0.0091
FZD5	3.97	1.17E-03	0.0083
HIST1H4L	3.96	2.56E-05	0.0021
POLE2	3.96	5.37E-05	0.0027
ENST000003027590	3.96	1.55E-03	0.0096
MCM4	3.95	6.00E-06	0.0016
GOLIM4	3.95	3.01E-03	0.0137
LGALS1	3.95	5.88E-05	0.0028
PIH1D2	3.94	5.93E-03	0.0205
SMS	3.94	8.79E-05	0.0031
SRD5A3	3.94	1.20E-03	0.0084
CCNE2	3.94	8.72E-06	0.0016
RF00003.25	3.92	1.93E-03	0.0107
CU634019.1	3.92	1.78E-04	0.0039
GINS4	3.92	4.49E-04	0.0053
HIST1H2BF	3.91	1.83E-04	0.0039
TRIB1	3.91	1.80E-05	0.0020
PLK4	3.91	6.71E-06	0.0016
HIST1H2BI	3.91	3.55E-05	0.0023
PRR5L	3.91	1.37E-04	0.0035
CENPN	3.91	1.24E-05	0.0018
HIST1H2BK	3.90	4.98E-06	0.0016
EPDR1	3.90	8.35E-03	0.0254
ENST000003423860	3.90	7.64E-04	0.0068
DUSP6	3.90	1.79E-03	0.0103

GAPDH	3.90	3.90E-06	0.0015
MED12L	3.89	1.37E-03	0.0089
HIST1H2BE	3.89	5.32E-05	0.0027
PRR11	3.89	4.63E-05	0.0025
HIST2H3D	3.89	1.72E-05	0.0020
AURKB	3.88	1.40E-05	0.0018
ENST000002703011	3.88	9.25E-04	0.0075
CDT1	3.87	8.90E-04	0.0073
UNQ6494	3.87	1.60E-04	0.0037
SAPCD2	3.85	2.53E-05	0.0021
TUBB	3.85	7.01E-06	0.0016
ENST000001894440	3.84	2.97E-03	0.0136
LINC01943	3.84	1.83E-03	0.0104
TBX21	3.83	1.94E-05	0.0020
OSCP1	3.83	5.86E-03	0.0204
ENST000002603590	3.83	9.51E-05	0.0031
WDR34	3.83	1.73E-04	0.0038
AZIN1-AS1	3.83	1.48E-04	0.0036
GINS1	3.83	2.11E-05	0.0020
PHLDA1	3.82	4.64E-04	0.0054
H2AFX	3.82	1.28E-04	0.0035
PACSIN3	3.82	9.85E-04	0.0077
IRF4	3.81	2.73E-05	0.0021
STRIP2	3.81	2.03E-04	0.0040
SLC8A1	3.81	1.37E-04	0.0035
KIF11	3.81	5.47E-06	0.0016
HIST1H2AM	3.81	2.20E-05	0.0020
VLDLR	3.81	1.52E-04	0.0036
PLEKHA7	3.80	4.80E-03	0.0180
SERPINE2	3.80	1.11E-02	0.0306
ENST000003278922	3.80	2.58E-03	0.0126
GINS3	3.80	8.43E-05	0.0030
RNU5E-6P	3.80	7.97E-05	0.0030
CENPH	3.80	2.51E-04	0.0043
POLR3G	3.80	1.06E-04	0.0033
SLC25A10	3.79	3.26E-03	0.0143
CU639417.1	3.79	1.35E-05	0.0018
H2BFS	3.79	1.35E-05	0.0018
CORO1C	3.78	1.12E-03	0.0081
HIST1H2AE	3.78	1.85E-05	0.0020
ADPRH	3.77	2.26E-04	0.0041
EIF4EP2	3.77	2.39E-03	0.0122
RFC3	3.77	8.40E-06	0.0016
HIST1H3A	3.76	1.24E-05	0.0018
HIST1H3D	3.76	1.18E-02	0.0320
UBE2T	3.75	1.54E-05	0.0019
HIST1H4C	3.75	4.98E-06	0.0016
AP001453.3	3.75	1.31E-02	0.0341
ZC3H12C	3.74	1.37E-03	0.0089
TEAD1	3.74	4.59E-03	0.0176

CD83	3.74	3.74E-03	0.0155
FAM234B	3.73	4.21E-04	0.0052
TUBA1C	3.73	6.44E-06	0.0016
CLECL1	3.72	7.82E-04	0.0069
CXCR5	3.71	1.60E-02	0.0394
ENST000003509971	3.71	6.30E-04	0.0062
C2CD4D-AS1	3.71	1.48E-04	0.0036
NEFH	3.71	5.48E-05	0.0027
KIF18B	3.70	1.94E-03	0.0107
PVR	3.70	2.69E-04	0.0044
STMN1	3.70	2.17E-05	0.0020
AC051619.8	3.70	1.16E-04	0.0034
TMEM273	3.69	2.72E-05	0.0021
ENST000003195182	3.69	1.09E-02	0.0302
HOPX	3.69	4.51E-03	0.0174
CFAP58	3.69	2.34E-03	0.0120
PSMC3IP	3.69	9.75E-05	0.0031
ENST000002632741	3.68	1.64E-05	0.0019
TPI1P1	3.68	1.85E-03	0.0105
TUBBP1	3.67	1.20E-02	0.0324
PIP5K1B	3.67	1.48E-03	0.0093
KCNN4	3.67	1.41E-05	0.0018
CHEK1	3.67	6.61E-06	0.0016
LINC01679	3.66	6.72E-04	0.0064
HIST1H4D	3.66	5.38E-06	0.0016
WDHD1	3.66	6.15E-06	0.0016
C15orf48	3.66	1.19E-03	0.0083
CDR2L	3.66	1.12E-02	0.0308
CYP27B1	3.65	6.73E-04	0.0064
PAICS	3.65	1.04E-05	0.0017
LGALSL	3.65	7.75E-05	0.0030
ARHGEF37	3.65	2.63E-03	0.0128
ADGRA3	3.64	1.84E-05	0.0020
DBN1	3.64	2.90E-03	0.0135
ENST000003285141	3.63	1.40E-02	0.0357
HIST1H2AG	3.63	5.86E-05	0.0028
UBE2S	3.62	5.84E-05	0.0028
MAD2L1	3.62	1.48E-05	0.0019
CYP51A1	3.62	1.05E-03	0.0079
UCK2	3.60	1.00E-04	0.0032
NCAPG2	3.59	3.58E-04	0.0049
HIST1H2BH	3.59	5.10E-05	0.0027
GCK	3.59	1.52E-02	0.0379
CENPS	3.59	1.05E-04	0.0033
ZNF695	3.59	1.13E-03	0.0081
FANCA	3.59	1.29E-04	0.0035
CHST10	3.58	1.56E-03	0.0096
HIST1H3H	3.57	3.35E-05	0.0023
SNORA51	3.57	1.47E-03	0.0093
IRF5	3.57	3.35E-04	0.0047

AC254633.1	3.57	1.74E-03	0.0102
NRN1	3.57	1.18E-03	0.0083
DUSP5	3.57	3.91E-03	0.0160
ZNRF1	3.56	6.44E-04	0.0062
FGF11	3.56	2.17E-05	0.0020
ANKRD33B	3.56	3.20E-03	0.0142
TCF19	3.56	1.12E-05	0.0018
TNFSF14	3.55	5.99E-04	0.0060
HMGB3	3.55	3.52E-05	0.0023
ME3	3.54	1.33E-04	0.0035
PRC1	3.54	7.21E-06	0.0016
ENST000002645523	3.54	1.85E-04	0.0039
NUSAP1	3.53	2.78E-05	0.0021
ENST000002498060	3.53	7.89E-04	0.0069
SGO1	3.53	7.45E-04	0.0067
CLIC4	3.53	1.21E-02	0.0325
PARVB	3.53	6.68E-05	0.0029
KDELR3	3.52	4.81E-03	0.0180
HIST2H2BF	3.52	3.37E-05	0.0023
PGK1	3.51	2.15E-05	0.0020
ENST000003253071	3.51	3.57E-05	0.0023
IL12RB2	3.51	1.08E-03	0.0080
AP000880.1	3.51	1.73E-02	0.0414
ABHD17C	3.51	1.58E-04	0.0037
KIF18A	3.50	8.22E-06	0.0016
ENST000000090411	3.50	3.06E-03	0.0139
NDFIP2	3.50	1.94E-03	0.0107
NOL3	3.49	2.03E-03	0.0110
Z93241.1	3.49	3.61E-03	0.0152
ENST000003027631	3.49	7.15E-05	0.0029
LRP2	3.49	1.35E-02	0.0349
FABP5	3.48	4.85E-03	0.0181
ENST000003023620	3.48	1.23E-04	0.0034
MTFR2	3.48	3.08E-05	0.0022
VTRNA1-3	3.48	1.32E-03	0.0087
TRAIP	3.48	5.59E-04	0.0059
RF00003.28	3.48	1.88E-03	0.0106
RF00003.34	3.48	1.88E-03	0.0106
AC103591.3	3.46	1.28E-02	0.0336
LINC00881	3.46	2.42E-03	0.0122
HIST1H2AD	3.45	9.22E-05	0.0031
HOMER1	3.45	4.05E-04	0.0051
ID2	3.44	5.36E-04	0.0058
KRT7	3.44	1.53E-03	0.0095
MT-TP	3.44	5.16E-03	0.0188
NME1	3.44	3.05E-05	0.0022
ATAD5	3.44	4.31E-05	0.0024
SNORA74D	3.44	1.20E-04	0.0034
ENST000003615720	3.44	1.28E-03	0.0086
N4BP3	3.43	5.75E-04	0.0060

NPM3	3.43	5.92E-05	0.0028
BLM	3.43	2.50E-05	0.0021
LONP1	3.43	8.40E-06	0.0016
TNFSF9	3.43	4.84E-03	0.0181
LINC01353	3.42	1.79E-02	0.0425
ENST000003549190	3.42	9.38E-05	0.0031
LINC00892	3.42	4.55E-04	0.0054
ENST000002218560	3.42	4.68E-04	0.0054
CTLA4	3.41	7.48E-06	0.0016
FAM57A	3.40	1.65E-03	0.0099
MUC1	3.40	2.04E-03	0.0110
SPRED1	3.40	9.16E-04	0.0074
PIK3AP1	3.40	2.62E-03	0.0128
ATF3	3.39	1.27E-03	0.0086
ENST000003299620	3.39	1.10E-03	0.0080
LAYN	3.39	1.24E-02	0.0330
AC093512.2	3.39	2.07E-03	0.0111
MID1	3.38	2.11E-03	0.0113
RAD54B	3.38	2.08E-05	0.0020
NETO2	3.38	1.85E-03	0.0105
AP002414.2	3.38	5.26E-03	0.0190
AC004069.1	3.38	2.75E-03	0.0131
PLK2	3.38	3.44E-04	0.0048
AC092718.4	3.37	2.38E-04	0.0042
HSPD1	3.37	1.75E-05	0.0020
IL18RAP	3.37	1.54E-04	0.0036
ENST000002863981	3.37	1.59E-03	0.0097
RF00003.31	3.37	3.06E-04	0.0046
PYCR1	3.37	4.04E-04	0.0051
MIR222HG	3.36	1.68E-04	0.0038
HIST1H2BC	3.36	1.04E-05	0.0017
MCM7	3.36	1.85E-05	0.0020
OIP5	3.35	4.39E-05	0.0025
MCAM	3.35	8.84E-05	0.0031
SUV39H2	3.34	1.27E-04	0.0035
C2orf48	3.34	7.38E-04	0.0066
KIF9	3.34	1.23E-03	0.0085
BNIP3	3.34	1.90E-05	0.0020
ENST000003286970	3.34	7.67E-05	0.0030
CCNF	3.34	4.33E-05	0.0024
LINC02416	3.33	4.40E-04	0.0053
RNU5E-4P	3.33	8.52E-03	0.0258
NFIL3	3.33	4.91E-03	0.0183
STIL	3.33	2.86E-05	0.0022
AC135178.5	3.32	4.49E-03	0.0173
HIST1H4J	3.32	3.77E-05	0.0023
JAG2	3.32	7.59E-03	0.0239
RFC5	3.31	4.12E-05	0.0024
AC020904.2	3.31	2.45E-03	0.0123
ERI2	3.31	1.95E-05	0.0020

KNSTRN	3.31	3.72E-04	0.0049
GBE1	3.30	1.70E-05	0.0020
ATP8B4	3.29	1.03E-02	0.0292
KIF7	3.29	6.55E-04	0.0063
ODC1	3.28	2.93E-04	0.0045
CCDC58	3.28	2.61E-05	0.0021
IL3	3.28	1.46E-02	0.0368
ENO1	3.28	4.06E-04	0.0051
BCAT1	3.28	2.79E-03	0.0132
ENST000002958302	3.28	2.39E-04	0.0042
NMB	3.28	1.91E-04	0.0039
CPNE7	3.28	1.01E-02	0.0287
TTC26	3.28	5.43E-04	0.0058
UPK1A-AS1	3.28	5.86E-05	0.0028
PALLD	3.27	5.85E-03	0.0204
TRAF4	3.27	8.93E-05	0.0031
ALDOA	3.27	1.00E-05	0.0017
NUDT10	3.27	9.37E-04	0.0075
ENST000002886703	3.27	7.71E-03	0.0242
PLS3	3.26	1.28E-03	0.0086
HIST1H4F	3.25	1.13E-05	0.0018
ECT2	3.25	1.82E-04	0.0039
ZGRF1	3.24	2.09E-05	0.0020
SHC4	3.24	1.67E-03	0.0100
RHEBL1	3.24	3.28E-03	0.0144
ENST000002848112	3.24	2.36E-03	0.0121
GK	3.23	2.63E-05	0.0021
HIST1H2BG	3.23	3.39E-05	0.0023
HIST2H2AB	3.22	5.26E-05	0.0027
SH3D21	3.22	1.07E-04	0.0033
RANBP1	3.21	1.76E-05	0.0020
EIF4EBP1	3.21	6.79E-04	0.0064
SLC7A5	3.21	5.96E-04	0.0060
KPNA2	3.21	6.08E-05	0.0028
BRCA2	3.21	5.87E-05	0.0028
PCNA	3.21	1.00E-04	0.0032
KCNQ5	3.21	1.06E-04	0.0033
SLC35F2	3.21	1.33E-04	0.0035
CENPE	3.19	9.85E-06	0.0017
WARS	3.19	5.45E-05	0.0027
VDAC1	3.19	9.50E-06	0.0017
CD70	3.19	8.74E-05	0.0031
ZNF215	3.19	1.24E-02	0.0330
TMEM97	3.19	2.44E-04	0.0042
MSH5-SAPCD1	3.19	6.32E-04	0.0062
CIART	3.18	8.57E-04	0.0072
TPRG1	3.18	2.00E-02	0.0462
ENST000003522971	3.18	2.26E-05	0.0020
CORO6	3.18	2.31E-04	0.0042
CYTOR	3.18	4.96E-05	0.0026

SAMD15	3.18	1.21E-02	0.0325
DHCR24	3.17	1.58E-04	0.0037
MYL6B	3.17	4.29E-04	0.0052
ENST000002637411	3.17	8.23E-03	0.0252
PTCH2	3.17	5.12E-04	0.0056
SQLE	3.17	8.85E-05	0.0031
ENST000002541930	3.16	2.87E-04	0.0045
CDKN1A	3.16	5.96E-04	0.0060
TIMELESS	3.16	5.94E-05	0.0028
SLC16A14	3.15	1.25E-03	0.0086
PSRC1	3.14	6.13E-05	0.0028
SGO2	3.14	9.42E-05	0.0031
ENST000003516250	3.13	3.51E-05	0.0023
AC234772.2	3.13	4.24E-04	0.0052
ENST000002566860	3.13	2.73E-05	0.0021
ST7	3.12	3.48E-04	0.0048
BUB1	3.12	6.98E-03	0.0228
HSPE1	3.11	2.66E-05	0.0021
GLYATL1	3.11	1.37E-02	0.0353
SLC16A3	3.10	2.29E-05	0.0020
H2AFZ	3.10	2.80E-05	0.0021
NAB2	3.10	3.23E-05	0.0023
DBIL5P	3.10	2.00E-03	0.0109
GLB1L2	3.09	6.15E-03	0.0210
BATF	3.09	6.83E-04	0.0064
H1F0	3.09	5.65E-05	0.0028
GMNN	3.09	3.79E-05	0.0023
HIST2H2AA4	3.09	1.72E-03	0.0101
PMCH	3.09	3.32E-04	0.0047
ENST000000117000	3.09	2.46E-03	0.0123
DTYMK	3.08	1.08E-04	0.0033
TOMM34	3.08	3.10E-05	0.0022
EPAS1	3.08	1.11E-03	0.0080
RPL22L1	3.08	5.19E-05	0.0027
TLN2	3.08	6.31E-04	0.0062
CENPV	3.07	2.63E-04	0.0044
ARNTL2	3.07	2.16E-04	0.0041
HELLS	3.07	1.98E-05	0.0020
RF00494.3	3.07	3.18E-03	0.0141
RRAS2	3.06	4.32E-04	0.0052
AHCY	3.06	5.57E-05	0.0027
NDC80	3.05	1.53E-05	0.0019
ENST000002973231	3.05	1.12E-03	0.0081
ENST000002114020	3.05	2.87E-03	0.0134
COQ3	3.05	7.07E-05	0.0029
SPRED2	3.05	2.39E-03	0.0122
CCDC86	3.05	3.63E-04	0.0049
ETV5	3.05	2.24E-03	0.0117
NOD2	3.04	4.59E-04	0.0054
ENST000003270350	3.04	4.12E-05	0.0024

HIVEP3	3.04	1.31E-04	0.0035
CBX2	3.03	1.80E-03	0.0104
RPA3	3.03	7.00E-05	0.0029
EDARADD	3.03	1.19E-02	0.0321
AL133215.2	3.03	4.78E-03	0.0180
ENST000002860310	3.03	4.23E-03	0.0167
ZWILCH	3.01	7.69E-05	0.0030
ENST000002795501	3.01	5.55E-04	0.0059
AL031777.3	3.01	9.03E-05	0.0031
DNMT3B	3.01	3.10E-04	0.0046
HIST1H2BD	3.01	1.45E-04	0.0036
ANKRD18DP	3.00	6.26E-03	0.0212
CAVIN1	3.00	1.05E-04	0.0033
ENST000002459341	3.00	1.40E-04	0.0036
TSPAN13	3.00	1.32E-03	0.0088
RHOB	3.00	6.25E-04	0.0062
PSD	3.00	4.47E-03	0.0173
C19orf48	2.99	6.78E-05	0.0029
ENST000002298541	2.98	2.93E-05	0.0022
BCL2L1	2.98	1.51E-03	0.0094
RGS3	2.98	3.32E-05	0.0023
MIF	2.98	1.39E-05	0.0018
ENST000002528160	2.98	7.23E-05	0.0029
HIST1H4B	2.98	1.31E-04	0.0035
CDC42EP4	2.97	4.94E-03	0.0183
PLAGL2	2.97	2.53E-04	0.0043
TRIP10	2.97	1.88E-02	0.0442
39692	2.97	2.49E-03	0.0124
AGRN	2.97	1.50E-04	0.0036
RPSAP13	2.97	1.78E-02	0.0425
ENST000002233360	2.97	2.60E-03	0.0127
VEGFA	2.96	1.09E-02	0.0303
TLE6	2.96	3.06E-03	0.0139
TBC1D12	2.96	1.07E-04	0.0033
HIST2H2BE	2.96	3.13E-05	0.0022
AC008074.2	2.95	9.03E-03	0.0267
DNA2	2.95	9.73E-05	0.0031
PIMREG	2.95	1.85E-03	0.0105
ENST000002748910	2.95	3.53E-03	0.0150
C4orf46	2.95	2.35E-04	0.0042
TMPO-AS1	2.94	8.15E-05	0.0030
MRTO4	2.94	1.36E-04	0.0035
ENST000002965090	2.94	1.68E-05	0.0020
PIM3	2.94	3.24E-05	0.0023
ENST000003364521	2.94	1.85E-05	0.0020
ENST000002657532	2.94	9.09E-04	0.0074
MTHFD2	2.94	4.09E-04	0.0051
RF00003.1	2.93	2.03E-03	0.0110
CDK2AP1	2.93	2.09E-05	0.0020
ELL2	2.93	9.39E-05	0.0031

BEND4	2.93	6.56E-03	0.0219
FANCI	2.93	4.26E-04	0.0052
AC108879.1	2.92	2.63E-03	0.0128
SLC1A5	2.92	4.64E-04	0.0054
IFI30	2.92	2.51E-03	0.0125
CHAF1A	2.92	2.77E-04	0.0044
C19orf57	2.92	1.75E-03	0.0102
ALDH1B1	2.92	7.36E-05	0.0029
LYAR	2.92	8.91E-05	0.0031
ENST000002712770	2.91	8.39E-04	0.0071
CENPO	2.91	1.88E-04	0.0039
ENST00000580163	2.90	2.44E-03	0.0123
PDSS1	2.90	9.50E-05	0.0031
DCTPP1	2.90	3.66E-05	0.0023
TUBB4B	2.90	1.54E-05	0.0019
AL138759.1	2.89	1.86E-02	0.0439
RAN	2.89	2.76E-05	0.0021
FAM83D	2.89	3.36E-05	0.0023
RNF122	2.89	1.46E-04	0.0036
CRYBG2	2.89	1.22E-02	0.0326
ZNF367	2.89	2.60E-05	0.0021
FURIN	2.88	4.45E-03	0.0172
RHPN2	2.88	2.01E-04	0.0040
RMI2	2.88	2.43E-05	0.0021
THOP1	2.88	3.10E-05	0.0022
ANP32E	2.88	3.34E-05	0.0023
FANCD2	2.88	6.38E-05	0.0028
DMC1	2.88	6.12E-05	0.0028
ENST000003533791	2.88	1.55E-02	0.0384
CHST2	2.88	1.00E-02	0.0286
AC007952.4	2.87	2.23E-03	0.0117
AL606834.1	2.87	1.93E-03	0.0107
PNMA2	2.87	7.57E-03	0.0239
TIPIN	2.87	1.53E-04	0.0036
AC147651.3	2.86	5.53E-04	0.0059
ENST000000031002	2.86	6.26E-05	0.0028
MRPL37	2.86	1.61E-05	0.0019
ENST000003386310	2.86	1.19E-04	0.0034
HMGB2	2.86	1.74E-05	0.0020
AC004832.3	2.86	5.37E-04	0.0058
RNU5E-1	2.86	2.83E-03	0.0133
FOXM1	2.85	8.45E-05	0.0030
ENST000003252140	2.85	1.70E-02	0.0410
BOLA3-AS1	2.85	1.94E-03	0.0107
KIF3B	2.85	2.24E-04	0.0041
SPDL1	2.85	2.16E-05	0.0020
REEP2	2.84	1.97E-04	0.0040
GPAT2	2.84	3.50E-03	0.0149
GAS2L3	2.84	1.91E-04	0.0039
AC012073.1	2.84	2.71E-03	0.0130

HSP90AB1	2.83	7.92E-05	0.0030
DHFR	2.83	1.88E-05	0.0020
PRMT1	2.83	8.41E-05	0.0030
GNGT2	2.83	4.21E-03	0.0167
ZNF768	2.83	4.67E-04	0.0054
TANC1	2.83	9.65E-04	0.0076
PPIAP22	2.83	9.97E-03	0.0285
IL2RA	2.82	3.32E-04	0.0047
NAPSA	2.82	1.76E-04	0.0039
TNFAIP3	2.82	3.40E-04	0.0048
SHMT2	2.81	2.20E-05	0.0020
UPK1A	2.81	7.81E-03	0.0244
RNVU1-15	2.81	6.18E-03	0.0210
BTBD3	2.81	5.49E-05	0.0027
WSB2	2.81	2.41E-04	0.0042
DDX11-AS1	2.81	1.00E-02	0.0286
RASSF4	2.80	1.86E-03	0.0105
RFC2	2.80	1.16E-04	0.0034
XRCC3	2.80	1.46E-04	0.0036
PDF	2.80	1.82E-03	0.0104
AIF1	2.80	4.83E-03	0.0181
TUBG1	2.80	5.40E-05	0.0027
EBNA1BP2	2.80	1.65E-04	0.0037
DEPP1	2.79	1.34E-02	0.0347
DIXDC1	2.79	2.12E-05	0.0020
CHST3	2.79	9.33E-03	0.0273
DTD1	2.79	2.27E-05	0.0020
FANCB	2.79	3.07E-04	0.0046
AC004585.1	2.79	1.44E-03	0.0092
ENST000003535480	2.78	3.50E-04	0.0048
ENST000003616110	2.78	2.39E-04	0.0042
FIGNL1	2.78	6.23E-05	0.0028
SPIRE1	2.78	2.10E-03	0.0113
AC073611.1	2.78	6.72E-03	0.0222
SNHG3	2.78	7.74E-05	0.0030
FAM43A	2.77	9.36E-04	0.0075
TNK1	2.77	7.23E-03	0.0232
HIST3H2BB	2.77	5.12E-04	0.0056
PRSS23	2.77	2.43E-03	0.0123
HNRNPAB	2.76	5.52E-05	0.0027
FOXO3	2.76	1.43E-02	0.0364
ENST000002247840	2.76	1.22E-02	0.0326
RYR1	2.75	2.15E-04	0.0041
SLC7A11	2.75	2.70E-03	0.0130
MAP3K20	2.75	2.52E-03	0.0125
NUDT1	2.75	6.95E-05	0.0029
TICRR	2.75	3.69E-03	0.0154
DRP2	2.75	4.62E-04	0.0054
RAI14	2.75	3.80E-04	0.0050
LIMA1	2.74	1.07E-04	0.0033

AC245014.3	2.74	1.23E-04	0.0034
VPS9D1-AS1	2.74	4.04E-03	0.0162
TCAF2P1	2.74	3.68E-04	0.0049
GAB1	2.74	3.63E-03	0.0152
ENST000002957462	2.74	1.36E-04	0.0035
PUS7	2.74	2.48E-05	0.0021
CSRNP1	2.74	3.40E-04	0.0048
FBXO5	2.74	6.25E-05	0.0028
AC009309.1	2.73	5.72E-03	0.0201
ENST000002639850	2.73	1.43E-04	0.0036
BYSL	2.73	3.42E-03	0.0147
HIST1H3I	2.73	2.54E-04	0.0043
CCDC144CP	2.73	1.64E-02	0.0400
ENST000003386350	2.73	3.19E-04	0.0047
CMTM4	2.73	1.18E-03	0.0083
ASB2	2.73	3.90E-03	0.0159
ALDH1L2	2.73	1.16E-02	0.0316
AC073529.1	2.73	3.09E-04	0.0046
BOLA3	2.72	1.08E-04	0.0033
ARHGEF12	2.72	2.91E-04	0.0045
HIST1H1E	2.72	8.71E-05	0.0031
PPP4R4	2.72	3.12E-03	0.0140
CHTF18	2.71	4.12E-04	0.0051
CENPP	2.71	8.41E-03	0.0256
SQOR	2.71	2.36E-05	0.0021
CDCA7L	2.71	2.69E-04	0.0044
PPP1R14B	2.71	2.08E-04	0.0040
LYSMD1	2.71	1.55E-04	0.0036
AGFG1	2.71	2.32E-05	0.0021
C9orf40	2.71	4.65E-04	0.0054
MCM8	2.71	3.61E-04	0.0049
FAM216A	2.71	1.35E-04	0.0035
RNU5D-1	2.70	2.63E-04	0.0044
SNORD91B	2.70	3.53E-03	0.0150
ATRNL1	2.70	3.77E-03	0.0156
ALYREF	2.70	5.44E-05	0.0027
SYTL3	2.69	7.18E-05	0.0029
APBB2	2.69	1.41E-03	0.0091
CST7	2.69	5.70E-04	0.0059
CD82	2.69	1.95E-04	0.0040
BZW1P2	2.69	1.16E-04	0.0034
FAH	2.69	8.14E-04	0.0070
PKM	2.68	2.70E-05	0.0021
LRFN4	2.68	6.33E-04	0.0062
PTP4A3	2.68	2.42E-03	0.0122
CU634019.6	2.68	1.35E-02	0.0349
ENST000003253240	2.68	5.80E-05	0.0028
ENST000002710024	2.68	1.45E-04	0.0036
DBNDD2	2.68	1.61E-03	0.0098
NRIP3	2.67	5.86E-04	0.0060

CD276	2.67	1.69E-03	0.0101
SEPT8	2.67	2.22E-02	0.0497
PFKM	2.67	2.22E-05	0.0020
GALE	2.67	3.19E-04	0.0047
LMNB1	2.67	7.56E-05	0.0030
PPIF	2.67	1.12E-04	0.0033
GNAI1	2.66	8.52E-03	0.0258
RDM1	2.66	2.19E-02	0.0493
DHCR7	2.66	1.25E-04	0.0035
NUDT12	2.66	7.69E-03	0.0241
CCDC74A	2.66	6.17E-03	0.0210
BCL2L2	2.66	5.19E-04	0.0057
RFC4	2.66	2.21E-05	0.0020
IFRD2	2.65	1.59E-04	0.0037
GIHCG	2.65	6.91E-05	0.0029
ENST000003548580	2.65	1.62E-04	0.0037
SLC35G2	2.65	7.94E-04	0.0069
HK2	2.65	2.27E-04	0.0042
MYO1D	2.65	7.68E-03	0.0241
BCKDK	2.64	8.11E-04	0.0070
RNASEH2A	2.64	6.09E-05	0.0028
HMGB1P6	2.64	4.31E-05	0.0024
SPINT1	2.64	1.77E-03	0.0102
HAUS6P3	2.64	8.67E-03	0.0261
TTC30A	2.64	2.55E-03	0.0126
NR4A2	2.63	1.62E-02	0.0396
AL390719.1	2.63	6.09E-03	0.0209
MCM6	2.63	2.86E-05	0.0022
PPAN	2.63	1.09E-04	0.0033
FANCG	2.63	7.28E-05	0.0029
SORD	2.63	7.00E-04	0.0065
HIST1H1C	2.63	3.31E-05	0.0023
SNORA71A	2.63	8.28E-03	0.0253
CHAC2	2.63	4.76E-05	0.0026
ENST000002546630	2.62	2.67E-03	0.0129
FAM92A	2.62	1.01E-03	0.0077
AJM1	2.62	5.35E-04	0.0058
ARHGAP11A	2.62	2.65E-05	0.0021
CHRNA5	2.62	1.18E-03	0.0083
LINC00888	2.62	2.38E-04	0.0042
NFKBIA	2.62	2.43E-05	0.0021
KRT18	2.62	1.55E-04	0.0036
ADORA2B	2.62	1.82E-04	0.0039
PDXP	2.61	3.71E-04	0.0049
ENST000003574290	2.61	6.33E-04	0.0062
CD27-AS1	2.61	6.20E-04	0.0062
LMNB2	2.61	2.76E-05	0.0021
ENST000003131170	2.61	5.45E-03	0.0195
SAP30	2.61	3.07E-05	0.0022
ST7-OT4	2.61	3.14E-04	0.0046

SRGN	2.60	1.96E-04	0.0040
C15orf38-AP3S2	2.60	1.67E-02	0.0405
C3orf14	2.60	6.51E-04	0.0063
TFDP1	2.60	2.76E-05	0.0021
ENST000003162181	2.60	1.41E-03	0.0091
SNRPE	2.60	4.31E-05	0.0024
ENST000002656020	2.60	3.01E-04	0.0046
BZW1	2.60	4.13E-05	0.0024
ZC3H12D	2.60	1.12E-03	0.0081
AC125611.2	2.60	3.84E-03	0.0158
LMNB1-DT	2.60	2.02E-03	0.0110
IFIT1P1	2.60	6.32E-03	0.0214
PSMD1	2.59	2.03E-02	0.0467
POLA1	2.59	3.61E-05	0.0023
RACGAP1	2.59	3.91E-05	0.0024
INCENP	2.59	2.69E-04	0.0044
ENST000003339420	2.58	9.38E-03	0.0274
MAPK12	2.58	9.57E-04	0.0076
ENST00000629913	2.58	9.30E-03	0.0272
PLAGL1	2.58	8.01E-04	0.0069
ENST000002162812	2.58	1.38E-04	0.0035
MAP7D2	2.57	1.99E-02	0.0460
AC012645.1	2.57	2.70E-03	0.0130
SNX33	2.57	1.76E-03	0.0102
RPL26L1	2.57	8.03E-05	0.0030
PHGDH	2.57	5.26E-03	0.0190
PLK1	2.57	4.23E-04	0.0052
GFOD1	2.57	3.15E-03	0.0141
HMGA1	2.57	7.55E-04	0.0067
GGCT	2.56	3.22E-05	0.0023
TST	2.56	1.12E-02	0.0308
ACOT7	2.56	2.57E-04	0.0043
POLD2	2.56	4.22E-04	0.0052
ENST000003340820	2.56	1.21E-04	0.0034
SGMS2	2.56	4.97E-03	0.0184
SMC2	2.56	4.48E-05	0.0025
BEST4	2.56	1.15E-02	0.0313
CIP2A	2.56	2.56E-05	0.0021
IRAK3	2.55	2.60E-04	0.0044
TCP1	2.55	5.90E-05	0.0028
HSPA2	2.55	1.04E-03	0.0079
RNU4ATAC	2.55	8.50E-03	0.0257
CDK2	2.55	3.49E-05	0.0023
MRPL39	2.55	1.36E-04	0.0035
SLC25A4	2.55	2.34E-03	0.0120
ST3GAL4	2.55	1.53E-04	0.0036
ENST000003048341	2.55	5.23E-03	0.0190
CYCS	2.55	9.80E-05	0.0031
MDH1B	2.55	1.32E-02	0.0343
STK24-AS1	2.54	5.87E-03	0.0204

TXN	2.54	1.80E-03	0.0103
ENST000002579040	2.54	1.26E-04	0.0035
NINJ1	2.54	1.33E-04	0.0035
IL1R1	2.53	4.25E-03	0.0167
ENST000003243662	2.53	3.22E-05	0.0023
CDCA7	2.53	1.07E-04	0.0033
RP9P	2.53	2.60E-03	0.0127
ZNF334	2.53	3.55E-04	0.0048
PTGIR	2.53	1.74E-03	0.0102
NDUFV2P1	2.53	1.31E-02	0.0342
SCML2	2.52	2.50E-03	0.0124
ZBED8	2.52	3.51E-04	0.0048
C11orf24	2.52	5.90E-03	0.0204
NDC1	2.52	6.72E-05	0.0029
MCM5	2.52	3.43E-05	0.0023
GPT2	2.52	2.42E-04	0.0042
PERP	2.52	5.90E-04	0.0060
HIF1A-AS2	2.52	1.06E-02	0.0297
AK6	2.52	4.53E-05	0.0025
C1QBP	2.51	1.11E-04	0.0033
DGUOK-AS1	2.51	9.60E-03	0.0278
TMEM106C	2.51	1.11E-03	0.0081
IL18R1	2.51	1.54E-02	0.0382
ERRFI1	2.51	2.06E-02	0.0472
RF00592.2	2.50	9.82E-04	0.0077
RAB13	2.50	2.36E-03	0.0121
DARS2	2.50	5.84E-05	0.0028
ZNF492	2.50	1.08E-02	0.0302
SYT11	2.50	1.57E-04	0.0037
GNB4	2.49	1.91E-04	0.0039
ENST000002503792	2.49	7.24E-03	0.0232
PSMA2	2.49	2.82E-05	0.0021
ENST000002166052	2.49	2.31E-04	0.0042
ENST000002831794	2.49	4.09E-04	0.0051
ENST000003069320	2.49	2.44E-03	0.0123
AC073263.2	2.49	7.10E-04	0.0065
UBE2E2	2.48	2.25E-03	0.0118
WEE1	2.48	5.21E-04	0.0057
LINC00239	2.48	1.17E-02	0.0318
SNRNP25	2.48	1.11E-03	0.0081
TLCD1	2.48	5.21E-03	0.0190
CENPJ	2.48	3.76E-05	0.0023
PRG4	2.48	3.03E-03	0.0138
NDUFA6	2.48	6.47E-04	0.0063
TOMM40	2.47	1.57E-04	0.0037
MASTL	2.47	3.69E-05	0.0023
RHOC	2.47	4.27E-04	0.0052
CD109	2.47	2.83E-04	0.0045
NCKAP1	2.47	2.16E-03	0.0114
ZNF788P	2.47	4.23E-05	0.0024

MIS18A	2.47	8.56E-05	0.0030
TRAP1	2.47	7.82E-05	0.0030
NOP16	2.47	2.01E-04	0.0040
FSTL3	2.47	3.69E-04	0.0049
ENST000003212330	2.46	1.44E-04	0.0036
ENST000002716361	2.46	9.98E-03	0.0285
TIMM10	2.46	1.14E-04	0.0033
KLHL23	2.46	5.55E-04	0.0059
KDM4A-AS1	2.45	2.82E-03	0.0133
ENST000003183880	2.45	1.50E-04	0.0036
PMAIP1	2.45	8.96E-04	0.0073
RN7SL3	2.45	2.37E-04	0.0042
AC027031.2	2.45	1.11E-02	0.0307
HES2	2.45	7.52E-03	0.0238
AGAP1	2.45	4.04E-03	0.0162
FAM96AP2	2.45	6.71E-03	0.0222
TMEM44	2.45	8.57E-04	0.0072
PROB1	2.45	2.18E-03	0.0115
AC084117.1	2.45	1.82E-03	0.0104
METTL1	2.44	2.59E-04	0.0044
MTFP1	2.44	1.94E-04	0.0039
E2F1	2.44	3.13E-04	0.0046
TMEM231	2.44	1.37E-02	0.0353
GPN3	2.43	2.85E-04	0.0045
ZNF711	2.43	1.34E-02	0.0347
ICAM1	2.43	1.42E-04	0.0036
B9D1	2.43	1.69E-03	0.0100
ENST000003612190	2.43	7.70E-05	0.0030
ENST000002186521	2.43	1.67E-02	0.0406
DCUN1D5	2.43	1.94E-04	0.0039
GNG8	2.43	1.80E-02	0.0428
PFKP	2.43	3.50E-05	0.0023
MRPL12	2.43	1.73E-03	0.0102
RBBP8	2.43	1.56E-04	0.0036
UPRT	2.43	1.46E-04	0.0036
FAM210A	2.43	3.53E-05	0.0023
LSM4	2.42	3.25E-03	0.0143
ENST000003334791	2.42	6.89E-05	0.0029
GALK1	2.42	3.74E-04	0.0049
RCC1	2.42	1.65E-04	0.0037
SNRPD1	2.42	8.27E-05	0.0030
SIAH2	2.42	4.44E-05	0.0025
ENST000003559680	2.42	1.51E-04	0.0036
CHAF1B	2.42	8.42E-05	0.0030
AL138724.1	2.42	3.45E-03	0.0148
ENST000003381930	2.41	3.54E-05	0.0023
CCDC50	2.41	9.10E-03	0.0268
POFUT1	2.41	9.01E-05	0.0031
P4HA1	2.41	3.55E-05	0.0023
PCBD1	2.41	1.29E-04	0.0035

ENST000002808920	2.41	4.29E-04	0.0052
GATM	2.41	5.52E-03	0.0196
ADCY3	2.41	2.42E-03	0.0122
ENST000002492701	2.41	5.96E-04	0.0060
MRPL11	2.41	2.80E-04	0.0045
ENST000003146660	2.40	3.68E-04	0.0049
FDPS	2.40	5.25E-05	0.0027
STIP1	2.40	1.97E-04	0.0040
AL078644.1	2.40	1.22E-02	0.0326
SNHG15	2.40	1.36E-04	0.0035
SHMT1	2.40	7.67E-04	0.0068
NUDT15	2.40	1.41E-04	0.0036
CDCA4	2.40	5.99E-05	0.0028
HIST1H4H	2.40	3.82E-04	0.0050
PAQR7	2.40	1.81E-03	0.0104
CENPQ	2.39	1.92E-04	0.0039
TIAM2	2.39	1.21E-04	0.0034
SNRPB	2.39	7.11E-05	0.0029
COPG2	2.39	3.14E-04	0.0046
RRM1	2.39	4.92E-05	0.0026
ATAD2	2.39	4.53E-05	0.0025
TNFRSF12A	2.39	2.94E-04	0.0045
SFXN4	2.39	2.63E-04	0.0044
C1orf74	2.39	1.90E-04	0.0039
NUDT11	2.39	6.79E-03	0.0224
SMPDL3B	2.38	1.73E-03	0.0102
CALM3	2.38	6.78E-05	0.0029
SNORD12C	2.38	2.05E-02	0.0470
PKN3	2.38	2.50E-03	0.0124
ENST000002641590	2.38	9.27E-03	0.0271
SLC31A1	2.38	5.50E-05	0.0027
AK2	2.38	6.36E-05	0.0028
PRDX3	2.37	6.09E-05	0.0028
ENST000003290922	2.37	5.22E-03	0.0190
PARPBP	2.37	1.87E-04	0.0039
MICAL2	2.37	7.08E-04	0.0065
ENST000003546941	2.37	6.08E-05	0.0028
CPS1	2.37	5.00E-03	0.0185
PHB	2.37	1.45E-04	0.0036
PRDX4	2.37	5.24E-04	0.0057
POMP	2.37	1.90E-04	0.0039
AC004825.2	2.37	6.28E-03	0.0213
NPM1P9	2.37	2.83E-03	0.0133
SUV39H1	2.36	8.53E-05	0.0030
TTC23	2.36	4.38E-03	0.0171
ENST000003213581	2.36	8.15E-05	0.0030
IMPDH2	2.36	4.17E-05	0.0024
MRPL17	2.36	3.96E-05	0.0024
CHCHD2	2.36	4.43E-05	0.0025
AC130324.3	2.36	4.50E-04	0.0054

SNORD83A	2.36	4.13E-03	0.0165
SMCO4	2.36	8.28E-04	0.0071
AC087632.1	2.36	2.05E-04	0.0040
ENST000002982880	2.36	3.27E-03	0.0143
SNORA71D	2.35	4.74E-03	0.0179
NR2F6	2.35	1.23E-02	0.0328
XRCC2	2.35	6.14E-05	0.0028
ENST000002810811	2.35	7.78E-05	0.0030
MERTK	2.35	8.86E-03	0.0264
PDLIM7	2.35	8.49E-04	0.0071
GPSM2	2.35	8.75E-05	0.0031
RDH10	2.35	1.44E-04	0.0036
WDR76	2.35	4.37E-05	0.0024
POLD1	2.35	5.85E-04	0.0060
MT1L	2.35	7.32E-03	0.0234
SNORA65	2.35	9.99E-03	0.0286
MESP1	2.35	7.33E-03	0.0234
DUSP3	2.34	2.99E-04	0.0046
MORF4L2	2.34	9.14E-05	0.0031
SSRP1	2.34	6.74E-05	0.0029
RNU5F-1	2.34	2.38E-03	0.0121
ENST000003251100	2.34	1.51E-04	0.0036
RUVBL2	2.33	1.31E-04	0.0035
ENST000002331142	2.33	7.85E-05	0.0030
DUT	2.33	6.10E-05	0.0028
SNHG4	2.33	3.37E-04	0.0048
ENST000002974400	2.33	1.67E-04	0.0038
GLA	2.33	1.13E-04	0.0033
FKBP4	2.33	3.86E-04	0.0050
MBOAT2	2.33	2.81E-04	0.0045
AC243587.1	2.33	1.08E-03	0.0080
HMGN2P5	2.33	1.08E-03	0.0080
ITPRIPL1	2.33	6.90E-05	0.0029
FRRS1	2.33	5.46E-03	0.0195
WDR54	2.33	7.02E-05	0.0029
ADAM9	2.33	3.22E-04	0.0047
ENST000002045170	2.32	4.44E-04	0.0053
PTPN7	2.32	7.57E-05	0.0030
LAMA2	2.32	2.01E-02	0.0464
ENST000003016862	2.32	2.38E-03	0.0121
PALD1	2.32	3.79E-03	0.0156
BCL2L12	2.32	1.76E-03	0.0102
NINJ2	2.31	1.90E-02	0.0445
ALDH4A1	2.31	1.65E-03	0.0099
MSRB1	2.30	1.16E-04	0.0034
ENST000003237440	2.30	1.68E-03	0.0100
ENST000002993000	2.30	1.13E-04	0.0033
ZNF730	2.30	1.82E-03	0.0104
AL031733.2	2.30	4.85E-03	0.0181
SNX10	2.30	1.06E-03	0.0079

FAM89A	2.30	3.76E-04	0.0049
STX11	2.30	4.24E-04	0.0052
POLA2	2.30	5.66E-05	0.0028
RAPH1	2.29	1.47E-04	0.0036
ENST000003055600	2.29	1.31E-02	0.0342
ENST000002958091	2.29	1.96E-03	0.0108
SLC38A5	2.29	1.07E-03	0.0080
FHL3	2.29	9.93E-04	0.0077
ENST000003044001	2.29	6.36E-03	0.0214
PSMA6	2.29	5.79E-05	0.0028
ENO2	2.29	1.19E-04	0.0034
ENST000003738330	2.28	3.14E-04	0.0046
ORC6	2.28	1.06E-04	0.0033
SNX9	2.28	3.48E-04	0.0048
ENST000002904290	2.28	1.51E-03	0.0094
AC098818.2	2.28	1.78E-02	0.0423
MATK	2.28	2.39E-04	0.0042
GLO1	2.28	4.58E-04	0.0054
MAD2L2	2.28	6.55E-05	0.0029
ZC3HAV1L	2.28	2.06E-03	0.0111
HMGCS1	2.27	1.90E-04	0.0039
WDFY3	2.27	3.59E-03	0.0151
ENST000002967010	2.27	2.90E-03	0.0135
TOM1L1	2.27	7.10E-03	0.0230
PPIL1	2.27	3.32E-04	0.0047
FOSL2	2.27	1.27E-02	0.0336
ENST000002642112	2.27	3.11E-03	0.0140
GVQW2	2.27	4.30E-03	0.0169
MZT1	2.26	1.36E-04	0.0035
IFI27L1	2.26	5.70E-04	0.0059
CD160	2.26	3.41E-04	0.0048
TBC1D16	2.26	3.41E-03	0.0147
ENST000002850391	2.26	2.06E-02	0.0472
CNTNAP1	2.25	1.55E-03	0.0096
NDUFAB1	2.25	1.92E-04	0.0039
PTMA	2.25	3.39E-04	0.0048
SASS6	2.25	1.06E-04	0.0033
HSPA9	2.25	8.72E-05	0.0031
ENST000002346260	2.25	7.59E-05	0.0030
SPA17	2.25	8.92E-04	0.0073
TMPO	2.24	4.54E-05	0.0025
ENST000003213941	2.24	6.37E-04	0.0062
ENST000002916340	2.24	1.65E-04	0.0037
CCT5	2.24	1.06E-04	0.0033
KIF20B	2.24	5.03E-05	0.0026
SNRPF	2.24	6.21E-05	0.0028
SCARNA22	2.24	2.64E-04	0.0044
XYLB	2.24	7.25E-04	0.0066
C8orf88	2.24	3.25E-03	0.0143
SNORA80B	2.23	3.85E-03	0.0158

AC018413.1	2.23	1.40E-02	0.0358
ENST000003243403	2.23	8.64E-05	0.0030
AC136632.1	2.23	1.40E-03	0.0091
SFXN1	2.23	3.04E-04	0.0046
PTPRK	2.22	5.70E-04	0.0059
ENST000002511011	2.22	1.71E-03	0.0101
ENTHD1	2.22	1.96E-02	0.0455
ANAPC11	2.22	9.51E-05	0.0031
SAV1	2.22	2.43E-04	0.0042
GSTCD	2.22	5.98E-05	0.0028
SNRPG	2.22	1.22E-04	0.0034
NDST1	2.22	1.54E-02	0.0383
C10orf25	2.22	2.96E-03	0.0136
ENST000003413691	2.22	2.80E-03	0.0133
TEDC1	2.22	1.74E-04	0.0038
ENST000003319440	2.22	3.68E-04	0.0049
TONSL	2.21	9.50E-04	0.0076
STOML2	2.21	1.84E-04	0.0039
EHD4	2.21	2.16E-04	0.0041
ENST000002595230	2.21	4.38E-03	0.0171
C9orf64	2.21	6.47E-05	0.0028
FZD3	2.21	4.89E-04	0.0055
CLPB	2.21	2.68E-04	0.0044
MXD3	2.20	1.64E-03	0.0099
RF00003.30	2.20	6.17E-03	0.0210
ENST000002455390	2.20	1.98E-04	0.0040
SKA2	2.20	7.95E-05	0.0030
AC005076.1	2.20	8.90E-04	0.0073
KBTBD11	2.20	1.06E-02	0.0296
ENST000003089821	2.20	1.02E-04	0.0032
AL161891.1	2.20	1.20E-04	0.0034
MAP3K8	2.19	3.52E-04	0.0048
GPR19	2.19	1.70E-03	0.0101
LINC01678	2.19	7.36E-03	0.0234
HMGN5	2.19	8.68E-03	0.0261
TCEAL9	2.19	1.22E-02	0.0327
ANKRD13B	2.19	1.69E-03	0.0101
THG1L	2.19	2.36E-04	0.0042
BRI3BP	2.19	2.70E-04	0.0044
SNORD19	2.19	7.34E-03	0.0234
KIAA0895L	2.19	8.37E-04	0.0071
C18orf54	2.19	2.11E-03	0.0113
SLFN11	2.19	9.08E-05	0.0031
CCT7	2.19	9.38E-05	0.0031
YWHAG	2.18	8.92E-05	0.0031
ENST000003305031	2.18	1.41E-04	0.0036
MEA1	2.18	5.91E-05	0.0028
HIST1H2APS5	2.18	9.25E-04	0.0075
ENST000003227760	2.18	2.12E-04	0.0041
PRELID1	2.18	1.66E-04	0.0037

ENST000002781000	2.18	2.09E-04	0.0040
C21orf59-TCP10L	2.18	3.02E-03	0.0138
SOGA3	2.18	8.60E-03	0.0259
TMED8	2.18	1.33E-04	0.0035
SNRPC	2.18	1.37E-04	0.0035
LRRC42	2.17	8.40E-04	0.0071
FAM162A	2.17	1.27E-04	0.0035
ENST000003162730	2.17	1.13E-03	0.0081
NAT14	2.17	1.34E-03	0.0088
PAQR4	2.17	1.46E-04	0.0036
CHPF	2.16	1.09E-03	0.0080
GOT2	2.15	5.36E-05	0.0027
DNAJC18	2.15	2.07E-04	0.0040
HDGF	2.15	1.74E-04	0.0038
KIAA1614-AS1	2.15	6.08E-03	0.0208
CACYBP	2.15	8.08E-05	0.0030
UTP11	2.15	1.96E-04	0.0040
ASIC1	2.15	1.48E-03	0.0093
NUP37	2.15	1.45E-04	0.0036
TIMM21	2.15	2.91E-04	0.0045
KNTC1	2.15	1.10E-04	0.0033
MSH5	2.15	3.82E-04	0.0050
RAB20	2.15	2.74E-03	0.0131
UBE2E3	2.14	3.86E-04	0.0050
AC012254.2	2.14	2.45E-03	0.0123
ENST000002961220	2.14	1.83E-04	0.0039
BOP1	2.14	1.78E-03	0.0103
MAN1A1	2.14	7.36E-05	0.0029
NOCT	2.14	1.78E-04	0.0039
PLS1	2.14	9.24E-04	0.0075
ETF1	2.14	8.61E-05	0.0030
DBI	2.14	7.00E-05	0.0029
ENKD1	2.13	1.16E-03	0.0083
TCEAL4	2.13	1.70E-03	0.0101
B4GALT5	2.13	2.09E-03	0.0112
WDR18	2.13	3.73E-04	0.0049
EEF1E1	2.13	4.15E-04	0.0051
ENST000003238332	2.13	2.00E-04	0.0040
TNF	2.13	7.96E-04	0.0069
ADAP1	2.13	2.16E-03	0.0114
ARHGAP19	2.13	6.03E-05	0.0028
CTNNA1	2.13	2.62E-03	0.0128
SLIRP	2.13	6.30E-05	0.0028
TRIQK	2.12	3.05E-03	0.0138
TIMM8A	2.12	4.94E-04	0.0056
TSR1	2.12	2.27E-04	0.0041
RHOBTB3	2.12	1.61E-02	0.0395
FUOM	2.12	1.29E-03	0.0087
AC007253.1	2.12	8.68E-03	0.0261
SLC25A19	2.12	4.04E-04	0.0051

PSAT1	2.12	1.15E-02	0.0314
RAVER2	2.12	5.85E-03	0.0204
GPI	2.11	1.04E-04	0.0033
MYB	2.11	4.68E-03	0.0178
EXOSC4	2.11	2.82E-04	0.0045
MPZL1	2.11	8.95E-05	0.0031
SLC37A4	2.11	3.80E-04	0.0050
ENST000002531071	2.11	1.99E-02	0.0460
TOMM5	2.11	3.45E-04	0.0048
SLC39A14	2.11	1.34E-03	0.0088
BRIP1	2.11	1.93E-04	0.0039
SET	2.11	1.69E-04	0.0038
TXNDC5	2.11	5.08E-04	0.0056
ARMCX1	2.11	2.06E-02	0.0472
AC051619.7	2.11	8.33E-03	0.0254
C20orf27	2.11	1.67E-02	0.0405
ENST000002996130	2.11	1.39E-04	0.0036
HIST4H4	2.11	1.23E-04	0.0034
ENST000003328580	2.11	1.63E-03	0.0099
RRAGD	2.11	3.03E-04	0.0046
ENST000003192482	2.10	2.00E-04	0.0040
COX5A	2.10	2.96E-04	0.0046
ENST000002855180	2.10	9.38E-05	0.0031
AHI1	2.10	4.62E-03	0.0176
CU638689.5	2.10	4.01E-03	0.0162
ATAD3A	2.10	5.43E-04	0.0058
GEN1	2.10	6.65E-05	0.0029
ATP2A1-AS1	2.10	1.21E-02	0.0326
EEF1AKMT4	2.10	1.83E-03	0.0104
PSMD14	2.10	1.50E-04	0.0036
TRIP6	2.10	3.69E-04	0.0049
FBXW9	2.10	1.19E-03	0.0083
SLC17A9	2.10	7.51E-04	0.0067
ENST000002718360	2.10	2.05E-02	0.0470
ACAT2	2.10	9.62E-05	0.0031
PA2G4	2.10	1.66E-04	0.0037
SEC61G	2.10	9.57E-05	0.0031
ENST000003167881	2.09	8.56E-05	0.0030
MTHFD1L	2.09	9.09E-04	0.0074
ARL3	2.09	3.99E-04	0.0051
PPIAP45	2.09	4.60E-03	0.0176
ENST000002965950	2.09	1.78E-04	0.0039
ENST000003889950	2.09	2.25E-04	0.0041
NCL	2.09	8.63E-05	0.0030
HSPG2	2.09	3.14E-03	0.0140
AC099850.1	2.09	1.43E-03	0.0092
ERAL1	2.09	2.02E-04	0.0040
NUP58	2.09	1.11E-04	0.0033
MRPL13	2.08	1.33E-04	0.0035
CYC1	2.08	2.05E-04	0.0040

MRPL36	2.08	1.81E-03	0.0104
TXNDC17	2.08	2.21E-04	0.0041
INSIG1	2.08	8.86E-05	0.0031
YWHAQ	2.08	2.08E-04	0.0040
CSTF2	2.08	2.89E-04	0.0045
SH2D2A	2.08	7.12E-04	0.0065
FAM207A	2.08	5.69E-03	0.0200
MIR4435-2HG	2.08	3.27E-04	0.0047
TNFRSF21	2.08	1.22E-02	0.0326
UQCRQ	2.08	8.98E-05	0.0031
NFE2L3	2.08	1.35E-04	0.0035
UQCRC1	2.07	1.05E-04	0.0033
KDELC1	2.07	2.14E-02	0.0485
NLE1	2.07	9.15E-05	0.0031
NCAPH2	2.07	9.81E-05	0.0031
PAK1IP1	2.07	8.67E-04	0.0072
BSPRY	2.07	5.11E-03	0.0187
ENST000002587871	2.07	3.40E-04	0.0048
SLC25A5	2.07	1.28E-04	0.0035
MEST	2.07	1.85E-02	0.0436
WRAP53	2.07	2.46E-04	0.0042
AL109918.1	2.07	6.45E-04	0.0062
PHF6	2.07	4.30E-04	0.0052
SGPP2	2.07	4.46E-04	0.0053
TUBAP2	2.07	6.67E-03	0.0221
U62317.2	2.06	1.85E-03	0.0105
CELSR3	2.06	4.00E-03	0.0161
TRIO	2.06	2.09E-03	0.0112
ENST000003601280	2.06	3.24E-04	0.0047
ENST000003010680	2.06	4.10E-03	0.0164
FANCC	2.06	1.99E-04	0.0040
HSPBP1	2.06	1.61E-03	0.0098
TNS3	2.06	7.13E-03	0.0230
CTPS1	2.06	2.82E-04	0.0045
ABCG1	2.06	1.28E-02	0.0337
AGK	2.06	1.26E-04	0.0035
GNPNAT1	2.06	6.15E-04	0.0061
HIST1H1D	2.06	9.81E-05	0.0031
CHN1	2.06	7.59E-03	0.0239
ENST000002913580	2.05	1.91E-03	0.0107
ILF2	2.05	1.09E-04	0.0033
NRAV	2.05	1.02E-02	0.0290
PLOD1	2.05	4.53E-04	0.0054
DKC1	2.05	1.77E-04	0.0039
RRP12	2.05	3.71E-04	0.0049
PARK7	2.05	7.39E-05	0.0029
ALDH18A1	2.04	7.45E-05	0.0029
NCAPD2	2.04	2.26E-04	0.0041
LAPTM4B	2.04	3.09E-04	0.0046
ZNF165	2.04	3.54E-03	0.0150

PEX26	2.04	1.39E-04	0.0036
ENST000003588960	2.04	2.34E-03	0.0120
COX17	2.04	7.16E-05	0.0029
CPNE2	2.04	6.47E-04	0.0063
PSMC3	2.04	9.48E-05	0.0031
PPIA	2.04	8.48E-05	0.0030
DPP3	2.04	2.24E-04	0.0041
EFCAB11	2.04	3.07E-03	0.0139
ENST000003206761	2.03	7.13E-05	0.0029
KCTD12	2.03	1.07E-02	0.0299
ENST000002373051	2.03	1.71E-02	0.0412
ZFP92	2.03	4.00E-03	0.0161
PRELID2	2.03	2.35E-03	0.0121
JUNB	2.03	5.87E-04	0.0060
ENST000002615070	2.02	5.05E-04	0.0056
NLN	2.02	2.23E-03	0.0117
LAP3	2.02	2.73E-04	0.0044
PTPN6	2.02	6.60E-04	0.0063
CREM	2.02	2.49E-03	0.0124
PTPN11	2.02	6.91E-05	0.0029
ENST000003047861	2.02	1.25E-04	0.0035
ERH	2.02	1.41E-04	0.0036
CTH	2.02	8.28E-03	0.0253
GNL3	2.02	4.38E-04	0.0053
ENST000002702251	2.02	3.98E-04	0.0051
MPZL3	2.02	1.34E-04	0.0035
LRRC59	2.02	1.99E-04	0.0040
PRMT5	2.02	1.15E-03	0.0082
TUBB2A	2.02	4.42E-03	0.0172
RCN1	2.01	8.83E-04	0.0073
ALCAM	2.01	1.35E-03	0.0089
RNU11	2.01	1.10E-02	0.0304
C21orf58	2.01	3.46E-03	0.0148
COQ2	2.01	1.32E-04	0.0035
ALDOC	2.01	5.92E-04	0.0060
ENST000002938312	2.01	1.79E-04	0.0039
POLR2H	2.01	1.64E-04	0.0037
MRC2	2.01	8.54E-03	0.0258
RRP1	2.01	2.24E-04	0.0041
ENST000002253082	2.01	1.47E-03	0.0093
GALNT2	2.01	3.30E-04	0.0047
ENST000003113370	2.01	1.41E-03	0.0091
ACAT1	2.01	9.52E-05	0.0031
NOLC1	2.01	1.10E-03	0.0080
MAP3K21	2.01	4.30E-04	0.0052
SNHG16	2.01	7.50E-04	0.0067
SAAL1	2.01	3.60E-04	0.0049
LSM5	2.00	1.86E-04	0.0039
FUT11	2.00	3.01E-04	0.0046
ENST000002674151	2.00	4.73E-04	0.0055

AC090825.1	2.00	3.39E-03	0.0146
GSTT1	2.00	7.70E-03	0.0241
Z83847.1	2.00	1.16E-02	0.0316
CCT4	2.00	7.98E-05	0.0030
PLXNB2	2.00	9.21E-03	0.0270
VPS25	2.00	1.10E-04	0.0033
CHCHD4	2.00	1.99E-04	0.0040
ENST000002422572	2.00	1.52E-03	0.0095
HECTD2	1.99	5.67E-04	0.0059
UBE2M	1.99	4.18E-04	0.0052
MFSD10	1.99	3.04E-04	0.0046
EPB41L2	1.99	3.20E-03	0.0142
TIFA	1.99	5.54E-04	0.0059
ENST000003163860	1.99	4.56E-04	0.0054
RNVU1-1	1.99	8.87E-03	0.0264
ENST000003204810	1.99	4.81E-03	0.0180
AFMID	1.99	4.22E-04	0.0052
ENST000003239970	1.99	8.42E-03	0.0256
PRPS2	1.99	1.68E-04	0.0038
NUP155	1.99	1.54E-04	0.0036
HLF	1.98	9.51E-04	0.0076
NASP	1.98	8.41E-05	0.0030
SLC39A1	1.98	1.99E-04	0.0040
ENST000002428270	1.98	9.58E-04	0.0076
AIMP2	1.98	2.43E-03	0.0123
DCLRE1B	1.98	7.39E-04	0.0066
SNORD99	1.98	1.17E-02	0.0317
ENST000003485020	1.98	9.57E-05	0.0031
ATP5MD	1.98	1.33E-04	0.0035
RPF2	1.98	2.13E-03	0.0114
SERBP1	1.98	1.61E-04	0.0037
MAK16	1.97	6.58E-04	0.0063
ENST000003255992	1.97	3.07E-04	0.0046
HIRIP3	1.97	6.46E-04	0.0063
ZSCAN20	1.97	8.32E-04	0.0071
CCT8	1.97	3.78E-03	0.0156
AC016026.1	1.97	5.45E-03	0.0195
ENST000003514500	1.97	1.50E-04	0.0036
NSD2	1.97	9.49E-05	0.0031
AL365181.3	1.97	5.86E-03	0.0204
NDUFA7	1.97	3.31E-04	0.0047
PDE4A	1.97	5.31E-03	0.0191
ENST000002679502	1.97	1.54E-04	0.0036
ENST000003104540	1.96	9.86E-04	0.0077
ENST000003033050	1.96	4.83E-04	0.0055
RNVU1-7	1.96	2.47E-04	0.0042
KCTD11	1.96	5.08E-04	0.0056
TAGLN2	1.96	9.92E-04	0.0077
MAP2K3	1.96	3.44E-04	0.0048
CCDC51	1.96	5.33E-04	0.0058

PSMB2	1.96	3.14E-04	0.0046
ENST000002489240	1.96	6.27E-04	0.0062
NCKIPSD	1.96	3.01E-04	0.0046
RPS10	1.96	1.52E-04	0.0036
HSPA8P3	1.96	6.62E-03	0.0220
RANGAP1	1.95	3.61E-04	0.0049
RSPH3	1.95	2.12E-04	0.0041
LRP8	1.95	2.86E-03	0.0134
ENST000003155672	1.95	3.03E-04	0.0046
PAM	1.95	1.55E-04	0.0036
NHP2	1.95	4.88E-04	0.0055
TUFM	1.95	9.71E-05	0.0031
AL590399.1	1.95	2.97E-03	0.0136
MLF2	1.95	3.34E-04	0.0047
CD69	1.95	2.58E-04	0.0043
HMBS	1.95	2.87E-03	0.0134
PPP1CC	1.95	3.47E-04	0.0048
GOT1	1.95	1.06E-04	0.0033
FBXL19	1.94	3.39E-04	0.0048
MEX3D	1.94	5.50E-03	0.0196
AL021068.1	1.94	1.04E-02	0.0293
HIST1H2BN	1.94	9.32E-04	0.0075
FERMT2	1.94	6.81E-03	0.0224
UAP1	1.94	4.59E-04	0.0054
AC005229.4	1.94	2.89E-03	0.0135
IKBIP	1.94	5.73E-04	0.0059
HMGN2	1.94	2.31E-04	0.0042
VARS	1.94	9.13E-05	0.0031
INSIG2	1.94	6.68E-04	0.0064
ENST000003234410	1.94	1.21E-02	0.0325
VSIG10	1.94	3.23E-04	0.0047
GTPBP4	1.94	2.26E-04	0.0041
SYP	1.94	8.34E-04	0.0071
CCDC18	1.94	1.38E-04	0.0035
GADD45GIP1	1.94	1.34E-04	0.0035
C19orf38	1.94	1.69E-02	0.0409
ENST000002494420	1.93	1.54E-04	0.0036
ACY1	1.93	1.50E-03	0.0094
ENST000002625070	1.93	6.95E-04	0.0065
ADD2	1.93	1.63E-02	0.0398
PSMB6	1.93	2.09E-04	0.0040
SF3B6	1.93	1.41E-04	0.0036
TUBA4A	1.93	9.54E-04	0.0076
PSMB3	1.93	2.42E-04	0.0042
SNRPD3	1.93	1.56E-04	0.0036
NCAPD3	1.93	4.43E-04	0.0053
LSM3	1.93	3.28E-04	0.0047
ENST000002859680	1.93	2.00E-04	0.0040
ENST000003022710	1.93	2.73E-03	0.0131
MRPL3	1.93	4.03E-04	0.0051

GPATCH4	1.93	8.02E-04	0.0069
ZNF593	1.93	1.59E-03	0.0097
AL133338.1	1.93	3.77E-03	0.0156
TIMM17A	1.93	2.36E-04	0.0042
AC129492.1	1.92	1.73E-03	0.0102
RUVBL1	1.92	3.03E-04	0.0046
BRIX1	1.92	4.73E-04	0.0055
GBAP1	1.92	7.21E-04	0.0065
FAM173A	1.92	6.43E-04	0.0062
GEMIN7	1.92	2.38E-04	0.0042
ENST000001997062	1.92	1.71E-03	0.0101
ABCE1	1.92	3.06E-04	0.0046
CCDC34	1.92	7.63E-03	0.0240
RMI1	1.91	1.83E-04	0.0039
ENST000002316683	1.91	1.39E-03	0.0090
ENST000003131150	1.91	1.70E-03	0.0101
TMEM237	1.91	1.86E-03	0.0105
ENST000003057470	1.91	4.97E-04	0.0056
P3H3	1.91	1.17E-02	0.0317
SMC4	1.91	3.47E-04	0.0048
RBPJ	1.91	1.31E-04	0.0035
NEMP1	1.91	2.40E-04	0.0042
HES6	1.91	2.10E-02	0.0477
MORN2	1.91	1.32E-02	0.0343
PHB2	1.91	1.47E-04	0.0036
ENST000002167560	1.91	2.84E-04	0.0045
C1orf112	1.91	7.82E-04	0.0069
PFDN4	1.91	2.82E-04	0.0045
IFT46	1.91	2.56E-04	0.0043
LINC01465	1.91	4.67E-03	0.0178
SMIM30	1.90	3.03E-04	0.0046
SNORD82	1.90	5.58E-03	0.0197
COX6A1	1.90	1.45E-04	0.0036
ENST000001695510	1.90	7.86E-03	0.0245
DDX21	1.90	4.94E-04	0.0056
YRDC	1.90	3.24E-04	0.0047
SLC9A7	1.90	1.11E-03	0.0081
HMGB1P5	1.90	2.87E-04	0.0045
FKBPL	1.90	4.54E-03	0.0175
ENST000003103800	1.90	3.49E-03	0.0149
CALCRL	1.90	7.01E-03	0.0228
CENPX	1.90	4.54E-03	0.0175
LSM2	1.90	1.33E-03	0.0088
MLH1	1.90	1.05E-04	0.0033
AKAP5	1.90	1.45E-02	0.0367
INSR	1.89	6.43E-04	0.0062
LSM12P1	1.89	3.35E-03	0.0146
ARHGAP31	1.89	5.37E-03	0.0193
STAT3	1.89	3.09E-04	0.0046
ENST000002969330	1.89	3.37E-04	0.0048

AC026401.3	1.89	1.19E-03	0.0083
AC243742.3	1.89	1.19E-03	0.0083
PTPRG	1.89	5.00E-03	0.0185
LYPLA1	1.89	1.23E-04	0.0034
NRSN2-AS1	1.89	1.09E-03	0.0080
PEA15	1.89	2.64E-04	0.0044
TKFC	1.89	2.24E-04	0.0041
TNIP3	1.88	1.26E-03	0.0086
NPM1	1.88	3.12E-04	0.0046
CHSY1	1.88	3.10E-03	0.0140
SINHCAF	1.88	9.64E-05	0.0031
MTCH2	1.87	2.22E-04	0.0041
PRXL2A	1.87	8.61E-04	0.0072
ENST000002823440	1.87	2.34E-04	0.0042
IARS	1.87	1.05E-03	0.0079
ENST000000751201	1.87	1.08E-04	0.0033
MLF1	1.87	8.17E-03	0.0251
STOM	1.87	2.65E-03	0.0129
RFTN1	1.87	2.91E-04	0.0045
GNPDA1	1.87	2.42E-03	0.0122
MACROD1	1.87	1.65E-03	0.0099
ETFB	1.87	1.49E-04	0.0036
GNG5	1.87	1.05E-03	0.0079
HYAL2	1.87	8.09E-04	0.0070
ACTL6A	1.87	1.20E-04	0.0034
NABP2	1.87	3.14E-04	0.0046
DHX32	1.87	6.94E-04	0.0065
RAB11FIP5	1.87	1.03E-03	0.0078
EARS2	1.86	9.96E-04	0.0077
MPHOSPH6	1.86	2.01E-04	0.0040
BNIP3L	1.86	2.35E-04	0.0042
EIF5A	1.86	4.13E-03	0.0165
PLCXD1	1.86	2.96E-03	0.0136
MIR17HG	1.86	1.53E-03	0.0095
MRPL51	1.86	2.37E-04	0.0042
PPP5C	1.86	2.06E-04	0.0040
CD72	1.86	8.41E-04	0.0071
AP002990.1	1.86	3.23E-03	0.0142
RRM2B	1.86	6.74E-04	0.0064
NDUFAF8	1.86	2.19E-03	0.0115
MAGOHB	1.86	1.28E-03	0.0086
DARS	1.86	3.05E-04	0.0046
PRDX1	1.86	2.87E-03	0.0134
LONRF1	1.86	3.94E-04	0.0050
OAZ1	1.86	1.13E-04	0.0033
EED	1.86	1.50E-04	0.0036
ZEB2	1.86	4.18E-04	0.0052
UQCRH	1.86	1.61E-04	0.0037
CCT6A	1.86	1.49E-04	0.0036
ENST000002606651	1.86	3.15E-04	0.0046

BOLA2B	1.86	2.00E-04	0.0040
TIMM50	1.85	1.82E-04	0.0039
EIF2D	1.85	1.10E-04	0.0033
SLC16A1	1.85	8.29E-04	0.0071
PPAT	1.85	7.87E-04	0.0069
RAB8B	1.85	7.53E-04	0.0067
SNX21	1.85	8.21E-03	0.0252
TFR2	1.85	8.94E-03	0.0265
ROMO1	1.85	2.56E-04	0.0043
FADS1	1.85	2.81E-03	0.0133
ENST000003248711	1.85	1.40E-03	0.0091
HPRT1	1.85	4.95E-04	0.0056
ABCB6	1.85	1.97E-04	0.0040
SPTY2D1OS	1.85	7.47E-03	0.0237
COX8A	1.85	7.07E-04	0.0065
GARS	1.85	1.64E-03	0.0099
HSD17B12	1.85	1.00E-02	0.0286
MRPS23	1.84	4.47E-04	0.0053
TEX30	1.84	3.56E-04	0.0048
MCAT	1.84	3.53E-03	0.0150
RCC2	1.84	1.72E-04	0.0038
SAMD1	1.84	1.82E-03	0.0104
CDKN2AIPNL	1.84	1.69E-04	0.0038
PELO	1.84	1.73E-03	0.0102
TIMM23	1.84	1.13E-04	0.0033
AP3S1	1.84	3.92E-04	0.0050
FDX2	1.84	1.17E-03	0.0083
ENST000002197820	1.84	6.02E-03	0.0207
MAGEF1	1.84	6.30E-04	0.0062
PLA2G16	1.84	7.41E-03	0.0235
PPA1	1.84	2.60E-04	0.0044
CIAO2B	1.84	4.93E-04	0.0056
MCOLN2	1.84	1.77E-02	0.0423
AC112496.1	1.84	3.06E-03	0.0139
MZB1	1.83	1.84E-02	0.0435
CLUH	1.83	3.66E-04	0.0049
EHBP1L1	1.83	3.99E-04	0.0051
OTUB2	1.83	4.48E-03	0.0173
CYB5R2	1.83	6.71E-03	0.0222
SLC25A15	1.83	3.31E-04	0.0047
CYSTM1	1.83	6.34E-04	0.0062
DSN1	1.83	3.05E-04	0.0046
ENST000002979330	1.83	5.79E-04	0.0060
TRIM6	1.82	1.92E-02	0.0448
TPM4	1.82	2.12E-04	0.0041
YBX3	1.82	9.53E-03	0.0277
DNAJB5	1.82	1.92E-02	0.0448
EIF3I	1.82	1.58E-04	0.0037
ENST000003165190	1.82	2.35E-04	0.0042
CYBRD1	1.82	3.35E-03	0.0146

ENST000002624322	1.82	2.36E-04	0.0042
POLD3	1.82	1.80E-04	0.0039
AHSA1	1.82	1.90E-04	0.0039
GSTO1	1.82	6.37E-04	0.0062
FAAP24	1.82	1.04E-03	0.0079
GAR1	1.82	1.18E-03	0.0083
ENST000002167740	1.82	1.81E-04	0.0039
RNF19A	1.82	2.46E-03	0.0124
UBE2V2	1.81	2.11E-04	0.0041
TOMM6	1.81	1.86E-04	0.0039
ELAVL1	1.81	1.21E-04	0.0034
ING2	1.81	3.13E-04	0.0046
ELOVL4	1.81	2.13E-02	0.0483
PCK2	1.81	1.09E-03	0.0080
VBP1	1.81	2.59E-04	0.0044
F5	1.81	4.11E-03	0.0164
MVK	1.81	3.77E-04	0.0050
TTLL12	1.81	2.03E-04	0.0040
LSM12	1.81	1.44E-04	0.0036
HSPH1	1.81	2.02E-04	0.0040
GJB6	1.81	3.00E-03	0.0137
HMGB1	1.81	2.00E-04	0.0040
SLCO4A1	1.81	2.76E-04	0.0044
ENST000002660140	1.81	2.47E-03	0.0124
HAT1	1.80	4.06E-04	0.0051
RCCD1	1.80	4.95E-04	0.0056
PSMD3	1.80	2.39E-04	0.0042
DUSP23	1.80	5.46E-04	0.0058
RBM19	1.80	1.30E-04	0.0035
PSMB1	1.80	2.77E-04	0.0044
XRCC6	1.79	1.88E-04	0.0039
BANF1	1.79	1.44E-02	0.0365
MSH6	1.79	1.61E-04	0.0037
ENST000002161941	1.79	1.90E-04	0.0039
PPIP5K1	1.79	4.89E-03	0.0182
NRAS	1.79	2.29E-04	0.0042
ENST000002653040	1.79	7.06E-04	0.0065
PDAP1	1.79	3.55E-04	0.0048
ENST000003387581	1.79	1.72E-03	0.0101
FANCE	1.79	7.83E-04	0.0069
ANKRD9	1.79	1.46E-03	0.0093
WARS2	1.79	3.09E-04	0.0046
PFKFB3	1.79	2.12E-03	0.0113
DLD	1.79	1.16E-04	0.0034
ENST000003268400	1.79	1.45E-02	0.0368
TSPYL5	1.79	1.43E-02	0.0364
PIGA	1.79	2.35E-04	0.0042
OTUD7B	1.79	1.37E-04	0.0035
MRM3	1.79	4.09E-04	0.0051
TMEM217	1.78	1.48E-02	0.0372

ATP5MF	1.78	2.17E-04	0.0041
MAGI1	1.78	6.59E-03	0.0220
FH	1.78	2.80E-04	0.0045
ANKRD7	1.78	6.71E-03	0.0222
UBB	1.78	1.79E-04	0.0039
ALKBH5	1.78	1.30E-04	0.0035
AC245033.4	1.78	6.87E-03	0.0226
SOX4	1.78	3.54E-03	0.0150
PLEKHH1	1.78	4.81E-03	0.0180
RUNX3	1.78	3.48E-04	0.0048
NECTIN3	1.78	7.52E-03	0.0238
ENST000002585342	1.78	8.02E-03	0.0248
ENST000002222542	1.77	1.09E-02	0.0302
UTP20	1.77	6.12E-04	0.0061
LINC01952	1.77	1.49E-02	0.0373
EFNA3	1.77	8.36E-03	0.0254
APOO	1.77	3.20E-04	0.0047
TOMM22	1.77	1.92E-04	0.0039
TRMT10C	1.77	4.83E-04	0.0055
PSMB7	1.77	2.79E-04	0.0045
CDC123	1.77	5.28E-04	0.0057
MRPS18A	1.77	2.35E-04	0.0042
HCG20	1.77	1.20E-02	0.0323
WSB1	1.77	3.80E-04	0.0050
ENST000003060610	1.77	8.16E-03	0.0251
BZW2	1.77	1.25E-04	0.0035
ASL	1.76	5.69E-04	0.0059
MRPS17	1.76	1.86E-04	0.0039
FZD6	1.76	3.49E-04	0.0048
HNRNPC	1.76	1.53E-04	0.0036
GPR108	1.76	1.24E-02	0.0330
PES1	1.76	2.43E-03	0.0123
FOXRED2	1.76	4.99E-03	0.0184
GCHFR	1.76	4.25E-04	0.0052
DCTN6	1.76	2.76E-04	0.0044
PSMA1	1.76	1.47E-04	0.0036
GRPEL1	1.76	7.19E-04	0.0065
EIF4H	1.76	1.25E-04	0.0035
PHLPP1	1.76	7.62E-03	0.0240
PSMA4	1.76	1.51E-04	0.0036
PSMD8	1.76	5.60E-04	0.0059
TNFSF4	1.76	2.88E-03	0.0134
TIGIT	1.76	8.57E-04	0.0072
AK7	1.76	4.11E-03	0.0164
BIK	1.76	5.68E-03	0.0200
ZNF35	1.76	2.03E-03	0.0110
ENST000003296080	1.76	5.10E-03	0.0187
GTDC1	1.76	3.52E-04	0.0048
TIMM13	1.76	4.86E-04	0.0055
NUP62	1.75	2.34E-03	0.0120

ZNF670	1.75	4.25E-04	0.0052
ENOPH1	1.75	2.47E-04	0.0042
PARP2	1.75	1.46E-04	0.0036
RRP7A	1.75	2.14E-04	0.0041
RF00003.26	1.75	1.19E-02	0.0321
RF00003.41	1.75	1.19E-02	0.0321
IL21R	1.75	5.21E-03	0.0190
DNAJA1	1.75	1.78E-04	0.0039
ATP5MC1	1.75	6.66E-04	0.0064
YWHAH	1.75	2.50E-03	0.0124
ENST000003401921	1.75	4.86E-03	0.0181
NANP	1.75	4.10E-04	0.0051
FBL	1.75	2.22E-04	0.0041
LRRC75A-AS1	1.75	4.68E-04	0.0054
AC084082.1	1.75	7.66E-03	0.0241
STAP2	1.75	1.84E-03	0.0105
ATP1B3	1.75	4.42E-04	0.0053
ENST000003306510	1.74	9.07E-04	0.0074
AL445231.1	1.74	6.24E-03	0.0212
IMPA2	1.74	2.72E-03	0.0131
CMSS1	1.74	1.33E-03	0.0088
FASN	1.74	3.39E-04	0.0048
NUP107	1.74	3.17E-04	0.0046
PSMB5	1.74	1.73E-03	0.0102
TFB2M	1.74	3.42E-03	0.0147
SRM	1.74	1.41E-02	0.0360
CYFIP1	1.73	4.61E-04	0.0054
SCOC	1.73	2.82E-04	0.0045
FOSB	1.73	2.66E-03	0.0129
PRIM1	1.73	4.24E-04	0.0052
DONSON	1.73	2.40E-04	0.0042
CCDC6	1.73	2.14E-04	0.0041
LRR1	1.73	7.05E-04	0.0065
CCT3	1.73	2.07E-04	0.0040
ENST000003464030	1.73	2.15E-02	0.0487
LMAN1	1.73	8.11E-04	0.0070
AL441992.1	1.73	7.64E-04	0.0068
AP1S1	1.73	3.69E-03	0.0154
KCNQ5-IT1	1.73	2.90E-03	0.0135
TMEM56	1.73	9.34E-03	0.0273
BCL2A1	1.73	1.37E-04	0.0035
NME2	1.72	3.55E-04	0.0048
FAM86C1	1.72	1.56E-02	0.0387
AL121603.2	1.72	6.11E-04	0.0061
PRRT3	1.72	6.91E-03	0.0226
SLX1B	1.72	5.12E-04	0.0056
PIGW	1.72	1.46E-03	0.0093
SPTSSA	1.72	1.11E-03	0.0081
USP54	1.72	1.29E-02	0.0339
EIF4G1	1.72	2.99E-04	0.0046

RPL7L1	1.72	3.13E-04	0.0046
SLC19A1	1.71	1.01E-03	0.0078
BRWD1-AS2	1.71	9.19E-03	0.0270
CYB5B	1.71	6.46E-04	0.0062
C5orf30	1.71	2.10E-03	0.0113
COA7	1.71	1.94E-04	0.0039
KHSRP	1.71	2.30E-04	0.0042
RAD51C	1.71	2.45E-04	0.0042
GALM	1.71	1.52E-04	0.0036
EZH2	1.71	3.50E-04	0.0048
GEMIN4	1.71	2.52E-04	0.0043
ZNF395	1.71	8.48E-04	0.0071
CHCHD6	1.71	7.30E-04	0.0066
POLDIP2	1.71	5.34E-04	0.0058
ENST000003419800	1.71	2.53E-03	0.0125
RAD18	1.71	2.51E-04	0.0043
MTMR2	1.71	1.09E-03	0.0080
PINX1	1.71	6.35E-04	0.0062
MRPL47	1.71	4.62E-04	0.0054
CKAP5	1.71	1.92E-04	0.0039
EFEMP2	1.70	9.82E-03	0.0283
MTBP	1.70	3.99E-04	0.0051
ACTG1	1.70	3.03E-04	0.0046
ENST000003122630	1.70	6.63E-04	0.0063
ITSN1	1.70	9.46E-04	0.0076
MRPS34	1.70	1.65E-04	0.0037
ATP5MC3	1.70	1.54E-04	0.0036
AC069499.1	1.70	3.16E-03	0.0141
DUSP14	1.70	5.26E-04	0.0057
SNORD12B	1.70	7.38E-03	0.0235
SLIT3	1.70	6.30E-03	0.0213
RCC1L	1.70	2.41E-04	0.0042
ENSA	1.70	3.25E-04	0.0047
ENST000003142620	1.70	2.43E-03	0.0123
PSMC1	1.70	2.60E-04	0.0044
PSMA3	1.70	1.95E-04	0.0040
OXCT1	1.69	2.05E-03	0.0111
UROD	1.69	4.63E-04	0.0054
MRPL28	1.69	8.01E-03	0.0248
NACC1	1.69	7.80E-04	0.0068
ENST000003506380	1.69	7.36E-03	0.0234
POLR2K	1.69	2.47E-04	0.0042
GPNMB	1.69	6.04E-03	0.0208
IDI1	1.69	5.53E-04	0.0058
LRP12	1.69	4.99E-03	0.0184
C1orf198	1.69	1.89E-02	0.0443
ENST000000800591	1.69	1.30E-02	0.0341
AL512633.1	1.69	1.25E-02	0.0332
UBBP4	1.69	9.40E-03	0.0274
MRPS15	1.69	1.99E-04	0.0040

SDAD1P1	1.69	1.81E-02	0.0430
ANO7	1.69	8.14E-03	0.0251
PSMD13	1.69	1.81E-04	0.0039
LRRN4CL	1.69	1.15E-02	0.0314
PC	1.69	9.87E-03	0.0283
RFWD3	1.69	2.75E-04	0.0044
EIF2S1	1.68	2.74E-04	0.0044
COMTD1	1.68	1.10E-03	0.0080
ENST000002760960	1.68	2.38E-03	0.0121
ENST000003262662	1.68	3.35E-04	0.0047
DLK2	1.68	1.18E-02	0.0320
EIF2B3	1.68	3.88E-04	0.0050
ENST000003571660	1.68	9.57E-03	0.0277
MLST8	1.68	4.27E-04	0.0052
MDH2	1.67	2.56E-03	0.0126
POP7	1.67	7.15E-04	0.0065
RPGRIP1L	1.67	6.08E-04	0.0061
SRSF1	1.67	2.57E-04	0.0043
FBXO45	1.67	3.59E-04	0.0049
MRPS2	1.67	3.94E-04	0.0050
LSM11	1.67	3.90E-04	0.0050
ENST00000508524	1.67	1.60E-03	0.0098
MRPL21	1.67	3.75E-03	0.0156
PFAS	1.67	8.60E-04	0.0072
HK1	1.67	2.90E-04	0.0045
PPFIA3	1.67	1.67E-02	0.0405
SLC3A2	1.67	1.30E-03	0.0087
MRPL33	1.67	5.14E-04	0.0057
CSPG5	1.67	3.00E-03	0.0137
ACTB	1.67	2.15E-04	0.0041
PRPF4	1.67	4.71E-04	0.0054
PFDN2	1.67	3.94E-04	0.0050
SLC25A32	1.67	3.11E-04	0.0046
ENST000003567081	1.67	1.59E-04	0.0037
SDHB	1.67	2.45E-04	0.0042
YEATS4	1.66	3.92E-03	0.0160
PTPN14	1.66	3.72E-04	0.0049
CSE1L	1.66	4.11E-04	0.0051
EIF1AX	1.66	1.06E-02	0.0298
EMC6	1.66	4.11E-03	0.0164
SESTD1	1.66	1.89E-02	0.0442
ENST000003334834	1.66	1.01E-02	0.0287
PPIH	1.66	9.89E-04	0.0077
NOP56	1.66	5.02E-04	0.0056
PSTPIP2	1.66	2.61E-04	0.0044
GLIDR	1.65	6.95E-03	0.0227
JPT2	1.65	9.01E-04	0.0074
RBBP7	1.65	1.66E-04	0.0038
EMC9	1.65	9.47E-04	0.0076
EXOSC8	1.65	1.84E-04	0.0039

ENST000002562160	1.65	9.58E-04	0.0076
PEMT	1.65	8.20E-04	0.0070
ADPRHL2	1.65	7.76E-04	0.0068
BCKDHA	1.65	7.72E-04	0.0068
ENST000002155871	1.65	7.00E-04	0.0065
BICDL2	1.65	3.51E-03	0.0149
MIR621	1.65	2.38E-03	0.0121
ENST000003048003	1.65	1.25E-02	0.0332
STARD7	1.65	1.70E-04	0.0038
METAP2	1.65	2.62E-04	0.0044
MT-ND6	1.65	9.72E-04	0.0076
KIAA1324	1.64	2.11E-02	0.0480
NUTF2	1.64	4.05E-04	0.0051
MT2A	1.64	2.37E-03	0.0121
NFKB2	1.64	3.81E-03	0.0157
AC007384.1	1.64	3.68E-03	0.0154
ENST000003519860	1.64	2.06E-04	0.0040
NIPAL1	1.64	2.21E-03	0.0116
AC144652.1	1.64	9.20E-03	0.0270
BOLA2-SMG1P6	1.64	2.19E-02	0.0493
ATP5PF	1.64	2.46E-04	0.0042
PMF1	1.64	2.85E-03	0.0134
TRIAP1	1.64	1.65E-03	0.0099
AP001347.1	1.64	1.46E-03	0.0093
ATP5F1B	1.64	3.02E-04	0.0046
EID1	1.64	3.80E-04	0.0050
RPIA	1.63	5.16E-03	0.0188
ENST000002611911	1.63	4.56E-04	0.0054
BUD31	1.63	3.15E-04	0.0046
ENST000002854070	1.63	7.66E-03	0.0241
AP000769.1	1.63	2.13E-03	0.0114
ANKRD34A	1.63	2.44E-03	0.0123
NUP93	1.63	5.09E-04	0.0056
C1orf174	1.63	5.35E-04	0.0058
ENST000002820580	1.63	2.22E-04	0.0041
FAM153B	1.63	3.04E-03	0.0138
C5orf34	1.63	5.08E-04	0.0056
CDC42BPB	1.63	3.92E-03	0.0160
E2F5	1.63	1.44E-02	0.0366
CBX3	1.63	1.96E-04	0.0040
ELOVL6	1.63	3.74E-03	0.0155
DDR1	1.63	2.76E-03	0.0131
ENST000003090611	1.63	7.79E-03	0.0243
NUP35	1.63	3.34E-04	0.0047
IDH2	1.63	2.23E-03	0.0117
IFT81	1.63	7.44E-04	0.0067
STARD4	1.63	1.53E-02	0.0381
CDK7	1.63	4.73E-04	0.0055
DHODH	1.63	1.04E-03	0.0078
CIAO2A	1.62	1.80E-04	0.0039

CUL7	1.62	3.59E-04	0.0049
AC093616.1	1.62	2.06E-02	0.0471
BNIP1	1.62	4.86E-04	0.0055
RRP9	1.62	2.91E-03	0.0135
CCNB1IP1	1.62	5.23E-04	0.0057
SZRD1	1.62	6.05E-04	0.0061
NAP1L5	1.62	2.33E-03	0.0120
VDAC2	1.62	2.19E-04	0.0041
TXNL4A	1.62	5.77E-04	0.0060
JPT1	1.62	3.21E-04	0.0047
TRMT5	1.62	6.05E-04	0.0061
SNORA80D	1.62	1.52E-02	0.0379
TMEM107	1.62	1.25E-03	0.0085
ACVR1B	1.62	2.74E-03	0.0131
CHRAC1	1.62	7.73E-04	0.0068
LNP1	1.62	6.89E-03	0.0226
C5orf15	1.62	3.71E-04	0.0049
ENST000003588670	1.62	2.34E-03	0.0120
ADGRG1	1.62	4.36E-03	0.0170
GSTP1	1.62	1.30E-03	0.0087
AC009275.1	1.62	1.44E-02	0.0365
DOHH	1.61	1.06E-03	0.0079
CD3EAP	1.61	1.28E-02	0.0337
RTCB	1.61	2.15E-04	0.0041
WDR90	1.61	6.77E-04	0.0064
C12orf60	1.61	4.00E-03	0.0161
TRIB3	1.61	3.09E-03	0.0140
TMED1	1.61	1.13E-03	0.0081
ACSL1	1.61	1.21E-03	0.0084
NEIL3	1.61	7.69E-04	0.0068
ENST000003121890	1.61	5.93E-03	0.0205
EIF3B	1.61	3.25E-04	0.0047
ENST000002561902	1.61	3.47E-04	0.0048
PM20D2	1.61	7.88E-04	0.0069
GOLGA2P7	1.61	4.84E-03	0.0181
SUPT16H	1.61	2.84E-04	0.0045
FARSA	1.61	1.07E-03	0.0080
ENST000002616000	1.61	8.62E-04	0.0072
ENST000003048743	1.61	1.62E-03	0.0098
ZMYND19	1.61	3.03E-04	0.0046
AJUBA	1.61	1.42E-03	0.0091
NUDC	1.61	1.17E-03	0.0083
KIAA1958	1.60	2.08E-02	0.0475
RILPL2	1.60	5.14E-04	0.0057
UBA1	1.60	5.96E-04	0.0060
TMEM201	1.60	2.08E-03	0.0112
PFDN6	1.60	2.44E-04	0.0042
UQCRFS1	1.60	2.09E-04	0.0040
ENST000002620330	1.60	6.98E-04	0.0065
CD22	1.60	8.63E-03	0.0259

MCTS1	1.60	2.32E-04	0.0042
TOPBP1	1.60	2.14E-04	0.0041
PUM3	1.60	8.67E-04	0.0072
ENST000003512880	1.60	7.93E-04	0.0069
SCLY	1.60	7.05E-04	0.0065
ENST000002289551	1.60	1.77E-03	0.0102
GEMIN5	1.60	3.46E-04	0.0048
NOP10	1.60	3.16E-04	0.0046
FAM229B	1.60	8.15E-04	0.0070
NR6A1	1.60	2.39E-03	0.0122
ATF4	1.60	1.60E-03	0.0098
TIMM17B	1.59	2.77E-03	0.0132
PXMP2	1.59	7.99E-03	0.0247
RPS10-NUDT3	1.59	4.16E-04	0.0051
ENST000002705171	1.59	3.77E-03	0.0156
AC026464.6	1.59	2.90E-03	0.0135
IPO5	1.59	3.94E-04	0.0050
LMCD1	1.59	1.54E-03	0.0095
EIF6	1.59	2.13E-03	0.0114
UQCC2	1.59	8.51E-04	0.0071
MRPL16	1.59	2.35E-04	0.0042
TTC27	1.59	7.47E-04	0.0067
PKIG	1.59	1.12E-02	0.0308
DYNC2LI1	1.59	1.48E-02	0.0372
EPS8L2	1.59	2.51E-03	0.0124
WDR12	1.59	7.83E-04	0.0069
FARSB	1.59	2.70E-04	0.0044
FARP2	1.58	8.53E-04	0.0072
HDAC7	1.58	2.58E-03	0.0127
ENST000003010711	1.58	3.35E-04	0.0047
AC002407.1	1.58	2.06E-02	0.0472
TMEM126A	1.58	8.99E-04	0.0074
AC099522.2	1.58	1.04E-02	0.0293
RNLS	1.58	1.61E-02	0.0395
CEP72	1.58	1.88E-03	0.0106
FIRRE	1.58	1.87E-03	0.0105
MRPS28	1.58	5.76E-04	0.0060
ENST000003347051	1.58	2.93E-03	0.0135
SRRD	1.58	1.36E-03	0.0089
DNAJC9	1.58	3.42E-04	0.0048
ITGB1BP2	1.58	2.73E-03	0.0131
HTRA2	1.58	6.87E-04	0.0064
CBX1	1.58	5.10E-04	0.0056
ENST000002517220	1.58	5.05E-03	0.0186
YWHAE	1.58	1.55E-03	0.0096
PGM1	1.58	5.17E-04	0.0057
GTF3C5	1.58	3.56E-04	0.0048
ANAPC1	1.58	5.70E-04	0.0059
GRSF1	1.58	2.91E-04	0.0045
CCND2	1.58	1.53E-03	0.0095

C7orf50	1.58	1.60E-02	0.0393
PHF5A	1.57	1.87E-03	0.0106
GART	1.57	2.53E-04	0.0043
AC097534.2	1.57	4.87E-03	0.0182
ECHS1	1.57	2.93E-04	0.0045
PGRMC1	1.57	5.20E-04	0.0057
PPP1R16B	1.57	2.34E-04	0.0042
MRPL14	1.57	1.78E-03	0.0103
TMEM99	1.57	5.52E-03	0.0196
POLR2E	1.57	1.84E-03	0.0105
MAPKAPK3	1.57	2.90E-03	0.0135
TMA16	1.57	7.62E-04	0.0068
ENST000002730640	1.57	6.17E-03	0.0210
ZCCHC24	1.57	1.19E-02	0.0321
TRIT1	1.57	9.37E-04	0.0075
GRWD1	1.57	5.80E-04	0.0060
SAMD4A	1.57	1.19E-03	0.0083
PDHA1	1.57	2.19E-04	0.0041
STAT5A	1.57	2.28E-04	0.0042
PXN-AS1	1.57	1.19E-03	0.0083
SPIN4	1.57	1.43E-03	0.0092
AP1S3	1.57	4.82E-03	0.0181
PCCB	1.57	4.48E-04	0.0053
AC004847.1	1.57	1.04E-02	0.0293
STAMBP	1.56	2.43E-04	0.0042
SP140	1.56	6.37E-03	0.0215
RBBP9	1.56	2.93E-04	0.0045
ENST000003375140	1.56	2.35E-03	0.0121
HSPA4	1.56	3.10E-04	0.0046
CNOT9	1.56	3.66E-04	0.0049
MFAP1	1.56	2.59E-04	0.0044
EIF4A3	1.56	5.27E-04	0.0057
SAE1	1.56	1.04E-03	0.0079
ARID5A	1.56	1.69E-03	0.0100
ZCRB1	1.56	2.16E-04	0.0041
CD58	1.56	2.81E-03	0.0133
ENST000003301371	1.56	5.31E-04	0.0058
ENST000003154231	1.56	1.28E-03	0.0086
ACTR1B	1.56	3.82E-04	0.0050
GLB1L	1.56	7.32E-03	0.0234
CCDC47	1.56	4.01E-04	0.0051
RAD23A	1.56	1.89E-03	0.0106
LACTB	1.56	8.99E-04	0.0074
ENST000003220302	1.56	4.98E-04	0.0056
FBLN7	1.56	5.72E-04	0.0059
ENST000002204292	1.56	3.36E-03	0.0146
ENST000003552380	1.56	2.76E-04	0.0044
ENST000002646890	1.56	3.82E-04	0.0050
SNHG17	1.56	4.05E-04	0.0051
NAA20	1.56	5.52E-04	0.0058

ENST000002722981	1.56	3.98E-04	0.0051
NXT1	1.55	4.51E-04	0.0054
EEF1AKMT1	1.55	3.78E-03	0.0156
NUCKS1	1.55	2.88E-04	0.0045
SUPT4H1	1.55	2.96E-04	0.0046
BCCIP	1.55	4.84E-04	0.0055
VMP1	1.55	1.33E-03	0.0088
CCNE1	1.55	1.76E-03	0.0102
PI4K2B	1.55	2.46E-04	0.0042
FAR2	1.55	2.16E-02	0.0487
PLXNA1	1.55	1.77E-03	0.0102
PGAM5	1.55	3.73E-04	0.0049
MSH2	1.55	2.97E-04	0.0046
PRKDC	1.55	2.77E-04	0.0044
NDUFS6	1.55	4.76E-04	0.0055
ENST000003067040	1.55	1.68E-03	0.0100
AL035461.2	1.55	5.02E-03	0.0185
MAPK6	1.55	3.66E-03	0.0153
MRPL19	1.55	3.07E-04	0.0046
SNAPC1	1.55	4.05E-04	0.0051
PRELID3B	1.55	5.57E-04	0.0059
NUDCD1	1.54	5.51E-04	0.0058
RYBP	1.54	4.54E-04	0.0054
PMPCA	1.54	5.77E-04	0.0060
ASPHD2	1.54	3.92E-03	0.0160
IFNLR1	1.54	3.16E-03	0.0141
EBP	1.54	7.02E-04	0.0065
ENST000002637950	1.54	6.02E-03	0.0207
INAFM2	1.54	3.35E-03	0.0146
SNRPA	1.54	3.17E-03	0.0141
POLR1A	1.54	1.66E-03	0.0099
AC127070.1	1.54	1.55E-02	0.0384
MTX1	1.54	3.26E-04	0.0047
ENST000003278000	1.54	9.66E-04	0.0076
VRK1	1.54	2.39E-04	0.0042
MRPS26	1.54	1.24E-02	0.0330
MRPS18B	1.54	7.48E-04	0.0067
ENST000002994383	1.54	4.38E-03	0.0171
GHITM	1.54	4.96E-03	0.0184
SLC7A1	1.54	8.16E-03	0.0251
TMEM69	1.53	3.98E-04	0.0051
ENST000003136082	1.53	3.41E-04	0.0048
SNORA14B	1.53	5.51E-03	0.0196
CDK4	1.53	9.25E-03	0.0271
CBX6	1.53	2.50E-04	0.0043
RAB8A	1.53	7.31E-04	0.0066
TNC	1.53	1.34E-02	0.0348
STRBP	1.53	6.24E-04	0.0062
HIST2H2AC	1.53	3.59E-04	0.0049
EFTUD2	1.53	9.74E-04	0.0076

ACP1	1.53	6.62E-04	0.0063
ENST000002680350	1.53	5.55E-03	0.0197
ENST000003448431	1.52	7.35E-04	0.0066
PNPT1	1.52	6.70E-04	0.0064
SMIM15	1.52	7.30E-04	0.0066
MRPL15	1.52	4.41E-04	0.0053
CPSF3	1.52	2.98E-04	0.0046
TEC	1.52	9.15E-04	0.0074
ENST00000621148	1.52	2.09E-02	0.0476
ZNF653	1.52	1.94E-03	0.0107
ENST000003245930	1.52	7.72E-03	0.0242
DHRS3	1.52	1.00E-03	0.0077
ENST000003042710	1.52	4.42E-03	0.0172
UBE2L3	1.52	3.24E-04	0.0047
WDR35	1.52	4.77E-03	0.0180
ENST000003152862	1.52	4.35E-04	0.0053
ATP13A3	1.52	6.91E-04	0.0064
PLEKHG4	1.51	5.72E-04	0.0059
SARNP	1.51	2.92E-04	0.0045
SLBP	1.51	2.68E-04	0.0044
RDX	1.51	1.77E-03	0.0102
ENST000003017641	1.51	4.69E-04	0.0054
MRM1	1.51	4.16E-03	0.0165
XPNPEP1	1.51	4.18E-04	0.0052
ENST000002644511	1.51	6.93E-03	0.0227
SMYD5	1.51	1.61E-03	0.0098
APRT	1.51	3.22E-04	0.0047
PKP4	1.51	4.94E-04	0.0056
NDUFB3	1.51	2.75E-04	0.0044
DAZAP1	1.51	6.56E-04	0.0063
GPD2	1.51	9.58E-04	0.0076
ILVBL	1.51	6.01E-04	0.0060
EIF5B	1.51	3.63E-04	0.0049
SF3B5	1.51	2.77E-04	0.0044
COL27A1	1.51	1.10E-03	0.0080
MRPS12	1.51	1.31E-03	0.0087
ZBTB17	1.51	3.26E-04	0.0047
SURF4	1.51	7.72E-04	0.0068
PDIA6	1.51	5.60E-04	0.0059
SNHG25	1.50	2.92E-03	0.0135
SRPK1	1.50	2.46E-04	0.0042
SARS2	1.50	1.75E-03	0.0102
SMC1A	1.50	1.51E-02	0.0377
ERF	1.50	1.04E-02	0.0292
SLC30A9	1.50	2.68E-03	0.0129
ETFA	1.50	3.69E-04	0.0049
PDCD5	1.50	3.34E-04	0.0047
HAUS7	1.50	3.17E-03	0.0141
AP002360.1	1.50	1.22E-03	0.0084
ENST000003287031	1.50	2.99E-03	0.0137

SNX8	1.50	1.87E-03	0.0105
KPTN	1.50	4.37E-03	0.0170
RF00003.32	1.50	2.86E-03	0.0134
RF00003.40	1.50	2.86E-03	0.0134
MRPL45	1.50	2.25E-03	0.0117
ENST000002912953	1.50	1.06E-03	0.0079
SNHG10	1.50	7.08E-04	0.0065
VASH1	1.50	3.40E-03	0.0147
POP1	1.49	7.40E-04	0.0066
ENST000003299660	1.49	2.64E-03	0.0128
GLRX3	1.49	7.32E-04	0.0066
COA6	1.49	5.87E-04	0.0060
ANK1	1.49	6.71E-03	0.0222
TFAM	1.49	5.40E-04	0.0058
COX7A2	1.49	2.99E-04	0.0046
ENST000002605851	1.49	4.64E-04	0.0054
HAUS8	1.49	7.66E-04	0.0068
PEX10	1.49	1.60E-03	0.0098
ENST000002885320	1.49	3.89E-04	0.0050
ENST000003086960	1.49	2.55E-03	0.0126
ASNS	1.49	7.47E-04	0.0067
SLC25A3	1.49	2.64E-04	0.0044
HSD17B6	1.49	2.95E-03	0.0136
LRRCC1	1.49	6.76E-04	0.0064
MAMDC4	1.49	1.77E-02	0.0421
HNRNPA1P48	1.49	4.67E-03	0.0178
JUN	1.49	5.54E-03	0.0197
ROBO3	1.49	7.80E-03	0.0243
PTBP1	1.49	1.04E-03	0.0078
PSMB8	1.48	2.80E-04	0.0045
NEIL2	1.48	1.06E-03	0.0079
NUDT21	1.48	3.39E-04	0.0048
EIF2S2	1.48	7.45E-04	0.0067
DLAT	1.48	3.26E-03	0.0143
SLC25A1	1.48	1.07E-03	0.0080
TDRKH	1.48	1.53E-03	0.0095
NOC3L	1.48	1.01E-03	0.0078
DPAGT1	1.48	1.73E-03	0.0102
H2AFV	1.48	4.66E-04	0.0054
STRAP	1.48	3.75E-04	0.0049
AC145207.5	1.48	7.10E-04	0.0065
NDUFB6	1.48	2.35E-03	0.0121
ENST000002656632	1.48	1.31E-03	0.0087
DHRS7B	1.48	1.05E-03	0.0079
COX7B	1.48	4.79E-04	0.0055
USP28	1.48	8.47E-04	0.0071
ENST000001972682	1.48	3.32E-03	0.0145
C8orf37	1.48	6.36E-03	0.0214
DCLRE1A	1.48	3.97E-04	0.0051
MAST2	1.48	1.22E-02	0.0326

ENST000003367330	1.48	1.76E-03	0.0102
NAB1	1.48	2.92E-03	0.0135
CETN3	1.47	2.80E-04	0.0045
CPT2	1.47	6.30E-04	0.0062
GPX1	1.47	2.01E-03	0.0110
ABCB10	1.47	1.31E-03	0.0087
PGM2	1.47	4.05E-04	0.0051
ARMC9	1.47	7.20E-03	0.0231
CCDC136	1.47	6.07E-03	0.0208
MVD	1.47	7.68E-04	0.0068
MTND2P28	1.47	8.43E-03	0.0256
ANP32A	1.47	2.74E-04	0.0044
ENST000003437880	1.47	2.77E-04	0.0044
MYO1A	1.47	6.06E-03	0.0208
EEF2KMT	1.47	1.17E-03	0.0083
MRPS35	1.47	6.34E-04	0.0062
ENST000003232050	1.47	1.77E-02	0.0422
ALKBH2	1.47	9.73E-04	0.0076
SRXN1	1.47	3.23E-03	0.0142
RBFA	1.46	5.41E-04	0.0058
NIPSNAP2	1.46	4.27E-04	0.0052
CREB3	1.46	3.95E-04	0.0050
TIMM44	1.46	3.26E-04	0.0047
HEATR1	1.46	5.54E-04	0.0059
SHPK	1.46	2.76E-03	0.0132
DHX33	1.46	6.78E-04	0.0064
HSPA4L	1.46	1.44E-03	0.0092
CARM1	1.46	6.29E-04	0.0062
HDLBP	1.46	4.03E-04	0.0051
TMEM160	1.46	3.74E-03	0.0155
ELK1	1.46	1.89E-03	0.0106
MAP3K6	1.46	2.42E-03	0.0122
PSMD12	1.46	7.58E-04	0.0067
HIST1H2AC	1.46	1.01E-03	0.0077
USP1	1.46	3.86E-04	0.0050
EXOSC2	1.46	4.23E-04	0.0052
IRAK1	1.45	3.55E-03	0.0150
PSMD11	1.45	2.89E-04	0.0045
ARPC5L	1.45	1.75E-03	0.0102
C11orf95	1.45	1.32E-02	0.0345
NFKBID	1.45	2.44E-03	0.0123
COA4	1.45	5.79E-04	0.0060
WDR45B	1.45	2.83E-04	0.0045
CCDC71L	1.45	1.48E-03	0.0093
ENST000003307522	1.45	8.41E-04	0.0071
GCSH	1.45	3.74E-03	0.0155
MINDY4	1.45	1.63E-02	0.0398
SREBF1	1.45	1.21E-03	0.0084
WDR43	1.45	7.09E-04	0.0065
NOP58	1.45	7.37E-04	0.0066

TIMM8B	1.45	3.84E-04	0.0050
MRPL22	1.45	3.47E-04	0.0048
WBP4	1.45	5.07E-04	0.0056
GLRX5	1.44	9.98E-04	0.0077
DBF4	1.44	3.35E-04	0.0047
GLS2	1.44	1.20E-02	0.0323
ENST000002632700	1.44	7.16E-04	0.0065
ENST000002799071	1.44	5.83E-04	0.0060
ODF2	1.44	1.20E-03	0.0084
TRIM69	1.44	6.80E-04	0.0064
RPLP0	1.44	6.09E-04	0.0061
ENST000003175020	1.44	2.44E-03	0.0123
APOBEC3G	1.44	5.94E-03	0.0205
HUWE1	1.44	4.05E-04	0.0051
ICA1L	1.44	7.47E-04	0.0067
AC132192.2	1.44	2.42E-03	0.0122
CENPL	1.44	8.86E-04	0.0073
AC011462.1	1.44	2.44E-03	0.0123
ENST000003594290	1.44	4.02E-03	0.0162
CEP152	1.44	6.31E-04	0.0062
UBR7	1.44	3.13E-04	0.0046
PSPH	1.44	1.65E-02	0.0401
AC083899.1	1.43	1.16E-03	0.0082
NOC2L	1.43	1.21E-03	0.0084
GTF2H3	1.43	1.88E-03	0.0106
AARSD1	1.43	2.88E-03	0.0134
ENST000003112751	1.43	1.75E-02	0.0419
NOL7	1.43	5.19E-04	0.0057
GSTZ1	1.43	1.72E-03	0.0101
MAP7	1.43	8.24E-03	0.0253
WDR17	1.43	3.99E-03	0.0161
XPO1	1.43	3.98E-04	0.0051
NT5M	1.43	4.73E-03	0.0179
ENST000003310790	1.43	8.49E-04	0.0071
MRPL27	1.43	1.14E-03	0.0082
SNORD104	1.43	1.29E-02	0.0339
EIF3C	1.43	2.98E-04	0.0046
CCDC138	1.43	4.44E-04	0.0053
NDUFB8	1.43	2.99E-04	0.0046
JOSD1	1.43	3.34E-04	0.0047
KIF21A	1.43	1.02E-02	0.0290
KARS	1.43	4.98E-04	0.0056
SNORA79B	1.43	1.17E-02	0.0318
SNX5	1.42	3.44E-04	0.0048
DEK	1.42	3.27E-04	0.0047
CTU2	1.42	5.56E-03	0.0197
HPF1	1.42	2.29E-03	0.0119
CD63	1.42	3.26E-03	0.0143
SUCLA2	1.42	6.53E-04	0.0063
BARD1	1.42	3.63E-04	0.0049

KPNB1	1.42	6.58E-04	0.0063
FANK1	1.42	1.52E-02	0.0380
LIN9	1.42	6.17E-04	0.0061
AMD1	1.42	4.17E-04	0.0052
RHOG	1.42	6.25E-03	0.0212
RGCC	1.42	1.29E-03	0.0087
SENP3	1.42	7.58E-04	0.0067
PSMC4	1.42	3.14E-04	0.0046
GEMIN6	1.42	9.69E-04	0.0076
KIF22	1.42	3.47E-04	0.0048
WDR5	1.42	5.18E-04	0.0057
SNX22	1.42	2.82E-03	0.0133
HDHD5	1.42	6.22E-04	0.0062
PRPS1	1.41	4.89E-04	0.0055
PLAUR	1.41	1.29E-03	0.0087
ATP5F1A	1.41	4.05E-04	0.0051
IPO7	1.41	6.52E-04	0.0063
SNHG1	1.41	2.16E-03	0.0114
GCNT1	1.41	1.00E-02	0.0286
TMX1	1.41	6.26E-04	0.0062
MDH1	1.41	3.47E-04	0.0048
ENST000003449890	1.41	2.24E-03	0.0117
MDFIC	1.41	1.61E-02	0.0395
SOWAHC	1.41	1.16E-02	0.0316
ENST000002615311	1.41	3.20E-04	0.0047
ENST000003230642	1.41	5.53E-03	0.0196
JUND	1.41	5.06E-03	0.0186
ENST000003261971	1.41	2.06E-03	0.0111
GTF2H2C_2	1.41	1.20E-03	0.0084
SRP68	1.41	3.69E-04	0.0049
ENST000002944011	1.41	7.60E-03	0.0239
MISP3	1.41	1.96E-02	0.0455
ENST000002388232	1.41	2.15E-03	0.0114
COTL1	1.41	1.81E-03	0.0104
ACOT4	1.41	4.28E-03	0.0168
ENST000002526224	1.41	1.48E-03	0.0093
MAPK13	1.41	9.54E-04	0.0076
ATP5ME	1.41	1.37E-03	0.0089
ENST000003389950	1.40	2.23E-03	0.0117
SPX	1.40	9.04E-03	0.0267
RBM17	1.40	5.71E-04	0.0059
SNORA70	1.40	2.31E-03	0.0119
CKAP2	1.40	1.43E-03	0.0091
BLVRA	1.40	1.17E-02	0.0317
TAF1A	1.40	8.59E-04	0.0072
SPRYD7	1.40	8.30E-04	0.0071
FAM136A	1.40	3.94E-04	0.0050
PPM1G	1.40	6.69E-04	0.0064
SNRPB2	1.40	4.08E-04	0.0051
SNRNP40	1.40	4.87E-04	0.0055

COX6C	1.40	4.05E-04	0.0051
TOE1	1.40	7.84E-04	0.0069
C9orf43	1.40	9.45E-03	0.0275
IPMK	1.40	4.92E-04	0.0056
SUMO1	1.40	3.59E-04	0.0049
TNPO3	1.40	3.31E-04	0.0047
ASNA1	1.40	5.55E-04	0.0059
AC027559.1	1.40	2.83E-03	0.0133
MCM3	1.40	1.68E-03	0.0100
ENST000002183282	1.39	3.47E-04	0.0048
GPSM1	1.39	1.70E-03	0.0101
ATP5MPL	1.39	6.16E-04	0.0061
ENST000003737150	1.39	1.94E-03	0.0107
ENST000003411540	1.39	8.72E-03	0.0262
RPSA	1.39	5.49E-04	0.0058
CEP57L1	1.39	2.30E-03	0.0119
SDHAF3	1.39	2.41E-03	0.0122
ENST000002256651	1.39	6.23E-04	0.0062
PRELID1P1	1.39	1.62E-02	0.0396
FIBP	1.39	5.99E-04	0.0060
ENST000003328590	1.39	7.96E-04	0.0069
RAB9B	1.39	2.14E-02	0.0484
IDH3A	1.39	5.71E-04	0.0059
ENST000002991734	1.39	1.31E-02	0.0342
ENST000003008704	1.38	3.94E-04	0.0050
CHCHD1	1.38	4.40E-04	0.0053
SCRIB	1.38	9.55E-04	0.0076
DMXL2	1.38	5.66E-03	0.0199
BORA	1.38	6.23E-04	0.0062
APTX	1.38	2.03E-03	0.0110
XRCC5	1.38	3.95E-04	0.0050
PGBD1	1.38	1.55E-02	0.0385
C8orf33	1.38	8.51E-04	0.0071
MRPL18	1.38	2.00E-03	0.0109
AC064850.1	1.38	4.41E-03	0.0172
TWISTNB	1.38	7.07E-04	0.0065
RIDA	1.38	8.21E-04	0.0070
ADO	1.38	4.05E-03	0.0162
SEC61B	1.38	8.58E-04	0.0072
ERI1	1.37	5.95E-04	0.0060
P2RX5	1.37	4.85E-03	0.0181
DRG1	1.37	6.61E-04	0.0063
OLA1	1.37	6.84E-04	0.0064
UBL4A	1.37	6.39E-04	0.0062
GPN1	1.37	5.08E-04	0.0056
ESD	1.37	4.06E-04	0.0051
AFG3L2	1.37	5.50E-04	0.0058
HAUS6	1.37	1.24E-03	0.0085
ENST000002707762	1.37	3.23E-03	0.0142
SF3B3	1.37	5.45E-04	0.0058

UBALD2	1.37	8.63E-03	0.0260
ENST000002525123	1.37	1.46E-03	0.0093
ACACA	1.37	5.16E-04	0.0057
PDCD2L	1.37	1.33E-03	0.0088
PPP4C	1.37	7.09E-03	0.0230
ENST000003117131	1.37	3.85E-03	0.0158
ENST000003589010	1.37	4.54E-04	0.0054
MRPL40	1.37	2.28E-03	0.0119
TOMM40L	1.37	1.03E-03	0.0078
RBX1	1.37	4.81E-04	0.0055
ENST000003299670	1.37	3.67E-03	0.0154
PSME3	1.37	7.37E-04	0.0066
TTC8	1.37	2.13E-03	0.0113
KBTBD8	1.37	6.37E-04	0.0062
ENST000003022431	1.37	5.18E-04	0.0057
PUF60	1.37	6.53E-04	0.0063
EIF2A	1.37	4.29E-04	0.0052
CCNG2	1.36	3.16E-03	0.0141
MRPS16	1.36	4.94E-04	0.0056
RASGRP4	1.36	4.20E-03	0.0166
PHPT1	1.36	1.61E-03	0.0098
ENST000003261810	1.36	1.23E-03	0.0085
PEX3	1.36	1.88E-03	0.0106
GJC1	1.36	3.50E-03	0.0149
PRPF31	1.36	1.23E-03	0.0085
UMPS	1.36	1.81E-03	0.0104
PSMG3	1.36	1.05E-03	0.0079
ENST000002735412	1.36	6.67E-04	0.0064
ENST000002425770	1.36	1.14E-03	0.0081
IPO4	1.36	6.98E-04	0.0065
ENST000003545030	1.36	1.62E-03	0.0098
LINC02595	1.36	5.51E-03	0.0196
NSDHL	1.36	1.20E-03	0.0084
MBD3	1.36	1.52E-03	0.0095
APOBEC3C	1.36	9.04E-03	0.0267
GTF3C6	1.36	1.21E-03	0.0084
ITPA	1.36	2.50E-03	0.0124
ENST000003345981	1.36	1.68E-02	0.0407
TSPAN3	1.36	5.45E-03	0.0195
EMD	1.36	8.86E-04	0.0073
ADK	1.36	5.12E-04	0.0056
DOT1L	1.36	4.09E-03	0.0164
PSMD7	1.36	4.89E-04	0.0055
DDX39A	1.36	6.77E-04	0.0064
NIFK	1.36	1.16E-03	0.0082
ENST000003104411	1.35	2.51E-03	0.0125
TRMT112	1.35	8.34E-04	0.0071
ARMC1	1.35	5.11E-04	0.0056
SNORD93	1.35	1.92E-02	0.0448
EIPR1	1.35	8.84E-04	0.0073

CBWD1	1.35	2.18E-03	0.0115
ENST00000625834	1.35	5.81E-04	0.0060
ZFP91-CNTF	1.35	6.72E-03	0.0222
YEATS2	1.35	4.66E-04	0.0054
PSMD10	1.35	1.12E-03	0.0081
IDH3B	1.35	8.08E-04	0.0070
GNL2	1.35	1.25E-03	0.0086
ABHD10	1.35	5.82E-04	0.0060
TYMP	1.35	1.47E-02	0.0371
MAGOH	1.35	7.18E-04	0.0065
ABCB7	1.35	9.26E-04	0.0075
CLPP	1.35	9.03E-04	0.0074
ENST000003017881	1.35	9.41E-04	0.0075
NCEH1	1.35	6.23E-03	0.0212
RFC1	1.35	1.09E-03	0.0080
RARS	1.35	9.59E-04	0.0076
TDG	1.35	2.44E-03	0.0123
ASRGL1	1.35	2.85E-03	0.0134
MCUR1	1.34	8.08E-04	0.0070
ENST000002805570	1.34	4.03E-04	0.0051
USP37	1.34	4.21E-04	0.0052
ENST000002529841	1.34	1.36E-02	0.0351
ENST000003286490	1.34	1.52E-03	0.0095
NUDCD2	1.34	4.82E-04	0.0055
SRI	1.34	4.10E-04	0.0051
MICAL3	1.34	3.94E-03	0.0160
DDX47	1.34	4.51E-04	0.0054
ENST000000130700	1.34	5.66E-04	0.0059
SNHG12	1.34	1.44E-03	0.0092
ENST000002648930	1.34	6.08E-04	0.0061
DDX39B	1.34	6.00E-04	0.0060
TCEA1	1.34	4.74E-04	0.0055
MMS22L	1.34	4.71E-03	0.0178
NAA15	1.34	6.07E-04	0.0061
PARL	1.34	5.07E-04	0.0056
KSR1	1.34	1.29E-03	0.0087
MBOAT7	1.34	7.99E-03	0.0247
AIFM1	1.34	5.04E-04	0.0056
WDR74	1.34	1.97E-03	0.0108
ENST000003260050	1.34	1.21E-03	0.0084
QPCTL	1.34	3.04E-03	0.0138
PSMG1	1.33	6.60E-04	0.0063
PABPN1	1.33	4.54E-04	0.0054
RCL1	1.33	6.65E-04	0.0063
AKR1A1	1.33	2.86E-03	0.0134
NDUFA2	1.33	9.92E-04	0.0077
PMVK	1.33	1.28E-03	0.0086
AURKAIP1	1.33	7.29E-04	0.0066
THNSL1	1.33	1.49E-03	0.0094
SEC13	1.33	2.07E-03	0.0112

UBL7-AS1	1.33	1.11E-03	0.0081
PSMC2	1.33	6.35E-04	0.0062
NDUFS5	1.33	4.94E-04	0.0056
EZR	1.33	9.00E-04	0.0074
AP003084.1	1.33	1.22E-03	0.0084
CISD1	1.33	6.37E-04	0.0062
NOL10	1.33	1.25E-03	0.0085
C6orf52	1.33	5.46E-03	0.0195
NUDT4P2	1.33	1.58E-03	0.0097
SLC25A13	1.33	4.54E-03	0.0175
POLR2F	1.32	7.31E-04	0.0066
BLVRB	1.32	9.61E-04	0.0076
DENND1A	1.32	7.88E-04	0.0069
MRPL58	1.32	6.31E-04	0.0062
ENST000003393740	1.32	3.01E-03	0.0137
SRRT	1.32	1.51E-03	0.0094
DBF4B	1.32	5.60E-03	0.0198
WDR36	1.32	1.21E-03	0.0084
SLC46A1	1.32	7.09E-03	0.0230
MTRF1L	1.32	9.56E-04	0.0076
IFITM3	1.32	1.46E-02	0.0370
ARID3A	1.32	1.96E-03	0.0108
AL591845.1	1.32	7.73E-03	0.0242
ATP5IF1	1.32	7.97E-04	0.0069
ALAS1	1.32	6.71E-04	0.0064
CHID1	1.32	1.27E-02	0.0334
ENST000002459320	1.32	1.02E-03	0.0078
AC073896.1	1.32	2.90E-03	0.0135
MRPL10	1.32	1.04E-03	0.0078
DDX50	1.32	5.10E-04	0.0056
TMEM109	1.32	1.79E-03	0.0103
ACLY	1.32	9.01E-04	0.0074
CRLS1	1.32	6.30E-04	0.0062
TSFM	1.32	2.53E-03	0.0125
SLA	1.31	3.74E-03	0.0155
ENST000003129702	1.31	2.06E-02	0.0472
ACSL4	1.31	2.71E-03	0.0130
GAS8	1.31	2.95E-03	0.0136
RNASEH1-AS1	1.31	4.35E-03	0.0170
ENST000003189743	1.31	3.19E-03	0.0141
AL022067.1	1.31	1.93E-02	0.0450
NDUFAF2	1.31	9.94E-04	0.0077
MTLN	1.31	3.45E-03	0.0148
GNA11	1.31	3.41E-03	0.0147
TCEAL3	1.31	3.18E-03	0.0141
SNRPD2	1.31	1.64E-03	0.0099
AC099548.2	1.31	5.30E-04	0.0058
HNRNPL	1.31	4.97E-04	0.0056
TBRG4	1.31	1.09E-03	0.0080
RNF121	1.31	1.27E-03	0.0086

RPP30	1.31	2.65E-03	0.0128
LAS1L	1.31	2.11E-03	0.0113
DPY19L2P2	1.31	4.62E-03	0.0177
ENST000003213311	1.31	6.01E-04	0.0060
SEM1	1.31	6.25E-04	0.0062
LSM6	1.31	6.90E-04	0.0064
IZUMO4	1.31	3.77E-03	0.0156
BEND3	1.31	3.20E-03	0.0142
CLDN12	1.31	8.95E-04	0.0073
ERO1A	1.30	5.35E-04	0.0058
BMS1	1.30	7.13E-04	0.0065
ENST000002646700	1.30	1.02E-03	0.0078
FBXL6	1.30	8.25E-03	0.0253
LACTB2	1.30	7.57E-04	0.0067
WDR83	1.30	5.08E-03	0.0186
RAD23B	1.30	2.36E-03	0.0121
STK32C	1.30	6.43E-03	0.0216
MCMBP	1.30	6.70E-04	0.0064
ZMIZ1	1.30	5.79E-03	0.0202
NFKBIE	1.30	8.71E-04	0.0072
PTRH2	1.30	1.14E-03	0.0082
CD99	1.30	4.61E-04	0.0054
APP	1.30	2.55E-03	0.0126
HS6ST1	1.30	4.01E-03	0.0162
PPCDC	1.30	8.29E-03	0.0253
GUK1	1.30	4.76E-04	0.0055
MREG	1.30	1.26E-03	0.0086
ZNF282	1.30	4.16E-03	0.0165
HIST1H2AK	1.30	7.73E-03	0.0242
GTF2A2	1.30	2.02E-03	0.0110
ENST000003025505	1.30	5.03E-04	0.0056
RNF187	1.30	2.08E-03	0.0112
ENST000002603241	1.30	1.61E-02	0.0395
AIG1	1.29	1.37E-03	0.0089
MTHFD1	1.29	1.05E-03	0.0079
RAB27A	1.29	3.90E-03	0.0159
CMAHP	1.29	6.37E-03	0.0214
CISD3	1.29	3.13E-03	0.0140
FECH	1.29	1.23E-03	0.0085
AC006449.6	1.29	6.18E-03	0.0210
AC006449.9	1.29	6.18E-03	0.0210
ENST000003591910	1.29	7.04E-03	0.0229
FHOD1	1.29	4.71E-03	0.0178
PARP1	1.29	4.89E-04	0.0055
ENST000002623061	1.29	3.72E-03	0.0155
TBL3	1.29	2.79E-03	0.0133
CNTROB	1.29	1.50E-03	0.0094
HSPA14	1.29	1.27E-03	0.0086
CCDC24	1.29	8.97E-03	0.0266
AIFM2	1.29	1.86E-02	0.0439

ENST000002814560	1.29	5.40E-03	0.0194
MRPL44	1.29	4.89E-04	0.0055
AC004477.1	1.29	4.66E-03	0.0177
SNHG19	1.29	2.19E-02	0.0492
UBFD1	1.29	8.03E-04	0.0070
SMG5	1.29	2.14E-03	0.0114
JAKMIP1	1.29	3.73E-03	0.0155
HMGN1	1.29	4.91E-04	0.0056
DPM1	1.29	1.95E-03	0.0108
ENST000002623190	1.29	1.76E-02	0.0421
AL138781.1	1.29	8.65E-03	0.0260
NARS2	1.29	9.75E-03	0.0281
MRPL54	1.29	8.62E-04	0.0072
C16orf91	1.29	2.89E-03	0.0134
PSMA5	1.29	5.72E-04	0.0059
ENST000003160773	1.28	1.23E-02	0.0329
DNAJC25	1.28	2.93E-03	0.0135
ENST000003164230	1.28	7.21E-03	0.0231
GNPAT	1.28	6.77E-04	0.0064
NAP1L4P1	1.28	4.12E-03	0.0164
ENST000003162923	1.28	4.14E-03	0.0165
UHRF1BP1L	1.28	1.78E-03	0.0103
AC002116.2	1.28	6.46E-03	0.0217
DLG3	1.28	1.51E-03	0.0094
H3F3B	1.28	8.59E-03	0.0259
PSMD2	1.28	7.87E-04	0.0069
SERPINB8	1.28	1.26E-03	0.0086
PPP1R3B	1.28	8.96E-03	0.0266
FANCM	1.28	5.46E-04	0.0058
BICDL1	1.28	1.12E-02	0.0309
KHK	1.28	3.17E-03	0.0141
UBA2	1.28	5.53E-04	0.0058
CSNK2A1	1.28	4.72E-04	0.0055
CCR4	1.28	8.49E-03	0.0257
CNP	1.28	1.50E-03	0.0094
IMMT	1.28	6.71E-04	0.0064
WDR3	1.28	1.97E-03	0.0108
CNTLN	1.28	7.07E-03	0.0229
PFN2	1.28	1.77E-02	0.0422
ABCF1	1.28	1.33E-03	0.0088
MRPS22	1.27	7.39E-04	0.0066
KNOP1	1.27	2.13E-03	0.0113
WDR46	1.27	9.88E-04	0.0077
LAMTOR5	1.27	5.48E-04	0.0058
AC135977.1	1.27	1.52E-02	0.0379
SLC25A33	1.27	1.68E-03	0.0100
ARMCX3	1.27	1.74E-03	0.0102
SRP72	1.27	7.82E-04	0.0069
RPSAP58	1.27	1.95E-02	0.0453
TNFRSF14-AS1	1.27	3.35E-03	0.0145

HDDC3	1.27	1.64E-03	0.0099
FUNDC2	1.27	1.21E-03	0.0084
EIF4G3	1.27	1.31E-03	0.0087
RPL29	1.27	2.52E-03	0.0125
HSPA8	1.27	5.52E-04	0.0058
ARPC2	1.27	9.88E-04	0.0077
PCTP	1.27	8.92E-03	0.0265
TIMM10B	1.27	2.05E-02	0.0469
LYST	1.27	3.83E-03	0.0158
ITGA1	1.27	1.40E-03	0.0091
ENST000002649320	1.26	2.97E-03	0.0136
DDX18	1.26	8.33E-04	0.0071
FAM161A	1.26	6.17E-03	0.0210
VARS2	1.26	3.41E-03	0.0147
PRELP	1.26	3.61E-03	0.0152
SMN2	1.26	1.15E-03	0.0082
TXNL1	1.26	2.20E-03	0.0116
LRP11	1.26	2.61E-03	0.0127
NUDT2	1.26	4.68E-03	0.0178
PNO1	1.26	1.17E-03	0.0083
CEP83	1.26	1.72E-03	0.0101
COMMD3-BMI1	1.26	1.21E-03	0.0084
SUZ12	1.26	5.10E-04	0.0056
PPP1R7	1.26	5.45E-04	0.0058
DAD1	1.26	7.93E-04	0.0069
RNF207	1.26	4.43E-03	0.0172
RPL6	1.26	2.47E-03	0.0124
P2RY11	1.26	5.81E-03	0.0203
FDXR	1.26	5.82E-03	0.0203
CYB5D1	1.26	4.52E-03	0.0174
EPRS	1.26	7.15E-04	0.0065
ENST000002298660	1.26	2.69E-03	0.0130
TARS	1.26	3.82E-03	0.0157
AC141586.1	1.26	2.00E-02	0.0461
PRDX2	1.26	8.64E-04	0.0072
DIMT1	1.26	3.14E-03	0.0140
CD151	1.26	8.07E-03	0.0249
ZNF620	1.26	1.77E-02	0.0423
DPH2	1.25	4.31E-03	0.0169
TTC4	1.25	1.75E-03	0.0102
SNHG8	1.25	5.93E-04	0.0060
ENST000002364121	1.25	3.49E-03	0.0149
PHF19	1.25	1.50E-02	0.0376
TTC38	1.25	8.48E-03	0.0257
MAIP1	1.25	1.01E-03	0.0078
CETN2	1.25	3.93E-03	0.0160
RAB11FIP1	1.25	1.50E-02	0.0376
HNRNPA1	1.25	1.09E-03	0.0080
CGAS	1.25	1.07E-03	0.0079
LAMTOR2	1.25	8.17E-04	0.0070

MYO19	1.25	1.78E-03	0.0103
FHL2	1.25	1.45E-02	0.0367
NDUFB4	1.25	6.07E-04	0.0061
MMAB	1.25	2.08E-03	0.0112
ENST000002701420	1.25	1.84E-03	0.0105
ISOC1	1.25	1.34E-03	0.0088
PSMA7	1.25	1.77E-03	0.0102
NAT10	1.25	1.22E-03	0.0084
SUMO2	1.25	6.14E-04	0.0061
DHX15	1.25	5.63E-04	0.0059
TIMMDC1	1.25	1.26E-03	0.0086
MXI1	1.24	2.84E-03	0.0134
EXOC3-AS1	1.24	1.06E-02	0.0296
MBD2	1.24	1.15E-03	0.0082
ENST000003600010	1.24	2.60E-03	0.0127
NAE1	1.24	1.70E-03	0.0101
ZNF726	1.24	1.19E-03	0.0083
ATG101	1.24	6.71E-04	0.0064
AC024940.5	1.24	9.60E-03	0.0278
PRELID3A	1.24	1.03E-02	0.0291
CFAP20	1.24	6.16E-04	0.0061
RPS19BP1	1.24	2.16E-03	0.0114
P4HB	1.24	1.84E-03	0.0105
DNAJA3	1.24	1.44E-03	0.0092
PLGRKT	1.24	1.04E-02	0.0294
UQCR10	1.24	6.99E-04	0.0065
FAM27E3	1.24	7.66E-03	0.0241
HNRNPF	1.24	8.55E-04	0.0072
HIST1H4E	1.24	1.58E-03	0.0097
POLE	1.24	5.68E-04	0.0059
PPP2R3B	1.24	3.49E-03	0.0149
LARS2	1.24	1.74E-03	0.0102
WDR75	1.23	1.51E-03	0.0094
GTF2F2	1.23	8.71E-04	0.0072
CCDC113	1.23	5.08E-03	0.0187
RABL3	1.23	7.40E-04	0.0066
TMEM67	1.23	3.33E-03	0.0145
C17orf51	1.23	1.22E-03	0.0084
MBNL1-AS1	1.23	8.25E-03	0.0253
ZNF850	1.23	2.06E-03	0.0111
ENST000002653511	1.23	7.56E-04	0.0067
QDPR	1.23	3.35E-03	0.0146
URB2	1.23	2.49E-03	0.0124
ENST000003141000	1.23	7.90E-03	0.0245
RBM8A	1.23	2.35E-03	0.0121
MRPS9	1.23	3.11E-03	0.0140
ENST000002304310	1.23	2.96E-03	0.0136
SEC14L2	1.23	1.02E-03	0.0078
SMARCB1	1.23	3.39E-03	0.0147
TAP1	1.23	1.63E-03	0.0099

ENST000002252981	1.23	1.33E-03	0.0088
DNMT1	1.22	1.33E-03	0.0088
TMEM147	1.22	1.74E-03	0.0102
ENST000002311981	1.22	8.52E-04	0.0072
CCDC124	1.22	7.44E-03	0.0236
WRB	1.22	1.31E-03	0.0087
ENST000002336300	1.22	1.21E-02	0.0324
ADRM1	1.22	2.05E-03	0.0111
DOCK7	1.22	1.88E-03	0.0106
PUSL1	1.22	1.93E-02	0.0450
C1orf35	1.22	1.50E-03	0.0094
PSMB4	1.22	1.45E-03	0.0092
EXTL2	1.22	8.89E-03	0.0265
VDAC3	1.22	1.06E-03	0.0079
ENST000003228970	1.22	4.93E-03	0.0183
EVA1C	1.22	5.83E-03	0.0203
FOXRED1	1.22	2.81E-03	0.0133
KCTD17	1.22	5.90E-03	0.0204
CALU	1.22	9.70E-04	0.0076
HSBP1	1.22	9.74E-04	0.0076
TMA7	1.22	1.73E-03	0.0101
AGPAT5	1.22	4.96E-03	0.0184
GALNT1	1.22	8.18E-04	0.0070
ENST000003378590	1.22	7.06E-04	0.0065
AC025423.2	1.22	7.75E-03	0.0242
MALSU1	1.22	1.16E-03	0.0082
FKBP1A	1.21	4.44E-03	0.0172
IMP4	1.21	1.03E-03	0.0078
CKLF	1.21	1.25E-03	0.0086
PLIN2	1.21	2.99E-03	0.0137
ENST000002788860	1.21	1.79E-02	0.0427
ENST000002623750	1.21	1.42E-03	0.0091
RPS2P5	1.21	3.22E-03	0.0142
NTMT1	1.21	1.54E-03	0.0095
CDC34	1.21	2.10E-03	0.0113
BMI1	1.21	2.91E-03	0.0135
NXT2	1.21	6.11E-03	0.0209
PFKL	1.21	1.49E-03	0.0094
PLIN3	1.21	2.03E-03	0.0110
NDUFA4	1.21	7.18E-04	0.0065
TMEM263	1.21	1.58E-03	0.0097
IARS2	1.21	6.61E-04	0.0063
MRPL9	1.21	1.26E-03	0.0086
ISOC2	1.21	2.95E-03	0.0136
GLTP	1.21	8.62E-04	0.0072
TXN2	1.20	1.86E-03	0.0105
NAA50	1.20	1.49E-03	0.0094
AAMP	1.20	1.29E-03	0.0087
UTP6	1.20	6.28E-04	0.0062
NIP7	1.20	1.27E-03	0.0086

ZNF219	1.20	1.30E-02	0.0340
KYAT3	1.20	8.84E-04	0.0073
RPP25L	1.20	4.26E-03	0.0168
SAPCD1	1.20	8.61E-03	0.0259
NENF	1.20	3.83E-03	0.0158
UGDH	1.20	3.97E-03	0.0161
PDP2	1.20	7.56E-04	0.0067
METTL5	1.20	1.23E-03	0.0085
COX16	1.20	1.03E-03	0.0078
ENST000003060520	1.20	7.54E-03	0.0238
BCL6	1.20	1.18E-02	0.0319
CCSAP	1.20	5.57E-03	0.0197
ENST000003405780	1.20	1.80E-02	0.0427
ACADM	1.20	9.43E-04	0.0075
CCDC15	1.20	1.24E-02	0.0331
ENST000003343790	1.20	8.33E-04	0.0071
DCTD	1.20	1.40E-03	0.0091
MRPS27	1.20	9.54E-04	0.0076
AL512791.2	1.20	2.61E-03	0.0127
TAF13	1.20	1.31E-03	0.0087
MMADHC	1.20	7.37E-04	0.0066
TIAM1	1.19	1.94E-03	0.0107
ARF6	1.19	1.10E-03	0.0080
AC072061.1	1.19	5.68E-03	0.0200
HSD17B8	1.19	1.26E-02	0.0334
IPPK	1.19	9.55E-04	0.0076
POLR3K	1.19	4.15E-03	0.0165
TELO2	1.19	5.37E-03	0.0193
CWC27	1.19	2.15E-03	0.0114
CES3	1.19	4.19E-03	0.0166
ZC3HC1	1.19	5.39E-03	0.0193
ENST000002403330	1.19	1.39E-02	0.0357
NSMCE1	1.19	1.58E-03	0.0097
ENST000002664810	1.19	5.06E-03	0.0186
NDUFAF1	1.19	8.16E-04	0.0070
ENST000003131460	1.19	8.95E-03	0.0266
DNPEP	1.19	4.15E-03	0.0165
DDX41	1.19	9.02E-04	0.0074
RNF181	1.19	1.37E-02	0.0353
RPS17	1.19	7.36E-04	0.0066
ENST000003605791	1.19	1.26E-03	0.0086
ACYP1	1.19	3.28E-03	0.0144
NUDT5	1.19	1.27E-03	0.0086
INPP1	1.19	1.42E-03	0.0091
COPS3	1.19	8.74E-04	0.0072
BMP1	1.19	1.27E-02	0.0336
ENST000002803302	1.19	3.14E-03	0.0140
HAUS2	1.19	7.28E-04	0.0066
DDX49	1.19	1.07E-03	0.0079
RNF26	1.19	2.75E-03	0.0131

NCOA7	1.19	3.12E-03	0.0140
SF3A3	1.19	1.13E-03	0.0081
MT-ATP8	1.19	5.37E-03	0.0193
ICMT	1.19	1.19E-03	0.0083
ENST000003315521	1.18	1.70E-02	0.0410
KRR1	1.18	9.41E-04	0.0075
CCND3	1.18	1.68E-03	0.0100
ENST000001682160	1.18	3.47E-03	0.0149
KIF5C	1.18	3.26E-03	0.0143
GNB1	1.18	6.52E-04	0.0063
TXLNA	1.18	1.30E-03	0.0087
ETV6	1.18	1.03E-03	0.0078
NDUFA8	1.18	2.15E-03	0.0114
MRPL49	1.18	4.04E-03	0.0162
AC103706.1	1.18	1.46E-02	0.0368
ATAT1	1.18	7.69E-03	0.0241
GABPB1	1.18	1.19E-03	0.0083
TCOF1	1.18	2.42E-03	0.0123
AKR1B1	1.18	1.55E-03	0.0096
DDX54	1.18	1.13E-03	0.0081
ENST000003302440	1.18	7.97E-04	0.0069
NUP160	1.18	1.74E-03	0.0102
BTF3L4P2	1.18	1.13E-02	0.0310
ZNF146	1.18	1.08E-03	0.0080
SFT2D1	1.18	1.08E-03	0.0080
GOLGA2P10	1.18	2.13E-02	0.0483
EXD2	1.18	1.83E-03	0.0104
TCAF2	1.18	8.41E-04	0.0071
BABAM1	1.18	1.22E-03	0.0084
MTERF3	1.18	9.52E-04	0.0076
DDX1	1.17	7.60E-04	0.0067
ATP5PO	1.17	8.54E-04	0.0072
POLR1C	1.17	2.30E-03	0.0119
POMT2	1.17	3.20E-03	0.0142
FSD1	1.17	1.05E-02	0.0294
LRRC40	1.17	2.34E-03	0.0120
POLR2J	1.17	2.54E-03	0.0125
TBL2	1.17	1.19E-03	0.0083
VASP	1.17	5.07E-03	0.0186
ENST000002658661	1.17	1.44E-02	0.0366
MRPL24	1.17	4.82E-03	0.0181
FTSJ1	1.17	1.16E-03	0.0082
NOP14	1.17	1.60E-02	0.0394
SNAPC4	1.17	7.15E-03	0.0231
SLC25A22	1.17	1.92E-02	0.0448
ARMCX2	1.17	3.59E-03	0.0151
NELFE	1.17	1.38E-03	0.0090
RNASEH1	1.17	5.23E-03	0.0190
TRMT10A	1.17	7.45E-03	0.0236
RADX	1.17	1.45E-03	0.0092

NDUFS3	1.17	1.42E-03	0.0091
PSMC1P1	1.17	2.67E-03	0.0129
UCHL5	1.17	1.20E-03	0.0084
ENST000002950872	1.17	1.92E-03	0.0107
CDK16	1.17	2.26E-03	0.0118
DESI1	1.16	1.22E-03	0.0084
TIMM9	1.16	2.69E-03	0.0130
ENST000002903490	1.16	1.44E-03	0.0092
TRAF2	1.16	8.65E-04	0.0072
SRSF9	1.16	1.13E-03	0.0081
LARP7	1.16	1.52E-03	0.0095
PDK3	1.16	1.74E-03	0.0102
ENST000002605990	1.16	4.36E-03	0.0170
RNPS1	1.16	1.16E-03	0.0082
FAM86C2P	1.16	2.12E-02	0.0481
EMG1	1.16	2.54E-03	0.0125
BUD13	1.16	9.34E-04	0.0075
POLR2I	1.16	4.57E-03	0.0175
MAP3K13	1.16	2.53E-03	0.0125
RABGGTB	1.16	5.43E-03	0.0194
NCDN	1.16	6.33E-03	0.0214
C19orf53	1.16	1.67E-03	0.0100
ENST000003077410	1.16	1.75E-02	0.0418
TACO1	1.16	1.73E-03	0.0101
POU2AF1	1.16	3.86E-03	0.0158
C16orf87	1.16	1.12E-03	0.0081
RAB34	1.16	9.04E-03	0.0267
OXSR1	1.15	7.83E-04	0.0069
AL132780.3	1.15	5.10E-03	0.0187
RPL23AP53	1.15	1.40E-02	0.0358
UBE2N	1.15	1.25E-03	0.0086
TTI2	1.15	2.01E-03	0.0110
SOCS7	1.15	1.04E-03	0.0078
ENST000003078922	1.15	1.07E-02	0.0299
RELB	1.15	1.72E-03	0.0101
PIGU	1.15	1.37E-03	0.0089
GFI1	1.15	8.64E-03	0.0260
DCAF13	1.15	1.11E-03	0.0081
SLC25A44	1.15	5.62E-03	0.0198
E2F6	1.15	2.15E-03	0.0114
MRPS24	1.15	9.97E-04	0.0077
EBPL	1.15	1.53E-02	0.0380
CCDC25	1.15	8.42E-04	0.0071
CCDC88A	1.15	5.03E-03	0.0185
CYREN	1.15	1.95E-03	0.0108
SERPINH1	1.15	5.79E-03	0.0202
BLOC1S2	1.15	1.71E-03	0.0101
API5	1.15	9.97E-04	0.0077
MYCBP	1.15	2.95E-03	0.0136
ENST000003213371	1.15	6.85E-03	0.0225

PSMD4	1.15	9.27E-04	0.0075
MDC1	1.15	5.68E-03	0.0200
SP2-AS1	1.15	1.31E-02	0.0342
RNPEP	1.15	6.58E-03	0.0220
NFS1	1.15	1.30E-03	0.0087
NEDD1	1.15	1.02E-03	0.0078
VGLL4	1.15	3.98E-03	0.0161
NFYB	1.15	2.15E-03	0.0114
PNPO	1.15	1.95E-03	0.0107
HCAR1	1.15	6.76E-03	0.0223
HBS1L	1.15	1.04E-03	0.0079
DHX37	1.15	1.35E-03	0.0089
MFN2	1.15	1.17E-03	0.0083
AZIN1	1.14	8.82E-04	0.0073
TMEM70	1.14	3.87E-03	0.0159
HMGCL	1.14	4.62E-03	0.0176
AHCYL1	1.14	1.24E-03	0.0085
PRIM2	1.14	3.00E-03	0.0137
PSMB9	1.14	1.60E-03	0.0098
DNAJA2	1.14	1.09E-03	0.0080
MRGBP	1.14	9.17E-04	0.0074
PAM16	1.14	2.91E-03	0.0135
NUP88	1.14	9.56E-04	0.0076
ENST000003594660	1.14	1.02E-02	0.0290
CHMP5	1.14	1.20E-03	0.0084
UTP15	1.14	3.29E-03	0.0144
ELP5	1.14	1.91E-03	0.0106
UNG	1.14	1.56E-03	0.0096
MSANTD3	1.14	2.08E-02	0.0474
UEVLD	1.14	4.66E-03	0.0177
RPS6KB2	1.14	8.67E-04	0.0072
PCMT1	1.14	1.09E-03	0.0080
ZNF827	1.14	5.11E-03	0.0187
VCP	1.14	1.12E-03	0.0081
CCHCR1	1.14	2.44E-03	0.0123
ZNF584	1.13	4.40E-03	0.0171
C1orf43	1.13	7.90E-04	0.0069
PHF13	1.13	2.71E-03	0.0130
LINC00963	1.13	7.05E-03	0.0229
SRSF3	1.13	9.58E-04	0.0076
XXYLT1	1.13	2.05E-02	0.0470
EML2	1.13	8.10E-04	0.0070
HAUS5	1.13	2.14E-03	0.0114
KCTD5	1.13	4.64E-03	0.0177
HNRNPLL	1.13	1.98E-03	0.0109
LRTOMT	1.13	1.62E-02	0.0396
RAD1	1.13	1.38E-03	0.0090
CDC26	1.13	2.03E-03	0.0110
NDUFS4	1.13	1.19E-03	0.0083
NIF3L1	1.13	1.35E-03	0.0089

TWNK	1.13	4.31E-03	0.0169
MCU	1.13	8.44E-03	0.0256
RAB5IF	1.13	9.89E-04	0.0077
ARMC6	1.13	5.79E-03	0.0202
CCDC117	1.13	8.97E-04	0.0073
PIM2	1.13	2.28E-03	0.0119
ENST000002267250	1.13	9.29E-03	0.0272
PER1	1.13	7.74E-03	0.0242
CCNC	1.13	9.51E-04	0.0076
SNORD3A	1.13	3.50E-03	0.0149
NEDD8	1.13	1.90E-03	0.0106
ZNF724	1.13	9.82E-04	0.0077
ENY2	1.13	2.26E-03	0.0118
HMGXB4	1.13	6.10E-03	0.0209
ENST000002261050	1.13	5.30E-03	0.0191
PRDX5	1.12	1.76E-03	0.0102
CHD1L	1.12	1.30E-03	0.0087
CEP295	1.12	4.55E-03	0.0175
ARL1	1.12	1.27E-03	0.0086
AC105137.2	1.12	8.41E-03	0.0256
NFE2L1	1.12	2.48E-03	0.0124
ENST000002777460	1.12	9.57E-03	0.0277
CANX	1.12	3.29E-03	0.0144
AMIGO2	1.12	8.44E-03	0.0256
IER5	1.12	1.10E-03	0.0080
GUCD1	1.12	9.29E-04	0.0075
NDUFA1	1.12	1.02E-03	0.0078
CHML	1.12	3.35E-03	0.0146
CDC27	1.12	8.41E-04	0.0071
LRRC58	1.12	1.08E-03	0.0080
RFT1	1.12	5.14E-03	0.0188
NDUFA9	1.12	2.03E-03	0.0110
STX1A	1.12	5.09E-03	0.0187
SPINT2	1.12	1.43E-02	0.0365
HYAL3	1.12	1.26E-02	0.0333
SNF8	1.12	2.79E-03	0.0132
SYNCRIP	1.12	1.31E-03	0.0087
CEP128	1.12	1.74E-03	0.0102
PAWR	1.12	1.37E-03	0.0089
HEXIM2	1.12	1.61E-02	0.0395
ENST000003252120	1.12	1.74E-03	0.0102
GPANK1	1.12	2.29E-03	0.0119
IMPAD1	1.11	1.14E-03	0.0082
ENST000003371471	1.11	6.63E-03	0.0221
DSTN	1.11	1.18E-03	0.0083
EIF3D	1.11	1.02E-03	0.0078
RPL4	1.11	1.70E-03	0.0101
ENST000003320350	1.11	9.66E-03	0.0279
CHCHD3	1.11	1.34E-03	0.0088
EIF4A1	1.11	1.39E-03	0.0090

ENST000003171680	1.11	2.92E-03	0.0135
SREBF2	1.11	1.78E-03	0.0103
TMSB10	1.11	9.35E-04	0.0075
C12orf75	1.11	1.42E-03	0.0091
HNRNPM	1.11	2.47E-03	0.0124
NUDT4	1.11	1.63E-03	0.0099
PRADC1	1.11	4.23E-03	0.0167
WDR4	1.11	2.62E-03	0.0128
TMEM64	1.11	1.73E-03	0.0102
CAPZA1	1.11	1.04E-03	0.0078
PROS1	1.11	6.25E-03	0.0212
REEP4	1.11	9.47E-03	0.0275
DERA	1.11	2.31E-03	0.0119
HAUS4	1.11	1.74E-03	0.0102
ZDHHC16	1.11	2.06E-03	0.0111
TOP3A	1.11	2.10E-03	0.0112
MYDGF	1.11	6.83E-03	0.0225
NONO	1.11	1.37E-03	0.0089
ATP5PB	1.11	1.08E-03	0.0080
CDC23	1.11	1.12E-03	0.0081
C12orf43	1.10	1.27E-03	0.0086
ENST000003348161	1.10	1.45E-02	0.0367
RBM38	1.10	2.69E-03	0.0130
LARP4	1.10	2.95E-03	0.0136
ENST000003613830	1.10	9.45E-04	0.0076
ENST000003010720	1.10	2.57E-03	0.0126
GFM2	1.10	9.89E-04	0.0077
PPP2R3C	1.10	1.65E-03	0.0099
IFT140	1.10	3.31E-03	0.0144
PPIL3	1.10	1.67E-03	0.0100
RAE1	1.10	9.51E-04	0.0076
ZBTB21	1.10	3.34E-03	0.0145
TDP1	1.10	1.28E-03	0.0086
UTP14A	1.10	1.61E-03	0.0098
CMAS	1.10	1.13E-03	0.0081
TOMM20	1.10	1.77E-03	0.0102
MAP2K1	1.10	3.54E-03	0.0150
NAGA	1.10	2.91E-03	0.0135
IRGQ	1.10	1.11E-03	0.0081
CISD2	1.10	2.13E-03	0.0114
GNL1	1.10	3.53E-03	0.0150
ENST000002369800	1.10	7.98E-03	0.0247
WDR41	1.10	1.85E-03	0.0105
SEC23B	1.10	2.49E-03	0.0124
OTUD6B	1.10	3.67E-03	0.0153
ZNF511	1.10	2.18E-02	0.0491
RPAP3	1.10	1.83E-03	0.0104
EIF4E	1.10	1.62E-03	0.0098
GDI2	1.10	8.06E-03	0.0249
ENST000003225350	1.10	9.33E-04	0.0075

COLGALT1	1.10	1.39E-03	0.0090
UQCRC2	1.10	1.10E-03	0.0080
RIOK1	1.10	1.98E-03	0.0109
HACD1	1.10	1.34E-02	0.0348
RHOA	1.10	9.74E-04	0.0076
ENST000003034360	1.09	5.22E-03	0.0190
SYNGR2	1.09	2.91E-03	0.0135
ANKLE2	1.09	9.48E-04	0.0076
OPA1	1.09	1.76E-03	0.0102
HMGCR	1.09	1.13E-03	0.0081
ACAA2	1.09	1.07E-03	0.0080
HSPA13	1.09	5.75E-03	0.0201
CAMSAP1	1.09	1.06E-02	0.0297
AL355388.2	1.09	6.26E-03	0.0212
NDUFA13	1.09	1.88E-03	0.0106
DCPS	1.09	1.59E-03	0.0097
CUEDC2	1.09	1.61E-03	0.0098
DLEU2	1.09	1.18E-03	0.0083
LTV1	1.09	1.54E-03	0.0095
CLTA	1.09	1.10E-03	0.0080
FAM104B	1.09	2.54E-03	0.0125
RAB33A	1.09	1.88E-03	0.0106
CMTM6	1.09	1.13E-03	0.0081
PIH1D1	1.09	1.76E-03	0.0102
PSMC6	1.09	1.95E-03	0.0107
FKBP3	1.08	1.05E-03	0.0079
NDUFB7	1.08	1.85E-03	0.0105
D2HGDH	1.08	5.60E-03	0.0198
PTPA	1.08	9.13E-03	0.0269
AC107871.1	1.08	1.64E-02	0.0401
POLR1B	1.08	1.35E-03	0.0089
SWI5	1.08	3.68E-03	0.0154
CCDC167	1.08	4.89E-03	0.0182
MTIF2	1.08	1.50E-03	0.0094
SIGMAR1	1.08	1.43E-03	0.0091
UBA5	1.08	6.57E-03	0.0219
ENST000002159171	1.08	2.16E-03	0.0114
CREG1	1.08	3.94E-03	0.0160
RBM3	1.08	3.66E-03	0.0153
RPL37A	1.08	1.87E-03	0.0105
PER2	1.08	2.62E-03	0.0128
ABCC4	1.08	1.80E-03	0.0103
DPY19L1	1.08	7.64E-03	0.0240
EAF2	1.08	4.00E-03	0.0161
SNRNP70	1.08	1.81E-03	0.0104
PAXIP1	1.08	1.34E-03	0.0088
FADD	1.08	1.78E-03	0.0103
FASTKD2	1.08	1.67E-03	0.0100
ENST000003360530	1.08	1.30E-03	0.0087
PRMT3	1.08	8.79E-03	0.0263

NTAN1	1.08	2.35E-03	0.0121
TPRKB	1.08	2.69E-03	0.0130
XPOT	1.08	3.46E-03	0.0148
URB1	1.08	2.37E-03	0.0121
CAND1	1.08	1.17E-03	0.0083
BAP1	1.07	1.70E-03	0.0101
TPM3	1.07	1.15E-03	0.0082
DNM1L	1.07	9.81E-04	0.0077
NT5C	1.07	1.13E-02	0.0311
SNU13	1.07	1.23E-03	0.0085
ACSF2	1.07	4.87E-03	0.0182
ME2	1.07	1.65E-03	0.0099
POLR2L	1.07	8.77E-03	0.0262
ENST000002642540	1.07	2.90E-03	0.0135
HNRNPK	1.07	9.97E-04	0.0077
MTPAP	1.07	4.34E-03	0.0170
ENST000003209854	1.07	6.38E-03	0.0215
THUMPD2	1.07	1.08E-03	0.0080
PCNP	1.07	9.71E-04	0.0076
ENST000002161211	1.07	2.26E-03	0.0118
PCGF6	1.07	3.83E-03	0.0157
CBX5	1.07	4.28E-03	0.0168
IAH1	1.07	1.23E-03	0.0085
USP13	1.07	3.23E-03	0.0142
FGFBP3	1.07	1.86E-02	0.0439
PPM1F	1.07	2.86E-03	0.0134
NLRX1	1.07	8.37E-03	0.0255
GTSF1	1.07	1.99E-02	0.0459
TTF2	1.07	1.16E-03	0.0082
SMC3	1.07	1.24E-03	0.0085
CLTC	1.07	1.35E-03	0.0089
RELT	1.07	2.18E-03	0.0115
AC009779.2	1.07	1.88E-02	0.0441
GPX4	1.07	1.72E-03	0.0101
UBE2I	1.07	1.05E-03	0.0079
CHMP2A	1.07	1.90E-02	0.0444
ENST000003119431	1.07	3.81E-03	0.0157
NIPA2	1.06	6.25E-03	0.0212
ENST000002457960	1.06	3.10E-03	0.0140
ENST000002623023	1.06	8.55E-03	0.0258
CASP3	1.06	3.96E-03	0.0160
ENST000002168071	1.06	5.73E-03	0.0201
NCBP2	1.06	2.23E-03	0.0117
RITA1	1.06	6.22E-03	0.0211
EXOSC9	1.06	1.32E-03	0.0087
MDM2	1.06	5.88E-03	0.0204
ENST000003018210	1.06	1.63E-02	0.0399
MLLT1	1.06	3.75E-03	0.0156
NFU1	1.06	3.13E-03	0.0140
MRPL46	1.06	1.29E-02	0.0338

MED30	1.06	1.75E-03	0.0102
EMC3-AS1	1.06	3.95E-03	0.0160
ENST000002693730	1.06	9.86E-03	0.0283
RAC1	1.06	1.06E-03	0.0079
TMEM256	1.06	1.47E-02	0.0370
SEC24A	1.06	1.74E-03	0.0102
DLG5	1.06	2.23E-02	0.0499
SCO2	1.06	4.33E-03	0.0169
AC083880.1	1.06	2.21E-02	0.0496
ENST000002161902	1.06	3.95E-03	0.0160
RRP1B	1.06	2.61E-03	0.0127
ENST00000639674	1.06	5.44E-03	0.0194
DUSP1	1.06	1.71E-02	0.0411
MRPS10	1.06	1.55E-03	0.0096
ENST000002686680	1.05	1.49E-03	0.0094
DNAJC2	1.05	9.18E-03	0.0270
DMD	1.05	7.74E-03	0.0242
HNRNPA2B1	1.05	1.98E-03	0.0109
EXOSC5	1.05	2.84E-03	0.0134
RABEPK	1.05	8.85E-03	0.0264
FAHD2A	1.05	1.89E-03	0.0106
CAPZA2	1.05	1.57E-03	0.0097
ARPC4	1.05	1.09E-03	0.0080
BCAP29	1.05	3.02E-03	0.0137
ENST000003181580	1.05	2.66E-03	0.0129
DDB2	1.05	1.46E-03	0.0093
PPIE	1.05	4.88E-03	0.0182
TTC33	1.05	2.02E-03	0.0110
GYS1	1.05	2.18E-03	0.0115
U2AF2	1.05	1.42E-03	0.0091
APOPT1	1.05	2.10E-03	0.0113
RIC8A	1.05	1.12E-03	0.0081
SPATA5	1.05	3.81E-03	0.0157
CAD	1.05	2.42E-03	0.0122
THUMPD3	1.05	3.05E-03	0.0139
TTF1	1.05	1.13E-03	0.0081
SLC19A2	1.05	1.67E-02	0.0406
ACADVL	1.05	3.58E-03	0.0151
TRAF3	1.05	5.73E-03	0.0201
YIF1A	1.05	3.05E-03	0.0139
PBDC1	1.05	2.68E-03	0.0129
OVCA2	1.05	4.76E-03	0.0179
BAK1	1.05	9.79E-03	0.0282
HINT2	1.05	6.02E-03	0.0207
ENST000003418720	1.04	1.09E-02	0.0303
NDUFC1	1.04	1.48E-03	0.0093
ENST000002516070	1.04	3.93E-03	0.0160
NFKBIL1	1.04	9.95E-03	0.0285
ENST000003031451	1.04	2.40E-03	0.0122
MPG	1.04	1.62E-03	0.0098

PSME2	1.04	1.66E-03	0.0099
ENST000002536921	1.04	4.43E-03	0.0172
STK3	1.04	2.38E-03	0.0121
C2CD2	1.04	9.50E-03	0.0276
FDFT1	1.04	2.05E-03	0.0111
EFHD2	1.04	8.16E-03	0.0251
INTS2	1.04	2.46E-03	0.0124
AP3M2	1.04	1.61E-02	0.0395
JMJD6	1.04	1.49E-03	0.0094
ACO2	1.04	3.23E-03	0.0142
IFI27L2	1.04	2.07E-02	0.0474
NTHL1	1.04	1.51E-02	0.0377
ACOX3	1.04	1.80E-03	0.0103
USP39	1.04	2.77E-03	0.0132
ZRANB3	1.04	2.17E-03	0.0115
PTK7	1.04	5.35E-03	0.0192
TIMM22	1.03	1.63E-03	0.0099
SNORD11B	1.03	1.74E-02	0.0416
RRS1	1.03	1.84E-03	0.0105
SART3	1.03	1.22E-03	0.0084
ENST000002660871	1.03	4.79E-03	0.0180
ENST000003409130	1.03	1.42E-03	0.0091
ARPC1A	1.03	6.39E-03	0.0215
LCP1	1.03	8.28E-03	0.0253
C16orf95	1.03	2.08E-02	0.0475
ENST000002766920	1.03	8.62E-03	0.0259
GRB2	1.03	1.17E-03	0.0083
PPP4R2	1.03	1.88E-03	0.0106
SKP2	1.03	7.14E-03	0.0231
NDUFS1	1.03	1.26E-03	0.0086
G6PD	1.03	5.04E-03	0.0186
ENST000002750361	1.03	1.78E-03	0.0103
CSNK1E	1.03	3.12E-03	0.0140
SEH1L	1.03	1.28E-03	0.0086
DHX9	1.03	2.49E-03	0.0124
ENST000003383820	1.03	4.67E-03	0.0178
PHETA2	1.03	5.62E-03	0.0198
MED28	1.03	4.91E-03	0.0183
NR2C2AP	1.03	1.29E-02	0.0339
PANX1	1.03	5.76E-03	0.0202
C4orf3	1.03	1.27E-03	0.0086
AARS2	1.03	3.71E-03	0.0155
ENST000002644330	1.03	1.64E-02	0.0399
ADSL	1.03	3.33E-03	0.0145
USP5	1.03	1.28E-03	0.0086
NUFIP1	1.03	5.01E-03	0.0185
MRPS36	1.02	1.92E-03	0.0107
PGP	1.02	3.45E-03	0.0148
HDDC2	1.02	2.29E-03	0.0119
RNF8	1.02	4.60E-03	0.0176

ENST000003283920	1.02	1.21E-03	0.0084
RINL	1.02	1.46E-03	0.0093
MPDU1	1.02	1.63E-03	0.0098
LIG1	1.02	2.54E-03	0.0125
HACE1	1.02	1.86E-02	0.0438
KIAA0408	1.02	6.62E-03	0.0220
ENST000002643121	1.02	5.34E-03	0.0192
KMT5A	1.02	2.45E-03	0.0123
BTF3L4	1.02	5.83E-03	0.0203
NAA38	1.02	2.42E-03	0.0122
ENST000002100601	1.02	1.09E-02	0.0304
FAM184A	1.02	1.37E-02	0.0353
GPR180	1.02	3.07E-03	0.0139
SLC6A6	1.02	8.73E-03	0.0262
AVEN	1.02	2.22E-03	0.0117
ENST000003006050	1.02	9.04E-03	0.0267
RHNO1	1.02	1.47E-02	0.0370
SOCS1	1.02	2.21E-02	0.0496
RBM10	1.02	1.57E-03	0.0097
CLNS1A	1.02	2.39E-03	0.0122
AL136040.1	1.02	1.14E-02	0.0313
HIF1A	1.01	2.87E-03	0.0134
ENST000003248682	1.01	2.40E-03	0.0122
SNHG5	1.01	3.21E-03	0.0142
PDRG1	1.01	2.47E-03	0.0124
CALR	1.01	8.88E-03	0.0264
ENST000002760792	1.01	1.25E-03	0.0086
C1orf109	1.01	1.78E-03	0.0103
ENST000003116692	1.01	1.19E-02	0.0321
INPP5F	1.01	2.67E-03	0.0129
TERC	1.01	9.75E-03	0.0281
GTF2H4	1.01	4.63E-03	0.0177
AKIP1	1.01	5.79E-03	0.0202
SCRN3	1.01	6.97E-03	0.0228
CDC42	1.01	1.31E-03	0.0087
GLB1	1.01	2.82E-03	0.0133
NELFCD	1.01	3.62E-03	0.0152
ENST000003100782	1.00	2.42E-03	0.0122
SSNA1	1.00	2.89E-03	0.0135
ENST000003273001	1.00	1.40E-03	0.0091
SLC27A4	1.00	5.40E-03	0.0194
ARPP19	1.00	2.16E-03	0.0114
CPOX	1.00	3.11E-03	0.0140
TNIP1	1.00	8.64E-03	0.0260
COPS4	1.00	1.73E-03	0.0102
COPS9	1.00	3.28E-03	0.0144
UBE2J1	1.00	1.34E-03	0.0088
STX10	1.00	6.12E-03	0.0209
PHAX	1.00	1.65E-03	0.0099
NMD3	1.00	8.35E-03	0.0254

TFB1M	1.00	1.67E-03	0.0100
PIP5K1A	1.00	1.67E-03	0.0100
ENST000003316660	1.00	1.14E-02	0.0313
NUP54	1.00	1.95E-03	0.0108
WDR1	1.00	3.93E-03	0.0160
ENST000003336510	1.00	2.15E-03	0.0114
METTL2B	1.00	3.76E-03	0.0156
TCF3	1.00	3.36E-03	0.0146
RSL1D1	1.00	1.59E-03	0.0097
PSMG2	1.00	1.74E-03	0.0102
NUP188	1.00	2.16E-03	0.0114
TGIF2	1.00	1.87E-03	0.0105
PEF1	0.99	1.87E-03	0.0105
MID1IP1	0.99	2.41E-03	0.0122
DANCR	0.99	5.35E-03	0.0192
ENST000002549502	0.99	7.86E-03	0.0245
FTSJ3	0.99	3.57E-03	0.0151
MRPL43	0.99	7.81E-03	0.0244
C17orf75	0.99	2.41E-03	0.0122
ARL6IP4	0.99	1.68E-03	0.0100
HSCB	0.99	1.94E-02	0.0452
FAM20B	0.99	1.46E-03	0.0093
NDE1	0.99	5.42E-03	0.0194
ZNF664	0.99	2.78E-03	0.0132
B3GNT10	0.99	3.50E-03	0.0149
HIBADH	0.99	1.91E-03	0.0107
MYO1G	0.99	4.31E-03	0.0169
STT3A	0.99	2.10E-03	0.0113
ATP6V1A	0.99	3.43E-03	0.0148
PPP1R16A	0.99	1.41E-02	0.0360
CAPRIN1	0.99	1.49E-03	0.0094
CBR3	0.99	2.78E-03	0.0132
MRRF	0.99	1.50E-03	0.0094
OCRL	0.99	1.10E-02	0.0304
KIAA0100	0.99	1.41E-03	0.0091
ENST000003101440	0.99	7.53E-03	0.0238
ENST000003369150	0.99	1.83E-02	0.0434
PPP2R5B	0.99	2.84E-03	0.0133
BUD23	0.99	1.98E-03	0.0108
SNX4	0.99	1.48E-03	0.0093
GNB2	0.98	2.29E-03	0.0119
UBE2E1	0.98	6.19E-03	0.0211
PEBP1	0.98	2.93E-03	0.0135
ENST000002292682	0.98	3.52E-03	0.0150
PSMD6	0.98	2.63E-03	0.0128
DCAF12	0.98	2.24E-03	0.0117
ZW10	0.98	1.81E-03	0.0104
CCP110	0.98	4.38E-03	0.0171
ATP23	0.98	5.43E-03	0.0194
ATP6V0B	0.98	9.31E-03	0.0272

CUL1	0.98	1.47E-03	0.0093
ELP6	0.98	6.37E-03	0.0215
NF1	0.98	1.44E-03	0.0092
CSKMT	0.98	1.89E-02	0.0443
ZNF544	0.98	1.14E-02	0.0313
PURB	0.98	2.25E-03	0.0118
PRPF19	0.98	3.72E-03	0.0155
RSL24D1	0.98	2.77E-03	0.0132
LANCL2	0.98	8.49E-03	0.0257
APEX1	0.98	2.05E-03	0.0111
PWP1	0.98	2.69E-03	0.0130
DFFA	0.98	4.51E-03	0.0174
MRPS33	0.97	3.39E-03	0.0147
RPL35	0.97	1.53E-03	0.0095
POU2F2	0.97	1.27E-02	0.0335
GLE1	0.97	1.81E-03	0.0104
MAPKAPK2	0.97	4.68E-03	0.0178
AMPD2	0.97	9.93E-03	0.0285
MTA2	0.97	2.58E-03	0.0127
KCMF1	0.97	2.53E-03	0.0125
LSM1	0.97	2.41E-03	0.0122
ENST000002828910	0.97	1.71E-02	0.0411
COPS6	0.97	3.77E-03	0.0156
SPATA20	0.97	1.92E-02	0.0447
PDCD11	0.97	2.82E-03	0.0133
POLR1E	0.97	2.61E-03	0.0127
MTG1	0.97	1.15E-02	0.0314
POP4	0.97	1.59E-03	0.0097
SNORD101	0.97	2.04E-02	0.0468
PNP	0.97	3.65E-03	0.0153
ENST000002617722	0.97	1.47E-02	0.0370
CEP57	0.97	2.90E-03	0.0135
RPS2	0.96	1.64E-03	0.0099
TMEM141	0.96	7.34E-03	0.0234
RASSF1	0.96	2.66E-03	0.0129
FAHD1	0.96	2.95E-03	0.0136
E2F2	0.96	1.56E-02	0.0387
SCMH1	0.96	4.14E-03	0.0165
SNX12	0.96	2.82E-03	0.0133
UBA6	0.96	1.59E-03	0.0097
GFM1	0.96	2.72E-03	0.0130
SMARCD2	0.96	2.00E-03	0.0109
ABRACL	0.96	3.80E-03	0.0157
PYCR3	0.96	9.00E-03	0.0267
LDLR	0.96	8.96E-03	0.0266
DDX56	0.96	1.91E-03	0.0106
SPINDOC	0.96	8.39E-03	0.0255
VMA21	0.96	6.54E-03	0.0218
HDX	0.96	1.45E-02	0.0367
RAD21	0.96	5.03E-03	0.0185

HDGFL2	0.96	1.99E-03	0.0109
MCRIP2	0.96	4.87E-03	0.0182
NAXE	0.96	7.17E-03	0.0231
HNRNPR	0.96	2.34E-03	0.0120
MRPL35	0.96	2.32E-03	0.0120
UHRF1BP1	0.96	1.91E-03	0.0106
ZNF121	0.95	6.62E-03	0.0220
PRCP	0.95	5.65E-03	0.0199
DDX10	0.95	2.75E-03	0.0131
ENST000003079610	0.95	3.87E-03	0.0158
GSS	0.95	6.81E-03	0.0224
ENST000002992590	0.95	2.48E-03	0.0124
ENST000002658720	0.95	4.17E-03	0.0166
ENST000003013290	0.95	2.27E-03	0.0118
SRSF7	0.95	3.37E-03	0.0146
AC008543.1	0.95	1.25E-02	0.0331
EXOC5	0.95	2.80E-03	0.0133
G3BP1	0.95	2.04E-03	0.0110
AC245060.4	0.95	1.05E-02	0.0295
ZBTB10	0.95	1.32E-02	0.0343
HCCS	0.95	3.38E-03	0.0146
AC020928.1	0.95	1.63E-02	0.0397
SRP14	0.95	1.45E-02	0.0368
AC011815.1	0.95	9.08E-03	0.0268
MAT2A	0.95	6.21E-03	0.0211
ABCA3	0.95	1.41E-02	0.0361
CCDC43	0.95	7.68E-03	0.0241
BAZ1B	0.95	1.86E-03	0.0105
FASTKD1	0.95	1.88E-03	0.0106
PPP1R15A	0.95	8.35E-03	0.0254
SNRNP27	0.95	3.13E-03	0.0140
ARMC10	0.95	4.79E-03	0.0180
GSPT1	0.95	3.43E-03	0.0147
DOLPP1	0.94	5.92E-03	0.0205
ELOF1	0.94	4.78E-03	0.0180
CENPBD1P1	0.94	4.16E-03	0.0165
ORC3	0.94	1.73E-03	0.0102
RBL1	0.94	2.79E-03	0.0132
TSPAN5	0.94	3.06E-03	0.0139
TMEM241	0.94	2.01E-02	0.0463
PITHD1	0.94	1.88E-03	0.0106
MRPL30	0.94	1.84E-03	0.0105
NDUFAF3	0.94	1.33E-02	0.0345
DNAJC8	0.94	2.07E-03	0.0112
ENST000003141380	0.94	3.14E-03	0.0140
TCERG1	0.94	1.72E-03	0.0101
TRUB2	0.94	3.98E-03	0.0161
KAT5	0.94	3.07E-03	0.0139
COMMD8	0.94	2.26E-03	0.0118
ARMCX6	0.94	3.00E-03	0.0137

ENST000002547300	0.93	1.29E-02	0.0339
NOB1	0.93	4.94E-03	0.0183
CHRFAM7A	0.93	2.06E-02	0.0472
RSC1A1	0.93	1.01E-02	0.0287
ARL2	0.93	3.47E-03	0.0149
NDUFC2	0.93	4.81E-03	0.0180
SMU1	0.93	2.28E-03	0.0119
PAF1	0.93	2.38E-03	0.0121
TSR3	0.93	3.38E-03	0.0146
CIDEB	0.93	5.97E-03	0.0206
THOC7	0.93	4.22E-03	0.0167
ATPAF1	0.93	2.04E-03	0.0111
PSMD5	0.93	1.88E-03	0.0106
LYPLA2	0.93	4.47E-03	0.0173
PTOV1	0.93	3.85E-03	0.0158
POLB	0.93	2.03E-03	0.0110
ENST000003108640	0.93	2.29E-03	0.0119
CSTF1	0.93	2.14E-03	0.0114
CFL1	0.93	1.32E-02	0.0344
TARS2	0.93	5.65E-03	0.0199
SRPRB	0.93	9.42E-03	0.0274
C12orf10	0.93	4.23E-03	0.0167
EMC4	0.93	3.00E-03	0.0137
ZMPSTE24	0.93	4.74E-03	0.0179
AC007996.1	0.93	7.73E-03	0.0242
ALKBH1	0.93	3.33E-03	0.0145
ENST000002988182	0.93	1.68E-02	0.0407
GPHN	0.93	4.21E-03	0.0167
KDM1A	0.92	2.86E-03	0.0134
CBWD2	0.92	3.15E-03	0.0141
DUS4L	0.92	6.73E-03	0.0223
RPN1	0.92	8.54E-03	0.0258
CD81	0.92	4.50E-03	0.0174
MYBBP1A	0.92	6.09E-03	0.0209
COPS8	0.92	5.48E-03	0.0195
NME7	0.92	2.24E-03	0.0117
TBL1XR1	0.92	3.89E-03	0.0159
PARD6A	0.92	1.49E-02	0.0374
TKT	0.92	9.62E-03	0.0278
GPAA1	0.92	9.63E-03	0.0279
ENST000003027460	0.92	2.48E-03	0.0124
TTC1	0.92	2.28E-03	0.0119
CTSZ	0.92	2.81E-03	0.0133
CLIC1	0.92	6.57E-03	0.0219
TPD52L2	0.92	7.75E-03	0.0242
CPSF4	0.92	2.45E-03	0.0123
POLR2C	0.92	4.46E-03	0.0173
BIVM	0.92	2.92E-03	0.0135
RPS6KA1	0.92	8.62E-03	0.0259
POLR2D	0.92	3.29E-03	0.0144

TSN	0.92	2.04E-03	0.0111
BLOC1S1	0.92	5.16E-03	0.0188
ARHGEF35	0.92	6.75E-03	0.0223
NCLN	0.92	1.63E-02	0.0399
DNAJB2	0.92	2.80E-03	0.0133
ATL2	0.92	3.85E-03	0.0158
TRUB1	0.92	6.92E-03	0.0227
VTA1	0.92	1.97E-03	0.0108
ENST000003220671	0.91	4.71E-03	0.0178
ANP32B	0.91	1.95E-03	0.0108
HARS	0.91	2.23E-03	0.0117
RPL7	0.91	3.03E-03	0.0138
SLAMF6	0.91	1.49E-02	0.0374
INIP	0.91	1.98E-03	0.0109
TRMT61B	0.91	1.53E-02	0.0382
BCS1L	0.91	3.17E-03	0.0141
ESF1	0.91	5.70E-03	0.0200
B3GALT6	0.91	8.39E-03	0.0255
ENST000003494310	0.91	4.59E-03	0.0176
MAPRE1	0.91	2.55E-03	0.0126
COX5B	0.91	3.93E-03	0.0160
GON7	0.91	6.10E-03	0.0209
DHRS4	0.91	6.24E-03	0.0212
SDHD	0.91	2.55E-03	0.0126
COMMD1	0.91	4.56E-03	0.0175
GTF3C4	0.91	2.07E-03	0.0111
SENP8	0.91	7.04E-03	0.0229
TIPRL	0.91	2.17E-03	0.0114
RTEL1	0.91	1.65E-02	0.0402
MRPL32	0.91	2.33E-03	0.0120
FBXW11	0.91	5.11E-03	0.0187
NUP43	0.91	3.61E-03	0.0152
IER3IP1	0.91	4.37E-03	0.0171
SCYL1	0.90	3.01E-03	0.0137
GMFB	0.90	4.71E-03	0.0178
RPL6P27	0.90	8.88E-03	0.0264
GLS	0.90	2.53E-03	0.0125
BCAT2	0.90	2.03E-02	0.0467
METTL2A	0.90	2.42E-03	0.0122
TMEM165	0.90	2.53E-03	0.0125
PTPDC1	0.90	1.03E-02	0.0291
RBM15	0.90	1.99E-03	0.0109
CORO1B	0.90	3.53E-03	0.0150
POLE3	0.90	6.45E-03	0.0217
RRP8	0.90	4.03E-03	0.0162
FNTB	0.90	4.41E-03	0.0172
DPY30	0.90	3.21E-03	0.0142
DDX12P	0.90	7.11E-03	0.0230
VKORC1	0.90	1.00E-02	0.0286
IMP3	0.90	4.86E-03	0.0181

BABAM2	0.90	1.11E-02	0.0306
TMCO3	0.90	4.59E-03	0.0176
QRSL1	0.90	3.55E-03	0.0150
NDUFS2	0.90	2.65E-03	0.0128
APEX2	0.90	5.30E-03	0.0191
IVNS1ABP	0.90	5.76E-03	0.0202
DTD2	0.90	4.73E-03	0.0179
CCDC77	0.90	4.52E-03	0.0174
EIF2AK4	0.90	3.29E-03	0.0144
MT-ND2	0.90	3.76E-03	0.0156
SEPHS1	0.90	4.42E-03	0.0172
AP2M1	0.90	5.92E-03	0.0205
SEC11C	0.90	3.68E-03	0.0154
ZFP1	0.90	2.96E-03	0.0136
PAGR1	0.89	6.61E-03	0.0220
SVIL-AS1	0.89	2.23E-02	0.0499
EXOSC7	0.89	3.23E-03	0.0142
ACOT13	0.89	4.66E-03	0.0177
MYL6	0.89	3.77E-03	0.0156
NUDT19	0.89	7.52E-03	0.0238
TARBP2	0.89	1.23E-02	0.0328
ENST000002557590	0.89	2.53E-03	0.0125
ENST000002209660	0.89	1.95E-02	0.0453
TMX2	0.89	1.30E-02	0.0341
CWC15	0.89	2.27E-03	0.0118
ILF3	0.89	2.11E-03	0.0113
POT1	0.89	2.31E-03	0.0119
UTP4	0.89	4.21E-03	0.0167
SGCB	0.89	1.20E-02	0.0322
AKR7A2	0.89	2.88E-03	0.0134
TAF5	0.89	9.81E-03	0.0282
PDCL	0.89	3.56E-03	0.0151
NOM1	0.89	7.16E-03	0.0231
MIPEP	0.89	9.15E-03	0.0269
NPTN	0.89	3.34E-03	0.0145
ENST000002611822	0.89	6.13E-03	0.0210
SSBP1	0.89	4.10E-03	0.0164
MT-ND5	0.89	4.80E-03	0.0180
GCN1	0.89	3.96E-03	0.0160
BCAS2	0.89	3.88E-03	0.0159
TRAPPC4	0.89	1.71E-02	0.0411
ENST000002964110	0.89	9.90E-03	0.0284
ZUP1	0.89	3.24E-03	0.0143
FUBP3	0.89	3.24E-03	0.0142
CKAP4	0.89	5.26E-03	0.0190
ENST000003219193	0.89	1.04E-02	0.0293
ATN1	0.89	1.14E-02	0.0312
NDUFA11	0.89	1.15E-02	0.0314
MED19	0.89	9.39E-03	0.0274
NFKBIB	0.89	1.85E-02	0.0436

LIN54	0.89	3.01E-03	0.0137
TCTN1	0.89	7.17E-03	0.0231
EXOSC3	0.89	3.21E-03	0.0142
TAX1BP3	0.89	1.86E-02	0.0438
ZNF598	0.88	6.58E-03	0.0219
HNRNPA0	0.88	2.97E-03	0.0136
SLC5A6	0.88	1.11E-02	0.0307
MLEC	0.88	1.01E-02	0.0288
VPS35	0.88	4.70E-03	0.0178
CEP131	0.88	5.34E-03	0.0192
PLOD2	0.88	8.62E-03	0.0259
KPNA3	0.88	2.90E-03	0.0135
ECHDC1	0.88	2.90E-03	0.0135
UBR5	0.88	2.30E-03	0.0119
SRF	0.88	4.52E-03	0.0174
LETM2	0.88	2.23E-02	0.0499
LEO1	0.88	3.78E-03	0.0156
ZNF93	0.88	7.09E-03	0.0230
EEF1AKNMT	0.88	6.47E-03	0.0217
ENST000003331672	0.88	6.15E-03	0.0210
UBL5	0.88	2.31E-03	0.0119
CAAP1	0.88	3.49E-03	0.0149
IFT52	0.88	5.29E-03	0.0191
SSSCA1	0.88	1.77E-02	0.0423
RDH11	0.88	3.69E-03	0.0154
RAB1A	0.88	2.88E-03	0.0134
ENST000003085801	0.88	6.34E-03	0.0214
MAPKAP1	0.88	4.03E-03	0.0162
HRAS	0.88	2.00E-02	0.0460
NUS1	0.87	4.30E-03	0.0169
COQ7	0.87	6.75E-03	0.0223
ADAT2	0.87	8.48E-03	0.0257
ENST00000344736	0.87	2.04E-02	0.0468
CSNK2B	0.87	8.11E-03	0.0250
DNAJC11	0.87	2.85E-03	0.0134
THRAP3	0.87	2.50E-03	0.0124
ENST000003282731	0.87	1.76E-02	0.0421
PITPNA-AS1	0.87	1.61E-02	0.0395
TTC7A	0.87	1.57E-02	0.0388
SRSF10	0.87	3.50E-03	0.0149
ENST000002771650	0.87	4.08E-03	0.0163
SLC25A17	0.87	4.05E-03	0.0162
LETM1	0.87	4.03E-03	0.0162
MYC	0.87	9.40E-03	0.0274
ZDHHC18	0.87	3.11E-03	0.0140
AFAP1	0.87	2.06E-02	0.0471
RNF40	0.87	3.70E-03	0.0154
DDT	0.87	3.80E-03	0.0157
TRMT2A	0.87	2.14E-02	0.0485
TBC1D9B	0.87	3.93E-03	0.0160

TOMM70	0.87	2.93E-03	0.0135
DDX23	0.87	4.14E-03	0.0165
FYN	0.87	3.37E-03	0.0146
MGAT4B	0.87	1.45E-02	0.0367
IMPACT	0.87	7.05E-03	0.0229
TTLL4	0.87	5.23E-03	0.0190
ARPC5	0.87	4.91E-03	0.0183
CARNMT1	0.87	4.96E-03	0.0184
CCDC171	0.87	5.46E-03	0.0195
ENST000003088600	0.86	2.03E-02	0.0467
LDAH	0.86	4.12E-03	0.0164
WDR61	0.86	3.07E-03	0.0139
MRPS11	0.86	4.62E-03	0.0176
ENST000002782243	0.86	1.53E-02	0.0380
GBP2	0.86	4.50E-03	0.0174
CTDSPL2	0.86	3.42E-03	0.0147
DHX57	0.86	3.25E-03	0.0143
TMEM258	0.86	4.81E-03	0.0180
UBE3C	0.86	2.89E-03	0.0135
HIVEP1	0.86	2.48E-03	0.0124
ADGRE5	0.86	7.98E-03	0.0247
CAB39	0.86	4.61E-03	0.0176
ENTR1	0.86	2.56E-03	0.0126
ADNP2	0.86	3.29E-03	0.0144
AIMP1	0.86	4.22E-03	0.0167
BTBD1	0.86	3.34E-03	0.0145
CHAMP1	0.86	1.38E-02	0.0355
ARV1	0.86	4.74E-03	0.0179
PDHX	0.86	4.09E-03	0.0164
HSDL2	0.86	7.04E-03	0.0229
ANXA5	0.86	3.40E-03	0.0147
GRAMD4	0.86	4.45E-03	0.0172
CDADC1	0.86	2.11E-02	0.0480
MED8	0.86	6.18E-03	0.0210
CHUK	0.86	3.29E-03	0.0144
RAMAC	0.86	3.36E-03	0.0146
NDUFB1	0.86	2.66E-03	0.0129
ENST000003419230	0.86	2.98E-03	0.0137
SWAP70	0.86	6.66E-03	0.0221
KCTD20	0.86	2.59E-03	0.0127
ACSL3	0.85	9.57E-03	0.0278
RTCA	0.85	6.90E-03	0.0226
SESN2	0.85	2.00E-02	0.0461
ZDHHC14	0.85	2.05E-02	0.0470
DUS1L	0.85	8.49E-03	0.0257
ARMT1	0.85	2.76E-03	0.0131
IQGAP1	0.85	3.40E-03	0.0147
CPSF2	0.85	4.78E-03	0.0180
HIBCH	0.85	1.42E-02	0.0362
MRPL57	0.85	2.93E-03	0.0135

MCCC2	0.85	8.24E-03	0.0253
ENST000003147970	0.85	9.20E-03	0.0270
NUP133	0.85	2.60E-03	0.0127
SUPV3L1	0.85	6.99E-03	0.0228
PSMD9	0.85	3.38E-03	0.0146
POU2F1	0.85	5.43E-03	0.0194
ARHGAP21	0.85	3.64E-03	0.0153
QSER1	0.85	4.64E-03	0.0177
BCOR	0.85	4.00E-03	0.0161
C2orf69	0.85	6.44E-03	0.0216
PPP2CA	0.85	2.61E-03	0.0127
MFSD14B	0.85	2.53E-03	0.0125
DPP9	0.85	1.25E-02	0.0332
IPO11	0.85	9.37E-03	0.0273
PMM2	0.84	6.76E-03	0.0223
DHRS11	0.84	1.96E-02	0.0455
TNPO2	0.84	3.56E-03	0.0151
CEBPZOS	0.84	4.78E-03	0.0180
TOP1	0.84	4.61E-03	0.0176
RPS2P46	0.84	1.76E-02	0.0420
HSPB11	0.84	4.61E-03	0.0176
GRINA	0.84	1.71E-02	0.0412
TRAK1	0.84	5.63E-03	0.0198
TMEM9	0.84	1.14E-02	0.0312
ENST000002162881	0.84	2.75E-03	0.0131
METTL8	0.84	2.84E-03	0.0134
DDX55	0.84	3.10E-03	0.0140
UPF3B	0.84	3.20E-03	0.0142
KLHL12	0.84	3.35E-03	0.0145
LBR	0.84	3.70E-03	0.0154
PTGES2	0.84	1.00E-02	0.0286
ABHD5	0.84	8.74E-03	0.0262
HDAC3	0.84	4.07E-03	0.0163
ZNF330	0.84	3.11E-03	0.0140
KLHDC3	0.84	3.97E-03	0.0161
INTS14	0.84	5.86E-03	0.0204
GATB	0.84	4.37E-03	0.0170
ENST000002687971	0.84	6.78E-03	0.0223
SPATS2	0.84	1.50E-02	0.0376
DHX35	0.84	6.99E-03	0.0228
MARS	0.84	1.93E-02	0.0449
NAA25	0.84	4.00E-03	0.0161
ECPAS	0.84	3.17E-03	0.0141
AP1AR	0.84	4.15E-03	0.0165
ZDHHC12	0.84	1.23E-02	0.0328
MED24	0.84	5.82E-03	0.0203
SOGA1	0.84	1.47E-02	0.0370
RIOX2	0.84	1.15E-02	0.0314
MMS19	0.84	5.04E-03	0.0186
RAMACL	0.84	2.20E-02	0.0494

COA3	0.84	7.30E-03	0.0233
CFAP298	0.84	2.87E-03	0.0134
ENST000003346510	0.84	1.48E-02	0.0371
PSMB10	0.84	2.73E-03	0.0131
C3orf33	0.84	1.36E-02	0.0350
RAB10	0.84	5.92E-03	0.0205
ASCC1	0.84	1.43E-02	0.0363
ENST000003105130	0.84	1.46E-02	0.0369
RBM27	0.83	3.18E-03	0.0141
RNF219	0.83	1.09E-02	0.0303
MRPL42	0.83	2.90E-03	0.0135
UAP1L1	0.83	9.94E-03	0.0285
PABPC4	0.83	3.24E-03	0.0142
IL15RA	0.83	8.83E-03	0.0264
PRKRA	0.83	3.10E-03	0.0140
PDCD10	0.83	3.07E-03	0.0139
ZCCHC17	0.83	4.30E-03	0.0169
MTPN	0.83	4.70E-03	0.0178
AGO1	0.83	8.83E-03	0.0264
HSPA1B	0.83	3.40E-03	0.0147
NMT1	0.83	4.31E-03	0.0169
C12orf57	0.83	9.42E-03	0.0274
ARF1	0.83	8.80E-03	0.0263
SMPD4	0.83	6.30E-03	0.0213
ZNF678	0.83	1.04E-02	0.0292
PLEKHG2	0.83	1.15E-02	0.0314
MRPS5	0.83	3.00E-03	0.0137
GTF3C3	0.83	5.12E-03	0.0187
GTF2E2	0.83	7.14E-03	0.0230
RPL8	0.83	6.87E-03	0.0226
U2SURP	0.83	6.83E-03	0.0225
DYNLT3	0.82	1.97E-02	0.0456
CYTIP	0.82	3.99E-03	0.0161
ENST000003274350	0.82	1.40E-02	0.0358
AC091057.6	0.82	8.54E-03	0.0258
ARMH1	0.82	1.23E-02	0.0328
TYW3	0.82	3.93E-03	0.0160
XYLT2	0.82	7.49E-03	0.0237
SRSF4	0.82	3.36E-03	0.0146
OGFOD1	0.82	6.38E-03	0.0215
DDX52	0.82	7.04E-03	0.0229
DUSP16	0.82	1.19E-02	0.0321
ELOB	0.82	6.08E-03	0.0208
USP6NL	0.82	1.93E-02	0.0451
RAP1A	0.82	3.18E-03	0.0141
IFT57	0.82	3.94E-03	0.0160
TAF11	0.82	5.08E-03	0.0186
SLC39A3	0.82	1.01E-02	0.0287
GTF2H5	0.82	4.86E-03	0.0181
AKT1	0.82	5.08E-03	0.0186

HSF1	0.82	1.22E-02	0.0327
DSTYK	0.82	3.02E-03	0.0137
TMEM186	0.82	6.41E-03	0.0215
BAG4	0.81	3.93E-03	0.0160
TRIM25	0.81	4.21E-03	0.0167
CAP1	0.81	3.51E-03	0.0149
LIPT2	0.81	2.10E-02	0.0478
TCEAL1	0.81	1.69E-02	0.0409
DHX30	0.81	3.14E-03	0.0140
ZNF473	0.81	1.22E-02	0.0327
PPID	0.81	3.66E-03	0.0153
TAF9	0.81	5.33E-03	0.0192
SARS	0.81	6.12E-03	0.0209
LARP1B	0.81	7.38E-03	0.0235
ENST000003142892	0.81	3.11E-03	0.0140
ENST000003491570	0.81	4.91E-03	0.0183
ATAD3B	0.81	1.13E-02	0.0310
MPV17L2	0.81	1.19E-02	0.0321
RAP2C	0.81	5.89E-03	0.0204
ALKBH3	0.81	6.49E-03	0.0217
ACOT9	0.81	3.84E-03	0.0158
HSP90B1	0.81	5.84E-03	0.0203
MNAT1	0.81	9.40E-03	0.0274
RNASEH2C	0.81	5.43E-03	0.0194
ZNF232	0.81	6.05E-03	0.0208
MED1	0.81	4.31E-03	0.0169
PISD	0.81	1.04E-02	0.0294
NOP9	0.81	5.59E-03	0.0197
ALG8	0.81	2.05E-02	0.0471
DNTTIP2	0.81	3.76E-03	0.0156
PQLC1	0.80	1.02E-02	0.0289
RPS9	0.80	4.61E-03	0.0176
ST13	0.80	3.91E-03	0.0160
MRPL52	0.80	9.80E-03	0.0282
CLPX	0.80	4.27E-03	0.0168
HAVCR2	0.80	8.17E-03	0.0251
EXOSC10	0.80	3.70E-03	0.0154
GNL3L	0.80	1.24E-02	0.0330
WRN	0.80	5.49E-03	0.0196
ENST000002632024	0.80	5.60E-03	0.0198
DUSP12	0.80	1.17E-02	0.0318
PRPF40A	0.80	4.12E-03	0.0164
SLC39A9	0.80	4.21E-03	0.0167
RRAGA	0.80	6.32E-03	0.0214
CNOT1	0.80	4.89E-03	0.0182
ENST000002501131	0.80	1.04E-02	0.0294
TRMT1	0.80	6.95E-03	0.0227
NDFIP1	0.80	1.44E-02	0.0365
PPP1CA	0.80	1.13E-02	0.0310
CREB3L4	0.80	2.11E-02	0.0480

MRPS18C	0.80	3.85E-03	0.0158
ARPC1B	0.80	9.02E-03	0.0267
NUP153	0.80	3.50E-03	0.0149
DDOST	0.80	1.60E-02	0.0394
SLC35B4	0.80	5.26E-03	0.0190
BBIP1	0.80	4.41E-03	0.0172
ENST000003155542	0.80	9.13E-03	0.0269
PTGR2	0.80	1.39E-02	0.0357
EWSR1	0.79	3.86E-03	0.0158
RPL22	0.79	4.25E-03	0.0167
APOOL	0.79	7.66E-03	0.0241
SND1	0.79	4.20E-03	0.0166
ENST000002682061	0.79	4.51E-03	0.0174
TRMT6	0.79	7.93E-03	0.0246
PTPMT1	0.79	5.68E-03	0.0200
NUBP1	0.79	6.61E-03	0.0220
ENST000003055360	0.79	4.57E-03	0.0176
ARF5	0.79	8.47E-03	0.0257
ERLIN1	0.79	5.80E-03	0.0203
GOLPH3	0.79	7.17E-03	0.0231
IPP	0.79	4.79E-03	0.0180
EIF3M	0.79	7.27E-03	0.0233
AP5B1	0.79	1.02E-02	0.0289
PUS1	0.79	4.67E-03	0.0178
ALDH5A1	0.79	8.60E-03	0.0259
KIF1C	0.79	6.60E-03	0.0220
NBN	0.79	3.57E-03	0.0151
RWDD4	0.79	6.14E-03	0.0210
HADHA	0.79	4.23E-03	0.0167
PALB2	0.79	4.44E-03	0.0172
MED26	0.79	1.72E-02	0.0412
PRMT6	0.79	5.82E-03	0.0203
EGLN1	0.79	6.73E-03	0.0223
NELFB	0.79	4.09E-03	0.0164
THAP12	0.79	6.30E-03	0.0213
RBMXL1	0.79	9.10E-03	0.0268
L2HGDH	0.78	1.95E-02	0.0452
ERG28	0.78	2.07E-02	0.0474
SUB1	0.78	3.57E-03	0.0151
ADAM8	0.78	6.82E-03	0.0224
MTF2	0.78	4.25E-03	0.0168
WDYHV1	0.78	1.04E-02	0.0293
TATDN1	0.78	7.20E-03	0.0231
CHMP4B	0.78	1.38E-02	0.0355
SCO1	0.78	3.77E-03	0.0156
TMED2	0.78	1.53E-02	0.0382
MECR	0.78	1.11E-02	0.0308
DYRK4	0.78	9.56E-03	0.0277
PRXL2C	0.78	8.86E-03	0.0264
RRP36	0.78	7.71E-03	0.0242

MICU1	0.78	5.55E-03	0.0197
AC012314.13	0.78	6.34E-03	0.0214
AC012314.22	0.78	6.34E-03	0.0214
AC012314.24	0.78	6.34E-03	0.0214
AC012314.3	0.78	6.34E-03	0.0214
AC012314.30	0.78	6.34E-03	0.0214
AC012314.7	0.78	6.34E-03	0.0214
GLUD1	0.78	1.33E-02	0.0345
ABHD11	0.78	1.46E-02	0.0368
ASMTL	0.78	1.59E-02	0.0392
NGDN	0.78	1.03E-02	0.0292
ENST000003552720	0.78	4.35E-03	0.0170
E2F4	0.78	4.42E-03	0.0172
SFPQ	0.78	5.86E-03	0.0204
MINOS1	0.78	4.51E-03	0.0174
PLEKHJ1	0.78	7.60E-03	0.0239
CTPS2	0.78	8.72E-03	0.0262
ZFP91	0.78	7.33E-03	0.0234
PACSIN2	0.78	1.47E-02	0.0370
EIF2S3	0.78	6.19E-03	0.0211
MAP9	0.78	2.04E-02	0.0468
B3GNT2	0.78	1.55E-02	0.0385
CDK5	0.77	1.78E-02	0.0425
PPP1R8	0.77	4.64E-03	0.0177
ARL6IP6	0.77	4.49E-03	0.0173
ENST000002027733	0.77	3.97E-03	0.0161
ARFIP2	0.77	5.73E-03	0.0201
GNAI3	0.77	5.29E-03	0.0191
EIF2B1	0.77	3.93E-03	0.0160
LRRC8B	0.77	7.20E-03	0.0231
ENST000002650692	0.77	3.73E-03	0.0155
NDUFV1	0.77	6.64E-03	0.0221
INTS13	0.77	1.14E-02	0.0312
ZNF106	0.77	4.93E-03	0.0183
SLC2A1	0.77	1.55E-02	0.0384
EIF2B5	0.77	4.37E-03	0.0171
THAP4	0.77	7.62E-03	0.0240
ATF1	0.77	5.30E-03	0.0191
RPS27L	0.77	6.07E-03	0.0208
NOL11	0.77	5.92E-03	0.0205
RPL21	0.77	9.81E-03	0.0282
KIF1BP	0.77	5.51E-03	0.0196
ZSCAN5A	0.77	1.17E-02	0.0317
APLP2	0.77	1.41E-02	0.0360
ALKBH8	0.77	1.80E-02	0.0428
CRY1	0.77	1.62E-02	0.0396
TUBGCP4	0.77	9.75E-03	0.0281
MTMR4	0.77	7.05E-03	0.0229
ADCK2	0.77	8.21E-03	0.0252
ASNSD1	0.77	8.52E-03	0.0258

RPP40	0.77	1.97E-02	0.0456
PRKAR1A	0.77	1.74E-02	0.0418
C1orf122	0.77	1.91E-02	0.0446
GMDS	0.77	1.70E-02	0.0411
ERGIC2	0.77	5.35E-03	0.0192
SLC39A6	0.77	7.82E-03	0.0244
TAF2	0.76	8.25E-03	0.0253
CEP85	0.76	4.36E-03	0.0170
STK26	0.76	4.29E-03	0.0169
CMC2	0.76	3.96E-03	0.0161
SNRNP200	0.76	4.66E-03	0.0177
ENST000003237010	0.76	5.50E-03	0.0196
ENST000003268731	0.76	7.41E-03	0.0235
SMC5	0.76	6.27E-03	0.0212
THAP3	0.76	1.92E-02	0.0448
RPL5	0.76	5.73E-03	0.0201
ENST000002792300	0.76	1.34E-02	0.0347
CCNT1	0.76	4.94E-03	0.0183
BRCC3	0.76	1.64E-02	0.0400
COX6B1	0.76	5.81E-03	0.0203
UNC119B	0.76	5.87E-03	0.0204
AC006333.2	0.76	1.92E-02	0.0448
UBAC1	0.76	1.13E-02	0.0310
AGMAT	0.76	8.97E-03	0.0266
FAM49B	0.75	4.43E-03	0.0172
ADIPOR1	0.75	1.58E-02	0.0391
DHRS13	0.75	1.98E-02	0.0458
CEP97	0.75	1.23E-02	0.0327
STX6	0.75	6.30E-03	0.0213
CCT2	0.75	2.14E-02	0.0484
OST4	0.75	8.74E-03	0.0262
TRMT11	0.75	1.44E-02	0.0365
CEP76	0.75	8.49E-03	0.0257
HIKESHI	0.75	4.21E-03	0.0167
FCF1	0.75	9.97E-03	0.0285
ENST000002284950	0.75	5.57E-03	0.0197
COMMD5	0.75	9.16E-03	0.0270
RPL7A	0.75	4.07E-03	0.0163
BSG	0.75	6.27E-03	0.0212
PNN	0.75	4.18E-03	0.0166
COPB2	0.75	4.39E-03	0.0171
IRAK2	0.75	1.37E-02	0.0353
NUP50	0.75	4.67E-03	0.0178
EIF3L	0.75	7.83E-03	0.0244
EYA3	0.75	7.33E-03	0.0234
RAB9A	0.75	9.30E-03	0.0272
RPS24	0.75	4.68E-03	0.0178
IMPA1	0.74	1.22E-02	0.0326
CDC37	0.74	4.36E-03	0.0170
CD2	0.74	5.24E-03	0.0190

RPL18A	0.74	7.43E-03	0.0236
PAFAH1B2	0.74	7.62E-03	0.0240
ENST000003557650	0.74	5.43E-03	0.0194
SS18L2	0.74	1.26E-02	0.0333
OAT	0.74	6.04E-03	0.0208
ISY1	0.74	5.05E-03	0.0186
RMDN1	0.74	4.50E-03	0.0174
STK16	0.74	9.30E-03	0.0272
GMPS	0.74	4.95E-03	0.0183
HNRNPU	0.74	4.55E-03	0.0175
C11orf58	0.74	5.62E-03	0.0198
CENPB	0.74	1.12E-02	0.0309
CNOT10	0.74	1.13E-02	0.0310
PAAF1	0.74	1.24E-02	0.0330
PPP2R1B	0.74	1.10E-02	0.0304
ATP6V1B2	0.74	5.74E-03	0.0201
DPH3	0.74	6.78E-03	0.0224
STK24	0.74	4.62E-03	0.0176
VEZT	0.74	5.06E-03	0.0186
USP16	0.74	1.13E-02	0.0311
ENST000002164421	0.74	6.76E-03	0.0223
DMWD	0.74	2.17E-02	0.0490
FDXACB1	0.74	2.10E-02	0.0479
MED7	0.74	5.16E-03	0.0188
KCNAB2	0.74	4.43E-03	0.0172
YKT6	0.73	7.24E-03	0.0232
ABHD15	0.73	8.75E-03	0.0262
COMT	0.73	8.26E-03	0.0253
OXSM	0.73	1.83E-02	0.0433
ENST000002665083	0.73	1.82E-02	0.0430
SLC35G1	0.73	1.42E-02	0.0362
RPS6	0.73	4.24E-03	0.0167
DHX38	0.73	7.04E-03	0.0229
PRIMPOL	0.73	2.19E-02	0.0493
NDUFB2	0.73	6.49E-03	0.0217
NAP1L1	0.73	5.27E-03	0.0190
C1orf216	0.73	8.20E-03	0.0252
SLC41A1	0.73	1.35E-02	0.0349
DBR1	0.73	5.12E-03	0.0187
RPL36	0.73	6.20E-03	0.0211
PEX5	0.73	9.67E-03	0.0279
POLR3F	0.73	1.91E-02	0.0446
IFT22	0.73	1.22E-02	0.0326
RSRC1	0.73	4.77E-03	0.0180
RPL24	0.73	5.51E-03	0.0196
RPL22P1	0.73	8.09E-03	0.0250
CEP44	0.73	5.52E-03	0.0196
MED18	0.72	7.11E-03	0.0230
TUBGCP3	0.72	9.98E-03	0.0285
SENP1	0.72	6.88E-03	0.0226

ZCCHC9	0.72	4.76E-03	0.0180
AC091959.3	0.72	8.87E-03	0.0264
MIOS	0.72	8.65E-03	0.0260
SLC25A36	0.72	5.77E-03	0.0202
SRSF2	0.72	8.93E-03	0.0265
RTRAF	0.72	4.90E-03	0.0182
CNIH4	0.72	5.04E-03	0.0186
ENST000002633841	0.72	1.27E-02	0.0336
PRPSAP2	0.72	5.51E-03	0.0196
PMS1	0.72	5.74E-03	0.0201
NFATC1	0.72	1.69E-02	0.0408
CSTF3	0.72	5.54E-03	0.0196
DIS3	0.72	4.95E-03	0.0184
COIL	0.72	6.87E-03	0.0226
MTREX	0.72	6.99E-03	0.0228
TNIP2	0.72	9.40E-03	0.0274
MAP4K3-DT	0.72	2.03E-02	0.0467
SFR1	0.72	1.39E-02	0.0356
ENST000003044141	0.72	7.22E-03	0.0232
PRKAG1	0.72	1.03E-02	0.0291
ENST000003233450	0.72	4.93E-03	0.0183
ATP5MG	0.72	4.77E-03	0.0180
TXNDC9	0.72	5.27E-03	0.0191
UBE2D2	0.72	7.02E-03	0.0228
AGPS	0.72	5.27E-03	0.0190
GFER	0.72	1.80E-02	0.0428
ENST000002941890	0.72	8.23E-03	0.0252
ECD	0.72	9.10E-03	0.0269
KIF5B	0.72	6.66E-03	0.0221
ACTR3	0.72	6.89E-03	0.0226
FIG4	0.72	1.18E-02	0.0320
ATP5MC2	0.72	7.86E-03	0.0245
BCL9	0.72	1.12E-02	0.0309
LRPPRC	0.72	1.43E-02	0.0364
RNF6	0.72	1.43E-02	0.0364
UCHL3	0.72	6.16E-03	0.0210
SPICE1	0.72	5.15E-03	0.0188
CXorf40B	0.72	1.86E-02	0.0438
USP30	0.72	1.73E-02	0.0415
CAMK2D	0.72	1.71E-02	0.0411
MYEF2	0.72	1.37E-02	0.0352
RBM7	0.72	7.73E-03	0.0242
SLC15A4	0.72	1.33E-02	0.0346
SPOUT1	0.71	1.93E-02	0.0450
FOPNL	0.71	1.26E-02	0.0333
FXR1	0.71	6.88E-03	0.0226
MUM1	0.71	8.25E-03	0.0253
CUL4A	0.71	9.78E-03	0.0282
CEP78	0.71	5.10E-03	0.0187
SMARCA5	0.71	5.25E-03	0.0190

ACER3	0.71	6.67E-03	0.0221
TTI1	0.71	1.23E-02	0.0328
CCAR1	0.71	6.53E-03	0.0218
ELL	0.71	1.10E-02	0.0304
RPUSD4	0.71	5.45E-03	0.0195
ZNRD1	0.71	1.74E-02	0.0418
EIF4B	0.71	6.75E-03	0.0223
TMEM14B	0.71	9.78E-03	0.0282
SELENOH	0.71	6.86E-03	0.0225
COPS5	0.71	1.26E-02	0.0333
MAPK1IP1L	0.71	5.23E-03	0.0190
IWS1	0.71	6.18E-03	0.0210
SCARNA12	0.71	1.25E-02	0.0332
JAGN1	0.71	1.88E-02	0.0441
ENST000003049920	0.71	7.20E-03	0.0231
HDAC8	0.71	7.73E-03	0.0242
ENST000002064510	0.71	1.38E-02	0.0355
OSBPL3	0.71	1.13E-02	0.0311
CASP6	0.71	1.03E-02	0.0291
ILK	0.71	1.65E-02	0.0402
KIF1B	0.71	6.94E-03	0.0227
COX7A2L	0.71	5.23E-03	0.0190
NUDT22	0.71	1.04E-02	0.0293
AAGAB	0.71	1.01E-02	0.0288
CEBPZ	0.71	6.15E-03	0.0210
ENST000003140670	0.71	1.69E-02	0.0408
KLF9	0.70	7.80E-03	0.0243
COPS2	0.70	1.65E-02	0.0401
PUS3	0.70	9.01E-03	0.0267
NCOA3	0.70	5.43E-03	0.0194
LARP1	0.70	9.67E-03	0.0279
FUBP1	0.70	5.30E-03	0.0191
PET117	0.70	8.69E-03	0.0261
HENMT1	0.70	1.25E-02	0.0331
DIABLO	0.70	6.70E-03	0.0222
AKT2	0.70	9.88E-03	0.0284
ITPR2	0.70	9.89E-03	0.0284
BTBD6	0.70	1.05E-02	0.0294
PPTC7	0.70	6.14E-03	0.0210
ENST000002765202	0.70	8.85E-03	0.0264
MZT2B	0.70	2.05E-02	0.0470
SUCLG1	0.70	8.72E-03	0.0262
SELENOT	0.70	6.02E-03	0.0207
AUP1	0.70	2.19E-02	0.0492
COASY	0.70	1.31E-02	0.0342
EIF4G2	0.70	2.22E-02	0.0497
NDUFA5	0.70	9.25E-03	0.0271
PMPCB	0.70	5.83E-03	0.0203
ST3GAL3	0.70	1.84E-02	0.0436
TRIM65	0.69	1.58E-02	0.0391

FGFR1OP	0.69	1.07E-02	0.0298
TGDS	0.69	1.79E-02	0.0425
AFTPH	0.69	8.16E-03	0.0251
BAG2	0.69	8.43E-03	0.0256
TANGO6	0.69	1.07E-02	0.0299
ELOC	0.69	1.68E-02	0.0407
MRPL2	0.69	1.91E-02	0.0446
SENP2	0.69	1.54E-02	0.0383
GRHPR	0.69	1.89E-02	0.0443
RECQL	0.69	7.19E-03	0.0231
MEMO1	0.69	7.36E-03	0.0234
C22orf46	0.69	1.95E-02	0.0454
BRK1	0.69	1.16E-02	0.0315
UBE2F	0.69	1.27E-02	0.0334
APIP	0.69	1.76E-02	0.0421
ENST000003680260	0.69	1.68E-02	0.0407
COX7C	0.69	7.38E-03	0.0235
PLAA	0.69	8.45E-03	0.0256
SC5D	0.69	7.50E-03	0.0237
DNAJC7	0.69	1.35E-02	0.0349
SIL1	0.69	1.56E-02	0.0387
LRRFIP1	0.68	8.46E-03	0.0257
ENST000002288251	0.68	6.64E-03	0.0221
C11orf98	0.68	9.38E-03	0.0273
PRDX6	0.68	1.16E-02	0.0316
DHX16	0.68	6.44E-03	0.0216
INPPL1	0.68	1.48E-02	0.0372
FBXO4	0.68	1.69E-02	0.0408
CUTC	0.68	8.84E-03	0.0264
METTL15	0.68	1.33E-02	0.0345
EDEM1	0.68	6.19E-03	0.0211
TMED9	0.68	1.68E-02	0.0407
ENST000003115021	0.68	9.25E-03	0.0271
POLR2B	0.68	7.04E-03	0.0229
GFPT1	0.68	2.07E-02	0.0473
AGPAT3	0.68	1.25E-02	0.0332
TAF1D	0.68	8.66E-03	0.0260
SNAP47	0.68	8.18E-03	0.0251
AATF	0.68	6.67E-03	0.0221
COPE	0.68	7.32E-03	0.0234
USP22	0.68	1.58E-02	0.0390
RPS11	0.68	8.22E-03	0.0252
SAMM50	0.68	7.40E-03	0.0235
PLRG1	0.68	7.54E-03	0.0238
ENST000003521711	0.68	9.62E-03	0.0278
ZNF195	0.68	1.29E-02	0.0339
ENST000002789030	0.67	1.20E-02	0.0324
SMARCC1	0.67	7.00E-03	0.0228
AMZ2	0.67	7.96E-03	0.0247
ELOVL1	0.67	1.22E-02	0.0326

ERCC8	0.67	7.59E-03	0.0239
TUSC2	0.67	1.31E-02	0.0342
HNRNPD	0.67	9.31E-03	0.0272
C8orf59	0.67	6.48E-03	0.0217
LUC7L2	0.67	8.60E-03	0.0259
BUB3	0.67	6.25E-03	0.0212
NOP2	0.67	7.19E-03	0.0231
ENST000000021650	0.67	1.54E-02	0.0383
ATXN2L	0.67	9.72E-03	0.0281
PDSS2	0.67	1.08E-02	0.0301
PAIP2	0.67	1.62E-02	0.0396
FERMT3	0.67	1.02E-02	0.0289
NADK	0.67	1.59E-02	0.0392
ETAA1	0.67	7.87E-03	0.0245
AC091057.1	0.67	1.58E-02	0.0390
PNRC2	0.67	7.14E-03	0.0230
ABCD3	0.67	1.75E-02	0.0419
CCDC12	0.67	1.34E-02	0.0348
ING1	0.67	9.32E-03	0.0272
ENST000001993890	0.67	1.12E-02	0.0308
MRPS25	0.67	9.38E-03	0.0273
CIAPIN1	0.66	8.16E-03	0.0251
SVIP	0.66	7.70E-03	0.0242
CCDC59	0.66	8.77E-03	0.0262
PDIA3	0.66	1.99E-02	0.0459
ENST000003409410	0.66	1.72E-02	0.0414
ELAC2	0.66	7.42E-03	0.0236
PLPBP	0.66	7.91E-03	0.0246
JTB	0.66	1.08E-02	0.0301
NDUFA10	0.66	7.18E-03	0.0231
KRT10	0.66	1.39E-02	0.0356
ENST000003370180	0.66	9.04E-03	0.0267
ENST000003044340	0.66	1.14E-02	0.0313
INTS11	0.66	1.16E-02	0.0316
NDUFB9	0.66	7.99E-03	0.0247
ENST000003129161	0.66	7.09E-03	0.0230
ENST000003062701	0.66	1.39E-02	0.0356
TSNAX	0.66	7.09E-03	0.0230
APEH	0.66	1.18E-02	0.0320
SLC25A12	0.66	2.12E-02	0.0481
ANAPC15	0.66	1.62E-02	0.0397
PANK3	0.65	1.09E-02	0.0303
GTF2F1	0.65	1.59E-02	0.0392
NSFL1C	0.65	8.29E-03	0.0253
CRKL	0.65	9.61E-03	0.0278
GAN	0.65	7.12E-03	0.0230
PI4K2A	0.65	1.15E-02	0.0314
ANXA7	0.65	8.41E-03	0.0256
MEPCE	0.65	1.13E-02	0.0310
37500	0.65	1.42E-02	0.0362

MIS18BP1	0.65	1.48E-02	0.0371
RAB5A	0.65	1.71E-02	0.0412
SEPHS2	0.65	8.82E-03	0.0263
LRIG1	0.65	8.99E-03	0.0266
ZMAT2	0.65	1.15E-02	0.0314
ADIPOR2	0.65	8.09E-03	0.0250
LRRC47	0.65	2.22E-02	0.0497
PRRC2A	0.65	1.35E-02	0.0350
RPA2	0.65	7.86E-03	0.0245
RNFT2	0.65	2.04E-02	0.0468
SNHG20	0.65	1.51E-02	0.0377
WDCP	0.64	9.95E-03	0.0285
ANAPC7	0.64	1.14E-02	0.0313
ZNF273	0.64	9.13E-03	0.0269
FEM1A	0.64	1.83E-02	0.0434
AC012360.3	0.64	1.73E-02	0.0415
ALG9	0.64	9.09E-03	0.0268
PDK1	0.64	1.09E-02	0.0302
MAGT1	0.64	1.41E-02	0.0360
ZNF85	0.64	1.09E-02	0.0303
MRPL53	0.64	1.46E-02	0.0369
REXO2	0.64	1.88E-02	0.0441
GID8	0.64	7.54E-03	0.0238
MIS12	0.64	1.09E-02	0.0303
PREB	0.64	1.56E-02	0.0387
BBS7	0.64	1.53E-02	0.0382
RNF141	0.64	1.63E-02	0.0399
CNOT6	0.64	8.36E-03	0.0255
SERF2	0.64	7.96E-03	0.0247
TSR2	0.64	1.38E-02	0.0355
FAS	0.63	1.17E-02	0.0317
RPL41	0.63	1.61E-02	0.0396
ZBTB8OS	0.63	1.19E-02	0.0322
ENST000003450460	0.63	2.03E-02	0.0466
METTL9	0.63	1.10E-02	0.0305
WDR77	0.63	1.24E-02	0.0329
H2AFY	0.63	8.06E-03	0.0249
CFAP97	0.63	1.82E-02	0.0430
RPS13	0.63	1.42E-02	0.0362
ZNF212	0.63	2.09E-02	0.0477
SEPT7	0.63	1.30E-02	0.0340
CHD4	0.63	1.29E-02	0.0339
CSK	0.63	1.10E-02	0.0305
PDCD6	0.63	1.15E-02	0.0313
RPL23	0.63	8.27E-03	0.0253
CELF1	0.63	1.14E-02	0.0311
RNMT	0.63	1.08E-02	0.0301
C12orf4	0.63	1.29E-02	0.0339
FLII	0.63	1.25E-02	0.0332
RPL36A	0.62	8.62E-03	0.0259

ENST000003814010	0.62	1.35E-02	0.0350
NSL1	0.62	1.42E-02	0.0363
RPS5	0.62	1.32E-02	0.0345
IMMP2L	0.62	1.69E-02	0.0409
SLC25A25	0.62	1.53E-02	0.0381
ENST000003006510	0.62	1.02E-02	0.0288
FAM156A	0.62	1.14E-02	0.0311
ERLIN2	0.62	1.36E-02	0.0352
PCID2	0.62	1.30E-02	0.0341
ZNF770	0.62	1.10E-02	0.0304
TRIM28	0.62	1.87E-02	0.0440
ENST000003146160	0.62	9.28E-03	0.0272
ARL4A	0.62	1.53E-02	0.0381
NF2	0.62	9.31E-03	0.0272
TCAF1	0.62	1.26E-02	0.0334
COX18	0.62	1.00E-02	0.0286
TCTN3	0.62	1.68E-02	0.0407
ENST000003249321	0.62	8.94E-03	0.0266
RBM28	0.62	9.65E-03	0.0279
HACD3	0.61	1.34E-02	0.0347
MATR3	0.61	1.08E-02	0.0301
ZNF267	0.61	1.65E-02	0.0402
SLC30A5	0.61	1.40E-02	0.0358
AGO2	0.61	1.07E-02	0.0299
HADHB	0.61	1.00E-02	0.0286
COPB1	0.61	1.17E-02	0.0318
ZCCHC10	0.61	1.04E-02	0.0294
ECI1	0.61	1.38E-02	0.0354
SPPL2A	0.61	1.82E-02	0.0430
CNOT7	0.61	1.42E-02	0.0363
SACS	0.61	2.07E-02	0.0473
RBCK1	0.61	1.49E-02	0.0373
SKP1	0.61	9.84E-03	0.0283
ENST000002588862	0.61	1.50E-02	0.0376
NSUN5	0.61	1.71E-02	0.0412
PEX2	0.61	9.26E-03	0.0271
NUP85	0.61	1.14E-02	0.0312
NDUFAF4	0.61	1.69E-02	0.0408
ARL5B	0.61	1.07E-02	0.0300
TEX2	0.61	1.12E-02	0.0309
TRA2B	0.61	1.89E-02	0.0443
SETD7	0.61	1.28E-02	0.0337
GUF1	0.61	1.70E-02	0.0410
LYRM1	0.60	1.08E-02	0.0301
IFI16	0.60	1.33E-02	0.0345
ENST000002634610	0.60	2.00E-02	0.0462
AC026464.4	0.60	2.05E-02	0.0470
IL23A	0.60	1.48E-02	0.0372
SH3GL1	0.60	1.73E-02	0.0414
SMIM12	0.60	2.05E-02	0.0470

RIOK3	0.60	1.13E-02	0.0310
VKORC1L1	0.60	1.21E-02	0.0325
FKTN	0.60	1.33E-02	0.0345
CHTF8	0.60	9.72E-03	0.0281
NDUFB5	0.60	1.20E-02	0.0323
KLHL18	0.60	1.58E-02	0.0390
ANAPC16	0.60	9.69E-03	0.0280
TFG	0.60	1.67E-02	0.0405
MOB1A	0.60	1.23E-02	0.0328
TSG101	0.60	2.20E-02	0.0494
DNAJB6	0.60	1.46E-02	0.0369
FAM168B	0.60	1.29E-02	0.0338
DNAJC14	0.59	1.08E-02	0.0302
OTUD5	0.59	1.29E-02	0.0338
SETD3	0.59	1.15E-02	0.0313
STAU1	0.59	1.15E-02	0.0314
MPC2	0.59	2.14E-02	0.0484
TMEM173	0.59	1.08E-02	0.0302
TARDBP	0.59	1.44E-02	0.0365
ACBD6	0.59	1.05E-02	0.0296
YME1L1	0.59	1.55E-02	0.0385
ENST000002546301	0.59	1.65E-02	0.0401
UBE2V1	0.59	1.69E-02	0.0408
KPNA6	0.59	1.31E-02	0.0342
BAZ1A	0.59	1.17E-02	0.0318
MAP2K2	0.59	1.23E-02	0.0328
AASDHPPT	0.59	1.43E-02	0.0364
CFLAR	0.59	1.45E-02	0.0367
SPAG7	0.58	1.23E-02	0.0328
RTF2	0.58	1.12E-02	0.0308
RLF	0.58	1.11E-02	0.0306
PIGX	0.58	1.17E-02	0.0317
ZNF410	0.58	1.67E-02	0.0406
EDF1	0.58	1.39E-02	0.0357
CSDE1	0.58	1.50E-02	0.0376
YTHDF2	0.58	1.33E-02	0.0345
KATNBL1	0.58	1.12E-02	0.0309
SLC39A7	0.58	1.57E-02	0.0388
TMEM183A	0.58	1.28E-02	0.0337
IGFBP4	0.58	1.95E-02	0.0454
TBK1	0.58	1.21E-02	0.0324
GTF3C2	0.58	1.73E-02	0.0414
ZFYVE9	0.58	1.90E-02	0.0444
TMED4	0.58	1.45E-02	0.0368
CFDP1	0.58	1.25E-02	0.0331
SLF1	0.58	1.18E-02	0.0320
SNX17	0.58	1.71E-02	0.0411
CDK9	0.58	1.31E-02	0.0341
SRGAP2B	0.58	1.50E-02	0.0376
SLC35E1	0.57	1.46E-02	0.0368

TMBIM6	0.57	1.69E-02	0.0408
BRD7	0.57	1.38E-02	0.0354
TADA2A	0.57	1.53E-02	0.0380
GTF2A1	0.57	1.37E-02	0.0353
SPG21	0.57	1.24E-02	0.0329
B3GAT3	0.57	2.22E-02	0.0497
CDC5L	0.57	2.10E-02	0.0479
MFSD14A	0.57	1.53E-02	0.0382
INTS9	0.57	1.89E-02	0.0443
DDX27	0.57	1.52E-02	0.0380
ENST000002659860	0.57	1.62E-02	0.0396
EEF1B2	0.57	1.86E-02	0.0438
FAF2	0.57	1.25E-02	0.0331
ENST000002226900	0.57	1.49E-02	0.0374
RPS7	0.57	1.80E-02	0.0428
AP3M1	0.57	1.42E-02	0.0362
CBWD5	0.57	1.23E-02	0.0328
PTCD3	0.57	2.02E-02	0.0465
OSBPL9	0.56	1.21E-02	0.0325
NMNAT1	0.56	2.04E-02	0.0468
THAP1	0.56	1.50E-02	0.0376
ENST000002617140	0.56	1.76E-02	0.0420
OTUB1	0.56	1.56E-02	0.0386
HMGN4	0.56	1.57E-02	0.0388
CEBPG	0.56	1.55E-02	0.0384
PDCD2	0.56	1.92E-02	0.0448
HINT1	0.56	2.11E-02	0.0480
ARL10	0.56	2.17E-02	0.0489
FAF1	0.56	1.24E-02	0.0330
RPL15	0.56	1.33E-02	0.0345
NPEPPS	0.56	2.13E-02	0.0483
ENST000002553050	0.56	1.29E-02	0.0338
PSENEN	0.56	1.74E-02	0.0417
CS	0.56	1.70E-02	0.0410
PITPNA	0.56	1.68E-02	0.0406
SPCS2	0.55	2.21E-02	0.0496
DYNC1LI1	0.55	1.71E-02	0.0411
HMGXB3	0.55	1.51E-02	0.0378
ENST000002569352	0.55	1.82E-02	0.0430
AP4E1	0.55	1.59E-02	0.0392
RIF1	0.55	1.92E-02	0.0449
OSGIN2	0.55	1.54E-02	0.0382
MEX3C	0.55	1.57E-02	0.0388
GNE	0.55	1.46E-02	0.0368
TNPO1	0.55	1.50E-02	0.0376
RPF1	0.55	1.71E-02	0.0412
TBCE	0.55	2.23E-02	0.0498
BLOC1S5	0.55	1.36E-02	0.0351
TRNAU1AP	0.55	1.58E-02	0.0389
RPL12	0.55	1.34E-02	0.0348

TRIOBP	0.55	2.22E-02	0.0498
POLG	0.55	1.39E-02	0.0357
SRFBP1	0.55	1.80E-02	0.0428
LINC01215	0.55	1.62E-02	0.0397
PCBP1	0.55	2.05E-02	0.0470
MEAF6	0.55	1.87E-02	0.0441
PPP6R3	0.54	1.69E-02	0.0409
FAM98B	0.54	1.99E-02	0.0459
PRPF38A	0.54	1.45E-02	0.0367
MANEA	0.54	2.17E-02	0.0489
PPP1CB	0.54	1.82E-02	0.0432
DCTN2	0.54	1.57E-02	0.0388
GATC	0.54	1.73E-02	0.0416
ENST000002864481	0.54	2.19E-02	0.0493
UBE3A	0.54	1.60E-02	0.0393
TNFAIP1	0.53	2.23E-02	0.0498
C8orf76	0.53	2.16E-02	0.0489
UBE2Q1	0.53	1.66E-02	0.0403
LIAS	0.53	2.06E-02	0.0471
RSRC2	0.53	2.20E-02	0.0494
CDK5RAP2	0.52	1.67E-02	0.0405
CWF19L1	0.52	1.57E-02	0.0389
ABT1	0.52	1.99E-02	0.0459
ATF2	0.52	1.68E-02	0.0407
ENST000002196891	0.52	2.19E-02	0.0492
TRAPPC2L	0.52	1.92E-02	0.0448
CD2AP	0.52	1.60E-02	0.0393
ENST000003358952	0.52	1.94E-02	0.0451
UBTF	0.51	2.09E-02	0.0477
MT-ND4L	0.51	2.19E-02	0.0493
DCUN1D1	0.51	2.17E-02	0.0490
DNAJC1	0.51	1.77E-02	0.0422
DYNLL2	0.51	1.92E-02	0.0448
ATG4C	0.51	1.79E-02	0.0426
CDKN2D	0.51	1.96E-02	0.0455
CDV3	0.51	1.75E-02	0.0418
LTA4H	0.51	2.16E-02	0.0488
HPS4	0.51	1.90E-02	0.0445
TARBP1	0.51	1.98E-02	0.0459
RAP1B	0.51	2.12E-02	0.0481
UBA3	0.51	1.82E-02	0.0430
CRCP	0.50	1.98E-02	0.0458
ADSS	0.50	2.09E-02	0.0476
OARD1	0.50	1.86E-02	0.0438
ENST000002726383	0.49	2.15E-02	0.0486
MRPL50	0.49	2.22E-02	0.0497
RNGTT	0.48	2.04E-02	0.0468
WDR55	0.48	2.15E-02	0.0486
TRAPPC10	-0.47	2.22E-02	0.0497
UTP14C	-0.48	2.19E-02	0.0493

CCDC93	-0.48	2.16E-02	0.0488
APC	-0.48	2.15E-02	0.0486
IKZF1	-0.48	1.99E-02	0.0460
ORC4	-0.49	2.03E-02	0.0467
DCTN1	-0.49	2.16E-02	0.0488
DBNL	-0.49	2.18E-02	0.0492
MED23	-0.49	2.01E-02	0.0462
ZMYM5	-0.50	2.23E-02	0.0499
GOLGA4	-0.50	2.10E-02	0.0478
ZNF507	-0.50	1.99E-02	0.0460
ENST000003526890	-0.50	2.04E-02	0.0468
PIK3C3	-0.51	2.06E-02	0.0472
ANXA11	-0.51	2.23E-02	0.0499
VEZF1	-0.51	2.20E-02	0.0494
RSBN1L	-0.51	2.22E-02	0.0497
CHMP1B	-0.51	1.96E-02	0.0455
EBLN3P	-0.51	1.71E-02	0.0412
GOLGB1	-0.51	2.12E-02	0.0481
ENST000000525690	-0.51	1.90E-02	0.0445
HIPK3	-0.52	1.97E-02	0.0457
CCNDBP1	-0.52	1.70E-02	0.0410
NFATC3	-0.52	2.18E-02	0.0491
FAM217B	-0.52	1.85E-02	0.0436
EIF4EBP2	-0.53	2.02E-02	0.0466
CHD9	-0.53	1.96E-02	0.0455
ENST000003267930	-0.53	1.61E-02	0.0395
MFSD4B	-0.53	1.93E-02	0.0450
ATG16L2	-0.53	1.97E-02	0.0456
KMT2E	-0.53	1.82E-02	0.0432
TRBV7-2	-0.53	1.97E-02	0.0456
HEXA	-0.54	1.55E-02	0.0384
ZNF407	-0.54	1.59E-02	0.0392
SNX13	-0.54	1.46E-02	0.0368
GABARAP	-0.54	2.22E-02	0.0498
PAPOLA	-0.54	1.40E-02	0.0358
ENST000002630730	-0.54	1.56E-02	0.0387
KDM4C	-0.54	1.73E-02	0.0416
CEP85L	-0.54	1.44E-02	0.0366
PWWP2A	-0.55	1.76E-02	0.0420
IGBP1	-0.55	2.12E-02	0.0481
ANKHD1	-0.55	1.52E-02	0.0379
WASHC4	-0.55	1.81E-02	0.0430
MTO1	-0.55	1.83E-02	0.0433
EPM2AIP1	-0.55	1.43E-02	0.0364
SLC35A1	-0.56	2.07E-02	0.0473
CLN3	-0.56	1.81E-02	0.0430
PHF3	-0.56	2.05E-02	0.0470
PCYT1A	-0.56	1.45E-02	0.0367
SHC1	-0.56	2.13E-02	0.0482
TMEM59	-0.56	1.70E-02	0.0409

ZNF3	-0.56	1.50E-02	0.0376
VPS35L	-0.56	1.94E-02	0.0452
DUSP22	-0.56	1.73E-02	0.0415
NR2C1	-0.57	1.48E-02	0.0372
KDM2A	-0.57	1.44E-02	0.0366
SEL1L3	-0.57	1.24E-02	0.0330
CDK5RAP3	-0.57	1.68E-02	0.0407
MBNL3	-0.57	1.57E-02	0.0389
SUGT1	-0.57	2.19E-02	0.0493
MYSM1	-0.57	1.32E-02	0.0343
ZNF252P	-0.57	1.20E-02	0.0323
BRAF	-0.57	1.21E-02	0.0325
MIGA1	-0.57	1.22E-02	0.0326
ZSWIM6	-0.57	2.04E-02	0.0468
SLC20A2	-0.57	1.74E-02	0.0418
RBM41	-0.58	1.22E-02	0.0327
POGLUT1	-0.58	2.13E-02	0.0484
USP53	-0.58	1.50E-02	0.0376
XAB2	-0.58	1.58E-02	0.0389
TGOLN2	-0.58	1.45E-02	0.0368
SLC38A9	-0.59	1.69E-02	0.0408
MKRN1	-0.59	1.45E-02	0.0367
INTS8	-0.59	2.18E-02	0.0491
SUMF1	-0.59	2.21E-02	0.0496
PIP4K2A	-0.59	1.82E-02	0.0432
KMT5B	-0.59	1.21E-02	0.0325
MSL3	-0.59	1.66E-02	0.0403
NFX1	-0.59	2.03E-02	0.0466
RPS6KC1	-0.59	1.34E-02	0.0347
AGA	-0.59	1.34E-02	0.0348
LEMD3	-0.59	1.53E-02	0.0381
DMAP1	-0.59	1.20E-02	0.0323
CCDC142	-0.60	1.92E-02	0.0448
ILKAP	-0.60	1.27E-02	0.0336
ZNF780A	-0.60	1.05E-02	0.0295
SETX	-0.60	1.00E-02	0.0286
CCT6P3	-0.60	2.13E-02	0.0484
PAK2	-0.60	1.63E-02	0.0397
ZNF430	-0.60	1.09E-02	0.0304
CYHR1	-0.60	2.05E-02	0.0470
FMR1	-0.60	1.79E-02	0.0426
PIGG	-0.60	1.11E-02	0.0306
TMEM131	-0.61	1.05E-02	0.0294
SAFB2	-0.61	1.87E-02	0.0439
C9orf78	-0.61	1.24E-02	0.0330
ZNF32	-0.61	1.38E-02	0.0355
ZFAND6	-0.61	9.99E-03	0.0286
CNKSR2	-0.61	1.30E-02	0.0341
TAF1	-0.61	9.69E-03	0.0280
ABRAXAS1	-0.61	1.84E-02	0.0436

AP001486.2	-0.61	1.91E-02	0.0446
GABPB1-AS1	-0.61	1.92E-02	0.0448
ATXN1L	-0.61	9.82E-03	0.0283
PRKCQ	-0.61	1.31E-02	0.0343
CEP104	-0.62	1.22E-02	0.0326
C18orf32	-0.62	1.62E-02	0.0397
TARSL2	-0.62	1.89E-02	0.0443
WDR19	-0.62	1.89E-02	0.0442
L3MBTL1	-0.62	2.07E-02	0.0474
SPG7	-0.62	1.52E-02	0.0378
ACAP3	-0.62	1.47E-02	0.0371
ESYT1	-0.62	1.63E-02	0.0397
TBCD	-0.62	1.09E-02	0.0303
TRIM35	-0.62	1.02E-02	0.0290
ENST000003079681	-0.62	1.29E-02	0.0339
SP110	-0.62	1.38E-02	0.0356
MORN3	-0.62	2.17E-02	0.0489
RNF2	-0.62	1.25E-02	0.0332
ATG14	-0.62	1.45E-02	0.0367
ZNF217	-0.62	1.38E-02	0.0355
GBA2	-0.63	1.14E-02	0.0311
C1QTNF6	-0.63	1.85E-02	0.0436
RALGAPB	-0.63	9.37E-03	0.0273
GIT2	-0.63	8.77E-03	0.0262
SMARCC2	-0.63	1.12E-02	0.0309
EP400	-0.63	1.11E-02	0.0306
HERC4	-0.63	1.02E-02	0.0289
SERINC3	-0.63	8.58E-03	0.0259
DCK	-0.63	1.79E-02	0.0425
EHMT1	-0.63	1.02E-02	0.0288
SH3GLB1	-0.63	1.27E-02	0.0335
PPWD1	-0.63	8.80E-03	0.0263
MORC4	-0.63	7.97E-03	0.0247
PDCD6IP	-0.63	1.19E-02	0.0322
ULK3	-0.63	1.77E-02	0.0422
ZNF468	-0.64	1.03E-02	0.0292
SLC33A1	-0.64	1.06E-02	0.0297
MDM4	-0.64	9.17E-03	0.0270
CHD2	-0.64	9.16E-03	0.0269
KDM5A	-0.64	7.69E-03	0.0241
DIAPH1	-0.64	1.72E-02	0.0412
UBAC2	-0.64	1.23E-02	0.0329
UBE2H	-0.64	9.46E-03	0.0275
MINDY3	-0.64	1.61E-02	0.0395
DET1	-0.64	2.21E-02	0.0496
ANKRD36C	-0.64	8.31E-03	0.0254
ZNF254	-0.64	1.79E-02	0.0426
SLC35E3	-0.64	8.16E-03	0.0251
SHPRH	-0.64	8.72E-03	0.0262
NUTM2A-AS1	-0.64	2.22E-02	0.0498

ZNF567	-0.64	8.57E-03	0.0259
C6orf89	-0.64	1.30E-02	0.0340
ENST000002933730	-0.64	1.01E-02	0.0288
MOAP1	-0.64	1.32E-02	0.0343
ERAP1	-0.64	1.99E-02	0.0460
THBS3	-0.64	1.73E-02	0.0415
MRTFB	-0.64	1.51E-02	0.0377
NISCH	-0.64	1.62E-02	0.0397
KIFAP3	-0.64	1.01E-02	0.0288
ZNF527	-0.64	1.14E-02	0.0313
FOXJ3	-0.65	9.24E-03	0.0271
MRFAP1L1	-0.65	1.40E-02	0.0359
VPS18	-0.65	1.17E-02	0.0318
TAB2	-0.65	1.36E-02	0.0352
GABPB2	-0.65	7.51E-03	0.0238
CDR2	-0.65	1.01E-02	0.0287
RAB5C	-0.65	1.46E-02	0.0369
ENST000003987520	-0.65	1.80E-02	0.0428
PRR5	-0.65	1.95E-02	0.0454
BTN2A1	-0.65	2.02E-02	0.0465
TRMT10B	-0.65	1.62E-02	0.0397
ZNF287	-0.65	1.26E-02	0.0334
CCNL2	-0.65	1.88E-02	0.0441
NBEAL2	-0.66	1.75E-02	0.0419
MOSMO	-0.66	8.61E-03	0.0259
LRCH3	-0.66	1.13E-02	0.0311
WDTC1	-0.66	1.88E-02	0.0441
TMEM189	-0.66	1.82E-02	0.0431
ALG11	-0.66	1.24E-02	0.0329
ICAM3	-0.66	1.16E-02	0.0315
MFSD1	-0.66	1.89E-02	0.0443
ENST000002678900	-0.66	1.26E-02	0.0333
ZBTB14	-0.66	1.08E-02	0.0302
PIGC	-0.66	2.09E-02	0.0476
CWF19L2	-0.66	9.26E-03	0.0271
TAS2R30	-0.66	1.88E-02	0.0442
SRSF5	-0.66	1.38E-02	0.0354
TRAPPC3L	-0.66	1.91E-02	0.0446
PRKCH	-0.66	1.48E-02	0.0371
PXYLP1	-0.66	1.22E-02	0.0326
NEK9	-0.67	1.70E-02	0.0410
DIAPH2	-0.67	7.17E-03	0.0231
HYPK	-0.67	1.73E-02	0.0416
C6orf47	-0.67	1.15E-02	0.0315
ENST000002694450	-0.67	1.63E-02	0.0398
ZNF800	-0.67	1.07E-02	0.0299
ZNF440	-0.67	1.58E-02	0.0389
SDHAP1	-0.67	8.90E-03	0.0265
ENST000002371770	-0.67	2.13E-02	0.0483
INF2	-0.67	1.01E-02	0.0288

AP001372.2	-0.67	2.17E-02	0.0490
CUL9	-0.67	9.07E-03	0.0268
C12orf76	-0.67	1.21E-02	0.0325
MYO9B	-0.67	1.71E-02	0.0411
ANGEL2	-0.67	7.24E-03	0.0232
PKN2	-0.68	8.92E-03	0.0265
PARGP1	-0.68	9.43E-03	0.0274
ZNF445	-0.68	6.50E-03	0.0218
KLHL9	-0.68	6.73E-03	0.0223
TMEM42	-0.68	1.46E-02	0.0368
C1GALT1	-0.68	1.48E-02	0.0371
STXBP4	-0.68	1.15E-02	0.0314
NBPF14	-0.68	1.36E-02	0.0351
RBM45	-0.68	7.56E-03	0.0239
PAPOLG	-0.68	7.46E-03	0.0236
EFCAB14	-0.68	1.32E-02	0.0343
ZFP90	-0.68	8.34E-03	0.0254
ZNF354A	-0.68	1.24E-02	0.0330
GNB5	-0.68	1.50E-02	0.0376
AP4S1	-0.68	9.71E-03	0.0280
CNOT6L	-0.68	7.17E-03	0.0231
KIAA1328	-0.69	1.16E-02	0.0317
ENST000002982481	-0.69	2.12E-02	0.0481
ZNF283	-0.69	7.30E-03	0.0233
ARID1A	-0.69	1.10E-02	0.0304
ITGA5	-0.69	1.26E-02	0.0334
AC073073.2	-0.69	2.20E-02	0.0495
CYB5R1	-0.69	8.31E-03	0.0254
TRBC1	-0.69	9.03E-03	0.0267
UBA6-AS1	-0.69	7.43E-03	0.0236
COG3	-0.69	5.88E-03	0.0204
TES	-0.69	1.88E-02	0.0442
KLHDC2	-0.69	6.67E-03	0.0221
ENST000003343440	-0.69	9.02E-03	0.0267
SEPT7P2	-0.69	8.54E-03	0.0258
RBM48	-0.69	8.26E-03	0.0253
PHF12	-0.69	6.93E-03	0.0227
TNK2	-0.69	1.30E-02	0.0340
KRAS	-0.69	7.81E-03	0.0243
TNKS2	-0.69	2.03E-02	0.0467
SMG1	-0.69	2.20E-02	0.0494
TBC1D15	-0.70	1.09E-02	0.0303
NAPEPLD	-0.70	1.00E-02	0.0286
NPAT	-0.70	7.50E-03	0.0237
ENST000003413760	-0.70	7.66E-03	0.0241
NOS3	-0.70	2.06E-02	0.0472
AC055713.1	-0.70	1.71E-02	0.0412
FAM47E	-0.70	1.75E-02	0.0419
DCLRE1C	-0.70	8.02E-03	0.0248
NECAP2	-0.70	1.84E-02	0.0436

ENST000003360860	-0.70	1.68E-02	0.0406
LUC7L3	-0.70	8.61E-03	0.0259
ENST000002511080	-0.70	1.45E-02	0.0367
CLCN3	-0.70	6.69E-03	0.0222
ENST000002294650	-0.70	2.08E-02	0.0474
ENST000003159300	-0.70	8.17E-03	0.0251
CASD1	-0.70	6.48E-03	0.0217
ZNF623	-0.70	5.78E-03	0.0202
ENST000003077142	-0.70	1.38E-02	0.0355
ENST000002822724	-0.70	7.29E-03	0.0233
ENST000003250832	-0.70	1.47E-02	0.0370
ENST000002693910	-0.70	7.01E-03	0.0228
TEN1-CDK3	-0.70	1.75E-02	0.0419
VPS11	-0.71	1.07E-02	0.0299
FCHSD1	-0.71	8.75E-03	0.0262
STIM1	-0.71	1.01E-02	0.0288
ZNF605	-0.71	7.07E-03	0.0229
ENST000003110481	-0.71	9.12E-03	0.0269
ZNF720	-0.71	1.35E-02	0.0349
SECISBP2	-0.71	9.84E-03	0.0283
AL163051.2	-0.71	1.98E-02	0.0458
ENST000002639970	-0.71	1.67E-02	0.0406
SMAD4	-0.71	5.20E-03	0.0189
AL031595.2	-0.71	1.46E-02	0.0369
LPCAT4	-0.71	9.93E-03	0.0285
RGP1	-0.72	8.13E-03	0.0251
SNX15	-0.72	1.76E-02	0.0420
AC124312.4	-0.72	1.60E-02	0.0393
TFEB	-0.72	1.84E-02	0.0435
ZNFX1	-0.72	8.79E-03	0.0263
PHF2	-0.72	1.65E-02	0.0401
RECK	-0.72	1.64E-02	0.0400
AP1G2	-0.72	5.70E-03	0.0200
TRAM1	-0.72	1.11E-02	0.0307
OGA	-0.72	1.02E-02	0.0289
POLI	-0.72	1.71E-02	0.0411
STK17B	-0.72	5.92E-03	0.0205
NIPBL	-0.72	9.12E-03	0.0269
ENST000003344440	-0.72	1.09E-02	0.0303
YPEL5	-0.72	8.37E-03	0.0255
FLOT2	-0.72	2.00E-02	0.0460
ENST000002431891	-0.72	1.71E-02	0.0411
TUG1	-0.72	4.79E-03	0.0180
ACSF3	-0.72	1.75E-02	0.0419
PRORSD1P	-0.72	2.13E-02	0.0483
ARSK	-0.72	7.70E-03	0.0242
MLLT3	-0.72	1.99E-02	0.0460
NPEPL1	-0.72	1.26E-02	0.0333
ARL14EP	-0.73	1.11E-02	0.0307
FNDC3A	-0.73	1.94E-02	0.0451

TIAL1	-0.73	8.32E-03	0.0254
ZC3HAV1	-0.73	6.58E-03	0.0220
GANC	-0.73	5.55E-03	0.0197
OGFR	-0.73	2.03E-02	0.0467
ENST000003159701	-0.73	7.31E-03	0.0234
PAQR3	-0.73	8.47E-03	0.0257
TSC22D2	-0.73	1.43E-02	0.0364
INO80D	-0.73	5.67E-03	0.0200
HSD17B4	-0.73	8.90E-03	0.0265
ADAMTS4	-0.73	1.64E-02	0.0401
STX16	-0.73	1.03E-02	0.0292
KRBOX4	-0.73	1.54E-02	0.0383
ENST000003058772	-0.73	5.87E-03	0.0204
RAD52	-0.73	1.82E-02	0.0432
RAB5B	-0.73	1.29E-02	0.0339
AC003681.1	-0.73	1.06E-02	0.0297
CAPN7	-0.73	8.49E-03	0.0257
ABCA11P	-0.73	2.20E-02	0.0493
ZNF791	-0.73	7.78E-03	0.0243
ENST000003553541	-0.73	1.65E-02	0.0401
IDS	-0.73	2.15E-02	0.0487
TTC17	-0.73	5.89E-03	0.0204
PI4KAP2	-0.73	1.66E-02	0.0404
CCDC107	-0.74	9.97E-03	0.0285
ZNF721	-0.74	5.78E-03	0.0202
LINC01226	-0.74	7.62E-03	0.0240
MAPKAPK5-AS1	-0.74	1.79E-02	0.0426
MYLK3	-0.74	2.02E-02	0.0465
ZNF137P	-0.74	1.08E-02	0.0302
ENST000002546050	-0.74	2.08E-02	0.0475
PTBP2	-0.74	1.60E-02	0.0394
UBXN1	-0.74	7.61E-03	0.0240
C5orf63	-0.74	1.04E-02	0.0294
ICAM2	-0.74	1.19E-02	0.0322
BTBD7	-0.74	6.97E-03	0.0228
BBS10	-0.74	7.24E-03	0.0232
LDB1	-0.74	5.54E-03	0.0196
SLC10A3	-0.74	1.88E-02	0.0441
ACSL6	-0.74	7.12E-03	0.0230
YIPF4	-0.74	4.88E-03	0.0182
ZNF277	-0.74	5.22E-03	0.0190
SLC18B1	-0.74	6.60E-03	0.0220
HCFC2	-0.74	4.56E-03	0.0175
FAM111A-DT	-0.74	1.30E-02	0.0341
USP15	-0.74	1.42E-02	0.0363
ZNF37BP	-0.74	1.11E-02	0.0307
MON2	-0.74	4.82E-03	0.0180
CDK13	-0.74	5.80E-03	0.0202
FNBP4	-0.74	4.89E-03	0.0182
ZBED5	-0.74	4.80E-03	0.0180

HNRNPH2	-0.74	9.52E-03	0.0276
ZNF155	-0.74	1.75E-02	0.0418
BRI3	-0.74	1.54E-02	0.0383
ELMO1	-0.74	6.07E-03	0.0208
CRLF3	-0.75	6.02E-03	0.0207
SAT2	-0.75	1.20E-02	0.0324
FAM160A2	-0.75	1.25E-02	0.0331
UBN2	-0.75	6.05E-03	0.0208
PIP4P2	-0.75	1.85E-02	0.0436
CYP4A22-AS1	-0.75	1.97E-02	0.0457
38777	-0.75	4.84E-03	0.0181
AK9	-0.75	1.95E-02	0.0452
FAM200B	-0.75	6.52E-03	0.0218
OGG1	-0.75	9.83E-03	0.0283
UCKL1	-0.75	2.06E-02	0.0472
RABGAP1	-0.75	7.60E-03	0.0239
PIGK	-0.75	5.12E-03	0.0187
ENST000002981590	-0.75	1.67E-02	0.0405
LINC01562	-0.75	1.08E-02	0.0302
ENST000003243060	-0.75	1.29E-02	0.0339
UVSSA	-0.75	6.03E-03	0.0207
LINC00294	-0.75	1.96E-02	0.0455
PIK3CD	-0.75	1.48E-02	0.0371
WAC-AS1	-0.75	7.76E-03	0.0243
PPP1R21	-0.75	1.77E-02	0.0421
CALCOCO2	-0.76	4.59E-03	0.0176
ANKRD49	-0.76	5.63E-03	0.0198
PTPRE	-0.76	1.08E-02	0.0301
LINC00662	-0.76	1.23E-02	0.0328
ENST00000639751	-0.76	6.26E-03	0.0212
SETD2	-0.76	5.64E-03	0.0199
ZNF891	-0.76	7.11E-03	0.0230
ZFC3H1	-0.76	4.89E-03	0.0182
CLK1	-0.76	9.26E-03	0.0271
DNAJC16	-0.76	4.79E-03	0.0180
WDR11	-0.76	2.03E-02	0.0467
PARP16	-0.76	1.80E-02	0.0428
NEK1	-0.76	1.21E-02	0.0326
RABL2B	-0.76	1.46E-02	0.0368
MTM1	-0.76	2.06E-02	0.0472
PRKAG2	-0.76	8.97E-03	0.0266
AC099811.1	-0.76	1.31E-02	0.0341
ITSN2	-0.76	4.20E-03	0.0167
DENND1B	-0.76	9.94E-03	0.0285
TDRD3	-0.76	4.44E-03	0.0172
PURA	-0.76	4.28E-03	0.0168
AP000873.2	-0.76	1.98E-02	0.0459
STK4	-0.77	1.02E-02	0.0289
CSAD	-0.77	1.22E-02	0.0326
PCF11	-0.77	4.29E-03	0.0169

MAP4K2	-0.77	7.73E-03	0.0242
WDR5B	-0.77	1.44E-02	0.0366
BLZF1	-0.77	9.99E-03	0.0286
ENST000002615560	-0.77	1.43E-02	0.0364
FBXO15	-0.77	1.98E-02	0.0459
COQ10A	-0.77	9.12E-03	0.0269
TRAV5	-0.77	1.66E-02	0.0403
ELMOD3	-0.77	8.76E-03	0.0262
SRD5A3-AS1	-0.77	2.08E-02	0.0474
C16orf70	-0.77	1.54E-02	0.0383
LINC01568	-0.77	1.44E-02	0.0366
DDX5	-0.77	7.02E-03	0.0228
AC090114.2	-0.77	1.88E-02	0.0442
ELK3	-0.77	4.71E-03	0.0178
PIAS1	-0.77	6.13E-03	0.0210
ARL6IP5	-0.77	7.63E-03	0.0240
MOB3C	-0.77	6.53E-03	0.0218
ENST000002620511	-0.77	6.58E-03	0.0219
PCDHA4	-0.77	1.10E-02	0.0304
MIR100HG	-0.77	1.96E-02	0.0455
LRRC8A	-0.77	5.09E-03	0.0187
RGS14	-0.77	1.12E-02	0.0309
ZNF234	-0.78	5.37E-03	0.0193
ZNF169	-0.78	6.29E-03	0.0213
C8orf34-AS1	-0.78	1.49E-02	0.0374
HMBOX1	-0.78	1.44E-02	0.0365
ARHGEF1	-0.78	1.50E-02	0.0376
MYLK	-0.78	2.07E-02	0.0473
AC007998.4	-0.78	8.34E-03	0.0254
TAOK1	-0.78	4.21E-03	0.0167
ZKSCAN4	-0.78	9.95E-03	0.0285
ZNF529	-0.78	4.37E-03	0.0171
OPRM1	-0.78	1.98E-02	0.0459
FAM172A	-0.78	3.99E-03	0.0161
ARPC3	-0.78	6.37E-03	0.0214
WASH5P	-0.78	7.68E-03	0.0241
IKZF5	-0.78	4.92E-03	0.0183
BAHD1	-0.78	1.17E-02	0.0317
ZEB1	-0.78	9.40E-03	0.0274
HCG18	-0.78	7.12E-03	0.0230
TUBGCP6	-0.79	4.76E-03	0.0179
RNF111	-0.79	9.43E-03	0.0274
APBB3	-0.79	1.15E-02	0.0315
DAAM1	-0.79	1.28E-02	0.0337
OPTN	-0.79	9.11E-03	0.0269
FBXO11	-0.79	8.44E-03	0.0256
GUSBP1	-0.79	9.65E-03	0.0279
CNOT4	-0.79	5.57E-03	0.0197
TRBV18	-0.79	1.94E-02	0.0451
RN7SL49P	-0.79	2.05E-02	0.0470

CES4A	-0.79	6.37E-03	0.0215
ENST000003213410	-0.79	1.13E-02	0.0309
CEP70	-0.79	1.16E-02	0.0315
DUSP28	-0.79	1.13E-02	0.0309
LATS1	-0.79	5.83E-03	0.0203
SMAP1	-0.79	5.41E-03	0.0194
ZNF557	-0.79	6.25E-03	0.0212
DEDD2	-0.79	1.35E-02	0.0350
KMT2B	-0.79	1.02E-02	0.0288
AC084083.1	-0.79	1.50E-02	0.0376
TRAV12-1	-0.79	1.42E-02	0.0362
STAG3L3	-0.79	5.93E-03	0.0205
AC040162.1	-0.79	2.15E-02	0.0486
ENST000003300221	-0.79	9.63E-03	0.0279
ARID4A	-0.79	4.03E-03	0.0162
MYO18A	-0.79	1.00E-02	0.0286
WDR37	-0.79	3.39E-03	0.0147
IKZF2	-0.79	1.40E-02	0.0358
TMEM127	-0.80	6.01E-03	0.0207
CD247	-0.80	8.10E-03	0.0250
ZNF565	-0.80	2.01E-02	0.0462
KLHL36	-0.80	2.16E-02	0.0488
ETS1	-0.80	1.08E-02	0.0302
STAT2	-0.80	1.96E-02	0.0455
RALGAPA1	-0.80	1.75E-02	0.0419
CSRNP2	-0.80	8.22E-03	0.0252
ACAD11	-0.80	3.92E-03	0.0160
LINC01016	-0.80	1.25E-02	0.0331
USF3	-0.80	4.75E-03	0.0179
ABR	-0.80	6.22E-03	0.0211
PIK3CB	-0.80	1.17E-02	0.0317
AGO4	-0.80	9.66E-03	0.0279
CPLANE1	-0.80	3.38E-03	0.0146
ENST000003208760	-0.80	6.37E-03	0.0215
LINC02503	-0.80	7.85E-03	0.0244
PPP1R3D	-0.80	2.23E-02	0.0499
SRSF8	-0.80	4.80E-03	0.0180
PSKH1	-0.80	1.69E-02	0.0408
SPTAN1	-0.80	4.78E-03	0.0180
HABP4	-0.80	5.16E-03	0.0188
BCAS4	-0.80	6.08E-03	0.0208
TEPSIN	-0.80	2.18E-02	0.0492
MALAT1	-0.80	4.12E-03	0.0164
AC007000.2	-0.80	2.09E-02	0.0477
EXOC8	-0.81	3.67E-03	0.0153
TBRG1	-0.81	3.59E-03	0.0151
KLF3-AS1	-0.81	1.19E-02	0.0321
SLC9A8	-0.81	1.73E-02	0.0416
SGMS1	-0.81	3.48E-03	0.0149
ARGLU1	-0.81	7.37E-03	0.0235

GNPTG	-0.81	6.00E-03	0.0207
KIAA1257	-0.81	2.17E-02	0.0489
LAMP1	-0.81	6.98E-03	0.0228
ENST000003130501	-0.81	7.16E-03	0.0231
ZBTB39	-0.81	7.41E-03	0.0235
RIN3	-0.81	1.58E-02	0.0390
LMBRD1	-0.81	9.07E-03	0.0268
AC117529.1	-0.81	9.39E-03	0.0274
TMEM245	-0.81	7.78E-03	0.0243
DENND1C	-0.81	1.47E-02	0.0370
PPP1R12B	-0.81	3.56E-03	0.0150
ELF2	-0.81	4.46E-03	0.0173
ZNF597	-0.81	1.39E-02	0.0357
AC013652.1	-0.81	4.98E-03	0.0184
MPHOSPH8	-0.81	3.14E-03	0.0140
KLHL20	-0.81	3.33E-03	0.0145
CD3E	-0.82	5.42E-03	0.0194
GUSBP9	-0.82	1.09E-02	0.0303
ALDH2	-0.82	7.21E-03	0.0231
GLMP	-0.82	1.99E-02	0.0460
PNISR	-0.82	3.61E-03	0.0152
SMYD4	-0.82	8.87E-03	0.0264
SRPRA	-0.82	1.34E-02	0.0347
VPS36	-0.82	3.21E-03	0.0142
KIAA0319L	-0.82	5.26E-03	0.0190
MGAT4EP	-0.82	2.13E-02	0.0484
ACAA1	-0.82	1.69E-02	0.0409
KANSL1	-0.82	1.48E-02	0.0373
HECTD4	-0.82	6.87E-03	0.0226
AP000640.2	-0.82	1.83E-02	0.0433
FGF23	-0.82	8.26E-03	0.0253
FRS2	-0.82	6.91E-03	0.0226
DEF6	-0.82	6.62E-03	0.0220
STX7	-0.82	5.02E-03	0.0185
LINS1	-0.82	8.86E-03	0.0264
AC073052.1	-0.82	1.71E-02	0.0411
TMBIM4	-0.82	1.53E-02	0.0381
ZKSCAN2-DT	-0.82	6.86E-03	0.0225
ADAM10	-0.82	1.55E-02	0.0384
TSPAN6	-0.82	1.52E-02	0.0379
LINC01694	-0.82	2.11E-02	0.0480
TSHZ1	-0.82	1.66E-02	0.0403
RASA2	-0.82	5.17E-03	0.0188
PIKFYVE	-0.82	5.51E-03	0.0196
INPP4B	-0.82	3.17E-03	0.0141
AL162171.3	-0.82	1.54E-02	0.0382
AC004156.1	-0.83	1.90E-02	0.0445
CAMKMT	-0.83	6.59E-03	0.0220
BMS1P1	-0.83	4.40E-03	0.0171
AKAP9	-0.83	3.33E-03	0.0145

FAM45A	-0.83	3.38E-03	0.0146
TTC32	-0.83	1.90E-02	0.0445
RBSN	-0.83	1.09E-02	0.0304
BPTF	-0.83	3.33E-03	0.0145
SNORA12	-0.83	2.09E-02	0.0476
C2CD5	-0.83	2.82E-03	0.0133
KIAA0556	-0.83	2.03E-02	0.0467
LINC00342	-0.83	3.73E-03	0.0155
FCHO1	-0.83	6.76E-03	0.0223
AL356235.1	-0.83	1.18E-02	0.0319
ENST000002751590	-0.83	6.86E-03	0.0225
AC009113.1	-0.83	2.18E-02	0.0491
PIP5K1C	-0.83	1.35E-02	0.0349
SPTBN1	-0.83	9.04E-03	0.0267
TSC2	-0.83	1.67E-02	0.0406
KLF11	-0.83	2.00E-02	0.0461
CIZ1	-0.83	1.96E-02	0.0455
NIN	-0.84	1.17E-02	0.0318
ZNF433-AS1	-0.84	1.33E-02	0.0345
PRKAB1	-0.84	1.04E-02	0.0293
LINC01814	-0.84	9.04E-03	0.0267
AC087894.1	-0.84	9.28E-03	0.0272
LUC7L	-0.84	4.67E-03	0.0178
AL355997.1	-0.84	2.11E-02	0.0480
SEC31A	-0.84	9.97E-03	0.0285
ZMAT3	-0.84	7.96E-03	0.0247
NECAB3	-0.84	1.56E-02	0.0386
WDR45	-0.84	6.48E-03	0.0217
FBXO44	-0.84	6.39E-03	0.0215
AC138393.1	-0.84	8.88E-03	0.0264
ZNF14	-0.84	3.29E-03	0.0144
AL049646.1	-0.84	1.39E-02	0.0357
TMEM123	-0.84	5.44E-03	0.0194
UXS1	-0.84	1.98E-02	0.0458
ENST000002850211	-0.84	1.45E-02	0.0367
TRIM52-AS1	-0.84	9.23E-03	0.0271
ENST000003084880	-0.84	4.01E-03	0.0162
KLF13	-0.84	7.15E-03	0.0231
ACOT11	-0.84	8.15E-03	0.0251
SLC1A6	-0.84	2.03E-02	0.0467
ARMC7	-0.84	9.33E-03	0.0273
AAK1	-0.84	4.28E-03	0.0168
NFE2L2	-0.84	1.08E-02	0.0302
SLC25A45	-0.84	2.02E-02	0.0464
RUBCN	-0.84	3.71E-03	0.0155
RBM33	-0.84	3.27E-03	0.0143
PHKB	-0.84	3.64E-03	0.0153
SLAIN1	-0.84	9.22E-03	0.0271
EPG5	-0.85	3.79E-03	0.0156
ZFYVE26	-0.85	4.98E-03	0.0184

FRG1JP	-0.85	1.61E-02	0.0395
RN7SL732P	-0.85	2.19E-02	0.0493
AC060234.2	-0.85	1.42E-02	0.0362
SDR39U1	-0.85	5.69E-03	0.0200
ZNF609	-0.85	2.75E-03	0.0131
HAPLN3	-0.85	7.20E-03	0.0231
IRAK4	-0.85	2.85E-03	0.0134
AC093752.1	-0.85	1.20E-02	0.0323
TRIM34	-0.85	1.22E-02	0.0326
AP005131.1	-0.85	1.65E-02	0.0402
CORO7	-0.85	6.95E-03	0.0227
FNIP1	-0.85	6.85E-03	0.0225
VPS53	-0.85	3.31E-03	0.0144
WBP1L	-0.85	8.27E-03	0.0253
ZNF606	-0.85	5.14E-03	0.0188
ZBTB8B	-0.85	8.74E-03	0.0262
AC093297.2	-0.85	6.78E-03	0.0223
TMEM161B	-0.85	6.34E-03	0.0214
RNASEH2B	-0.85	1.45E-02	0.0367
PDPR	-0.85	2.20E-02	0.0495
MAP3K12	-0.86	5.77E-03	0.0202
NSUN5P2	-0.86	1.63E-02	0.0399
AP000919.1	-0.86	7.68E-03	0.0241
MACF1	-0.86	3.64E-03	0.0153
EPS15L1	-0.86	1.21E-02	0.0324
AC137932.3	-0.86	1.16E-02	0.0316
VPS26B	-0.86	4.62E-03	0.0176
ENST000003582320	-0.86	2.72E-03	0.0131
YY1AP1	-0.86	2.12E-02	0.0481
MXD1	-0.86	4.57E-03	0.0176
AC020978.7	-0.86	8.58E-03	0.0259
RNF149	-0.86	2.48E-03	0.0124
ENST000002621600	-0.86	1.11E-02	0.0307
ARHGAP27	-0.86	4.29E-03	0.0169
SPPL2B	-0.86	1.60E-02	0.0394
BTBD9	-0.86	2.80E-03	0.0133
CLUAP1	-0.86	7.28E-03	0.0233
ZNF780B	-0.86	2.98E-03	0.0137
NCOA6	-0.86	5.40E-03	0.0194
NDEL1	-0.86	1.26E-02	0.0333
PBLD	-0.86	1.12E-02	0.0308
CBLB	-0.86	4.90E-03	0.0182
SMARCA2	-0.87	1.06E-02	0.0297
ZNF91	-0.87	1.24E-02	0.0330
C11orf71	-0.87	1.18E-02	0.0318
NBPF11	-0.87	1.22E-02	0.0326
XPA	-0.87	3.09E-03	0.0140
AC092910.3	-0.87	1.39E-02	0.0357
AC106820.4	-0.87	1.25E-02	0.0331
CYB561D2	-0.87	7.78E-03	0.0243

AFF1	-0.87	1.85E-02	0.0437
ARHGEF6	-0.87	4.44E-03	0.0172
ZKSCAN8	-0.87	2.61E-03	0.0127
TRDMT1	-0.87	6.73E-03	0.0223
AC138409.2	-0.87	1.26E-02	0.0333
OR5AS1	-0.87	3.22E-03	0.0142
AC103957.1	-0.87	9.18E-03	0.0270
ZNF441	-0.87	2.68E-03	0.0129
MYORG	-0.87	6.69E-03	0.0222
AC018554.1	-0.87	1.85E-02	0.0436
SLC7A6	-0.87	2.12E-02	0.0481
OSBPL2	-0.87	3.05E-03	0.0138
KIF6	-0.87	1.60E-02	0.0393
YAF2	-0.87	3.86E-03	0.0158
ZNF230	-0.88	6.43E-03	0.0216
TRAF5	-0.88	3.46E-03	0.0148
SCARNA9	-0.88	9.64E-03	0.0279
WASHC2A	-0.88	3.07E-03	0.0139
AC025279.1	-0.88	1.95E-02	0.0453
SNN	-0.88	3.65E-03	0.0153
AFF4	-0.88	5.53E-03	0.0196
CDH26	-0.88	1.15E-02	0.0315
PAXIP1-AS1	-0.88	1.02E-02	0.0290
MED15	-0.88	1.66E-02	0.0403
COG1	-0.88	7.98E-03	0.0247
AC074141.1	-0.88	1.45E-02	0.0368
ENST000003162863	-0.88	1.16E-02	0.0317
DGKZ	-0.88	7.66E-03	0.0241
AF131215.6	-0.88	7.78E-03	0.0243
MBTD1	-0.88	2.84E-03	0.0133
TMC6	-0.88	3.83E-03	0.0158
MEI1	-0.88	1.03E-02	0.0292
AL353625.1	-0.88	1.42E-02	0.0362
CDKL1	-0.88	2.14E-02	0.0485
KLHL21	-0.88	4.19E-03	0.0166
RUFY2	-0.88	2.79E-03	0.0133
SLC38A7	-0.88	8.62E-03	0.0259
WTIP	-0.88	1.40E-02	0.0357
ZNF845	-0.88	3.44E-03	0.0148
METTL25	-0.88	1.60E-02	0.0393
OR5BA1P	-0.88	1.02E-02	0.0290
OR10G3	-0.88	1.15E-02	0.0315
PTEN	-0.88	2.45E-03	0.0123
AC118459.2	-0.88	1.54E-02	0.0382
SLC22A5	-0.88	1.05E-02	0.0294
IFT80	-0.88	4.50E-03	0.0174
AC013394.1	-0.88	4.09E-03	0.0164
TNFAIP8L2	-0.88	3.94E-03	0.0160
HIF3A	-0.89	1.66E-02	0.0404
RNF139-AS1	-0.89	1.80E-02	0.0428

AC005005.4	-0.89	5.30E-03	0.0191
GNAQ	-0.89	2.66E-03	0.0129
ELF1	-0.89	7.03E-03	0.0229
LINC02018	-0.89	1.26E-02	0.0334
AL360270.2	-0.89	6.78E-03	0.0223
TTC28	-0.89	1.81E-02	0.0429
RF00017.187	-0.89	2.21E-02	0.0496
RNF146	-0.89	2.79E-03	0.0133
KCNQ1OT1	-0.89	2.09E-03	0.0112
C8orf44	-0.89	1.17E-02	0.0318
AC010680.2	-0.89	1.15E-02	0.0314
AP5M1	-0.89	7.82E-03	0.0244
CDC37L1	-0.89	2.32E-03	0.0120
BMS1P2	-0.89	5.99E-03	0.0207
CCDC88C	-0.89	1.60E-02	0.0393
LNPEP	-0.89	4.92E-03	0.0183
FCRL5	-0.89	7.06E-03	0.0229
MAU2	-0.89	1.56E-02	0.0387
GPR21	-0.89	2.12E-02	0.0482
SMG1P7	-0.89	5.12E-03	0.0187
CMC1	-0.89	7.00E-03	0.0228
LINC00346	-0.90	8.00E-03	0.0247
AC105285.1	-0.90	1.64E-02	0.0400
CD55	-0.90	2.73E-03	0.0131
PAIP2B	-0.90	6.09E-03	0.0209
TRBV15	-0.90	1.05E-02	0.0296
NUPR1	-0.90	1.34E-02	0.0347
CD3G	-0.90	2.55E-03	0.0126
GUSBP2	-0.90	7.79E-03	0.0243
ZNF776	-0.90	3.32E-03	0.0145
MIR193BHG	-0.90	2.21E-02	0.0496
SETDB2	-0.90	8.25E-03	0.0253
AC006504.1	-0.90	7.09E-03	0.0230
AC106820.2	-0.90	1.29E-02	0.0339
GPR135	-0.90	8.72E-03	0.0262
AP001469.3	-0.90	8.73E-03	0.0262
ZNF816	-0.90	1.32E-02	0.0344
BBS9	-0.90	1.78E-02	0.0425
TEX9	-0.90	1.21E-02	0.0326
ENST000003064810	-0.90	1.29E-02	0.0339
AC016907.2	-0.90	1.28E-02	0.0337
RBM26	-0.90	1.22E-02	0.0327
HEATR5B	-0.90	6.32E-03	0.0214
BLMH	-0.91	6.91E-03	0.0226
GPD1L	-0.91	2.49E-03	0.0124
TPD52L1	-0.91	1.68E-02	0.0407
MAP4K4	-0.91	1.98E-03	0.0108
ZNF416	-0.91	9.33E-03	0.0273
ZAP70	-0.91	1.02E-02	0.0290
AL355075.4	-0.91	4.16E-03	0.0165

SP140L	-0.91	1.49E-02	0.0374
PIK3CA	-0.91	1.98E-02	0.0458
AC024568.1	-0.91	1.27E-02	0.0336
C5orf22	-0.91	7.19E-03	0.0231
FAM117B	-0.91	1.70E-02	0.0409
ENST000003196703	-0.91	1.93E-02	0.0450
BRD1	-0.91	1.02E-02	0.0290
JMJD1C	-0.91	2.31E-03	0.0119
ELP4	-0.91	2.62E-03	0.0128
ERBIN	-0.91	2.85E-03	0.0134
SYDE2	-0.91	5.22E-03	0.0190
NAAA	-0.91	7.88E-03	0.0245
SPACA6P-AS	-0.91	1.30E-02	0.0339
RC3H2	-0.91	1.95E-03	0.0108
ZFYVE16	-0.91	3.08E-03	0.0139
ZNF701	-0.91	1.01E-02	0.0287
ZNF573	-0.91	5.43E-03	0.0194
ZNF284	-0.91	1.13E-02	0.0310
ZNF615	-0.91	2.87E-03	0.0134
ZNF337	-0.91	2.90E-03	0.0135
AC020978.6	-0.91	8.32E-03	0.0254
EP400P1	-0.91	4.70E-03	0.0178
SARM1	-0.91	4.29E-03	0.0169
LRIG2	-0.91	2.64E-03	0.0128
ZNF83	-0.91	1.80E-02	0.0428
LZTFL1	-0.91	9.11E-03	0.0269
MRNIP	-0.91	4.96E-03	0.0184
ZNF251	-0.91	1.29E-02	0.0338
DAZAP2	-0.92	5.56E-03	0.0197
UBOX5	-0.92	6.70E-03	0.0222
KAT6A	-0.92	2.10E-03	0.0113
ENST000002489010	-0.92	2.12E-03	0.0113
STK33	-0.92	8.28E-03	0.0253
ENST000003405351	-0.92	8.81E-03	0.0263
CDC42SE2	-0.92	1.96E-03	0.0108
GYG1	-0.92	1.07E-02	0.0300
CBL	-0.92	3.42E-03	0.0147
TBCEL	-0.92	6.55E-03	0.0219
RPGR	-0.92	1.11E-02	0.0306
ZNF658B	-0.92	1.10E-02	0.0305
DNAJC27	-0.92	2.14E-02	0.0485
CDKL5	-0.92	1.51E-02	0.0377
CARD6	-0.92	9.90E-03	0.0284
SPPL3	-0.92	7.91E-03	0.0246
KMT2C	-0.92	5.40E-03	0.0194
AC007326.5	-0.92	9.94E-03	0.0285
SPNS1	-0.92	7.35E-03	0.0234
ENST000003033910	-0.92	4.43E-03	0.0172
ARHGEF26-AS1	-0.92	1.68E-02	0.0407
SLC39A10	-0.92	3.12E-03	0.0140

OR6Y1	-0.92	6.70E-03	0.0222
TTYH1	-0.92	1.72E-02	0.0414
SCIMP	-0.92	1.72E-02	0.0414
MSL2	-0.92	2.73E-03	0.0131
ENST000002740300	-0.93	2.01E-02	0.0462
LNX2	-0.93	7.47E-03	0.0237
CACNA1A	-0.93	8.36E-03	0.0255
PDCD4	-0.93	1.32E-02	0.0344
Z97989.1	-0.93	1.26E-02	0.0333
ANKRD13D	-0.93	3.78E-03	0.0156
ARHGEF9	-0.93	1.37E-02	0.0353
SPATA6L	-0.93	7.99E-03	0.0247
NPIPB4	-0.93	1.84E-02	0.0435
AC068620.3	-0.93	1.95E-02	0.0454
ENST000002584840	-0.93	2.59E-03	0.0127
ZNF611	-0.93	6.13E-03	0.0210
PPARA	-0.93	2.54E-03	0.0125
ERCC6	-0.93	5.91E-03	0.0205
ENST000003443370	-0.93	1.93E-03	0.0107
RPAP2	-0.93	1.91E-03	0.0106
FAM199X	-0.93	3.14E-03	0.0140
LINC01695	-0.93	5.86E-03	0.0204
ENST000002992400	-0.93	9.34E-03	0.0273
PCDHB1	-0.93	1.27E-02	0.0334
PCIF1	-0.93	1.31E-02	0.0342
ZNF580	-0.93	8.04E-03	0.0248
RAB11FIP2	-0.93	2.81E-03	0.0133
RN7SL760P	-0.93	2.11E-02	0.0480
AC103740.1	-0.93	1.48E-02	0.0372
PAN3	-0.93	2.15E-03	0.0114
TJP1	-0.93	2.15E-02	0.0486
ZMYM4	-0.93	1.45E-02	0.0367
AC007342.4	-0.93	2.01E-02	0.0464
ZXDA	-0.93	3.98E-03	0.0161
PLEKHM3	-0.93	2.52E-03	0.0125
DENND4B	-0.94	5.82E-03	0.0203
WASL	-0.94	1.33E-02	0.0345
AC092326.1	-0.94	1.30E-02	0.0340
AC246789.2	-0.94	1.30E-02	0.0340
RGR	-0.94	1.67E-02	0.0405
SPATA17	-0.94	1.04E-02	0.0293
ZNF461	-0.94	2.98E-03	0.0137
EFHC1	-0.94	4.13E-03	0.0165
FBXO25	-0.94	1.20E-02	0.0323
ZNF33A	-0.94	4.05E-03	0.0162
S100PBP	-0.94	2.36E-03	0.0121
TRIM68	-0.94	2.18E-02	0.0491
TAS2R64P	-0.94	5.23E-03	0.0190
OR8A1	-0.94	8.74E-03	0.0262
TIA1	-0.94	2.33E-03	0.0120

LINC00680	-0.94	5.24E-03	0.0190
TBXAS1	-0.94	1.15E-02	0.0314
ANKRD36BP2	-0.94	3.84E-03	0.0158
HIPK1	-0.94	3.08E-03	0.0139
OR5M2P	-0.94	1.53E-02	0.0380
AF131215.5	-0.94	1.59E-02	0.0391
AC011939.1	-0.94	1.97E-02	0.0457
HERC2P8	-0.94	2.06E-02	0.0471
SLC26A2	-0.94	4.26E-03	0.0168
TESMIN	-0.94	1.86E-02	0.0438
LINC00174	-0.94	8.36E-03	0.0255
KDM7A-DT	-0.94	1.76E-02	0.0419
ZNF266	-0.94	2.48E-03	0.0124
GON4L	-0.94	2.81E-03	0.0133
CAPN10-DT	-0.94	1.07E-02	0.0300
PPP3CC	-0.94	4.20E-03	0.0166
LDLRAD4	-0.94	1.20E-02	0.0322
MPRIP	-0.94	3.20E-03	0.0142
ENST000003710680	-0.94	9.82E-03	0.0282
FAM120B	-0.94	3.25E-03	0.0143
CD53	-0.94	1.93E-03	0.0107
LMF2	-0.95	7.19E-03	0.0231
NRP1	-0.95	1.93E-02	0.0449
ENST000002856790	-0.95	2.97E-03	0.0136
CSMD3	-0.95	6.08E-03	0.0208
EMP2	-0.95	9.52E-03	0.0276
AL713866.1	-0.95	1.63E-02	0.0398
TYW1B	-0.95	8.58E-03	0.0259
HARBI1	-0.95	1.28E-02	0.0337
ENST000003170252	-0.95	4.76E-03	0.0179
ZIM2-AS1	-0.95	8.91E-03	0.0265
STAG3L1	-0.95	1.19E-02	0.0321
LINC02471	-0.95	1.84E-02	0.0435
GADL1	-0.95	7.20E-03	0.0231
ABLIM1	-0.95	4.19E-03	0.0166
KLRD1	-0.95	3.38E-03	0.0146
ZNF655	-0.95	1.71E-03	0.0101
BBOF1	-0.95	7.53E-03	0.0238
NBPF10	-0.95	9.59E-03	0.0278
AC256309.2	-0.95	1.22E-02	0.0326
AC099550.1	-0.95	1.27E-02	0.0336
AC010327.5	-0.95	1.83E-02	0.0434
GCC2	-0.95	5.66E-03	0.0199
HIVEP2	-0.95	7.45E-03	0.0236
KYNU	-0.95	2.38E-03	0.0121
ASTE1	-0.95	8.56E-03	0.0258
LYRM9	-0.95	8.45E-03	0.0256
CNTNAP3B	-0.95	2.19E-02	0.0493
ENST000003559770	-0.95	6.94E-03	0.0227
AC132008.2	-0.96	7.17E-03	0.0231

MOK	-0.96	6.45E-03	0.0216
SH3BGRL	-0.96	1.73E-02	0.0415
ENST000002665460	-0.96	1.72E-02	0.0413
PAXX	-0.96	5.55E-03	0.0197
ABCC10	-0.96	2.73E-03	0.0131
AL603840.1	-0.96	6.81E-03	0.0224
PRKAB2	-0.96	3.12E-03	0.0140
CDKN1B	-0.96	7.42E-03	0.0236
ZNF585B	-0.96	2.93E-03	0.0135
CD37	-0.96	7.20E-03	0.0231
ATP2A3	-0.96	4.25E-03	0.0167
SEMA4F	-0.96	6.48E-03	0.0217
ESYT2	-0.96	1.20E-02	0.0324
ENST000002633980	-0.96	1.28E-02	0.0338
HELLPAR	-0.96	1.84E-03	0.0105
AL513523.1	-0.96	9.51E-03	0.0276
AL513523.5	-0.96	9.51E-03	0.0276
TECR	-0.96	5.11E-03	0.0187
LACTB2-AS1	-0.96	1.06E-02	0.0297
IRF7	-0.96	1.78E-02	0.0423
LINC01578	-0.96	1.97E-03	0.0108
AGAP5	-0.96	6.12E-03	0.0209
ZNF394	-0.96	1.58E-03	0.0097
MSS51	-0.96	1.49E-02	0.0375
AL117378.1	-0.96	6.23E-03	0.0211
SF1	-0.96	1.19E-02	0.0322
AC123768.4	-0.96	1.01E-02	0.0288
AC016708.1	-0.96	3.73E-03	0.0155
TRAK2	-0.97	1.05E-02	0.0294
LYSMD4	-0.97	4.13E-03	0.0165
ENST000002908550	-0.97	1.32E-02	0.0344
ZNF548	-0.97	2.88E-03	0.0134
AC092821.3	-0.97	1.52E-02	0.0379
KMT2D	-0.97	1.83E-02	0.0433
TTLL7-IT1	-0.97	1.98E-02	0.0459
RBM5	-0.97	1.63E-03	0.0098
HERC1	-0.97	2.08E-03	0.0112
TMEM116	-0.97	8.18E-03	0.0251
OR7A17	-0.97	1.56E-02	0.0386
AC002551.1	-0.97	2.17E-02	0.0490
AL033397.1	-0.97	8.08E-03	0.0249
AL590764.1	-0.97	1.25E-02	0.0332
MRTFA	-0.97	1.96E-02	0.0454
EP300	-0.97	2.44E-03	0.0123
ZNF592	-0.97	4.71E-03	0.0178
TRAJ35	-0.97	1.51E-02	0.0378
ZNF260	-0.97	1.78E-03	0.0103
TRAJ5	-0.97	1.11E-02	0.0306
LMCD1-AS1	-0.97	1.58E-02	0.0390
CLYBL	-0.97	2.20E-02	0.0495

CNTRL	-0.97	3.20E-03	0.0142
ACER2	-0.97	8.96E-03	0.0266
Z95114.2	-0.97	1.20E-02	0.0324
ZNF773	-0.97	3.25E-03	0.0143
LENG1	-0.97	6.94E-03	0.0227
AP000866.6	-0.98	9.83E-03	0.0283
RAB33B	-0.98	7.97E-03	0.0247
E2F3-IT1	-0.98	1.40E-02	0.0359
C17orf77	-0.98	7.82E-03	0.0244
VILL	-0.98	2.10E-02	0.0478
HYDIN	-0.98	2.10E-02	0.0478
SSH2	-0.98	1.56E-03	0.0096
CFAP69	-0.98	1.15E-02	0.0314
ZNF596	-0.98	8.11E-03	0.0250
SMCR8	-0.98	6.02E-03	0.0207
TRAJ33	-0.98	5.25E-03	0.0190
CNPY4	-0.98	1.62E-02	0.0397
ZNF674	-0.98	3.85E-03	0.0158
ITFG2	-0.98	2.09E-03	0.0112
PNRC1	-0.98	1.25E-02	0.0331
LINC01359	-0.98	1.58E-02	0.0389
KLHL28	-0.98	5.56E-03	0.0197
SLITRK2	-0.98	1.27E-02	0.0335
ENST00000562313	-0.98	1.49E-02	0.0374
ORMDL1	-0.98	2.45E-03	0.0123
AC004494.1	-0.98	1.43E-02	0.0363
CLIP1	-0.98	6.21E-03	0.0211
PCED1B	-0.98	1.22E-02	0.0326
MBLAC2	-0.98	3.54E-03	0.0150
LINC02057	-0.98	2.14E-02	0.0484
GABPB1-IT1	-0.98	5.13E-03	0.0188
ACTR3C	-0.98	5.35E-03	0.0192
EFCAB5	-0.98	5.27E-03	0.0190
JAK1	-0.98	2.54E-03	0.0126
IQCF3	-0.99	6.90E-03	0.0226
BAZ2B	-0.99	1.62E-02	0.0396
ENST000002444260	-0.99	5.84E-03	0.0203
PCCA	-0.99	1.89E-03	0.0106
ENST000002621890	-0.99	1.52E-03	0.0095
AL080250.1	-0.99	7.49E-03	0.0237
TBCK	-0.99	2.48E-03	0.0124
AC134349.1	-0.99	1.22E-02	0.0327
AC134349.4	-0.99	1.22E-02	0.0327
AC245078.2	-0.99	1.22E-02	0.0327
ENST000002889851	-0.99	8.06E-03	0.0249
MPC1	-0.99	7.41E-03	0.0235
TRPM8	-0.99	1.43E-02	0.0363
LRRK1	-0.99	1.86E-02	0.0439
FLI1	-0.99	2.59E-03	0.0127
AGO3	-0.99	1.47E-03	0.0093

VASH1-AS1	-0.99	7.93E-03	0.0246
RF00017.127	-0.99	2.17E-02	0.0489
TIGD1	-0.99	1.36E-03	0.0089
LTO1	-0.99	1.96E-03	0.0108
LFNG	-0.99	2.12E-02	0.0481
STX5	-0.99	5.11E-03	0.0187
EPC2	-0.99	5.65E-03	0.0199
AC114980.1	-0.99	1.17E-02	0.0318
AC009061.2	-0.99	1.14E-02	0.0311
C5orf17	-0.99	1.36E-02	0.0350
AC087286.4	-0.99	7.03E-03	0.0229
PTAR1	-0.99	1.52E-03	0.0095
AL049874.3	-0.99	2.21E-02	0.0496
ENST000002420670	-0.99	7.94E-03	0.0247
TRAJ40	-0.99	9.48E-03	0.0275
GABRE	-0.99	1.52E-02	0.0379
HERPUD2	-0.99	4.84E-03	0.0181
ZC3H12B	-0.99	7.54E-03	0.0238
AC009948.2	-0.99	6.90E-03	0.0226
CCDC169	-0.99	5.16E-03	0.0188
GPLD1	-0.99	1.04E-02	0.0293
CDK11A	-0.99	1.87E-03	0.0106
TMX4	-1.00	3.14E-03	0.0140
PRDM5	-1.00	4.74E-03	0.0179
ENST000003130401	-1.00	1.37E-02	0.0353
AC061975.1	-1.00	1.89E-02	0.0443
PARP11	-1.00	3.89E-03	0.0159
TOB1	-1.00	1.80E-03	0.0103
CARD8	-1.00	1.48E-03	0.0093
SGSM3	-1.00	3.60E-03	0.0152
ENST000003269250	-1.00	9.42E-03	0.0274
RFX3	-1.00	1.62E-03	0.0098
ENST000003668550	-1.00	3.58E-03	0.0151
RWDD2A	-1.00	1.61E-02	0.0395
FBXW4	-1.00	2.58E-03	0.0127
MBTPS1	-1.00	5.40E-03	0.0194
AC011726.2	-1.00	8.87E-03	0.0264
ENST000003140280	-1.00	1.88E-02	0.0441
AC027288.3	-1.00	4.21E-03	0.0167
GOLGA6L5P	-1.00	2.08E-02	0.0475
DDR1-DT	-1.00	3.45E-03	0.0148
PDXDC2P-NPIPB14P	-1.00	1.40E-02	0.0359
ASH1L	-1.00	1.90E-03	0.0106
CALN1	-1.00	1.30E-02	0.0339
RRAGB	-1.00	4.39E-03	0.0171
ENST000003530470	-1.01	5.59E-03	0.0198
ZNF354B	-1.01	7.04E-03	0.0229
SFTPD-AS1	-1.01	1.10E-02	0.0304
ZNF264	-1.01	3.98E-03	0.0161
TRAPPC6A	-1.01	7.98E-03	0.0247

GALR1	-1.01	8.15E-03	0.0251
AC092474.1	-1.01	9.26E-03	0.0271
ENST000003408550	-1.01	5.13E-03	0.0188
ENST000002496360	-1.01	1.41E-03	0.0091
RENBP	-1.01	1.22E-02	0.0326
ADCY2	-1.01	1.13E-02	0.0311
UBAP1L	-1.01	5.48E-03	0.0195
AP002490.1	-1.01	1.89E-02	0.0443
ENST000002949522	-1.01	1.83E-02	0.0434
ADCY7	-1.01	1.77E-03	0.0102
ENST000002880710	-1.01	1.55E-02	0.0385
AL121652.1	-1.01	1.54E-02	0.0383
ITGA9-AS1	-1.01	2.06E-02	0.0472
DLGAP1-AS2	-1.01	4.01E-03	0.0162
RRN3P3	-1.01	4.91E-03	0.0183
ZNF483	-1.01	8.29E-03	0.0253
ARRDC3-AS1	-1.01	6.76E-03	0.0223
SAG	-1.01	8.37E-03	0.0255
NAGPA-AS1	-1.01	1.20E-02	0.0323
ENST000003361281	-1.01	1.30E-02	0.0340
PRRC2B	-1.01	1.06E-02	0.0297
RN7SL711P	-1.01	1.06E-02	0.0297
SRP14-AS1	-1.01	8.49E-03	0.0257
NSD3	-1.02	1.82E-03	0.0104
MORC2	-1.02	1.89E-03	0.0106
AL139385.1	-1.02	5.63E-03	0.0198
ENST000002704600	-1.02	6.97E-03	0.0228
IPO5P1	-1.02	2.54E-03	0.0125
AL683813.1	-1.02	4.01E-03	0.0162
FLCN	-1.02	4.64E-03	0.0177
ACSM2B	-1.02	4.59E-03	0.0176
AP000662.1	-1.02	6.85E-03	0.0225
RIPK1	-1.02	1.31E-03	0.0087
LINC01206	-1.02	1.58E-02	0.0390
DGKD	-1.02	2.35E-03	0.0121
ZNF619	-1.02	4.56E-03	0.0175
AC005041.1	-1.02	4.93E-03	0.0183
AC023389.2	-1.02	1.96E-02	0.0455
MROH1	-1.02	4.90E-03	0.0182
PLXNA3	-1.02	8.89E-03	0.0265
AC087284.1	-1.02	2.14E-02	0.0484
RAPGEF6	-1.02	3.54E-03	0.0150
ASTN2	-1.02	3.51E-03	0.0149
TRAJ31	-1.02	5.86E-03	0.0204
AC092902.4	-1.02	4.82E-03	0.0181
AC010601.1	-1.02	1.02E-02	0.0290
SNX16	-1.02	1.47E-02	0.0370
AL159169.2	-1.03	1.59E-02	0.0391
GPCPD1	-1.03	2.50E-03	0.0124
AHNAK	-1.03	4.23E-03	0.0167

OR1I1	-1.03	3.55E-03	0.0150
ENST000002630950	-1.03	3.85E-03	0.0158
CROT	-1.03	1.94E-03	0.0107
ENST000003180102	-1.03	3.65E-03	0.0153
RETREG3	-1.03	2.06E-03	0.0111
LRRC37A2	-1.03	3.61E-03	0.0152
CRY2	-1.03	2.64E-03	0.0128
LINC00652	-1.03	1.33E-02	0.0345
AC245060.6	-1.03	5.12E-03	0.0187
CHP2	-1.03	5.20E-03	0.0189
NPIPB13	-1.03	6.27E-03	0.0212
AP000704.1	-1.03	1.96E-02	0.0455
LRRC69	-1.03	4.05E-03	0.0162
AC015871.3	-1.03	4.31E-03	0.0169
PRDM2	-1.03	4.36E-03	0.0170
RPS27	-1.03	2.28E-03	0.0118
ULK1	-1.03	8.22E-03	0.0252
P4HTM	-1.03	6.70E-03	0.0222
IQCH-AS1	-1.03	3.13E-03	0.0140
AC005899.6	-1.03	1.35E-02	0.0349
ENST000003164913	-1.03	7.90E-03	0.0246
YLPM1	-1.03	1.38E-03	0.0090
LINC01232	-1.03	3.70E-03	0.0154
STYX	-1.04	1.36E-03	0.0089
ZNF157	-1.04	1.89E-02	0.0443
AL807752.1	-1.04	1.92E-02	0.0449
ENST000003357273	-1.04	9.16E-03	0.0269
CFL1P1	-1.04	1.17E-02	0.0317
LMF1	-1.04	1.52E-02	0.0379
ENST000002994270	-1.04	1.04E-02	0.0293
ENST000003006191	-1.04	2.08E-03	0.0112
ENST000002194780	-1.04	3.11E-03	0.0140
KCNQ1	-1.04	7.23E-03	0.0232
PPP3CA	-1.04	1.78E-03	0.0103
ZNRF2	-1.04	5.48E-03	0.0195
CAT	-1.04	8.56E-03	0.0258
MIA2	-1.04	1.39E-03	0.0090
LINC00847	-1.04	3.67E-03	0.0153
IGSF9B	-1.04	3.17E-03	0.0141
KIAA1109	-1.04	1.11E-03	0.0081
CYP3A5	-1.04	7.07E-03	0.0229
NDRG1	-1.04	4.98E-03	0.0184
AC008969.1	-1.04	8.47E-03	0.0257
XKR8	-1.04	1.17E-02	0.0318
RFFL	-1.04	2.44E-03	0.0123
SYNJ1	-1.04	7.12E-03	0.0230
TMEM212	-1.04	2.89E-03	0.0135
TRAJ10	-1.04	6.08E-03	0.0208
ENST000003071380	-1.04	2.35E-03	0.0121
USP20	-1.04	4.03E-03	0.0162

AC073569.3	-1.04	5.52E-03	0.0196
HDAC10	-1.04	2.10E-02	0.0478
AC092431.2	-1.04	1.80E-02	0.0428
TRBV27	-1.05	1.92E-02	0.0448
SCAMP2	-1.05	8.88E-03	0.0264
CARMN	-1.05	2.06E-02	0.0472
OR10H1	-1.05	1.12E-02	0.0309
AC139887.2	-1.05	6.50E-03	0.0218
CNN2	-1.05	1.25E-02	0.0332
DGKQ	-1.05	1.56E-02	0.0387
LINC01238	-1.05	3.79E-03	0.0157
NOTCH2NLA	-1.05	3.12E-03	0.0140
AC006145.1	-1.05	3.73E-03	0.0155
HERC2P2	-1.05	1.40E-03	0.0090
PRH1	-1.05	1.51E-02	0.0378
AC007038.2	-1.05	5.44E-03	0.0194
MAPK10	-1.05	1.63E-03	0.0098
GHET1	-1.05	5.06E-03	0.0186
OLMALINC	-1.05	2.77E-03	0.0132
ENST000003172211	-1.05	3.30E-03	0.0144
ENST000002628110	-1.05	3.17E-03	0.0141
ENST000002662541	-1.05	2.19E-02	0.0493
MARK2	-1.05	4.07E-03	0.0163
ENST000003386371	-1.05	4.72E-03	0.0178
AC008764.8	-1.05	1.10E-02	0.0304
TRIML2	-1.05	1.58E-02	0.0391
TBC1D10C	-1.05	7.58E-03	0.0239
CLSTN1	-1.05	2.12E-03	0.0113
IL16	-1.05	1.06E-02	0.0297
FAM120C	-1.05	9.94E-03	0.0285
LINC02073	-1.05	5.17E-03	0.0188
RNF216P1	-1.05	7.73E-03	0.0242
AC093591.2	-1.05	1.90E-02	0.0444
PQLC3	-1.05	2.71E-03	0.0130
ARHGAP1	-1.05	1.54E-02	0.0383
ZNF510	-1.05	1.34E-03	0.0088
ZNF18	-1.06	2.23E-03	0.0117
AC011933.2	-1.06	4.30E-03	0.0169
ZDHHC2	-1.06	1.59E-02	0.0392
AL513314.2	-1.06	2.12E-02	0.0481
SMARCE1P1	-1.06	5.37E-03	0.0193
EIF1B-AS1	-1.06	1.20E-02	0.0322
SGSM2	-1.06	5.61E-03	0.0198
MIPOL1	-1.06	1.93E-03	0.0107
ZNF667-AS1	-1.06	3.89E-03	0.0159
NDUFA6-DT	-1.06	4.08E-03	0.0163
TUT1	-1.06	8.82E-03	0.0263
AP000708.1	-1.06	2.15E-02	0.0486
ZNF236	-1.06	6.23E-03	0.0212
HIP1R	-1.06	7.10E-03	0.0230

FBLN1	-1.06	1.20E-02	0.0322
ODF2L	-1.06	1.10E-03	0.0080
PHYKPL	-1.06	1.11E-03	0.0080
OTUD1	-1.06	5.30E-03	0.0191
AC008079.1	-1.06	3.88E-03	0.0159
TSC1	-1.06	3.64E-03	0.0153
SNRPN	-1.06	1.52E-03	0.0095
LINC02246	-1.06	1.08E-02	0.0301
ZBTB49	-1.06	1.20E-02	0.0322
LINC01684	-1.06	1.58E-03	0.0097
FAM78A	-1.06	1.57E-03	0.0096
AC135050.1	-1.06	1.22E-02	0.0327
ZNF10	-1.06	8.46E-03	0.0257
TRAV1-1	-1.06	2.21E-02	0.0496
CD302	-1.06	1.04E-02	0.0294
LIPT1	-1.06	4.83E-03	0.0181
37226	-1.06	4.65E-03	0.0177
RAD21-AS1	-1.06	1.80E-02	0.0428
AP003175.1	-1.06	1.88E-02	0.0441
ZZEF1	-1.06	2.26E-03	0.0118
ZNF782	-1.06	3.44E-03	0.0148
AC124016.2	-1.07	1.01E-02	0.0288
PDE4DIP	-1.07	3.95E-03	0.0160
SCRG1	-1.07	8.53E-03	0.0258
ARIH2OS	-1.07	1.25E-02	0.0332
IZUMO1	-1.07	1.67E-02	0.0405
SARAF	-1.07	8.56E-03	0.0258
NPIPB5	-1.07	1.08E-02	0.0301
AL139353.1	-1.07	1.64E-02	0.0400
NUP50-DT	-1.07	2.16E-02	0.0488
LINC00663	-1.07	7.79E-03	0.0243
NOP53	-1.07	2.55E-03	0.0126
CLEC19A	-1.07	1.17E-02	0.0318
HMGN1P8	-1.07	9.05E-03	0.0267
MFSD8	-1.07	1.40E-03	0.0091
AP000542.2	-1.07	4.82E-03	0.0181
TRAJ36	-1.07	2.03E-02	0.0467
GGT5	-1.07	1.65E-02	0.0402
UBE2Q2	-1.07	1.99E-03	0.0109
PATL2	-1.07	1.97E-02	0.0456
AC139494.1	-1.07	1.59E-02	0.0393
NFRKB	-1.07	1.42E-03	0.0091
GALC	-1.07	2.38E-03	0.0121
BTBD8	-1.07	5.29E-03	0.0191
VCPIP1	-1.07	3.52E-03	0.0150
RF00017.35	-1.07	1.75E-02	0.0419
ENST000003412490	-1.07	1.01E-02	0.0288
AC093323.1	-1.07	2.24E-03	0.0117
GNRHR2	-1.07	1.24E-02	0.0330
ST6GAL1	-1.08	1.31E-03	0.0087

SMCHD1	-1.08	5.24E-03	0.0190
ARL2BP	-1.08	4.41E-03	0.0172
SLC25A25-AS1	-1.08	3.16E-03	0.0141
BEX2	-1.08	1.28E-02	0.0336
ANKRD36B	-1.08	5.17E-03	0.0188
HMGB3P4	-1.08	1.71E-02	0.0411
CD44	-1.08	6.42E-03	0.0216
LRCH4	-1.08	1.88E-02	0.0442
AC004918.1	-1.08	1.17E-02	0.0318
AC004918.2	-1.08	1.17E-02	0.0318
ZNF677	-1.08	1.00E-02	0.0286
IRF2	-1.08	2.91E-03	0.0135
N4BP2L2	-1.08	1.20E-03	0.0084
NMI	-1.08	2.36E-03	0.0121
GATAD2B	-1.08	1.60E-03	0.0098
UBA7	-1.08	3.48E-03	0.0149
PHACTR3	-1.08	5.90E-03	0.0204
LINC01579	-1.08	1.07E-02	0.0300
TRIM14	-1.08	7.48E-03	0.0237
NLRC5	-1.08	4.79E-03	0.0180
AC004817.4	-1.08	5.78E-03	0.0202
ENST000002441740	-1.08	5.62E-03	0.0198
SERINC1	-1.08	1.20E-03	0.0084
ZNF398	-1.08	1.73E-03	0.0101
ZNF417	-1.08	5.53E-03	0.0196
CCDC80	-1.08	3.29E-03	0.0144
ADAM19	-1.08	1.39E-02	0.0357
RNF44	-1.08	3.74E-03	0.0155
IGSF6	-1.09	1.87E-02	0.0441
ENST000002640651	-1.09	1.04E-03	0.0079
CDH12	-1.09	5.76E-03	0.0202
COLCA1	-1.09	1.39E-02	0.0356
RUSC1-AS1	-1.09	2.12E-02	0.0482
AC009053.1	-1.09	3.01E-03	0.0137
SPIN2B	-1.09	4.96E-03	0.0184
MARF1	-1.09	1.66E-03	0.0100
DENND2A	-1.09	1.39E-02	0.0357
ARHGAP26	-1.09	1.46E-02	0.0369
ENST000003130281	-1.09	1.43E-02	0.0364
FBXO24	-1.09	1.67E-02	0.0405
UGT1A1	-1.09	4.20E-03	0.0166
LPIN1	-1.09	6.93E-03	0.0227
TRAJ41	-1.09	6.10E-03	0.0209
ENST000003221531	-1.09	1.03E-02	0.0292
AL590867.1	-1.09	8.34E-03	0.0254
PWWP2B	-1.09	1.61E-02	0.0394
RANBP17	-1.09	9.27E-03	0.0271
NSUN5P1	-1.09	1.43E-03	0.0092
SCHLAP1	-1.09	7.37E-03	0.0235
LINC01761	-1.09	4.25E-03	0.0168

BTD	-1.09	4.14E-03	0.0165
PRR4	-1.09	1.02E-02	0.0289
APOL6	-1.09	1.10E-02	0.0304
FAM13B	-1.09	1.28E-03	0.0087
PLEKHA8P1	-1.09	1.95E-02	0.0453
N4BP2L1	-1.10	5.23E-03	0.0190
BCL11B	-1.10	4.69E-03	0.0178
AC127070.2	-1.10	6.76E-03	0.0223
ATF7IP	-1.10	1.14E-02	0.0313
PCED1B-AS1	-1.10	3.94E-03	0.0160
ATP6V1G1	-1.10	1.62E-03	0.0098
KIAA0040	-1.10	1.18E-02	0.0320
NAP1L2	-1.10	5.85E-03	0.0204
AC078846.1	-1.10	8.38E-03	0.0255
LINC00939	-1.10	4.45E-03	0.0172
SZT2	-1.10	1.28E-03	0.0086
AC139530.1	-1.10	1.29E-02	0.0339
TULP4	-1.10	1.14E-03	0.0081
ATF6B	-1.10	4.17E-03	0.0166
TTC39B	-1.10	7.77E-03	0.0243
ERVK13-1	-1.10	1.67E-03	0.0100
IFFO1	-1.10	1.92E-03	0.0107
LINC00843	-1.10	1.38E-02	0.0355
ZMYND11	-1.10	1.52E-03	0.0095
LINC01128	-1.10	1.42E-03	0.0091
Z95114.1	-1.10	5.19E-03	0.0189
NECAP1	-1.10	2.29E-03	0.0119
ZDHHC15	-1.10	1.26E-02	0.0333
SLC30A1	-1.10	3.43E-03	0.0147
HLA-DQA1	-1.10	2.43E-03	0.0123
MYLIP	-1.10	7.21E-03	0.0231
TNIK	-1.10	6.60E-03	0.0220
IGFL4	-1.10	7.79E-03	0.0243
GKAP1	-1.10	6.86E-03	0.0225
SECISBP2L	-1.10	9.25E-03	0.0271
AC016582.3	-1.10	1.18E-02	0.0320
AP001992.1	-1.11	1.94E-02	0.0451
ENST00000641520	-1.11	6.41E-03	0.0216
ZNF570	-1.11	3.02E-03	0.0137
AL606760.3	-1.11	4.81E-03	0.0180
AC253576.2	-1.11	1.17E-02	0.0317
ABCC9	-1.11	3.16E-03	0.0141
SLC35F6	-1.11	4.69E-03	0.0178
HMGN2P28	-1.11	1.37E-02	0.0354
ZNF578	-1.11	5.56E-03	0.0197
PCSK7	-1.11	1.09E-03	0.0080
AP000347.1	-1.11	6.54E-03	0.0218
OCEL1	-1.11	8.89E-03	0.0265
TBX19	-1.11	1.39E-02	0.0356
C2CD3	-1.11	2.23E-03	0.0117

RHBDF2	-1.11	3.12E-03	0.0140
AC019131.2	-1.11	1.48E-02	0.0371
AC022893.1	-1.11	6.96E-03	0.0227
ZNF585A	-1.11	1.68E-03	0.0100
ORAI1	-1.11	1.25E-02	0.0331
MAP2K5	-1.11	2.79E-03	0.0132
AC117529.2	-1.11	9.79E-03	0.0282
ENST000002175150	-1.11	1.07E-02	0.0298
EPB41L4A	-1.11	1.35E-02	0.0350
CLN5	-1.11	1.03E-03	0.0078
LY96	-1.11	5.34E-03	0.0192
AC008115.4	-1.11	3.43E-03	0.0147
NTRK3	-1.11	1.44E-02	0.0366
MTHFD2P1	-1.11	8.84E-03	0.0264
AC098820.1	-1.11	7.98E-03	0.0247
AC007495.1	-1.11	9.48E-03	0.0275
SKIL	-1.11	1.43E-03	0.0091
ADAM32	-1.12	1.11E-03	0.0080
ENST000002961350	-1.12	1.51E-02	0.0378
RALGPS1	-1.12	8.13E-03	0.0250
AL138899.1	-1.12	3.67E-03	0.0153
AC121333.1	-1.12	5.98E-03	0.0206
ENST00000639970	-1.12	1.23E-03	0.0085
DOCK2	-1.12	7.08E-03	0.0229
AP001107.1	-1.12	9.68E-03	0.0280
NKTR	-1.12	1.00E-03	0.0077
AL137782.1	-1.12	7.95E-03	0.0247
KCNMB4	-1.12	1.90E-02	0.0444
RN7SL744P	-1.12	7.28E-03	0.0233
TMEM106B	-1.12	2.60E-03	0.0127
ENST00000640587	-1.12	1.29E-03	0.0087
BAIAP2-DT	-1.12	3.48E-03	0.0149
AL391825.1	-1.12	8.73E-03	0.0262
OR4K17	-1.12	2.04E-02	0.0468
BMPR2	-1.12	3.00E-03	0.0137
AL022344.1	-1.12	5.87E-03	0.0204
ABCD1	-1.12	9.88E-03	0.0284
SBF1	-1.12	2.85E-03	0.0134
FAM129C	-1.12	4.02E-03	0.0162
AL080317.2	-1.12	9.23E-03	0.0271
AC090912.2	-1.12	1.90E-02	0.0444
SLITRK5	-1.12	9.70E-03	0.0280
TMUB2	-1.12	8.71E-03	0.0261
AC006262.1	-1.12	1.50E-02	0.0376
AC022540.1	-1.12	6.20E-03	0.0211
PTCHD1	-1.12	6.68E-03	0.0222
AL355482.1	-1.12	3.57E-03	0.0151
CCDC97	-1.13	4.54E-03	0.0175
FBXL2	-1.13	4.61E-03	0.0176
AC004967.1	-1.13	1.36E-02	0.0352

TSIX	-1.13	1.13E-03	0.0081
LCN15	-1.13	5.26E-03	0.0190
VCL	-1.13	2.41E-03	0.0122
EML1	-1.13	1.54E-02	0.0383
AC008764.6	-1.13	1.20E-02	0.0323
DHRS12	-1.13	6.02E-03	0.0207
TTC22	-1.13	9.53E-03	0.0276
AC124312.5	-1.13	3.16E-03	0.0141
AC006141.1	-1.13	1.70E-03	0.0101
AC009135.1	-1.13	4.58E-03	0.0176
RNA5SP281	-1.13	2.08E-02	0.0475
LIFR-AS1	-1.13	3.84E-03	0.0158
AL137058.2	-1.13	1.29E-02	0.0339
MS4A2	-1.13	1.35E-02	0.0349
FO393401.1	-1.13	1.63E-02	0.0398
HYKK	-1.13	3.19E-03	0.0141
AC069222.1	-1.13	9.83E-03	0.0283
RELCH	-1.13	8.19E-04	0.0070
LINC01949	-1.13	3.29E-03	0.0144
WARS2-IT1	-1.13	1.60E-03	0.0098
CCDC66	-1.13	1.47E-03	0.0093
AL158064.1	-1.13	1.70E-03	0.0101
ACAP2	-1.13	3.93E-03	0.0160
AC106886.5	-1.13	7.18E-03	0.0231
PGBD2	-1.13	2.12E-03	0.0113
P2RX4	-1.13	4.11E-03	0.0164
LIN37	-1.13	8.49E-03	0.0257
AKR1B15	-1.13	1.06E-02	0.0298
OR2A1-AS1	-1.13	1.24E-02	0.0330
VPS13B	-1.13	9.40E-04	0.0075
AC068790.5	-1.13	1.85E-02	0.0436
ZFP36L2	-1.14	3.25E-03	0.0143
BCLAF3	-1.14	2.14E-02	0.0484
KIZ	-1.14	1.24E-03	0.0085
ARSA	-1.14	6.05E-03	0.0208
AC009120.2	-1.14	2.02E-03	0.0110
WRAP73	-1.14	4.80E-03	0.0180
AC093423.2	-1.14	1.31E-02	0.0343
RBMS1	-1.14	1.03E-03	0.0078
CYLD	-1.14	2.62E-03	0.0128
SMIM29	-1.14	1.78E-02	0.0425
LAPTM5	-1.14	4.03E-03	0.0162
PAFAH2	-1.14	2.03E-03	0.0110
FP325330.3	-1.14	1.76E-02	0.0421
OXR1	-1.14	6.13E-03	0.0209
AJ009632.2	-1.14	1.69E-02	0.0408
ZGPAT	-1.14	8.77E-03	0.0262
ENST000003173980	-1.14	4.70E-03	0.0178
LINC01268	-1.14	9.42E-03	0.0274
CPAMD8	-1.14	4.35E-03	0.0170

PRDM8	-1.14	2.18E-02	0.0491
VPS13D	-1.14	4.26E-03	0.0168
CASC15	-1.14	8.46E-03	0.0257
CPEB2	-1.14	1.09E-03	0.0080
LMBR1L	-1.14	3.15E-03	0.0141
AC109466.1	-1.14	6.99E-03	0.0228
TMEM45A	-1.15	1.68E-03	0.0100
KCNB1	-1.15	7.48E-03	0.0237
AC023051.1	-1.15	4.81E-03	0.0180
AC019117.1	-1.15	6.76E-03	0.0223
AC024075.1	-1.15	8.34E-03	0.0254
ENST000002832432	-1.15	2.15E-02	0.0486
ZMYM6	-1.15	9.42E-04	0.0075
AC109992.2	-1.15	1.08E-02	0.0301
TRAJ29	-1.15	8.16E-03	0.0251
CLEC2B	-1.15	1.20E-02	0.0323
ENST000002558820	-1.15	9.22E-03	0.0271
ZNF484	-1.15	9.25E-04	0.0075
AC016831.6	-1.15	1.38E-02	0.0356
ENST000003485130	-1.15	2.62E-03	0.0128
OCIAD2	-1.15	7.63E-04	0.0068
ENST000003739540	-1.15	1.68E-03	0.0100
PAG1	-1.15	8.55E-03	0.0258
AC239868.1	-1.15	3.65E-03	0.0153
NBPF9	-1.15	1.16E-03	0.0082
AC010168.2	-1.15	2.01E-03	0.0110
ANKRD36	-1.15	8.73E-04	0.0072
TMEM161B-AS1	-1.15	2.57E-03	0.0126
TESPA1	-1.15	8.78E-04	0.0073
DICER1-AS1	-1.15	2.83E-03	0.0133
BRWD1	-1.15	1.16E-03	0.0082
CGGBP1	-1.15	1.41E-03	0.0091
RGL2	-1.15	3.39E-03	0.0146
RCOR3	-1.15	1.46E-03	0.0093
PARVG	-1.15	1.22E-02	0.0326
GSTO2	-1.15	1.36E-02	0.0351
LINC00426	-1.15	1.54E-02	0.0383
RYR3	-1.15	8.86E-03	0.0264
ENST000002778740	-1.15	1.52E-02	0.0379
PLCD4	-1.15	1.45E-02	0.0368
AC012467.2	-1.15	1.32E-02	0.0343
STX17	-1.16	1.12E-02	0.0308
RHOH	-1.16	8.85E-04	0.0073
AC026150.1	-1.16	1.86E-02	0.0438
AC254960.1	-1.16	1.86E-02	0.0438
ASPRV1	-1.16	1.98E-02	0.0458
TLE4	-1.16	5.53E-03	0.0196
SYT12	-1.16	1.60E-02	0.0393
FAM66C	-1.16	9.12E-03	0.0269
RSF1-IT2	-1.16	1.97E-02	0.0456

PPIAP46	-1.16	3.09E-03	0.0140
CASZ1	-1.16	1.80E-02	0.0428
LENG8	-1.16	2.28E-03	0.0118
SPTLC3	-1.16	4.53E-03	0.0174
HSP90AB4P	-1.16	4.73E-03	0.0179
RASGRP1	-1.16	5.78E-03	0.0202
AC092747.4	-1.16	4.84E-03	0.0181
LINC01515	-1.16	3.79E-03	0.0157
DNAJC22	-1.16	1.09E-02	0.0303
BX004987.1	-1.16	1.16E-02	0.0316
R3HDM2	-1.16	1.89E-03	0.0106
APOBR	-1.16	1.63E-02	0.0399
ARHGAP4	-1.16	1.87E-03	0.0105
AL358334.2	-1.16	2.08E-02	0.0474
LINC01481	-1.16	8.36E-03	0.0255
KIR3DX1	-1.16	8.91E-03	0.0265
AKR7L	-1.16	8.34E-03	0.0254
CCDC191	-1.16	6.99E-03	0.0228
KIAA2012	-1.16	1.73E-02	0.0415
OR2T12	-1.16	2.78E-03	0.0132
ARHGAP5	-1.16	2.36E-03	0.0121
ZC3H6	-1.16	1.38E-02	0.0354
LINC01427	-1.16	7.87E-03	0.0245
TNRC6B	-1.16	7.02E-04	0.0065
AL157786.1	-1.16	9.75E-03	0.0281
OR2A5	-1.16	3.09E-03	0.0140
MLXIP	-1.16	4.95E-03	0.0184
TMEM175	-1.17	3.16E-03	0.0141
NBPF25P	-1.17	3.11E-03	0.0140
ENST000002985644	-1.17	1.46E-02	0.0369
AMZ2P1	-1.17	1.08E-02	0.0300
GLRX	-1.17	1.95E-03	0.0108
ENST000002528400	-1.17	1.93E-03	0.0107
TPGS1	-1.17	9.41E-03	0.0274
RPL21P135	-1.17	1.35E-02	0.0350
AC008267.2	-1.17	1.96E-02	0.0455
TRPM3	-1.17	6.45E-03	0.0217
GARS-DT	-1.17	1.95E-03	0.0108
STXBP5	-1.17	2.52E-03	0.0125
MAS1	-1.17	1.81E-02	0.0430
CCDC84	-1.17	5.67E-03	0.0199
AC145285.2	-1.17	8.33E-03	0.0254
ENST00000440190	-1.17	1.03E-02	0.0291
AC027514.1	-1.17	7.27E-03	0.0233
ZNF280D	-1.17	4.03E-03	0.0162
ENST00000606754	-1.17	2.13E-02	0.0483
AC007390.2	-1.17	2.82E-03	0.0133
AC017099.1	-1.17	1.04E-03	0.0079
AP5Z1	-1.17	8.07E-03	0.0249
ENST000002568580	-1.17	5.27E-03	0.0190

Z82217.1	-1.17	3.82E-03	0.0157
STIM2	-1.17	2.02E-03	0.0110
OFD1	-1.17	2.58E-03	0.0126
ZNF383	-1.17	1.24E-03	0.0085
ZNF362	-1.17	7.24E-03	0.0232
MIR4453HG	-1.17	3.05E-03	0.0139
LNCARSR	-1.17	1.46E-02	0.0368
OR2M3	-1.17	1.52E-03	0.0095
MTHFR	-1.18	1.35E-03	0.0089
ENST000002329782	-1.18	4.31E-03	0.0169
SNHG14	-1.18	6.83E-04	0.0064
ENST000003177750	-1.18	1.35E-02	0.0350
S100A4	-1.18	5.94E-03	0.0205
TMEM191C	-1.18	1.25E-02	0.0331
AC022762.2	-1.18	1.45E-02	0.0367
ENST000002511701	-1.18	1.66E-02	0.0404
SNURF	-1.18	3.22E-03	0.0142
HELQ	-1.18	1.11E-03	0.0081
LGSN	-1.18	1.94E-02	0.0451
TBC1D10A	-1.18	1.52E-03	0.0095
SERPINB9P1	-1.18	6.22E-03	0.0211
Z98755.1	-1.18	1.06E-02	0.0297
NDUFV2-AS1	-1.18	9.97E-03	0.0285
ZNF546	-1.18	8.47E-04	0.0071
AC124804.1	-1.18	1.61E-02	0.0395
TBC1D8B	-1.18	3.17E-03	0.0141
TTC16	-1.18	5.90E-03	0.0204
ZFAND2B	-1.18	2.73E-03	0.0131
AC107959.1	-1.18	1.93E-02	0.0450
THSD4	-1.19	4.10E-03	0.0164
ZNF471	-1.19	1.18E-03	0.0083
AC009137.2	-1.19	4.86E-03	0.0181
AL133520.1	-1.19	1.62E-02	0.0397
PCDHGA6	-1.19	1.62E-02	0.0397
RF00019.708	-1.19	8.07E-03	0.0249
AK3P3	-1.19	1.61E-02	0.0395
RF00017.190	-1.19	7.55E-03	0.0238
AC136475.1	-1.19	1.64E-02	0.0399
ZCWPW1	-1.19	6.03E-03	0.0207
AKAP13	-1.19	1.90E-03	0.0106
ARID1B	-1.19	1.30E-03	0.0087
TTC28-AS1	-1.19	3.33E-03	0.0145
ENST000002234590	-1.19	9.71E-03	0.0280
ATP10B	-1.19	5.06E-03	0.0186
INTS6-AS1	-1.19	2.93E-03	0.0135
PPP1R13B	-1.19	7.19E-03	0.0231
ENST000002964221	-1.19	2.17E-02	0.0489
TRAJ23	-1.19	9.20E-03	0.0270
DOCK11	-1.19	1.89E-03	0.0106
AC009961.1	-1.19	3.51E-03	0.0149

LHFPL5	-1.19	2.63E-03	0.0128
ANKRD12	-1.20	1.17E-03	0.0083
CAMK2G	-1.20	1.71E-03	0.0101
SNORA74B	-1.20	9.82E-03	0.0283
DNAH10OS	-1.20	2.03E-02	0.0467
AC009148.1	-1.20	5.49E-03	0.0196
ENST000003165090	-1.20	1.03E-02	0.0290
SULT1C2	-1.20	1.93E-02	0.0450
GAB2	-1.20	2.21E-02	0.0496
GABRG2	-1.20	4.94E-03	0.0183
AL031118.1	-1.20	2.85E-03	0.0134
MTMR3	-1.20	3.65E-03	0.0153
ENST00000492128	-1.20	8.76E-03	0.0262
MYBL1	-1.20	3.81E-03	0.0157
TRAJ6	-1.20	3.45E-03	0.0148
AC008840.1	-1.20	1.69E-02	0.0408
CTSC	-1.20	7.17E-04	0.0065
AP002812.5	-1.20	1.00E-02	0.0286
ENST000003098630	-1.20	1.07E-02	0.0298
AL731563.3	-1.20	1.27E-02	0.0336
WDR7	-1.20	6.16E-04	0.0061
TRIM13	-1.20	6.16E-04	0.0061
RN7SKP250	-1.20	1.08E-02	0.0302
AC135048.1	-1.20	1.17E-02	0.0318
AC009120.5	-1.20	3.60E-03	0.0152
ZNF846	-1.20	3.03E-03	0.0138
R3HDM4	-1.20	3.12E-03	0.0140
ENTPD4	-1.20	1.31E-03	0.0087
ATP8A1	-1.20	2.33E-03	0.0120
AC068338.2	-1.20	7.19E-03	0.0231
BTG1	-1.20	4.58E-03	0.0176
AC110716.2	-1.20	2.23E-02	0.0499
CSGALNACT2	-1.21	1.94E-03	0.0107
MANBA	-1.21	1.29E-03	0.0087
MBP	-1.21	1.61E-03	0.0098
SCARNA6	-1.21	1.04E-02	0.0294
RGS17P1	-1.21	2.16E-02	0.0487
AL645568.1	-1.21	2.54E-03	0.0126
NEK10	-1.21	9.73E-03	0.0281
ENST000003114591	-1.21	1.85E-03	0.0105
ZSWIM7	-1.21	1.17E-02	0.0318
AC008124.1	-1.21	8.35E-03	0.0254
TRAJ11	-1.21	2.03E-02	0.0466
KCNG4	-1.21	9.91E-03	0.0284
NAV1	-1.21	4.78E-03	0.0180
NMT2	-1.21	3.20E-03	0.0142
LO000005.1	-1.21	1.84E-03	0.0105
AP000873.4	-1.21	1.70E-02	0.0410
AL591684.2	-1.21	1.58E-02	0.0389
CAPN1	-1.21	3.27E-03	0.0143

AC104564.5	-1.21	4.61E-03	0.0176
LINC00933	-1.21	2.23E-02	0.0499
IQCM	-1.21	5.56E-03	0.0197
N6AMT1	-1.21	2.13E-03	0.0114
KDM7A	-1.21	2.71E-03	0.0130
RN7SL608P	-1.21	9.78E-03	0.0282
RN7SKP9	-1.21	6.18E-03	0.0210
C17orf100	-1.21	2.57E-03	0.0126
FLRT2	-1.21	2.90E-03	0.0135
ATG16L1	-1.21	1.14E-03	0.0081
SLC9B1	-1.22	5.69E-03	0.0200
SULT1A3	-1.22	5.88E-03	0.0204
SNTB1	-1.22	7.25E-03	0.0232
LINC02334	-1.22	1.12E-02	0.0309
OR52A1	-1.22	5.15E-03	0.0188
AC090517.2	-1.22	1.27E-02	0.0334
TMEM104	-1.22	1.49E-02	0.0374
AC009318.3	-1.22	7.70E-03	0.0241
TRAJ19	-1.22	4.88E-03	0.0182
ENST000000546660	-1.22	9.72E-04	0.0076
ENST000002730090	-1.22	5.95E-03	0.0206
CFAP54	-1.22	2.35E-03	0.0121
AC012349.1	-1.22	2.12E-03	0.0113
UBQLN2	-1.22	2.35E-03	0.0121
AC005304.2	-1.22	2.56E-03	0.0126
TRAJ27	-1.22	1.18E-02	0.0320
ENST000003246771	-1.22	1.13E-02	0.0310
ADI1	-1.22	7.93E-04	0.0069
REPS2	-1.22	6.41E-03	0.0215
GPS2	-1.22	5.73E-03	0.0201
LINC01722	-1.22	1.16E-02	0.0316
DOP1B	-1.22	5.46E-03	0.0195
MAP3K3	-1.22	2.24E-03	0.0117
AC007878.1	-1.22	4.24E-03	0.0167
AC079341.1	-1.22	3.07E-03	0.0139
AC091185.1	-1.22	6.84E-03	0.0225
TRAJ20	-1.23	2.38E-03	0.0121
ADAMTSL3	-1.23	2.87E-03	0.0134
AL450384.2	-1.23	1.56E-03	0.0096
RNF103	-1.23	1.00E-03	0.0077
36951	-1.23	1.91E-02	0.0446
VPS13C	-1.23	5.94E-04	0.0060
GLCCI1	-1.23	5.87E-03	0.0204
SLC17A5	-1.23	9.86E-04	0.0077
SLC7A14	-1.23	4.70E-03	0.0178
TMC8	-1.23	4.00E-03	0.0161
RNF216	-1.23	1.92E-03	0.0107
TMEM154	-1.23	7.25E-03	0.0232
NBPF12	-1.23	2.32E-03	0.0120
AC002553.2	-1.23	4.67E-03	0.0178

SCN7A	-1.23	2.19E-02	0.0493
TRAV21	-1.23	3.55E-03	0.0150
ENST000002460691	-1.23	2.01E-02	0.0463
ASPA	-1.23	1.60E-03	0.0097
LINC01355	-1.23	9.67E-04	0.0076
TCTA	-1.23	5.37E-03	0.0193
EPB41	-1.23	2.37E-03	0.0121
CTSK	-1.23	3.96E-03	0.0161
RNPC3	-1.23	1.02E-03	0.0078
ARNTL	-1.23	1.64E-02	0.0399
AC138904.3	-1.23	5.87E-03	0.0204
TRAJ16	-1.23	8.50E-03	0.0257
MOGAT3	-1.23	3.84E-03	0.0158
ABHD13	-1.23	6.14E-04	0.0061
FOCAD	-1.23	1.67E-02	0.0405
ENST000003410711	-1.24	3.36E-03	0.0146
AC018521.7	-1.24	7.56E-03	0.0239
AC008938.1	-1.24	4.65E-03	0.0177
SEC14L6	-1.24	2.19E-02	0.0493
SCAMP1-AS1	-1.24	1.63E-03	0.0099
PKD2	-1.24	1.33E-02	0.0345
AC005498.3	-1.24	9.75E-03	0.0281
TRAJ26	-1.24	6.61E-03	0.0220
PSMA3-AS1	-1.24	6.44E-04	0.0062
HUNK	-1.24	9.33E-03	0.0273
AC079684.1	-1.24	5.21E-03	0.0190
ZNF852	-1.24	1.10E-03	0.0080
OR5A1	-1.24	2.07E-02	0.0473
AL135999.1	-1.24	1.10E-02	0.0304
NFIA	-1.24	5.95E-03	0.0205
RPRD2	-1.24	1.12E-03	0.0081
CYP46A1	-1.24	9.57E-04	0.0076
C12orf50	-1.24	1.95E-03	0.0108
RREB1	-1.24	1.73E-03	0.0102
AGER	-1.24	1.25E-02	0.0331
DGCR6	-1.24	2.18E-02	0.0491
AL133372.2	-1.24	1.65E-02	0.0402
LINC01653	-1.24	5.18E-03	0.0189
OR2AT4	-1.25	1.82E-03	0.0104
ZNF233	-1.25	6.67E-03	0.0221
HEXD	-1.25	1.78E-03	0.0103
SEPSECS-AS1	-1.25	1.00E-03	0.0077
CYTH4	-1.25	9.89E-03	0.0284
GBP4	-1.25	3.46E-03	0.0148
CLDND1	-1.25	1.93E-03	0.0107
USP3	-1.25	8.08E-04	0.0070
NAA60	-1.25	2.48E-03	0.0124
GMIP	-1.25	1.35E-03	0.0089
ENST000002735501	-1.25	3.70E-03	0.0154
RNF157	-1.25	2.14E-03	0.0114

SMG1P3	-1.25	1.60E-02	0.0393
AP001429.1	-1.25	6.03E-03	0.0207
TRAJ37	-1.25	1.06E-02	0.0297
TRIM22	-1.25	3.04E-03	0.0138
OR10K1	-1.25	7.01E-03	0.0228
ANKAR	-1.25	1.74E-03	0.0102
ENST000002562550	-1.25	2.75E-03	0.0131
ZSCAN26	-1.25	1.50E-03	0.0094
GCNT3	-1.25	8.03E-03	0.0248
ENST000003212502	-1.25	9.07E-04	0.0074
TSBP1-AS1	-1.25	2.57E-03	0.0126
MAPK8IP3	-1.25	6.80E-04	0.0064
ENST000003350252	-1.25	1.85E-03	0.0105
AC068587.4	-1.26	1.36E-03	0.0089
CCDC186	-1.26	9.37E-04	0.0075
U2AF1L4	-1.26	4.31E-03	0.0169
AC006946.2	-1.26	2.78E-03	0.0132
HIGD1AP16	-1.26	2.04E-02	0.0468
TUT4	-1.26	6.70E-04	0.0064
VGLL3	-1.26	1.28E-02	0.0336
TLR2	-1.26	1.44E-02	0.0366
AC114776.1	-1.26	1.55E-02	0.0385
AC023509.3	-1.26	4.66E-03	0.0177
AL592437.2	-1.26	1.00E-02	0.0286
AP003774.4	-1.26	8.93E-03	0.0265
ENST00000639349	-1.26	8.93E-03	0.0265
TRMO	-1.26	7.28E-04	0.0066
KIAA2026	-1.26	7.59E-04	0.0067
DNM1P47	-1.26	1.62E-03	0.0098
RF00019.470	-1.27	1.30E-02	0.0339
CD47	-1.27	2.30E-03	0.0119
ZNF319	-1.27	4.46E-03	0.0173
ARPIN	-1.27	6.00E-03	0.0207
ENST000002482110	-1.27	3.08E-03	0.0139
AP000350.10	-1.27	5.59E-03	0.0197
AP000350.7	-1.27	5.59E-03	0.0197
ZBTB40	-1.27	7.13E-04	0.0065
SH3YL1	-1.27	2.03E-03	0.0110
AC079466.1	-1.27	3.97E-03	0.0161
HCG27	-1.27	1.96E-02	0.0456
BLCAP	-1.27	2.05E-03	0.0111
AC243960.11	-1.27	5.86E-03	0.0204
FBXW8	-1.27	3.03E-03	0.0138
LRRC74B	-1.27	1.96E-03	0.0108
PCAT14	-1.27	7.08E-03	0.0229
ARHGAP12	-1.27	6.30E-03	0.0213
AC087473.1	-1.27	5.79E-04	0.0060
AC108463.3	-1.27	1.51E-02	0.0378
AC092802.1	-1.27	3.29E-03	0.0144
SHISA9	-1.27	1.68E-02	0.0407

RASAL3	-1.27	7.10E-03	0.0230
CLK4	-1.27	1.17E-03	0.0083
SNORD116-13	-1.27	1.31E-02	0.0342
ENST000002960881	-1.27	7.91E-03	0.0246
ENST00000623130	-1.28	5.46E-04	0.0058
MAN1A2	-1.28	1.05E-03	0.0079
ZBTB41	-1.28	9.52E-04	0.0076
RPL7P49	-1.28	1.48E-02	0.0372
WDFY2	-1.28	8.58E-04	0.0072
AC139494.4	-1.28	2.23E-02	0.0499
RORA	-1.28	3.44E-03	0.0148
XKR4	-1.28	2.23E-02	0.0499
GRIA1	-1.28	1.68E-02	0.0407
KANSL1L	-1.28	1.92E-03	0.0107
RN7SKP173	-1.28	1.87E-02	0.0439
AC022400.6	-1.28	1.26E-02	0.0334
UPP1	-1.28	4.75E-03	0.0179
ANKRD44	-1.28	6.36E-04	0.0062
AL035563.1	-1.28	6.99E-03	0.0228
AC104964.2	-1.28	1.29E-02	0.0339
TRAJ4	-1.28	3.39E-03	0.0147
MR1	-1.28	8.81E-04	0.0073
TRDC	-1.28	4.64E-03	0.0177
LINC02193	-1.28	2.93E-03	0.0135
GABRD	-1.28	2.83E-03	0.0133
AL139147.1	-1.28	4.29E-03	0.0169
ZBTB4	-1.28	9.76E-04	0.0076
ZNF528	-1.28	7.97E-04	0.0069
OR56A1	-1.28	1.18E-02	0.0319
AC034236.2	-1.28	1.86E-02	0.0438
AL136146.2	-1.28	7.18E-03	0.0231
CCPG1	-1.28	3.55E-03	0.0150
AC243960.1	-1.28	6.30E-03	0.0213
AC018845.3	-1.28	1.07E-02	0.0298
ACYP2	-1.28	1.27E-03	0.0086
LCP2	-1.28	1.22E-03	0.0084
BIN1	-1.29	2.33E-03	0.0120
ENST000002505593	-1.29	6.34E-04	0.0062
WHAMMP3	-1.29	2.70E-03	0.0130
SCARNA21	-1.29	4.01E-03	0.0162
NOD1	-1.29	1.56E-03	0.0096
LINC01630	-1.29	1.37E-02	0.0353
ENST000003173611	-1.29	2.18E-02	0.0491
AKNA	-1.29	1.42E-03	0.0091
TRBV11-2	-1.29	6.68E-03	0.0222
AC005224.1	-1.29	1.43E-02	0.0364
LINC02577	-1.29	9.31E-03	0.0272
MEF2D	-1.29	1.81E-03	0.0104
ZNF547	-1.29	2.82E-03	0.0133
AL034417.3	-1.29	3.61E-03	0.0152

RN7SKP180	-1.29	1.93E-02	0.0450
CNST	-1.29	1.13E-03	0.0081
AL021368.2	-1.29	8.87E-04	0.0073
AC022182.2	-1.30	4.07E-03	0.0163
LINC00667	-1.30	1.09E-02	0.0303
PAN2	-1.30	1.47E-03	0.0093
AMT	-1.30	1.21E-02	0.0324
CAPRIN2	-1.30	8.44E-04	0.0071
LINC00547	-1.30	7.31E-03	0.0234
CDC42SE1	-1.30	4.99E-04	0.0056
PTPRJ	-1.30	6.59E-04	0.0063
AC103957.2	-1.30	1.47E-02	0.0370
C3orf62	-1.30	1.70E-03	0.0101
CROCC	-1.30	1.28E-02	0.0337
ZNF566	-1.30	1.06E-03	0.0079
ZFYVE28	-1.30	1.72E-02	0.0413
LINC00670	-1.30	1.85E-03	0.0105
AP002807.1	-1.30	2.29E-03	0.0119
ATP2B4	-1.30	1.41E-03	0.0091
VAMP4	-1.30	1.91E-02	0.0446
PCNX1	-1.30	4.22E-03	0.0167
ORMDL3	-1.30	1.47E-03	0.0093
ENST00000640719	-1.30	2.42E-03	0.0122
AL049779.1	-1.30	7.11E-03	0.0230
ZNF224	-1.30	5.79E-04	0.0060
FAM228B	-1.30	7.19E-04	0.0065
TECRL	-1.31	5.09E-03	0.0187
ENST000003147422	-1.31	6.94E-03	0.0227
CLHC1	-1.31	1.13E-02	0.0311
GNRH1	-1.31	1.63E-03	0.0099
RNASET2	-1.31	9.94E-03	0.0285
CBFA2T2	-1.31	1.11E-03	0.0081
OR1A1	-1.31	1.36E-03	0.0089
ENST000003735040	-1.31	1.10E-03	0.0080
APOL4	-1.31	3.91E-03	0.0160
AC080013.1	-1.31	1.07E-02	0.0298
ATCAY	-1.31	8.58E-03	0.0259
CRYGS	-1.31	3.14E-03	0.0140
AL021368.4	-1.31	4.23E-03	0.0167
AL138889.1	-1.31	3.25E-03	0.0143
HNRNPA1P54	-1.31	1.18E-03	0.0083
INPP5D	-1.31	1.01E-03	0.0078
USP46	-1.31	7.68E-04	0.0068
C3orf35	-1.31	2.57E-03	0.0126
AC092279.1	-1.31	2.18E-03	0.0115
PDLIM2	-1.31	1.81E-03	0.0104
GPC2	-1.31	2.05E-02	0.0470
HTN3	-1.32	9.84E-03	0.0283
ENST000002160191	-1.32	1.31E-03	0.0087
ZNF385C	-1.32	1.36E-02	0.0352

TEX41	-1.32	8.27E-03	0.0253
SCARNA2	-1.32	1.43E-02	0.0364
AL358178.1	-1.32	1.75E-02	0.0419
LINC00623	-1.32	1.05E-02	0.0294
SKI	-1.32	1.47E-02	0.0370
ERVK9-11	-1.32	7.78E-03	0.0243
DIRC3	-1.32	4.69E-03	0.0178
RNASEL	-1.32	5.25E-03	0.0190
SLC30A4	-1.32	2.62E-03	0.0128
AC010632.2	-1.32	1.11E-02	0.0307
TRAJ22	-1.32	1.35E-03	0.0089
AC091078.3	-1.32	2.84E-03	0.0134
WASHC2C	-1.32	5.86E-04	0.0060
CCDC17	-1.32	2.67E-03	0.0129
PLEKHM1	-1.32	3.89E-03	0.0159
ENST00000640347	-1.32	6.50E-04	0.0063
AC119428.2	-1.32	1.17E-02	0.0318
TPP1	-1.32	2.75E-03	0.0131
AC004812.2	-1.32	1.99E-03	0.0109
SLC12A9	-1.32	2.36E-03	0.0121
CRTC3	-1.32	8.73E-04	0.0072
ENST00000640720	-1.32	9.79E-04	0.0077
SCARNA7	-1.32	2.26E-03	0.0118
AL162586.1	-1.32	1.87E-02	0.0440
PCSK5	-1.32	1.10E-02	0.0304
AC092944.1	-1.32	2.22E-03	0.0117
WWP1	-1.32	7.05E-04	0.0065
ZNF181	-1.32	9.61E-04	0.0076
EIF4E3	-1.32	5.58E-03	0.0197
PAXIP1-AS2	-1.32	3.18E-03	0.0141
ZBED5-AS1	-1.33	5.15E-03	0.0188
TRAPPC9	-1.33	1.37E-03	0.0089
ENST000002193430	-1.33	1.22E-02	0.0327
AC002519.1	-1.33	1.87E-02	0.0440
POU5F2	-1.33	5.83E-04	0.0060
RSRP1	-1.33	2.24E-03	0.0117
GSTK1	-1.33	4.73E-04	0.0055
TAB1	-1.33	2.04E-02	0.0468
NXPE3	-1.33	2.80E-03	0.0133
AC097634.1	-1.33	7.34E-03	0.0234
MPP3	-1.33	1.28E-02	0.0337
RNF166	-1.33	4.54E-03	0.0175
AL080317.3	-1.33	1.68E-03	0.0100
LINC01970	-1.33	1.44E-02	0.0366
KCNE4	-1.33	3.16E-03	0.0141
PLEKHO1	-1.33	6.98E-03	0.0228
PAQR8	-1.33	7.15E-03	0.0231
TSNAX-DISC1	-1.33	1.47E-03	0.0093
ING4	-1.33	1.41E-03	0.0091
FITM2	-1.33	3.92E-03	0.0160

LINC01776	-1.33	3.26E-03	0.0143
HSPBAP1	-1.33	8.66E-03	0.0260
CRYBG1	-1.33	9.17E-04	0.0074
AL353743.1	-1.33	1.30E-03	0.0087
HS3ST3B1	-1.34	8.01E-04	0.0069
AC010531.3	-1.34	6.71E-03	0.0222
AC002451.1	-1.34	1.61E-02	0.0395
ATAD2B	-1.34	5.88E-04	0.0060
AC009404.1	-1.34	8.15E-03	0.0251
LINC01934	-1.34	1.29E-02	0.0338
CCNT2	-1.34	4.00E-04	0.0051
AC044810.9	-1.34	5.10E-03	0.0187
GSTM2	-1.34	4.10E-03	0.0164
ENST000003415110	-1.34	6.25E-03	0.0212
ENST000003080080	-1.34	3.20E-03	0.0142
NPIPB3	-1.34	1.43E-03	0.0091
LDHAL6CP	-1.34	4.91E-03	0.0183
AC021016.2	-1.34	7.92E-03	0.0246
PGBD5	-1.34	1.39E-02	0.0357
C1QTNF3	-1.34	3.10E-03	0.0140
LINC00937	-1.34	6.16E-03	0.0210
AL139220.1	-1.34	1.27E-02	0.0335
Z93930.2	-1.34	1.91E-03	0.0106
AC099792.1	-1.34	4.61E-03	0.0176
KPNA5	-1.34	4.29E-04	0.0052
PDHB	-1.34	5.40E-03	0.0194
FUT2	-1.34	4.98E-03	0.0184
AL022323.4	-1.34	3.74E-03	0.0155
ABHD3	-1.34	2.00E-03	0.0109
PODNL1	-1.34	1.61E-02	0.0395
LINC00550	-1.34	9.42E-03	0.0274
OR7C1	-1.34	2.05E-03	0.0111
TMC7	-1.34	6.24E-03	0.0212
NAGK	-1.34	5.38E-04	0.0058
ABO	-1.35	5.82E-03	0.0203
BICD1	-1.35	2.19E-02	0.0493
LINC00504	-1.35	1.25E-03	0.0086
MOB2	-1.35	1.09E-03	0.0080
OS9	-1.35	4.52E-03	0.0174
BTN3A3	-1.35	1.64E-02	0.0400
OR2I1P	-1.35	1.37E-02	0.0353
TMC3	-1.35	1.38E-02	0.0355
RELL1	-1.35	9.66E-04	0.0076
AC058823.1	-1.35	1.52E-02	0.0379
ENST000002738530	-1.35	5.91E-04	0.0060
GOLGA8B	-1.35	4.53E-04	0.0054
FLJ37035	-1.35	1.39E-02	0.0357
BTNL9	-1.35	1.02E-02	0.0289
TPRG1L	-1.35	1.06E-03	0.0079
AC026979.2	-1.35	4.35E-03	0.0170

ANKRD20A5P	-1.35	2.92E-03	0.0135
ARAF	-1.35	4.16E-03	0.0165
ARRDC2	-1.35	1.85E-03	0.0105
ZNF493	-1.35	5.02E-03	0.0185
LCORL	-1.35	6.27E-04	0.0062
AC096543.1	-1.35	7.63E-03	0.0240
AC087359.1	-1.36	5.87E-03	0.0204
ZNF550	-1.36	4.27E-04	0.0052
NANOG	-1.36	3.46E-03	0.0148
TRAJ18	-1.36	1.23E-03	0.0085
TBC1D25	-1.36	1.45E-03	0.0092
ZNF737	-1.36	3.58E-03	0.0151
ST3GAL1	-1.36	4.94E-04	0.0056
AC239799.1	-1.36	1.36E-03	0.0089
PRR12	-1.36	2.13E-03	0.0113
AC034213.1	-1.36	5.51E-03	0.0196
TRAJ28	-1.36	5.84E-03	0.0203
ZCWPW2	-1.36	7.20E-03	0.0231
AC103760.1	-1.36	2.13E-03	0.0113
ZNF528-AS1	-1.36	2.45E-03	0.0123
FLJ45513	-1.36	7.00E-03	0.0228
ENST000003352510	-1.36	4.71E-04	0.0054
TNFRSF25	-1.36	8.02E-04	0.0069
AC121758.1	-1.36	2.91E-03	0.0135
AC096644.4	-1.36	1.87E-02	0.0440
THEMIS2	-1.36	4.34E-04	0.0052
ERVW-1	-1.36	5.93E-03	0.0205
MAP3K1	-1.36	2.36E-03	0.0121
VPS8	-1.36	6.72E-04	0.0064
AL139220.2	-1.36	1.81E-03	0.0104
ZBTB37	-1.36	4.77E-04	0.0055
NUTM2B-AS1	-1.36	4.23E-04	0.0052
TTC34	-1.37	7.17E-03	0.0231
AL022310.1	-1.37	8.14E-03	0.0251
RGS17	-1.37	2.68E-03	0.0129
NUCB2	-1.37	1.26E-03	0.0086
PI4KA	-1.37	6.57E-04	0.0063
MC1R	-1.37	1.15E-02	0.0313
AIDA	-1.37	5.42E-03	0.0194
AP006222.1	-1.37	1.37E-03	0.0089
ZKSCAN3	-1.37	3.08E-03	0.0139
MIR548AJ2	-1.37	1.04E-02	0.0294
L3MBTL3	-1.37	3.82E-03	0.0157
TMEM198B	-1.37	1.65E-02	0.0402
NBPF2P	-1.37	1.31E-02	0.0342
ENST000003430030	-1.37	5.63E-04	0.0059
CR382287.2	-1.37	3.20E-03	0.0142
HCST	-1.37	1.09E-02	0.0303
ZNF699	-1.37	1.64E-03	0.0099
GPR55	-1.37	2.26E-03	0.0118

AP002008.3	-1.37	9.11E-03	0.0269
LINC01534	-1.37	1.55E-03	0.0096
RN7SL166P	-1.37	8.15E-03	0.0251
ENST000003898570	-1.37	4.40E-04	0.0053
PCNX2	-1.37	4.79E-03	0.0180
INPP5K	-1.37	5.61E-03	0.0198
MTMR10	-1.37	3.99E-04	0.0051
AL049840.5	-1.37	4.69E-03	0.0178
TEX35	-1.37	1.41E-03	0.0091
BCO2	-1.38	2.61E-03	0.0127
TPT1-AS1	-1.38	2.67E-03	0.0129
RN7SL650P	-1.38	1.77E-02	0.0423
TRAJ32	-1.38	1.87E-03	0.0105
POTEC	-1.38	3.09E-03	0.0140
HEXD-IT1	-1.38	1.91E-03	0.0107
MYLK4	-1.38	7.94E-03	0.0246
ACVR2B-AS1	-1.38	1.05E-02	0.0296
SCART1	-1.38	3.30E-03	0.0144
ZXDB	-1.38	9.87E-04	0.0077
KIFC2	-1.38	1.03E-03	0.0078
RN7SKP70	-1.38	9.52E-03	0.0276
C8orf34	-1.38	5.29E-03	0.0191
AC123768.3	-1.38	1.96E-02	0.0455
OR2A9P	-1.38	7.46E-03	0.0237
OSBPL7	-1.38	1.23E-03	0.0085
RDH16	-1.38	2.46E-03	0.0123
TRAJ38	-1.38	2.18E-02	0.0491
SNX29	-1.38	1.67E-03	0.0100
PPM1L	-1.39	1.31E-02	0.0341
ADH4	-1.39	6.52E-03	0.0218
MIA3	-1.39	5.17E-03	0.0188
RCAN3	-1.39	6.39E-03	0.0215
ENST000002433492	-1.39	1.88E-02	0.0441
TTC39C	-1.39	8.75E-04	0.0072
AC034102.4	-1.39	1.88E-02	0.0441
AC027097.1	-1.39	1.23E-03	0.0085
ADPRM	-1.39	1.20E-03	0.0084
CCDC91	-1.39	4.66E-04	0.0054
AC128685.1	-1.39	2.86E-03	0.0134
ANKMY1	-1.39	8.33E-04	0.0071
ZNF350	-1.39	5.05E-04	0.0056
AC012435.1	-1.39	5.62E-03	0.0198
PLCD1	-1.39	1.08E-02	0.0300
BICRAL	-1.39	6.71E-04	0.0064
ZNF708	-1.39	5.14E-04	0.0057
ENST000002646580	-1.39	8.82E-04	0.0073
AC010186.2	-1.39	1.66E-03	0.0099
AL158212.3	-1.39	1.31E-03	0.0087
SELL	-1.39	5.73E-04	0.0059
YPEL1	-1.39	1.85E-02	0.0436

DYNAP	-1.40	1.08E-02	0.0301
CD44-AS1	-1.40	3.01E-03	0.0137
ZNF577	-1.40	8.99E-03	0.0267
C5AR2	-1.40	8.09E-03	0.0250
RPS15AP10	-1.40	3.60E-03	0.0152
AC008770.3	-1.40	1.19E-02	0.0321
AC097376.2	-1.40	8.42E-04	0.0071
LPAR2	-1.40	1.90E-03	0.0106
ENST000003215213	-1.40	2.04E-03	0.0110
TTC12	-1.41	2.74E-03	0.0131
CD52	-1.41	6.02E-03	0.0207
B4GALNT2	-1.41	1.83E-03	0.0104
IL6R	-1.41	4.99E-04	0.0056
CCDC26	-1.41	8.14E-04	0.0070
STEAP4	-1.41	6.11E-03	0.0209
ENST000003241030	-1.41	8.83E-03	0.0264
37316	-1.41	1.05E-03	0.0079
ENST000003160051	-1.41	2.97E-03	0.0136
RPL34-AS1	-1.41	2.36E-03	0.0121
AP000866.1	-1.41	2.49E-03	0.0124
AC243922.3	-1.41	9.47E-04	0.0076
LINC01873	-1.41	1.01E-03	0.0078
AL732372.2	-1.41	7.73E-03	0.0242
AC073957.3	-1.41	5.06E-03	0.0186
AP003774.3	-1.41	1.33E-02	0.0345
TRAJ51	-1.41	1.83E-02	0.0433
KRT8P33	-1.41	1.32E-02	0.0343
TBCC	-1.41	8.30E-04	0.0071
MYO3B-AS1	-1.41	6.62E-03	0.0220
CYTH1	-1.41	7.53E-04	0.0067
GMDS-DT	-1.41	8.59E-04	0.0072
TACC2	-1.41	3.23E-03	0.0142
POF1B	-1.41	2.85E-03	0.0134
AL157838.1	-1.41	1.22E-02	0.0326
CBX7	-1.41	1.65E-03	0.0099
AP005432.1	-1.41	9.19E-03	0.0270
AC024257.3	-1.41	2.35E-03	0.0121
CASP4	-1.41	9.20E-04	0.0075
MEGF9	-1.42	1.04E-03	0.0079
CC2D1B	-1.42	5.13E-04	0.0057
FAM242A	-1.42	4.63E-03	0.0177
DNAJC4	-1.42	1.38E-02	0.0356
39508	-1.42	7.12E-04	0.0065
AC139795.1	-1.42	2.13E-03	0.0113
ATP9B	-1.42	5.88E-04	0.0060
ALOX15P1	-1.42	1.15E-02	0.0314
AL162578.1	-1.42	1.11E-02	0.0306
SNORA16B	-1.42	2.20E-02	0.0494
LINC00506	-1.42	5.98E-04	0.0060
ZNF517	-1.42	1.51E-03	0.0094

TMEM260	-1.42	3.01E-03	0.0137
AC096711.2	-1.42	6.22E-03	0.0211
ZNF487	-1.42	1.12E-03	0.0081
FAM13A-AS1	-1.42	1.98E-02	0.0458
TIRAP	-1.42	7.18E-03	0.0231
EPC1	-1.42	3.91E-04	0.0050
NKAPP1	-1.42	4.97E-03	0.0184
SMURF2	-1.42	3.99E-04	0.0051
ANO8	-1.42	1.29E-03	0.0087
LINC01088	-1.42	8.57E-03	0.0259
CDC37L1-DT	-1.42	1.66E-02	0.0403
GCLC	-1.43	1.05E-02	0.0296
DNMBP-AS1	-1.43	1.00E-02	0.0286
GLG1	-1.43	9.45E-04	0.0076
ARHGEF18	-1.43	7.05E-03	0.0229
TRAJ52	-1.43	1.69E-02	0.0408
PLLP	-1.43	1.85E-03	0.0105
ALOX12-AS1	-1.43	2.16E-03	0.0114
HERC2P9	-1.43	2.14E-03	0.0114
TRBJ2-3	-1.43	1.75E-02	0.0419
AC109927.2	-1.43	3.76E-03	0.0156
TRAJ7	-1.43	1.06E-02	0.0298
AC244207.2	-1.43	2.20E-02	0.0494
RNF125	-1.43	1.34E-02	0.0347
AC025539.1	-1.43	9.17E-04	0.0074
AC073896.2	-1.43	1.04E-02	0.0293
AC027097.2	-1.43	9.61E-03	0.0278
AC009318.1	-1.43	3.45E-03	0.0148
PPFIA1	-1.43	5.51E-03	0.0196
AP001099.1	-1.43	6.04E-03	0.0208
TRAJ21	-1.43	1.83E-03	0.0104
KLF12	-1.43	1.06E-03	0.0079
KCNJ5	-1.43	1.10E-02	0.0305
TFPI	-1.43	4.83E-04	0.0055
HELB	-1.43	4.65E-04	0.0054
AC087276.1	-1.44	1.87E-02	0.0439
ZNF805	-1.44	1.51E-03	0.0094
SHISA5	-1.44	6.01E-03	0.0207
USP25	-1.44	1.07E-03	0.0080
ENST000003134313	-1.44	1.13E-02	0.0310
PWAR6	-1.44	5.43E-04	0.0058
ENST000002621330	-1.44	1.03E-03	0.0078
GIMAP8	-1.44	1.25E-02	0.0331
CCDC30	-1.44	8.49E-04	0.0071
PLPP6	-1.44	2.35E-03	0.0121
AC135050.3	-1.44	6.85E-03	0.0225
C14orf28	-1.44	5.93E-03	0.0205
FRAT2	-1.44	4.22E-03	0.0167
SIPA1L3	-1.44	2.42E-03	0.0123
LINC02473	-1.44	5.40E-03	0.0194

ZNF226	-1.44	6.77E-04	0.0064
REXO1L1P	-1.44	2.98E-03	0.0137
FCHSD2	-1.44	1.03E-03	0.0078
AC112484.1	-1.44	1.37E-02	0.0353
ENST000003369760	-1.44	3.75E-04	0.0049
LINC02551	-1.44	1.20E-02	0.0322
AC007637.1	-1.44	1.06E-02	0.0297
DDX50P1	-1.44	9.18E-03	0.0270
LINC02444	-1.44	2.30E-03	0.0119
AL450992.1	-1.44	4.42E-03	0.0172
C10orf143	-1.44	9.67E-03	0.0279
AL513412.1	-1.45	1.08E-02	0.0302
AP002884.1	-1.45	4.57E-03	0.0176
DYRK1B	-1.45	1.08E-02	0.0301
AL157831.2	-1.45	5.16E-03	0.0188
PLSCR3	-1.45	2.21E-03	0.0116
CCDC122	-1.45	3.60E-03	0.0152
SERPINI1	-1.45	1.64E-03	0.0099
ZNF549	-1.45	3.73E-04	0.0049
NEGR1	-1.45	9.62E-04	0.0076
UGDH-AS1	-1.45	3.43E-04	0.0048
IGIP	-1.45	5.25E-04	0.0057
AC003991.1	-1.45	2.35E-03	0.0121
AL513548.1	-1.45	2.01E-03	0.0110
LLGL2	-1.45	4.48E-03	0.0173
ENST00000527515	-1.45	2.09E-02	0.0476
PIK3R1	-1.45	7.88E-04	0.0069
PDK2	-1.45	2.83E-03	0.0133
L3MBTL4-AS1	-1.45	6.27E-03	0.0212
AC048382.5	-1.45	2.53E-03	0.0125
TRAJ12	-1.45	1.63E-03	0.0099
NPHP3	-1.45	1.08E-03	0.0080
PPIAP41	-1.45	1.24E-03	0.0085
ENST000002861860	-1.45	5.58E-04	0.0059
CREBBP	-1.45	4.65E-04	0.0054
HGSNAT	-1.45	1.11E-02	0.0306
MED13L	-1.45	8.72E-04	0.0072
FRK	-1.46	5.29E-04	0.0057
AC007040.2	-1.46	4.59E-03	0.0176
RETREG1	-1.46	2.18E-03	0.0115
ENST000003392821	-1.46	9.60E-04	0.0076
PACS1	-1.46	4.48E-03	0.0173
DCDC1	-1.46	1.77E-02	0.0423
CDC20B	-1.46	6.53E-03	0.0218
ENST000002315243	-1.46	1.04E-03	0.0079
AL121992.3	-1.46	1.49E-02	0.0374
LINC00960	-1.46	8.90E-03	0.0265
TRG-AS1	-1.46	1.19E-03	0.0083
TRIM52	-1.46	4.12E-04	0.0051
AC004889.1	-1.46	1.24E-02	0.0329

TMEM72-AS1	-1.46	8.02E-03	0.0248
AC008728.1	-1.46	2.66E-03	0.0129
HMGA1P4	-1.47	1.63E-02	0.0399
AC009961.2	-1.47	2.21E-02	0.0496
LINC02260	-1.47	2.44E-03	0.0123
ARHGAP45	-1.47	4.06E-03	0.0163
LRP10	-1.47	1.31E-03	0.0087
GABRG3	-1.47	1.97E-02	0.0456
TMEM187	-1.47	3.66E-03	0.0153
TTC13	-1.47	1.28E-03	0.0086
DAP	-1.47	6.62E-03	0.0220
CASP8	-1.47	8.50E-04	0.0071
AC005261.3	-1.47	5.51E-03	0.0196
AC015819.1	-1.47	6.54E-03	0.0218
ACCS	-1.47	6.15E-03	0.0210
LGALS9	-1.47	1.15E-02	0.0313
AC009163.3	-1.47	9.71E-03	0.0280
ENST000003219451	-1.47	2.16E-03	0.0114
SLC13A4	-1.47	1.51E-02	0.0378
ZBTB11-AS1	-1.47	2.58E-03	0.0127
ENST000002603235	-1.47	1.09E-02	0.0303
UNC80	-1.47	3.45E-03	0.0148
ENST000003181302	-1.47	2.92E-03	0.0135
TMEM225B	-1.47	7.55E-03	0.0238
VNN2	-1.47	1.25E-02	0.0332
ITIH5	-1.47	4.82E-03	0.0181
ARL15	-1.47	9.60E-04	0.0076
FBXO3	-1.48	6.79E-04	0.0064
ENST000002950950	-1.48	9.18E-04	0.0074
AC027698.1	-1.48	1.83E-02	0.0433
RABGAP1L-IT1	-1.48	5.40E-03	0.0194
AC009283.1	-1.48	3.93E-03	0.0160
AC006978.2	-1.48	3.52E-03	0.0150
ATP8B2	-1.48	4.55E-04	0.0054
AC078906.1	-1.48	8.14E-03	0.0251
LEF1	-1.48	9.12E-03	0.0269
AC092329.4	-1.48	2.94E-03	0.0135
AC091614.1	-1.48	2.03E-02	0.0466
RPS3AP38	-1.48	1.20E-02	0.0323
AP001062.1	-1.48	2.21E-03	0.0116
GBP5	-1.48	6.46E-03	0.0217
PPIAP72	-1.48	2.22E-02	0.0497
ARHGAP25	-1.48	1.22E-02	0.0327
MACROD2	-1.48	1.60E-02	0.0394
LIX1	-1.48	2.85E-03	0.0134
AC106865.1	-1.48	1.56E-03	0.0096
AC104046.1	-1.48	1.67E-02	0.0406
RICTOR	-1.48	7.25E-04	0.0066
CAPN2	-1.48	2.08E-03	0.0112
RF00017.196	-1.48	5.37E-03	0.0193

CATSPERE	-1.49	1.21E-02	0.0324
ZNF222	-1.49	1.60E-03	0.0098
AL354943.1	-1.49	1.23E-02	0.0328
SOCS5P4	-1.49	5.39E-03	0.0193
SCHIP1	-1.49	4.70E-03	0.0178
CATSPERG	-1.49	1.86E-03	0.0105
KATNAL2	-1.49	2.47E-03	0.0124
LPAR5	-1.49	3.52E-03	0.0150
AL360013.2	-1.49	5.39E-03	0.0193
SUCLG2-AS1	-1.49	2.95E-03	0.0136
GPR89B	-1.49	1.54E-03	0.0095
ACAP2-IT1	-1.49	2.06E-02	0.0472
CTSS	-1.49	3.22E-04	0.0047
AC016727.1	-1.49	2.47E-03	0.0124
AC007743.1	-1.49	9.90E-04	0.0077
TMEM80	-1.49	7.95E-04	0.0069
ENST000003356700	-1.50	2.87E-03	0.0134
BCDIN3D	-1.50	1.61E-03	0.0098
ABI3BP	-1.50	8.95E-03	0.0266
PCMTD1	-1.50	1.68E-03	0.0100
LINC01697	-1.50	4.86E-03	0.0181
ENST00000641104	-1.50	8.04E-03	0.0248
TNRC6C	-1.50	5.42E-04	0.0058
AC104452.1	-1.50	2.20E-02	0.0493
AL669831.3	-1.50	1.69E-02	0.0408
CD163	-1.50	4.59E-03	0.0176
TAS2R5	-1.50	7.90E-03	0.0245
OMA1	-1.50	4.94E-04	0.0056
ANKRD23	-1.50	1.90E-03	0.0106
BLZF2P	-1.50	8.64E-03	0.0260
TMEM169	-1.50	5.35E-03	0.0192
SWT1	-1.50	1.14E-03	0.0082
AC020907.3	-1.50	4.85E-03	0.0181
C7orf43	-1.50	2.52E-03	0.0125
AC008115.3	-1.50	2.23E-02	0.0499
AC114490.3	-1.50	1.60E-03	0.0098
ASAH1	-1.51	2.87E-04	0.0045
ITGAL	-1.51	4.74E-03	0.0179
B3GAT1	-1.51	4.85E-03	0.0181
AC011374.2	-1.51	1.87E-02	0.0439
PROM2	-1.51	2.01E-02	0.0462
NKAIN3-IT1	-1.51	1.12E-03	0.0081
LINC01550	-1.51	9.00E-04	0.0074
CNNM2	-1.51	5.69E-04	0.0059
LINC00276	-1.51	6.29E-04	0.0062
CCM2	-1.51	1.74E-03	0.0102
AC010904.2	-1.51	1.71E-02	0.0411
MIR663B	-1.51	2.07E-02	0.0472
TRAV14DV4	-1.51	8.67E-03	0.0261
FAM41C	-1.51	1.32E-02	0.0343

CYYR1-AS1	-1.51	4.73E-03	0.0179
LINC02108	-1.51	1.78E-03	0.0103
SLC4A8	-1.51	6.18E-03	0.0210
TNPO1P1	-1.51	4.55E-03	0.0175
HBP1	-1.51	9.93E-04	0.0077
AC244669.1	-1.51	6.95E-03	0.0227
FLJ40194	-1.51	1.39E-02	0.0356
IYD	-1.51	1.47E-03	0.0093
FAM133DP	-1.51	1.09E-02	0.0303
C6orf163	-1.52	2.90E-03	0.0135
ZNF345	-1.52	1.26E-03	0.0086
TRAJ25	-1.52	7.38E-03	0.0235
AL662844.4	-1.52	6.62E-03	0.0220
EEF1A1P3	-1.52	1.11E-03	0.0081
GIMAP5	-1.52	1.28E-02	0.0336
PCMTD2	-1.52	9.94E-04	0.0077
USP11	-1.52	1.23E-03	0.0085
SNORD89	-1.52	2.03E-02	0.0466
AC007686.3	-1.52	1.25E-02	0.0331
MBD5	-1.52	7.18E-04	0.0065
FBXL20	-1.52	1.35E-03	0.0089
MPPE1	-1.52	9.48E-03	0.0275
SGTB	-1.52	5.88E-04	0.0060
AL136985.3	-1.52	1.19E-03	0.0083
FLG-AS1	-1.52	4.48E-03	0.0173
AC108751.4	-1.52	1.74E-02	0.0418
GABBR1	-1.52	1.37E-02	0.0353
NBPF1	-1.52	3.36E-03	0.0146
ENST000003234921	-1.53	1.03E-02	0.0291
ATXN7L1	-1.53	1.09E-03	0.0080
SLC35C1	-1.53	5.08E-03	0.0187
CREB5	-1.53	6.77E-03	0.0223
BEND6	-1.53	3.20E-03	0.0142
LINC00862	-1.53	1.52E-02	0.0379
ENST000003190801	-1.53	3.22E-03	0.0142
CHURC1	-1.53	2.24E-04	0.0041
AC008467.1	-1.53	7.03E-04	0.0065
AL354919.1	-1.53	1.26E-02	0.0334
COL1A2	-1.53	1.14E-02	0.0312
LMOD3	-1.53	1.76E-02	0.0421
OR2F1	-1.53	1.16E-02	0.0316
ZNF767P	-1.53	1.76E-03	0.0102
ZNF792	-1.54	2.78E-03	0.0132
MIR122HG	-1.54	1.85E-03	0.0105
SF3B1	-1.54	6.87E-04	0.0064
AL109659.1	-1.54	1.34E-02	0.0347
IQCN	-1.54	1.94E-02	0.0451
AC105233.5	-1.54	8.28E-03	0.0253
TRBV12-3	-1.54	3.88E-03	0.0159
AC211486.3	-1.54	2.66E-03	0.0129

AC092119.2	-1.54	1.98E-03	0.0109
HGD	-1.54	1.03E-02	0.0291
HFE	-1.54	3.37E-03	0.0146
KLHL22	-1.54	2.82E-03	0.0133
LINC00891	-1.54	1.03E-02	0.0291
HSPA1L	-1.54	1.21E-03	0.0084
CRISPLD2	-1.54	2.48E-03	0.0124
TRMT2B-AS1	-1.54	1.08E-02	0.0301
PLCL2	-1.54	1.60E-03	0.0098
ZNF44	-1.54	3.49E-04	0.0048
PATJ	-1.54	5.39E-04	0.0058
TAC4	-1.54	9.05E-03	0.0267
MARCH6	-1.54	8.78E-03	0.0263
AC007663.4	-1.54	1.84E-02	0.0435
SMYD3-IT1	-1.54	1.49E-02	0.0374
MPV17L	-1.54	3.30E-03	0.0144
ENST000003247652	-1.55	9.18E-03	0.0270
BX248409.1	-1.55	1.02E-02	0.0289
JMY	-1.55	2.61E-03	0.0127
AC007342.5	-1.55	1.76E-02	0.0421
LINC01224	-1.55	7.11E-03	0.0230
TSGA10	-1.55	1.44E-03	0.0092
CASR	-1.55	5.62E-03	0.0198
RF00017.2	-1.55	1.94E-02	0.0451
AP000542.3	-1.55	1.30E-03	0.0087
NMRK1	-1.55	2.12E-03	0.0113
ZSCAN16	-1.55	1.04E-03	0.0079
PRMT2	-1.55	1.26E-03	0.0086
DNAJB9	-1.55	6.13E-04	0.0061
SENP7	-1.55	6.94E-04	0.0065
ENST000003076581	-1.55	2.28E-03	0.0118
ENST000003152491	-1.55	3.93E-03	0.0160
PLCB2	-1.56	6.21E-04	0.0062
LRRC37A5P	-1.56	7.42E-03	0.0236
SLC44A2	-1.56	3.48E-03	0.0149
AC005072.1	-1.56	1.29E-02	0.0339
TRAJ8	-1.56	1.43E-02	0.0363
LRRC37B	-1.56	1.32E-03	0.0088
CACNA2D4	-1.56	4.31E-03	0.0169
TAS2R63P	-1.56	1.11E-02	0.0307
LINC01881	-1.56	1.05E-02	0.0295
CFAP70	-1.56	3.97E-03	0.0161
KLF7-IT1	-1.56	1.30E-03	0.0087
AC068870.1	-1.56	1.29E-02	0.0339
TADA2B	-1.56	5.67E-04	0.0059
RTCA-AS1	-1.56	6.31E-03	0.0213
AC138749.6	-1.56	1.73E-03	0.0101
TTC14	-1.56	5.47E-04	0.0058
AC017076.1	-1.56	5.02E-03	0.0185
ENST000002628940	-1.56	1.71E-03	0.0101

KCNJ3	-1.56	1.27E-02	0.0334
FAM71F2	-1.56	1.85E-02	0.0436
DHX58	-1.56	8.05E-03	0.0249
RF00017.88	-1.56	6.16E-03	0.0210
UBL3	-1.56	2.08E-04	0.0040
AC005232.1	-1.56	1.06E-02	0.0296
PTOV1-AS1	-1.56	6.99E-03	0.0228
AC139494.2	-1.56	8.00E-03	0.0247
PHC3	-1.56	2.99E-04	0.0046
FRG1DP	-1.56	3.17E-03	0.0141
C9orf72	-1.56	1.34E-03	0.0088
AC232271.1	-1.57	2.26E-03	0.0118
AC099489.1	-1.57	3.19E-03	0.0141
SPIN2A	-1.57	5.70E-03	0.0200
KLF8	-1.57	5.07E-03	0.0186
AL049830.3	-1.57	2.43E-03	0.0123
AC013474.1	-1.57	1.20E-02	0.0323
AC025171.1	-1.57	2.78E-03	0.0132
SLC2A5	-1.57	1.60E-02	0.0394
AL354718.1	-1.57	1.29E-02	0.0339
AC006511.5	-1.57	1.88E-02	0.0442
AL023755.1	-1.57	5.64E-03	0.0199
TBC1D2	-1.57	1.04E-02	0.0294
CCDC18-AS1	-1.57	8.38E-04	0.0071
ENST000003567980	-1.57	2.19E-03	0.0115
AC093726.1	-1.57	4.72E-03	0.0178
GRAP2	-1.57	3.52E-03	0.0150
LYG1	-1.57	7.26E-03	0.0232
AC010186.3	-1.57	2.08E-03	0.0112
AC241520.1	-1.58	6.05E-03	0.0208
AC044802.1	-1.58	8.53E-03	0.0258
NLRP12	-1.58	1.42E-02	0.0362
MPP7	-1.58	1.54E-03	0.0095
CASP1	-1.58	9.37E-04	0.0075
AC087239.1	-1.58	3.80E-03	0.0157
AC007342.1	-1.58	9.86E-03	0.0283
SYNJ2BP	-1.58	2.44E-04	0.0042
L3HYPDH	-1.58	1.59E-03	0.0097
DNASE1	-1.58	3.60E-03	0.0152
AC112503.2	-1.58	1.41E-02	0.0361
CBX4	-1.58	1.64E-03	0.0099
MAP2K6	-1.58	5.32E-03	0.0192
AC138150.1	-1.58	7.32E-03	0.0234
SLC22A2	-1.58	1.43E-02	0.0365
SCAF4	-1.58	3.96E-03	0.0161
ENST000003432101	-1.59	1.12E-03	0.0081
KLHL3	-1.59	1.07E-03	0.0080
AL031430.1	-1.59	1.07E-03	0.0080
EXOC6B	-1.59	9.96E-03	0.0285
AL604028.2	-1.59	1.46E-03	0.0093

NEB	-1.59	3.21E-03	0.0142
LINC01089	-1.59	6.41E-04	0.0062
LINC00221	-1.59	5.47E-03	0.0195
ZNF658	-1.59	4.43E-04	0.0053
MORC3	-1.59	2.34E-04	0.0042
KIAA0355	-1.59	5.41E-04	0.0058
OXNAD1	-1.60	5.35E-04	0.0058
AL606760.2	-1.60	9.95E-03	0.0285
AC012103.1	-1.60	1.80E-02	0.0428
AC053513.1	-1.60	1.66E-03	0.0099
AC099063.4	-1.60	1.96E-03	0.0108
AL138479.1	-1.60	5.23E-03	0.0190
UTRN	-1.60	5.60E-04	0.0059
AKAP7	-1.60	4.26E-03	0.0168
ENST000002678071	-1.60	5.67E-04	0.0059
CCDC112	-1.60	1.42E-03	0.0091
AL160191.1	-1.60	1.97E-02	0.0456
OR14J1	-1.60	7.77E-04	0.0068
SSBP2	-1.60	2.62E-04	0.0044
APPL2	-1.60	6.13E-04	0.0061
AC010680.5	-1.60	2.34E-03	0.0120
FBLIM1	-1.60	1.55E-02	0.0385
ENST000002620962	-1.60	1.03E-03	0.0078
MANEA-DT	-1.60	3.50E-03	0.0149
MAL	-1.60	6.60E-03	0.0220
ENST000003280890	-1.60	1.78E-02	0.0423
ENST000003442560	-1.60	1.04E-03	0.0079
ENST000003403600	-1.60	2.01E-02	0.0463
AL592183.1	-1.60	1.58E-03	0.0097
PIK3CG	-1.60	1.46E-02	0.0368
SGSH	-1.61	6.03E-03	0.0207
OR2M4	-1.61	4.60E-03	0.0176
AC092017.4	-1.61	1.19E-03	0.0083
TRAJ55	-1.61	1.54E-02	0.0383
AC079610.1	-1.61	1.54E-03	0.0095
ENST000003088240	-1.61	6.90E-04	0.0064
ZFAND4	-1.61	5.63E-04	0.0059
ENST000003094460	-1.61	3.18E-03	0.0141
AL589740.1	-1.61	1.52E-02	0.0379
AP000692.1	-1.61	1.76E-03	0.0102
RN7SL834P	-1.61	8.54E-03	0.0258
KRT18P31	-1.61	4.18E-03	0.0166
PRKXP1	-1.61	8.04E-03	0.0248
AL627230.2	-1.61	6.62E-03	0.0220
TTC9	-1.61	7.42E-04	0.0067
AC068790.2	-1.61	6.38E-03	0.0215
GIMAP6	-1.61	1.69E-03	0.0101
SPINK9	-1.61	4.64E-03	0.0177
TMEM108	-1.61	1.73E-02	0.0415
AC027455.2	-1.62	5.75E-03	0.0201

AC017035.1	-1.62	6.62E-03	0.0220
AC005154.2	-1.62	2.81E-03	0.0133
ENST000002915981	-1.62	9.35E-04	0.0075
AC116913.1	-1.62	1.68E-03	0.0100
HMGA2	-1.62	7.39E-03	0.0235
OR51E2	-1.62	1.46E-02	0.0368
APBB1IP	-1.62	9.54E-04	0.0076
ZNF571	-1.62	3.17E-03	0.0141
AC007842.1	-1.62	1.29E-02	0.0338
AC007842.2	-1.62	1.29E-02	0.0338
SFN	-1.62	1.29E-02	0.0338
TAS2R50	-1.62	1.14E-02	0.0313
AC005332.4	-1.62	6.48E-03	0.0217
TIAF1	-1.62	8.60E-03	0.0259
MAPK8IP2	-1.62	1.42E-02	0.0362
FAM186A	-1.62	1.37E-02	0.0353
ENST000002488990	-1.62	1.79E-02	0.0425
CELF4	-1.62	1.49E-02	0.0373
SLFN12L	-1.62	2.61E-03	0.0127
ZFP36	-1.62	7.13E-03	0.0230
AC009120.1	-1.62	1.72E-03	0.0101
KIAA0825	-1.62	1.50E-03	0.0094
PBX4	-1.62	3.46E-03	0.0148
AP000688.2	-1.62	1.86E-02	0.0439
TRBV29-1	-1.62	2.10E-03	0.0113
C2orf91	-1.62	1.25E-03	0.0086
SYNRG	-1.63	6.03E-04	0.0061
RN7SL600P	-1.63	1.78E-02	0.0424
SNTN	-1.63	3.17E-03	0.0141
GGT6	-1.63	3.92E-03	0.0160
TSPAN19	-1.63	1.47E-03	0.0093
LINC00863	-1.63	1.08E-03	0.0080
SCARNA10	-1.63	1.99E-04	0.0040
CPQ	-1.63	1.31E-02	0.0342
DRAXIN	-1.63	3.36E-03	0.0146
ZNF75D	-1.63	5.39E-04	0.0058
AC016405.1	-1.63	9.87E-04	0.0077
OCLN	-1.63	6.79E-04	0.0064
PLGLB1	-1.63	6.06E-03	0.0208
SLC2A4RG	-1.63	9.98E-03	0.0285
FAM21EP	-1.63	4.53E-03	0.0174
RN7SL577P	-1.63	4.43E-03	0.0172
AVIL	-1.63	1.38E-03	0.0090
ZNF30	-1.63	4.59E-04	0.0054
ITPKB	-1.63	3.03E-03	0.0138
AC008937.3	-1.63	2.20E-02	0.0495
AC005912.1	-1.64	1.34E-02	0.0348
FBXL17	-1.64	7.03E-03	0.0228
AC114956.3	-1.64	3.58E-03	0.0151
MAP3K5	-1.64	4.76E-04	0.0055

OMG	-1.64	9.11E-03	0.0269
AC022706.1	-1.64	6.52E-03	0.0218
FCER1G	-1.64	1.65E-02	0.0402
GGNBP1	-1.64	2.13E-03	0.0114
TRAJ44	-1.64	1.28E-02	0.0338
AL669831.4	-1.64	5.02E-03	0.0185
LRRTM4	-1.64	5.92E-03	0.0205
AC021660.3	-1.64	3.43E-03	0.0147
UBASH3A	-1.64	4.63E-03	0.0177
AC078795.3	-1.64	1.41E-02	0.0361
ENST000002643352	-1.65	2.14E-02	0.0484
IQSEC1	-1.65	5.77E-04	0.0060
TAS2R4	-1.65	3.49E-03	0.0149
ZNF862	-1.65	1.47E-03	0.0093
RN7SL775P	-1.65	1.97E-02	0.0456
UCKL1-AS1	-1.65	1.11E-02	0.0307
RNU6-82P	-1.65	6.48E-03	0.0217
MIAT	-1.65	2.06E-03	0.0111
AC026992.2	-1.65	9.05E-03	0.0267
EFHD1	-1.65	1.39E-02	0.0356
AC007383.2	-1.65	2.49E-03	0.0124
AC007383.4	-1.65	2.49E-03	0.0124
AL034546.1	-1.65	1.60E-02	0.0394
VAMP2	-1.65	8.39E-04	0.0071
AC136352.10	-1.66	1.66E-03	0.0100
AL627402.1	-1.66	1.01E-02	0.0288
ANKRD44-IT1	-1.66	6.88E-03	0.0226
NOSTRIN	-1.66	1.07E-02	0.0300
LRRC2	-1.66	7.64E-03	0.0240
ERMN	-1.66	1.65E-03	0.0099
FAM19A2	-1.66	1.38E-03	0.0090
PAX8-AS1	-1.66	7.49E-03	0.0237
KCNMB1	-1.66	1.14E-02	0.0313
LINC01091	-1.66	1.12E-02	0.0309
TCF7	-1.67	2.67E-03	0.0129
ENST00000550837	-1.67	6.37E-04	0.0062
ALKBH7	-1.67	1.03E-03	0.0078
SLC35E2A	-1.67	2.33E-03	0.0120
GPM6A	-1.67	1.98E-03	0.0109
INMT	-1.67	2.19E-03	0.0115
AC093157.2	-1.67	1.28E-02	0.0338
AL049697.1	-1.67	1.76E-03	0.0102
AC007529.1	-1.67	3.27E-03	0.0143
TRBV14	-1.67	2.80E-03	0.0133
NBPF15	-1.67	7.79E-04	0.0068
ENST000002026771	-1.67	3.29E-04	0.0047
FAM157B	-1.67	1.05E-02	0.0294
PART1	-1.68	9.29E-03	0.0272
PDP1	-1.68	6.80E-04	0.0064
NATD1	-1.68	6.07E-03	0.0208

RN7SL648P	-1.68	2.00E-03	0.0109
SEMA4C	-1.68	3.13E-03	0.0140
AL645933.2	-1.68	1.85E-02	0.0436
AC087664.2	-1.68	6.00E-03	0.0207
HSFX1	-1.68	1.98E-02	0.0458
AC013448.1	-1.68	7.86E-03	0.0245
ENST000002860630	-1.68	1.42E-02	0.0362
CRBN	-1.68	2.12E-04	0.0041
PATE4	-1.69	1.49E-02	0.0373
SYNPO2L	-1.69	1.49E-02	0.0373
EMB	-1.69	4.07E-04	0.0051
DIP2A	-1.69	1.78E-03	0.0103
AC090518.1	-1.69	1.65E-02	0.0402
AC006518.4	-1.69	6.81E-03	0.0224
LRRC37A4P	-1.69	4.48E-04	0.0053
TIPARP-AS1	-1.69	2.88E-03	0.0134
FYCO1	-1.69	2.89E-04	0.0045
GIMAP2	-1.69	8.26E-04	0.0071
TIMP1	-1.69	2.36E-03	0.0121
AC102953.2	-1.69	5.98E-03	0.0206
LINC02365	-1.69	9.05E-03	0.0267
TRAJ1	-1.69	8.95E-03	0.0266
AC009090.1	-1.69	2.99E-03	0.0137
ENST000003136241	-1.69	4.48E-03	0.0173
LMTK3	-1.69	8.72E-03	0.0262
YWHABP2	-1.69	1.41E-02	0.0361
AL355816.2	-1.69	3.96E-03	0.0160
C21orf62-AS1	-1.69	1.61E-03	0.0098
DGKA	-1.69	1.79E-03	0.0103
RPS3P2	-1.70	6.69E-03	0.0222
PGGHG	-1.70	2.68E-04	0.0044
C9orf131	-1.70	6.46E-03	0.0217
AC006042.3	-1.70	1.29E-02	0.0339
MAN2B2	-1.70	1.95E-04	0.0039
ANO9	-1.70	6.71E-04	0.0064
CCDC69	-1.70	1.90E-04	0.0039
AL157392.3	-1.70	1.13E-03	0.0081
CHST12	-1.70	2.96E-04	0.0046
LINC00336	-1.70	8.82E-03	0.0263
AC111182.1	-1.70	2.94E-03	0.0136
AL589993.1	-1.70	5.94E-04	0.0060
CDKN2A	-1.70	7.76E-03	0.0243
ENST000002946640	-1.70	2.21E-02	0.0495
GAL3ST4	-1.70	5.23E-03	0.0190
TSC22D1-AS1	-1.70	1.55E-02	0.0384
FTX	-1.71	2.71E-04	0.0044
SBK1	-1.71	5.15E-03	0.0188
AC122718.2	-1.71	5.45E-03	0.0195
OR2A7	-1.71	2.97E-03	0.0136
AC104966.1	-1.71	1.73E-02	0.0414

ZBTB20-AS4	-1.71	2.19E-02	0.0493
SCARNA5	-1.71	6.64E-04	0.0063
TRAV8-3	-1.71	1.07E-03	0.0079
BID	-1.71	4.74E-04	0.0055
FABP2	-1.71	1.07E-03	0.0080
AL133245.1	-1.71	2.67E-03	0.0129
NPM1P37	-1.71	1.09E-03	0.0080
STK10	-1.71	2.80E-04	0.0045
PTPN12	-1.72	9.80E-04	0.0077
AC100832.2	-1.72	1.62E-02	0.0397
SPAG5-AS1	-1.72	1.13E-03	0.0081
BTN3A2	-1.72	5.77E-04	0.0060
C20orf144	-1.72	1.21E-02	0.0324
CBR3-AS1	-1.72	5.86E-04	0.0060
SCN2A	-1.72	6.64E-04	0.0063
HNRNPH1P1	-1.72	1.89E-02	0.0442
RF00017.166	-1.72	4.73E-03	0.0179
ENST000002742033	-1.72	1.81E-04	0.0039
AC090198.1	-1.72	6.25E-04	0.0062
CASK	-1.73	1.02E-03	0.0078
PFN1P2	-1.73	1.32E-02	0.0343
STRA6LP	-1.73	1.40E-03	0.0091
JMJD7-PLA2G4B	-1.73	5.91E-04	0.0060
KIAA1324L	-1.73	1.52E-02	0.0379
PTGDS	-1.73	1.06E-02	0.0297
AC096921.2	-1.73	2.94E-03	0.0136
OR7E122P	-1.73	6.93E-04	0.0065
ENST000003180231	-1.73	3.99E-03	0.0161
HSD17B11	-1.73	1.64E-03	0.0099
PCAT1	-1.73	6.80E-04	0.0064
AL359962.2	-1.74	1.53E-03	0.0095
ENST000002814710	-1.74	2.60E-03	0.0127
CASP17P	-1.74	3.16E-03	0.0141
AL356259.1	-1.74	1.65E-02	0.0401
SLC39A12-AS1	-1.74	2.54E-03	0.0126
ERN1	-1.74	3.20E-03	0.0142
SMAD9	-1.74	2.56E-03	0.0126
ENST000003677710	-1.74	3.70E-04	0.0049
TMEM140	-1.74	1.64E-03	0.0099
FAM229A	-1.74	3.45E-03	0.0148
ZNF211	-1.74	3.44E-03	0.0148
RNPEPL1	-1.74	2.61E-03	0.0127
ALS2CR12	-1.74	3.52E-03	0.0150
POT1-AS1	-1.74	7.46E-04	0.0067
PCYOX1L	-1.74	2.18E-04	0.0041
DENND4C	-1.74	2.31E-04	0.0042
DBP	-1.74	4.69E-04	0.0054
FSIP2	-1.75	3.51E-04	0.0048
AC004890.2	-1.75	7.08E-04	0.0065
LRRC37A3	-1.75	1.09E-02	0.0303

CASS4	-1.75	1.88E-02	0.0442
OBSCN	-1.75	8.08E-04	0.0070
AL360091.1	-1.75	1.28E-02	0.0337
AC095057.3	-1.75	2.77E-03	0.0132
TGIF2P1	-1.75	2.11E-02	0.0480
PRDM11	-1.75	2.14E-03	0.0114
ENST000003414550	-1.75	1.95E-03	0.0107
AC016705.2	-1.75	5.46E-03	0.0195
AC011477.1	-1.75	2.73E-03	0.0131
FAM66E	-1.75	1.56E-02	0.0385
AC089987.1	-1.76	1.50E-02	0.0376
SLC25A21	-1.76	1.99E-02	0.0460
ARHGAP42P4	-1.76	6.71E-03	0.0222
SIDT1	-1.76	7.20E-04	0.0065
RSU1P2	-1.76	1.32E-02	0.0343
ZFP3	-1.76	2.89E-03	0.0134
AC007038.1	-1.76	1.22E-03	0.0084
SLC29A3	-1.76	1.05E-02	0.0296
AL606834.2	-1.76	1.92E-03	0.0107
TMIGD2	-1.76	2.26E-04	0.0041
PIK3R5	-1.76	1.57E-02	0.0387
TRIM73	-1.76	1.04E-03	0.0078
WBP2NL	-1.76	1.63E-03	0.0098
AL357054.1	-1.76	1.69E-02	0.0408
SYCE1L	-1.76	1.57E-02	0.0388
PGM5	-1.76	2.85E-03	0.0134
TOMM20P2	-1.76	6.69E-03	0.0222
CARF	-1.76	1.77E-03	0.0102
IGF1R	-1.76	3.79E-03	0.0156
TSC22D1	-1.76	5.36E-04	0.0058
AC010900.1	-1.76	1.13E-02	0.0310
RRS1-AS1	-1.76	8.80E-04	0.0073
ABHD12B	-1.76	9.99E-03	0.0285
FBLN5	-1.77	9.06E-03	0.0268
MIR22HG	-1.77	3.16E-03	0.0141
TRHDE-AS1	-1.77	1.73E-03	0.0101
AC022929.2	-1.77	2.38E-04	0.0042
AL136981.2	-1.77	2.79E-03	0.0133
ENST000002327442	-1.77	8.18E-03	0.0251
AP003486.1	-1.77	3.23E-04	0.0047
MCOLN3	-1.77	1.40E-02	0.0358
CYSLTR1	-1.77	9.31E-03	0.0272
IL6R-AS1	-1.77	1.40E-02	0.0359
SETD1B	-1.77	1.60E-03	0.0098
Z98048.1	-1.78	1.99E-02	0.0460
RF00017.200	-1.78	1.94E-02	0.0451
ENST000003136631	-1.78	1.37E-02	0.0353
SOX5	-1.78	7.50E-03	0.0237
MIR5194	-1.78	1.19E-02	0.0321
AC108010.1	-1.78	1.45E-03	0.0092

ENST000003209964	-1.78	3.22E-03	0.0142
AC090241.3	-1.78	1.75E-03	0.0102
IKBKE	-1.78	8.19E-04	0.0070
EFCAB12	-1.78	9.04E-03	0.0267
ENST000002825162	-1.78	3.92E-04	0.0050
DCN	-1.78	1.43E-02	0.0364
AC005775.1	-1.78	1.42E-02	0.0362
AP000766.1	-1.78	3.72E-03	0.0155
AC113420.1	-1.78	2.09E-02	0.0476
ZNF425	-1.79	2.21E-03	0.0116
DOP1A	-1.79	2.20E-04	0.0041
FLT3LG	-1.79	1.86E-03	0.0105
TRBV19	-1.79	3.19E-04	0.0047
TECPR1	-1.79	4.95E-04	0.0056
NONOP2	-1.79	4.48E-03	0.0173
TRAJ3	-1.79	8.45E-04	0.0071
NBPF19	-1.79	6.41E-04	0.0062
ZNF350-AS1	-1.79	9.75E-03	0.0281
IL12A-AS1	-1.80	1.15E-02	0.0313
TNFRSF10A	-1.80	2.68E-04	0.0044
RNU4-24P	-1.80	1.44E-02	0.0366
ENST000003485200	-1.80	4.85E-04	0.0055
MYO3B	-1.80	7.08E-03	0.0229
C1orf54	-1.80	1.10E-02	0.0304
ENST000003385601	-1.80	2.60E-04	0.0044
SMAD3	-1.80	1.68E-03	0.0100
AL359962.1	-1.80	1.23E-03	0.0085
SLC35E2B	-1.80	3.80E-03	0.0157
LINC01719	-1.80	6.20E-03	0.0211
LILRB4	-1.80	1.17E-02	0.0318
ERICH6-AS1	-1.80	1.57E-03	0.0097
ENST000002971090	-1.81	1.76E-04	0.0039
AL008727.1	-1.81	2.18E-03	0.0115
PINK1	-1.81	2.76E-04	0.0044
HACD4	-1.81	8.10E-03	0.0250
AL355596.1	-1.81	3.54E-03	0.0150
KLHL6-AS1	-1.81	8.92E-04	0.0073
GPR155	-1.81	5.22E-03	0.0190
AL671710.1	-1.81	7.04E-03	0.0229
WHAMM	-1.81	1.22E-03	0.0084
LMO7	-1.81	3.89E-04	0.0050
AC040977.1	-1.81	9.34E-03	0.0273
AC090377.1	-1.81	1.57E-03	0.0096
AC007406.5	-1.81	1.29E-03	0.0087
MDGA1	-1.81	5.89E-04	0.0060
AC092747.2	-1.82	1.46E-02	0.0370
BDNF-AS	-1.82	4.30E-03	0.0169
C3orf20	-1.82	1.98E-02	0.0459
GABRP	-1.82	5.72E-03	0.0201
ZNF252P-AS1	-1.82	1.35E-02	0.0349

PITPNM2	-1.82	1.43E-02	0.0364
MITF	-1.82	1.07E-02	0.0299
AC114490.2	-1.82	2.57E-03	0.0126
STX3	-1.82	2.87E-04	0.0045
ENST000002609081	-1.82	5.00E-04	0.0056
LINC02615	-1.83	4.10E-03	0.0164
SUGT1P4-STRA6LP	-1.83	5.22E-03	0.0190
OR4D1	-1.83	1.21E-03	0.0084
AP001160.3	-1.83	8.94E-03	0.0266
MIR421	-1.83	1.82E-03	0.0104
AC136475.3	-1.83	7.20E-04	0.0065
SELENOP	-1.83	1.96E-02	0.0455
AC020779.1	-1.83	1.38E-02	0.0356
AL356020.1	-1.83	7.51E-03	0.0238
AC098679.2	-1.83	1.24E-02	0.0331
FAM8A1	-1.83	4.96E-04	0.0056
AC005332.3	-1.83	4.38E-03	0.0171
RF00554.5	-1.83	1.59E-02	0.0393
TRAJ13	-1.83	7.37E-04	0.0066
LINC00868	-1.83	4.70E-03	0.0178
AC122718.1	-1.83	9.00E-03	0.0267
AL133330.1	-1.83	7.06E-03	0.0229
MGAT4A	-1.83	5.12E-04	0.0056
POC1B-AS1	-1.84	2.66E-04	0.0044
SEC31B	-1.84	4.74E-04	0.0055
PSMD6-AS1	-1.84	2.10E-02	0.0479
ZPLD1	-1.84	2.20E-02	0.0495
ENST000002272140	-1.84	4.98E-03	0.0184
RPS4XP16	-1.84	1.95E-03	0.0107
AL391684.1	-1.84	2.73E-03	0.0131
KRBA2	-1.84	7.75E-04	0.0068
AC011726.3	-1.84	1.29E-02	0.0339
LINC01122	-1.84	1.81E-02	0.0430
ENST00000471810	-1.84	9.02E-03	0.0267
AC005670.2	-1.84	1.52E-02	0.0380
AC008072.1	-1.84	9.43E-03	0.0274
AC008760.1	-1.84	2.02E-02	0.0465
ENST000003946571	-1.84	9.97E-03	0.0285
ENST000002850130	-1.85	1.26E-03	0.0086
TENT5C	-1.85	2.17E-03	0.0115
MUC20	-1.85	1.38E-03	0.0090
ATP5MGL	-1.85	7.21E-03	0.0231
EDEM3	-1.85	1.88E-04	0.0039
RF00017.62	-1.85	1.16E-02	0.0317
LRRC37A17P	-1.85	1.88E-02	0.0441
AL365255.1	-1.85	1.29E-02	0.0339
Z83843.1	-1.85	1.70E-04	0.0038
AC079062.1	-1.85	1.49E-02	0.0374
ENST000002160370	-1.85	7.22E-03	0.0232
LINC02362	-1.85	1.57E-03	0.0097

CTSD	-1.85	2.49E-04	0.0043
ACSS1	-1.85	2.63E-04	0.0044
ZNF101	-1.86	1.40E-04	0.0036
MMP25-AS1	-1.86	2.41E-03	0.0122
CECR2	-1.86	5.40E-03	0.0194
IDO2	-1.86	1.87E-02	0.0439
LINC02325	-1.86	3.34E-04	0.0047
LINC01772	-1.86	1.37E-03	0.0089
MIR548E	-1.86	1.75E-02	0.0418
MPP4	-1.86	1.89E-02	0.0443
LYSMD3	-1.86	2.53E-03	0.0125
BMS1P22	-1.86	1.14E-02	0.0313
TRGC1	-1.86	7.83E-03	0.0244
NELL2	-1.87	1.16E-02	0.0316
GLI4	-1.87	3.93E-03	0.0160
TMEM8A	-1.87	2.46E-03	0.0124
RIPK3	-1.87	1.20E-03	0.0084
AC009803.3	-1.87	1.72E-02	0.0414
AL354793.1	-1.87	7.95E-03	0.0247
AL445531.1	-1.87	2.61E-03	0.0127
RPS2P45	-1.87	1.51E-03	0.0094
THRB-IT1	-1.87	1.90E-02	0.0444
RABL2A	-1.87	1.47E-03	0.0093
AC055717.1	-1.87	2.83E-03	0.0133
PLEKHM1P1	-1.87	2.44E-04	0.0042
MHENCR	-1.87	3.61E-03	0.0152
SPP2	-1.87	9.98E-04	0.0077
TMEM50B	-1.87	1.62E-04	0.0037
NDRG2	-1.87	9.37E-04	0.0075
AC008758.2	-1.87	1.06E-02	0.0297
EGFL6	-1.87	1.86E-02	0.0438
NHSL2	-1.87	3.47E-03	0.0148
RN7SKP36	-1.87	7.70E-03	0.0241
PHF1	-1.88	3.43E-04	0.0048
TAPT1-AS1	-1.88	5.11E-03	0.0187
VIM	-1.88	1.76E-03	0.0102
ATP8A2	-1.88	1.20E-02	0.0322
PPDPF	-1.88	4.13E-03	0.0164
ENST000003552800	-1.88	3.92E-03	0.0160
HDAC4	-1.88	1.04E-03	0.0078
SH3BP5	-1.88	1.70E-03	0.0101
SLC6A4	-1.88	5.08E-03	0.0187
ITPKB-IT1	-1.88	9.60E-03	0.0278
CD84	-1.88	9.14E-04	0.0074
AC006518.2	-1.88	3.87E-03	0.0158
AC006518.6	-1.88	3.87E-03	0.0158
AC244197.3	-1.88	9.06E-03	0.0268
ENST000002681840	-1.88	2.77E-03	0.0132
AL359752.1	-1.89	1.93E-03	0.0107
AC124312.1	-1.89	5.16E-03	0.0188

HAS3	-1.89	1.06E-02	0.0297
AC068790.3	-1.89	8.74E-03	0.0262
LINC00882	-1.89	5.89E-03	0.0204
GRAP	-1.89	1.08E-04	0.0033
AC008574.1	-1.89	7.50E-03	0.0237
AC124312.3	-1.89	8.62E-04	0.0072
COL8A1	-1.89	6.15E-03	0.0210
N4BP2L2-IT2	-1.89	1.44E-04	0.0036
LINC00672	-1.90	4.43E-04	0.0053
LZTS3	-1.90	7.17E-03	0.0231
SYTL1	-1.90	1.25E-04	0.0035
ACVR2A	-1.90	2.73E-03	0.0131
SATB1-AS1	-1.90	7.70E-04	0.0068
RFX4	-1.90	1.62E-02	0.0396
AC015911.3	-1.90	2.56E-03	0.0126
ZNF879	-1.90	3.15E-03	0.0141
SPSB3	-1.90	1.19E-03	0.0083
AC011476.3	-1.90	1.26E-02	0.0333
HTN1	-1.90	3.39E-04	0.0048
AC084782.1	-1.90	7.60E-04	0.0067
GIMAP1-GIMAP5	-1.90	2.03E-04	0.0040
ENST000003468320	-1.90	1.14E-03	0.0082
ARL4C	-1.90	9.42E-03	0.0274
FGF7P6	-1.90	5.31E-03	0.0191
CACNA1C-AS1	-1.91	8.78E-03	0.0263
AC004706.1	-1.91	2.31E-03	0.0119
MX2	-1.91	1.49E-04	0.0036
LINC00894	-1.91	5.88E-03	0.0204
AC037459.3	-1.91	2.41E-03	0.0122
NPM1P40	-1.91	6.25E-04	0.0062
SLC12A6	-1.91	1.38E-03	0.0090
AL360270.1	-1.91	5.92E-03	0.0205
HTRA4	-1.91	3.91E-04	0.0050
GIMAP4	-1.91	1.26E-04	0.0035
BST1	-1.91	3.64E-03	0.0153
AC006359.3	-1.91	3.51E-03	0.0149
BBS2	-1.92	7.33E-04	0.0066
ITM2B	-1.92	1.74E-04	0.0038
LINC00824	-1.92	6.39E-03	0.0215
ATXN8OS	-1.92	4.65E-03	0.0177
PYHIN1	-1.92	1.81E-03	0.0104
AC092683.1	-1.92	1.03E-04	0.0033
ENST000003222721	-1.92	1.19E-03	0.0083
C1QTNF9	-1.92	1.80E-02	0.0428
GUSBP11	-1.92	1.69E-03	0.0101
LINC00324	-1.92	3.29E-03	0.0144
SFRP4	-1.93	1.22E-02	0.0327
CCDC92	-1.93	7.56E-04	0.0067
AL359641.1	-1.93	2.03E-03	0.0110
GALT	-1.93	3.83E-04	0.0050

TRAJ30	-1.93	2.02E-03	0.0110
VPS37B	-1.93	3.53E-04	0.0048
DIRC2	-1.93	2.98E-04	0.0046
LINC01787	-1.93	7.88E-03	0.0245
AC134508.1	-1.93	1.98E-03	0.0109
PDE3A	-1.93	1.09E-03	0.0080
SINHCAFP3	-1.93	3.93E-04	0.0050
ITGA6-AS1	-1.93	1.99E-02	0.0460
AL117342.1	-1.93	9.25E-03	0.0271
AC005534.1	-1.93	1.02E-02	0.0289
EZH1	-1.93	1.87E-04	0.0039
CCDC102B	-1.93	1.91E-03	0.0107
AP002026.1	-1.94	4.68E-03	0.0178
ENST000003109580	-1.94	3.24E-03	0.0142
AC116351.1	-1.94	1.00E-03	0.0077
AC116351.3	-1.94	1.00E-03	0.0077
SCML2P2	-1.94	8.31E-03	0.0254
RN7SL246P	-1.94	1.33E-02	0.0346
BCL9L	-1.94	4.05E-03	0.0162
AC093157.1	-1.94	1.61E-04	0.0037
AC011287.2	-1.94	5.65E-04	0.0059
BTN3A1	-1.94	1.45E-02	0.0367
SYNPO2	-1.95	6.47E-04	0.0063
LINC01422	-1.95	4.11E-03	0.0164
AC005070.3	-1.95	1.46E-03	0.0093
MIR29B2CHG	-1.95	6.03E-04	0.0061
AC007666.1	-1.95	2.27E-03	0.0118
AC011816.2	-1.95	3.89E-03	0.0159
AC007620.2	-1.95	4.70E-04	0.0054
ACTA2-AS1	-1.95	4.01E-03	0.0162
HMGB1P3	-1.95	7.54E-03	0.0238
TRGV10	-1.95	1.66E-02	0.0404
INPP4A	-1.95	2.50E-04	0.0043
ADCY10	-1.95	7.19E-03	0.0231
LNCTAM34A	-1.95	1.47E-03	0.0093
ZNF480	-1.95	1.03E-03	0.0078
CEP68	-1.96	1.24E-03	0.0085
AC008669.1	-1.96	1.19E-03	0.0083
VN1R83P	-1.96	2.19E-03	0.0115
ENST000003410111	-1.96	1.84E-02	0.0434
GTF2IRD2	-1.96	1.86E-03	0.0105
ENST000003495330	-1.96	1.34E-02	0.0347
ANKRD20A8P	-1.96	1.44E-02	0.0365
NPIPB9	-1.96	1.46E-03	0.0093
AL139317.3	-1.96	2.85E-03	0.0134
TMOD2	-1.96	2.40E-04	0.0042
AC097662.1	-1.96	1.65E-02	0.0401
AL137244.1	-1.96	9.06E-03	0.0268
MT-RNR2	-1.96	1.69E-02	0.0409
CYB561D1	-1.96	1.74E-03	0.0102

AL355312.2	-1.97	1.60E-03	0.0098
PDE7A	-1.97	3.77E-04	0.0050
AL050343.1	-1.97	5.92E-03	0.0205
AC073476.3	-1.97	9.75E-03	0.0281
GSDMB	-1.97	2.74E-03	0.0131
AC010680.1	-1.97	7.59E-03	0.0239
CREG2	-1.97	5.13E-03	0.0187
ENST000003208920	-1.97	1.89E-03	0.0106
PTPRC	-1.97	2.94E-04	0.0045
SEMA6C	-1.97	6.70E-03	0.0222
FAM210B	-1.97	1.25E-04	0.0035
AL356599.1	-1.97	2.77E-04	0.0044
OSTN-AS1	-1.97	7.00E-03	0.0228
AC011825.3	-1.98	2.87E-03	0.0134
AC139720.1	-1.98	1.48E-03	0.0093
PARP4	-1.98	8.62E-05	0.0030
FRG1GP	-1.98	9.46E-03	0.0275
AC026803.1	-1.98	7.35E-03	0.0234
THEMIS	-1.98	6.49E-04	0.0063
AC005674.2	-1.98	3.53E-03	0.0150
PDE4B	-1.98	3.07E-04	0.0046
AC018644.1	-1.99	6.73E-04	0.0064
ENST000003571860	-1.99	5.68E-03	0.0200
ENST000003515780	-1.99	9.51E-04	0.0076
ZNF555	-1.99	2.11E-04	0.0041
DOCK9	-1.99	3.62E-04	0.0049
ZHX2	-1.99	6.20E-04	0.0062
SLC12A7	-2.00	8.64E-04	0.0072
LINC00476	-2.00	2.05E-03	0.0111
RPL11P3	-2.00	4.03E-04	0.0051
AC027020.2	-2.00	4.79E-03	0.0180
ENST000003240520	-2.00	1.03E-02	0.0291
AC027763.1	-2.00	9.27E-03	0.0271
ZNF101P2	-2.00	3.49E-03	0.0149
RIMBP3	-2.00	5.60E-03	0.0198
SLC46A3	-2.00	1.49E-02	0.0373
AC120498.9	-2.00	1.41E-02	0.0360
ZNF641	-2.01	1.52E-04	0.0036
NPR3	-2.01	2.10E-02	0.0479
PTPRCAP	-2.01	4.13E-03	0.0164
CASTOR3	-2.01	1.93E-03	0.0107
ENST000002947371	-2.01	1.29E-02	0.0339
AC006978.1	-2.01	4.97E-04	0.0056
AC093330.1	-2.01	7.23E-03	0.0232
ENST000002563244	-2.01	1.01E-02	0.0287
TRAJ48	-2.01	7.65E-04	0.0068
LINC00599	-2.01	1.19E-02	0.0321
MT-RNR1	-2.01	9.73E-03	0.0281
MIR181A1HG	-2.01	2.77E-03	0.0132
SNRPEP9	-2.02	6.52E-03	0.0218

PECAM1	-2.02	1.81E-04	0.0039
SLC8B1	-2.03	3.01E-04	0.0046
RCBTB2	-2.03	7.05E-04	0.0065
CYP4V2	-2.03	2.23E-04	0.0041
AC006960.3	-2.03	1.92E-03	0.0107
DNAH1	-2.03	2.34E-04	0.0042
AL359220.1	-2.03	9.74E-04	0.0076
ISG20	-2.04	2.88E-04	0.0045
ENST000002361921	-2.04	3.33E-04	0.0047
ACTG1P9	-2.04	6.50E-03	0.0218
HLA-V	-2.05	2.12E-02	0.0482
ROR1-AS1	-2.05	2.29E-04	0.0042
MIATNB	-2.05	2.37E-04	0.0042
TRAM2	-2.05	4.43E-04	0.0053
MIR663A	-2.05	7.12E-03	0.0230
AC048341.1	-2.05	1.16E-03	0.0083
TMEM184A	-2.05	3.09E-03	0.0140
PRKCQ-AS1	-2.05	7.03E-04	0.0065
CYP8B1	-2.05	5.74E-03	0.0201
AC110615.1	-2.05	8.17E-03	0.0251
AL512306.3	-2.05	1.37E-02	0.0352
LINC01320	-2.05	3.05E-03	0.0138
Z82206.1	-2.06	8.16E-04	0.0070
TMEM71	-2.06	1.05E-03	0.0079
ENST000003313431	-2.06	4.87E-03	0.0182
AKTIP	-2.06	2.72E-04	0.0044
ADRB2	-2.06	7.44E-03	0.0236
MAST3	-2.06	4.79E-03	0.0180
C8orf31	-2.06	9.95E-03	0.0285
SLC6A16	-2.06	4.52E-03	0.0174
DGCR9	-2.06	7.70E-03	0.0242
AC244216.3	-2.06	3.19E-03	0.0141
AC245056.4	-2.06	3.19E-03	0.0141
AC245056.5	-2.06	3.19E-03	0.0141
TSPAN14	-2.06	3.36E-03	0.0146
LINC02019	-2.07	3.63E-03	0.0152
ENST000002472262	-2.07	1.27E-02	0.0335
MIRLET7BHG	-2.07	1.11E-03	0.0081
OSTM1	-2.07	1.69E-04	0.0038
LSM14B	-2.07	1.64E-03	0.0099
CCDC121	-2.07	2.25E-03	0.0117
GPRASP1	-2.07	2.02E-04	0.0040
C16orf54	-2.07	3.66E-04	0.0049
ABCC2	-2.07	6.19E-03	0.0211
AL583810.1	-2.07	7.88E-03	0.0245
AC244034.2	-2.07	1.14E-03	0.0082
AC092656.1	-2.07	4.52E-04	0.0054
AL355574.1	-2.07	1.86E-02	0.0438
STMN3	-2.07	1.91E-03	0.0107
HRAT17	-2.08	7.06E-03	0.0229

GABARAPL1	-2.08	1.41E-03	0.0091
ZNF235	-2.08	9.61E-05	0.0031
AC009152.1	-2.08	3.16E-03	0.0141
LRRC63	-2.08	1.59E-02	0.0391
SNRK	-2.08	3.52E-03	0.0150
TPM2	-2.08	1.83E-03	0.0104
SH3BP5-AS1	-2.08	2.16E-03	0.0114
TTC21A	-2.08	2.18E-02	0.0491
AC027544.2	-2.08	1.23E-02	0.0329
LINC00298	-2.08	1.23E-02	0.0329
EFCAB13	-2.08	1.84E-03	0.0105
AC078795.2	-2.08	4.25E-03	0.0167
SMIM14	-2.08	9.38E-04	0.0075
AC018868.2	-2.08	3.76E-03	0.0156
TMEM150A	-2.08	2.03E-03	0.0110
KCTD7	-2.08	3.64E-04	0.0049
AC004865.2	-2.09	1.08E-02	0.0302
AL121845.3	-2.09	9.21E-04	0.0075
ARRDC3	-2.09	3.79E-04	0.0050
AC244230.2	-2.09	2.67E-03	0.0129
NLRP6	-2.09	1.32E-02	0.0343
AC084824.3	-2.09	1.34E-02	0.0347
ENST000003082341	-2.09	1.02E-03	0.0078
RNU6-60P	-2.09	8.31E-03	0.0254
LINC02324	-2.09	1.32E-02	0.0344
PRNCR1	-2.09	5.87E-04	0.0060
AC241952.1	-2.10	9.44E-04	0.0076
PNPLA2	-2.10	4.19E-03	0.0166
MALINC1	-2.10	3.54E-04	0.0048
WASF3-AS1	-2.10	2.94E-03	0.0136
RNU6-638P	-2.10	2.26E-03	0.0118
REM2	-2.10	6.79E-03	0.0224
LINC01825	-2.11	7.58E-03	0.0239
AC008906.1	-2.11	9.37E-04	0.0075
DDX58	-2.11	1.22E-04	0.0034
MIR548C	-2.11	7.04E-04	0.0065
AL513325.1	-2.11	8.35E-03	0.0254
ITGA4	-2.11	1.48E-04	0.0036
PYCARD	-2.11	2.95E-04	0.0046
SULT1B1	-2.11	1.92E-04	0.0039
IRS2	-2.11	2.05E-03	0.0111
Z98884.2	-2.12	4.44E-03	0.0172
GOLGA8M	-2.12	5.90E-03	0.0204
ENST000002700011	-2.12	7.81E-05	0.0030
AC104763.1	-2.12	8.25E-03	0.0253
TNS1	-2.12	9.26E-03	0.0271
PVRIG	-2.12	7.81E-03	0.0244
TP53INP1	-2.12	1.14E-03	0.0082
FRG1FP	-2.12	5.18E-03	0.0188
BSN-DT	-2.13	6.98E-03	0.0228

ADAMTS1	-2.13	9.07E-03	0.0268
AL109811.2	-2.13	7.35E-03	0.0234
NEUROD2	-2.13	9.42E-04	0.0075
SAMD12	-2.13	6.99E-04	0.0065
AL512306.2	-2.13	3.99E-03	0.0161
LINC02328	-2.13	2.66E-04	0.0044
AC007620.3	-2.13	1.57E-02	0.0387
DLEC1	-2.14	1.97E-03	0.0108
KLHDC1	-2.14	3.16E-04	0.0046
LINC00528	-2.14	9.61E-03	0.0278
SNAI3	-2.14	5.23E-04	0.0057
AC010326.4	-2.14	4.48E-03	0.0173
SGK3	-2.14	8.41E-05	0.0030
TET1	-2.14	2.06E-03	0.0111
SPRY3	-2.14	1.39E-03	0.0090
AL022328.3	-2.15	1.75E-02	0.0418
RNU1-106P	-2.15	1.75E-02	0.0418
KAT2B	-2.15	1.72E-03	0.0101
CA5B	-2.15	3.63E-03	0.0152
SYCP3	-2.15	7.43E-03	0.0236
SOS1-IT1	-2.15	1.54E-03	0.0096
BNIP3P5	-2.15	2.36E-03	0.0121
AC008280.3	-2.15	4.15E-03	0.0165
DHRS4L1	-2.15	1.67E-02	0.0405
AC079921.2	-2.15	4.49E-03	0.0173
BCAS3	-2.15	2.16E-04	0.0041
ENST00000480419	-2.15	3.22E-04	0.0047
ENST000003328840	-2.15	4.91E-04	0.0056
GREM2	-2.15	1.17E-02	0.0317
IRGM	-2.15	5.46E-03	0.0195
EEF1DP7	-2.16	1.21E-03	0.0084
AP003352.1	-2.16	3.79E-03	0.0156
LINC01967	-2.16	1.06E-02	0.0297
SEPT14P19	-2.16	5.16E-03	0.0188
LMO7-AS1	-2.16	4.54E-03	0.0175
AL589642.2	-2.16	1.40E-02	0.0358
SIGIRR	-2.16	1.86E-04	0.0039
ENST000002817723	-2.16	2.18E-03	0.0115
SNORD116-24	-2.17	2.02E-02	0.0465
AGGF1P2	-2.17	1.07E-02	0.0300
ZNF763	-2.17	1.30E-03	0.0087
LINC002481	-2.17	1.64E-02	0.0400
ZNF204P	-2.17	1.13E-02	0.0309
AL353807.3	-2.17	1.30E-03	0.0087
RN7SKP56	-2.17	2.99E-03	0.0137
TTYH2	-2.17	9.33E-04	0.0075
PEG13	-2.18	9.76E-03	0.0281
LRRC6	-2.18	9.49E-04	0.0076
AC022113.1	-2.18	6.89E-03	0.0226
AL031667.3	-2.18	1.10E-02	0.0304

ENST000003387450	-2.18	1.90E-04	0.0039
CDKL4	-2.18	1.06E-02	0.0297
AP003715.1	-2.18	1.56E-02	0.0387
PSMD6-AS2	-2.18	5.64E-04	0.0059
CTC1	-2.18	1.99E-02	0.0459
TRBJ1-6	-2.18	4.99E-03	0.0184
ACTG1P3	-2.19	2.09E-03	0.0112
ZNF582	-2.19	1.57E-03	0.0097
FP236383.1	-2.19	4.77E-04	0.0055
FP671120.1	-2.19	4.77E-04	0.0055
AC018638.7	-2.19	9.44E-03	0.0274
ENST000002893610	-2.19	2.71E-04	0.0044
FAM117A	-2.19	3.36E-04	0.0047
LINC00205	-2.19	6.92E-04	0.0064
AC100827.4	-2.19	1.65E-02	0.0402
SLCO3A1	-2.19	7.98E-03	0.0247
PARP8	-2.19	1.04E-04	0.0033
AC125616.1	-2.20	1.70E-02	0.0411
ADAMTS17	-2.20	4.91E-03	0.0183
EMBP1	-2.21	1.57E-03	0.0097
PLEKHA1	-2.21	5.89E-04	0.0060
NLRC3	-2.21	5.90E-04	0.0060
TENT5A	-2.21	2.30E-03	0.0119
AL136038.4	-2.21	2.10E-03	0.0113
RAP2C-AS1	-2.21	1.26E-03	0.0086
APOC4	-2.21	1.52E-02	0.0380
SETP20	-2.21	5.86E-03	0.0204
LY9	-2.22	1.30E-02	0.0339
S1PR4	-2.22	6.76E-04	0.0064
AC109449.1	-2.22	9.97E-03	0.0285
SNORD116-17	-2.22	3.77E-03	0.0156
SNORD116-19	-2.22	3.77E-03	0.0156
PLG	-2.22	3.06E-03	0.0139
AC087741.1	-2.22	5.30E-04	0.0058
AL592148.3	-2.22	1.61E-04	0.0037
ANXA1	-2.22	2.41E-03	0.0122
GXYLT2	-2.22	1.44E-02	0.0366
LRRC25	-2.22	1.28E-02	0.0338
ZNF836	-2.23	1.64E-04	0.0037
ARHGAP15	-2.23	2.32E-04	0.0042
AF127577.4	-2.23	2.79E-03	0.0132
RAD51-AS1	-2.23	1.26E-04	0.0035
C1DP5	-2.23	4.83E-03	0.0181
SNHG22	-2.23	1.72E-03	0.0101
RF00017.25	-2.23	6.35E-03	0.0214
AL109933.1	-2.23	5.45E-03	0.0195
RASSF3	-2.23	3.20E-04	0.0047
AL356273.2	-2.23	3.16E-03	0.0141
RNF222	-2.23	1.57E-02	0.0389
OPCML-IT1	-2.24	1.47E-03	0.0093

SYTL2	-2.24	4.31E-03	0.0169
ATF7IP2	-2.24	1.80E-04	0.0039
CCT4P2	-2.24	1.22E-03	0.0084
TRPV2	-2.24	5.57E-03	0.0197
SUN2	-2.24	1.88E-04	0.0039
LIMD1-AS1	-2.24	1.71E-02	0.0412
CCDC7	-2.24	1.91E-04	0.0039
UTS2B	-2.24	2.08E-03	0.0112
FAM102A	-2.24	3.59E-04	0.0049
LCMT1-AS2	-2.25	1.78E-02	0.0425
FRAT1	-2.25	1.23E-03	0.0085
TRAT1	-2.25	3.67E-04	0.0049
ZNF69	-2.25	9.34E-03	0.0273
AC026356.2	-2.25	1.18E-03	0.0083
TOM1L2	-2.25	5.17E-04	0.0057
EPSTI1	-2.25	6.86E-03	0.0225
HELZ2	-2.26	2.10E-03	0.0113
LIMD2	-2.26	1.40E-04	0.0036
SNPH	-2.26	3.39E-03	0.0146
ENST000003474660	-2.26	2.75E-03	0.0131
RN7SL258P	-2.26	3.50E-03	0.0149
ENST000003193630	-2.26	2.45E-04	0.0042
AC079781.5	-2.26	8.89E-04	0.0073
AC008676.1	-2.26	9.23E-03	0.0271
TRIM62	-2.27	1.24E-02	0.0330
AC023908.3	-2.27	3.69E-03	0.0154
APBA2	-2.27	2.89E-03	0.0135
ITGB7	-2.27	3.09E-04	0.0046
DISC1	-2.27	6.91E-04	0.0064
AL138694.1	-2.27	3.54E-03	0.0150
SKINT1L	-2.27	3.65E-04	0.0049
FBXO32	-2.27	1.19E-04	0.0034
AL390195.2	-2.28	6.11E-03	0.0209
PARP15	-2.28	6.30E-04	0.0062
XYLT1	-2.28	1.29E-03	0.0087
CAPS	-2.28	1.11E-04	0.0033
FGD3	-2.28	1.04E-04	0.0033
PELI2	-2.28	3.39E-03	0.0146
AL513534.1	-2.28	3.23E-04	0.0047
SHISAL2A	-2.28	1.90E-03	0.0106
C5orf56	-2.28	3.16E-04	0.0046
AL139415.1	-2.28	6.40E-04	0.0062
AC122710.2	-2.29	1.94E-03	0.0107
AC109454.2	-2.29	3.11E-03	0.0140
NRBP2	-2.29	2.71E-04	0.0044
CALCOCO1	-2.29	2.12E-04	0.0041
S1PR1	-2.29	4.32E-03	0.0169
TPRXL	-2.29	2.68E-04	0.0044
TCEANC	-2.29	4.45E-04	0.0053
HEATR6	-2.29	3.28E-03	0.0144

ENST000002957560	-2.30	3.32E-03	0.0145
AC008014.1	-2.30	8.28E-04	0.0071
Z93930.3	-2.30	5.22E-04	0.0057
WDR81	-2.30	5.79E-05	0.0028
ACKR3	-2.31	2.91E-03	0.0135
AC084036.1	-2.31	9.04E-04	0.0074
FAM214A	-2.31	5.65E-05	0.0028
AC069281.2	-2.31	2.65E-03	0.0128
CHRM3-AS2	-2.31	1.70E-03	0.0101
TMTC1	-2.31	1.48E-02	0.0371
RASA2-IT1	-2.32	1.44E-02	0.0365
DYRK2	-2.32	1.68E-04	0.0038
WHAMMP2	-2.32	1.98E-04	0.0040
AC133552.4	-2.32	3.05E-03	0.0139
ERBB3	-2.32	2.99E-03	0.0137
AC011939.3	-2.32	6.52E-03	0.0218
NLRP1	-2.32	1.18E-04	0.0034
ENST000003382572	-2.32	4.50E-04	0.0054
AC092794.1	-2.32	1.66E-02	0.0404
RAB37	-2.32	3.16E-03	0.0141
RPS6KA5	-2.33	1.81E-04	0.0039
ARMC2	-2.33	3.19E-03	0.0141
ENST00000557775	-2.33	1.08E-02	0.0301
ZNF33B	-2.33	7.54E-05	0.0030
AC015911.8	-2.33	1.93E-03	0.0107
LINC00893	-2.34	8.76E-03	0.0262
LINC01730	-2.34	2.25E-03	0.0118
TSC22D3	-2.34	7.59E-04	0.0067
AL121890.4	-2.34	9.41E-03	0.0274
PBXIP1	-2.34	4.11E-04	0.0051
DPPA4	-2.34	8.27E-03	0.0253
AC006001.2	-2.34	1.34E-03	0.0088
AC006064.2	-2.34	2.07E-02	0.0474
AXIN2	-2.34	3.11E-04	0.0046
LOXL2	-2.34	7.73E-04	0.0068
C20orf203	-2.34	8.38E-03	0.0255
ENST000003156842	-2.34	2.27E-04	0.0041
RASA3	-2.35	6.50E-05	0.0028
AMY2B	-2.35	7.60E-04	0.0067
KRT18P61	-2.35	4.60E-03	0.0176
IFIT1	-2.35	4.06E-03	0.0163
PYGM	-2.35	1.26E-03	0.0086
GSAP	-2.35	7.47E-04	0.0067
SERINC5	-2.35	2.22E-04	0.0041
LINC00092	-2.35	1.31E-02	0.0342
RPL5P13	-2.35	7.03E-03	0.0228
RPL7L1P12	-2.36	3.37E-03	0.0146
CADM2-AS1	-2.36	2.90E-03	0.0135
FAM204BP	-2.36	3.78E-03	0.0156
FAM209A	-2.36	2.83E-03	0.0133

GTF2IRD2B	-2.37	1.65E-04	0.0037
ENST000002601971	-2.37	1.82E-04	0.0039
TNRC6C-AS1	-2.37	3.92E-04	0.0050
CDC42BPG	-2.37	2.33E-03	0.0120
CCDC180	-2.37	6.96E-04	0.0065
SCARB2	-2.38	7.41E-05	0.0029
TMEM229B	-2.38	5.22E-03	0.0190
PKMP3	-2.38	1.85E-02	0.0437
AC138915.3	-2.38	9.80E-03	0.0282
ADCY10P1	-2.38	5.88E-04	0.0060
PARP12	-2.38	4.19E-04	0.0052
YPEL2	-2.38	1.02E-03	0.0078
AC025171.3	-2.38	3.56E-03	0.0150
CHIAP2	-2.38	3.01E-03	0.0137
AC090948.1	-2.38	3.60E-04	0.0049
AC084816.1	-2.38	3.34E-04	0.0047
CLEC18A	-2.38	3.67E-03	0.0153
OR52N4	-2.38	5.31E-04	0.0058
AC107980.1	-2.38	7.37E-03	0.0235
AC243686.1	-2.38	7.37E-03	0.0235
AL160408.3	-2.38	7.37E-03	0.0235
VPS9D1	-2.39	6.20E-03	0.0211
AC025164.1	-2.39	2.69E-03	0.0130
ENST000002841650	-2.39	2.25E-04	0.0041
GVINP1	-2.39	8.13E-04	0.0070
AC245100.4	-2.39	2.73E-04	0.0044
DSEL	-2.39	1.60E-02	0.0394
WDR78	-2.40	9.84E-04	0.0077
RF00026.108	-2.40	2.52E-03	0.0125
ITPK1-AS1	-2.40	2.86E-03	0.0134
SMIM10L2A	-2.40	1.65E-03	0.0099
ERO1B	-2.42	5.05E-04	0.0056
AC074138.1	-2.42	2.71E-04	0.0044
AC007256.1	-2.42	6.74E-03	0.0223
AC013731.1	-2.42	2.16E-04	0.0041
ITGAM	-2.42	9.03E-03	0.0267
AF279873.3	-2.42	1.42E-02	0.0362
LINC02109	-2.42	1.05E-02	0.0296
LINC01609	-2.42	9.00E-04	0.0074
ARHGAP27P1-BPTFP1-KPNA2P3	-2.43	1.59E-04	0.0037
EEF1DP2	-2.43	1.53E-03	0.0095
AC126177.4	-2.43	1.83E-02	0.0433
AC024884.2	-2.43	2.27E-03	0.0118
KCNMB2-AS1	-2.43	1.13E-02	0.0310
FLJ42102	-2.43	1.26E-02	0.0334
AC079922.2	-2.43	4.20E-04	0.0052
AL627309.1	-2.44	1.84E-03	0.0105
NKD1	-2.44	3.68E-04	0.0049
ENST000003142611	-2.44	6.76E-05	0.0029
AC159540.2	-2.45	8.88E-05	0.0031

KCNA2	-2.45	1.47E-02	0.0370
EVL	-2.45	2.19E-04	0.0041
PPP3CB-AS1	-2.45	9.91E-05	0.0032
CAPN8	-2.45	6.44E-03	0.0216
CD180	-2.46	6.39E-03	0.0215
AL591623.1	-2.46	1.52E-04	0.0036
AC011816.1	-2.46	2.79E-03	0.0133
GPR18	-2.46	5.63E-03	0.0198
PDE3B	-2.46	5.94E-04	0.0060
RBM43	-2.46	1.37E-03	0.0089
ICAM4	-2.46	4.70E-03	0.0178
AC068134.3	-2.47	1.11E-03	0.0080
ANKRD55	-2.47	5.64E-03	0.0199
MYO1F	-2.48	5.77E-05	0.0028
AC022306.2	-2.48	5.29E-03	0.0191
GYS2	-2.48	1.67E-02	0.0406
RPS3AP29	-2.48	1.72E-02	0.0413
AC110994.2	-2.48	1.94E-03	0.0107
AC242842.1	-2.48	4.32E-04	0.0052
TLR3	-2.48	3.60E-03	0.0152
TAS2R3	-2.48	5.96E-03	0.0206
BOC	-2.48	3.99E-04	0.0051
ENST000003284030	-2.49	3.03E-04	0.0046
CDKN2B-AS1	-2.49	1.01E-03	0.0077
B3GALT4	-2.49	1.07E-04	0.0033
CAMK4	-2.49	6.48E-04	0.0063
ABCA10	-2.49	1.70E-02	0.0411
TIPARP	-2.49	1.31E-04	0.0035
TMEM86A	-2.50	1.45E-02	0.0368
RF00017.176	-2.50	5.95E-03	0.0205
ENST000003567660	-2.50	9.54E-03	0.0277
TKTL1	-2.50	1.03E-03	0.0078
AC106799.3	-2.50	9.10E-03	0.0268
NUAK2	-2.50	7.22E-03	0.0231
AC127521.1	-2.51	2.15E-02	0.0487
BIN2	-2.51	3.74E-05	0.0023
RBL2	-2.51	7.40E-05	0.0029
ENST000002762040	-2.51	4.59E-05	0.0025
AC011379.2	-2.51	2.15E-04	0.0041
AC015911.4	-2.51	3.65E-04	0.0049
AC055822.1	-2.51	1.39E-03	0.0090
AL034550.2	-2.52	3.11E-03	0.0140
AC006369.1	-2.52	2.06E-03	0.0111
AC240274.1	-2.52	1.01E-02	0.0287
GBP3	-2.52	1.08E-04	0.0033
CFP	-2.52	3.81E-04	0.0050
AC104825.1	-2.52	8.19E-04	0.0070
ENST000002779002	-2.52	2.69E-04	0.0044
OVGP1	-2.52	5.10E-04	0.0056
CEACAM1	-2.52	1.59E-02	0.0391

LINC02009	-2.52	4.91E-03	0.0183
FHIT	-2.52	5.96E-05	0.0028
ENST000002532701	-2.53	3.10E-04	0.0046
AC010619.2	-2.53	9.16E-03	0.0270
OBSCN-AS1	-2.53	1.51E-02	0.0378
CHMP7	-2.54	9.44E-05	0.0031
AL157935.2	-2.54	1.26E-03	0.0086
AC092652.3	-2.54	2.13E-04	0.0041
KIF4CP	-2.55	7.87E-03	0.0245
CDC14A	-2.55	1.17E-04	0.0034
ZMYM1	-2.55	9.88E-04	0.0077
KCNA3	-2.55	4.88E-03	0.0182
AC009133.3	-2.56	1.73E-03	0.0102
SORL1	-2.56	3.68E-04	0.0049
TTN	-2.56	1.57E-04	0.0037
AC091488.1	-2.56	1.05E-02	0.0295
AC011632.1	-2.57	5.30E-03	0.0191
LINC01138	-2.57	1.93E-04	0.0039
LINC02472	-2.58	7.17E-04	0.0065
ENST000003503200	-2.58	4.42E-03	0.0172
SLC4A5	-2.58	1.55E-04	0.0036
SYNE1	-2.58	9.29E-04	0.0075
PTENP1	-2.58	5.00E-03	0.0185
PRKACB	-2.59	3.49E-04	0.0048
ERP27	-2.59	1.04E-04	0.0033
LIPC	-2.59	1.37E-03	0.0089
AL365203.2	-2.59	3.67E-03	0.0153
ADD3	-2.59	2.01E-04	0.0040
AC009495.2	-2.60	5.22E-03	0.0190
DNAJC19P5	-2.60	1.01E-03	0.0078
CTSO	-2.60	3.61E-04	0.0049
AC110792.3	-2.60	7.26E-03	0.0232
LINC00920	-2.61	4.50E-04	0.0054
AC092681.3	-2.61	1.08E-02	0.0302
CTSF	-2.61	1.99E-03	0.0109
AC139887.4	-2.61	6.68E-03	0.0222
AC138761.3	-2.62	1.83E-03	0.0104
RPSAP48	-2.62	5.04E-04	0.0056
AC092653.1	-2.62	2.81E-03	0.0133
MIR4473	-2.62	1.35E-03	0.0089
LINC01145	-2.62	1.16E-03	0.0082
AC245297.3	-2.62	1.09E-04	0.0033
DPYD	-2.63	1.67E-04	0.0038
RNF144B	-2.63	1.11E-04	0.0033
GPR15	-2.63	1.76E-03	0.0102
PIWIL4	-2.63	1.50E-02	0.0376
CTXND1	-2.63	2.62E-03	0.0128
RNU6-202P	-2.64	7.13E-04	0.0065
ADCY4	-2.64	8.14E-04	0.0070
AL355103.1	-2.64	9.38E-03	0.0274

ANKEF1	-2.64	7.24E-05	0.0029
AC245519.2	-2.64	1.77E-02	0.0422
AL135818.2	-2.65	5.84E-04	0.0060
SOS1	-2.65	5.83E-04	0.0060
LINC01891	-2.65	8.72E-03	0.0262
CDHR3	-2.65	7.83E-03	0.0244
ENST000002576261	-2.65	4.06E-03	0.0163
FAM238C	-2.66	9.85E-03	0.0283
TMEM63A	-2.66	3.42E-05	0.0023
SUDS3P1	-2.66	2.55E-03	0.0126
IL10RA	-2.66	2.34E-05	0.0021
ENST000002618371	-2.66	2.03E-03	0.0110
LINC02035	-2.66	1.77E-04	0.0039
PPEF2	-2.66	7.39E-03	0.0235
GRAPL	-2.67	4.19E-04	0.0052
NLGN3	-2.67	8.28E-04	0.0071
ATXN7	-2.67	8.43E-03	0.0256
ALS2CL	-2.67	5.41E-04	0.0058
YPEL3	-2.68	6.71E-04	0.0064
SYNE2	-2.68	9.51E-05	0.0031
AC018809.2	-2.68	3.30E-03	0.0144
ENST000003019233	-2.68	4.15E-03	0.0165
STK38	-2.69	4.84E-05	0.0026
RN7SL517P	-2.69	8.32E-04	0.0071
AC064834.1	-2.70	1.34E-02	0.0347
AC099568.1	-2.70	1.58E-02	0.0389
AC011978.2	-2.70	8.47E-03	0.0257
KLHL24	-2.70	1.45E-04	0.0036
SYNM	-2.71	1.54E-02	0.0383
CCR5	-2.71	3.49E-03	0.0149
TNNT3	-2.71	3.14E-03	0.0141
AC006299.1	-2.71	9.17E-03	0.0270
AC104162.1	-2.72	8.17E-03	0.0251
ENST000003046210	-2.72	6.67E-03	0.0221
AC025031.4	-2.72	6.81E-04	0.0064
KIAA1551	-2.73	1.92E-04	0.0039
LINC00869	-2.73	1.32E-04	0.0035
PTPRN2	-2.73	4.37E-04	0.0053
AC016957.1	-2.73	5.54E-03	0.0196
ZBTB20	-2.74	2.64E-04	0.0044
MIR374B	-2.74	1.79E-02	0.0425
AC233309.1	-2.74	6.94E-03	0.0227
RPL10P19	-2.74	3.52E-03	0.0150
TXNIP	-2.75	3.85E-05	0.0023
PTPRO	-2.75	6.75E-03	0.0223
ZNF276	-2.75	2.29E-05	0.0020
SNX18	-2.75	9.43E-05	0.0031
AL008638.3	-2.75	1.15E-02	0.0314
IPCEF1	-2.75	4.36E-03	0.0170
ENST000002959241	-2.76	6.64E-05	0.0029

OR52U1P	-2.76	4.03E-03	0.0162
AC073111.5	-2.77	1.85E-04	0.0039
TMEM200A	-2.78	1.51E-02	0.0378
FCMR	-2.78	6.99E-04	0.0065
AL596220.1	-2.78	2.80E-04	0.0045
P2RY8	-2.78	2.21E-04	0.0041
ENST000003184450	-2.78	8.15E-05	0.0030
CREBRF	-2.79	4.17E-05	0.0024
AL354809.1	-2.79	1.59E-03	0.0097
AC090921.1	-2.79	3.12E-03	0.0140
OR7E7P	-2.79	2.64E-03	0.0128
AC092375.2	-2.79	5.02E-03	0.0185
AC241988.2	-2.79	5.02E-03	0.0185
AL392023.2	-2.79	9.79E-03	0.0282
SULT1C4	-2.80	5.50E-03	0.0196
SELPLG	-2.81	3.56E-05	0.0023
AC092111.1	-2.81	8.58E-03	0.0259
AC239804.1	-2.81	1.84E-02	0.0434
DIP2C	-2.81	1.73E-02	0.0414
XAF1	-2.81	7.91E-03	0.0246
DPP4	-2.82	5.71E-04	0.0059
ENST000002428100	-2.82	8.84E-05	0.0031
KCNRG	-2.82	9.02E-03	0.0267
AC090186.1	-2.83	5.00E-03	0.0185
LINC00304	-2.83	9.57E-03	0.0277
AL162391.1	-2.83	2.89E-03	0.0135
SESN3	-2.83	9.51E-04	0.0076
ZNF449	-2.84	6.80E-04	0.0064
C12orf42	-2.84	1.60E-02	0.0394
SLC35D2	-2.84	1.38E-04	0.0036
AC127024.4	-2.85	5.02E-03	0.0185
AP003028.1	-2.85	5.51E-03	0.0196
CCDC141	-2.85	4.41E-04	0.0053
ZBP1	-2.86	1.32E-04	0.0035
EFHC2	-2.87	7.81E-03	0.0243
MIR2355	-2.87	2.68E-03	0.0129
ATM	-2.87	1.73E-04	0.0038
AC135050.5	-2.87	1.01E-02	0.0288
AL365361.1	-2.87	1.22E-03	0.0084
AC090950.1	-2.88	4.09E-03	0.0164
AC138207.6	-2.88	5.59E-03	0.0198
IMPG2	-2.88	8.29E-05	0.0030
AC068790.7	-2.88	2.67E-03	0.0129
AP003717.1	-2.88	6.89E-03	0.0226
AL158163.1	-2.89	9.50E-05	0.0031
SAMD9L	-2.90	1.16E-03	0.0083
SMPD1	-2.90	1.06E-04	0.0033
ABCD2	-2.90	4.42E-04	0.0053
AC138123.1	-2.90	2.86E-03	0.0134
TPTE2P5	-2.90	4.25E-03	0.0167

AC009035.1	-2.91	2.47E-03	0.0124
AC022079.1	-2.91	3.96E-04	0.0050
AL445649.1	-2.91	4.41E-03	0.0172
NSG1	-2.92	1.89E-02	0.0443
AL021707.8	-2.92	1.25E-03	0.0086
AKR1C8P	-2.92	5.30E-03	0.0191
FYB1	-2.92	7.94E-04	0.0069
CATSPERB	-2.93	2.12E-02	0.0481
AC036176.1	-2.93	2.55E-03	0.0126
AL158163.2	-2.93	1.76E-04	0.0039
LINC02033	-2.93	8.19E-04	0.0070
ZNF652	-2.93	2.98E-05	0.0022
SCML4	-2.93	1.33E-03	0.0088
AL158196.1	-2.93	3.92E-03	0.0160
CTSW	-2.94	1.76E-03	0.0102
LINC01336	-2.94	1.02E-02	0.0289
ENST000003387970	-2.95	4.65E-04	0.0054
AC004492.1	-2.96	8.74E-04	0.0072
AC004492.2	-2.96	8.74E-04	0.0072
ENST000002669430	-2.96	1.20E-04	0.0034
PLA2G2D	-2.96	6.04E-03	0.0208
STMN1P1	-2.97	2.30E-03	0.0119
SLC26A11	-2.97	4.48E-03	0.0173
TJP3	-2.97	4.19E-04	0.0052
ZNF540	-2.98	2.89E-05	0.0022
RSAD2	-2.98	2.09E-02	0.0476
E2F3P2	-2.98	2.40E-03	0.0122
AC009754.1	-2.98	7.06E-04	0.0065
RTP4	-2.98	3.83E-04	0.0050
IL11RA	-2.98	7.30E-05	0.0029
SAMD9	-2.99	2.45E-04	0.0042
ZBTB18	-2.99	2.21E-04	0.0041
FMN1	-2.99	3.15E-03	0.0141
CFH	-2.99	2.23E-02	0.0499
ZMAT1	-3.00	5.39E-04	0.0058
CARD16	-3.00	6.69E-04	0.0064
TSPAN32	-3.00	4.60E-03	0.0176
ENST000002353721	-3.01	1.62E-03	0.0098
AL359885.1	-3.01	1.29E-03	0.0087
ZNF815P	-3.01	3.09E-04	0.0046
PTPN4	-3.02	2.42E-05	0.0021
LOXHD1	-3.03	3.80E-03	0.0157
ENST000003263511	-3.03	6.13E-03	0.0209
AC241640.1	-3.04	3.75E-03	0.0156
PYROXD2	-3.05	1.68E-04	0.0038
KPNA2P3	-3.05	7.77E-05	0.0030
GIMAP1	-3.06	7.67E-05	0.0030
LTB	-3.07	1.43E-04	0.0036
ZNF564	-3.07	7.43E-03	0.0236
C1GALT1P1	-3.07	9.03E-04	0.0074

PCAT19	-3.07	9.70E-04	0.0076
COLQ	-3.08	8.78E-05	0.0031
AZIN2	-3.08	1.83E-02	0.0434
AC011450.1	-3.08	3.58E-03	0.0151
AC005332.2	-3.08	5.31E-04	0.0058
CCDC146	-3.09	1.66E-04	0.0038
TCP11L2	-3.09	1.27E-04	0.0035
TMEM191A	-3.10	3.03E-03	0.0138
NMRAL2P	-3.10	4.27E-03	0.0168
HSPE1P13	-3.10	1.13E-03	0.0081
VMAC	-3.11	1.33E-04	0.0035
ENST000002824881	-3.11	5.64E-04	0.0059
AC006023.2	-3.12	2.15E-03	0.0114
AC078962.1	-3.13	3.42E-03	0.0147
EVI2B	-3.13	1.69E-03	0.0101
AC027373.1	-3.14	8.39E-05	0.0030
RPL21P44	-3.15	6.88E-04	0.0064
AL591895.1	-3.15	7.04E-04	0.0065
LINC01876	-3.15	1.02E-02	0.0289
MGC16275	-3.16	8.42E-03	0.0256
AC016957.2	-3.16	1.85E-04	0.0039
IDUA	-3.16	4.70E-04	0.0054
ENST000003615440	-3.16	1.31E-03	0.0087
LPAR6	-3.16	1.08E-05	0.0017
ANTXR2	-3.17	3.56E-04	0.0048
AC040162.3	-3.17	1.64E-03	0.0099
SPSB2	-3.17	1.29E-03	0.0087
SAMHD1	-3.17	1.28E-04	0.0035
AL157762.1	-3.18	2.70E-03	0.0130
AC012651.1	-3.18	1.47E-03	0.0093
TXK	-3.19	1.25E-05	0.0018
PTTG2	-3.19	2.65E-03	0.0129
LRRN3	-3.19	1.34E-05	0.0018
MAP1A	-3.19	1.31E-02	0.0342
EAF1-AS1	-3.20	1.52E-02	0.0379
AC025171.5	-3.21	6.26E-03	0.0212
LINC01176	-3.21	6.70E-04	0.0064
AL773551.1	-3.23	1.89E-02	0.0442
AC000068.3	-3.23	6.96E-03	0.0227
TMEM45B	-3.24	2.94E-04	0.0045
LINC02361	-3.25	3.88E-04	0.0050
HSPE1P3	-3.26	1.34E-03	0.0088
HIGD1AP14	-3.26	9.43E-03	0.0274
HAVCR1	-3.26	5.83E-04	0.0060
AC048382.6	-3.27	5.31E-03	0.0191
AC025171.2	-3.27	1.81E-04	0.0039
AC008543.5	-3.27	2.68E-03	0.0129
PNPLA7	-3.27	3.09E-03	0.0140
MXD4	-3.27	4.57E-04	0.0054
ABCA7	-3.28	3.77E-04	0.0050

RNU6-807P	-3.28	1.19E-03	0.0084
GIMAP7	-3.29	8.68E-05	0.0031
PPIAL4G	-3.30	5.14E-05	0.0027
RARRES3	-3.30	2.33E-04	0.0042
GOLGA5P1	-3.30	3.69E-03	0.0154
PLCL1	-3.32	1.90E-04	0.0039
SPON1	-3.33	3.46E-03	0.0148
RNF144A	-3.33	1.91E-04	0.0039
LAIR1	-3.34	1.87E-05	0.0020
NUDT16L1	-3.34	7.05E-04	0.0065
LINC00954	-3.34	1.74E-02	0.0418
AC119044.1	-3.35	2.10E-04	0.0040
GPR61	-3.35	2.45E-03	0.0123
NR3C2	-3.35	1.24E-03	0.0085
CD96	-3.36	4.02E-05	0.0024
ENST000003056232	-3.37	7.71E-03	0.0242
IFNG-AS1	-3.37	3.09E-03	0.0140
PIK3IP1	-3.38	2.24E-04	0.0041
CUBN	-3.39	3.53E-04	0.0048
RF02271.32	-3.39	2.52E-03	0.0125
RNU6-838P	-3.40	3.54E-03	0.0150
AC114490.1	-3.40	2.11E-03	0.0113
AL391056.1	-3.42	7.32E-03	0.0234
ENST00000588041	-3.43	1.07E-03	0.0080
CEACAMP3	-3.44	1.02E-03	0.0078
AC004067.1	-3.44	9.46E-04	0.0076
GCNT4	-3.45	1.38E-03	0.0090
ZFP14	-3.45	2.08E-04	0.0040
TC2N	-3.45	1.46E-04	0.0036
AC093725.2	-3.45	8.60E-04	0.0072
AF165147.1	-3.47	2.88E-03	0.0134
C11orf21	-3.47	8.40E-03	0.0255
CNTN6	-3.47	1.61E-02	0.0395
LRFN2	-3.47	1.61E-02	0.0395
DPYD-IT1	-3.49	1.17E-04	0.0034
C16orf74	-3.50	1.56E-02	0.0386
LINC00921	-3.50	2.64E-03	0.0128
AC091180.2	-3.51	1.55E-02	0.0385
CASP1P2	-3.51	1.29E-03	0.0087
AC093726.2	-3.51	9.90E-04	0.0077
LDLRAP1	-3.52	1.34E-04	0.0035
AL591926.7	-3.52	9.19E-04	0.0075
AC134669.1	-3.54	1.02E-03	0.0078
SFR1P1	-3.57	1.45E-02	0.0368
MAN1C1	-3.57	5.17E-04	0.0057
AL391994.1	-3.59	1.24E-02	0.0330
MIR4441	-3.59	1.21E-02	0.0325
AP000977.1	-3.60	5.73E-04	0.0059
ENST000003466520	-3.60	8.83E-04	0.0073
AC055764.2	-3.60	1.46E-02	0.0370

AC119396.1	-3.60	5.43E-05	0.0027
NT5C3AP1	-3.61	1.05E-03	0.0079
TRBV23-1	-3.61	6.09E-05	0.0028
SMIM10L2B-AS1	-3.61	1.19E-02	0.0321
ADAMTS10	-3.64	1.25E-04	0.0035
OLFM2	-3.64	4.05E-03	0.0163
ENST000003340031	-3.64	1.07E-03	0.0079
LPAL2	-3.65	1.48E-02	0.0372
ABTB1	-3.67	6.05E-03	0.0208
ANXA2R	-3.67	3.72E-04	0.0049
AC026355.2	-3.72	2.17E-02	0.0489
CCDC89	-3.72	2.17E-02	0.0489
MED28P7	-3.72	1.08E-02	0.0300
ANTXRLP1	-3.72	1.23E-04	0.0034
C1orf162	-3.72	3.21E-05	0.0023
SLC9A9	-3.73	6.26E-05	0.0028
AC009084.1	-3.73	1.03E-02	0.0292
LINC00544	-3.73	1.79E-04	0.0039
NUTM2B	-3.73	2.13E-02	0.0484
NR1D2	-3.74	5.06E-05	0.0026
NRIR	-3.75	2.13E-02	0.0483
RAD23BP1	-3.75	3.59E-04	0.0049
HLTF-AS1	-3.76	1.01E-02	0.0288
OLFM5P	-3.76	1.05E-02	0.0294
ARHGEF11	-3.76	8.55E-05	0.0030
AC005532.2	-3.77	4.82E-04	0.0055
ENST000003430171	-3.77	9.74E-04	0.0076
AC010983.1	-3.78	1.03E-03	0.0078
AC092902.2	-3.78	2.23E-02	0.0499
LINC01163	-3.79	1.76E-03	0.0102
ST8SIA6	-3.80	9.19E-03	0.0270
AQP3	-3.81	1.74E-04	0.0038
RIPOR2	-3.82	7.03E-05	0.0029
RF00017.53	-3.82	9.61E-03	0.0278
DDX60L	-3.83	1.88E-04	0.0039
AL117350.1	-3.83	8.99E-03	0.0267
MUC20-OT1-585	-3.84	5.44E-03	0.0194
AL121839.2	-3.84	4.86E-05	0.0026
AL121839.4	-3.84	4.86E-05	0.0026
AC090948.2	-3.86	7.50E-05	0.0030
TRANK1	-3.87	1.03E-03	0.0078
ENST000003403420	-3.88	3.90E-03	0.0159
AC008878.3	-3.88	4.11E-05	0.0024
ISM1	-3.88	8.62E-04	0.0072
MEGF6	-3.90	4.25E-04	0.0052
AL357033.4	-3.90	1.25E-03	0.0086
LINC01725	-3.90	1.49E-03	0.0094
AC013468.1	-3.92	4.04E-04	0.0051
SMPD3	-3.93	5.16E-03	0.0188
VIPR1	-3.95	5.09E-04	0.0056

AL133355.1	-3.95	1.51E-03	0.0094
EPHA4	-3.95	2.26E-04	0.0041
SLFN5	-3.96	1.28E-03	0.0086
AL135790.1	-3.96	6.17E-04	0.0061
UBQLNL	-3.98	7.57E-03	0.0239
TNNI3K	-3.98	7.96E-03	0.0247
TNFSF12	-3.98	1.93E-02	0.0450
AC127459.2	-4.00	8.27E-03	0.0253
AK5	-4.00	2.43E-04	0.0042
AKR1C3	-4.02	3.12E-03	0.0140
AC104964.3	-4.04	1.16E-02	0.0316
AC103810.5	-4.06	8.67E-03	0.0260
AC090948.3	-4.07	9.78E-04	0.0077
AC009690.1	-4.08	1.51E-02	0.0377
TLR1	-4.08	3.80E-03	0.0157
AC246785.2	-4.09	7.73E-03	0.0242
HERC6	-4.10	2.99E-03	0.0137
AC005842.1	-4.10	2.43E-04	0.0042
LINC01801	-4.11	2.72E-03	0.0131
HKDC1	-4.13	2.36E-05	0.0021
C13orf46	-4.15	1.02E-03	0.0078
AC121334.1	-4.16	6.74E-03	0.0223
AL357033.3	-4.17	2.32E-04	0.0042
SPATA41	-4.17	7.81E-03	0.0243
KLF3	-4.17	6.41E-05	0.0028
BTG1P1	-4.18	6.34E-03	0.0214
ENST000003429920	-4.18	4.60E-04	0.0054
AC008555.5	-4.19	4.42E-03	0.0172
AMIGO1	-4.19	1.16E-02	0.0316
LINC02591	-4.21	1.86E-02	0.0438
AKR1D1P1	-4.21	9.82E-03	0.0283
AC008555.1	-4.21	9.91E-05	0.0032
AC244260.1	-4.21	5.43E-04	0.0058
AL645568.2	-4.21	1.57E-03	0.0097
CAPN3	-4.22	1.33E-02	0.0345
LIME1	-4.24	7.43E-05	0.0029
NUTM2D	-4.25	2.09E-02	0.0476
CARD17	-4.26	8.15E-04	0.0070
AL121845.2	-4.31	4.86E-05	0.0026
LINC02390	-4.33	9.41E-04	0.0075
CTSL	-4.37	6.06E-03	0.0208
ENST000003486550	-4.38	1.13E-02	0.0311
ARRDC5	-4.38	2.42E-05	0.0021
STEAP1B	-4.39	5.89E-03	0.0204
SEC22B4P	-4.41	5.88E-03	0.0204
AC022916.1	-4.42	1.30E-04	0.0035
CACNA1C-AS2	-4.43	5.32E-03	0.0192
ADHFE1	-4.44	6.32E-05	0.0028
GOLGA7B	-4.45	5.63E-04	0.0059
AC138894.1	-4.46	5.25E-03	0.0190

RPL7L1P9	-4.47	1.87E-03	0.0105
ENST000003165490	-4.50	3.11E-05	0.0022
LINC01727	-4.51	1.01E-02	0.0287
LDLRAD1	-4.51	1.78E-03	0.0103
AL606491.1	-4.51	1.03E-02	0.0291
AC055748.1	-4.52	3.40E-05	0.0023
AL158210.1	-4.52	1.13E-04	0.0033
TRIB2	-4.55	9.68E-05	0.0031
MUC20-OT1-616	-4.56	1.84E-03	0.0105
ENST000003288233	-4.56	5.60E-03	0.0198
A2M-AS1	-4.56	2.09E-03	0.0112
KLRB1	-4.57	9.89E-04	0.0077
AC015722.1	-4.57	1.72E-03	0.0101
ITGA6	-4.58	7.17E-03	0.0231
ENST000003562060	-4.60	1.82E-03	0.0104
KRT73	-4.61	8.22E-03	0.0252
ENST000003322460	-4.61	6.33E-04	0.0062
CACNA1I	-4.61	1.38E-03	0.0090
DPEP2	-4.64	1.15E-04	0.0033
EDAR	-4.66	1.23E-02	0.0328
AC080162.1	-4.67	1.53E-03	0.0095
ENST000002686761	-4.69	1.80E-02	0.0428
FAM47E-STBD1	-4.74	2.18E-02	0.0491
AC103769.1	-4.74	1.37E-03	0.0089
ST6GALNAC1	-4.76	1.66E-02	0.0404
AC078927.1	-4.76	1.81E-03	0.0104
AL139123.1	-4.78	1.25E-03	0.0086
ICOS	-4.78	1.58E-03	0.0097
TENM1	-4.80	1.67E-02	0.0405
GCOM1	-4.80	2.12E-02	0.0481
AUTS2	-4.88	1.88E-03	0.0106
AC239800.2	-4.89	1.38E-03	0.0090
LINC02478	-4.91	1.06E-03	0.0079
PI16	-4.94	8.60E-03	0.0259
CD200R1	-4.95	1.23E-03	0.0085
ADA2	-4.99	4.23E-05	0.0024
LINC01259	-5.01	3.40E-03	0.0147
LINC01726	-5.02	1.46E-03	0.0093
SORCS3	-5.03	8.66E-04	0.0072
AC025171.4	-5.04	3.77E-03	0.0156
SNED1	-5.07	1.05E-03	0.0079
AC068790.4	-5.09	8.24E-04	0.0070
PLEKHB1	-5.11	3.63E-03	0.0153
Z98200.1	-5.12	8.09E-04	0.0070
RGMB	-5.13	7.64E-04	0.0068
GP6	-5.14	7.71E-04	0.0068
CSGALNACT1	-5.15	7.01E-03	0.0228
AC138932.1	-5.15	7.48E-04	0.0067
ENST000003180413	-5.18	1.33E-03	0.0088
ENST000003158084	-5.19	1.07E-03	0.0080

SAMD3	-5.19	5.99E-04	0.0060
PLXNA4	-5.21	3.06E-04	0.0046
DSC1	-5.21	1.98E-03	0.0109
MMRN1	-5.22	7.48E-04	0.0067
LINC02142	-5.22	1.08E-03	0.0080
SLC22A23	-5.24	5.02E-03	0.0185
RASGRP2	-5.25	7.53E-06	0.0016
ENST000002617260	-5.25	3.17E-03	0.0141
CCDC65	-5.26	1.64E-05	0.0019
ENST000003241940	-5.30	1.57E-03	0.0096
IGFBP3	-5.30	8.88E-04	0.0073
ENST000003417560	-5.36	9.26E-04	0.0075
OCM	-5.45	1.56E-03	0.0096
AC106795.2	-5.45	5.39E-04	0.0058
AC007952.5	-5.45	1.60E-03	0.0098
ENST000000556820	-5.50	4.56E-04	0.0054
RNF157-AS1	-5.54	7.31E-03	0.0234
AC068400.2	-5.54	5.96E-04	0.0060
FBXO39	-5.54	4.14E-04	0.0051
IL7R	-5.54	1.25E-02	0.0332
LINC00861	-5.58	6.76E-06	0.0016
IL17RA	-5.58	8.15E-04	0.0070
ENST000003617380	-5.59	2.24E-03	0.0117
ENST000002632051	-5.59	6.17E-04	0.0061
ENST000002601841	-5.60	4.61E-03	0.0176
ENST000003406600	-5.61	1.38E-03	0.0090
TSPAN18	-5.62	1.10E-04	0.0033
AC139677.1	-5.71	3.69E-04	0.0049
WNT7A	-5.72	1.36E-03	0.0089
ENST000003568871	-5.80	3.20E-04	0.0047
LINC00565	-5.80	4.73E-03	0.0179
ENST000003410121	-5.80	2.75E-04	0.0044
AC107626.1	-5.82	3.45E-04	0.0048
BORCS7-ASMT	-5.85	3.00E-04	0.0046
FGF7	-5.85	1.32E-03	0.0087
ENST000002681502	-5.93	3.78E-04	0.0050
ENST000002646900	-5.94	2.69E-04	0.0044
GZMA	-5.95	7.76E-05	0.0030
TAS2R10	-5.96	2.19E-04	0.0041
CTD1I18.1	-5.96	7.58E-05	0.0030
AC233976.1	-5.97	2.88E-04	0.0045
ENST000003750250	-5.98	2.51E-04	0.0043
AL355581.1	-5.99	3.88E-03	0.0159
BX248123.1	-6.01	2.94E-04	0.0045
AC100827.3	-6.01	2.18E-04	0.0041
ENST000002653753	-6.05	3.95E-04	0.0050
ENST000002157901	-6.17	1.68E-04	0.0038
ENST000003510710	-6.20	1.59E-04	0.0037
COPS8P2	-6.22	2.45E-04	0.0042
AP002365.1	-6.23	2.31E-04	0.0042

ENST000003543211	-6.24	3.97E-03	0.0161
GPA33	-6.32	2.26E-05	0.0020
ENST000002538110	-6.40	4.34E-04	0.0052
ENST000002616061	-6.44	1.45E-04	0.0036
QRICH2	-6.48	1.07E-04	0.0033
RAP1GAP2	-6.50	6.81E-03	0.0224
VSIG1	-6.54	1.89E-05	0.0020
B3GALT2	-6.71	1.17E-04	0.0034
ENST000003275641	-6.86	6.33E-05	0.0028
CCR2	-6.97	6.21E-04	0.0062
ENST000002672571	-7.06	3.16E-04	0.0046
CDKN2B	-7.17	5.37E-04	0.0058
FAM19A1	-7.19	3.92E-04	0.0050
ENST000003599200	-7.48	4.08E-05	0.0024
ARMH2	-7.54	2.93E-05	0.0022
ENST000002925621	-8.20	3.86E-05	0.0023
ENST000002968590	-8.20	1.42E-04	0.0036
KLF2	-8.61	7.14E-04	0.0065
ENST000003256741	-9.09	8.68E-06	0.0016
