# Supplementary material for: Human splice factors contribute to latent HIV infection in primary cell models and blood CD4+ T cells from ART-treated individuals
Source: PLoS Pathog. 2020 Nov 30;16(11):e1009060. doi: 10.1371/journal.ppat.1009060 (PMC7728277; doi:10.1371/journal.ppat.1009060)
Supplement: S3 Table — Genes are ranked by the average log2 fold change (FC). (PDF) [file ppat.1009060.s003.pdf]

Table S3. List of statistically significant differentially expressed genes between unstimulated and stimulated peripheral CD4+ T cells from ART-suppressed individuals. Genes are ranked by the average log2 fold change (FC).

Gene	FC (log2)	p value	FDR
ZBED2	13.31	8.86E-07	0.0008
AP003472.1	10.78	2.04E-05	0.0012
IL9	10.63	1.88E-06	0.0008
CCL3	10.30	1.77E-04	0.0028
LIF	9.96	1.25E-03	0.0073
ENST000003364582	9.85	7.59E-07	0.0008
ENST000003218051	9.71	1.06E-06	0.0008
ENST000003069320	9.61	2.75E-06	0.0008
ENST000003150501	9.30	5.47E-06	0.0009
ENST000003298752	9.19	1.19E-06	0.0008
STC2	9.06	3.11E-06	0.0009
ENST000002533630	9.05	7.02E-06	0.0010
ENST000003435280	9.03	3.24E-06	0.0009
ENST000003237600	8.99	4.08E-06	0.0009
ENST000003388911	8.96	1.48E-05	0.0011
ENST000003083610	8.95	8.95E-06	0.0010
IL1A	8.82	4.82E-06	0.0009
ENST000003566570	8.76	3.99E-06	0.0009
IL21	8.59	2.20E-04	0.0031
GZMB	8.55	1.65E-05	0.0011
ENST000002632741	8.51	1.90E-05	0.0011
ENST000002947321	8.31	1.30E-04	0.0025
ENST000003283540	8.22	1.10E-05	0.0010
FAM72D	8.21	6.90E-04	0.0053
ENST000002616000	8.21	2.87E-03	0.0118
IL24	8.19	8.43E-06	0.0010
ENST000003232744	8.13	3.89E-04	0.0040
IL13	8.10	1.27E-05	0.0010
ENST000003299670	8.09	7.84E-05	0.0020
ENST000002679352	8.09	1.36E-05	0.0011
ENST000002603831	8.05	7.33E-06	0.0010
ENST000003127830	8.04	2.54E-05	0.0013
ENST000003144231	7.97	7.06E-06	0.0010
ENST000003018432	7.95	4.17E-05	0.0016
IL1RN	7.88	1.94E-04	0.0030
ENST000002681381	7.87	8.95E-05	0.0021
CBSL	7.86	1.89E-04	0.0029
EPOP	7.82	1.64E-04	0.0027
C7orf55-LUC7L2	7.79	3.28E-05	0.0015
EGR2	7.77	8.49E-04	0.0059
ENST000002501240	7.77	5.45E-05	0.0017

HSPE1-MOB4	7.76	2.90E-05	0.0014
ENST000003351830	7.76	1.21E-03	0.0072
ENST000003141631	7.72	1.98E-05	0.0012
CCL4	7.70	7.15E-06	0.0010
ENST000002743760	7.65	4.70E-05	0.0016
ENST000003351250	7.64	1.26E-05	0.0010
TNFRSF8	7.63	3.98E-04	0.0040
GPR87	7.62	1.42E-05	0.0011
ENST000003310291	7.60	2.10E-05	0.0012
ENST000003546750	7.60	2.16E-05	0.0012
IL2	7.59	1.26E-06	0.0008
NCS1	7.57	1.74E-05	0.0011
ENST000002233360	7.57	4.75E-04	0.0044
ENST000002848112	7.55	5.18E-03	0.0173
ENST000003506120	7.49	1.52E-05	0.0011
ENST000002637020	7.47	1.50E-05	0.0011
HMSD	7.45	1.59E-05	0.0011
ENST000002568760	7.45	7.29E-07	0.0008
IL17F	7.44	1.76E-04	0.0028
CAVIN3	7.44	1.85E-04	0.0029
CCL20	7.41	7.00E-05	0.0019
DGKI	7.41	3.56E-04	0.0038
ENST000003000272	7.38	4.09E-04	0.0041
ENST000003016341	7.36	4.53E-04	0.0043
FAM72C	7.36	6.00E-04	0.0049
KIF20A	7.35	9.03E-07	0.0008
EIF4EP2	7.34	1.19E-04	0.0024
ENST000002288433	7.34	3.60E-05	0.0015
RF00017.13	7.29	2.14E-04	0.0031
RF00017.14	7.29	2.14E-04	0.0031
RF00017.21	7.29	2.14E-04	0.0031
RF00017.22	7.29	2.14E-04	0.0031
RN7SL471P	7.29	2.14E-04	0.0031
ENST000000004420	7.28	2.11E-04	0.0030
ENST000001776483	7.27	5.56E-05	0.0017
CBS	7.25	1.25E-04	0.0025
CDC45	7.23	7.72E-06	0.0010
ENST000002965950	7.22	5.81E-03	0.0186
GJB2	7.21	2.28E-04	0.0032
ENST00000409111	7.17	2.78E-05	0.0014
LINC02341	7.17	7.05E-07	0.0008
ENST000003405560	7.16	8.01E-05	0.0020
IQGAP3	7.15	5.49E-05	0.0017
HMMR	7.15	1.98E-06	0.0008
SCD	7.13	8.68E-05	0.0021
IFNG	7.13	1.95E-06	0.0008
LPL	7.11	1.37E-04	0.0026

HIST3H2BA	7.10	2.60E-04	0.0033
ENST000003109550	7.07	2.29E-06	0.0008
EME1	7.06	1.22E-05	0.0010
BATF3	7.03	3.63E-05	0.0015
ENST000002503830	7.02	2.99E-05	0.0014
ENST000002275033	6.98	1.80E-04	0.0028
UBE2C	6.96	4.89E-05	0.0017
ENST000003083170	6.95	7.46E-05	0.0020
AC010655.4	6.92	4.51E-05	0.0016
ENST000003004030	6.92	1.37E-06	0.0008
ENST000002613770	6.91	1.99E-03	0.0095
PBK	6.90	6.43E-06	0.0010
DLGAP5	6.85	5.45E-05	0.0017
ENST000003289281	6.84	8.99E-05	0.0022
MYOF	6.83	1.12E-05	0.0010
ENST000003514500	6.78	1.62E-02	0.0377
ENST000002587810	6.76	9.13E-03	0.0252
IL21-AS1	6.75	1.84E-05	0.0011
ENST000002463140	6.74	8.79E-05	0.0021
ENST000002292650	6.70	5.24E-05	0.0017
KIF2C	6.69	3.14E-06	0.0009
ENST000002803523	6.67	9.17E-05	0.0022
ENST000003611620	6.64	1.47E-02	0.0352
CEP55	6.62	7.78E-06	0.0010
ENST000002939681	6.60	2.88E-04	0.0035
CYP1B1	6.57	2.14E-05	0.0012
ENST000003045933	6.54	6.67E-05	0.0019
IL1R2	6.51	5.43E-05	0.0017
ENST000002284250	6.49	1.63E-04	0.0027
ENST000002052140	6.48	8.68E-04	0.0060
AC093512.2	6.47	7.00E-03	0.0211
DHFRP1	6.45	3.60E-03	0.0136
SPR	6.42	7.51E-04	0.0055
ENST000003203891	6.41	2.43E-04	0.0032
ZBTB32	6.40	1.51E-05	0.0011
MCM10	6.40	3.92E-05	0.0016
ENST000003401161	6.38	8.28E-05	0.0021
CALD1	6.37	1.56E-04	0.0027
ENST000003544640	6.37	1.96E-03	0.0094
PKMYT1	6.37	5.59E-05	0.0017
CDCA2	6.36	8.26E-07	0.0008
MAOA	6.36	6.99E-05	0.0019
CCNA2	6.33	9.28E-06	0.0010
GALNT18	6.32	5.49E-04	0.0047
HIST2H3A	6.32	2.28E-06	0.0008
HIST2H3C	6.32	2.28E-06	0.0008
ENST000003242290	6.31	9.63E-05	0.0022

SEMA7A	6.30	8.25E-07	0.0008
CDC25A	6.28	1.48E-05	0.0011
DSCC1	6.28	2.38E-06	0.0008
AP000424.1	6.28	4.37E-04	0.0042
ENST000000090411	6.27	1.25E-02	0.0314
CDCA5	6.27	1.43E-04	0.0026
HIST1H3G	6.26	4.59E-05	0.0016
FAM72B	6.25	2.06E-06	0.0008
POC1B-GALNT4	6.25	3.23E-03	0.0127
PYCR1	6.20	1.15E-04	0.0024
ENST000002920671	6.19	8.58E-03	0.0241
NAPSA	6.19	5.05E-05	0.0017
CCNB1	6.18	3.07E-05	0.0014
MIR155HG	6.18	1.69E-06	0.0008
CDK1	6.17	4.93E-06	0.0009
ENST000002584943	6.17	3.14E-03	0.0125
CHAC1	6.17	1.74E-05	0.0011
AL357992.1	6.16	1.85E-03	0.0091
ENST000003294202	6.16	1.22E-04	0.0025
ENST000002842591	6.15	5.65E-04	0.0048
AURKA	6.14	5.78E-07	0.0008
ENST000003380420	6.14	4.68E-03	0.0162
ENST000002875980	6.13	4.59E-06	0.0009
RRM2	6.13	3.85E-06	0.0009
HILPDA	6.12	6.74E-06	0.0010
ENST000002304800	6.12	1.26E-04	0.0025
ITPKA	6.11	1.98E-04	0.0030
FABP5P7	6.11	4.37E-06	0.0009
IL17A	6.09	7.41E-04	0.0055
EXO1	6.08	1.28E-06	0.0008
DTL	6.07	5.74E-07	0.0008
UHRF1	6.07	1.82E-05	0.0011
ENST000003891460	6.06	3.30E-04	0.0037
CDC6	6.05	4.86E-07	0.0008
ENST000003051882	6.04	1.71E-05	0.0011
GAD1	6.04	4.36E-04	0.0042
ENST000002218560	6.02	1.79E-04	0.0028
CAPSL	6.01	1.51E-04	0.0026
TMPRSS6	6.01	7.42E-04	0.0055
FAM72A	6.00	4.84E-06	0.0009
TEDC2	6.00	6.52E-05	0.0019
MELK	5.98	1.42E-06	0.0008
AL034430.1	5.98	2.80E-03	0.0116
STRIP2	5.97	4.09E-06	0.0009
TTK	5.96	2.47E-06	0.0008
ENST000003484330	5.95	8.86E-04	0.0060
ENST000003285960	5.94	3.39E-03	0.0131

TK1	5.93	2.77E-04	0.0034
AC068547.1	5.92	3.12E-05	0.0014
HPDL	5.90	9.79E-05	0.0022
HIST1H3C	5.89	4.46E-05	0.0016
DOK5	5.89	1.94E-04	0.0030
NDFIP2	5.88	6.54E-07	0.0008
DEPDC1	5.88	3.49E-06	0.0009
ENST000003253851	5.87	2.38E-03	0.0106
CSF2	5.86	9.22E-04	0.0062
ANLN	5.86	1.08E-06	0.0008
TOP2A	5.85	6.88E-07	0.0008
TEAD4	5.85	2.69E-04	0.0034
NCAPG	5.85	6.81E-06	0.0010
VDR	5.85	9.36E-05	0.0022
PTGFRN	5.84	1.45E-05	0.0011
MT3	5.82	7.56E-04	0.0055
MND1	5.81	1.96E-05	0.0012
ENST000003500260	5.81	2.52E-04	0.0033
MYO1B	5.80	7.89E-04	0.0056
KIF15	5.80	3.79E-05	0.0015
ENST000002197820	5.78	2.36E-04	0.0032
KIFC1	5.74	4.75E-06	0.0009
RAD54L	5.74	5.01E-06	0.0009
IFNB1	5.74	3.46E-04	0.0038
LTA	5.73	5.31E-06	0.0009
ENST000002436620	5.72	4.17E-04	0.0041
AK4	5.71	9.07E-06	0.0010
CD68	5.71	1.09E-03	0.0067
ENST000002721980	5.70	6.52E-04	0.0051
ANK2	5.70	3.69E-05	0.0015
ENST000002623153	5.70	4.30E-03	0.0153
UBE2T	5.68	9.65E-06	0.0010
ENST000003617450	5.67	3.87E-04	0.0040
ENST000002908811	5.67	4.03E-04	0.0040
CCL17	5.66	3.10E-04	0.0036
ENST000003222690	5.65	3.03E-04	0.0036
ENST000002633600	5.64	4.34E-05	0.0016
G0S2	5.63	2.15E-05	0.0012
DNAJC12	5.63	3.07E-05	0.0014
ENST000003228861	5.61	5.98E-03	0.0190
GCSHP5	5.61	2.00E-03	0.0095
HIST1H3B	5.61	2.93E-05	0.0014
CENPA	5.60	4.06E-05	0.0016
BIRC5	5.60	1.45E-04	0.0026
ENST000003062563	5.59	3.19E-04	0.0037
ENST000003195182	5.59	3.02E-04	0.0036
ZNF823	5.58	7.51E-04	0.0055

SLC6A9	5.58	5.46E-04	0.0046
LHFPL6	5.57	2.32E-03	0.0104
IER3	5.57	9.76E-06	0.0010
MYBL2	5.56	2.77E-05	0.0014
CTTN	5.55	5.03E-04	0.0045
DHCR24	5.55	5.80E-04	0.0048
ENST000002938722	5.54	6.92E-04	0.0053
ENST000002500562	5.54	7.24E-04	0.0054
CDKN3	5.54	5.61E-05	0.0017
SLCO4A1	5.54	7.05E-05	0.0019
C17orf58	5.53	2.47E-04	0.0032
HASPIN	5.53	7.37E-04	0.0055
CCNB2	5.53	3.59E-06	0.0009
AC112721.1	5.52	1.47E-03	0.0080
ENST000003386310	5.52	5.64E-03	0.0183
ENST00000629913	5.51	3.95E-04	0.0040
IL4I1	5.51	8.74E-06	0.0010
CDCA8	5.51	6.92E-06	0.0010
MKI67	5.50	6.91E-06	0.0010
ENST000003024500	5.50	2.99E-06	0.0009
TRIP13	5.50	1.93E-04	0.0030
ENST000002988380	5.50	3.59E-04	0.0038
ENST000002590431	5.50	2.01E-04	0.0030
ENST000003183642	5.48	4.88E-04	0.0044
PSRC1	5.47	8.81E-06	0.0010
HIST1H3F	5.47	1.98E-06	0.0008
ARG2	5.46	2.07E-05	0.0012
POLQ	5.46	1.32E-05	0.0010
RIBC2	5.45	4.09E-05	0.0016
KIF23	5.45	7.34E-06	0.0010
AURKB	5.45	2.48E-06	0.0008
ASS1	5.43	2.44E-04	0.0032
ENST000003378811	5.42	3.87E-04	0.0040
HIST1H4I	5.42	2.12E-06	0.0008
KIF4A	5.42	8.75E-05	0.0021
ENST000003158774	5.42	4.13E-04	0.0041
RHEBL1	5.42	9.57E-03	0.0260
AIM2	5.41	3.24E-06	0.0009
CKAP2L	5.41	4.30E-05	0.0016
PSMC1P1	5.41	1.94E-03	0.0094
ENST000002915351	5.41	5.35E-04	0.0046
ENST000002594602	5.40	1.05E-02	0.0276
HJURP	5.40	2.76E-06	0.0008
FNDC9	5.39	4.02E-04	0.0040
ENST000003285141	5.39	2.73E-03	0.0115
DHRS9	5.39	2.41E-03	0.0107
INSM1	5.38	4.29E-04	0.0041

SLC25A10	5.36	6.07E-03	0.0192
KIF14	5.36	3.03E-05	0.0014
AC009005.1	5.36	4.22E-04	0.0041
NEK2	5.36	1.30E-06	0.0008
KIF24	5.35	2.09E-05	0.0012
HIST1H2AL	5.35	2.10E-05	0.0012
ASPM	5.34	3.68E-05	0.0015
ERCC6L	5.33	1.82E-06	0.0008
ENST000002500920	5.32	7.07E-06	0.0010
XIRP1	5.31	8.01E-04	0.0057
SLC29A1	5.31	3.99E-05	0.0016
ENST000002636461	5.30	5.16E-04	0.0045
PPFIA4	5.30	2.49E-05	0.0013
GINS2	5.30	1.90E-04	0.0029
ZNF697	5.30	1.45E-03	0.0080
ENST000003512880	5.30	1.61E-02	0.0375
RAD51	5.29	2.70E-06	0.0008
ENST000003265871	5.28	1.07E-02	0.0281
ASF1B	5.28	2.17E-05	0.0012
ENST000002756351	5.27	6.39E-04	0.0051
MFSD2A	5.27	2.01E-04	0.0030
CFAP58	5.27	1.39E-03	0.0078
ENST000002793871	5.26	1.79E-02	0.0406
ENST000003027590	5.26	5.53E-05	0.0017
SKA3	5.24	3.96E-06	0.0009
ENST000003132852	5.23	1.97E-06	0.0008
PCDH12	5.23	3.49E-05	0.0015
AL133215.2	5.23	5.34E-04	0.0046
SLC27A2	5.22	9.00E-06	0.0010
PFKFB4	5.22	4.71E-05	0.0016
ENST000002459341	5.22	7.89E-06	0.0010
ENST000002490660	5.22	5.57E-04	0.0047
AC112777.1	5.19	1.70E-03	0.0087
SLC43A3	5.18	1.62E-06	0.0008
IL5	5.18	7.14E-03	0.0213
ENST000003196220	5.17	6.14E-04	0.0050
EPAS1	5.17	9.06E-06	0.0010
ENST000002650561	5.16	2.06E-06	0.0008
ATF3	5.15	6.71E-04	0.0052
KIF18B	5.14	2.07E-03	0.0097
CCDC74B	5.14	7.64E-04	0.0056
DIAPH3	5.14	2.34E-06	0.0008
ENST000001894440	5.13	1.30E-05	0.0010
BRCA1	5.12	1.16E-06	0.0008
ENST000003112751	5.12	9.12E-03	0.0252
CDC25C	5.12	5.26E-06	0.0009
AC007283.1	5.12	1.25E-03	0.0073

AIF1L	5.11	7.20E-04	0.0054
ENST000002632680	5.10	1.03E-03	0.0066
AUNIP	5.10	1.78E-04	0.0028
ENST000002045170	5.09	2.02E-02	0.0442
PSAT1	5.09	1.51E-06	0.0008
ENST000003442790	5.09	7.08E-04	0.0054
VEGFA	5.09	8.77E-06	0.0010
CKS2	5.09	7.40E-06	0.0010
LAMB3	5.08	1.26E-03	0.0073
MCM2	5.08	2.65E-06	0.0008
ENST000003048341	5.08	6.88E-04	0.0053
AK4P1	5.07	9.27E-03	0.0254
DEPDC1B	5.07	2.34E-06	0.0008
PAQR4	5.07	4.23E-03	0.0152
ENST000002850391	5.05	1.56E-03	0.0083
ENST000002541930	5.05	4.12E-05	0.0016
ENST000002603590	5.05	1.05E-05	0.0010
ENST000003568360	5.05	7.35E-04	0.0055
DDIAS	5.04	9.83E-06	0.0010
ADM	5.04	5.56E-04	0.0047
SDC4	5.04	1.35E-05	0.0011
NAMPTP1	5.04	1.95E-03	0.0094
TYMS	5.03	4.95E-06	0.0009
IGSF3	5.03	3.78E-04	0.0039
PTMS	5.03	2.15E-04	0.0031
RAD51AP1	5.03	2.36E-06	0.0008
ENST000002790361	5.03	1.25E-02	0.0313
CENPW	5.03	1.35E-04	0.0025
POC1A	5.02	1.46E-05	0.0011
CLSPN	5.01	8.49E-06	0.0010
HIST1H2AJ	5.00	5.98E-05	0.0018
KCNK5	4.98	4.09E-06	0.0009
HIST1H2BJ	4.98	1.96E-05	0.0012
NUF2	4.98	1.12E-06	0.0008
ENST000002699320	4.97	5.58E-03	0.0181
MPZL2	4.97	7.54E-06	0.0010
HSD11B1	4.96	4.64E-05	0.0016
HIST1H2AI	4.96	1.17E-05	0.0010
PKN3	4.96	2.03E-03	0.0096
IGF2	4.95	8.80E-04	0.0060
ATP5MF-PTCD1	4.94	8.24E-04	0.0058
AP001453.2	4.94	8.84E-04	0.0060
EBI3	4.93	1.06E-03	0.0066
ENST000003411540	4.93	5.50E-03	0.0179
ECSCR	4.93	2.74E-03	0.0115
UBD	4.92	8.77E-04	0.0060
UNQ6494	4.92	3.13E-03	0.0125

E2F1	4.92	2.27E-06	0.0008
ENST000003556360	4.91	1.20E-03	0.0071
ENST000003588670	4.91	2.68E-03	0.0114
CXCL9	4.90	1.04E-04	0.0023
AC007207.1	4.90	9.25E-04	0.0062
ENST000002621245	4.90	2.91E-04	0.0035
SGO1	4.90	1.45E-05	0.0011
SLC7A11	4.89	3.58E-06	0.0009
ENST000002503792	4.88	1.07E-02	0.0280
P4HA2	4.88	1.27E-04	0.0025
CENPS-CORT	4.88	4.87E-03	0.0166
IRF4	4.87	8.87E-07	0.0008
PDXP	4.86	7.81E-04	0.0056
ENST000002880871	4.86	1.17E-03	0.0070
IFIT2	4.85	4.83E-06	0.0009
ORC1	4.85	2.73E-06	0.0008
SLC1A5	4.84	4.34E-06	0.0009
ENST000003023620	4.83	5.37E-05	0.0017
NUSAP1	4.82	3.99E-05	0.0016
IL22	4.82	1.28E-03	0.0074
B3GNT5	4.82	4.15E-04	0.0041
ENST000003332560	4.81	2.38E-02	0.0498
ATP2A1	4.81	1.25E-03	0.0073
NRM	4.81	7.75E-03	0.0226
SPAG5	4.78	1.51E-06	0.0008
LRP2	4.77	3.69E-04	0.0039
ESPL1	4.77	2.56E-05	0.0013
KRT7	4.77	1.37E-03	0.0077
HIST2H4A	4.76	1.05E-06	0.0008
HIST1H3J	4.76	1.58E-05	0.0011
S100A2	4.74	1.07E-03	0.0067
RNU5A-8P	4.73	7.83E-03	0.0227
KDELR3	4.72	2.83E-03	0.0117
PHGDH	4.72	1.76E-05	0.0011
ALDH1L2	4.72	1.78E-03	0.0090
RMI2	4.71	1.39E-04	0.0026
ENST000002747471	4.71	1.29E-03	0.0074
TNFRSF12A	4.71	8.64E-04	0.0059
FAM71B	4.70	2.75E-04	0.0034
ARHGAP11A	4.70	1.61E-06	0.0008
ENST000002335052	4.70	1.29E-03	0.0074
ENST000002572610	4.69	9.77E-03	0.0263
CD200	4.69	3.87E-04	0.0040
ENST000002611921	4.69	8.38E-05	0.0021
ENST000003176150	4.69	3.40E-03	0.0131
SHCBP1	4.68	1.22E-05	0.0010
AC112721.2	4.68	2.57E-05	0.0013

WARS	4.66	1.02E-05	0.0010
ENST000002161902	4.66	2.15E-03	0.0099
HIST1H1B	4.65	1.47E-06	0.0008
KIF11	4.65	4.58E-06	0.0009
CKS1B	4.65	1.07E-05	0.0010
PRG4	4.65	6.77E-04	0.0052
ENST000002972580	4.64	2.22E-05	0.0012
ZWINT	4.63	4.34E-05	0.0016
CIT	4.63	3.75E-06	0.0009
MKX	4.62	1.50E-03	0.0081
HIST1H2BL	4.61	2.59E-06	0.0008
CENPF	4.61	1.26E-05	0.0010
FBXO43	4.61	3.19E-04	0.0037
OIP5	4.60	1.13E-05	0.0010
CDC20	4.59	7.93E-04	0.0057
OSCP1	4.59	1.08E-02	0.0283
ENST000002373051	4.58	2.02E-02	0.0442
HIST1H2AH	4.58	3.66E-05	0.0015
EIF4EBP1	4.57	1.16E-05	0.0010
NUDT8	4.57	4.49E-04	0.0042
HIST1H2BM	4.57	7.35E-06	0.0010
ENST000003218980	4.57	7.46E-03	0.0220
CDCA3	4.56	7.85E-06	0.0010
TNF	4.56	5.26E-05	0.0017
SH3D21	4.54	1.04E-05	0.0010
ZNF334	4.53	1.88E-03	0.0092
ENST000002605990	4.52	1.48E-02	0.0353
C17orf53	4.52	6.35E-05	0.0019
PSMC3IP	4.52	4.34E-05	0.0016
C6orf223	4.51	5.44E-03	0.0178
RECQL4	4.51	1.63E-04	0.0027
AC005324.4	4.51	1.79E-03	0.0090
AL021578.1	4.51	1.79E-03	0.0090
SGO2	4.51	1.81E-05	0.0011
ANGPTL6	4.50	2.25E-05	0.0012
DYRK3	4.50	1.90E-05	0.0011
EMP1	4.50	1.47E-04	0.0026
MCM4	4.47	4.77E-06	0.0009
CENPU	4.47	2.43E-06	0.0008
IL26	4.47	2.37E-03	0.0106
E2F8	4.46	8.81E-06	0.0010
ENST000002186521	4.45	9.86E-06	0.0010
IL1R1	4.44	6.75E-04	0.0052
ENST000003509971	4.44	7.72E-04	0.0056
HIST2H2AA3	4.43	1.56E-05	0.0011
AC004069.1	4.43	1.72E-03	0.0088
SAPCD2	4.42	6.99E-04	0.0053

TNFRSF18	4.39	6.16E-06	0.0010
AP000781.2	4.39	4.25E-05	0.0016
DPCD	4.38	7.38E-05	0.0020
COL6A3	4.38	4.47E-06	0.0009
MYH10	4.38	1.47E-04	0.0026
TRIB3	4.38	3.72E-04	0.0039
CHEK1	4.38	1.78E-06	0.0008
ZACN	4.37	5.62E-03	0.0182
NAMPT	4.36	2.00E-05	0.0012
SMC1B	4.36	1.01E-04	0.0022
AP003472.2	4.36	3.51E-03	0.0134
GK	4.36	4.65E-06	0.0009
ENST000003107751	4.35	2.04E-05	0.0012
CENPE	4.34	3.99E-06	0.0009
HIST2H3D	4.34	5.06E-06	0.0009
KNL1	4.33	2.26E-06	0.0008
BAG6-427	4.33	5.24E-03	0.0174
MAK	4.33	3.85E-04	0.0039
H2AFX	4.32	3.71E-05	0.0015
CLIC4	4.32	2.61E-04	0.0033
ENST000002656020	4.31	4.75E-03	0.0163
HIST2H2AA4	4.31	4.40E-05	0.0016
UCK2	4.30	2.07E-05	0.0012
LINC00158	4.30	1.40E-04	0.0026
ENST000002994980	4.29	3.11E-03	0.0124
IL23R	4.29	2.29E-04	0.0032
TUBA1B	4.29	4.30E-05	0.0016
IFIT3	4.28	3.46E-06	0.0009
FLT1	4.28	1.08E-05	0.0010
SORD	4.28	1.47E-02	0.0352
ENST000003045141	4.28	2.24E-02	0.0477
NCAPH	4.28	8.43E-06	0.0010
SKA1	4.28	8.07E-06	0.0010
PGAM1	4.27	8.00E-06	0.0010
KIF18A	4.27	5.07E-06	0.0009
POLE2	4.27	1.11E-05	0.0010
ENST000002114020	4.26	1.06E-03	0.0066
BUB1B	4.26	1.51E-05	0.0011
HIST1H2BO	4.26	4.98E-06	0.0009
HOPX	4.25	2.24E-05	0.0012
TNFRSF9	4.25	2.87E-06	0.0009
TMPRSS3	4.25	1.83E-03	0.0091
SPC25	4.24	1.03E-05	0.0010
CENPI	4.24	1.89E-05	0.0011
ENST00000567345	4.24	3.69E-05	0.0015
AC017002.3	4.24	4.94E-04	0.0044
PCNA	4.24	1.22E-05	0.0010

WDHD1	4.24	2.28E-06	0.0008
LINC01943	4.24	7.80E-04	0.0056
FAM57A	4.23	3.02E-03	0.0122
TWIST1	4.22	2.32E-02	0.0488
IL12RB2	4.22	7.99E-06	0.0010
MTHFD2	4.21	4.25E-06	0.0009
NME1-NME2	4.21	6.34E-05	0.0019
ENST000003408481	4.21	2.38E-04	0.0032
APOBEC3B	4.20	7.74E-03	0.0226
TROAP	4.20	7.97E-05	0.0020
PTTG1	4.20	1.50E-05	0.0011
AC096920.1	4.20	5.17E-03	0.0172
ENST000002155871	4.20	1.25E-05	0.0010
CAMK1	4.20	1.98E-05	0.0012
STMN1	4.19	2.34E-05	0.0012
DBN1	4.19	4.05E-05	0.0016
ENST000003183880	4.19	2.63E-03	0.0113
SDCBP2	4.18	2.42E-04	0.0032
EGR1	4.18	1.24E-03	0.0073
RBBP8	4.17	8.08E-06	0.0010
HIP1	4.17	1.17E-02	0.0299
WDR34	4.17	8.19E-04	0.0058
RGS16	4.17	6.92E-05	0.0019
NCAPG2	4.15	9.53E-06	0.0010
DMC1	4.15	3.49E-04	0.0038
AC126696.3	4.13	2.71E-03	0.0114
THBS4	4.13	7.09E-04	0.0054
AC008147.4	4.12	2.98E-03	0.0121
HIST1H2BH	4.11	1.40E-05	0.0011
PTPRF	4.11	1.48E-02	0.0354
BATF	4.10	4.31E-05	0.0016
ENST000003616110	4.10	2.97E-05	0.0014
SMTN	4.09	3.43E-04	0.0038
DUSP6	4.09	3.24E-04	0.0037
TUBB	4.08	7.78E-06	0.0010
B4GALNT1	4.08	4.59E-03	0.0159
GOLM1	4.08	7.58E-04	0.0055
BYSL	4.07	8.45E-06	0.0010
TNFRSF4	4.07	4.06E-05	0.0016
AC009159.2	4.07	4.47E-03	0.0156
ENST000003243662	4.06	7.08E-04	0.0054
IL2RA	4.05	3.84E-06	0.0009
NPM3	4.05	3.94E-06	0.0009
AC073896.1	4.03	2.08E-02	0.0452
AC012073.1	4.03	6.27E-03	0.0195
ENST000003569480	4.03	1.81E-04	0.0029
SNORA21B	4.02	2.22E-02	0.0473

CYP27B1	4.02	3.63E-05	0.0015
MTFR2	4.01	4.53E-05	0.0016
DNAJB5	4.01	2.46E-05	0.0013
GPR84	4.01	3.28E-03	0.0128
NMRAL2P	4.01	4.01E-03	0.0146
LINC01281	4.00	1.00E-05	0.0010
HIST1H4L	3.99	3.96E-04	0.0040
TUBA1C	3.99	5.78E-05	0.0018
AC007240.1	3.99	9.69E-06	0.0010
KNSTRN	3.98	8.66E-06	0.0010
EGR3	3.98	6.00E-04	0.0049
SNORA74D	3.98	2.95E-05	0.0014
IGFBP6	3.97	2.58E-03	0.0111
ENST000003262323	3.97	7.08E-04	0.0054
LINC01229	3.96	1.72E-02	0.0394
TAGLN3	3.95	3.66E-03	0.0138
NAB2	3.95	1.57E-05	0.0011
MTHFD1L	3.94	5.13E-04	0.0045
CENPH	3.94	5.53E-06	0.0009
FKBPL	3.94	1.80E-04	0.0029
ENST000002388232	3.93	3.87E-05	0.0016
HK2	3.93	1.11E-05	0.0010
PARD3	3.93	1.57E-03	0.0083
SPSB1	3.92	7.59E-03	0.0222
AC010655.2	3.92	1.11E-02	0.0289
GAPDH	3.92	2.33E-06	0.0008
PLTP	3.91	5.51E-03	0.0180
ENST000003549190	3.91	5.04E-05	0.0017
TUBB6	3.90	3.07E-04	0.0036
LINC01480	3.90	2.18E-05	0.0012
CU639417.1	3.89	3.74E-05	0.0015
H2BFS	3.89	3.74E-05	0.0015
GINS1	3.89	1.29E-05	0.0010
PAICS	3.89	5.12E-06	0.0009
AL360181.3	3.89	2.35E-02	0.0493
OASL	3.89	1.25E-04	0.0025
CXCL10	3.89	2.38E-03	0.0106
CENPN	3.89	8.81E-06	0.0010
TNFSF14	3.89	3.11E-04	0.0036
TCF19	3.89	2.97E-05	0.0014
CCDC150	3.88	1.53E-05	0.0011
AC004466.2	3.88	6.05E-04	0.0049
CHAC2	3.88	8.48E-05	0.0021
HIST1H2BK	3.87	4.52E-06	0.0009
RACGAP1	3.87	1.58E-05	0.0011
PRC1	3.86	4.10E-06	0.0009
NPTX1	3.86	1.31E-04	0.0025

ILDR2	3.86	1.96E-04	0.0030
NHS	3.86	1.76E-03	0.0089
CCNF	3.85	1.08E-05	0.0010
AL031846.1	3.85	1.12E-02	0.0291
HIST1H3H	3.85	7.58E-06	0.0010
TPI1	3.84	9.12E-06	0.0010
ENST000003304300	3.84	2.10E-02	0.0454
CIP2A	3.84	1.38E-05	0.0011
SHMT2	3.83	4.35E-06	0.0009
ENST000002595230	3.83	8.91E-03	0.0248
CTNNAL1	3.83	5.36E-05	0.0017
CD109	3.82	1.06E-05	0.0010
ROCK1P1	3.82	6.08E-04	0.0049
ENST000003411810	3.82	5.33E-06	0.0009
AC006329.1	3.81	1.24E-03	0.0073
CDC7	3.81	8.11E-03	0.0233
ENST000002863981	3.81	2.96E-04	0.0035
FURIN	3.81	1.36E-03	0.0076
SPAG1	3.80	1.29E-05	0.0010
ENST000002703011	3.80	1.32E-04	0.0025
AL109918.1	3.80	3.11E-03	0.0124
TTC25	3.80	7.21E-04	0.0054
DHFR	3.80	7.20E-06	0.0010
PROB1	3.79	2.01E-04	0.0030
HMGB3	3.79	1.64E-04	0.0028
FEN1	3.79	1.16E-05	0.0010
HSPA1B	3.78	3.58E-05	0.0015
HS6ST2	3.78	5.60E-03	0.0182
B4GALT2	3.78	4.12E-05	0.0016
HIST1H2AB	3.77	8.24E-06	0.0010
MCM7	3.77	6.76E-06	0.0010
AC112496.1	3.76	3.65E-04	0.0039
WASF1	3.76	1.53E-02	0.0362
ENST000003157112	3.76	4.78E-05	0.0016
C2orf48	3.76	2.07E-04	0.0030
TRPC3	3.75	1.23E-03	0.0073
LINC01132	3.75	7.12E-05	0.0019
HIST1H2BE	3.74	8.80E-06	0.0010
C4orf47	3.74	3.79E-03	0.0141
AL162231.1	3.74	9.91E-03	0.0266
ENST000002795501	3.73	3.23E-05	0.0014
ENST000002214130	3.73	1.65E-04	0.0028
HIST1H2BB	3.73	1.19E-04	0.0024
RPL39L	3.72	1.15E-04	0.0024
SNRNP25	3.72	1.30E-04	0.0025
ENST000003423860	3.72	1.16E-05	0.0010
AGRN	3.72	5.23E-04	0.0045

HIST1H4D	3.72	5.49E-06	0.0009
TUBG1	3.72	1.32E-04	0.0025
NME1	3.71	5.30E-05	0.0017
CGREF1	3.71	1.51E-04	0.0026
SPHK1	3.71	6.51E-05	0.0019
PRR11	3.71	3.96E-05	0.0016
CDT1	3.70	6.74E-05	0.0019
ENST000003615720	3.70	9.05E-05	0.0022
ICAM1	3.70	9.39E-05	0.0022
ENST000002913580	3.70	2.02E-03	0.0096
ENO1	3.70	6.84E-06	0.0010
TMEM97	3.69	8.53E-05	0.0021
AC051619.8	3.69	1.67E-04	0.0028
GGH	3.68	4.94E-05	0.0017
ECT2	3.68	4.85E-06	0.0009
STIL	3.68	3.93E-05	0.0016
ENST000002298541	3.68	8.17E-06	0.0010
PLK4	3.68	4.20E-06	0.0009
ANKRD13B	3.67	2.76E-04	0.0034
SEMA4A	3.67	1.45E-03	0.0079
BUB1	3.67	7.79E-05	0.0020
ENST000002860310	3.66	3.09E-04	0.0036
TIPIN	3.66	2.24E-05	0.0012
ENST000003286970	3.66	1.42E-04	0.0026
GSTA4	3.66	3.61E-05	0.0015
TST	3.65	2.15E-03	0.0099
PCLAF	3.65	2.06E-05	0.0012
C1orf74	3.65	4.52E-05	0.0016
HIST2H2BF	3.65	8.17E-06	0.0010
PHKA1	3.64	4.30E-04	0.0041
KCNK1	3.64	1.27E-02	0.0318
COLGALT2	3.64	1.13E-04	0.0024
C19orf57	3.64	1.93E-04	0.0029
HIST1H2BF	3.64	7.29E-06	0.0010
MAD2L1	3.64	5.85E-06	0.0009
MIR210HG	3.64	2.20E-04	0.0031
ASB2	3.63	2.07E-02	0.0450
CDK2AP1	3.63	1.13E-05	0.0010
ARNTL2	3.63	1.01E-05	0.0010
AHCY	3.62	8.49E-06	0.0010
TERT	3.61	2.53E-03	0.0110
TRIP10	3.61	2.47E-04	0.0032
EVI5	3.61	1.62E-03	0.0085
ENST000002957462	3.60	2.34E-05	0.0012
TRAIP	3.60	5.24E-05	0.0017
TIMELESS	3.60	7.38E-06	0.0010
BLM	3.60	2.00E-05	0.0012

GPT2	3.60	3.97E-05	0.0016
ENST000002166052	3.59	2.79E-05	0.0014
RCC1	3.59	5.22E-05	0.0017
ENST000003352091	3.59	2.81E-04	0.0035
PCK2	3.59	1.58E-05	0.0011
HRH1	3.58	6.01E-03	0.0190
ENST000003160773	3.58	2.16E-05	0.0012
DIXDC1	3.58	1.11E-03	0.0068
EML2-AS1	3.57	2.77E-04	0.0034
HIST1H4J	3.57	4.44E-06	0.0009
CDCA7	3.57	3.23E-05	0.0014
AP001453.3	3.57	1.79E-03	0.0090
PLK1	3.57	3.21E-04	0.0037
RPL38P4	3.56	6.79E-03	0.0206
FERMT2	3.55	1.11E-04	0.0023
CISH	3.54	2.31E-05	0.0012
ZCCHC12	3.53	2.18E-02	0.0467
AL390719.1	3.53	8.59E-05	0.0021
HSD11B1-AS1	3.53	2.85E-04	0.0035
AC099552.1	3.53	1.28E-02	0.0318
GALK1	3.52	3.07E-04	0.0036
EXOSC4	3.52	2.92E-04	0.0035
SQLE	3.51	1.38E-05	0.0011
TXN	3.51	9.65E-06	0.0010
RN7SL3	3.51	2.21E-04	0.0031
NEURL3	3.50	1.82E-03	0.0091
GNA15	3.49	8.50E-04	0.0059
FANCA	3.49	1.48E-05	0.0011
CENPM	3.49	5.60E-05	0.0017
ENST000002579040	3.48	2.29E-05	0.0012
GMNN	3.48	1.63E-05	0.0011
GTSE1	3.48	5.43E-05	0.0017
NTRK2	3.48	7.06E-03	0.0212
C5orf34	3.48	2.20E-05	0.0012
MERTK	3.47	9.96E-04	0.0064
LDHA	3.47	6.36E-06	0.0010
FANCI	3.46	3.69E-05	0.0015
PDCD1	3.46	1.55E-04	0.0027
ENST000003253071	3.46	7.32E-05	0.0020
UBE2S	3.45	3.76E-05	0.0015
SESN2	3.44	3.39E-05	0.0015
SLC7A5	3.43	1.48E-04	0.0026
NT5DC2	3.43	1.20E-04	0.0024
BRCA2	3.43	4.24E-05	0.0016
RAB33A	3.43	3.26E-05	0.0014
QPCT	3.43	2.77E-03	0.0116
SPC24	3.43	1.80E-05	0.0011

RDH10	3.42	1.16E-05	0.0010
ESCO2	3.42	2.17E-04	0.0031
CTH	3.41	1.04E-04	0.0023
FTH1P22	3.41	1.36E-03	0.0076
LAG3	3.41	1.02E-03	0.0065
HIST1H2AD	3.41	5.79E-06	0.0009
ARHGEF25	3.40	1.51E-03	0.0081
ENST000003197881	3.40	1.49E-02	0.0356
DNA2	3.40	1.78E-05	0.0011
HIST1H2AM	3.40	1.79E-05	0.0011
AC005943.1	3.40	2.06E-04	0.0030
ENST000003175510	3.40	3.99E-04	0.0040
ENST000003010680	3.40	6.21E-03	0.0194
RPUSD2	3.39	2.47E-04	0.0032
RFC2	3.39	1.52E-05	0.0011
SERPINE2	3.39	3.03E-03	0.0122
ENST000003545990	3.38	6.22E-03	0.0194
ATAD5	3.38	7.54E-06	0.0010
ENST000002679502	3.38	1.08E-04	0.0023
ENST000002331142	3.37	5.62E-05	0.0017
PPP4R4	3.37	7.10E-05	0.0019
PDLIM7	3.36	1.49E-03	0.0081
LMNA	3.36	5.00E-04	0.0045
VDAC1	3.35	9.91E-06	0.0010
SCCPDH	3.34	1.72E-04	0.0028
HIST1H2AG	3.34	6.30E-06	0.0010
AC108879.1	3.34	2.11E-03	0.0098
GOT1	3.34	8.80E-06	0.0010
HIST1H1A	3.34	3.24E-04	0.0037
PPP1R14B	3.34	8.06E-05	0.0020
RFC4	3.33	2.21E-05	0.0012
LMNB1	3.33	9.26E-06	0.0010
ENST000002965090	3.33	5.63E-05	0.0017
ENST000002617722	3.32	2.82E-05	0.0014
CORO6	3.30	5.47E-05	0.0017
MRPL17	3.30	4.69E-05	0.0016
FAH	3.30	1.02E-04	0.0023
NEFH	3.30	3.77E-04	0.0039
TOMM34	3.29	6.72E-05	0.0019
PLAGL2	3.29	1.16E-04	0.0024
HIST1H4F	3.29	5.82E-06	0.0009
FAM131B	3.28	1.65E-03	0.0086
LONP1	3.28	1.41E-05	0.0011
THOP1	3.28	5.33E-05	0.0017
CCDC51	3.27	1.38E-04	0.0026
KL	3.27	5.46E-03	0.0179
ACOT7	3.26	4.58E-05	0.0016

PKM	3.26	2.40E-05	0.0012
WDR76	3.26	1.39E-05	0.0011
MYL6B	3.26	7.81E-05	0.0020
POLR3G	3.26	5.38E-05	0.0017
GIHCG	3.26	4.12E-05	0.0016
DTYMK	3.26	6.55E-05	0.0019
GINS3	3.25	1.81E-04	0.0029
ERI2	3.25	2.72E-05	0.0013
ENST000003048743	3.25	4.66E-03	0.0161
SLC35G2	3.25	7.74E-04	0.0056
AC147651.3	3.25	3.20E-05	0.0014
AL591806.3	3.25	8.77E-04	0.0060
DARS2	3.25	8.93E-06	0.0010
RMI1	3.24	2.47E-05	0.0013
ENST000002400790	3.24	4.69E-05	0.0016
ENST000003131150	3.24	1.45E-04	0.0026
AL391832.3	3.24	5.70E-04	0.0048
SLC39A14	3.23	2.59E-05	0.0013
POLD1	3.23	2.24E-05	0.0012
ENST000002528160	3.23	1.71E-05	0.0011
DCTPP1	3.23	1.39E-04	0.0026
TPI1P1	3.23	2.85E-03	0.0118
CCNE2	3.23	4.04E-04	0.0040
ENST000002645523	3.22	3.00E-05	0.0014
ENST000002943041	3.22	4.12E-04	0.0041
ZNF593	3.22	1.77E-04	0.0028
HMGCS1	3.22	5.63E-05	0.0017
H2AFZ	3.22	1.64E-05	0.0011
KCNC3	3.22	3.19E-03	0.0126
C3orf14	3.21	7.48E-05	0.0020
PVR	3.21	7.99E-05	0.0020
N4BP3	3.21	1.34E-03	0.0076
STIP1	3.20	3.62E-05	0.0015
TTC23	3.20	1.01E-04	0.0022
GPN3	3.20	3.19E-05	0.0014
AC092718.4	3.20	2.70E-04	0.0034
CA2	3.19	2.06E-03	0.0097
CD27-AS1	3.19	7.81E-05	0.0020
ARHGEF39	3.19	1.08E-04	0.0023
SLC22A4	3.18	2.09E-03	0.0098
PIF1	3.18	1.39E-04	0.0026
AL590399.1	3.18	8.95E-04	0.0061
RANBP1	3.18	6.24E-05	0.0018
SMC2	3.18	1.05E-05	0.0010
MCM6	3.18	1.36E-05	0.0011
ENST000002716361	3.18	2.18E-03	0.0100
HIST1H4H	3.17	1.32E-05	0.0010

ENST000003322110	3.17	1.47E-05	0.0011
ENST000003574290	3.17	8.99E-04	0.0061
HSPD1	3.16	3.87E-05	0.0016
VLDLR	3.16	7.08E-04	0.0054
HIST1H3I	3.16	7.91E-05	0.0020
GSTO1	3.16	1.80E-05	0.0011
NEIL3	3.16	7.55E-05	0.0020
HIST1H4C	3.15	8.30E-06	0.0010
GNGT2	3.14	1.73E-04	0.0028
ENST000003296080	3.14	1.06E-04	0.0023
NFIL3	3.14	3.47E-03	0.0133
TNFSF11	3.14	1.36E-04	0.0025
ENST000002808920	3.13	1.13E-03	0.0069
SPDL1	3.13	3.77E-05	0.0015
DCLRE1B	3.13	3.72E-05	0.0015
UACA	3.13	2.69E-04	0.0034
INCENP	3.13	1.88E-05	0.0011
PALLD	3.13	2.39E-03	0.0106
AMACR	3.12	8.99E-05	0.0022
BLVRB	3.12	4.37E-05	0.0016
CDK2	3.12	1.11E-05	0.0010
STX1A	3.12	9.21E-05	0.0022
DGUOK-AS1	3.11	6.69E-03	0.0204
SHF	3.11	1.71E-04	0.0028
ENST000002603241	3.10	4.41E-03	0.0155
ZBED9	3.10	9.59E-04	0.0063
ENST000002615070	3.10	4.39E-04	0.0042
NCAPD2	3.10	2.32E-05	0.0012
CHRNA6	3.10	1.53E-03	0.0082
BCL2L1	3.10	3.36E-05	0.0015
CENPO	3.09	1.48E-04	0.0026
HMGA1	3.09	2.54E-04	0.0033
MIF	3.09	5.56E-05	0.0017
SLC41A2	3.09	2.13E-05	0.0012
AL445524.1	3.09	3.56E-04	0.0038
ANKRD7	3.09	5.46E-04	0.0046
ENST000002664810	3.08	1.06E-04	0.0023
IRF8	3.07	2.76E-04	0.0034
NRSN2-AS1	3.06	3.73E-04	0.0039
TUBB4B	3.06	2.04E-04	0.0030
ENST000003386631	3.06	9.54E-03	0.0260
MCM5	3.06	1.29E-05	0.0010
TTC26	3.05	1.54E-04	0.0027
IMPDH2	3.05	1.10E-05	0.0010
AC234772.2	3.05	1.23E-03	0.0072
FRMD6	3.05	4.31E-05	0.0016
RPL22L1	3.05	1.51E-05	0.0011

GGCT	3.04	1.16E-05	0.0010
SLC39A1	3.04	1.80E-05	0.0011
FOXM1	3.04	6.27E-04	0.0050
ENST000003027631	3.04	7.09E-05	0.0019
PA2G4	3.04	2.56E-05	0.0013
MRPS26	3.03	1.01E-04	0.0022
ANKRD18DP	3.03	5.13E-04	0.0045
ZNF367	3.03	7.39E-05	0.0020
HIST1H2AE	3.03	3.23E-05	0.0014
CORO1C	3.03	1.19E-04	0.0024
MRC2	3.02	3.79E-04	0.0039
ENST000002489240	3.02	6.20E-05	0.0018
NUDT10	3.02	1.82E-03	0.0091
HIST1H2BI	3.02	1.12E-05	0.0010
LRFN4	3.02	2.87E-04	0.0035
ENST000002566860	3.02	4.94E-05	0.0017
ALDOA	3.02	1.42E-05	0.0011
EGFL6	3.01	1.63E-03	0.0085
PARPBP	3.01	2.26E-05	0.0012
PRDX3	3.01	2.23E-05	0.0012
LOXL3	3.01	2.97E-03	0.0121
CBR3	3.01	4.18E-04	0.0041
KCNN4	3.00	3.11E-04	0.0036
MRPL37	3.00	1.67E-05	0.0011
FGF2	3.00	2.66E-04	0.0034
MRM1	3.00	5.14E-05	0.0017
PXDC1	3.00	6.82E-03	0.0207
GPR19	2.99	2.01E-04	0.0030
EBNA1BP2	2.99	1.32E-04	0.0025
GAS2L3	2.99	1.38E-03	0.0077
ENST000003234410	2.99	9.27E-03	0.0254
ENST000003200951	2.99	1.93E-02	0.0429
NOCT	2.98	8.94E-05	0.0021
RFC5	2.98	6.84E-05	0.0019
ENST000003113370	2.98	8.58E-05	0.0021
ENST000003192482	2.98	1.74E-04	0.0028
COPZ2	2.98	2.53E-03	0.0110
LGALS1	2.98	2.78E-04	0.0034
RNASEH2A	2.98	6.45E-05	0.0019
ENST000002289551	2.97	5.41E-05	0.0017
SNORD3B-1	2.97	7.45E-04	0.0055
MID1IP1	2.96	2.45E-05	0.0013
ENST000002369593	2.96	3.66E-04	0.0039
CCDC86	2.96	8.73E-04	0.0060
TMCC2	2.96	3.27E-03	0.0128
CDKN1A	2.96	3.01E-04	0.0035
GALE	2.96	3.03E-05	0.0014

LMNB2	2.95	2.26E-05	0.0012
EZH2	2.95	1.80E-05	0.0011
PFKM	2.95	2.50E-05	0.0013
FAM111B	2.95	1.89E-03	0.0092
SLC16A1	2.95	4.30E-05	0.0016
ENST000003251100	2.95	2.17E-05	0.0012
TONSL	2.95	2.40E-04	0.0032
HMBS	2.94	1.36E-03	0.0076
C9orf40	2.94	3.67E-04	0.0039
ATAD3A	2.94	3.60E-04	0.0038
FLVCR2	2.94	5.69E-03	0.0184
SLC38A5	2.94	1.01E-04	0.0022
PHPT1	2.94	7.25E-05	0.0020
TARS	2.93	9.60E-06	0.0010
NDC80	2.93	1.24E-05	0.0010
MT-TP	2.93	3.91E-03	0.0143
MRPL12	2.93	4.75E-05	0.0016
HIST1H2BC	2.93	3.32E-05	0.0015
SYNGR3	2.92	6.88E-03	0.0208
TMEM217	2.92	4.06E-03	0.0147
ENST000003334791	2.91	1.85E-05	0.0011
HELLS	2.91	1.70E-05	0.0011
LRP8	2.91	2.97E-04	0.0035
MCM8	2.91	2.24E-05	0.0012
ALDH1B1	2.91	3.05E-04	0.0036
ENST000003381930	2.91	2.75E-05	0.0014
TTLL12	2.90	4.73E-04	0.0044
ENST00000379612	2.90	9.73E-05	0.0022
ENST000003088600	2.90	9.05E-03	0.0250
RF00003.22	2.89	1.25E-03	0.0073
FANCG	2.89	2.95E-05	0.0014
GSDME	2.89	1.23E-02	0.0310
ENST000002710024	2.89	2.38E-04	0.0032
ZNF704	2.88	3.71E-04	0.0039
LINC00892	2.88	9.72E-04	0.0064
FBXO5	2.88	6.63E-05	0.0019
OPLAH	2.88	3.02E-03	0.0122
SPRED2	2.88	2.23E-02	0.0474
GLA	2.88	3.39E-05	0.0015
HIST1H4B	2.88	1.26E-05	0.0010
TXNDC17	2.87	5.13E-05	0.0017
CCNE1	2.87	9.95E-05	0.0022
ALDH4A1	2.87	1.41E-04	0.0026
MIS18A	2.87	7.31E-05	0.0020
GPRIN1	2.86	3.81E-03	0.0141
CCDC34	2.86	1.26E-03	0.0073
U62317.1	2.86	1.44E-03	0.0079

AC099329.1	2.86	6.82E-03	0.0207
ATP9A	2.86	2.11E-02	0.0455
ENST000002820580	2.85	2.63E-05	0.0013
RFC3	2.85	5.94E-05	0.0018
ENTHD1	2.85	1.07E-02	0.0281
AC013460.1	2.84	6.05E-03	0.0191
VARS	2.84	2.01E-05	0.0012
FSCN1	2.84	2.44E-03	0.0108
ENST000002707081	2.84	2.05E-02	0.0446
GARS	2.84	2.83E-05	0.0014
TRAP1	2.83	2.45E-05	0.0013
LRR1	2.83	4.00E-05	0.0016
MIR222HG	2.83	4.43E-05	0.0016
MPZL1	2.83	2.78E-05	0.0014
SPTBN4	2.83	2.47E-03	0.0108
LAMP3	2.82	2.23E-03	0.0101
AK6	2.82	3.22E-05	0.0014
NPM1P9	2.82	6.66E-03	0.0204
HIST3H2BB	2.82	1.97E-04	0.0030
ABCA1	2.82	5.40E-03	0.0177
PPIL1	2.81	7.46E-05	0.0020
ACY1	2.81	1.81E-04	0.0029
LIMA1	2.81	3.60E-05	0.0015
COX8A	2.81	4.05E-05	0.0016
ENST000003227760	2.81	3.30E-05	0.0015
OXCT2	2.81	8.56E-03	0.0241
ENST000002993000	2.80	7.57E-05	0.0020
PSPH	2.80	1.65E-04	0.0028
ENST000003287031	2.80	2.19E-05	0.0012
ENST000002625070	2.80	6.23E-05	0.0018
MRPL51	2.80	2.43E-04	0.0032
NFE2L3	2.80	8.89E-05	0.0021
ALPK2	2.80	1.01E-04	0.0022
POLD2	2.79	1.16E-04	0.0024
HSPE1	2.79	2.59E-04	0.0033
NOP16	2.79	3.28E-04	0.0037
PCBD1	2.78	6.89E-05	0.0019
THG1L	2.78	9.64E-04	0.0063
ENST000003738330	2.78	9.79E-04	0.0064
ENST000003146660	2.78	6.30E-05	0.0019
MRPL15	2.78	4.46E-05	0.0016
TRAF4	2.78	3.17E-05	0.0014
CPS1	2.77	8.40E-05	0.0021
PRMT1	2.77	1.72E-04	0.0028
CSF1	2.77	2.56E-03	0.0111
SUV39H2	2.77	7.14E-05	0.0020
AC091057.6	2.77	3.74E-05	0.0015

EEF1AKMT4	2.77	4.23E-03	0.0152
ENST000003253240	2.77	3.71E-04	0.0039
RPL26L1	2.76	2.45E-04	0.0032
ENST000003364521	2.76	2.18E-04	0.0031
MICAL2	2.76	2.13E-03	0.0099
AC004687.1	2.76	3.63E-04	0.0039
CKB	2.76	8.81E-03	0.0246
CENPS	2.76	1.29E-03	0.0074
ZNRF1	2.76	1.23E-04	0.0025
MCAT	2.76	7.78E-03	0.0226
GPAT2	2.75	2.28E-04	0.0032
HIST1H4A	2.75	8.66E-05	0.0021
ENST000003299620	2.75	1.04E-02	0.0276
EMC9	2.75	1.55E-03	0.0083
PTPRK	2.75	2.41E-03	0.0107
ENST000003213941	2.75	3.83E-04	0.0039
ENST00000537344	2.74	1.07E-04	0.0023
RIMKLA	2.74	7.56E-04	0.0055
GINS4	2.74	3.99E-05	0.0016
AC135977.1	2.74	3.62E-04	0.0038
ENST000003522971	2.74	1.74E-04	0.0028
CDCA4	2.73	2.88E-05	0.0014
FASLG	2.73	1.42E-04	0.0026
RUVBL2	2.73	1.76E-04	0.0028
LSM2	2.73	1.08E-03	0.0067
SMS	2.73	6.44E-05	0.0019
GART	2.73	2.92E-05	0.0014
PAK1IP1	2.73	4.24E-04	0.0041
ENST000002657092	2.73	9.76E-03	0.0263
PRDX1	2.72	2.97E-04	0.0035
ALYREF	2.72	2.65E-04	0.0034
AC073611.1	2.72	2.72E-03	0.0115
RBFOX2	2.72	6.47E-04	0.0051
LSM4	2.72	3.99E-05	0.0016
AL391832.2	2.72	1.72E-02	0.0394
TEX15	2.72	2.44E-03	0.0107
MRTO4	2.72	3.84E-04	0.0039
E2F7	2.72	2.28E-02	0.0482
GEM	2.71	4.89E-04	0.0044
TG	2.71	4.25E-04	0.0041
FKBP4	2.71	9.52E-05	0.0022
BCAT1	2.71	6.86E-05	0.0019
ALAS1	2.71	6.41E-05	0.0019
PNP	2.71	1.71E-05	0.0011
BBS12	2.71	5.33E-03	0.0176
ENST000002556741	2.71	4.28E-04	0.0041
SMCO4	2.71	1.08E-04	0.0023

GLB1L2	2.71	1.04E-03	0.0066
FDPS	2.70	1.62E-05	0.0011
WDR54	2.70	3.96E-05	0.0016
RPA3	2.70	3.49E-05	0.0015
CENPQ	2.70	2.56E-04	0.0033
CACYBP	2.70	6.56E-05	0.0019
ENST000003033050	2.70	1.58E-04	0.0027
AJM1	2.70	5.17E-04	0.0045
ATAD2	2.70	2.00E-05	0.0012
CHAF1A	2.70	2.60E-05	0.0013
PMAIP1	2.70	6.56E-05	0.0019
MT2A	2.70	3.29E-04	0.0037
AL031777.3	2.70	4.59E-05	0.0016
SNHG4	2.69	3.41E-05	0.0015
ENST000003334834	2.69	1.01E-04	0.0022
ENST000002639850	2.69	4.13E-03	0.0149
ENST000003364301	2.69	1.16E-03	0.0070
RYR1	2.69	9.88E-05	0.0022
KIF7	2.69	2.00E-03	0.0095
CCT5	2.69	1.07E-04	0.0023
AC009237.3	2.68	8.00E-04	0.0057
COQ3	2.68	1.41E-04	0.0026
POP7	2.68	1.82E-04	0.0029
MAD2L2	2.68	1.12E-04	0.0024
PDF	2.68	1.75E-03	0.0089
ENST000003594290	2.68	6.26E-05	0.0018
ENST000002903490	2.67	4.41E-05	0.0016
UTP11	2.67	9.92E-05	0.0022
PDCD2L	2.67	6.62E-05	0.0019
FIGNL1	2.67	8.30E-05	0.0021
RTN2	2.67	6.04E-03	0.0191
AC141586.1	2.67	4.20E-04	0.0041
GALM	2.66	9.15E-05	0.0022
IL10	2.66	1.05E-02	0.0278
PHB	2.66	1.89E-04	0.0029
BCL2L12	2.66	4.94E-04	0.0044
SPX	2.66	3.12E-03	0.0124
FZD5	2.66	3.47E-03	0.0133
GRK3	2.66	1.27E-04	0.0025
GLO1	2.65	1.18E-04	0.0024
CSTF2	2.65	8.57E-05	0.0021
TCEAL9	2.65	2.56E-04	0.0033
PIR	2.65	3.71E-03	0.0139
GSTP1	2.65	7.06E-05	0.0019
LAP3	2.65	2.33E-05	0.0012
CORO1B	2.65	8.42E-05	0.0021
ZNF165	2.65	2.76E-04	0.0034

STOML2	2.65	4.35E-05	0.0016
PARVB	2.64	7.22E-05	0.0020
EIF2S2	2.64	1.21E-05	0.0010
ENST000002916340	2.64	3.34E-04	0.0037
TBX21	2.64	3.32E-04	0.0037
AP002852.1	2.64	2.40E-03	0.0106
MACROD1	2.63	2.52E-04	0.0033
CYP51A1	2.63	2.14E-04	0.0031
ASNS	2.63	3.01E-05	0.0014
SPINT1	2.63	1.86E-04	0.0029
C1QBP	2.63	7.62E-05	0.0020
SLC7A1	2.63	1.86E-04	0.0029
DONSON	2.63	4.25E-05	0.0016
NUDT11	2.63	1.69E-04	0.0028
HAVCR2	2.62	8.23E-04	0.0058
NME2	2.62	5.14E-05	0.0017
CALM3	2.62	4.67E-05	0.0016
MIR663A	2.62	7.24E-04	0.0054
ENST000003278000	2.62	6.52E-05	0.0019
DUT	2.62	6.46E-05	0.0019
BNIP3	2.62	2.11E-05	0.0012
ENST000002387883	2.62	1.81E-02	0.0409
ENST000003165434	2.61	6.17E-03	0.0194
NEMP1	2.61	4.68E-05	0.0016
ZNF436-AS1	2.61	2.09E-03	0.0098
HNRNPAB	2.61	6.77E-04	0.0052
CCDC58	2.60	5.38E-05	0.0017
ENST000003516250	2.60	4.90E-04	0.0044
ENST000002886703	2.60	1.28E-03	0.0074
MAP3K20-AS1	2.60	4.06E-03	0.0147
NUDT1	2.60	3.91E-04	0.0040
ENST000003559680	2.60	1.97E-04	0.0030
ENO2	2.60	1.10E-04	0.0023
PRELID1	2.59	1.04E-04	0.0023
IKBIP	2.59	9.21E-05	0.0022
VTRNA1-3	2.59	4.39E-04	0.0042
HIST2H2AB	2.59	4.04E-05	0.0016
CHPF	2.59	2.43E-04	0.0032
UNC13B	2.59	1.04E-03	0.0066
MED20	2.59	1.84E-04	0.0029
CES3	2.59	1.42E-03	0.0078
CYC1	2.58	5.20E-05	0.0017
SVIL2P	2.58	1.34E-03	0.0076
ZBED8	2.58	5.56E-04	0.0047
LINC02595	2.58	1.24E-03	0.0073
FADS1	2.58	2.02E-02	0.0442
BRIP1	2.58	4.94E-05	0.0017

OAS1	2.58	1.97E-03	0.0095
POLA2	2.58	9.52E-05	0.0022
WEE1	2.58	4.63E-05	0.0016
SSRP1	2.58	1.82E-05	0.0011
WDR18	2.58	3.14E-04	0.0036
ENST000003162181	2.58	8.37E-04	0.0058
SPRY1	2.58	7.01E-04	0.0054
BCAT2	2.57	1.75E-03	0.0089
DOK4	2.57	4.98E-04	0.0045
JOSD2	2.57	1.27E-03	0.0074
ARFGEF3	2.57	9.58E-03	0.0260
SMOX	2.57	1.03E-02	0.0273
HSP90AB1	2.57	3.32E-05	0.0015
AC004816.1	2.57	4.48E-03	0.0157
BOLA3	2.57	1.58E-04	0.0027
ASPHD2	2.57	9.16E-05	0.0022
SEPT14P12	2.56	8.41E-03	0.0238
DHCR7	2.56	1.91E-04	0.0029
AC026403.1	2.56	3.52E-03	0.0134
NOL3	2.56	1.23E-02	0.0310
CHMP5	2.56	1.39E-04	0.0026
WRAP53	2.56	2.10E-04	0.0030
SRM	2.56	5.05E-04	0.0045
ENST000002422572	2.56	1.28E-04	0.0025
ETHE1	2.56	4.04E-04	0.0040
POMP	2.55	6.22E-05	0.0018
BEND5	2.55	2.24E-04	0.0031
KCTD5	2.55	2.95E-05	0.0014
HSD17B6	2.55	1.06E-02	0.0278
SLC37A4	2.55	6.63E-05	0.0019
RAD54B	2.54	2.82E-05	0.0014
SURF4	2.54	3.62E-05	0.0015
ENST000003237440	2.54	5.29E-04	0.0046
POLA1	2.54	2.07E-04	0.0030
TFRC	2.54	9.62E-04	0.0063
LSM12P1	2.54	3.43E-03	0.0132
BCKDK	2.54	6.94E-04	0.0053
ENST000000031002	2.54	6.68E-05	0.0019
DCLRE1A	2.54	1.35E-04	0.0025
SNX8	2.53	1.17E-04	0.0024
ENST000003546941	2.53	4.63E-04	0.0043
SLC25A5	2.53	4.55E-05	0.0016
PELO	2.53	6.76E-05	0.0019
ENST000002707762	2.53	1.13E-04	0.0024
CHTF18	2.53	1.12E-03	0.0068
C16orf45	2.53	3.59E-03	0.0136
GPSM2	2.53	1.46E-04	0.0026

PPIAP45	2.52	2.04E-02	0.0445
EHD4	2.52	2.08E-04	0.0030
AC008736.1	2.52	2.36E-03	0.0105
ENST000003485020	2.52	4.34E-04	0.0042
ZWILCH	2.52	9.39E-05	0.0022
NUP37	2.51	4.99E-05	0.0017
POLD3	2.51	3.83E-05	0.0015
XRCC3	2.51	1.69E-04	0.0028
PRMT5	2.51	7.64E-05	0.0020
TP53I11	2.51	8.37E-04	0.0058
PLAT	2.51	4.74E-04	0.0044
CHAF1B	2.51	5.31E-05	0.0017
SPRED1	2.51	3.85E-03	0.0142
AIMP2	2.51	1.55E-04	0.0027
WIPI1	2.51	6.44E-05	0.0019
ENST000002938312	2.50	9.19E-05	0.0022
TMEM106C	2.50	1.67E-04	0.0028
ABHD17C	2.50	1.49E-03	0.0081
ENST000002304310	2.50	4.40E-04	0.0042
EBP	2.50	3.98E-04	0.0040
SCG5	2.50	4.18E-03	0.0150
PPA1	2.50	2.22E-05	0.0012
FAM207A	2.50	3.13E-03	0.0125
ENST000002958302	2.50	7.88E-05	0.0020
MSH5	2.50	4.70E-05	0.0016
ENST000002961220	2.49	3.24E-05	0.0014
CD70	2.49	7.58E-04	0.0055
STX11	2.49	1.81E-04	0.0029
HDGF	2.49	4.58E-05	0.0016
PLSCR1	2.49	2.46E-04	0.0032
ENST000001997062	2.49	1.25E-03	0.0073
SLC25A19	2.49	1.91E-04	0.0029
CDCA7L	2.49	3.29E-04	0.0037
CCDC74A	2.49	1.09E-03	0.0068
HMGB2	2.49	6.17E-05	0.0018
CISD3	2.49	2.88E-04	0.0035
AC130324.3	2.49	2.77E-03	0.0116
ENST000002653040	2.49	1.30E-04	0.0025
MCM3	2.49	8.74E-05	0.0021
SNORA71C	2.48	2.34E-03	0.0105
ZSWIM4	2.48	1.92E-03	0.0094
CD80	2.48	1.96E-04	0.0030
C9orf64	2.48	2.45E-04	0.0032
ZGRF1	2.48	3.04E-05	0.0014
SQOR	2.48	8.34E-05	0.0021
ENST000002637950	2.48	3.74E-04	0.0039
ELL2	2.48	1.19E-04	0.0024

USP18	2.48	4.09E-04	0.0041
ZC3HAV1L	2.48	1.68E-04	0.0028
AC002116.2	2.48	2.22E-03	0.0101
AC083880.1	2.48	4.12E-03	0.0149
ENST000003301883	2.48	8.13E-03	0.0233
CD7	2.48	8.11E-04	0.0057
ENST000002267250	2.48	3.93E-03	0.0144
RRP9	2.48	1.11E-03	0.0068
LRRC42	2.48	9.64E-05	0.0022
CCT7	2.48	4.54E-05	0.0016
RPP25	2.47	7.02E-03	0.0211
MSH6	2.47	2.23E-05	0.0012
PDIA5	2.47	1.65E-03	0.0086
FANCB	2.47	4.91E-05	0.0017
AC006449.6	2.47	2.31E-04	0.0032
AC006449.9	2.47	2.31E-04	0.0032
RSAD2	2.47	1.41E-03	0.0078
DCUN1D5	2.47	7.25E-05	0.0020
RRM1	2.47	5.85E-05	0.0018
KDM4D	2.47	7.04E-04	0.0054
MRPS18B	2.46	2.78E-05	0.0014
PIM3	2.46	3.44E-05	0.0015
TMEM99	2.46	1.06E-03	0.0067
DEPDC4	2.46	5.83E-04	0.0048
ENST000002653511	2.46	7.71E-05	0.0020
PPIF	2.46	1.74E-04	0.0028
REXO5	2.45	1.99E-02	0.0438
TFDP1	2.45	1.15E-04	0.0024
IARS	2.45	2.26E-05	0.0012
ABCB6	2.45	4.08E-04	0.0041
FDX2	2.45	2.20E-03	0.0101
PFDN2	2.45	5.45E-05	0.0017
SNRPE	2.45	9.31E-05	0.0022
RAN	2.45	2.75E-04	0.0034
AGFG1	2.44	4.03E-05	0.0016
ENST000003142620	2.44	2.36E-04	0.0032
FANCD2	2.44	4.16E-05	0.0016
ENST000003238511	2.44	9.76E-03	0.0263
POFUT1	2.44	4.08E-05	0.0016
C8orf88	2.44	5.72E-03	0.0184
ENST000003345271	2.44	1.86E-03	0.0092
NFKBID	2.43	1.55E-04	0.0027
RAI14	2.43	5.07E-03	0.0170
LRRC34	2.43	3.03E-04	0.0036
ENST000002611911	2.43	3.13E-05	0.0014
HIST1H2BG	2.43	9.11E-05	0.0022
NHP2	2.43	7.48E-05	0.0020

SIPA1L2	2.43	1.74E-03	0.0088
CCT6A	2.43	3.27E-04	0.0037
ENST000002643352	2.43	1.87E-04	0.0029
ENST000003165190	2.43	1.05E-03	0.0066
DUSP14	2.42	2.06E-04	0.0030
FAM234B	2.42	1.03E-03	0.0066
RAC3	2.42	1.34E-03	0.0076
SEPT8	2.42	4.67E-04	0.0043
GBE1	2.42	3.80E-05	0.0015
SPESP1	2.42	1.70E-02	0.0391
TPRG1	2.42	7.11E-03	0.0213
PAOX	2.42	1.10E-02	0.0286
RPF2	2.42	1.12E-04	0.0024
MAPKAPK3	2.42	3.36E-05	0.0015
DNAJC18	2.42	1.51E-04	0.0026
ENST000003238332	2.42	8.25E-04	0.0058
AC064850.1	2.41	2.45E-03	0.0108
COA6	2.41	1.39E-04	0.0026
PRG2	2.41	2.31E-03	0.0104
NSD2	2.41	2.36E-05	0.0012
LINC01355	2.41	1.18E-03	0.0071
NLN	2.41	7.00E-05	0.0019
SAP30	2.41	8.03E-05	0.0020
MRPS23	2.41	4.34E-04	0.0042
ZNF775	2.41	2.94E-04	0.0035
ETS2	2.41	6.00E-04	0.0049
CPXM1	2.41	1.98E-02	0.0435
ENST000002167740	2.41	3.73E-05	0.0015
MFAP1	2.41	6.19E-05	0.0018
ETV4	2.40	6.65E-03	0.0204
SYP	2.40	4.53E-04	0.0043
PPFIA3	2.40	5.47E-04	0.0046
TYMP	2.40	1.36E-03	0.0076
ENST000003011593	2.40	1.89E-02	0.0422
C11orf24	2.40	4.75E-04	0.0044
ENST000003177451	2.40	2.83E-03	0.0117
KDELC1	2.39	2.14E-04	0.0031
C19orf48	2.39	1.44E-04	0.0026
NDUFAB1	2.39	6.38E-05	0.0019
ACAT2	2.39	1.51E-04	0.0026
NR4A1	2.39	5.44E-03	0.0178
NASP	2.39	4.25E-05	0.0016
IFRD2	2.39	7.10E-04	0.0054
NFE2L1	2.39	2.69E-05	0.0013
ENST000002760792	2.39	4.46E-04	0.0042
ADAM9	2.39	5.65E-05	0.0017
PGK1	2.39	1.61E-05	0.0011

ENST000003383820	2.38	5.37E-05	0.0017
CD83	2.38	5.48E-05	0.0017
AL035681.1	2.38	2.48E-03	0.0109
MRPL13	2.38	5.97E-05	0.0018
ENST000003612190	2.38	1.81E-04	0.0029
PRDX4	2.38	1.34E-04	0.0025
PSMG1	2.38	1.08E-04	0.0023
CCDC113	2.38	3.57E-04	0.0038
CCT8	2.38	2.14E-05	0.0012
CHCHD4	2.38	3.79E-04	0.0039
METTL1	2.38	2.51E-03	0.0110
DPP3	2.38	1.39E-04	0.0026
YWHAG	2.38	6.91E-05	0.0019
FANCL	2.37	3.40E-04	0.0037
PNPO	2.37	3.09E-04	0.0036
ENST000002498060	2.37	5.43E-03	0.0178
CENPL	2.37	4.28E-04	0.0041
ADPRH	2.37	3.02E-04	0.0035
CCDC189	2.37	2.72E-03	0.0115
TMPO-AS1	2.37	3.45E-04	0.0038
WDR63	2.36	2.99E-03	0.0121
CLUH	2.36	3.06E-04	0.0036
FARSA	2.36	1.15E-04	0.0024
TEAD1	2.36	1.32E-02	0.0327
ENKD1	2.36	3.73E-03	0.0140
C1orf112	2.36	6.77E-05	0.0019
ENST000002530990	2.36	9.13E-04	0.0061
ENST000002831794	2.36	1.33E-04	0.0025
SNRPG	2.36	1.93E-04	0.0029
HTATSF1P2	2.35	1.03E-02	0.0273
MASTL	2.35	3.44E-04	0.0038
LAGE3	2.35	3.32E-04	0.0037
SLC35F2	2.35	8.30E-05	0.0021
ENST000002712770	2.35	1.90E-04	0.0029
ENST000002161941	2.35	7.83E-05	0.0020
TCP1	2.35	1.61E-04	0.0027
MIR3142HG	2.35	4.97E-03	0.0168
PUS7	2.35	1.47E-04	0.0026
ENST000002996130	2.35	5.21E-04	0.0045
PSMD14	2.35	1.95E-04	0.0030
FDXR	2.35	8.15E-04	0.0057
PDAP1	2.35	1.20E-04	0.0024
COX17	2.34	4.40E-05	0.0016
CETN3	2.34	2.55E-04	0.0033
SNX10	2.34	8.44E-05	0.0021
CEP152	2.34	5.97E-05	0.0018
HIST2H2BE	2.34	3.38E-05	0.0015

MZT1	2.34	4.32E-05	0.0016
ENST000003573240	2.34	3.64E-03	0.0137
PPP2R5D	2.34	2.61E-03	0.0112
ENST000002167560	2.34	1.21E-04	0.0024
C18orf54	2.34	6.22E-04	0.0050
RAC2	2.34	3.29E-04	0.0037
TOM1L1	2.34	1.27E-02	0.0317
MXD3	2.33	2.61E-03	0.0112
AL138759.1	2.33	5.47E-03	0.0179
PC	2.33	3.60E-04	0.0038
PLAGL1	2.33	1.54E-02	0.0365
FH	2.33	1.01E-04	0.0022
PSMB3	2.33	2.09E-04	0.0030
ENST000002760960	2.33	1.21E-04	0.0024
BOP1	2.33	2.24E-03	0.0102
PEA15	2.33	5.41E-04	0.0046
CCT4	2.33	4.00E-05	0.0016
CYSTM1	2.32	3.11E-04	0.0036
EEF1E1	2.32	1.39E-04	0.0026
IFNLR1	2.32	3.89E-03	0.0143
C20orf27	2.32	5.52E-03	0.0180
RFWD3	2.32	3.16E-05	0.0014
ENST000002162812	2.32	1.66E-04	0.0028
PFDN6	2.32	1.46E-04	0.0026
ENST000003316660	2.32	2.28E-03	0.0103
C11orf98	2.31	1.29E-04	0.0025
MSH5-SAPCD1	2.31	1.10E-03	0.0068
SUV39H1	2.31	4.07E-04	0.0041
HIRIP3	2.31	8.79E-05	0.0021
KNTC1	2.31	1.33E-04	0.0025
BCL2L14	2.31	4.30E-04	0.0041
AC069499.1	2.31	5.57E-03	0.0181
NDUFA8	2.31	9.80E-05	0.0022
CCNB1IP1	2.30	4.83E-05	0.0016
GNG5	2.30	1.16E-04	0.0024
RHOC	2.30	1.49E-04	0.0026
CD274	2.30	1.19E-04	0.0024
AC118553.1	2.30	9.09E-03	0.0251
ENST000003595271	2.30	3.90E-03	0.0143
HSPA4	2.30	1.22E-04	0.0025
NACC1	2.30	1.96E-04	0.0030
TMEM273	2.30	5.57E-05	0.0017
ARMCX1	2.29	3.46E-04	0.0038
NUP62	2.29	5.30E-05	0.0017
MRPL39	2.29	9.15E-05	0.0022
ENST000002818710	2.29	1.83E-03	0.0091
ENST000002687971	2.29	2.64E-04	0.0034

TTI2	2.29	1.21E-04	0.0024
LAPTM4B	2.29	8.21E-04	0.0058
NUDCD1	2.29	1.69E-04	0.0028
TIMM10	2.29	3.69E-04	0.0039
CXCR5	2.29	2.56E-04	0.0033
LDHAP4	2.29	1.41E-02	0.0342
MARS	2.29	5.00E-05	0.0017
CDC42BPA	2.29	9.90E-04	0.0064
ITPRIPL2	2.28	1.52E-04	0.0027
NFKBIE	2.28	2.63E-04	0.0033
KLHL23	2.28	2.76E-04	0.0034
SHMT1	2.28	7.30E-05	0.0020
MSRB1	2.28	4.31E-04	0.0041
RUSC2	2.28	2.13E-03	0.0099
MIR663B	2.28	2.68E-03	0.0114
ENST00000621148	2.28	1.02E-03	0.0065
IL23A	2.28	7.64E-05	0.0020
HOMER1	2.28	3.61E-03	0.0137
GEN1	2.28	4.48E-05	0.0016
ENST000003305031	2.28	1.30E-04	0.0025
DBI	2.27	4.77E-04	0.0044
AK2	2.27	3.03E-05	0.0014
ENST000003237010	2.27	3.60E-03	0.0136
CD151	2.27	3.17E-04	0.0037
C4orf46	2.27	9.04E-05	0.0022
IDH1	2.27	2.34E-04	0.0032
ENST000002782243	2.27	6.01E-04	0.0049
VMP1	2.27	7.94E-05	0.0020
LDLR	2.27	1.26E-03	0.0073
TIFA	2.27	4.00E-04	0.0040
SEMA3A	2.26	1.20E-02	0.0305
SH2D3C	2.26	1.47E-02	0.0352
ENST000002678143	2.26	1.86E-03	0.0092
PSMA3	2.26	5.50E-05	0.0017
ENST000003262662	2.26	2.60E-04	0.0033
OTUD7B	2.26	9.74E-05	0.0022
RGS3	2.26	1.83E-04	0.0029
ENST000002982922	2.26	4.47E-04	0.0042
PARP2	2.26	3.60E-04	0.0038
MANF	2.25	9.72E-05	0.0022
ENST000002455390	2.25	9.13E-05	0.0022
ENST000003055600	2.25	3.15E-04	0.0036
COMTD1	2.25	6.14E-04	0.0050
ENST000003042710	2.25	6.18E-04	0.0050
SMIM3	2.25	1.34E-03	0.0076
C21orf58	2.25	4.64E-04	0.0043
ARPC5L	2.25	7.23E-05	0.0020

ZNF668	2.25	1.76E-02	0.0400
NOP10	2.25	1.39E-04	0.0026
PDP2	2.25	2.37E-04	0.0032
BANF1	2.25	2.97E-03	0.0121
ISOC2	2.25	2.49E-03	0.0109
TNIP3	2.24	7.55E-03	0.0222
GEMIN7	2.24	7.47E-04	0.0055
TIAM2	2.24	1.91E-03	0.0093
CLDN12	2.24	4.62E-04	0.0043
ANAPC11	2.24	3.43E-04	0.0038
EPDR1	2.24	1.06E-02	0.0280
MPZ	2.24	1.97E-03	0.0095
PLOD1	2.24	1.93E-03	0.0094
ENST000002994383	2.24	4.86E-03	0.0166
FAM162A	2.24	1.39E-04	0.0026
SLC26A6	2.24	1.68E-03	0.0086
BCL2A1	2.24	6.03E-05	0.0018
ENST000002623750	2.24	3.71E-04	0.0039
FGF14-AS2	2.24	1.78E-02	0.0403
CCT3	2.23	2.19E-04	0.0031
TRIP6	2.23	2.77E-04	0.0034
COX5A	2.23	2.46E-04	0.0032
RAB19	2.23	1.94E-03	0.0094
STARD4	2.23	1.16E-03	0.0070
YIF1A	2.23	3.49E-04	0.0038
KIF3B	2.23	8.06E-05	0.0020
ENST000002606052	2.23	9.02E-04	0.0061
DDX39A	2.23	4.70E-04	0.0044
GOT2	2.22	9.72E-05	0.0022
ENST000002531071	2.22	5.68E-03	0.0184
LMAN1	2.22	4.02E-05	0.0016
FUOM	2.22	6.06E-04	0.0049
RNU1-13P	2.22	2.15E-02	0.0463
ENST000003270350	2.21	4.33E-04	0.0042
ENST000003299082	2.21	2.88E-04	0.0035
ENST000003121890	2.21	6.11E-03	0.0192
HINT2	2.21	6.11E-04	0.0050
VLDLR-AS1	2.21	2.76E-03	0.0116
PXMP2	2.21	7.75E-04	0.0056
NAPSB	2.21	1.26E-03	0.0073
CHCHD2	2.21	4.13E-05	0.0016
PIMREG	2.21	9.71E-03	0.0262
SNHG3	2.21	2.16E-04	0.0031
SASS6	2.21	8.56E-05	0.0021
ENST000002959081	2.20	4.57E-04	0.0043
FADD	2.20	1.72E-04	0.0028
ENST000003307522	2.20	1.40E-04	0.0026

TLN2	2.20	3.28E-04	0.0037
TIMM13	2.20	3.91E-04	0.0040
S100A11	2.20	5.07E-04	0.0045
ENST000002447761	2.20	6.28E-04	0.0050
ENST000003086960	2.20	7.88E-04	0.0056
BRSK1	2.20	5.47E-03	0.0179
AC006538.2	2.20	7.12E-03	0.0213
GADD45GIP1	2.20	1.97E-04	0.0030
ITPKC	2.20	2.28E-04	0.0032
CYFIP1	2.20	2.54E-04	0.0033
NDC1	2.20	8.49E-05	0.0021
TTYH3	2.20	2.94E-03	0.0120
EEF1AKNMT	2.19	7.39E-05	0.0020
MIR663AHG	2.19	8.30E-04	0.0058
ETFB	2.19	5.53E-05	0.0017
C17orf51	2.19	1.40E-04	0.0026
RNVU1-7	2.19	1.18E-02	0.0300
PRKAR1B	2.19	1.28E-04	0.0025
MDH2	2.19	4.39E-04	0.0042
BORA	2.19	6.33E-05	0.0019
POLR1A	2.19	7.33E-04	0.0055
APOBEC3H	2.19	1.50E-03	0.0081
COL5A2	2.19	2.02E-02	0.0442
LINC00888	2.19	1.42E-02	0.0343
E2F2	2.19	3.26E-04	0.0037
CPSF3	2.18	1.92E-04	0.0029
AIFM2	2.18	1.09E-03	0.0068
PARK7	2.18	1.46E-04	0.0026
POP1	2.18	8.52E-04	0.0059
CDK4	2.18	2.88E-04	0.0035
ANP32E	2.18	2.19E-04	0.0031
MAP2K3	2.18	3.86E-04	0.0040
EIF3CL	2.18	2.81E-03	0.0117
YARS	2.18	4.79E-04	0.0044
B4GALNT3	2.18	1.73E-02	0.0395
BZW2	2.18	2.06E-04	0.0030
ENST000003090611	2.18	6.45E-04	0.0051
AHSA1	2.18	2.61E-04	0.0033
PSMC3	2.18	3.59E-04	0.0038
CD9	2.18	9.28E-03	0.0255
PTPN6	2.18	3.91E-04	0.0040
ENST000003549400	2.18	9.72E-03	0.0263
FAM216A	2.18	1.30E-04	0.0025
ENST000003302870	2.18	8.15E-04	0.0057
TOMM40	2.18	1.12E-03	0.0068
IGFBP4	2.18	4.41E-04	0.0042
SLAMF1	2.17	1.36E-04	0.0025

LRRC3	2.17	8.21E-03	0.0234
CU634019.6	2.17	1.86E-02	0.0418
AC099343.2	2.17	4.30E-03	0.0153
ENST000002991734	2.17	7.53E-03	0.0221
F5	2.17	1.90E-04	0.0029
RASAL1	2.17	1.16E-02	0.0298
TDRKH	2.17	1.89E-03	0.0092
NCAPD3	2.17	8.84E-05	0.0021
PSMA6	2.17	9.62E-05	0.0022
KHSRP	2.17	1.78E-04	0.0028
PTGIR	2.17	2.80E-03	0.0116
CARM1	2.17	1.40E-04	0.0026
SLC25A15	2.17	2.46E-04	0.0032
RRP1	2.17	4.71E-04	0.0044
PRPS2	2.17	8.44E-05	0.0021
PARS2	2.16	7.87E-03	0.0228
ENST000002261050	2.16	2.29E-03	0.0103
AC004943.2	2.16	1.10E-03	0.0068
NDUFAF1	2.16	5.40E-04	0.0046
IFI6	2.16	3.57E-03	0.0136
SPIRE1	2.16	1.54E-03	0.0082
AHRR	2.16	9.79E-03	0.0264
MGST2	2.16	2.09E-02	0.0453
CKAP5	2.16	3.55E-05	0.0015
PPP5C	2.16	2.25E-04	0.0031
AP1S1	2.16	1.38E-04	0.0026
BRI3BP	2.16	1.03E-04	0.0023
CD72	2.16	5.06E-03	0.0170
ENST000003089821	2.16	1.77E-03	0.0089
ENST000003228970	2.16	7.33E-04	0.0055
PPAT	2.16	1.08E-04	0.0023
LACTB2	2.16	3.67E-04	0.0039
PSMB6	2.16	3.51E-04	0.0038
GCHFR	2.16	4.78E-04	0.0044
AL591379.1	2.15	1.08E-03	0.0067
NUTF2	2.15	1.46E-04	0.0026
ABHD10	2.15	1.44E-04	0.0026
TALDO1	2.15	1.05E-04	0.0023
PSMD3	2.15	3.11E-04	0.0036
XPOT	2.15	3.90E-05	0.0016
AL441992.1	2.15	2.68E-04	0.0034
NSDHL	2.15	2.99E-04	0.0035
TIMM8A	2.15	2.69E-04	0.0034
GSTZ1	2.15	4.19E-04	0.0041
NINJ1	2.15	8.41E-04	0.0058
ENST000003315521	2.15	1.22E-03	0.0072
ARL3	2.15	1.70E-04	0.0028

IPO5	2.15	3.45E-05	0.0015
ENST000003243403	2.15	6.04E-04	0.0049
BHLHE40	2.15	8.72E-04	0.0060
FIBP	2.14	2.62E-04	0.0033
YWHAQ	2.14	1.35E-04	0.0025
RUVBL1	2.14	3.36E-04	0.0037
CENPJ	2.14	9.79E-05	0.0022
ZC3H12C	2.14	5.04E-03	0.0169
COX6A1	2.14	1.18E-04	0.0024
C2CD4A	2.14	5.36E-03	0.0176
PSMD8	2.14	2.53E-04	0.0033
ENST000002336300	2.14	1.09E-02	0.0285
GRAMD4	2.14	2.69E-04	0.0034
ENST000003105640	2.14	3.38E-03	0.0131
UQCRC1	2.14	5.19E-05	0.0017
WSB2	2.14	3.86E-04	0.0040
UQCRQ	2.14	2.99E-04	0.0035
MFSD10	2.14	1.09E-04	0.0023
NTHL1	2.14	5.99E-04	0.0049
NAGS	2.14	8.00E-03	0.0231
NDUFA7	2.13	1.09E-04	0.0023
ENST000003588960	2.13	3.31E-04	0.0037
BOLA2B	2.13	4.03E-03	0.0146
MIR621	2.13	1.58E-03	0.0083
ID3	2.13	8.54E-04	0.0059
ENST000003261810	2.13	2.23E-04	0.0031
H1F0	2.13	5.61E-04	0.0047
PRDX5	2.13	3.20E-04	0.0037
MRPL11	2.13	7.13E-04	0.0054
TRMT5	2.13	1.76E-04	0.0028
SNRPA	2.13	4.58E-04	0.0043
TREML2	2.13	2.64E-03	0.0113
GCK	2.13	9.93E-03	0.0266
UGDH	2.13	1.03E-03	0.0066
ENST000002967010	2.13	2.64E-04	0.0034
POLR2H	2.12	3.50E-04	0.0038
MVK	2.12	3.00E-04	0.0035
PNMA2	2.12	5.99E-03	0.0190
TUFM	2.12	7.53E-05	0.0020
AL445231.1	2.12	1.02E-02	0.0271
GPX1	2.12	1.15E-04	0.0024
EXOSC5	2.12	6.14E-04	0.0050
DSN1	2.12	1.06E-04	0.0023
C2CD4D-AS1	2.12	1.29E-03	0.0074
CNTROB	2.12	8.02E-05	0.0020
PFKP	2.12	4.16E-04	0.0041
TIMM44	2.12	8.78E-05	0.0021

ARL2	2.12	6.10E-04	0.0050
ATP5MC1	2.12	2.84E-04	0.0035
PTPN7	2.12	1.74E-04	0.0028
CLPP	2.11	2.38E-04	0.0032
ZNF90	2.11	1.61E-04	0.0027
PRPF4	2.11	9.98E-05	0.0022
ALDOC	2.11	2.82E-03	0.0117
NUDC	2.11	4.06E-04	0.0040
FAM210A	2.11	1.11E-04	0.0023
ENST000002539251	2.11	1.81E-03	0.0090
ENST000002702251	2.11	2.44E-04	0.0032
NANP	2.11	5.34E-04	0.0046
NUP58	2.11	5.79E-05	0.0018
CD63	2.11	3.28E-04	0.0037
ACOX3	2.11	4.15E-04	0.0041
AC009779.2	2.11	8.30E-03	0.0236
SCO2	2.11	1.82E-03	0.0090
RNASEH1-AS1	2.11	1.95E-04	0.0030
LRRCC1	2.10	1.56E-03	0.0083
GPR180	2.10	5.82E-04	0.0048
FAM89A	2.10	2.19E-04	0.0031
ADRM1	2.10	5.01E-04	0.0045
UBE2M	2.10	1.09E-04	0.0023
ENST000002642743	2.10	2.12E-03	0.0099
FLOT1	2.10	3.26E-04	0.0037
ENST000003318890	2.10	3.31E-03	0.0129
DOHH	2.10	9.75E-04	0.0064
PPAN	2.10	2.08E-03	0.0097
ENST000002247562	2.09	7.83E-04	0.0056
PSMA2	2.09	1.31E-04	0.0025
AL021068.1	2.09	3.75E-03	0.0140
FBXO6	2.09	1.94E-03	0.0094
ENST000003048003	2.09	6.95E-04	0.0053
ARHGAP19	2.09	6.99E-05	0.0019
EXOSC8	2.09	5.81E-05	0.0018
TKT	2.09	1.91E-04	0.0029
CMTM4	2.09	1.14E-03	0.0069
ZFP92	2.09	5.97E-03	0.0190
ENST000003213581	2.09	8.75E-05	0.0021
BCYRN1	2.08	2.48E-04	0.0032
NOLC1	2.08	7.75E-04	0.0056
VAV2	2.08	1.28E-02	0.0319
CMAHP	2.08	8.84E-05	0.0021
ENST000002998215	2.08	9.91E-03	0.0266
NOB1	2.08	1.29E-04	0.0025
IMPA2	2.08	6.77E-04	0.0052
NR4A3	2.08	1.17E-02	0.0298

NTRK1	2.08	2.37E-02	0.0496
PSMB5	2.08	2.01E-04	0.0030
SLC19A1	2.08	2.08E-03	0.0098
CCDC85B	2.08	3.05E-03	0.0123
RCC2	2.08	6.93E-05	0.0019
FBXL19	2.08	2.35E-04	0.0032
IFI27L1	2.08	3.07E-04	0.0036
CD3EAP	2.08	1.74E-03	0.0088
RCN1	2.08	8.36E-04	0.0058
GNPNAT1	2.08	4.56E-04	0.0043
HK1	2.07	2.63E-04	0.0033
ENST000002649320	2.07	1.76E-04	0.0028
CENPV	2.07	1.67E-04	0.0028
LUCAT1	2.07	6.26E-04	0.0050
MRPS34	2.07	1.01E-04	0.0022
CTU2	2.07	7.17E-04	0.0054
HPRT1	2.07	1.51E-04	0.0026
TTC9C	2.07	1.58E-04	0.0027
TPM4	2.07	4.74E-05	0.0016
SYTL3	2.07	4.72E-03	0.0162
TAX1BP3	2.06	6.50E-04	0.0051
UBL4A	2.06	1.52E-04	0.0026
ENST000002587871	2.06	6.55E-04	0.0051
INSIG1	2.06	5.74E-05	0.0017
ENST000003519860	2.06	2.59E-04	0.0033
ENST000003340820	2.06	4.22E-04	0.0041
JPT1	2.06	2.56E-04	0.0033
SLC31A1	2.05	6.38E-04	0.0051
GSTCD	2.05	2.12E-03	0.0099
CLPB	2.05	2.95E-04	0.0035
NDUFV2P1	2.05	7.00E-03	0.0211
LYSMD1	2.05	7.32E-04	0.0055
CIAO2B	2.05	8.07E-05	0.0020
HMGB1P6	2.05	1.97E-03	0.0095
LRRC59	2.05	1.61E-04	0.0027
PUS3	2.05	1.43E-04	0.0026
EIF3I	2.05	1.41E-04	0.0026
CYTOR	2.05	1.12E-03	0.0068
PGM2	2.05	1.02E-04	0.0022
HSPH1	2.05	6.01E-04	0.0049
OSBPL6	2.05	7.04E-03	0.0212
ZDHHC12	2.05	5.03E-04	0.0045
RHPN2	2.05	2.56E-03	0.0111
NDUFB6	2.05	7.32E-04	0.0055
LRP11	2.04	2.47E-04	0.0032
ENST000003260190	2.04	3.11E-03	0.0124
ENST000002160992	2.04	5.04E-04	0.0045

FAM83D	2.04	1.18E-03	0.0071
PEMT	2.04	1.11E-03	0.0068
SNRPB	2.04	1.18E-04	0.0024
AKIP1	2.04	1.40E-03	0.0078
MTCH2	2.04	6.08E-04	0.0049
CISD1	2.04	4.19E-04	0.0041
ENST000003181580	2.04	1.02E-03	0.0065
ENST000003600010	2.04	3.66E-04	0.0039
WDR12	2.04	1.61E-04	0.0027
JADE3	2.04	1.21E-03	0.0072
CCDC18	2.04	1.57E-03	0.0083
NUP93	2.04	1.02E-04	0.0023
DHRS7B	2.04	3.37E-04	0.0037
UBR7	2.04	1.73E-04	0.0028
ENST000003421731	2.04	9.81E-04	0.0064
ILF2	2.04	2.99E-04	0.0035
AL138724.1	2.04	6.24E-03	0.0195
ENST000003393740	2.04	2.62E-04	0.0033
ENST000002459320	2.04	2.85E-04	0.0035
HCFC1R1	2.03	1.22E-02	0.0309
ZCRB1	2.03	1.25E-04	0.0025
ENST000002615311	2.03	2.18E-04	0.0031
AC051619.7	2.03	5.76E-03	0.0185
CPM	2.03	3.42E-04	0.0038
ZNF311	2.03	2.64E-03	0.0113
SKA2	2.03	2.83E-04	0.0035
EFCAB11	2.03	2.33E-03	0.0104
IDH2	2.03	1.05E-04	0.0023
SRD5A3	2.03	1.32E-03	0.0075
ENST000003336510	2.03	4.02E-04	0.0040
ENST000003176101	2.03	1.49E-03	0.0081
TXNRD1	2.03	1.47E-04	0.0026
ZNF232	2.03	2.52E-03	0.0110
DPAGT1	2.03	1.52E-04	0.0027
RNU5E-6P	2.03	7.08E-03	0.0212
AP002990.1	2.03	3.41E-03	0.0131
MLEC	2.02	6.61E-05	0.0019
CDC123	2.02	4.78E-04	0.0044
CIAO2A	2.02	1.46E-04	0.0026
ODF2	2.02	2.14E-04	0.0031
MEA1	2.02	9.83E-04	0.0064
STAP2	2.02	5.38E-03	0.0177
ZNF670	2.02	7.38E-04	0.0055
GPI	2.02	3.61E-04	0.0038
ECHDC3	2.02	4.29E-03	0.0153
TAF1A	2.02	1.84E-04	0.0029
ENST000002562160	2.02	1.56E-04	0.0027

ENST000002632700	2.02	7.80E-04	0.0056
CTPS1	2.02	5.37E-04	0.0046
DHX33	2.02	1.59E-04	0.0027
MPZL3	2.02	4.42E-04	0.0042
MED18	2.02	3.08E-04	0.0036
SFXN4	2.02	3.69E-04	0.0039
ENST000002641590	2.02	4.35E-03	0.0154
TREX1	2.02	5.22E-03	0.0173
AIFM1	2.02	8.63E-05	0.0021
SKP2	2.02	2.32E-04	0.0032
UNG	2.02	1.68E-04	0.0028
REEP2	2.01	1.07E-03	0.0067
RIDA	2.01	9.03E-04	0.0061
FHOD1	2.01	4.54E-04	0.0043
ACAA2	2.01	1.93E-03	0.0094
FBXO45	2.01	1.31E-04	0.0025
MELTF	2.01	1.22E-02	0.0309
RCCD1	2.01	3.00E-04	0.0035
ENST000002904290	2.01	2.88E-03	0.0118
PCCB	2.00	2.03E-04	0.0030
ENST000003101440	2.00	4.30E-04	0.0041
ENST000003889950	2.00	9.37E-04	0.0062
ENST000002221390	2.00	3.45E-03	0.0132
ENST000003286490	2.00	1.52E-04	0.0027
PSMA5	2.00	1.29E-04	0.0025
ENST000002674151	2.00	1.84E-02	0.0414
MRPL58	2.00	2.59E-04	0.0033
SMC4	2.00	1.12E-04	0.0024
MCUR1	2.00	8.79E-04	0.0060
PHB2	2.00	1.08E-04	0.0023
UBA1	2.00	1.32E-04	0.0025
TMA16	2.00	5.26E-04	0.0046
ACAT1	2.00	9.55E-05	0.0022
ADAM15	2.00	5.57E-04	0.0047
ENST000002253082	2.00	1.61E-02	0.0376
SNRPD1	2.00	4.99E-04	0.0045
ING2	1.99	2.39E-04	0.0032
AC084082.1	1.99	1.55E-03	0.0083
RALB	1.99	9.36E-05	0.0022
SDHB	1.99	1.36E-04	0.0025
PDCD5	1.99	1.28E-04	0.0025
KNOP1	1.99	3.07E-04	0.0036
RANGAP1	1.99	4.90E-04	0.0044
TEDC1	1.99	2.23E-03	0.0101
SRXN1	1.99	8.07E-04	0.0057
TRIM25	1.99	5.28E-03	0.0175
NFKBIL1	1.99	3.46E-04	0.0038

CKAP2	1.99	1.28E-04	0.0025
ENST000003401921	1.99	9.32E-04	0.0062
COL9A2	1.99	1.80E-03	0.0090
BOLA2-SMG1P6	1.99	3.66E-04	0.0039
ENST000003154231	1.98	6.17E-04	0.0050
HSPA2	1.98	6.11E-04	0.0050
ENST000003245930	1.98	5.22E-04	0.0045
MLH1	1.98	1.15E-04	0.0024
C1orf43	1.98	2.96E-04	0.0035
GPR161	1.98	2.32E-02	0.0488
SLC3A2	1.98	7.74E-05	0.0020
PARP1	1.98	1.50E-04	0.0026
C5orf30	1.98	5.03E-04	0.0045
PI4K2B	1.98	1.22E-04	0.0024
ENST000002624322	1.98	5.71E-04	0.0048
AKR1A1	1.98	1.89E-04	0.0029
NOTCH1	1.98	2.12E-04	0.0031
CCDC168	1.98	1.28E-03	0.0074
MRPL23	1.98	6.40E-04	0.0051
ENST000002974400	1.98	3.14E-04	0.0036
CREB3	1.98	1.83E-04	0.0029
RELB	1.98	4.75E-04	0.0044
CDK5	1.97	2.34E-03	0.0105
ENST000002183282	1.97	7.91E-04	0.0057
AFAP1	1.97	3.55E-04	0.0038
SH2D4A	1.97	2.17E-03	0.0100
RWDD2B	1.97	2.99E-03	0.0121
YEATS4	1.97	2.18E-04	0.0031
EIF2S1	1.97	5.19E-04	0.0045
ENST000002941610	1.97	7.67E-04	0.0056
NUP155	1.97	1.02E-04	0.0023
ICMT	1.97	6.51E-04	0.0051
ENST000003386350	1.97	2.79E-04	0.0034
HIST1H2BD	1.97	5.78E-05	0.0018
TRAF2	1.97	1.29E-04	0.0025
TBL3	1.97	2.63E-04	0.0033
NRIP3	1.97	1.67E-02	0.0386
CSE1L	1.97	3.46E-04	0.0038
HRH2	1.96	2.82E-03	0.0117
ENST000002256651	1.96	2.21E-04	0.0031
ZNF768	1.96	7.23E-04	0.0054
CEP170B	1.96	1.11E-02	0.0289
HSPBP1	1.96	8.06E-04	0.0057
C12orf10	1.96	4.67E-04	0.0043
HIVEP3	1.96	5.16E-04	0.0045
EPRS	1.96	4.53E-05	0.0016
RITA1	1.96	2.90E-04	0.0035

CHCHD1	1.96	2.10E-04	0.0030
MYO1A	1.96	6.11E-03	0.0192
CAD	1.96	4.53E-04	0.0043
MRPS15	1.96	1.62E-04	0.0027
MRPL16	1.96	5.37E-04	0.0046
CBX6	1.96	1.07E-04	0.0023
ENST000002298660	1.96	6.10E-04	0.0050
LACTB	1.96	9.69E-05	0.0022
ANKRD22	1.96	3.25E-03	0.0128
ATP5PF	1.95	2.19E-04	0.0031
ENST000003138353	1.95	3.20E-03	0.0126
SLX1B	1.95	2.07E-04	0.0030
RHOQP1	1.95	1.15E-03	0.0070
NUP43	1.95	8.28E-05	0.0021
MRPL3	1.95	2.99E-04	0.0035
ACTG1	1.95	4.02E-04	0.0040
CEP57L1	1.95	2.42E-04	0.0032
HSCB	1.95	7.70E-04	0.0056
ENST000003220671	1.95	2.02E-04	0.0030
ENST000002346260	1.95	1.19E-02	0.0303
ENST000003446291	1.95	9.16E-03	0.0253
CD82	1.95	1.48E-03	0.0080
FAM50B	1.95	5.79E-03	0.0186
ANKRD34A	1.95	1.57E-02	0.0370
SLIRP	1.94	1.28E-03	0.0074
BST2	1.94	6.54E-04	0.0051
PDIA6	1.94	1.31E-03	0.0075
GEMIN5	1.94	3.26E-04	0.0037
ERH	1.94	2.77E-04	0.0034
ARMCX2	1.94	6.91E-03	0.0209
GEMIN4	1.94	5.09E-04	0.0045
DNMT3B	1.94	1.90E-03	0.0093
ENST000002171952	1.94	1.20E-03	0.0071
PKIG	1.94	1.28E-03	0.0074
ENST000003318720	1.94	9.75E-03	0.0263
GTPBP4	1.94	5.74E-04	0.0048
MSH2	1.94	3.21E-04	0.0037
TMEM231	1.94	1.99E-02	0.0437
HCCS	1.94	5.73E-04	0.0048
RAD51D	1.94	3.62E-04	0.0038
PSMA4	1.93	7.83E-05	0.0020
ENST000002886422	1.93	3.69E-03	0.0139
ENST000003213371	1.93	1.02E-03	0.0065
ENST000003016862	1.93	4.12E-03	0.0149
HTATIP2	1.93	8.65E-05	0.0021
AC004477.1	1.93	1.29E-03	0.0074
IGHV1-3	1.93	1.48E-02	0.0355

LINC02416	1.93	8.47E-03	0.0239
CHCHD10	1.93	5.13E-04	0.0045
ACTL6A	1.93	3.76E-04	0.0039
SNAPC1	1.93	4.03E-04	0.0040
STAMBP	1.93	9.74E-05	0.0022
NQO1	1.93	4.47E-03	0.0157
ENST000001682160	1.93	1.43E-03	0.0079
ENST000003017641	1.93	2.00E-04	0.0030
VBP1	1.93	3.61E-04	0.0038
PCGF6	1.93	1.82E-04	0.0029
ST20-MTHFS	1.93	2.16E-03	0.0100
MAGT1	1.93	4.79E-04	0.0044
WDR83	1.93	9.04E-04	0.0061
ERAL1	1.92	1.62E-04	0.0027
ECI1	1.92	2.12E-04	0.0031
WBP4	1.92	4.16E-04	0.0041
HAT1	1.92	2.43E-04	0.0032
SNX5	1.92	6.48E-05	0.0019
NDUFS6	1.92	4.52E-04	0.0043
MVD	1.92	1.61E-04	0.0027
KCTD15	1.92	4.86E-03	0.0165
MAGI3	1.92	6.05E-03	0.0191
C17orf49	1.92	9.78E-03	0.0264
PDSS1	1.92	6.39E-04	0.0051
TMED7-TICAM2	1.92	5.77E-04	0.0048
AC004585.1	1.92	1.02E-02	0.0271
ROM1	1.92	6.74E-03	0.0205
NOC3L	1.92	3.67E-04	0.0039
METTL5	1.92	9.84E-05	0.0022
CNP	1.92	1.69E-04	0.0028
ASL	1.92	2.23E-03	0.0101
ENST000003167881	1.91	2.82E-04	0.0035
ENST000002646700	1.91	4.64E-04	0.0043
ATG101	1.91	3.03E-04	0.0036
SREBF2	1.91	6.66E-04	0.0052
ECHS1	1.91	1.07E-03	0.0067
IL2RB	1.91	3.23E-03	0.0127
OXCT1	1.91	8.62E-05	0.0021
OAZ1	1.91	1.19E-04	0.0024
ENST000002805510	1.91	1.88E-03	0.0092
AC091057.4	1.91	2.98E-03	0.0121
AC254944.2	1.91	2.98E-03	0.0121
ENST000003306510	1.91	1.11E-03	0.0068
SLC43A1	1.91	4.73E-04	0.0044
FKBP7	1.91	3.98E-03	0.0145
ENST000002476651	1.91	2.95E-04	0.0035
PDGFA	1.91	7.23E-03	0.0215

AC069368.1	1.91	1.94E-03	0.0094
CUL7	1.91	9.68E-04	0.0064
IFI35	1.91	2.68E-04	0.0034
TXLNA	1.91	1.01E-04	0.0022
GNL3	1.91	2.95E-04	0.0035
CLIC1	1.90	2.77E-04	0.0034
OAS2	1.90	2.79E-03	0.0116
EMILIN2	1.90	1.92E-03	0.0093
ZNF282	1.90	5.32E-03	0.0175
CD276	1.90	1.07E-02	0.0280
AL359504.2	1.90	1.85E-02	0.0416
LINC00235	1.90	3.90E-03	0.0143
CHRNA5	1.90	4.09E-03	0.0148
HSPA1A	1.90	4.64E-03	0.0161
CMAS	1.90	1.18E-04	0.0024
EML2	1.90	1.79E-04	0.0028
MGME1	1.90	1.95E-04	0.0030
GTF2H4	1.90	1.35E-03	0.0076
PMVK	1.90	2.81E-04	0.0035
HNRNPF	1.90	7.66E-05	0.0020
UEVLD	1.90	1.99E-04	0.0030
G6PD	1.90	2.90E-04	0.0035
TIMM23	1.90	7.19E-04	0.0054
C1orf109	1.90	5.26E-04	0.0046
NDUFB3	1.90	2.65E-04	0.0034
ACOT13	1.89	3.05E-04	0.0036
MRPL24	1.89	5.72E-04	0.0048
DDX11	1.89	5.11E-04	0.0045
ENST000002799071	1.89	1.29E-04	0.0025
RAD18	1.89	1.09E-04	0.0023
NOP56	1.89	1.26E-03	0.0073
UROD	1.89	1.77E-04	0.0028
DUSP4	1.89	5.68E-03	0.0184
SARS	1.89	1.55E-04	0.0027
LYPLA1	1.89	1.08E-04	0.0023
ENST000003077672	1.89	1.03E-02	0.0274
MCTS1	1.89	2.34E-04	0.0032
ENST000003489110	1.89	7.29E-03	0.0216
AL589666.1	1.89	1.47E-02	0.0352
ENST000002501131	1.89	2.36E-04	0.0032
ATP23	1.89	1.74E-02	0.0397
ENST000002656632	1.89	8.01E-04	0.0057
RAD51C	1.89	4.31E-03	0.0153
CUEDC2	1.89	1.79E-04	0.0028
RAB11FIP1	1.88	1.28E-04	0.0025
IL3	1.88	3.17E-03	0.0126
HERC5	1.88	7.16E-04	0.0054

SCARNA22	1.88	3.39E-03	0.0131
PSMD13	1.88	1.57E-04	0.0027
MRPS17	1.88	2.70E-03	0.0114
LAMTOR2	1.88	4.70E-04	0.0044
PPID	1.88	2.99E-04	0.0035
GOLGA2P7	1.88	3.77E-03	0.0140
EEF2KMT	1.88	6.29E-03	0.0196
RNF187	1.88	8.05E-04	0.0057
CELSR3	1.88	7.52E-04	0.0055
ELP5	1.88	4.90E-04	0.0044
FKBP1A	1.87	1.92E-04	0.0029
DKC1	1.87	5.77E-04	0.0048
CMPK2	1.87	7.37E-03	0.0218
PGAM5	1.87	4.28E-04	0.0041
GLE1	1.87	1.10E-04	0.0023
COA7	1.87	5.09E-04	0.0045
NIFK	1.87	2.13E-04	0.0031
ZBTB46	1.87	1.96E-02	0.0433
HTRA2	1.87	6.95E-04	0.0053
HYAL2	1.87	7.15E-03	0.0213
SUPT16H	1.87	2.90E-04	0.0035
ENST000002516070	1.87	1.78E-04	0.0028
PDHA1	1.87	3.00E-04	0.0035
TSR1	1.87	5.94E-04	0.0049
ENST000002855180	1.87	7.56E-04	0.0055
KIF20B	1.87	1.83E-04	0.0029
ALCAM	1.87	4.13E-04	0.0041
NAA38	1.87	7.14E-04	0.0054
TBKBP1	1.87	7.54E-04	0.0055
GNPDA1	1.87	3.77E-03	0.0140
RNF144A-AS1	1.87	1.84E-03	0.0091
CBX8	1.87	3.90E-03	0.0143
RAPGEF3	1.87	9.73E-03	0.0263
CCHCR1	1.86	1.73E-04	0.0028
SIRT4	1.86	6.31E-03	0.0196
ZNF771	1.86	1.35E-02	0.0332
COPG2	1.86	2.21E-04	0.0031
ANKRD24	1.86	4.78E-03	0.0164
SET	1.86	1.26E-03	0.0073
SNX9	1.86	2.05E-03	0.0097
CKLF	1.86	7.64E-04	0.0056
NT5C	1.86	8.24E-04	0.0058
EIF6	1.86	4.09E-03	0.0148
NUDT21	1.86	3.27E-04	0.0037
SWI5	1.86	4.94E-04	0.0044
ENST000002885320	1.86	4.48E-04	0.0042
ENST000002629603	1.86	4.82E-04	0.0044

FLYWCH2	1.86	8.49E-03	0.0240
RNF181	1.86	1.71E-04	0.0028
BCCIP	1.86	2.00E-04	0.0030
GEMIN6	1.86	7.09E-04	0.0054
AHCYL1	1.86	2.18E-04	0.0031
MTERF3	1.86	6.43E-04	0.0051
ENST000000130700	1.86	6.28E-04	0.0050
ENST000003147970	1.86	1.05E-04	0.0023
RPSAP13	1.86	4.16E-03	0.0150
FIRRE	1.86	3.87E-03	0.0143
ACLY	1.86	1.32E-04	0.0025
ENST000003281782	1.86	1.36E-03	0.0076
ATP5MD	1.86	2.46E-04	0.0032
UQCC2	1.86	1.98E-04	0.0030
ERG28	1.85	7.82E-04	0.0056
ELAVL1	1.85	2.12E-04	0.0031
SIAH2	1.85	9.41E-05	0.0022
GAR1	1.85	1.46E-03	0.0080
GTF3C5	1.85	1.95E-04	0.0030
MPG	1.85	3.24E-04	0.0037
ENST000003601280	1.85	2.47E-04	0.0032
AC097534.1	1.85	4.15E-03	0.0150
ENST000002425770	1.85	7.49E-03	0.0221
CCDC15	1.85	3.07E-03	0.0123
JPT2	1.85	6.98E-04	0.0053
ITGA2	1.85	4.91E-04	0.0044
MBD3	1.85	2.03E-03	0.0096
ELK1	1.85	4.11E-04	0.0041
SHC4	1.85	1.00E-03	0.0065
FP565260.1	1.85	1.38E-02	0.0336
AC018926.2	1.85	7.64E-03	0.0223
KPNA2	1.84	1.30E-04	0.0025
CCDC47	1.84	5.16E-04	0.0045
TNFAIP8L2	1.84	3.75E-04	0.0039
GMPPB	1.84	3.37E-03	0.0131
SLC2A1	1.84	4.57E-04	0.0043
ARID5A	1.84	4.06E-04	0.0040
ATP5MF	1.84	1.79E-04	0.0028
NAA20	1.84	3.58E-04	0.0038
ENST000003074500	1.84	4.49E-03	0.0157
MRPL19	1.84	2.42E-04	0.0032
AC010422.5	1.84	1.83E-03	0.0091
TWF2	1.84	1.25E-04	0.0025
HECTD2	1.84	1.07E-03	0.0067
ZNF473	1.84	7.30E-04	0.0055
KEAP1	1.84	4.98E-04	0.0045
REEP4	1.84	2.23E-03	0.0101

HUWE1	1.84	6.33E-04	0.0051
HDLBP	1.84	7.64E-05	0.0020
CENPP	1.83	8.84E-03	0.0246
ZNF57	1.83	2.85E-03	0.0118
POLDIP2	1.83	6.65E-04	0.0052
TMPO	1.83	3.23E-04	0.0037
RPP40	1.83	8.88E-03	0.0247
KCTD11	1.83	1.24E-02	0.0313
MRPL40	1.83	6.50E-04	0.0051
ZNF724	1.83	2.30E-04	0.0032
BUD31	1.83	2.32E-04	0.0032
MRPL45	1.83	1.96E-04	0.0030
PSMD2	1.83	3.19E-04	0.0037
ENST000002606651	1.82	9.33E-05	0.0022
SCOC	1.82	5.13E-04	0.0045
SNORD91B	1.82	5.88E-03	0.0188
ENST000003115593	1.82	8.98E-03	0.0249
WDR24	1.82	6.52E-04	0.0051
MRPL18	1.82	5.94E-04	0.0049
TBL2	1.82	3.53E-04	0.0038
LSM11	1.82	2.17E-04	0.0031
ENST000003084361	1.82	1.67E-03	0.0086
ENST000003403420	1.82	1.20E-02	0.0305
ETF1	1.82	9.98E-04	0.0064
POLR1B	1.82	2.05E-04	0.0030
TAP1	1.82	1.96E-04	0.0030
LIN9	1.82	4.57E-04	0.0043
SPA17	1.82	1.63E-03	0.0085
ULK4P2	1.82	7.44E-03	0.0220
MRPS12	1.82	7.28E-04	0.0055
TTC4	1.82	5.84E-04	0.0048
ENST000003188421	1.82	1.20E-03	0.0071
RNPEP	1.82	9.77E-04	0.0064
TMEM70	1.82	2.26E-04	0.0031
ENST000003082713	1.82	2.86E-03	0.0118
TSTA3	1.82	9.19E-04	0.0062
TMEM201	1.82	2.04E-02	0.0445
CANX	1.82	1.66E-04	0.0028
NDUFB7	1.82	5.17E-04	0.0045
HAUS4	1.81	1.65E-03	0.0086
GALNT2	1.81	2.07E-04	0.0030
ENST000003022431	1.81	1.16E-03	0.0070
PFN1	1.81	1.34E-03	0.0076
PSMG3	1.81	2.75E-03	0.0116
ENST000002403330	1.81	9.59E-04	0.0063
IFI27L2	1.81	3.89E-04	0.0040
HSPA13	1.81	1.18E-04	0.0024

IFT46	1.81	4.74E-04	0.0044
CMYA5	1.81	2.88E-03	0.0118
HIST1H1C	1.81	1.75E-04	0.0028
ARMT1	1.81	4.89E-04	0.0044
ENST000003047861	1.81	2.07E-04	0.0030
PPM1G	1.81	4.24E-04	0.0041
ENST000002674060	1.81	6.06E-03	0.0191
SMIM30	1.81	5.71E-04	0.0048
SRR	1.81	1.31E-03	0.0075
NTMT1	1.81	3.49E-04	0.0038
IRF7	1.81	4.36E-03	0.0154
HDAC7	1.81	6.73E-04	0.0052
RBPJ	1.81	1.11E-04	0.0023
MRPL36	1.81	9.91E-04	0.0064
APOO	1.80	1.33E-03	0.0075
NMB	1.80	7.84E-03	0.0228
IKZF4	1.80	1.43E-02	0.0345
NDST1	1.80	4.84E-03	0.0165
ENST000003060520	1.80	3.41E-04	0.0037
NDUFA6	1.80	2.09E-02	0.0452
HIST1H2AC	1.80	5.59E-04	0.0047
SLC25A17	1.80	4.46E-04	0.0042
BCL6	1.80	7.94E-03	0.0230
RPP25L	1.80	1.54E-03	0.0082
DAD1	1.80	5.86E-04	0.0048
SFXN1	1.80	1.51E-04	0.0026
C6orf52	1.80	1.01E-02	0.0270
ENST000003255992	1.80	6.11E-04	0.0050
CDK7	1.80	1.70E-04	0.0028
TCEA1	1.80	2.29E-04	0.0032
GUCD1	1.80	1.81E-04	0.0029
NDUFB8	1.80	1.08E-04	0.0023
MRPS18A	1.80	3.44E-04	0.0038
TFB1M	1.80	2.63E-04	0.0033
HGH1	1.80	2.30E-03	0.0104
GCNT1	1.79	1.49E-03	0.0081
IFIH1	1.79	2.51E-03	0.0109
RPL12P16	1.79	1.64E-02	0.0380
DNPH1	1.79	1.45E-02	0.0350
ENST000003017881	1.79	1.33E-03	0.0075
ENST000002982880	1.79	1.23E-02	0.0311
CEP72	1.79	4.50E-03	0.0157
DDB2	1.79	1.40E-04	0.0026
SSTR3	1.79	1.22E-02	0.0308
MRPL47	1.79	2.59E-04	0.0033
UBE2V2	1.79	1.54E-04	0.0027
XRCC6	1.79	2.60E-04	0.0033

ENST000003310790	1.79	1.36E-03	0.0076
LRRC20	1.79	2.16E-03	0.0100
ENST000002646890	1.79	1.10E-03	0.0068
MOSPD3	1.79	2.07E-02	0.0450
C6orf141	1.79	7.47E-03	0.0220
RDX	1.79	2.38E-04	0.0032
GPAA1	1.79	3.35E-03	0.0130
PALD1	1.79	1.70E-03	0.0087
RAPH1	1.79	1.35E-03	0.0076
ENST000002623061	1.79	1.03E-03	0.0066
AL590483.1	1.79	1.29E-02	0.0321
FSTL3	1.78	6.67E-03	0.0204
ABCF1	1.78	2.69E-03	0.0114
TMEM14C	1.78	2.09E-04	0.0030
PKP4	1.78	1.26E-03	0.0073
PSMB2	1.78	2.87E-04	0.0035
C16orf95	1.78	1.24E-02	0.0311
ENOPH1	1.78	1.21E-04	0.0024
MTHFD1	1.78	2.71E-04	0.0034
DYNC2LI1	1.78	1.69E-03	0.0087
MYCBP	1.78	2.25E-04	0.0031
SPINDOC	1.78	2.73E-03	0.0115
CTIF	1.78	1.79E-02	0.0405
PRIM1	1.78	9.55E-04	0.0063
ATP5F1B	1.78	1.29E-04	0.0025
DARS	1.78	1.10E-04	0.0023
ENST000002766920	1.78	7.75E-04	0.0056
TFPT	1.78	4.08E-04	0.0041
LYAR	1.78	1.69E-03	0.0086
DHX32	1.78	3.44E-04	0.0038
ENST000002810811	1.78	2.12E-03	0.0099
GNL2	1.78	4.83E-04	0.0044
ADPRHL2	1.78	3.40E-04	0.0037
SLBP	1.78	1.07E-03	0.0067
ODC1	1.78	7.91E-03	0.0229
SF3B6	1.78	3.64E-04	0.0039
FBL	1.78	7.87E-04	0.0056
IMMT	1.78	2.26E-04	0.0031
NCAPH2	1.78	1.66E-04	0.0028
BZW1P2	1.77	2.23E-03	0.0101
KHK	1.77	8.44E-04	0.0059
FANCE	1.77	2.14E-03	0.0099
UQCRH	1.77	4.00E-04	0.0040
MX1	1.77	4.39E-03	0.0155
P4HA1	1.77	2.93E-04	0.0035
RPLP0P6	1.77	2.30E-02	0.0485
PPP1R7	1.77	2.03E-04	0.0030

DESI1	1.77	3.75E-04	0.0039
PES1	1.77	1.29E-02	0.0321
NEK6	1.77	2.35E-02	0.0493
PSME2	1.77	6.88E-04	0.0053
ENST000003453581	1.77	1.08E-02	0.0282
BUD13	1.77	2.36E-04	0.0032
SRPRB	1.77	2.91E-04	0.0035
ENST000003418720	1.77	3.48E-03	0.0133
SIGMAR1	1.77	3.37E-03	0.0131
PPIH	1.77	2.98E-04	0.0035
NANS	1.77	3.36E-04	0.0037
VARS2	1.77	2.95E-04	0.0035
LARP4	1.77	4.04E-04	0.0040
MORF4L2	1.77	1.18E-04	0.0024
POLR3K	1.77	3.59E-04	0.0038
COPB2	1.77	9.62E-05	0.0022
TMED8	1.77	1.26E-04	0.0025
ENST000003131170	1.76	2.04E-02	0.0445
ENST000003367330	1.76	6.93E-03	0.0209
SLC17A9	1.76	2.08E-03	0.0097
TOMM40L	1.76	1.95E-03	0.0094
BZW1	1.76	2.08E-03	0.0098
COQ8B	1.76	5.80E-04	0.0048
MSMO1	1.76	1.23E-03	0.0073
PTPN11	1.76	2.17E-04	0.0031
CDKN2AIPNL	1.76	8.97E-04	0.0061
NPM1	1.76	1.66E-04	0.0028
ENST000003022710	1.76	7.48E-04	0.0055
MKS1	1.76	5.27E-03	0.0174
FAM43A	1.76	1.73E-02	0.0395
ENST000002834290	1.76	1.05E-02	0.0277
SMIM15	1.76	2.50E-04	0.0033
RAB27A	1.76	1.55E-04	0.0027
PLCXD1	1.76	1.03E-03	0.0066
ENST000003136082	1.76	1.24E-03	0.0073
TNFRSF14-AS1	1.76	7.06E-04	0.0054
NLRX1	1.75	1.05E-03	0.0066
B9D1	1.75	1.98E-02	0.0437
PSMD11	1.75	2.30E-04	0.0032
AC011893.1	1.75	1.32E-02	0.0327
PDCD11	1.75	1.91E-04	0.0029
BARD1	1.75	1.71E-04	0.0028
MAP3K20	1.75	1.24E-02	0.0311
NAGA	1.75	2.90E-04	0.0035
MRPL35	1.75	5.28E-04	0.0046
PRIMPOL	1.75	7.60E-03	0.0223
KIAA0895L	1.75	8.50E-03	0.0240

ENST000002294160	1.75	6.27E-03	0.0195
PLS1	1.75	1.01E-03	0.0065
AC129492.1	1.75	3.08E-03	0.0123
RF00024.1	1.75	5.30E-04	0.0046
ADD2	1.75	9.78E-04	0.0064
FARSB	1.75	2.81E-04	0.0035
DDX19B	1.75	3.93E-03	0.0144
SAE1	1.75	4.83E-04	0.0044
IPO7	1.75	1.83E-04	0.0029
NFKBIA	1.75	4.34E-04	0.0042
ATP5MC3	1.74	4.24E-04	0.0041
ENST000003140670	1.74	2.44E-03	0.0108
ENST000003292142	1.74	3.79E-03	0.0141
ST3GAL4	1.74	3.86E-03	0.0142
SLC50A1	1.74	4.05E-04	0.0040
HDGFL2	1.74	5.08E-04	0.0045
COA4	1.74	2.03E-04	0.0030
MYH15	1.74	1.61E-02	0.0375
ENST000003438180	1.74	3.42E-04	0.0038
DNMT1	1.74	1.88E-04	0.0029
HPF1	1.74	5.19E-04	0.0045
TRIM69	1.74	5.11E-04	0.0045
ZDHHC16	1.74	1.18E-03	0.0071
PDRG1	1.74	5.39E-04	0.0046
MAP3K21	1.74	1.06E-03	0.0067
INTS5	1.74	5.70E-04	0.0048
RAB20	1.74	3.21E-03	0.0127
NCKAP1	1.74	1.66E-03	0.0086
AC012146.1	1.74	2.93E-03	0.0120
TMEM186	1.73	2.03E-03	0.0096
ISG15	1.73	2.17E-02	0.0464
VPS25	1.73	2.90E-04	0.0035
HIF1A	1.73	2.90E-04	0.0035
AC105137.2	1.73	6.68E-03	0.0204
TWNK	1.73	2.04E-03	0.0096
IL15RA	1.73	1.41E-03	0.0078
C3orf52	1.73	1.67E-03	0.0086
SSSCA1	1.73	1.97E-02	0.0435
DUSP23	1.73	9.69E-03	0.0262
KIF5A	1.73	2.03E-02	0.0443
ENST000002623190	1.73	7.05E-03	0.0212
CBX1	1.73	2.81E-04	0.0035
DOLPP1	1.73	6.27E-04	0.0050
AC099850.1	1.73	1.57E-02	0.0368
LNP1	1.73	2.40E-03	0.0106
NUDT15	1.73	2.17E-04	0.0031
ENST000001695510	1.73	1.16E-03	0.0070

PIGX	1.73	6.07E-04	0.0049
CA11	1.73	2.40E-03	0.0106
TPD52L2	1.73	3.73E-04	0.0039
PIP5K1A	1.73	1.37E-04	0.0026
METAP2	1.73	3.70E-04	0.0039
SNRPF	1.73	5.34E-04	0.0046
SRPK1	1.73	1.58E-04	0.0027
RDH11	1.73	1.75E-04	0.0028
RNF26	1.73	3.35E-03	0.0130
APEX1	1.72	1.19E-03	0.0071
ZNF625-ZNF20	1.72	1.08E-03	0.0067
ENST000003343790	1.72	6.46E-04	0.0051
PNPT1	1.72	1.59E-04	0.0027
ENST000003114810	1.72	4.52E-04	0.0043
AL591845.1	1.72	3.86E-03	0.0142
PSMD4	1.72	2.13E-04	0.0031
TOPBP1	1.72	2.28E-04	0.0032
ENST000003163860	1.72	4.12E-04	0.0041
ZNF788P	1.72	2.94E-03	0.0120
ENST000002705171	1.72	2.61E-03	0.0112
ENST000003437880	1.72	8.87E-04	0.0060
GSS	1.72	2.23E-04	0.0031
POLR2I	1.72	1.09E-03	0.0067
AFMID	1.72	7.29E-04	0.0055
RINL	1.72	1.95E-04	0.0030
MTIF2	1.72	3.04E-04	0.0036
ENST000003075330	1.72	1.15E-03	0.0070
SEC23B	1.72	1.27E-04	0.0025
PAFAH1B3	1.71	1.67E-03	0.0086
ENST000002693730	1.71	3.68E-04	0.0039
RAB23	1.71	1.69E-03	0.0087
40422	1.71	2.79E-03	0.0116
DNAJA3	1.71	2.39E-04	0.0032
ENST000003034360	1.71	1.80E-04	0.0029
NUP107	1.71	2.94E-04	0.0035
PSMA1	1.71	3.38E-04	0.0037
NETO2	1.71	9.19E-03	0.0253
ENST000003571660	1.71	1.63E-02	0.0378
DNAJC9	1.71	9.43E-04	0.0063
PGRMC1	1.71	8.86E-04	0.0060
PSMD12	1.71	2.15E-04	0.0031
PPP1R18	1.71	3.60E-04	0.0038
SAC3D1	1.71	5.88E-03	0.0188
HDDC2	1.71	4.65E-04	0.0043
NCKIPSD	1.71	2.11E-03	0.0098
ALKBH2	1.71	7.61E-04	0.0056
ENST000002642050	1.71	2.75E-04	0.0034

ENST000002781000	1.71	1.04E-03	0.0066
ME2	1.70	1.91E-04	0.0029
WDR46	1.70	3.02E-03	0.0122
SNORA71A	1.70	2.17E-02	0.0464
GOLIM4	1.70	1.59E-03	0.0084
SNHG19	1.70	1.43E-03	0.0079
POLR2F	1.70	5.42E-04	0.0046
RBFA	1.70	3.30E-03	0.0129
RNF5	1.70	7.94E-04	0.0057
ROMO1	1.70	9.44E-04	0.0063
SERBP1	1.70	4.08E-04	0.0041
EPB41L2	1.70	2.68E-04	0.0034
KIAA1671	1.70	1.62E-03	0.0085
TIMM17B	1.70	3.14E-04	0.0036
ATP5PB	1.70	3.95E-04	0.0040
PUM3	1.70	7.58E-04	0.0055
AC087632.1	1.70	1.21E-03	0.0072
DMGDH	1.70	1.81E-03	0.0090
RTCB	1.70	2.35E-04	0.0032
GRWD1	1.70	2.40E-03	0.0107
STAT5A	1.70	1.44E-04	0.0026
CHMP2A	1.70	6.04E-04	0.0049
GLDC	1.70	2.07E-02	0.0451
IRF5	1.70	1.46E-03	0.0080
SRF	1.70	6.41E-04	0.0051
ZNF584	1.69	1.01E-03	0.0065
ZNF492	1.69	9.49E-04	0.0063
AC107871.1	1.69	1.10E-03	0.0068
WDR3	1.69	1.15E-03	0.0069
TMEM69	1.69	5.73E-04	0.0048
LRRC32	1.69	3.50E-03	0.0134
MON1A	1.69	1.11E-02	0.0289
ALDH18A1	1.69	3.17E-04	0.0036
MRPL22	1.69	2.45E-04	0.0032
UTP14A	1.69	9.89E-04	0.0064
ENST000003162730	1.69	2.46E-03	0.0108
MRPS2	1.69	1.02E-03	0.0065
ENST000003129702	1.69	3.96E-03	0.0145
TMEM177	1.69	9.50E-04	0.0063
ENST000002100601	1.69	1.25E-02	0.0314
ENST000003543591	1.69	6.00E-03	0.0190
HSD17B12	1.68	4.90E-04	0.0044
MSL3P1	1.68	5.05E-03	0.0170
SAAL1	1.68	5.13E-04	0.0045
PRIM2	1.68	4.05E-04	0.0040
FTSJ3	1.68	2.93E-03	0.0120
CEBPG	1.68	1.76E-04	0.0028

ENST000003230642	1.68	3.22E-03	0.0127
NUDT5	1.68	6.64E-04	0.0052
PEX26	1.68	3.31E-04	0.0037
AC017104.5	1.68	1.69E-02	0.0389
PHF19	1.68	1.06E-03	0.0066
WDCP	1.68	6.14E-04	0.0050
GFOD1	1.68	4.21E-03	0.0151
ENST000003164230	1.68	6.96E-03	0.0210
ENST000003020350	1.68	1.50E-04	0.0026
CBX3	1.68	3.28E-04	0.0037
ENST000003008704	1.68	2.33E-04	0.0032
DYNLL1	1.68	1.28E-03	0.0074
KCTD21	1.68	1.38E-02	0.0337
NBL1	1.68	8.25E-03	0.0235
MAP3K8	1.68	2.53E-04	0.0033
EIF2B3	1.68	4.28E-04	0.0041
PRELID3B	1.67	1.68E-04	0.0028
ENST000002623760	1.67	1.57E-03	0.0083
GTF3C6	1.67	3.64E-03	0.0137
CASP3	1.67	3.70E-04	0.0039
NSMCE1	1.67	3.74E-04	0.0039
SDF2L1	1.67	3.01E-03	0.0122
SLC39A7	1.67	1.95E-04	0.0030
SNRPD3	1.67	1.26E-03	0.0073
PMF1	1.67	3.30E-04	0.0037
ENST000003375391	1.67	6.53E-04	0.0051
DNAJA1	1.67	9.03E-04	0.0061
PSMB1	1.67	5.03E-04	0.0045
PSMB8	1.67	2.93E-04	0.0035
SARNP	1.67	1.54E-04	0.0027
TMEM147	1.67	4.89E-04	0.0044
SEC13	1.67	1.61E-04	0.0027
HAUS8	1.67	3.93E-04	0.0040
NRAV	1.67	1.52E-02	0.0361
P4HB	1.67	1.92E-04	0.0029
TRIM47	1.66	9.95E-04	0.0064
MCRIP2	1.66	1.41E-02	0.0342
FASN	1.66	7.48E-04	0.0055
ESD	1.66	2.02E-04	0.0030
BOLA1	1.66	6.65E-04	0.0052
TMED9	1.66	3.43E-04	0.0038
SCOC-AS1	1.66	1.83E-02	0.0413
CHIT1	1.66	1.02E-02	0.0272
ENST000002731561	1.66	7.77E-04	0.0056
HDHD5	1.66	2.99E-04	0.0035
BAK1	1.66	4.17E-04	0.0041
GLRX2	1.66	2.98E-03	0.0121

PFDN4	1.66	2.22E-04	0.0031
PRXL2A	1.66	4.52E-04	0.0043
LSM12	1.66	3.85E-04	0.0039
NR2F6	1.66	1.80E-02	0.0407
ZNF787	1.66	1.60E-03	0.0084
DBF4B	1.66	8.05E-03	0.0231
ENST000002906630	1.65	7.89E-03	0.0228
RAB39B	1.65	5.06E-04	0.0045
AL139300.1	1.65	6.49E-04	0.0051
EIF4G1	1.65	1.70E-04	0.0028
ENST000003187873	1.65	8.08E-03	0.0232
ZNF726	1.65	1.81E-03	0.0090
CHSY1	1.65	3.47E-04	0.0038
MAD2L1BP	1.65	3.68E-04	0.0039
SEC61B	1.65	3.64E-04	0.0039
RRAS2	1.65	5.09E-04	0.0045
TRAF3	1.65	2.55E-04	0.0033
TICRR	1.65	1.73E-03	0.0088
MTLN	1.65	4.23E-03	0.0152
SLC9A3-AS1	1.65	2.29E-02	0.0484
RAC1	1.65	3.32E-04	0.0037
ZBTB7B	1.65	8.68E-04	0.0060
FABP5	1.65	1.23E-02	0.0310
EARS2	1.65	1.34E-03	0.0076
EPS8L1	1.64	1.76E-02	0.0400
GDI2	1.64	2.61E-04	0.0033
ODF3B	1.64	2.64E-03	0.0113
ENST000003101320	1.64	2.14E-03	0.0099
PRKRA-AS1	1.64	6.50E-04	0.0051
SEPHS1	1.64	3.51E-04	0.0038
PRDX2	1.64	5.41E-04	0.0046
C1orf174	1.64	7.78E-04	0.0056
TMED1	1.64	1.59E-03	0.0084
RASGRP4	1.64	3.11E-03	0.0124
MRPS35	1.64	1.92E-04	0.0029
XRCC2	1.64	2.20E-04	0.0031
APTX	1.64	4.47E-04	0.0042
AL132780.3	1.64	2.93E-03	0.0120
SREBF1	1.64	9.03E-03	0.0250
AAMP	1.64	9.77E-04	0.0064
RAD1	1.64	4.22E-04	0.0041
NABP2	1.64	1.72E-03	0.0087
SLX1A	1.64	6.95E-03	0.0210
ERI1	1.64	5.89E-04	0.0049
NTAN1P2	1.64	5.33E-03	0.0176
EIF4A3	1.64	9.14E-04	0.0061
MRPL14	1.64	5.69E-04	0.0048

PYCR3	1.64	2.03E-02	0.0444
MAMDC4	1.64	1.87E-03	0.0092
ZW10	1.64	2.48E-03	0.0109
PGBD1	1.64	9.01E-03	0.0250
APOBEC3C	1.64	5.18E-03	0.0173
HYOU1	1.64	1.79E-04	0.0028
HNRNPUL2-BSCL2	1.64	1.94E-03	0.0094
MAST2	1.64	1.29E-03	0.0074
C12orf60	1.64	7.75E-03	0.0226
TMEM44	1.64	3.44E-03	0.0132
AC004706.3	1.64	1.68E-02	0.0387
RPA2	1.64	5.38E-04	0.0046
RF00002.1	1.63	4.27E-03	0.0152
RF00002.2	1.63	4.27E-03	0.0152
RF00002.5	1.63	4.27E-03	0.0152
RNA5-8SN1	1.63	4.27E-03	0.0152
RNA5-8SN2	1.63	4.27E-03	0.0152
RNA5-8SN3	1.63	4.27E-03	0.0152
RNA5-8SN4	1.63	4.27E-03	0.0152
RNA5-8SN5	1.63	4.27E-03	0.0152
PRKDC	1.63	1.08E-04	0.0023
DDX21	1.63	9.93E-04	0.0064
CGAS	1.63	1.26E-03	0.0073
AARS	1.63	1.56E-02	0.0367
TNFAIP1	1.63	1.85E-03	0.0091
PSMD10	1.63	4.05E-04	0.0040
ADGRA3	1.63	2.11E-02	0.0457
ACD	1.63	1.09E-03	0.0067
DDX41	1.63	7.15E-04	0.0054
ENST000003448431	1.63	8.51E-03	0.0240
AC091057.1	1.63	1.15E-03	0.0069
ITPRIPL1	1.63	2.74E-03	0.0115
SNORD3B-2	1.63	3.99E-04	0.0040
NEXN	1.62	4.25E-03	0.0152
LGALS14	1.62	2.06E-02	0.0448
GLUD1	1.62	1.84E-04	0.0029
CFAP20	1.62	2.43E-03	0.0107
ILVBL	1.62	2.03E-03	0.0096
ABCD3	1.62	6.52E-04	0.0051
PHLDA1	1.62	3.65E-03	0.0138
KMT5C	1.62	4.90E-03	0.0166
ENST000002845510	1.62	1.70E-02	0.0391
ENST000003321180	1.62	1.60E-02	0.0374
STT3A	1.62	2.42E-04	0.0032
APOBEC3G	1.62	9.70E-04	0.0064
BRIX1	1.62	5.66E-04	0.0048
ENST000003306192	1.62	1.04E-03	0.0066

ACADM	1.62	3.68E-04	0.0039
FAM161A	1.62	6.60E-03	0.0202
COQ2	1.62	1.31E-03	0.0075
ENST000003025505	1.62	4.28E-04	0.0041
ENST000002620330	1.62	2.29E-04	0.0032
RFC1	1.62	2.01E-04	0.0030
DHODH	1.61	9.85E-04	0.0064
C15orf38-AP3S2	1.61	2.33E-03	0.0104
SEC61G	1.61	2.99E-04	0.0035
POP4	1.61	1.65E-03	0.0086
PSMC4	1.61	1.58E-03	0.0083
ENST000003180031	1.61	1.09E-02	0.0285
UBB	1.61	7.32E-04	0.0055
TOMM22	1.61	9.56E-04	0.0063
NUDCD2	1.61	2.47E-04	0.0032
GPANK1	1.61	9.96E-04	0.0064
TKFC	1.61	9.55E-04	0.0063
TGDS	1.61	2.82E-04	0.0035
PHF6	1.61	3.45E-04	0.0038
RRM2B	1.61	3.79E-04	0.0039
MINPP1	1.61	9.83E-04	0.0064
DUSP3	1.61	1.44E-03	0.0079
TMEM173	1.61	8.57E-04	0.0059
IL21R	1.61	2.24E-03	0.0102
B4GALT5	1.61	5.88E-04	0.0049
TXN2	1.61	4.51E-04	0.0043
DCPS	1.61	3.68E-04	0.0039
KCTD17	1.60	3.39E-03	0.0131
ENST000003213311	1.60	2.85E-03	0.0118
GNB1L	1.60	2.96E-03	0.0121
RPL7L1	1.60	7.22E-04	0.0054
GON7	1.60	3.28E-03	0.0128
TMX1	1.60	2.44E-04	0.0032
FAM173A	1.60	4.41E-03	0.0155
CLTA	1.60	1.15E-03	0.0069
FAM173B	1.60	1.37E-03	0.0077
ENST000002735412	1.60	1.24E-03	0.0073
MAGOHB	1.60	4.74E-04	0.0044
NUP188	1.60	5.68E-04	0.0048
PIPSL	1.60	1.23E-02	0.0311
ETFA	1.60	5.33E-04	0.0046
ENST000003151444	1.60	5.36E-04	0.0046
ENST000003409181	1.60	4.23E-04	0.0041
XRCC5	1.60	9.86E-04	0.0064
SLC19A2	1.60	1.76E-03	0.0089
TAF5	1.60	4.30E-04	0.0041
RPLP0	1.60	8.93E-05	0.0021

TSR3	1.59	2.17E-03	0.0100
CCDC77	1.59	1.50E-03	0.0081
SLC1A4	1.59	8.38E-03	0.0238
DNTTIP1	1.59	1.38E-03	0.0077
PGAP2	1.59	7.51E-03	0.0221
ENST000002526224	1.59	1.85E-03	0.0091
COPS3	1.59	6.49E-04	0.0051
KIAA0319	1.59	1.50E-02	0.0357
HDDC3	1.59	6.45E-04	0.0051
ELOB	1.59	1.07E-03	0.0067
ICA1L	1.59	4.90E-04	0.0044
GPR137	1.59	1.26E-02	0.0316
NUDT12	1.59	4.72E-03	0.0163
OGFOD1	1.59	7.71E-04	0.0056
EIF3C	1.59	2.30E-03	0.0104
ZSCAN20	1.59	1.18E-03	0.0071
NR1H3	1.59	1.77E-03	0.0089
HMGB1P5	1.59	7.17E-03	0.0214
THNSL1	1.59	7.56E-04	0.0055
ABCE1	1.59	3.39E-04	0.0037
ZNF826P	1.59	8.53E-04	0.0059
GABPB1	1.59	3.62E-04	0.0038
RPS19BP1	1.59	7.95E-04	0.0057
HLA-K	1.59	1.90E-03	0.0093
DEK	1.59	7.97E-04	0.0057
NAXE	1.59	2.76E-03	0.0116
TIMM8B	1.59	2.69E-03	0.0114
THAP3	1.59	1.25E-03	0.0073
ZNF219	1.59	3.94E-03	0.0144
SLC25A1	1.59	9.04E-04	0.0061
CSNK2B	1.59	3.65E-03	0.0137
RBM10	1.59	5.96E-04	0.0049
MCMBP	1.59	5.31E-04	0.0046
NDUFAF2	1.58	7.47E-04	0.0055
TMEM60	1.58	1.52E-03	0.0081
RDM1	1.58	6.04E-03	0.0191
HIST3H2A	1.58	3.89E-03	0.0143
AL603756.1	1.58	5.10E-03	0.0171
PLGRKT	1.58	6.26E-04	0.0050
PLEKHF1	1.58	8.70E-04	0.0060
ZSWIM3	1.58	3.34E-03	0.0130
ACADS	1.58	7.31E-04	0.0055
CDK6	1.58	1.93E-03	0.0094
WDR5	1.58	5.92E-04	0.0049
FBXW9	1.58	1.58E-03	0.0083
GNB2	1.58	3.26E-04	0.0037
ACTB	1.58	3.50E-04	0.0038

EMC6	1.58	2.49E-03	0.0109
VRK1	1.58	5.66E-04	0.0048
C16orf91	1.58	3.96E-03	0.0145
MRPS9	1.57	6.47E-04	0.0051
GLRX3	1.57	1.00E-03	0.0065
KDELR2	1.57	2.73E-04	0.0034
HSPA9	1.57	4.77E-04	0.0044
ENST000003331672	1.57	9.99E-03	0.0267
TOR4A	1.57	1.31E-02	0.0324
PSMC2	1.57	8.86E-04	0.0060
TELO2	1.57	1.67E-02	0.0385
PINX1	1.57	1.51E-03	0.0081
YWHAH	1.57	4.03E-04	0.0040
CTNS	1.57	1.20E-03	0.0071
AC009533.1	1.57	6.06E-04	0.0049
MYC	1.57	1.23E-03	0.0073
GOLPH3L	1.57	6.68E-04	0.0052
CYB5B	1.57	2.72E-03	0.0115
EIF5A	1.57	2.71E-03	0.0114
UTP6	1.57	6.12E-04	0.0050
ARMC1	1.57	3.71E-04	0.0039
ENG	1.57	1.43E-02	0.0346
NDUFA13	1.57	2.10E-03	0.0098
FBXL6	1.57	1.64E-03	0.0085
CTLA4	1.57	3.73E-03	0.0140
DYRK4	1.57	4.86E-04	0.0044
ADGRF3	1.57	2.08E-02	0.0452
ENST000003301371	1.57	3.05E-03	0.0123
PHETA2	1.57	4.33E-03	0.0154
EFL1	1.57	9.88E-04	0.0064
FAM129B	1.57	2.13E-03	0.0099
NCL	1.57	1.88E-03	0.0092
TXNL1	1.56	4.78E-04	0.0044
HMGN2	1.56	1.18E-03	0.0070
PPP1R26	1.56	5.16E-03	0.0172
PXN-AS1	1.56	1.42E-02	0.0345
HNRNPC	1.56	1.30E-04	0.0025
ENST000002657532	1.56	1.16E-03	0.0070
PRKCD	1.56	1.46E-03	0.0080
MRPL21	1.56	1.37E-03	0.0077
YEATS2	1.56	3.45E-04	0.0038
TERC	1.56	4.64E-03	0.0161
VPS35	1.56	4.59E-04	0.0043
ZMPSTE24	1.56	2.45E-03	0.0108
EIF4H	1.56	1.72E-04	0.0028
ENST000003535480	1.56	1.16E-03	0.0070
MMADHC	1.56	2.35E-04	0.0032

MYDGF	1.56	3.44E-04	0.0038
PLP2	1.56	1.24E-03	0.0073
UHRF1BP1	1.56	5.41E-04	0.0046
NIT1	1.56	5.82E-04	0.0048
HSD17B8	1.56	1.37E-03	0.0077
FOXP4	1.56	4.92E-03	0.0167
TIPRL	1.56	2.47E-04	0.0032
SLC25A22	1.56	3.63E-03	0.0137
LINC01146	1.56	2.06E-03	0.0097
SEPHS2	1.56	2.64E-03	0.0113
ATP1B1	1.56	8.58E-03	0.0241
ATP5F1A	1.56	2.21E-04	0.0031
C4orf33	1.56	4.28E-03	0.0153
EEF1AKMT1	1.56	1.35E-03	0.0076
CCDC137	1.56	1.20E-02	0.0304
ANP32B	1.55	3.27E-04	0.0037
WDYHV1	1.55	7.29E-04	0.0055
MYO19	1.55	3.18E-04	0.0037
C21orf59-TCP10L	1.55	7.50E-03	0.0221
GPD2	1.55	2.45E-03	0.0108
OTOF	1.55	1.41E-02	0.0343
ILK	1.55	3.84E-04	0.0039
PIGW	1.55	1.98E-02	0.0435
CARS	1.55	3.24E-04	0.0037
MID1	1.55	2.08E-02	0.0451
ARHGEF12	1.55	6.51E-03	0.0200
ARMCX3	1.55	3.63E-04	0.0038
MAFG	1.55	2.05E-03	0.0097
YBX3	1.55	1.08E-02	0.0283
KYAT3	1.55	3.33E-04	0.0037
LSM5	1.55	6.00E-04	0.0049
KMT5A	1.55	1.11E-03	0.0068
MRPS16	1.55	7.69E-04	0.0056
ENST00000639674	1.55	3.75E-03	0.0140
EIF3B	1.54	1.39E-03	0.0077
EFTUD2	1.54	4.43E-04	0.0042
DLAT	1.54	3.66E-04	0.0039
SLC25A6	1.54	1.12E-03	0.0068
SERPINB6	1.54	1.16E-03	0.0070
ENST000003582960	1.54	4.79E-03	0.0164
PSMC1	1.54	5.27E-04	0.0046
NDUFS2	1.54	6.44E-04	0.0051
KPNB1	1.54	2.08E-04	0.0030
SNRPD2	1.54	2.77E-04	0.0034
LINC00877	1.54	1.30E-02	0.0323
POLR2E	1.54	6.17E-04	0.0050
INSIG2	1.54	5.69E-03	0.0184

TEN1	1.54	1.38E-02	0.0336
USP5	1.54	4.78E-04	0.0044
ZMYND19	1.54	2.27E-03	0.0103
ENST000002912953	1.54	1.50E-03	0.0081
ENST000002859680	1.54	2.06E-04	0.0030
FANCC	1.54	8.19E-04	0.0058
ITGAE	1.54	1.44E-03	0.0079
ERF	1.54	2.72E-03	0.0115
PPIA	1.54	9.78E-04	0.0064
NIP7	1.54	2.13E-03	0.0099
TMEM256	1.54	7.18E-04	0.0054
DHRS4	1.54	7.76E-03	0.0226
DRG1	1.54	6.67E-04	0.0052
NOP2	1.54	1.25E-03	0.0073
SLC31A2	1.53	1.54E-03	0.0082
SPHK2	1.53	6.77E-03	0.0206
TUBA4A	1.53	2.21E-03	0.0101
ENST000003602400	1.53	2.34E-02	0.0491
OLA1	1.53	2.84E-04	0.0035
ADCY3	1.53	5.08E-04	0.0045
ENST000002925771	1.53	1.03E-03	0.0066
ENST000003068230	1.53	2.04E-03	0.0096
IFT140	1.53	1.35E-03	0.0076
RAVER1	1.53	2.72E-03	0.0115
NAT10	1.53	6.60E-04	0.0052
ENST000003134331	1.53	7.89E-03	0.0229
MLST8	1.53	1.12E-03	0.0068
PSMA7	1.53	1.69E-03	0.0087
POLH	1.53	5.22E-04	0.0045
MPHOSPH6	1.53	1.73E-03	0.0088
BCS1L	1.53	5.71E-04	0.0048
STYXL1	1.53	8.85E-03	0.0246
NUDT22	1.53	6.36E-04	0.0051
HMGCR	1.53	3.41E-04	0.0037
RRNAD1	1.53	2.03E-03	0.0096
USP1	1.53	1.02E-03	0.0065
CDK3	1.53	2.13E-02	0.0458
LSM3	1.53	1.71E-03	0.0087
MFSD14B	1.53	1.17E-03	0.0070
GLRX5	1.53	2.30E-03	0.0103
NDRG4	1.53	2.21E-02	0.0471
ENST000002525060	1.52	1.89E-02	0.0422
IFIT1	1.52	1.36E-02	0.0334
RECQL	1.52	5.22E-04	0.0045
ENST000003274350	1.52	4.12E-03	0.0149
TTC7A	1.52	1.23E-03	0.0072
MT-ND6	1.52	9.20E-03	0.0253

ENST00000535810	1.52	3.91E-03	0.0144
RRAGD	1.52	8.05E-04	0.0057
USP44	1.52	1.47E-03	0.0080
TMEM258	1.52	5.21E-04	0.0045
IFI16	1.52	8.84E-04	0.0060
CD40LG	1.52	2.22E-03	0.0101
CHD1L	1.52	7.78E-04	0.0056
MPP1	1.52	2.29E-02	0.0483
AC009159.3	1.52	1.17E-02	0.0298
AC090527.2	1.52	6.75E-03	0.0206
TRMT112	1.52	9.62E-03	0.0261
PDIA4	1.52	2.95E-04	0.0035
ACADVL	1.52	4.28E-04	0.0041
EML5	1.52	3.18E-04	0.0037
MTHFS	1.52	1.35E-03	0.0076
LEO1	1.52	9.64E-04	0.0063
AVEN	1.52	5.25E-03	0.0174
JAML	1.52	5.88E-04	0.0049
SPATS2L	1.51	4.33E-03	0.0154
MYB	1.51	6.30E-03	0.0196
ACYP1	1.51	5.70E-04	0.0048
UCHL5	1.51	3.24E-04	0.0037
NLE1	1.51	8.13E-04	0.0057
ATP5IF1	1.51	3.70E-04	0.0039
ZNF215	1.51	3.39E-03	0.0131
BMS1	1.51	9.58E-04	0.0063
NFKBIB	1.51	1.75E-03	0.0088
C17orf75	1.51	4.67E-04	0.0043
TMEM214	1.51	3.94E-03	0.0144
FDFT1	1.51	3.77E-04	0.0039
NUDT2	1.51	8.29E-04	0.0058
KIF1BP	1.51	4.26E-04	0.0041
ENST000003225350	1.51	2.70E-03	0.0114
FAM167A	1.51	1.06E-02	0.0279
GHITM	1.51	1.12E-03	0.0068
AL031985.3	1.51	5.44E-03	0.0178
SCAT1	1.51	1.54E-02	0.0363
BLOC1S1	1.51	9.11E-04	0.0061
IDH3A	1.51	5.76E-04	0.0048
RFTN1	1.51	3.02E-04	0.0036
GPSM1	1.51	1.39E-03	0.0077
NDUFA11	1.51	3.18E-03	0.0126
DOK1	1.51	1.69E-03	0.0087
STARD7	1.51	5.41E-04	0.0046
ENST000003155672	1.50	6.21E-03	0.0194
C14orf119	1.50	4.00E-04	0.0040
VDAC2	1.50	3.45E-04	0.0038

EDEM1	1.50	1.02E-03	0.0065
ENST000003212330	1.50	1.20E-02	0.0305
ADM2	1.50	8.42E-03	0.0238
PSMB7	1.50	8.74E-04	0.0060
ASNA1	1.50	3.30E-03	0.0129
GMPPA	1.50	2.54E-03	0.0110
BEND3	1.50	4.18E-03	0.0150
PPP1CC	1.50	6.51E-04	0.0051
GRN	1.50	3.92E-03	0.0144
NOC2L	1.50	1.30E-03	0.0074
DTD1	1.50	1.38E-03	0.0077
RRP12	1.50	2.99E-03	0.0121
RPP38	1.50	8.19E-04	0.0058
TOR3A	1.50	1.14E-03	0.0069
ENST000002994020	1.50	5.61E-03	0.0182
GRINA	1.50	1.53E-02	0.0363
ARV1	1.50	7.91E-04	0.0057
TOMM6	1.50	4.27E-03	0.0152
ENST000003260050	1.50	1.01E-03	0.0065
INTS13	1.50	4.78E-04	0.0044
TTF2	1.50	3.07E-04	0.0036
RBBP9	1.50	5.16E-04	0.0045
GNPAT	1.50	3.43E-04	0.0038
ENST000002823440	1.50	2.71E-04	0.0034
ENST000003589010	1.50	1.68E-03	0.0086
CCND2	1.49	2.49E-04	0.0033
ENST000003252120	1.49	3.13E-04	0.0036
ATF4	1.49	6.42E-04	0.0051
DDIT4	1.49	3.31E-04	0.0037
PFAS	1.49	3.25E-03	0.0128
ITSN1	1.49	4.99E-03	0.0168
ENST000003152862	1.49	9.88E-04	0.0064
KARS	1.49	2.91E-04	0.0035
VDAC3	1.49	2.62E-04	0.0033
SNRPC	1.49	2.10E-03	0.0098
RYBP	1.49	1.27E-03	0.0074
BCKDHA	1.49	8.44E-04	0.0059
FOXP3	1.49	5.80E-03	0.0186
ENST000003370180	1.49	4.34E-04	0.0042
SIRT6	1.49	2.10E-03	0.0098
ELOVL6	1.49	1.92E-03	0.0093
ENST000002891750	1.49	1.54E-02	0.0364
NDUFC2	1.49	4.66E-04	0.0043
AC004847.1	1.49	1.56E-02	0.0367
TCOF1	1.49	9.71E-04	0.0064
PHF13	1.49	8.24E-03	0.0235
VSIG10	1.49	1.75E-03	0.0089

MRPL10	1.49	1.90E-03	0.0093
TNPO2	1.49	4.30E-04	0.0041
RNF121	1.49	6.90E-04	0.0053
PPP1R16A	1.49	1.80E-03	0.0090
PCMT1	1.49	5.25E-04	0.0046
NOP14	1.48	6.27E-04	0.0050
MRPS36	1.48	4.76E-04	0.0044
KPTN	1.48	8.00E-03	0.0231
ENST000002939710	1.48	4.39E-03	0.0155
ENST000003273001	1.48	3.84E-03	0.0142
COASY	1.48	3.26E-03	0.0128
AC105339.2	1.48	1.13E-03	0.0069
ENST000003378590	1.48	5.06E-04	0.0045
ABCB7	1.48	1.49E-03	0.0081
ERLIN1	1.48	7.65E-04	0.0056
TMEM237	1.48	2.65E-03	0.0113
SLC27A4	1.48	8.46E-03	0.0239
HSPA5	1.48	3.47E-03	0.0133
CEP19	1.48	3.01E-03	0.0122
SV2A	1.48	1.31E-02	0.0324
CLTC	1.48	7.35E-04	0.0055
ACSS2	1.48	3.72E-03	0.0140
SERTAD1	1.48	3.59E-03	0.0136
FARP2	1.48	4.46E-03	0.0156
PRPF19	1.48	1.96E-03	0.0094
ENST000002924940	1.48	4.11E-03	0.0149
ENST000003220302	1.48	7.07E-04	0.0054
ENST000003146160	1.47	6.44E-04	0.0051
ENST000003106141	1.47	2.52E-03	0.0110
AZIN1-AS1	1.47	3.24E-03	0.0127
SLC16A3	1.47	5.32E-04	0.0046
SNORA80D	1.47	2.30E-02	0.0484
ENST000002511011	1.47	2.21E-03	0.0101
GTF2A2	1.47	2.59E-03	0.0111
ZNF146	1.47	2.46E-04	0.0032
SFR1	1.47	1.92E-03	0.0093
ACTN4	1.47	9.15E-04	0.0062
MLF2	1.47	4.20E-04	0.0041
IFI44	1.47	1.74E-02	0.0398
SETD9	1.47	5.47E-03	0.0179
SMIM13	1.47	3.67E-03	0.0138
ENST000002446702	1.47	2.28E-02	0.0481
DNAJC25	1.47	8.58E-04	0.0059
SYNGR2	1.47	1.68E-03	0.0086
NIF3L1	1.47	6.62E-04	0.0052
NDUFB5	1.47	6.77E-04	0.0052
AC024060.1	1.46	4.51E-03	0.0157

AC026401.3	1.46	1.98E-03	0.0095
AC243742.3	1.46	1.98E-03	0.0095
AP3S1	1.46	3.81E-04	0.0039
RPN1	1.46	3.80E-04	0.0039
DDX49	1.46	3.61E-03	0.0137
NUFIP1	1.46	1.35E-03	0.0076
YES1	1.46	7.54E-03	0.0222
CHID1	1.46	7.47E-04	0.0055
MT1X	1.46	2.64E-03	0.0113
RNF219	1.46	4.08E-04	0.0041
RAB13	1.46	1.83E-02	0.0412
PIGV	1.46	1.52E-03	0.0082
ZFP69B	1.46	3.13E-03	0.0125
PSME3	1.46	8.66E-04	0.0060
GFM2	1.46	1.04E-03	0.0066
TMEM109	1.46	5.57E-03	0.0181
RAPGEFL1	1.46	2.04E-02	0.0445
QDPR	1.46	2.64E-03	0.0113
GATM	1.46	3.74E-03	0.0140
BBS7	1.46	2.09E-03	0.0098
SINHCAF	1.46	2.64E-04	0.0034
NARS	1.46	3.99E-04	0.0040
ENST000002631211	1.46	1.65E-02	0.0381
TACC3	1.45	2.67E-03	0.0113
ENST000003206761	1.45	9.29E-04	0.0062
SNAPC4	1.45	1.57E-03	0.0083
NDUFAF4	1.45	2.06E-03	0.0097
PPP1R16B	1.45	4.79E-04	0.0044
GCA	1.45	7.71E-04	0.0056
EIF5B	1.45	6.64E-04	0.0052
UNC45A	1.45	3.94E-04	0.0040
UBE2Z	1.45	5.33E-04	0.0046
NDUFB11	1.45	1.50E-03	0.0081
TBCE	1.45	1.93E-03	0.0094
SAMD1	1.45	1.73E-02	0.0396
S100A13	1.45	8.14E-03	0.0233
ZNF710	1.45	1.11E-03	0.0068
AC009133.1	1.45	1.51E-02	0.0359
SNRNP40	1.45	1.32E-03	0.0075
LINC00471	1.45	2.76E-03	0.0116
WRB	1.45	2.58E-03	0.0111
ABRACL	1.45	2.30E-03	0.0104
IER3IP1	1.45	7.91E-04	0.0057
PALB2	1.45	4.16E-04	0.0041
TADA2A	1.45	1.54E-03	0.0082
TNNC1	1.45	8.13E-03	0.0233
FAM136A	1.45	4.77E-04	0.0044

ENST000002988182	1.45	1.78E-03	0.0089
SUMO1	1.45	7.14E-04	0.0054
MBOAT7	1.44	1.56E-03	0.0083
ATP1B3	1.44	3.28E-03	0.0128
DOT1L	1.44	2.24E-03	0.0102
RRAS	1.44	1.15E-02	0.0296
RARS	1.44	1.90E-03	0.0093
CCDC25	1.44	4.87E-04	0.0044
SND1	1.44	2.99E-04	0.0035
MAP2K1	1.44	3.32E-04	0.0037
APRT	1.44	1.63E-03	0.0085
GRPEL1	1.44	9.19E-04	0.0062
ENST000002632024	1.44	1.30E-03	0.0074
GTF2H3	1.44	9.36E-04	0.0062
PREB	1.44	1.10E-03	0.0068
CSNK2A1	1.44	2.72E-04	0.0034
TSPAN3	1.44	3.26E-03	0.0128
CMSS1	1.44	1.58E-03	0.0083
ENST000003449890	1.44	1.19E-03	0.0071
ARMC10	1.44	2.58E-03	0.0111
FANCM	1.44	3.68E-04	0.0039
CHAMP1	1.44	2.80E-03	0.0116
ENST000002902163	1.44	1.33E-02	0.0328
CRAT	1.44	1.01E-02	0.0269
ULBP1	1.43	2.24E-02	0.0476
SUPT4H1	1.43	8.61E-04	0.0059
ADAM8	1.43	1.08E-02	0.0283
ENST000002204292	1.43	2.68E-03	0.0114
STK32C	1.43	1.44E-03	0.0079
ZNF93	1.43	3.04E-03	0.0122
CHD4	1.43	4.49E-04	0.0042
ENST000003108640	1.43	6.56E-04	0.0051
ARHGEF5	1.43	3.68E-03	0.0138
U2AF2	1.43	7.36E-04	0.0055
CCDC127	1.43	1.06E-03	0.0066
SELENOH	1.43	9.57E-04	0.0063
URB2	1.43	3.99E-03	0.0146
C19orf70	1.43	1.10E-03	0.0068
RMND1	1.43	2.50E-03	0.0109
GYS1	1.43	2.96E-03	0.0121
THUMPD3	1.43	5.07E-04	0.0045
ENST000002648180	1.43	2.28E-02	0.0481
AC083899.1	1.43	1.78E-03	0.0089
PAQR7	1.43	5.58E-03	0.0181
AURKAIP1	1.43	5.13E-03	0.0171
TSPO	1.43	5.78E-03	0.0186
RNF19A	1.43	8.02E-04	0.0057

ITPA	1.43	2.46E-03	0.0108
ENST000001636781	1.43	3.41E-03	0.0131
NFKB2	1.43	6.00E-04	0.0049
COMT	1.43	2.85E-03	0.0118
CYCS	1.43	1.59E-03	0.0083
PPFIBP1	1.43	4.25E-03	0.0152
ENST000003433881	1.43	6.32E-03	0.0196
RRAGC	1.42	5.17E-04	0.0045
RAB8A	1.42	7.83E-04	0.0056
C12orf73	1.42	3.72E-03	0.0140
KATNB1	1.42	1.61E-03	0.0084
LRRC75A-AS1	1.42	3.90E-04	0.0040
INPP1	1.42	1.50E-03	0.0081
SRSF1	1.42	1.47E-03	0.0080
LINC00884	1.42	1.01E-02	0.0270
FP236241.1	1.42	1.92E-02	0.0427
ITGB1BP1	1.42	9.30E-04	0.0062
HAUS7	1.42	9.89E-04	0.0064
SNX11	1.42	1.96E-03	0.0094
TFR2	1.42	2.43E-03	0.0107
FAAP24	1.42	1.13E-03	0.0069
AL442125.1	1.42	1.88E-02	0.0419
BARX2	1.42	1.49E-02	0.0355
CARD19	1.42	2.09E-03	0.0098
PRADC1	1.42	2.25E-02	0.0477
ATP13A3	1.42	3.04E-04	0.0036
AC091982.3	1.42	1.03E-02	0.0274
AKR7A2	1.42	6.94E-04	0.0053
PPP1R12A-AS1	1.42	2.37E-02	0.0496
HSPA14	1.42	1.04E-03	0.0066
MKLN1-AS	1.42	8.33E-03	0.0237
DIMT1	1.42	4.59E-04	0.0043
AP2S1	1.42	2.29E-03	0.0103
RAB5IF	1.42	4.77E-03	0.0164
HIST1H3A	1.42	1.43E-03	0.0079
HNRNPA1P48	1.42	1.14E-02	0.0293
SLC35A2	1.42	3.37E-03	0.0131
ZBTB17	1.42	6.68E-04	0.0052
NMT1	1.42	5.75E-04	0.0048
SPTSSA	1.42	1.93E-03	0.0094
PTPA	1.42	6.47E-04	0.0051
AL160314.2	1.42	1.81E-02	0.0409
ENST000003557650	1.42	1.71E-03	0.0087
HNRNPA1P10	1.42	1.68E-02	0.0388
MCMDC2	1.42	7.51E-03	0.0221
USP37	1.42	4.94E-04	0.0044
URB1	1.41	4.19E-04	0.0041

PRKACA	1.41	1.73E-03	0.0088
APEX2	1.41	2.01E-03	0.0096
IER5	1.41	5.37E-04	0.0046
HIST1H1E	1.41	3.48E-04	0.0038
SHPK	1.41	1.73E-03	0.0088
ENST000002660871	1.41	7.34E-04	0.0055
AC108134.2	1.41	9.10E-03	0.0252
ENST000002688930	1.41	3.90E-03	0.0143
ABHD11	1.41	7.41E-03	0.0219
ENST000002730640	1.41	9.45E-04	0.0063
TFAM	1.41	2.16E-03	0.0099
ZNRD1	1.41	5.79E-04	0.0048
AKAP2	1.41	9.17E-04	0.0062
SMARCB1	1.41	7.36E-04	0.0055
SNAPIN	1.41	7.93E-03	0.0229
FAHD2A	1.41	1.66E-03	0.0086
NIPA2	1.41	2.55E-03	0.0111
SLC38A2	1.41	8.98E-04	0.0061
AC015813.2	1.41	3.25E-03	0.0128
AP1B1	1.41	1.62E-03	0.0085
HIST1H4E	1.41	5.81E-04	0.0048
SDHAF3	1.41	9.80E-04	0.0064
RHBDD3	1.40	2.19E-03	0.0100
KSR1	1.40	1.42E-02	0.0344
FERMT3	1.40	4.53E-04	0.0043
NXT1	1.40	2.22E-03	0.0101
ZNF598	1.40	1.12E-03	0.0068
ACACA	1.40	7.80E-04	0.0056
VCP	1.40	8.96E-04	0.0061
POLE	1.40	6.15E-03	0.0193
SCRIB	1.40	5.81E-03	0.0186
PPCDC	1.40	1.36E-03	0.0076
ACSL3	1.40	7.61E-04	0.0056
SUCLA2	1.40	9.57E-04	0.0063
ENST000003328580	1.40	7.34E-03	0.0217
CDC34	1.40	1.82E-03	0.0091
ENST000003323450	1.40	1.31E-02	0.0324
RBCK1	1.40	2.86E-03	0.0118
DAZAP1	1.40	6.63E-04	0.0052
TNKS1BP1	1.40	4.90E-03	0.0166
UMPS	1.40	1.70E-03	0.0087
IQCG	1.40	1.45E-03	0.0079
ASMTL	1.40	2.89E-03	0.0119
HAUS2	1.40	1.50E-03	0.0081
CTNNA1	1.40	1.17E-03	0.0070
APOL1	1.39	1.08E-03	0.0067
JRKL	1.39	1.06E-03	0.0067

RCC1L	1.39	2.38E-03	0.0106
TMEM192	1.39	5.79E-03	0.0186
FSD1	1.39	1.21E-02	0.0307
PTRH2	1.39	7.34E-04	0.0055
CYB5A	1.39	6.71E-03	0.0205
TCAF1P1	1.39	7.43E-03	0.0219
CETN4P	1.39	1.68E-02	0.0388
NIPAL1	1.39	2.11E-03	0.0098
TADA3	1.39	6.04E-03	0.0191
ENST000003077120	1.39	1.79E-03	0.0090
B4GAT1	1.39	3.76E-03	0.0140
APEH	1.39	4.72E-04	0.0044
DNAAF2	1.39	6.52E-03	0.0201
CRNDE	1.39	5.58E-03	0.0181
GTF2H2C_2	1.39	2.51E-03	0.0110
ENST000002964141	1.39	2.26E-03	0.0102
PRMT3	1.39	1.57E-03	0.0083
RABEPK	1.39	4.19E-03	0.0150
IRAK1	1.39	3.14E-03	0.0125
DCAF12	1.39	5.83E-04	0.0048
E2F6	1.38	1.90E-03	0.0093
ENST00000636126	1.38	1.74E-02	0.0398
FRMD4B	1.38	1.15E-03	0.0070
AC243587.1	1.38	9.00E-03	0.0249
HMGN2P5	1.38	9.00E-03	0.0249
ARF6	1.38	6.21E-04	0.0050
PSMB9	1.38	1.35E-03	0.0076
OXSM	1.38	1.19E-03	0.0071
GTF2F1	1.38	5.83E-04	0.0048
GPR171	1.38	4.53E-04	0.0043
PRPF31	1.38	6.84E-03	0.0207
SRP72	1.38	4.82E-04	0.0044
DCTD	1.38	1.02E-03	0.0065
TEX30	1.38	4.19E-03	0.0151
FBLN7	1.38	1.35E-02	0.0331
AC040970.1	1.38	1.84E-02	0.0415
ENST000003055700	1.38	5.49E-03	0.0179
RPL13P5	1.38	5.90E-03	0.0188
CD320	1.38	8.05E-03	0.0231
LENG9	1.38	7.85E-03	0.0228
CAMSAP1	1.38	5.74E-04	0.0048
MRPL53	1.38	1.09E-03	0.0067
LARP1	1.38	6.56E-04	0.0051
NARS2	1.38	1.05E-03	0.0066
AC004241.1	1.37	2.28E-02	0.0481
ACSL1	1.37	1.25E-03	0.0073
CCDC152	1.37	7.09E-03	0.0212

SLFN11	1.37	7.97E-04	0.0057
GGT1	1.37	1.26E-02	0.0317
RIOK1	1.37	1.42E-03	0.0078
HMGCL	1.37	1.02E-03	0.0065
RPP30	1.37	9.46E-04	0.0063
UBA2	1.37	7.23E-04	0.0054
PERP	1.37	7.64E-03	0.0223
PSMG2	1.37	5.86E-04	0.0048
HIKESHI	1.37	1.20E-03	0.0072
BPGM	1.37	2.29E-03	0.0103
PHF23	1.37	1.32E-03	0.0075
ENST000003359720	1.37	1.15E-02	0.0296
RPIA	1.37	1.64E-03	0.0085
ENST000002686680	1.37	7.71E-04	0.0056
SCYL1	1.37	9.34E-04	0.0062
NAB1	1.37	7.47E-04	0.0055
SERPINB1	1.37	2.12E-02	0.0458
GNA11	1.37	3.04E-03	0.0122
CXCL8	1.37	1.39E-02	0.0338
AHI1	1.37	2.07E-03	0.0097
ENST000002120150	1.37	6.87E-03	0.0208
ST20	1.37	1.02E-02	0.0272
SF3B5	1.37	8.78E-04	0.0060
SLC9A7	1.37	2.64E-03	0.0113
RAB8B	1.37	1.06E-03	0.0067
SNF8	1.37	2.98E-03	0.0121
ENST000002634610	1.37	1.90E-03	0.0093
HEMK1	1.37	5.11E-04	0.0045
ENST000003248711	1.37	2.30E-02	0.0485
PHYH	1.37	1.98E-03	0.0095
GPR108	1.37	2.50E-03	0.0109
MPV17L2	1.36	1.48E-02	0.0354
COMMD8	1.36	3.17E-03	0.0126
ARPC1B	1.36	9.52E-04	0.0063
CALR	1.36	1.29E-03	0.0074
ENST000003115021	1.36	4.56E-03	0.0159
MRPS24	1.36	7.06E-04	0.0054
FASTKD1	1.36	6.07E-04	0.0049
MTMR2	1.36	1.09E-03	0.0067
METTL26	1.36	2.30E-02	0.0484
TTF1	1.36	4.85E-04	0.0044
MTBP	1.36	7.30E-04	0.0055
TBRG4	1.36	1.08E-03	0.0067
NT5C3B	1.36	4.16E-03	0.0150
AC002310.1	1.36	4.43E-03	0.0156
PLEKHO2	1.36	3.23E-03	0.0127
SPACA6	1.36	5.07E-03	0.0170

SSNA1	1.36	4.46E-03	0.0156
C19orf24	1.36	1.16E-02	0.0298
LIPE	1.36	1.68E-02	0.0387
TIMMDC1	1.36	1.98E-03	0.0095
NAA15	1.36	8.28E-04	0.0058
PMEPA1	1.36	1.49E-02	0.0355
MRM2	1.35	5.48E-03	0.0179
LARP1B	1.35	5.46E-04	0.0046
MRPS27	1.35	4.44E-04	0.0042
TIMM17A	1.35	3.85E-03	0.0142
ENST000002457960	1.35	5.47E-04	0.0046
COX7B	1.35	6.56E-04	0.0051
DRAP1	1.35	2.56E-03	0.0111
PSENEN	1.35	2.55E-03	0.0111
SLC25A33	1.35	1.15E-02	0.0295
POLR1C	1.35	5.73E-03	0.0185
AMIGO2	1.35	9.49E-04	0.0063
DPM2	1.35	6.13E-03	0.0193
SH3BP1	1.35	4.09E-03	0.0148
ARPC1A	1.35	1.73E-03	0.0088
MRPL28	1.35	7.64E-03	0.0223
NOL10	1.35	2.09E-03	0.0098
ALKBH5	1.35	4.90E-04	0.0044
ENST000003328590	1.35	8.66E-03	0.0243
DLD	1.35	1.45E-03	0.0080
UBFD1	1.35	9.55E-04	0.0063
CASP2	1.35	9.55E-04	0.0063
SERPINH1	1.35	1.25E-03	0.0073
ENST000002517220	1.35	1.18E-02	0.0302
USP28	1.35	5.50E-04	0.0047
POLB	1.35	5.36E-04	0.0046
ATP6V1A	1.35	5.81E-04	0.0048
C11orf65	1.35	3.22E-03	0.0127
TMEM102	1.35	5.69E-03	0.0184
HAUS5	1.34	1.94E-03	0.0094
OSTC	1.34	5.24E-04	0.0045
COL27A1	1.34	2.85E-03	0.0118
AC020658.3	1.34	1.93E-02	0.0428
ZNF625	1.34	2.38E-03	0.0106
RAD23B	1.34	1.02E-03	0.0065
AC103706.1	1.34	1.83E-02	0.0413
CREB3L4	1.34	2.71E-03	0.0114
SFT2D1	1.34	1.22E-03	0.0072
GPATCH4	1.34	1.61E-02	0.0375
GTF3C4	1.34	7.63E-04	0.0056
USO1	1.34	5.08E-04	0.0045
TCTEX1D2	1.34	2.54E-03	0.0110

ENST000003018210	1.34	3.39E-04	0.0037
INTS7	1.34	7.76E-04	0.0056
HIST1H2BN	1.34	3.28E-03	0.0128
SNHG17	1.34	3.59E-03	0.0136
TNIP1	1.34	8.36E-04	0.0058
ZNF620	1.34	1.63E-02	0.0379
DCAF13	1.34	1.19E-03	0.0071
FICD	1.34	1.48E-03	0.0080
APOBEC3F	1.34	8.29E-03	0.0236
ECE1	1.34	1.05E-03	0.0066
GLB1	1.34	6.40E-04	0.0051
SECTM1	1.34	9.58E-03	0.0260
SLC35E4	1.34	1.28E-02	0.0318
CISD2	1.34	9.00E-03	0.0249
TOMM5	1.34	6.77E-03	0.0206
DDX54	1.34	1.05E-02	0.0277
XYLB	1.34	1.86E-02	0.0418
BAX	1.34	2.38E-02	0.0497
SEC24A	1.34	1.35E-03	0.0076
ORC6	1.34	6.29E-04	0.0050
UTP20	1.34	4.86E-03	0.0165
SGCB	1.33	2.66E-03	0.0113
AP2A1	1.33	1.52E-03	0.0081
IMPA1	1.33	5.45E-04	0.0046
ATP6V0B	1.33	5.49E-03	0.0179
TOR1A	1.33	1.04E-03	0.0066
TWSG1	1.33	2.22E-03	0.0101
SMC1A	1.33	5.86E-04	0.0048
PARL	1.33	1.76E-03	0.0089
LACC1	1.33	2.58E-03	0.0111
ENST000002765202	1.33	6.87E-03	0.0208
ZUP1	1.33	5.11E-04	0.0045
RND1	1.33	1.59E-02	0.0371
CXorf40B	1.33	2.56E-03	0.0111
ENST000003413691	1.33	7.88E-03	0.0228
ZC3HC1	1.33	2.59E-03	0.0112
ARL1	1.33	6.55E-04	0.0051
APOLD1	1.33	1.54E-03	0.0082
BTF3L4P2	1.33	6.26E-03	0.0195
SRRD	1.33	1.45E-02	0.0349
NOL7	1.33	1.33E-03	0.0075
CCDC117	1.33	8.26E-04	0.0058
NRAS	1.33	1.11E-03	0.0068
HAUS6	1.33	4.52E-04	0.0043
NEDD1	1.33	4.62E-04	0.0043
MED8	1.33	1.10E-03	0.0068
TMEM101	1.33	1.87E-03	0.0092

SZRD1	1.33	1.60E-03	0.0084
ZNF330	1.33	5.64E-04	0.0048
AC007728.3	1.33	1.01E-02	0.0270
HARS	1.33	9.40E-04	0.0063
RTCA	1.33	7.94E-04	0.0057
KIF1C	1.32	4.80E-03	0.0164
ENST000003513930	1.32	1.90E-02	0.0424
C5orf15	1.32	8.26E-04	0.0058
GPX4	1.32	8.88E-04	0.0060
PPP1CA	1.32	1.65E-03	0.0086
CCDC6	1.32	1.22E-03	0.0072
TIMM50	1.32	1.54E-03	0.0082
CPSF4	1.32	7.43E-04	0.0055
CEP85	1.32	8.70E-04	0.0060
ABI3	1.32	1.55E-02	0.0365
ENST000003552720	1.32	1.41E-03	0.0078
RHNO1	1.32	1.31E-03	0.0075
CALU	1.32	5.29E-04	0.0046
TRIT1	1.32	1.00E-03	0.0065
ANXA5	1.32	1.63E-03	0.0085
IARS2	1.32	8.99E-04	0.0061
MRPS18C	1.32	1.06E-03	0.0066
AP4M1	1.32	5.16E-03	0.0172
NEDD8	1.32	9.97E-04	0.0064
GFPT1	1.32	6.03E-04	0.0049
PRKAG1	1.32	2.03E-03	0.0096
ABCB10	1.32	3.55E-03	0.0135
SLC39A8	1.32	4.91E-04	0.0044
CTSB	1.32	1.84E-03	0.0091
PDK3	1.32	7.03E-04	0.0054
ENST000002428270	1.32	1.33E-02	0.0327
KLHL25	1.32	9.77E-03	0.0263
HADH	1.31	8.53E-04	0.0059
ANKRD33B	1.31	4.17E-03	0.0150
WDR43	1.31	1.60E-03	0.0084
RTEL1	1.31	6.53E-03	0.0201
KIF22	1.31	7.83E-04	0.0056
INTS2	1.31	5.77E-04	0.0048
RAD23A	1.31	4.43E-03	0.0156
EIPR1	1.31	1.19E-03	0.0071
SMC3	1.31	4.75E-04	0.0044
CCDC9	1.31	7.32E-03	0.0217
ZNF2	1.31	9.54E-03	0.0260
SNX33	1.31	3.40E-03	0.0131
PARD6A	1.31	1.05E-02	0.0277
UPRT	1.31	4.29E-03	0.0153
NDUFAF3	1.31	2.92E-03	0.0119

CEP76	1.31	9.68E-04	0.0064
SNAP29	1.31	8.47E-03	0.0239
PSMD1	1.31	8.69E-04	0.0060
MTX1	1.31	1.30E-03	0.0075
MRPS33	1.31	1.04E-03	0.0066
BAD	1.31	5.07E-03	0.0170
UBAC1	1.31	9.94E-04	0.0064
RBM15	1.31	5.76E-04	0.0048
C11orf74	1.31	1.29E-02	0.0322
LCP1	1.30	2.84E-04	0.0035
HYLS1	1.30	1.94E-03	0.0094
ENST000003300451	1.30	1.36E-02	0.0332
AC008443.1	1.30	1.25E-02	0.0314
ENST000002292682	1.30	7.92E-03	0.0229
ENST000003506380	1.30	8.20E-03	0.0234
LETM1	1.30	2.07E-03	0.0097
PGM1	1.30	8.38E-04	0.0058
INTS14	1.30	6.69E-04	0.0052
ENST000002161211	1.30	1.30E-03	0.0074
DHFR2	1.30	1.45E-03	0.0079
SLC35B1	1.30	3.07E-03	0.0123
LLGL1	1.30	2.13E-03	0.0099
GRSF1	1.30	7.73E-04	0.0056
MTMR4	1.30	1.85E-03	0.0092
CENPX	1.30	1.36E-02	0.0332
CCDC102A	1.30	3.39E-03	0.0131
ENST000003521711	1.30	5.56E-04	0.0047
RAB34	1.30	7.91E-03	0.0229
ENST000003567081	1.30	6.49E-04	0.0051
MFN2	1.30	2.35E-03	0.0105
RNF145	1.30	7.43E-04	0.0055
ABHD5	1.30	3.74E-03	0.0140
RCAN1	1.30	2.04E-03	0.0096
RBBP7	1.30	4.61E-04	0.0043
GNG10	1.30	3.32E-03	0.0129
ERO1A	1.30	8.43E-04	0.0059
C1orf198	1.30	6.43E-03	0.0199
CD40	1.30	2.10E-02	0.0455
ZNF212	1.30	2.74E-03	0.0115
PSMD7	1.30	2.45E-03	0.0108
SMG5	1.30	9.07E-04	0.0061
B3GNT10	1.30	2.29E-02	0.0483
TOB2	1.30	3.57E-03	0.0136
PPP4R2	1.30	6.69E-04	0.0052
PRPS1	1.30	6.64E-04	0.0052
YIPF2	1.29	2.14E-03	0.0099
AL354920.1	1.29	2.33E-02	0.0489

IFI30	1.29	3.21E-03	0.0127
TACO1	1.29	2.87E-03	0.0118
EIF2D	1.29	7.70E-04	0.0056
ENST000003104540	1.29	1.30E-03	0.0074
TGIF2-RAB5IF	1.29	1.78E-02	0.0405
RFT1	1.29	2.34E-03	0.0105
THAP4	1.29	1.35E-03	0.0076
PPP1R8	1.29	1.49E-03	0.0081
THOC7	1.29	8.81E-04	0.0060
IDH3B	1.29	7.01E-04	0.0054
SNHG15	1.29	8.35E-04	0.0058
SLC12A4	1.29	3.10E-03	0.0124
BOLA2	1.29	4.95E-03	0.0167
NFKB1	1.29	3.84E-04	0.0039
ENST000002722981	1.29	5.63E-03	0.0182
TBCA	1.29	2.21E-03	0.0101
RAB7A	1.28	7.94E-03	0.0230
CNDP2	1.28	1.49E-03	0.0081
TXNL4B	1.28	1.04E-03	0.0066
NOD2	1.28	7.34E-03	0.0217
PAK1	1.28	1.40E-03	0.0078
SF3B3	1.28	1.51E-03	0.0081
DHRS11	1.28	6.40E-03	0.0198
UBE2L3	1.28	2.80E-03	0.0117
LAS1L	1.28	1.30E-03	0.0074
CAPRIN1	1.28	7.07E-04	0.0054
KLHL17	1.28	6.34E-03	0.0197
ENST000003239970	1.28	2.23E-02	0.0474
SMG9	1.28	2.55E-03	0.0111
DBF4	1.28	8.19E-04	0.0058
TMEM126A	1.28	4.72E-03	0.0162
AP000845.1	1.28	1.83E-02	0.0412
LYPLA2	1.28	4.32E-03	0.0153
BMP1	1.28	1.37E-02	0.0335
LGALSL	1.28	2.02E-02	0.0442
ENST000003347051	1.28	1.12E-03	0.0068
WSB1	1.28	3.63E-03	0.0137
PUS1	1.28	4.43E-03	0.0156
KBTBD8	1.27	7.26E-04	0.0055
SLC25A39	1.27	1.38E-02	0.0337
PTMA	1.27	8.32E-03	0.0236
SNRPB2	1.27	2.13E-03	0.0099
TOP1MT	1.27	8.02E-03	0.0231
NUP35	1.27	8.71E-04	0.0060
ENST000003027460	1.27	6.56E-04	0.0051
ENST000002623182	1.27	2.41E-03	0.0107
BUD23	1.27	1.78E-03	0.0089

NELFB	1.27	1.06E-03	0.0066
LGALS9	1.27	8.73E-03	0.0244
MYO1C	1.27	7.22E-03	0.0215
ENST000003140451	1.27	2.09E-03	0.0098
SNUPN	1.27	2.22E-03	0.0101
ACTR1B	1.27	6.71E-04	0.0052
SOGA3	1.27	2.23E-02	0.0474
ENST000003613830	1.27	2.67E-03	0.0113
PLIN3	1.27	1.34E-03	0.0076
LRIF1	1.27	4.21E-03	0.0151
RNASEH2C	1.27	5.84E-03	0.0187
ZNF132	1.27	7.06E-03	0.0212
POLR2J	1.27	2.35E-03	0.0105
COPE	1.27	1.30E-03	0.0075
FAM200A	1.27	3.59E-03	0.0136
FUT11	1.27	1.28E-03	0.0074
UQCRFS1	1.26	1.28E-03	0.0074
ARMC9	1.26	1.46E-02	0.0350
ELOVL1	1.26	1.83E-03	0.0091
ATP5ME	1.26	3.20E-03	0.0126
LTA4H	1.26	7.73E-04	0.0056
ENST000003104411	1.26	2.55E-03	0.0111
ENST000003346510	1.26	2.59E-03	0.0111
COPS7A	1.26	1.10E-02	0.0286
NADK	1.26	3.69E-03	0.0139
SLC6A6	1.26	3.53E-03	0.0134
AFG3L2	1.26	1.59E-03	0.0084
PDZD11	1.25	2.27E-03	0.0103
MRPS28	1.25	1.39E-03	0.0078
NFS1	1.25	1.11E-03	0.0068
C8orf37	1.25	1.85E-02	0.0416
TAGLN2	1.25	1.53E-03	0.0082
PLAUR	1.25	1.10E-02	0.0287
EPN1	1.25	1.93E-02	0.0428
LMAN2	1.25	2.19E-03	0.0100
RBM8A	1.25	6.08E-03	0.0192
SUMO2	1.25	1.87E-03	0.0092
TTLL4	1.25	4.91E-03	0.0166
KLC2	1.25	2.70E-03	0.0114
ENST000002605851	1.25	1.05E-03	0.0066
DERA	1.25	3.20E-03	0.0126
MRPL41	1.25	1.23E-02	0.0310
ENST000002252981	1.25	1.64E-03	0.0085
MCOLN2	1.25	7.23E-04	0.0054
UBTD2	1.25	7.32E-03	0.0217
SPRYD7	1.25	8.54E-03	0.0241
SGPL1	1.25	3.13E-03	0.0125

ENDOG	1.25	1.23E-02	0.0310
NENF	1.25	2.38E-03	0.0106
MRPL52	1.25	2.51E-03	0.0110
TWISTNB	1.25	2.07E-03	0.0097
CCDC167	1.24	2.21E-03	0.0101
AC090825.1	1.24	4.42E-03	0.0155
ENST000002992750	1.24	1.03E-02	0.0274
TARS2	1.24	8.13E-04	0.0057
NUP85	1.24	9.53E-04	0.0063
RUSC1	1.24	3.52E-03	0.0134
PLRG1	1.24	9.72E-04	0.0064
ENST000003241980	1.24	1.29E-03	0.0074
ITGB3BP	1.24	2.90E-03	0.0119
CHCHD3	1.24	3.61E-03	0.0137
FNTB	1.24	6.06E-03	0.0191
MRPL46	1.24	1.28E-03	0.0074
IFT81	1.24	1.05E-02	0.0277
BCAP29	1.24	8.36E-04	0.0058
ZBTB9	1.24	5.66E-03	0.0183
ENST00000508524	1.24	1.94E-02	0.0429
TSFM	1.24	4.64E-03	0.0161
MRPS5	1.24	8.12E-04	0.0057
SUCLG1	1.24	8.91E-04	0.0061
SEC11C	1.24	1.51E-03	0.0081
FLII	1.24	9.48E-04	0.0063
SUZ12	1.24	6.25E-04	0.0050
KRT8P39	1.24	1.61E-02	0.0376
HEATR1	1.24	1.80E-03	0.0090
TRMT6	1.24	5.53E-03	0.0180
PAM16	1.24	1.14E-02	0.0293
TM9SF1	1.24	2.13E-03	0.0099
LSM6	1.24	3.78E-03	0.0141
SLC5A3	1.24	6.35E-03	0.0197
MAGEF1	1.24	4.40E-03	0.0155
LDHB	1.24	1.08E-03	0.0067
ENST000003419230	1.24	3.05E-03	0.0123
ENST000003261320	1.24	3.49E-03	0.0133
TTC33	1.24	7.18E-04	0.0054
ENST000002658491	1.24	2.99E-03	0.0121
ENST000001972682	1.24	6.11E-03	0.0192
MRRF	1.24	1.13E-03	0.0069
MMS22L	1.24	6.05E-04	0.0049
SF3A3	1.23	1.35E-03	0.0076
DPH2	1.23	8.38E-03	0.0238
WDR75	1.23	2.60E-03	0.0112
WDR36	1.23	1.31E-03	0.0075
SP140	1.23	1.10E-03	0.0068

SRGN	1.23	2.49E-03	0.0109
PSMD5	1.23	1.06E-03	0.0066
ENST000002644330	1.23	1.11E-03	0.0068
UNC119B	1.23	2.04E-03	0.0096
BAZ1B	1.23	7.57E-04	0.0055
INSR	1.23	8.40E-03	0.0238
EBPL	1.23	7.06E-03	0.0212
INAFM1	1.23	2.13E-02	0.0460
PWP1	1.23	3.98E-03	0.0145
GTSF1	1.23	1.82E-02	0.0410
H2AFV	1.23	1.55E-03	0.0083
ASCC1	1.23	2.47E-03	0.0108
KLHL12	1.23	6.72E-04	0.0052
MLLT1	1.23	5.87E-03	0.0187
QPCTL	1.23	7.43E-03	0.0219
FKBP2	1.23	1.80E-03	0.0090
PKIB	1.23	1.82E-02	0.0410
DHX30	1.23	8.22E-04	0.0058
CCDC138	1.23	1.18E-03	0.0071
SNORD13	1.23	9.30E-03	0.0255
RAE1	1.23	1.01E-03	0.0065
SBNO2	1.23	2.32E-03	0.0104
BAG4	1.23	1.60E-03	0.0084
KLHL6	1.23	4.50E-03	0.0157
FBXW11	1.23	5.70E-03	0.0184
PTBP1	1.23	2.67E-03	0.0113
ISOC1	1.23	1.36E-03	0.0076
HSDL2	1.23	1.66E-03	0.0086
TMX2	1.23	1.44E-03	0.0079
AAAS	1.23	6.31E-03	0.0196
HLTF	1.23	1.48E-03	0.0080
CKAP4	1.22	2.02E-03	0.0096
ALG8	1.22	1.04E-03	0.0066
TRIM16L	1.22	5.47E-03	0.0179
ZNF490	1.22	7.05E-03	0.0212
UBE2I	1.22	7.59E-04	0.0055
ALG3	1.22	3.87E-03	0.0143
ACO2	1.22	2.81E-03	0.0117
FBXO41	1.22	5.54E-03	0.0181
COX6B1	1.22	7.51E-04	0.0055
ENST000003122630	1.22	1.91E-03	0.0093
LSM10	1.22	2.82E-03	0.0117
ENST000002194731	1.22	2.02E-03	0.0096
ENST000002805570	1.22	1.19E-03	0.0071
CST7	1.22	1.98E-02	0.0435
ENST000003591910	1.22	1.20E-02	0.0305
DCTN2	1.22	1.28E-03	0.0074

DOCK3	1.22	9.92E-03	0.0266
RAB9A	1.22	2.58E-03	0.0111
SRP68	1.22	6.29E-03	0.0196
HDAC3	1.22	1.08E-03	0.0067
TATDN1	1.22	1.78E-03	0.0089
ENST000001934030	1.22	2.13E-02	0.0460
UAP1	1.22	3.46E-03	0.0133
IPMK	1.22	1.86E-03	0.0092
ANAPC1	1.22	6.57E-04	0.0052
TAF1D	1.22	1.37E-03	0.0077
PLA2G4C	1.22	4.01E-03	0.0146
FAM214B	1.22	1.50E-02	0.0357
WRN	1.22	1.44E-03	0.0079
SSR3	1.22	1.52E-03	0.0082
ANP32A	1.21	2.30E-03	0.0104
METTL2A	1.21	1.00E-02	0.0268
JAM3	1.21	1.66E-03	0.0086
AAGAB	1.21	1.88E-03	0.0092
NDUFS5	1.21	3.37E-03	0.0131
PHF5A	1.21	1.45E-03	0.0079
AKR1B1	1.21	1.51E-03	0.0081
ENST000002701420	1.21	1.21E-02	0.0307
AGMAT	1.21	1.86E-03	0.0092
EDC3	1.21	3.80E-03	0.0141
ID2	1.21	4.76E-03	0.0163
ENST000002617140	1.21	1.42E-03	0.0079
ARID3A	1.21	1.49E-02	0.0356
TRMT10A	1.21	2.63E-03	0.0112
TPRKB	1.21	3.23E-03	0.0127
LEPR	1.21	5.33E-03	0.0176
CEP57	1.21	7.79E-04	0.0056
CS	1.21	3.81E-03	0.0141
PEX12	1.21	4.51E-03	0.0158
MZT2B	1.21	3.28E-03	0.0128
L2HGDH	1.21	1.29E-03	0.0074
ATP6AP1L	1.21	3.51E-03	0.0134
ZSCAN21	1.21	2.95E-03	0.0120
CTDNEP1	1.20	3.84E-03	0.0142
GLS2	1.20	8.75E-03	0.0245
TRIM21	1.20	1.62E-03	0.0085
MRPS6	1.20	3.14E-03	0.0125
RBX1	1.20	1.75E-03	0.0089
TRMT10C	1.20	3.18E-03	0.0126
RASL11A	1.20	1.49E-02	0.0356
SNHG10	1.20	6.83E-03	0.0207
SKIL	1.20	1.03E-02	0.0272
C19orf53	1.20	3.77E-03	0.0140

ENST000002908580	1.20	1.70E-03	0.0087
EIF1AY	1.20	1.25E-03	0.0073
CENPBD1P1	1.20	2.04E-03	0.0096
BSG	1.20	5.99E-03	0.0190
ENST000003339871	1.20	5.98E-03	0.0190
ENST000003545030	1.20	1.36E-03	0.0076
MAGOH	1.20	2.53E-03	0.0110
FOXRED1	1.20	1.51E-03	0.0081
ASAH2B	1.20	7.09E-03	0.0212
EFNB1	1.20	1.45E-02	0.0349
FAF2	1.20	8.95E-04	0.0061
DPM1	1.20	2.40E-03	0.0106
AGPAT2	1.20	6.58E-03	0.0202
ENST000002479560	1.20	6.54E-03	0.0201
FOXRED2	1.20	2.08E-02	0.0451
CYBC1	1.20	1.09E-02	0.0284
SWAP70	1.20	9.54E-03	0.0260
MST1	1.20	1.37E-02	0.0334
FAM171B	1.20	7.42E-03	0.0219
MRPS31	1.20	1.34E-03	0.0076
CHKA	1.20	9.17E-03	0.0253
SDF4	1.20	1.97E-03	0.0095
TUBE1	1.20	1.85E-03	0.0091
WARS2	1.20	2.26E-03	0.0102
ZNF764	1.19	5.18E-03	0.0173
OPN3	1.19	3.35E-03	0.0130
MIS18BP1	1.19	1.08E-03	0.0067
MRM3	1.19	2.71E-03	0.0114
SYT11	1.19	1.29E-03	0.0074
RAB10	1.19	1.75E-03	0.0089
IPO4	1.19	1.71E-03	0.0087
NUP153	1.19	6.59E-04	0.0052
VKORC1	1.19	1.97E-03	0.0095
NDUFS4	1.19	1.46E-03	0.0080
REPIN1	1.19	3.77E-03	0.0140
NUP160	1.19	7.51E-04	0.0055
ELP6	1.19	1.46E-03	0.0080
XRCC1	1.19	4.99E-03	0.0168
TTC38	1.19	3.93E-03	0.0144
TRPT1	1.19	2.28E-02	0.0482
GTF3C3	1.19	7.96E-04	0.0057
AC026464.4	1.19	2.33E-03	0.0104
SENP3	1.19	1.34E-02	0.0329
PEX10	1.19	1.27E-02	0.0318
CSTF1	1.19	8.99E-04	0.0061
ARHGAP11B	1.19	2.04E-02	0.0445
TSPAN33	1.19	8.04E-03	0.0231

RELL2	1.19	7.14E-03	0.0213
AP1AR	1.19	2.44E-03	0.0107
NRROS	1.19	1.05E-02	0.0277
RRP1B	1.18	1.01E-03	0.0065
ATP6V0A2	1.18	1.91E-03	0.0093
CEP83	1.18	3.26E-03	0.0128
IMPDH1	1.18	4.74E-03	0.0163
PITRM1	1.18	4.49E-03	0.0157
ENST000003419800	1.18	3.18E-03	0.0126
DDOST	1.18	1.23E-03	0.0072
ENST000002525123	1.18	9.68E-04	0.0064
XRCC4	1.18	2.91E-03	0.0119
ZNF672	1.18	4.86E-03	0.0166
NTAN1	1.18	3.06E-03	0.0123
ENST000002284950	1.18	5.74E-03	0.0185
CMTM6	1.18	5.88E-03	0.0188
ZDHHC13	1.18	1.19E-03	0.0071
MBOAT2	1.18	7.88E-03	0.0228
ENST000003266390	1.18	8.20E-03	0.0234
GRAMD2B	1.18	3.05E-03	0.0123
DAPK3	1.18	2.79E-03	0.0116
C8orf76	1.18	2.78E-03	0.0116
CDC27	1.18	1.17E-03	0.0070
SCAMP3	1.18	1.68E-03	0.0086
DPY30	1.18	1.24E-03	0.0073
ZNF322	1.18	5.79E-03	0.0186
MAPK6	1.18	1.16E-02	0.0297
DEXI	1.18	2.20E-03	0.0101
IPPK	1.18	1.55E-03	0.0083
CNOT9	1.18	2.84E-03	0.0117
KIAA0753	1.18	8.19E-03	0.0234
ARL13B	1.17	7.06E-03	0.0212
ENST000003055360	1.17	1.26E-03	0.0073
INIP	1.17	3.93E-03	0.0144
CBX5	1.17	3.89E-03	0.0143
SNAPC2	1.17	1.24E-02	0.0312
SNORA79B	1.17	4.48E-03	0.0157
ACTR3	1.17	7.10E-04	0.0054
CLCN5	1.17	8.06E-03	0.0231
C1RL-AS1	1.17	6.64E-03	0.0203
PRXL2B	1.17	3.95E-03	0.0144
ENST000002596321	1.17	3.68E-03	0.0139
IL18R1	1.17	1.25E-02	0.0313
PPM1M	1.17	3.40E-03	0.0131
MIEF1	1.17	5.85E-03	0.0187
RNF8	1.17	1.79E-03	0.0090
AKT1	1.17	1.63E-03	0.0085

NPHP4	1.17	8.05E-03	0.0231
AC245060.4	1.17	4.77E-03	0.0164
POLR1E	1.17	2.03E-03	0.0096
COX7A2	1.17	2.76E-03	0.0116
DDX1	1.17	1.84E-03	0.0091
ARCN1	1.17	1.49E-03	0.0081
KIAA1958	1.17	1.06E-02	0.0280
NDUFS3	1.17	2.55E-03	0.0111
TRAFD1	1.17	1.76E-02	0.0401
CPSF2	1.17	2.05E-03	0.0096
TFB2M	1.17	6.28E-03	0.0195
ZNF35	1.16	1.56E-02	0.0367
MRPS11	1.16	3.73E-03	0.0140
CCDC144CP	1.16	1.04E-02	0.0275
TMEM205	1.16	1.12E-02	0.0291
IWS1	1.16	8.74E-04	0.0060
FAM27E3	1.16	1.06E-02	0.0278
VEGFB	1.16	6.36E-03	0.0197
DDX50	1.16	7.89E-04	0.0056
ERCC2	1.16	2.53E-03	0.0110
TXNDC9	1.16	5.81E-03	0.0186
FZD4	1.16	6.48E-03	0.0200
RHOA	1.16	7.17E-04	0.0054
PLAA	1.16	1.41E-03	0.0078
KLHDC8B	1.16	2.20E-02	0.0469
ARHGAP18	1.16	4.69E-03	0.0162
FAM118B	1.16	1.50E-02	0.0358
SMARCD2	1.16	1.29E-03	0.0074
ZNF250	1.16	1.36E-02	0.0334
CFL1	1.16	1.29E-02	0.0322
CHST10	1.16	9.39E-03	0.0257
MRPS10	1.16	5.05E-03	0.0170
TCF3	1.16	2.74E-03	0.0115
PPRC1	1.16	8.20E-03	0.0234
PLPBP	1.16	3.67E-03	0.0138
API5	1.16	1.46E-03	0.0080
TTC1	1.16	5.47E-03	0.0179
B3GNT2	1.16	9.60E-04	0.0063
GMPS	1.16	8.93E-04	0.0061
ENST000002777460	1.16	7.61E-03	0.0223
ENST000003044001	1.15	2.13E-02	0.0458
MCAM	1.15	4.41E-03	0.0155
C1orf216	1.15	7.57E-03	0.0222
PPIL3	1.15	1.09E-03	0.0068
SDHAF1	1.15	2.96E-03	0.0121
VKORC1L1	1.15	8.29E-03	0.0236
CYB5D1	1.15	4.78E-03	0.0164

TMEM39B	1.15	1.58E-02	0.0370
ENST000002682512	1.15	1.70E-02	0.0391
PDCD10	1.15	1.83E-03	0.0091
LINC01137	1.15	1.02E-02	0.0271
TNFRSF1B	1.15	2.86E-03	0.0118
AC106795.4	1.15	8.03E-03	0.0231
AP2M1	1.15	2.21E-03	0.0101
CHUK	1.15	3.00E-03	0.0122
STK3	1.15	2.84E-03	0.0117
FTSJ1	1.15	1.83E-03	0.0091
RXRA	1.15	8.14E-03	0.0233
PGAP1	1.15	2.04E-02	0.0445
MRPL33	1.15	4.38E-03	0.0155
EXTL2	1.15	5.51E-03	0.0180
RTL8A	1.15	2.36E-02	0.0494
LY96	1.15	7.61E-03	0.0223
ENST000003189743	1.15	1.93E-03	0.0094
MRPL30	1.15	2.80E-03	0.0116
UHRF1BP1L	1.14	5.86E-03	0.0187
IMP4	1.14	4.22E-03	0.0151
PIH1D1	1.14	6.38E-03	0.0198
CRLS1	1.14	3.01E-03	0.0122
CHEK2	1.14	1.67E-02	0.0385
BABAM1	1.14	4.34E-03	0.0154
IQGAP1	1.14	8.05E-04	0.0057
RAD50	1.14	1.12E-03	0.0068
LRWD1	1.14	2.39E-02	0.0499
NDUFS1	1.14	1.45E-03	0.0080
TIMM21	1.14	1.95E-03	0.0094
PCBP1	1.14	1.21E-03	0.0072
APOL2	1.14	1.00E-03	0.0065
PEX5	1.14	2.41E-03	0.0107
MED7	1.14	1.43E-03	0.0079
ENST000002311981	1.14	2.26E-03	0.0102
PPP6R1	1.14	2.11E-03	0.0098
ACTR1A	1.14	1.48E-03	0.0080
IRF2BPL	1.14	5.08E-03	0.0170
ARMC6	1.14	5.80E-03	0.0186
MANEA	1.14	1.42E-03	0.0079
ECHDC1	1.14	5.83E-03	0.0187
SCML2	1.14	1.62E-02	0.0377
HS6ST1	1.14	4.06E-03	0.0147
ST13	1.13	1.07E-03	0.0067
CDC23	1.13	9.96E-04	0.0064
C9orf16	1.13	3.72E-03	0.0140
RNASEH1	1.13	7.34E-03	0.0217
ATRIP	1.13	2.32E-03	0.0104

CHTF8	1.13	1.18E-03	0.0071
CEP41	1.13	2.44E-03	0.0107
TSEN15	1.13	2.43E-03	0.0107
TMEM268	1.13	9.65E-03	0.0262
TNPO3	1.13	1.08E-03	0.0067
ADO	1.13	1.67E-03	0.0086
COX7A2L	1.13	1.01E-03	0.0065
COX16	1.13	5.57E-03	0.0181
SCLY	1.13	9.67E-03	0.0262
ENST000002645150	1.13	6.65E-03	0.0204
AC005832.4	1.13	8.68E-03	0.0243
ABRAXAS2	1.13	1.29E-02	0.0322
COA3	1.13	1.14E-02	0.0293
CALML4	1.13	4.17E-03	0.0150
GSEC	1.13	8.72E-03	0.0244
MRPL34	1.13	1.49E-02	0.0356
LRRC40	1.13	1.56E-03	0.0083
E2F3	1.13	3.71E-03	0.0139
NECAB3	1.13	1.25E-02	0.0314
ENST000003180080	1.13	1.44E-03	0.0079
SEC61A1	1.13	1.18E-03	0.0071
DNAJC25-GNG10	1.13	3.80E-03	0.0141
HBS1L	1.12	8.60E-04	0.0059
RPAP3	1.12	2.62E-03	0.0112
IRGQ	1.12	2.41E-03	0.0107
NUP62CL	1.12	8.95E-03	0.0249
TOP3A	1.12	1.20E-03	0.0071
MPDU1	1.12	4.42E-03	0.0155
ENST000002576942	1.12	1.05E-02	0.0278
UAP1L1	1.12	1.51E-02	0.0359
C3orf18	1.12	3.17E-03	0.0126
SACS	1.12	2.15E-03	0.0099
LSM1	1.12	4.19E-03	0.0150
TFE3	1.12	1.55E-02	0.0365
TOMM70	1.12	1.67E-03	0.0086
NOC4L	1.12	1.06E-02	0.0279
XPNPEP1	1.12	2.27E-03	0.0102
AMZ2	1.12	1.55E-03	0.0083
UBL7	1.12	2.80E-03	0.0116
TMCO3	1.12	1.99E-03	0.0095
RIC8A	1.12	3.00E-03	0.0122
TGIF2	1.12	1.88E-03	0.0092
HSPB11	1.12	3.25E-03	0.0128
JTB	1.12	1.95E-03	0.0094
ENST000003124050	1.12	1.25E-02	0.0314
KCNAB2	1.12	2.85E-03	0.0118
AEBP1	1.12	1.74E-02	0.0398

ZNF616	1.12	7.24E-03	0.0215
DSTN	1.12	1.94E-03	0.0094
ZNF511	1.12	2.97E-03	0.0121
SLC25A32	1.12	5.53E-03	0.0180
TCEAL8	1.12	2.46E-03	0.0108
COPB1	1.12	7.67E-04	0.0056
SDHD	1.11	1.81E-03	0.0090
RSL24D1	1.11	1.02E-03	0.0065
AC073529.1	1.11	1.05E-02	0.0277
CRIM1	1.11	5.71E-03	0.0184
EED	1.11	2.07E-03	0.0097
SARS2	1.11	8.51E-03	0.0240
EXOSC1	1.11	3.50E-03	0.0134
CCP110	1.11	1.80E-03	0.0090
PFKFB3	1.11	1.49E-02	0.0356
PPP4C	1.11	1.39E-03	0.0077
ZC3H12D	1.11	4.31E-03	0.0153
FASTKD3	1.11	6.07E-03	0.0192
MTRF1L	1.11	1.66E-03	0.0086
GNL3L	1.11	5.64E-03	0.0183
WDR83OS	1.11	2.92E-03	0.0119
EXOSC7	1.11	1.96E-03	0.0094
ENST000002643121	1.11	1.50E-03	0.0081
DNASE2	1.11	2.14E-03	0.0099
SMIM20	1.11	2.61E-03	0.0112
XYLT2	1.11	2.29E-03	0.0103
NAA50	1.11	8.35E-03	0.0237
ZNF100	1.10	3.95E-03	0.0145
ZNF79	1.10	2.34E-02	0.0492
DDX55	1.10	3.41E-03	0.0131
ENST000003136010	1.10	3.17E-03	0.0126
BMF	1.10	1.60E-02	0.0374
ATP6V1F	1.10	3.79E-03	0.0141
CHMP4B	1.10	1.89E-03	0.0092
ARPC5	1.10	1.25E-03	0.0073
ORC3	1.10	1.70E-03	0.0087
LDAH	1.10	1.53E-03	0.0082
MT-ND4	1.10	3.58E-03	0.0136
ENST000002682061	1.10	2.50E-03	0.0109
EIF2AK2	1.10	2.75E-03	0.0116
BLOC1S2	1.10	2.06E-03	0.0097
TJP2	1.10	2.34E-03	0.0105
DDRGK1	1.10	1.81E-03	0.0090
NME4	1.10	3.71E-03	0.0139
GCN1	1.10	1.67E-03	0.0086
ARF1	1.10	2.46E-03	0.0108
FRG1	1.10	1.85E-03	0.0091

PFKL	1.10	1.49E-03	0.0081
SLA2	1.10	3.10E-03	0.0124
SLA	1.10	5.37E-03	0.0177
UBA6	1.10	1.12E-03	0.0068
SMN2	1.10	2.16E-02	0.0464
COX5B	1.10	1.67E-03	0.0086
FAM3C2	1.10	2.28E-02	0.0483
SIT1	1.10	7.14E-03	0.0213
AC010132.3	1.10	1.36E-02	0.0332
CENPK	1.10	3.66E-03	0.0138
EIF2AK4	1.10	1.15E-03	0.0070
ENST000002878993	1.10	5.56E-03	0.0181
YIPF5	1.10	1.61E-03	0.0084
ARHGEF2	1.10	1.54E-03	0.0082
POLR2B	1.10	1.03E-03	0.0066
WDR77	1.10	1.99E-02	0.0437
LRRC8B	1.10	1.27E-03	0.0073
ENST000002850934	1.09	9.50E-03	0.0259
SLC4A2	1.09	4.10E-03	0.0148
ENST000002806650	1.09	1.09E-02	0.0285
GPN1	1.09	3.17E-03	0.0126
MGMT	1.09	3.62E-03	0.0137
ENST000002660140	1.09	7.75E-03	0.0226
PANX1	1.09	6.71E-03	0.0205
PRKCI	1.09	2.07E-03	0.0097
ANKRD54	1.09	4.89E-03	0.0166
NAE1	1.09	1.96E-03	0.0094
ATP5PO	1.09	1.96E-03	0.0094
GSKIP	1.09	2.62E-03	0.0112
SRA1	1.09	5.83E-03	0.0187
MFSD5	1.09	9.82E-03	0.0264
DLG4	1.09	5.76E-03	0.0185
NEU1	1.09	2.31E-03	0.0104
MMAB	1.09	3.44E-03	0.0132
ELAC2	1.09	4.34E-03	0.0154
ENST000001849560	1.09	6.21E-03	0.0194
TNFSF15	1.09	2.29E-02	0.0483
CCDC71L	1.09	3.18E-03	0.0126
ENST000002549502	1.09	6.79E-03	0.0206
EXOSC3	1.09	2.36E-03	0.0105
RMDN1	1.09	1.38E-03	0.0077
TRUB1	1.09	3.70E-03	0.0139
CAPZA1	1.09	1.25E-03	0.0073
DCAF7	1.09	1.88E-03	0.0092
FECH	1.08	2.69E-03	0.0114
ZNF443	1.08	5.82E-03	0.0186
PEF1	1.08	2.21E-03	0.0101

ECH1	1.08	1.62E-03	0.0085
WDR4	1.08	8.14E-03	0.0233
KRT8P12	1.08	2.26E-02	0.0478
USP49	1.08	1.07E-02	0.0280
NDUFA4	1.08	3.54E-03	0.0135
BIVM	1.08	4.25E-03	0.0152
UPF3B	1.08	3.16E-03	0.0125
EXD2	1.08	2.22E-03	0.0101
SNORA73A	1.08	1.44E-02	0.0348
TXNL4A	1.08	2.97E-03	0.0121
RBM19	1.08	3.52E-03	0.0134
SIL1	1.08	2.66E-03	0.0113
ENST000003253663	1.08	1.75E-02	0.0398
ENST000003142892	1.08	1.59E-03	0.0084
ARMH1	1.08	1.36E-02	0.0333
ENST000003409410	1.08	3.24E-03	0.0127
PAPSS1	1.08	2.12E-03	0.0099
ENST000002168071	1.08	1.35E-02	0.0332
SNRNP27	1.08	1.97E-03	0.0094
NDUFA2	1.08	3.32E-03	0.0129
MLX	1.08	3.53E-03	0.0134
RPS10-NUDT3	1.08	4.37E-03	0.0155
TMEM53	1.07	1.22E-02	0.0308
POLR2D	1.07	2.96E-03	0.0121
BABAM2	1.07	4.02E-03	0.0146
TBC1D31	1.07	2.76E-03	0.0116
MTPAP	1.07	7.75E-03	0.0226
PIGU	1.07	8.22E-03	0.0234
ENST000003814010	1.07	4.19E-03	0.0151
PMPCA	1.07	3.22E-03	0.0127
POLR2K	1.07	4.58E-03	0.0159
INPPL1	1.07	2.93E-03	0.0120
ANKLE2	1.07	1.04E-03	0.0066
TM7SF2	1.07	6.85E-03	0.0208
ARPC2	1.07	1.38E-03	0.0077
TM9SF4	1.07	2.60E-03	0.0112
ENST000000081803	1.07	6.76E-03	0.0206
ENST000002658661	1.07	9.11E-03	0.0252
UQCR10	1.07	4.52E-03	0.0158
PRDX6	1.07	1.67E-03	0.0086
GBP2	1.07	1.77E-03	0.0089
IFITM3	1.07	1.30E-02	0.0323
ENST000003248682	1.07	2.73E-03	0.0115
CAP1	1.07	1.13E-03	0.0069
AC022415.1	1.07	2.26E-02	0.0478
SVIL-AS1	1.07	3.50E-03	0.0134
CSTF3	1.07	3.75E-03	0.0140

ENST000003358952	1.07	2.93E-03	0.0120
PI4K2A	1.06	1.48E-02	0.0353
ANAPC7	1.06	6.52E-03	0.0201
CLNS1A	1.06	1.06E-03	0.0066
DNAJC6	1.06	2.38E-02	0.0497
ATP1A1-AS1	1.06	1.32E-02	0.0327
PTGES3P2	1.06	2.37E-02	0.0496
RF00004.10	1.06	1.56E-03	0.0083
RF00004.11	1.06	1.56E-03	0.0083
RF00004.12	1.06	1.56E-03	0.0083
RF00004.15	1.06	1.56E-03	0.0083
RF00004.16	1.06	1.56E-03	0.0083
RF00004.19	1.06	1.56E-03	0.0083
RF00004.2	1.06	1.56E-03	0.0083
RF00004.3	1.06	1.56E-03	0.0083
RF00004.5	1.06	1.56E-03	0.0083
RF00004.9	1.06	1.56E-03	0.0083
RNU2-1	1.06	1.56E-03	0.0083
BLVRA	1.06	4.33E-03	0.0154
CHST3	1.06	2.15E-02	0.0463
GTF2H2B	1.06	1.73E-02	0.0396
ENST000002964110	1.06	1.99E-03	0.0095
CRTAP	1.06	4.13E-03	0.0149
MCRIP1	1.06	3.87E-03	0.0143
ENST000002225110	1.06	2.96E-03	0.0121
RPS2	1.06	2.07E-03	0.0097
FAM20B	1.06	2.42E-03	0.0107
ESF1	1.06	2.26E-03	0.0102
C8orf33	1.06	2.73E-03	0.0115
NDUFC1	1.06	5.30E-03	0.0175
CORO1A	1.06	1.60E-03	0.0084
TRIAP1	1.06	2.39E-03	0.0106
EIF1AX	1.06	8.44E-03	0.0239
NUDT19	1.06	2.65E-03	0.0113
PAXIP1	1.06	2.85E-03	0.0118
GTDC1	1.06	6.20E-03	0.0194
KPNA3	1.06	1.63E-03	0.0085
ATP5MPL	1.06	2.67E-03	0.0113
MIIP	1.06	2.91E-03	0.0119
NSUN5	1.06	3.37E-03	0.0131
NELFE	1.06	6.72E-03	0.0205
MRPL44	1.06	1.94E-03	0.0094
TCEAL4	1.06	1.31E-02	0.0324
CFAP298	1.06	8.27E-03	0.0236
MRPL57	1.06	5.45E-03	0.0178
ENST000003283920	1.06	9.24E-03	0.0254
CAND1	1.06	9.87E-04	0.0064

SMUG1	1.06	4.54E-03	0.0158
MALSU1	1.05	3.38E-03	0.0131
TYW3	1.05	1.89E-02	0.0422
RRP8	1.05	6.55E-03	0.0201
UQCRC2	1.05	1.98E-03	0.0095
E2F4	1.05	2.76E-03	0.0116
SART1	1.05	3.15E-03	0.0125
DDX23	1.05	2.41E-03	0.0107
ACO1	1.05	2.80E-03	0.0116
CDC37	1.05	5.99E-03	0.0190
ENST000003120490	1.05	1.14E-02	0.0293
SS18L2	1.05	1.29E-02	0.0320
POC5	1.05	2.70E-03	0.0114
CEP295	1.05	4.90E-03	0.0166
ZDHHC18	1.05	2.55E-03	0.0111
GSR	1.05	3.31E-03	0.0129
STX10	1.05	1.99E-03	0.0095
AL157394.1	1.05	2.25E-02	0.0477
LXN	1.05	4.89E-03	0.0166
XPO1	1.05	4.56E-03	0.0159
TTC27	1.05	3.37E-03	0.0131
ATG4C	1.05	2.58E-03	0.0111
LIG1	1.05	3.88E-03	0.0143
STAT3	1.05	1.00E-02	0.0269
HIST1H1D	1.05	2.36E-03	0.0105
GTPBP2	1.05	2.08E-02	0.0451
MTA2	1.05	1.69E-03	0.0087
ENST000001993890	1.05	2.17E-03	0.0100
ALDH6A1	1.05	7.62E-03	0.0223
PPME1	1.04	7.82E-03	0.0227
ACOX1	1.04	5.69E-03	0.0184
APOPT1	1.04	2.44E-03	0.0108
IPP	1.04	9.02E-03	0.0250
RLN2	1.04	1.02E-02	0.0271
KIF21A	1.04	3.98E-03	0.0145
NEIL2	1.04	4.01E-03	0.0146
DDX18	1.04	3.85E-03	0.0142
PUF60	1.04	1.60E-02	0.0373
DAPP1	1.04	5.25E-03	0.0174
SGPP2	1.04	6.19E-03	0.0194
ZNF486	1.04	1.92E-03	0.0093
MYBBP1A	1.04	4.41E-03	0.0155
SMAD1	1.04	1.63E-02	0.0378
ZNF594	1.04	1.34E-02	0.0330
CPD	1.04	6.30E-03	0.0196
TMED2	1.04	1.84E-03	0.0091
RTN4IP1	1.04	6.58E-03	0.0202

DBNDD2	1.04	6.37E-03	0.0197
NAA10	1.04	3.71E-03	0.0139
TDP1	1.04	1.56E-03	0.0083
CAMK2D	1.04	1.56E-03	0.0083
MROH8	1.04	2.29E-02	0.0483
ALKBH3	1.04	8.03E-03	0.0231
TPM3	1.04	4.63E-03	0.0160
FLNA	1.04	1.01E-03	0.0065
PPP2CB	1.04	4.93E-03	0.0167
DFFA	1.04	9.39E-03	0.0257
AGAP5	1.04	8.83E-03	0.0246
RIOK2	1.04	3.90E-03	0.0143
STAT1	1.04	8.47E-03	0.0239
ALKBH1	1.04	3.38E-03	0.0131
ACSL4	1.04	7.37E-03	0.0218
WDR41	1.04	1.79E-03	0.0090
NMD3	1.04	1.50E-03	0.0081
SYNCRIP	1.04	1.57E-02	0.0370
SEC23IP	1.04	2.53E-03	0.0110
WDR62	1.04	1.80E-02	0.0407
KDELR1	1.04	2.06E-02	0.0448
SLC30A5	1.04	4.98E-03	0.0168
LTV1	1.03	1.30E-02	0.0322
AL161891.1	1.03	3.82E-03	0.0141
MPC2	1.03	3.65E-03	0.0138
EDARADD	1.03	2.31E-02	0.0486
SNX4	1.03	3.89E-03	0.0143
ENST000003249321	1.03	1.32E-03	0.0075
GNAI3	1.03	1.57E-03	0.0083
KRI1	1.03	3.24E-03	0.0127
TRAPPC4	1.03	1.65E-02	0.0383
PTGES2	1.03	1.05E-02	0.0276
DUSP2	1.03	2.63E-03	0.0113
CRKL	1.03	1.32E-03	0.0075
VTA1	1.03	2.79E-03	0.0116
MBD2	1.03	7.75E-03	0.0226
TTI1	1.03	2.38E-03	0.0106
EIF3D	1.03	1.78E-03	0.0089
ADCK2	1.03	4.19E-03	0.0150
IFT122	1.03	4.30E-03	0.0153
CEP250	1.03	1.96E-03	0.0094
ENST000003583141	1.03	9.98E-03	0.0267
C22orf46	1.03	4.33E-03	0.0154
TRIM65	1.03	7.43E-03	0.0220
ZFP57	1.03	1.21E-02	0.0308
AUH	1.03	9.57E-03	0.0260
EXOSC2	1.03	2.20E-03	0.0101

NIPSNAP2	1.03	3.31E-03	0.0129
SART3	1.03	2.25E-03	0.0102
TMEM14B	1.03	4.40E-03	0.0155
KDSR	1.03	1.84E-03	0.0091
JARID2	1.03	2.62E-03	0.0112
FAM104B	1.03	9.43E-03	0.0257
VASP	1.02	4.81E-03	0.0164
MED19	1.02	1.08E-02	0.0282
ENST000002642540	1.02	1.31E-02	0.0324
MRGBP	1.02	3.37E-03	0.0131
MYL6	1.02	7.28E-03	0.0216
RWDD4	1.02	5.32E-03	0.0175
ENST000002678840	1.02	1.36E-02	0.0332
MAK16	1.02	1.24E-02	0.0311
LIG4	1.02	5.97E-03	0.0190
GANAB	1.02	2.84E-03	0.0117
ENST000003311340	1.02	8.04E-03	0.0231
TRUB2	1.02	1.69E-02	0.0389
HNRNPL	1.02	1.95E-03	0.0094
SNHG12	1.02	4.59E-03	0.0159
PLIN2	1.02	6.49E-03	0.0200
GAS5	1.02	1.94E-03	0.0094
NCKAP1L	1.02	1.57E-03	0.0083
OXSR1	1.02	4.89E-03	0.0166
ZNF267	1.02	5.19E-03	0.0173
STK26	1.02	1.51E-03	0.0081
QSOX1	1.02	8.21E-03	0.0234
NDUFB1	1.02	6.49E-03	0.0200
BTBD1	1.02	1.70E-03	0.0087
EAF2	1.02	2.14E-02	0.0460
METTL6	1.02	6.20E-03	0.0194
LETM2	1.02	6.18E-03	0.0194
NPRL3	1.02	2.22E-02	0.0473
PTGR2	1.02	5.81E-03	0.0186
OTUD6B	1.02	4.95E-03	0.0167
CIAPIN1	1.02	6.87E-03	0.0208
CLTB	1.02	1.58E-02	0.0371
SRI	1.02	1.85E-03	0.0091
THYN1	1.02	1.68E-03	0.0086
DUS2	1.02	1.45E-02	0.0350
TSPAN5	1.02	2.69E-03	0.0114
CTSZ	1.01	7.14E-03	0.0213
CHST2	1.01	6.74E-03	0.0205
MAT2A	1.01	1.87E-02	0.0419
SERF2	1.01	2.52E-03	0.0110
ENST000003049920	1.01	3.00E-03	0.0122
OSGIN2	1.01	3.32E-03	0.0129

SLC9B2	1.01	4.33E-03	0.0154
MGAT2	1.01	3.19E-03	0.0126
SLC25A44	1.01	2.09E-02	0.0452
MIOS	1.01	1.97E-03	0.0095
VPS72	1.01	2.45E-03	0.0108
ENST000003129620	1.01	2.73E-03	0.0115
IMPAD1	1.01	2.03E-03	0.0096
COX6C	1.01	2.31E-03	0.0104
HSP90B1	1.01	1.84E-03	0.0091
SH3BGR	1.01	2.27E-02	0.0481
TOR1B	1.01	2.75E-03	0.0115
ENST000002803302	1.01	1.28E-02	0.0319
UCHL3	1.01	3.32E-03	0.0129
CCDC124	1.01	8.20E-03	0.0234
FBXO4	1.01	9.93E-03	0.0266
C6orf62	1.01	2.65E-03	0.0113
ENST000002288251	1.00	4.84E-03	0.0165
KIAA0100	1.00	3.73E-03	0.0140
GTF2E2	1.00	3.34E-03	0.0130
TRIM27	1.00	2.17E-03	0.0100
CD2	1.00	2.07E-03	0.0097
LINC00526	1.00	1.95E-02	0.0431
NDOR1	1.00	1.40E-02	0.0340
NDUFS7	1.00	9.70E-03	0.0262
LPCAT1	1.00	2.23E-02	0.0475
EIF4E	1.00	5.39E-03	0.0177
PRMT6	1.00	4.69E-03	0.0162
BICDL1	1.00	8.21E-03	0.0234
FAAP100	1.00	1.03E-02	0.0272
PEX3	1.00	2.63E-03	0.0113
SPPL2A	1.00	4.31E-03	0.0153
POLR3A	1.00	2.84E-03	0.0117
CEP44	1.00	2.77E-03	0.0116
MYO1G	1.00	1.90E-03	0.0093
GPR107	1.00	4.68E-03	0.0162
RSL1D1	1.00	4.95E-03	0.0167
ZNF691	1.00	9.47E-03	0.0258
GTF2F2	1.00	6.48E-03	0.0200
NUCKS1	1.00	2.41E-03	0.0107
SDHAF4	1.00	4.74E-03	0.0163
TOLLIP	1.00	1.07E-02	0.0281
CANT1	1.00	3.80E-03	0.0141
TOP1	1.00	2.06E-02	0.0449
GFER	0.99	6.84E-03	0.0207
SCO1	0.99	3.45E-03	0.0132
IL32	0.99	6.34E-03	0.0197
ASNSD1	0.99	2.79E-03	0.0116

TMEM141	0.99	2.27E-02	0.0481
PSMC5	0.99	6.85E-03	0.0208
MIR17HG	0.99	3.13E-03	0.0125
KRT18	0.99	1.76E-02	0.0400
ENST000003134211	0.99	2.42E-03	0.0107
UTP4	0.99	5.10E-03	0.0171
BTN2A2	0.99	6.66E-03	0.0204
DLEU2	0.99	4.55E-03	0.0159
COTL1	0.99	1.82E-03	0.0091
PSMD9	0.99	2.46E-03	0.0108
PMM1	0.99	8.07E-03	0.0232
SERAC1	0.99	1.15E-02	0.0295
RNF215	0.99	8.44E-03	0.0239
EXOSC9	0.99	2.88E-03	0.0118
LINC01588	0.99	4.39E-03	0.0155
BAG2	0.99	3.19E-03	0.0126
NUP133	0.99	3.02E-03	0.0122
AGO2	0.99	1.04E-02	0.0275
MED24	0.99	4.36E-03	0.0154
PGS1	0.99	1.64E-02	0.0381
CLPX	0.99	1.87E-03	0.0092
PROCR	0.99	2.23E-02	0.0475
TNFRSF10B	0.98	7.17E-03	0.0214
JAGN1	0.98	8.54E-03	0.0241
TESK1	0.98	1.26E-02	0.0316
DHX58	0.98	1.48E-02	0.0355
SLAMF7	0.98	1.38E-02	0.0336
HOMEZ	0.98	7.29E-03	0.0217
ZNF395	0.98	8.01E-03	0.0231
KCTD6	0.98	6.12E-03	0.0193
MTX2	0.98	2.37E-02	0.0496
DNAJC8	0.98	3.65E-03	0.0138
KAT2A	0.98	1.37E-02	0.0334
GBAP1	0.98	1.66E-02	0.0384
BNIP1	0.98	8.92E-03	0.0248
NET1	0.98	4.00E-03	0.0146
VEZT	0.98	3.45E-03	0.0132
COMMD5	0.98	5.85E-03	0.0187
SPAG7	0.98	3.04E-03	0.0122
NFYB	0.98	8.21E-03	0.0234
RRS1	0.98	6.24E-03	0.0195
ATP5MC2	0.98	3.18E-03	0.0126
GCH1	0.98	2.14E-03	0.0099
ENST000002219220	0.98	4.04E-03	0.0147
DYNC1LI1	0.98	4.82E-03	0.0165
ENST000003031271	0.98	2.18E-03	0.0100
RADX	0.98	4.60E-03	0.0160

TMEM165	0.98	7.52E-03	0.0221
MT-ND4L	0.98	3.98E-03	0.0145
BRD8	0.98	3.59E-03	0.0136
GPATCH11	0.98	3.52E-03	0.0134
YWHAE	0.98	5.00E-03	0.0168
EZR	0.98	1.06E-02	0.0279
ZFYVE21	0.98	9.00E-03	0.0250
ENST000003116351	0.98	1.74E-02	0.0398
MUM1	0.98	6.85E-03	0.0208
ENST000003475190	0.98	2.22E-02	0.0473
NDUFA9	0.98	2.65E-03	0.0113
REXO4	0.97	4.22E-03	0.0152
EIF4G3	0.97	6.28E-03	0.0195
TRIM16	0.97	4.57E-03	0.0159
ARHGAP31	0.97	7.50E-03	0.0221
MDH1	0.97	3.41E-03	0.0131
ATP6V1E1	0.97	4.68E-03	0.0162
TMEM216	0.97	8.65E-03	0.0243
RPUSD3	0.97	7.61E-03	0.0223
GNB1	0.97	1.61E-03	0.0084
ATF1	0.97	5.13E-03	0.0171
KCTD20	0.97	2.14E-03	0.0099
FBRS	0.97	1.03E-02	0.0274
THRAP3	0.97	2.59E-03	0.0112
PQBP1	0.97	9.88E-03	0.0265
ACSF2	0.97	1.53E-02	0.0362
SLC39A6	0.97	8.20E-03	0.0234
TIMM9	0.97	1.39E-02	0.0339
YIPF1	0.97	4.48E-03	0.0157
NCBP2	0.97	5.35E-03	0.0176
EEF2	0.97	1.10E-02	0.0287
AASDHPPT	0.97	5.72E-03	0.0184
ENST000003148302	0.97	5.64E-03	0.0183
LIMK2	0.97	3.87E-03	0.0143
LAMTOR5	0.97	3.99E-03	0.0145
FAM50A	0.97	9.66E-03	0.0262
NUP54	0.97	3.61E-03	0.0137
ENST000003312421	0.97	4.79E-03	0.0164
PELP1	0.97	1.68E-02	0.0387
PTER	0.96	2.40E-03	0.0106
EFHD2	0.96	7.26E-03	0.0216
THAP5	0.96	3.42E-03	0.0132
PTPMT1	0.96	5.53E-03	0.0180
ELOF1	0.96	7.38E-03	0.0218
TMEM263	0.96	2.38E-03	0.0106
CERS2	0.96	5.25E-03	0.0174
MAF	0.96	2.07E-02	0.0449

MSTO1	0.96	8.37E-03	0.0237
ZNF524	0.96	1.63E-02	0.0379
LARS	0.96	2.15E-03	0.0099
STX6	0.96	5.27E-03	0.0174
PEX2	0.96	2.22E-03	0.0101
YKT6	0.96	4.52E-03	0.0158
BRCC3	0.96	5.66E-03	0.0183
SPINT2	0.96	1.11E-02	0.0289
ZNF436	0.96	6.59E-03	0.0202
NR2C2AP	0.96	1.22E-02	0.0308
RGS10	0.96	2.79E-03	0.0116
ENST000003318250	0.96	1.49E-03	0.0081
BAP1	0.96	2.79E-03	0.0116
NXT2	0.96	1.36E-02	0.0332
ENST000002900370	0.96	2.37E-02	0.0496
COPS4	0.96	6.03E-03	0.0191
B3GAT3	0.96	8.05E-03	0.0231
PSMB4	0.96	5.62E-03	0.0182
ZNF696	0.96	1.14E-02	0.0293
MTAP	0.96	6.38E-03	0.0198
ENST000002618661	0.96	2.28E-03	0.0103
STRBP	0.96	5.37E-03	0.0177
SOCS7	0.96	7.15E-03	0.0213
ENST000002829900	0.96	8.62E-03	0.0242
EXOC5	0.96	4.00E-03	0.0146
CCDC61	0.96	1.79E-02	0.0405
PAAF1	0.96	4.08E-03	0.0148
NBN	0.96	4.29E-03	0.0153
CD2BP2	0.96	1.02E-02	0.0271
PPP2R3B	0.96	8.46E-03	0.0239
BCOR	0.96	6.26E-03	0.0195
ENST000002726383	0.96	1.71E-03	0.0087
CAB39L	0.96	5.58E-03	0.0181
ADAR	0.95	5.88E-03	0.0188
PIAS4	0.95	7.59E-03	0.0223
LY6E	0.95	1.38E-02	0.0337
DCAKD	0.95	1.50E-02	0.0356
NDUFA12	0.95	3.77E-03	0.0140
TMEM135	0.95	1.71E-02	0.0393
GTF2H1	0.95	6.94E-03	0.0209
BCL10	0.95	4.67E-03	0.0161
ZFAS1	0.95	3.52E-03	0.0134
AATF	0.95	6.36E-03	0.0197
WDR92	0.95	9.12E-03	0.0252
GOLGA2	0.95	3.74E-03	0.0140
DNAJC14	0.95	1.09E-02	0.0284
GNA12	0.95	9.26E-03	0.0254

BRAT1	0.95	6.09E-03	0.0192
NOA1	0.95	6.32E-03	0.0196
MRPL27	0.95	5.26E-03	0.0174
NCK1-DT	0.95	1.73E-02	0.0395
MAP3K11	0.95	2.26E-02	0.0478
OAT	0.95	5.47E-03	0.0179
PSMB10	0.95	1.27E-02	0.0318
TMEM64	0.95	7.81E-03	0.0227
IL10RB	0.94	7.76E-03	0.0226
MIS12	0.94	2.76E-03	0.0116
HMGB1	0.94	1.76E-02	0.0400
DHX34	0.94	6.69E-03	0.0204
FIG4	0.94	3.25E-03	0.0128
DHX16	0.94	4.71E-03	0.0162
ARL8B	0.94	2.88E-03	0.0118
FUNDC1	0.94	6.33E-03	0.0196
MCCC1	0.94	6.22E-03	0.0194
MOCS3	0.94	2.08E-02	0.0452
RRP36	0.94	1.83E-02	0.0413
TSPAN17	0.94	6.12E-03	0.0192
SNAPC3	0.94	4.02E-03	0.0146
CAB39	0.94	4.33E-03	0.0154
SLC39A9	0.94	2.95E-03	0.0120
MPND	0.94	2.20E-02	0.0470
EIF2S3	0.94	2.70E-03	0.0114
MED1	0.94	3.14E-03	0.0125
CPT2	0.94	4.90E-03	0.0166
BPNT1	0.94	1.09E-02	0.0285
CXorf40A	0.94	8.52E-03	0.0240
MTPN	0.94	3.81E-03	0.0141
SNU13	0.94	4.46E-03	0.0156
SRP19	0.94	4.29E-03	0.0153
ENST000002196891	0.93	7.76E-03	0.0226
PLEKHJ1	0.93	5.33E-03	0.0176
MRPL4	0.93	2.15E-02	0.0463
ATP5F1D	0.93	1.77E-02	0.0402
POMT2	0.93	9.96E-03	0.0267
GFM1	0.93	1.47E-02	0.0353
CD79B	0.93	8.62E-03	0.0242
MT-CO3	0.93	7.12E-03	0.0213
DNPEP	0.93	7.43E-03	0.0219
RSPH3	0.93	1.65E-02	0.0383
CENPBD1	0.93	2.05E-02	0.0447
ADK	0.93	4.94E-03	0.0167
SNX12	0.93	7.86E-03	0.0228
ENST000003093401	0.93	1.06E-02	0.0279
Z95115.1	0.93	1.33E-02	0.0329

GRHPR	0.93	1.29E-02	0.0321
LYRM1	0.93	4.66E-03	0.0161
FBXO22	0.93	2.52E-03	0.0110
ZNF749	0.93	8.09E-03	0.0232
NAT1	0.93	7.57E-03	0.0222
INTS11	0.93	3.06E-03	0.0123
CACNB1	0.93	2.34E-02	0.0491
ACP1	0.93	3.17E-03	0.0126
SAMM50	0.93	8.91E-03	0.0248
RILPL2	0.93	1.30E-02	0.0323
ENST000002636720	0.93	1.33E-02	0.0327
PIM2	0.92	1.34E-02	0.0330
HSBP1	0.92	4.46E-03	0.0156
ENST000003057470	0.92	2.25E-02	0.0477
B4GALT3	0.92	5.30E-03	0.0175
UBL5	0.92	4.07E-03	0.0148
RXYLT1	0.92	1.49E-02	0.0356
ZNF391	0.92	1.89E-02	0.0422
POT1	0.92	3.61E-03	0.0137
TMEM39A	0.92	3.01E-03	0.0122
SNORA65	0.92	2.12E-02	0.0457
LBR	0.92	4.07E-03	0.0147
GNE	0.92	3.05E-03	0.0123
PPIB	0.92	6.39E-03	0.0198
ARF3	0.92	6.82E-03	0.0207
LYRM4	0.92	5.20E-03	0.0173
SRP14	0.92	8.74E-03	0.0245
KCTD12	0.92	1.90E-02	0.0423
MT-ATP6	0.92	5.08E-03	0.0170
ALG5	0.92	5.26E-03	0.0174
ENST000002300852	0.92	1.34E-02	0.0330
COPA	0.92	3.30E-03	0.0129
AMMECR1L	0.92	1.31E-02	0.0324
COPZ1	0.91	4.76E-03	0.0163
NDUFB9	0.91	3.82E-03	0.0141
FXR1	0.91	2.17E-03	0.0100
KIF5C	0.91	3.70E-03	0.0139
FKBP8	0.91	6.60E-03	0.0202
HIBADH	0.91	4.74E-03	0.0163
YRDC	0.91	1.11E-02	0.0289
ARFGAP3	0.91	3.83E-03	0.0142
AGK	0.91	5.79E-03	0.0186
EIF2B2	0.91	1.41E-02	0.0341
NSUN4	0.91	6.26E-03	0.0195
NF2	0.91	6.25E-03	0.0195
ANKRD9	0.91	1.49E-02	0.0355
TAF13	0.91	8.21E-03	0.0234

SEM1	0.91	3.90E-03	0.0143
PTPN1	0.91	1.15E-02	0.0296
NPR2	0.91	1.07E-02	0.0281
ENST000002064510	0.91	2.64E-03	0.0113
ATP2A2	0.91	5.69E-03	0.0184
CAPZA2	0.91	6.27E-03	0.0195
EYA3	0.91	4.73E-03	0.0163
INTS9	0.91	4.90E-03	0.0166
RPL8	0.91	1.46E-02	0.0351
PHTF1	0.91	8.79E-03	0.0245
DHX37	0.91	7.16E-03	0.0214
DVL2	0.91	1.12E-02	0.0290
NUPL2	0.91	7.52E-03	0.0221
ECPAS	0.91	2.32E-03	0.0104
CHCHD6	0.91	1.08E-02	0.0283
COLGALT1	0.90	9.26E-03	0.0254
CAMKK2	0.90	5.86E-03	0.0187
ENST000003006510	0.90	3.18E-03	0.0126
PPP1R15A	0.90	5.36E-03	0.0176
GGA1	0.90	7.70E-03	0.0225
MNAT1	0.90	9.19E-03	0.0253
UQCR11	0.90	5.38E-03	0.0177
LIAS	0.90	8.79E-03	0.0245
TRAPPC5	0.90	1.46E-02	0.0350
POU2F2	0.90	2.22E-02	0.0473
EMC8	0.90	7.72E-03	0.0225
ATPAF1	0.90	9.46E-03	0.0258
C8orf82	0.90	1.67E-02	0.0387
PRKAR1A	0.90	3.83E-03	0.0142
ENST000003233450	0.90	6.22E-03	0.0194
TAF4B	0.90	6.40E-03	0.0198
MAZ	0.90	7.17E-03	0.0214
GCDH	0.90	2.14E-02	0.0460
SLC2A13	0.90	1.18E-02	0.0301
DNAJC2	0.90	1.85E-02	0.0416
PAFAH1B2	0.90	3.42E-03	0.0132
TRAPPC3	0.90	6.17E-03	0.0194
DNAJA2	0.90	1.49E-02	0.0355
RBL1	0.90	2.57E-03	0.0111
YTHDF2	0.90	2.49E-03	0.0109
NMRAL1	0.90	1.20E-02	0.0304
MPHOSPH10	0.90	9.25E-03	0.0254
ENST000003205850	0.89	1.27E-02	0.0318
AC124944.3	0.89	1.49E-02	0.0355
ZNF175	0.89	9.04E-03	0.0250
CHML	0.89	1.28E-02	0.0319
NUP50	0.89	3.78E-03	0.0141

ANXA7	0.89	1.01E-02	0.0270
RNF14	0.89	5.28E-03	0.0174
MYL12B	0.89	6.59E-03	0.0202
NSL1	0.89	6.29E-03	0.0196
FAM220A	0.89	1.12E-02	0.0290
RHEB	0.89	1.08E-02	0.0282
ZNF85	0.89	1.03E-02	0.0273
MAPRE1	0.89	5.18E-03	0.0173
MAPK3	0.89	8.95E-03	0.0249
RINT1	0.89	5.51E-03	0.0180
BCL2L13	0.89	3.03E-03	0.0122
C8orf59	0.89	4.06E-03	0.0147
DDX31	0.89	6.44E-03	0.0199
PMS1	0.89	1.33E-02	0.0327
ENST000002640801	0.89	1.12E-02	0.0291
ENST000002222542	0.89	1.45E-02	0.0349
RFXANK	0.89	7.88E-03	0.0228
MAP2K2	0.89	5.41E-03	0.0178
TMEM68	0.89	1.22E-02	0.0309
HADHA	0.89	3.87E-03	0.0143
DDX39B	0.89	3.43E-03	0.0132
LARP7	0.89	3.88E-03	0.0143
ORC5	0.89	1.12E-02	0.0290
UTP3	0.89	4.92E-03	0.0167
FCF1	0.89	3.70E-03	0.0139
ST7	0.88	1.91E-02	0.0425
UBA5	0.88	3.02E-03	0.0122
RPA1	0.88	7.12E-03	0.0213
TMEM267	0.88	4.16E-03	0.0150
TSG101	0.88	3.76E-03	0.0140
AP4E1	0.88	8.98E-03	0.0249
USP22	0.88	4.96E-03	0.0167
NDUFV1	0.88	3.84E-03	0.0142
MRPS22	0.88	4.35E-03	0.0154
ENST000002490710	0.88	5.28E-03	0.0174
HNRNPR	0.88	4.94E-03	0.0167
CPSF1	0.88	1.60E-02	0.0374
CARHSP1	0.88	5.91E-03	0.0188
WDR61	0.88	1.39E-02	0.0339
ALPK1	0.88	2.23E-02	0.0475
GJC1	0.88	1.35E-02	0.0331
PUDP	0.88	2.05E-02	0.0446
SENP1	0.88	4.60E-03	0.0160
ZNF664	0.88	9.06E-03	0.0251
SMAD2	0.88	3.45E-03	0.0132
ENST000002659860	0.88	2.28E-02	0.0482
MT-CO2	0.88	4.17E-03	0.0150

ZC3H10	0.88	1.74E-02	0.0397
ELOA	0.88	6.80E-03	0.0206
UBE2E1	0.88	4.81E-03	0.0164
WNK3	0.88	2.16E-02	0.0464
PPP1R3B	0.87	6.60E-03	0.0202
ZCCHC17	0.87	7.90E-03	0.0229
MT-ATP8	0.87	1.31E-02	0.0324
ENST000002458382	0.87	7.29E-03	0.0216
RTRAF	0.87	5.71E-03	0.0184
NDUFB4	0.87	3.24E-03	0.0127
CAPNS1	0.87	2.25E-02	0.0478
AP3M1	0.87	8.31E-03	0.0236
RBM26-AS1	0.87	1.03E-02	0.0272
ERLIN2	0.87	4.69E-03	0.0162
GPKOW	0.87	6.70E-03	0.0205
FPGT	0.87	9.25E-03	0.0254
SLC30A9	0.87	9.81E-03	0.0264
HNRNPM	0.87	1.24E-02	0.0312
NUS1	0.87	3.82E-03	0.0142
PBDC1	0.87	8.03E-03	0.0231
VAMP8	0.87	2.01E-02	0.0441
CAAP1	0.87	1.16E-02	0.0297
PDE6D	0.87	9.50E-03	0.0259
ZNF714	0.87	1.44E-02	0.0347
CTSH	0.87	9.94E-03	0.0266
TMA7	0.87	1.50E-02	0.0357
VPS37A	0.87	4.07E-03	0.0148
C1orf159	0.87	2.03E-02	0.0444
MPV17	0.87	5.85E-03	0.0187
BNIP3L	0.87	2.97E-03	0.0121
SLC25A20	0.87	1.44E-02	0.0347
UBE2V1	0.87	3.60E-03	0.0136
SPG21	0.87	7.79E-03	0.0227
ZNF280C	0.87	1.23E-02	0.0311
SMARCA4	0.86	4.38E-03	0.0155
CREM	0.86	1.89E-02	0.0422
EMG1	0.86	9.29E-03	0.0255
ENST000003219193	0.86	1.43E-02	0.0345
NAA25	0.86	3.77E-03	0.0140
PYCR2	0.86	1.17E-02	0.0300
ENST000002650692	0.86	3.60E-03	0.0136
EIF3J	0.86	1.62E-02	0.0377
MSANTD4	0.86	5.31E-03	0.0175
KAT5	0.86	5.99E-03	0.0190
CUL1	0.86	5.02E-03	0.0169
ATG3	0.86	5.94E-03	0.0189
ALG10	0.86	6.03E-03	0.0191

ATXN2L	0.86	1.95E-02	0.0431
OPA1	0.86	2.75E-03	0.0116
PARN	0.86	4.88E-03	0.0166
ZNF543	0.86	7.91E-03	0.0229
WDR1	0.86	5.25E-03	0.0174
RUNX3	0.86	2.11E-02	0.0456
WDFY1	0.86	2.99E-03	0.0121
LRRC45	0.86	2.39E-02	0.0499
AARSD1	0.86	8.78E-03	0.0245
AKT1S1	0.85	1.78E-02	0.0404
ABCF2	0.85	5.35E-03	0.0176
RPS6KA1	0.85	5.09E-03	0.0170
WDR90	0.85	1.32E-02	0.0326
MT-CO1	0.85	1.62E-03	0.0085
DUSP12	0.85	1.37E-02	0.0336
SRBD1	0.85	3.91E-03	0.0144
NQO2	0.85	7.02E-03	0.0211
CYREN	0.85	1.40E-02	0.0341
RBM17	0.85	1.06E-02	0.0279
ENST000003044141	0.85	5.91E-03	0.0188
COX15	0.85	7.47E-03	0.0220
MUT	0.85	1.31E-02	0.0324
UQCC3	0.85	1.94E-02	0.0429
ENST00000536999	0.85	2.27E-02	0.0480
LARS2	0.85	1.52E-02	0.0361
C19orf47	0.85	2.17E-02	0.0464
ENST000003031420	0.85	1.59E-02	0.0372
UBE2N	0.85	1.25E-02	0.0314
ATAD3B	0.85	1.48E-02	0.0355
PDCD6	0.85	7.53E-03	0.0222
ATP5MG	0.84	1.55E-02	0.0366
KLHDC3	0.84	1.27E-02	0.0318
SIRT3	0.84	9.84E-03	0.0265
DNAJA4	0.84	6.07E-03	0.0192
TRIM26	0.84	9.71E-03	0.0262
DHRS4-AS1	0.84	1.48E-02	0.0353
LIN52	0.84	1.58E-02	0.0371
CWC27	0.84	4.31E-03	0.0153
CLPTM1L	0.84	1.70E-02	0.0391
BTBD19	0.84	1.62E-02	0.0377
METTL2B	0.84	1.09E-02	0.0285
ASAH2	0.84	1.72E-02	0.0394
CCNC	0.84	4.67E-03	0.0161
ENST000002369800	0.84	5.12E-03	0.0171
ZKSCAN5	0.84	6.10E-03	0.0192
HMGN1	0.84	6.79E-03	0.0206
STX12	0.84	4.94E-03	0.0167

AC020915.5	0.84	1.76E-02	0.0401
IFT74	0.84	2.11E-02	0.0455
TMBIM6	0.84	2.65E-03	0.0113
ALKBH8	0.84	1.41E-02	0.0343
AGPS	0.84	5.86E-03	0.0187
MED28	0.84	1.45E-02	0.0349
RPS6KB2	0.84	8.16E-03	0.0234
PSD4	0.84	1.14E-02	0.0295
ELL	0.84	1.28E-02	0.0319
ENST000002341150	0.84	6.51E-03	0.0200
AC024940.5	0.84	2.03E-02	0.0444
SBF2-AS1	0.84	6.32E-03	0.0196
PRR3	0.84	7.90E-03	0.0229
C1GALT1C1	0.84	1.60E-02	0.0374
ENST000002939810	0.84	2.35E-02	0.0492
TP53	0.83	1.82E-02	0.0411
ENST000002864481	0.83	4.76E-03	0.0163
PAM	0.83	6.22E-03	0.0194
PHAX	0.83	8.92E-03	0.0248
AC006504.5	0.83	1.62E-02	0.0377
HNRNPUL2	0.83	8.02E-03	0.0231
EMC4	0.83	7.73E-03	0.0225
RPL7A	0.83	7.13E-03	0.0213
ENST000002950872	0.83	1.01E-02	0.0270
EIF4ENIF1	0.83	1.25E-02	0.0314
EIF3M	0.83	4.13E-03	0.0149
ANKRD40	0.83	8.76E-03	0.0245
NDE1	0.83	4.93E-03	0.0167
PEBP1	0.83	1.14E-02	0.0293
EHBP1L1	0.83	5.21E-03	0.0173
MKKS	0.83	1.11E-02	0.0289
PATL1	0.83	1.40E-02	0.0339
TFG	0.83	8.11E-03	0.0233
ATN1	0.83	2.39E-02	0.0499
ENST000003339900	0.83	1.23E-02	0.0311
TAF1B	0.83	4.88E-03	0.0166
AP5B1	0.83	2.16E-02	0.0464
PIN4	0.83	1.24E-02	0.0311
MMAA	0.83	5.94E-03	0.0189
ENST000002771650	0.83	5.31E-03	0.0175
ENST000003409130	0.83	1.03E-02	0.0274
RACK1	0.83	2.56E-03	0.0111
RCL1	0.83	1.30E-02	0.0323
HDAC11	0.82	2.26E-02	0.0478
TRNAU1AP	0.82	8.49E-03	0.0240
ZFYVE19	0.82	9.01E-03	0.0250
GIT1	0.82	1.85E-02	0.0416

ATG4B	0.82	6.08E-03	0.0192
STK38L	0.82	1.90E-02	0.0423
GUK1	0.82	8.11E-03	0.0233
RIOX2	0.82	8.55E-03	0.0241
C1orf122	0.82	2.02E-02	0.0443
TBK1	0.82	1.51E-02	0.0358
CNOT1	0.82	3.31E-03	0.0129
ABCG1	0.82	8.15E-03	0.0233
HNRNPA1	0.82	7.71E-03	0.0225
SLC46A1	0.82	1.93E-02	0.0428
AARS2	0.82	6.77E-03	0.0206
CCZ1	0.82	3.76E-03	0.0140
FPGS	0.82	8.53E-03	0.0241
DNAJC11	0.82	7.65E-03	0.0224
SNAP47	0.82	8.75E-03	0.0245
NCLN	0.81	1.83E-02	0.0412
SH3BP5L	0.81	2.14E-02	0.0460
ZNF106	0.81	3.25E-03	0.0128
ENST000003129161	0.81	6.13E-03	0.0193
GATB	0.81	1.25E-02	0.0314
EIF2A	0.81	4.28E-03	0.0153
UPF1	0.81	5.18E-03	0.0173
IDI1	0.81	1.63E-02	0.0378
QRSL1	0.81	1.24E-02	0.0311
RABGEF1	0.81	6.52E-03	0.0201
DTD2	0.81	1.52E-02	0.0360
TPRA1	0.81	1.59E-02	0.0373
G3BP1	0.81	1.82E-02	0.0410
ARL6IP6	0.81	1.35E-02	0.0331
PABPN1	0.81	1.99E-02	0.0438
HEXIM1	0.81	1.66E-02	0.0384
ARSB	0.81	9.10E-03	0.0252
ATL2	0.81	7.57E-03	0.0222
NELFCD	0.81	1.39E-02	0.0338
DBR1	0.81	7.33E-03	0.0217
SASH3	0.81	6.64E-03	0.0203
PHRF1	0.81	6.48E-03	0.0200
ZNF384	0.81	1.68E-02	0.0387
DYNC1I2	0.81	6.48E-03	0.0200
SLC30A6	0.81	6.10E-03	0.0192
DNAJC17	0.81	1.86E-02	0.0418
PDIA3	0.81	3.97E-03	0.0145
DGUOK	0.81	2.12E-02	0.0457
SMARCAD1	0.80	7.75E-03	0.0226
ZNF646	0.80	1.34E-02	0.0330
ENST000002635590	0.80	8.01E-03	0.0231
SH3BGRL3	0.80	2.06E-02	0.0449

PRPF40A	0.80	6.74E-03	0.0205
PANK4	0.80	8.31E-03	0.0236
CHRAC1	0.80	1.07E-02	0.0281
ZCCHC9	0.80	1.21E-02	0.0307
RNF34	0.80	4.39E-03	0.0155
SNHG26	0.80	1.38E-02	0.0336
TRIM38	0.80	9.22E-03	0.0254
MIPEP	0.80	1.23E-02	0.0310
DYNLRB1	0.80	1.33E-02	0.0327
CDC26	0.80	7.58E-03	0.0222
ATP5PD	0.80	5.25E-03	0.0174
STRAP	0.80	1.31E-02	0.0324
COPS6	0.80	1.35E-02	0.0332
TNFRSF1A	0.80	2.22E-02	0.0473
ENST000002375300	0.80	5.59E-03	0.0181
ZCCHC10	0.80	7.81E-03	0.0227
DDX10	0.80	1.23E-02	0.0310
GNPDA2	0.80	1.55E-02	0.0366
MAN1A1	0.80	2.28E-02	0.0482
TRIM5	0.80	2.13E-02	0.0460
QARS	0.79	6.50E-03	0.0200
IFT52	0.79	1.27E-02	0.0317
XPO5	0.79	1.47E-02	0.0352
DNAJB11	0.79	8.51E-03	0.0240
ENST000003062701	0.79	9.96E-03	0.0267
KCMF1	0.79	7.31E-03	0.0217
CDKN2D	0.79	1.94E-02	0.0429
MMS19	0.79	1.58E-02	0.0370
ENST000002902460	0.79	6.88E-03	0.0208
AMPD2	0.79	1.15E-02	0.0295
HIRA	0.79	6.31E-03	0.0196
CYB5D2	0.79	1.38E-02	0.0337
STK16	0.79	7.18E-03	0.0214
MRPL9	0.79	5.56E-03	0.0181
CNPY2	0.79	1.75E-02	0.0399
METTL15	0.79	1.27E-02	0.0318
PDCL	0.79	7.23E-03	0.0215
AIP	0.79	9.15E-03	0.0253
CMTR2	0.79	7.07E-03	0.0212
ARL6IP4	0.79	1.62E-02	0.0378
UBL7-AS1	0.79	2.26E-02	0.0479
BTF3L4	0.79	1.74E-02	0.0398
ZNF692	0.79	9.90E-03	0.0266
CNOT11	0.79	6.07E-03	0.0192
ENST000003229400	0.79	1.38E-02	0.0336
OSBP	0.79	6.26E-03	0.0195
TGS1	0.79	6.84E-03	0.0207

IQCE	0.79	1.63E-02	0.0379
GM2A	0.79	8.60E-03	0.0242
KDM1A	0.79	5.39E-03	0.0177
NPTN	0.79	8.60E-03	0.0242
MCCC2	0.79	7.03E-03	0.0211
PMS2P1	0.79	1.90E-02	0.0423
CCDC171	0.79	8.90E-03	0.0248
NFU1	0.79	9.02E-03	0.0250
RNF40	0.79	5.75E-03	0.0185
CHMP1A	0.78	1.59E-02	0.0373
TMEM9	0.78	1.31E-02	0.0324
DNAJC1	0.78	1.07E-02	0.0280
RNPS1	0.78	5.37E-03	0.0176
RAB1A	0.78	5.25E-03	0.0174
PAIP1	0.78	8.22E-03	0.0234
JAK2	0.78	1.50E-02	0.0357
MED11	0.78	2.35E-02	0.0493
TDP2	0.78	5.83E-03	0.0187
RNF213	0.78	1.91E-02	0.0425
RAB5A	0.78	1.18E-02	0.0302
GRB2	0.78	4.78E-03	0.0164
PABPC1L	0.78	1.77E-02	0.0402
KRT10	0.78	1.52E-02	0.0360
VAT1	0.78	2.19E-02	0.0468
FAM184A	0.78	1.34E-02	0.0330
MITD1	0.78	6.00E-03	0.0190
ZNF121	0.78	9.12E-03	0.0252
EWSR1	0.78	5.72E-03	0.0184
CDC5L	0.78	5.76E-03	0.0185
PITPNM1	0.78	1.21E-02	0.0307
BLOC1S5	0.78	1.01E-02	0.0270
TCERG1	0.78	7.84E-03	0.0228
PISD	0.78	1.37E-02	0.0335
MTREX	0.77	7.39E-03	0.0218
ADIPOR2	0.77	2.10E-02	0.0455
CDK16	0.77	1.66E-02	0.0385
RPL6P27	0.77	2.27E-02	0.0480
ARL5A	0.77	1.56E-02	0.0368
HACD2	0.77	8.17E-03	0.0234
WDR45B	0.77	6.76E-03	0.0206
TEX261	0.77	1.52E-02	0.0361
BRPF1	0.77	9.59E-03	0.0260
TXNRD2	0.77	1.32E-02	0.0325
ZNF684	0.77	2.22E-02	0.0473
STK24	0.77	5.29E-03	0.0175
SNHG16	0.77	8.63E-03	0.0242
PCNT	0.77	9.57E-03	0.0260

ZMAT5	0.77	1.58E-02	0.0371
TNFAIP8L1	0.77	2.10E-02	0.0455
EXOSC10	0.77	7.63E-03	0.0223
NUP88	0.77	8.87E-03	0.0247
ZNF622	0.77	8.26E-03	0.0235
MAP9	0.77	1.58E-02	0.0371
KIAA2013	0.76	1.05E-02	0.0277
SLC25A40	0.76	7.08E-03	0.0212
DNM1L	0.76	1.08E-02	0.0283
HTATSF1	0.76	1.46E-02	0.0350
RASSF5	0.76	5.11E-03	0.0171
THOC5	0.76	7.72E-03	0.0225
ADAT2	0.76	7.62E-03	0.0223
SRSF9	0.76	8.50E-03	0.0240
PDIK1L	0.76	7.88E-03	0.0228
ENST000002941890	0.76	9.43E-03	0.0257
UQCC1	0.76	9.85E-03	0.0265
ITGAV	0.76	1.19E-02	0.0303
SEC24C	0.76	2.15E-02	0.0462
AFDN	0.76	9.73E-03	0.0263
SMARCAL1	0.76	1.06E-02	0.0278
KRTCAP2	0.76	1.09E-02	0.0285
ENST000003552380	0.76	6.18E-03	0.0194
GTF2H5	0.76	1.67E-02	0.0386
SRGAP2	0.76	1.62E-02	0.0377
RAP1A	0.76	5.75E-03	0.0185
TRIM59	0.75	9.85E-03	0.0265
TAF2	0.75	5.93E-03	0.0189
ARMCX6	0.75	9.21E-03	0.0254
CUL4A	0.75	7.85E-03	0.0228
MUTYH	0.75	1.90E-02	0.0423
ANXA4	0.75	1.04E-02	0.0275
DNAJC21	0.75	1.40E-02	0.0340
ENST000002750361	0.75	1.94E-02	0.0429
CNOT10	0.75	9.70E-03	0.0262
RALBP1	0.75	8.09E-03	0.0232
LINC00963	0.75	1.78E-02	0.0405
GTF3A	0.75	1.99E-02	0.0437
ZFP91-CNTF	0.75	2.00E-02	0.0438
CERS4	0.75	1.93E-02	0.0428
ARFIP2	0.75	1.02E-02	0.0272
TMEM41B	0.75	1.54E-02	0.0364
RTF2	0.75	8.38E-03	0.0238
DENND3	0.75	2.36E-02	0.0495
TEX10	0.75	2.16E-02	0.0464
UBXN8	0.75	2.36E-02	0.0495
RPL35	0.75	5.84E-03	0.0187

SLC25A3	0.75	1.01E-02	0.0269
ANKRD39	0.75	2.34E-02	0.0491
RER1	0.75	1.45E-02	0.0350
GNL1	0.75	1.61E-02	0.0376
NUBP1	0.75	1.60E-02	0.0375
HINT1	0.75	6.43E-03	0.0199
CCND3	0.74	5.55E-03	0.0181
KXD1	0.74	1.06E-02	0.0280
MRPL48	0.74	1.73E-02	0.0395
COMMD1	0.74	1.87E-02	0.0418
PUS10	0.74	1.49E-02	0.0355
ENST000002789030	0.74	2.29E-02	0.0483
MRPS21	0.74	8.23E-03	0.0235
ENST000003258052	0.74	1.11E-02	0.0288
PARP14	0.74	1.40E-02	0.0340
TRIM41	0.74	1.12E-02	0.0291
CDK5RAP2	0.74	5.69E-03	0.0184
RALY	0.74	1.32E-02	0.0325
ASXL1	0.74	7.85E-03	0.0228
METTL9	0.74	7.75E-03	0.0226
APMAP	0.74	8.55E-03	0.0241
PPTC7	0.74	1.87E-02	0.0418
TRMT1L	0.74	9.15E-03	0.0252
MEAF6	0.74	1.44E-02	0.0348
FOXK2	0.74	1.23E-02	0.0311
C6orf203	0.74	1.49E-02	0.0355
SNRNP70	0.74	1.31E-02	0.0324
ERCC8	0.74	1.75E-02	0.0399
RRAGA	0.73	2.13E-02	0.0460
MZT2A	0.73	1.28E-02	0.0319
GUSB	0.73	1.21E-02	0.0306
AGO1	0.73	1.23E-02	0.0310
PSMD6	0.73	1.94E-02	0.0429
NPC2	0.73	1.52E-02	0.0360
ADIPOR1	0.73	1.33E-02	0.0329
RFESD	0.73	1.98E-02	0.0435
ENST000003414841	0.73	1.03E-02	0.0272
TNPO1	0.73	2.14E-02	0.0460
UBE2D2	0.73	8.19E-03	0.0234
EAF1	0.73	1.12E-02	0.0290
CSNK1A1	0.73	7.32E-03	0.0217
MAP4	0.73	1.64E-02	0.0381
NOP9	0.73	1.47E-02	0.0352
SMARCA5	0.73	1.16E-02	0.0298
LONRF1	0.73	1.85E-02	0.0416
ING1	0.73	1.47E-02	0.0352
RAD21	0.73	7.05E-03	0.0212

ZNF286A	0.73	9.74E-03	0.0263
COX7C	0.73	6.34E-03	0.0197
EIF3L	0.73	5.06E-03	0.0170
ENST000002406513	0.73	7.67E-03	0.0224
DHX15	0.72	8.13E-03	0.0233
INTS1	0.72	1.34E-02	0.0330
RNF41	0.72	1.32E-02	0.0326
DAP3	0.72	1.01E-02	0.0270
AP2B1	0.72	5.77E-03	0.0186
NKIRAS1	0.72	1.91E-02	0.0425
SSB	0.72	1.34E-02	0.0330
ETFDH	0.72	1.16E-02	0.0297
BUB3	0.72	6.19E-03	0.0194
FAM114A2	0.72	1.31E-02	0.0324
ATP6V1B2	0.72	8.59E-03	0.0242
HENMT1	0.72	2.16E-02	0.0463
MED27	0.72	1.84E-02	0.0414
PSMC6	0.72	1.27E-02	0.0318
PDCD2	0.72	1.73E-02	0.0396
OSTF1	0.72	8.04E-03	0.0231
RSPRY1	0.72	9.83E-03	0.0265
SPCS2	0.72	8.62E-03	0.0242
TCAIM	0.71	1.93E-02	0.0428
NAA35	0.71	1.00E-02	0.0268
MICB	0.71	1.37E-02	0.0335
FUBP3	0.71	1.73E-02	0.0396
RPL37A	0.71	4.38E-03	0.0155
GID8	0.71	9.29E-03	0.0255
FASTKD2	0.71	1.39E-02	0.0338
STAT6	0.71	1.37E-02	0.0336
ENST000003346900	0.71	1.51E-02	0.0358
PPP2R3C	0.71	8.04E-03	0.0231
PLEKHM2	0.71	1.22E-02	0.0309
SDF2	0.71	1.82E-02	0.0410
PRR5L	0.71	2.37E-02	0.0497
FKBP3	0.71	9.25E-03	0.0254
HIST4H4	0.71	8.05E-03	0.0231
SEH1L	0.71	1.79E-02	0.0405
CAMLG	0.71	1.49E-02	0.0355
RPL4	0.71	4.02E-03	0.0146
RNH1	0.71	1.40E-02	0.0341
PDCD7	0.71	9.44E-03	0.0258
NOP58	0.71	1.31E-02	0.0324
UBE2Q1	0.71	9.98E-03	0.0267
MTFMT	0.71	1.87E-02	0.0418
TPD52	0.71	1.23E-02	0.0310
MRS2	0.71	1.16E-02	0.0298

ENST000002653930	0.70	6.51E-03	0.0200
ADAL	0.70	1.70E-02	0.0390
OTUB1	0.70	1.65E-02	0.0382
TXLNG	0.70	9.93E-03	0.0266
FMNL1	0.70	2.36E-02	0.0494
PCNP	0.70	7.34E-03	0.0217
BFAR	0.70	1.37E-02	0.0336
DVL1	0.70	2.31E-02	0.0486
ZBED1	0.70	1.28E-02	0.0319
RNF6	0.70	1.33E-02	0.0327
ECI2	0.70	1.03E-02	0.0273
SURF6	0.70	9.69E-03	0.0262
ENDOD1	0.70	1.05E-02	0.0276
ZC3H8	0.70	1.53E-02	0.0363
SPTLC2	0.70	9.01E-03	0.0250
PRDM10	0.70	1.92E-02	0.0427
ADH5	0.70	9.27E-03	0.0254
HIST2H2AC	0.70	7.67E-03	0.0224
RTN3	0.70	1.75E-02	0.0399
PPP2R1B	0.70	2.32E-02	0.0488
ENST000002638570	0.70	1.73E-02	0.0395
KATNA1	0.69	1.77E-02	0.0403
RBM4B	0.69	1.90E-02	0.0423
CDC16	0.69	1.36E-02	0.0334
PTCD2	0.69	1.48E-02	0.0354
MDM2	0.69	8.87E-03	0.0247
ENST000003305011	0.69	1.21E-02	0.0307
ACTR2	0.69	1.19E-02	0.0303
BCL7C	0.69	1.93E-02	0.0428
ACTR6	0.69	9.97E-03	0.0267
MUS81	0.69	1.62E-02	0.0378
BRK1	0.69	2.11E-02	0.0455
URGCP	0.69	1.48E-02	0.0354
BMS1P4	0.69	1.99E-02	0.0437
C12orf4	0.69	1.57E-02	0.0369
GMPR2	0.69	1.17E-02	0.0299
CTR9	0.69	1.73E-02	0.0395
CLP1	0.68	1.63E-02	0.0378
ADSL	0.68	1.80E-02	0.0408
DNAJB2	0.68	1.09E-02	0.0285
EXO5	0.68	2.01E-02	0.0441
ENST000003921290	0.68	1.27E-02	0.0317
CNIH4	0.68	1.67E-02	0.0386
RASSF2	0.68	1.65E-02	0.0383
TRMT44	0.68	1.83E-02	0.0412
MRPL54	0.68	1.77E-02	0.0403
GOLGA3	0.68	8.25E-03	0.0235

RPL6	0.68	1.09E-02	0.0285
ECD	0.68	9.73E-03	0.0263
POC1B	0.68	1.35E-02	0.0332
DNAJC19	0.68	1.71E-02	0.0392
RAB35	0.68	2.16E-02	0.0464
GLMN	0.68	1.55E-02	0.0366
ENST000003184101	0.68	2.00E-02	0.0439
UBLCP1	0.68	1.89E-02	0.0421
FAM111A	0.68	1.44E-02	0.0347
UBE2D1	0.68	1.73E-02	0.0395
HMGXB4	0.68	1.30E-02	0.0322
SMU1	0.67	1.80E-02	0.0407
ZFP1	0.67	1.68E-02	0.0387
SAFB	0.67	1.43E-02	0.0345
MED22	0.67	1.65E-02	0.0383
HTT	0.67	1.43E-02	0.0345
PRKRA	0.67	1.07E-02	0.0281
AEN	0.67	1.02E-02	0.0271
DPH5	0.67	1.24E-02	0.0312
MAPKAPK2	0.67	1.14E-02	0.0293
LIN54	0.67	1.14E-02	0.0294
NNT	0.67	8.49E-03	0.0240
HPS5	0.67	1.08E-02	0.0283
MRE11	0.67	1.33E-02	0.0327
SELENOS	0.67	2.30E-02	0.0485
ENSA	0.67	1.21E-02	0.0306
TUBGCP3	0.67	1.35E-02	0.0332
EDC4	0.67	1.66E-02	0.0384
GTPBP8	0.67	1.29E-02	0.0321
SSR1	0.67	9.89E-03	0.0266
C2orf69	0.67	1.70E-02	0.0391
ENST000003317381	0.67	1.26E-02	0.0316
MTDH	0.67	2.03E-02	0.0443
LCMT1	0.67	2.01E-02	0.0441
HNRNPA2B1	0.66	1.38E-02	0.0336
CSNK1E	0.66	1.19E-02	0.0302
WDR55	0.66	1.44E-02	0.0347
HDAC1	0.66	1.84E-02	0.0414
PITHD1	0.66	1.30E-02	0.0322
NHLRC2	0.66	1.32E-02	0.0326
MCU	0.66	1.99E-02	0.0437
HADHB	0.66	1.32E-02	0.0326
UNC50	0.66	1.69E-02	0.0388
DCTN5	0.66	1.44E-02	0.0347
PMM2	0.66	1.14E-02	0.0294
CBWD1	0.66	1.07E-02	0.0281
ENST000003217570	0.66	9.85E-03	0.0265

UCK1	0.66	2.22E-02	0.0473
VAV1	0.66	1.02E-02	0.0272
RABGGTB	0.66	1.23E-02	0.0311
HEATR3	0.66	1.52E-02	0.0361
SDHC	0.66	1.14E-02	0.0295
ENST000002644511	0.66	1.31E-02	0.0325
KPNA6	0.66	1.22E-02	0.0308
NOL6	0.66	1.94E-02	0.0429
TAF8	0.66	1.49E-02	0.0355
SSX2IP	0.65	2.08E-02	0.0452
SRRT	0.65	2.07E-02	0.0450
NGLY1	0.65	1.08E-02	0.0283
VPS16	0.65	1.17E-02	0.0298
DHX38	0.65	1.68E-02	0.0388
KLHDC4	0.65	1.37E-02	0.0335
SEL1L	0.65	1.14E-02	0.0295
RBMXL1	0.65	1.84E-02	0.0415
RPL36A	0.65	6.80E-03	0.0206
TMEM209	0.65	1.47E-02	0.0352
LIG3	0.65	1.63E-02	0.0378
CD81	0.65	1.44E-02	0.0348
TMEM38B	0.65	1.71E-02	0.0392
RIF1	0.65	1.10E-02	0.0287
C2orf74	0.65	1.68E-02	0.0387
NEPRO	0.65	1.63E-02	0.0379
LTN1	0.65	1.01E-02	0.0269
CEP170	0.65	1.00E-02	0.0268
TBC1D9B	0.65	1.50E-02	0.0357
RBM28	0.64	1.22E-02	0.0309
TDRD7	0.64	2.39E-02	0.0499
DIS3	0.64	1.10E-02	0.0287
CWC22	0.64	1.11E-02	0.0289
U2SURP	0.64	8.38E-03	0.0238
ENST000002536921	0.64	1.78E-02	0.0404
UGGT1	0.64	1.07E-02	0.0280
MAN2B1	0.64	1.47E-02	0.0352
ENST000003389700	0.64	1.82E-02	0.0410
OARD1	0.64	1.22E-02	0.0308
NME6	0.64	1.81E-02	0.0409
GRK2	0.64	1.93E-02	0.0429
CNBP	0.64	1.99E-02	0.0437
ENST000003553541	0.64	1.60E-02	0.0375
SUSD3	0.64	1.87E-02	0.0419
KATNBL1	0.64	1.28E-02	0.0319
EHMT2	0.63	1.72E-02	0.0394
HMGXB3	0.63	1.77E-02	0.0401
GRPEL2	0.63	1.58E-02	0.0371

RPS5	0.63	1.57E-02	0.0369
RBBP4	0.63	1.29E-02	0.0321
RN7SL1	0.63	3.74E-03	0.0140
ENST000002569352	0.63	1.13E-02	0.0292
FZR1	0.63	2.33E-02	0.0490
MT-ND5	0.63	1.24E-02	0.0311
ZDHHC5	0.63	1.37E-02	0.0335
ZHX1	0.63	1.53E-02	0.0363
SNRNP48	0.63	1.84E-02	0.0414
SETD5	0.63	2.14E-02	0.0460
TUBGCP2	0.63	2.28E-02	0.0481
TAF9	0.63	2.09E-02	0.0453
TRIP12	0.62	8.24E-03	0.0235
KIF2A	0.62	1.27E-02	0.0318
SQSTM1	0.62	1.28E-02	0.0319
RDH14	0.62	1.76E-02	0.0400
CCDC14	0.62	1.71E-02	0.0393
TAF15	0.62	2.10E-02	0.0454
ZBTB8OS	0.62	2.16E-02	0.0464
POLDIP3	0.62	1.41E-02	0.0343
CEBPZ	0.62	2.31E-02	0.0487
TMSB10	0.62	1.07E-02	0.0281
SLC15A4	0.61	2.32E-02	0.0488
CSK	0.61	2.20E-02	0.0469
TMED3	0.61	1.67E-02	0.0386
UGP2	0.61	2.21E-02	0.0471
SEC11A	0.61	1.99E-02	0.0437
EXOC2	0.61	1.68E-02	0.0388
CFAP97	0.61	2.14E-02	0.0460
DNAJC7	0.61	1.70E-02	0.0392
DCLRE1C	0.61	1.62E-02	0.0377
UBC	0.61	7.39E-03	0.0219
CMTR1	0.61	1.89E-02	0.0422
EIF4B	0.61	1.17E-02	0.0300
GOLGA5	0.61	1.49E-02	0.0355
ELMSAN1	0.60	1.81E-02	0.0410
RBM18	0.60	1.87E-02	0.0418
MAP4K1	0.60	2.30E-02	0.0484
GGPS1	0.60	1.71E-02	0.0392
MGAT1	0.60	1.83E-02	0.0412
PPIP5K1	0.60	2.06E-02	0.0448
NVL	0.60	1.88E-02	0.0420
NSRP1	0.60	1.86E-02	0.0418
ENST000003010153	0.60	2.13E-02	0.0460
PMS2CL	0.60	2.28E-02	0.0482
DDX47	0.60	2.01E-02	0.0441
ZFP91	0.60	1.69E-02	0.0388

PLEKHA8	0.60	2.38E-02	0.0497
MVP	0.60	1.81E-02	0.0409
NBR1	0.60	2.36E-02	0.0495
TACC1	0.60	1.53E-02	0.0362
RPL24	0.59	1.22E-02	0.0308
ENST000002613401	0.59	2.08E-02	0.0452
RAD17	0.59	1.66E-02	0.0383
GPAT4	0.59	2.22E-02	0.0474
SLC25A12	0.58	1.84E-02	0.0414
HERPUD1	0.58	2.01E-02	0.0441
EEF1AKMT2	0.58	2.20E-02	0.0470
PJA1	0.58	2.32E-02	0.0487
ZFAND1	0.58	1.55E-02	0.0365
NCBP3	0.58	2.23E-02	0.0475
P2RX7	0.58	2.28E-02	0.0482
ANKRD27	0.58	1.79E-02	0.0406
GLS	0.58	1.16E-02	0.0297
GTF2A1	0.58	1.70E-02	0.0391
ERLEC1	0.58	1.81E-02	0.0408
NCOA4	0.58	1.53E-02	0.0362
DCAF1	0.58	2.07E-02	0.0450
ARIH2	0.58	1.63E-02	0.0379
ZNF544	0.58	2.15E-02	0.0462
UPF3A	0.58	2.00E-02	0.0439
CSNK2A2	0.58	2.17E-02	0.0464
AFTPH	0.57	2.08E-02	0.0452
LINC01215	0.57	2.00E-02	0.0439
ZNF197	0.57	2.13E-02	0.0460
RPL7	0.57	1.53E-02	0.0361
TRPM7	0.56	1.57E-02	0.0368
ELOC	0.56	2.30E-02	0.0486
PANK2	0.56	2.23E-02	0.0474
ENST000003035382	0.56	1.66E-02	0.0383
IGF2R	0.55	1.89E-02	0.0422
SPAG9	0.55	1.75E-02	0.0400
PTDSS1	0.55	1.86E-02	0.0417
IL27RA	0.55	2.30E-02	0.0485
AKAP11	0.55	1.89E-02	0.0421
ENST000002992590	0.55	2.25E-02	0.0477
EEF1B2	0.54	2.03E-02	0.0444
SPOP	0.54	2.39E-02	0.0499
SLC30A7	0.54	2.20E-02	0.0470
UBR5	0.54	1.92E-02	0.0427
DCP2	0.54	2.10E-02	0.0455
CLK2	0.54	2.35E-02	0.0493
XRN2	0.53	2.21E-02	0.0471
YME1L1	0.52	2.06E-02	0.0448

RPL10A	0.52	1.97E-02	0.0434
EEF1G	0.50	2.13E-02	0.0459
CNOT2	-0.51	2.38E-02	0.0497
LONP2	-0.52	2.04E-02	0.0445
PHIP	-0.53	1.97E-02	0.0435
ZBTB44	-0.53	2.35E-02	0.0493
WDR33	-0.54	2.02E-02	0.0442
SLMAP	-0.55	2.25E-02	0.0477
ENST000002856790	-0.55	1.88E-02	0.0420
ANKRD36C	-0.56	1.56E-02	0.0366
ATXN3	-0.56	2.18E-02	0.0467
RALGAPB	-0.56	1.98E-02	0.0435
ITSN2	-0.56	1.66E-02	0.0383
SCAF11	-0.56	1.94E-02	0.0429
MPHOSPH8	-0.56	1.96E-02	0.0432
MLLT10	-0.56	2.36E-02	0.0494
GLOD4	-0.57	2.35E-02	0.0493
ZNF644	-0.57	1.87E-02	0.0419
PIKFYVE	-0.57	1.83E-02	0.0412
KDM3A	-0.57	2.14E-02	0.0460
PDS5A	-0.57	2.32E-02	0.0488
ZNF791	-0.58	1.91E-02	0.0426
OGA	-0.58	2.10E-02	0.0455
PCGF5	-0.58	1.81E-02	0.0409
MAP3K2	-0.58	1.35E-02	0.0332
ZNF26	-0.58	2.19E-02	0.0468
PIGN	-0.58	1.47E-02	0.0353
DEGS1	-0.58	1.92E-02	0.0426
FOXO1	-0.58	1.45E-02	0.0348
RB1CC1	-0.59	1.55E-02	0.0366
CASP4	-0.59	1.68E-02	0.0387
COQ10B	-0.59	2.04E-02	0.0445
ZNF136	-0.59	1.86E-02	0.0418
CHKB	-0.59	2.08E-02	0.0451
FAM78A	-0.59	2.15E-02	0.0462
ENST000003343440	-0.60	2.18E-02	0.0467
LNPEP	-0.60	1.60E-02	0.0374
NUDT3	-0.60	1.79E-02	0.0405
KLHL5	-0.60	1.95E-02	0.0432
MED21	-0.60	2.14E-02	0.0460
PTPRJ	-0.60	1.34E-02	0.0330
COMMD6	-0.61	2.39E-02	0.0499
ARL6IP5	-0.61	1.55E-02	0.0366
PAPOLA	-0.61	2.05E-02	0.0446
FAM133B	-0.61	2.06E-02	0.0449
SP4	-0.61	1.68E-02	0.0388
CRACR2A	-0.61	1.99E-02	0.0437

MIA3	-0.61	1.49E-02	0.0356
LEMD3	-0.61	1.86E-02	0.0416
SFSWAP	-0.61	1.72E-02	0.0394
PHF3	-0.62	1.01E-02	0.0269
TFDP2	-0.62	1.60E-02	0.0375
SREK1	-0.62	1.72E-02	0.0395
RETREG3	-0.62	1.65E-02	0.0382
MPPE1	-0.62	1.87E-02	0.0418
TRAF5	-0.62	1.64E-02	0.0381
DIPK1A	-0.62	1.86E-02	0.0418
SOD2	-0.62	1.72E-02	0.0394
C12orf45	-0.62	1.49E-02	0.0356
ZNF431	-0.62	2.31E-02	0.0487
NUCB2	-0.63	2.06E-02	0.0449
TRA2B	-0.63	1.85E-02	0.0415
CCNDBP1	-0.63	1.15E-02	0.0296
C2CD3	-0.63	2.10E-02	0.0454
ZFAND5	-0.63	1.46E-02	0.0351
RBM5	-0.63	1.12E-02	0.0290
ENST000002952380	-0.63	2.38E-02	0.0497
POLR3E	-0.63	2.16E-02	0.0464
ZBED5	-0.63	1.47E-02	0.0353
PSME4	-0.63	1.84E-02	0.0413
DDB1	-0.63	2.28E-02	0.0482
DCAF16	-0.63	2.16E-02	0.0464
LRCH1	-0.63	2.37E-02	0.0495
HCG18	-0.63	1.17E-02	0.0298
TMEM123	-0.64	2.33E-02	0.0490
EXOC8	-0.64	1.38E-02	0.0336
TRAPPC2	-0.64	1.95E-02	0.0431
FAM98B	-0.64	1.94E-02	0.0429
CDR2	-0.64	1.10E-02	0.0286
NBPF8	-0.64	1.38E-02	0.0336
NBPF10	-0.64	1.61E-02	0.0376
CHD9	-0.64	1.13E-02	0.0291
NUTM2A-AS1	-0.64	1.99E-02	0.0437
COG1	-0.65	1.97E-02	0.0435
CNPY4	-0.65	1.96E-02	0.0433
TYW5	-0.65	1.19E-02	0.0304
STAG3L3	-0.65	1.43E-02	0.0345
SLC30A4	-0.65	1.93E-02	0.0429
RYK	-0.65	1.21E-02	0.0307
ARHGDIB	-0.65	1.92E-02	0.0427
TGFBR2	-0.65	2.29E-02	0.0483
GTF2IP1	-0.65	2.05E-02	0.0447
EBLN3P	-0.66	1.68E-02	0.0387
TRIM44	-0.66	2.23E-02	0.0474

FUS	-0.66	2.10E-02	0.0455
LCP2	-0.66	1.14E-02	0.0293
AL022311.1	-0.66	1.36E-02	0.0333
ZNF254	-0.66	1.79E-02	0.0405
ENST000003151831	-0.66	1.27E-02	0.0317
PRKCH	-0.66	9.12E-03	0.0252
MGAT5	-0.66	1.60E-02	0.0373
SECISBP2L	-0.66	1.44E-02	0.0347
RPL30	-0.67	6.36E-03	0.0197
AL390728.4	-0.67	1.10E-02	0.0287
FAM199X	-0.67	1.39E-02	0.0338
ATG12	-0.67	9.36E-03	0.0256
PCF11	-0.67	1.87E-02	0.0419
CLUAP1	-0.67	2.04E-02	0.0445
DOCK11	-0.67	1.05E-02	0.0277
PPM1A	-0.67	1.06E-02	0.0279
MLKL	-0.67	2.08E-02	0.0451
ENST000003677710	-0.67	1.71E-02	0.0393
NR2C1	-0.67	1.25E-02	0.0313
IFFO1	-0.67	2.26E-02	0.0478
COX10-AS1	-0.68	1.30E-02	0.0323
LUC7L3	-0.68	7.60E-03	0.0223
KANTR	-0.68	2.14E-02	0.0460
TOMM7	-0.68	1.42E-02	0.0344
ENST000002162642	-0.68	2.12E-02	0.0458
MFSD11	-0.68	2.16E-02	0.0463
VPS53	-0.68	2.11E-02	0.0455
ACVR1	-0.68	1.92E-02	0.0426
SPRYD4	-0.68	1.87E-02	0.0418
HIPK3	-0.68	1.24E-02	0.0311
PRPF38B	-0.68	1.29E-02	0.0322
NLRC5	-0.68	9.11E-03	0.0252
ADAM10	-0.68	1.29E-02	0.0321
SEPT7P2	-0.69	2.10E-02	0.0454
FTO	-0.69	8.45E-03	0.0239
AC092821.3	-0.69	2.19E-02	0.0468
FBXO25	-0.69	1.44E-02	0.0347
HECTD4	-0.69	1.63E-02	0.0379
RPS6KA5	-0.69	8.77E-03	0.0245
RAB18	-0.69	1.29E-02	0.0322
ENST000003084481	-0.69	1.42E-02	0.0343
CAMK2G	-0.69	1.70E-02	0.0391
ZNF37BP	-0.69	1.20E-02	0.0305
CBX7	-0.69	2.04E-02	0.0445
ZNF181	-0.69	1.54E-02	0.0363
HECA	-0.70	6.65E-03	0.0204
SULT1A1	-0.70	2.18E-02	0.0467

SAFB2	-0.70	1.13E-02	0.0292
ARAP2	-0.70	7.14E-03	0.0213
USP34	-0.70	1.05E-02	0.0277
ABHD17B	-0.70	1.20E-02	0.0304
TBC1D19	-0.70	1.38E-02	0.0336
AC013394.1	-0.70	1.90E-02	0.0424
HNRNPH1	-0.70	2.13E-02	0.0460
MSL2	-0.70	7.92E-03	0.0229
CD53	-0.70	1.34E-02	0.0330
ABCA5	-0.70	7.51E-03	0.0221
LINC00174	-0.70	2.00E-02	0.0439
VCPIP1	-0.70	1.54E-02	0.0364
VPS11	-0.70	1.17E-02	0.0298
ERC1	-0.70	8.85E-03	0.0246
RPS6KA3	-0.70	2.03E-02	0.0444
ENST000003267930	-0.70	1.37E-02	0.0335
CAPS2	-0.71	1.96E-02	0.0433
SARM1	-0.71	2.07E-02	0.0450
UST	-0.71	2.23E-02	0.0475
ZNF484	-0.71	1.97E-02	0.0435
THOC1	-0.71	8.18E-03	0.0234
PCSK7	-0.71	6.99E-03	0.0210
AC092652.2	-0.71	1.77E-02	0.0401
BCL2	-0.71	2.20E-02	0.0469
C20orf194	-0.71	1.92E-02	0.0426
RF00003.27	-0.71	9.68E-03	0.0262
RF00003.35	-0.71	9.68E-03	0.0262
RNU1-1	-0.71	9.68E-03	0.0262
RNU1-2	-0.71	9.68E-03	0.0262
RNU1-27P	-0.71	9.68E-03	0.0262
RNU1-28P	-0.71	9.68E-03	0.0262
RNU1-3	-0.71	9.68E-03	0.0262
RNU1-4	-0.71	9.68E-03	0.0262
RNVU1-18	-0.71	9.68E-03	0.0262
TRAF6	-0.71	8.57E-03	0.0241
ZNF566	-0.71	1.26E-02	0.0315
SLC39A10	-0.71	1.04E-02	0.0275
BEX2	-0.72	1.87E-02	0.0419
PRPF4B	-0.72	1.05E-02	0.0278
AC093752.1	-0.72	1.04E-02	0.0276
ZNF101	-0.72	7.87E-03	0.0228
PLXNA3	-0.72	1.81E-02	0.0409
ARID4A	-0.72	1.24E-02	0.0312
AL669831.5	-0.72	1.74E-02	0.0398
IDS	-0.72	1.53E-02	0.0363
EPM2AIP1	-0.72	7.12E-03	0.0213
ALG13	-0.72	2.21E-02	0.0471

KLHL36	-0.72	1.07E-02	0.0280
NBPF20	-0.72	1.04E-02	0.0276
SMG1P3	-0.72	1.04E-02	0.0275
FBXO7	-0.72	6.39E-03	0.0198
ENST000003128140	-0.73	1.02E-02	0.0272
ZNF527	-0.73	1.12E-02	0.0290
NAGK	-0.73	1.29E-02	0.0321
MTERF2	-0.73	1.59E-02	0.0373
TRAJ19	-0.73	2.16E-02	0.0463
TRBV7-9	-0.73	1.94E-02	0.0429
TAOK1	-0.73	5.45E-03	0.0178
TBCK	-0.73	9.44E-03	0.0258
COA1	-0.73	7.17E-03	0.0214
SMARCA2	-0.73	2.15E-02	0.0461
STYX	-0.74	7.97E-03	0.0230
RGS14	-0.74	1.12E-02	0.0291
ZNF430	-0.74	1.51E-02	0.0358
ISPD	-0.74	1.99E-02	0.0437
PIGC	-0.74	1.56E-02	0.0366
ENST000002644494	-0.74	6.16E-03	0.0193
ELOVL5	-0.74	1.15E-02	0.0296
CPEB2	-0.74	2.20E-02	0.0469
CDK17	-0.74	1.48E-02	0.0354
SMG1P1	-0.74	1.85E-02	0.0416
AL139385.1	-0.74	2.26E-02	0.0479
TMEM204	-0.74	1.82E-02	0.0411
NCOA1	-0.74	7.87E-03	0.0228
BCLAF1	-0.74	7.18E-03	0.0214
ENST000003180102	-0.74	1.78E-02	0.0404
AC024257.3	-0.74	2.29E-02	0.0483
TRMT2B	-0.74	1.70E-02	0.0391
GPATCH8	-0.74	1.95E-02	0.0432
SHPRH	-0.74	8.32E-03	0.0236
ARRB1	-0.74	1.58E-02	0.0371
OXR1	-0.75	1.47E-02	0.0353
YTHDC1	-0.75	6.44E-03	0.0199
TBC1D15	-0.75	1.09E-02	0.0285
AHSA2P	-0.75	1.62E-02	0.0377
ENST000002546050	-0.75	2.09E-02	0.0453
FBXW8	-0.75	2.19E-02	0.0469
ATXN1	-0.75	1.77E-02	0.0402
XPA	-0.75	7.96E-03	0.0230
SETD2	-0.75	1.75E-02	0.0398
NAF1	-0.75	1.45E-02	0.0349
OSER1	-0.75	1.18E-02	0.0301
STAM	-0.75	1.16E-02	0.0298
TMEM181	-0.76	5.81E-03	0.0186

TXNDC16	-0.76	1.67E-02	0.0385
RFX7	-0.76	2.07E-02	0.0449
HELQ	-0.76	1.59E-02	0.0372
ZNF75A	-0.76	1.76E-02	0.0401
WNK1	-0.76	8.49E-03	0.0240
ZNF737	-0.76	2.19E-02	0.0468
AC092910.3	-0.76	1.85E-02	0.0416
TBRG1	-0.76	7.04E-03	0.0212
MYCBP2	-0.76	3.96E-03	0.0145
KLHL20	-0.76	9.25E-03	0.0254
CROCC	-0.76	1.88E-02	0.0419
SFMBT1	-0.76	8.80E-03	0.0246
RAP2B	-0.76	2.16E-02	0.0463
GUSBP9	-0.76	1.68E-02	0.0387
ITM2A	-0.76	7.00E-03	0.0211
PTPN12	-0.77	1.73E-02	0.0396
AK9	-0.77	2.00E-02	0.0438
LINC01550	-0.77	2.13E-02	0.0459
DNAJB14	-0.77	4.27E-03	0.0152
ZNF274	-0.77	9.64E-03	0.0261
ENST00000638491	-0.77	1.08E-02	0.0283
ZNF518B	-0.77	8.60E-03	0.0242
PXYLP1	-0.77	1.30E-02	0.0323
GPR89B	-0.77	1.21E-02	0.0306
ARID4B	-0.77	6.90E-03	0.0208
TRAK2	-0.77	5.13E-03	0.0171
ANKRD23	-0.77	2.33E-02	0.0489
SNRPN	-0.77	2.37E-02	0.0496
ZNF81	-0.78	2.16E-02	0.0464
ZNF337	-0.78	7.09E-03	0.0212
PDXDC2P-NPIPB14P	-0.78	1.93E-02	0.0428
MLLT3	-0.78	1.52E-02	0.0361
ANKEF1	-0.78	2.02E-02	0.0442
RBSN	-0.78	9.24E-03	0.0254
POFUT2	-0.78	1.71E-02	0.0393
CPSF6	-0.78	5.03E-03	0.0169
MDM4	-0.78	1.67E-02	0.0385
CDK13	-0.78	4.76E-03	0.0163
UBR3	-0.78	4.49E-03	0.0157
TM2D3	-0.78	1.35E-02	0.0332
CNNM3	-0.78	1.59E-02	0.0371
JAK1	-0.78	4.46E-03	0.0156
PAQR3	-0.78	7.73E-03	0.0226
ABHD3	-0.78	1.25E-02	0.0314
TRAPPC9	-0.78	1.15E-02	0.0295
LENG1	-0.78	2.12E-02	0.0458
KMT2E	-0.78	1.73E-02	0.0395

Z93930.2	-0.78	1.96E-02	0.0432
EIF4A2	-0.78	1.21E-02	0.0307
ARGLU1	-0.78	4.70E-03	0.0162
ERVK3-1	-0.79	1.14E-02	0.0293
ALG11	-0.79	6.55E-03	0.0201
NRIP1	-0.79	1.32E-02	0.0326
MFSD8	-0.79	8.42E-03	0.0238
MORC2	-0.79	6.96E-03	0.0210
TCEA1P2	-0.79	1.58E-02	0.0371
MYLK3	-0.79	1.16E-02	0.0297
GSTO2	-0.79	1.98E-02	0.0435
TRBV2	-0.79	1.78E-02	0.0403
NT5DC1	-0.79	8.14E-03	0.0233
TTC17	-0.79	6.73E-03	0.0205
ZC2HC1A	-0.79	1.46E-02	0.0351
TMEM138	-0.79	1.46E-02	0.0350
CAPN7	-0.79	4.36E-03	0.0154
SNX15	-0.79	1.58E-02	0.0371
RBM39	-0.79	2.82E-03	0.0117
NR1H2	-0.80	1.39E-02	0.0338
PRDM1	-0.80	1.40E-02	0.0339
AMMECR1	-0.80	5.09E-03	0.0170
LINC01697	-0.80	2.30E-02	0.0484
ENST000003130281	-0.80	1.60E-02	0.0373
TMEM117	-0.80	1.54E-02	0.0364
RASA1	-0.80	1.27E-02	0.0317
AC004889.1	-0.80	1.57E-02	0.0370
SUMF1	-0.80	1.48E-02	0.0354
SH3TC1	-0.80	7.22E-03	0.0215
ENST000002213550	-0.80	1.31E-02	0.0324
SHISA5	-0.80	6.62E-03	0.0203
INO80D	-0.80	1.03E-02	0.0274
MPRIP	-0.80	4.46E-03	0.0156
KDM6A	-0.80	1.53E-02	0.0362
ENST000002582293	-0.80	5.12E-03	0.0171
HNRNPA3	-0.81	5.13E-03	0.0171
PIK3CA	-0.81	4.95E-03	0.0167
RNF214	-0.81	1.72E-02	0.0395
NR3C1	-0.81	8.51E-03	0.0240
STK4	-0.81	3.09E-03	0.0124
AFF4	-0.81	1.30E-02	0.0323
ERVW-1	-0.81	1.69E-02	0.0389
ZZEF1	-0.81	1.02E-02	0.0272
RUFY3	-0.81	1.01E-02	0.0269
ZNF33A	-0.81	1.03E-02	0.0274
ZNF134	-0.81	2.29E-02	0.0484
ENST000002822724	-0.81	1.05E-02	0.0276

ERCC6L2	-0.81	4.12E-03	0.0149
THUMPD3-AS1	-0.82	6.34E-03	0.0197
FBXO33	-0.82	4.53E-03	0.0158
FCHSD1	-0.82	1.49E-02	0.0356
PHYKPL	-0.82	2.32E-02	0.0488
ICA1	-0.82	1.91E-02	0.0426
LYSMD4	-0.82	8.95E-03	0.0249
LTB4R	-0.82	1.39E-02	0.0338
ENST000002315243	-0.82	6.22E-03	0.0194
SLC35E3	-0.82	1.79E-02	0.0405
SYS1	-0.82	1.73E-02	0.0396
KDM4C	-0.82	4.93E-03	0.0167
TRAV21	-0.82	1.87E-02	0.0418
R3HDM2	-0.82	1.85E-02	0.0415
IBA57	-0.82	8.15E-03	0.0233
NOTCH4	-0.82	2.25E-02	0.0477
PACS2	-0.82	1.60E-02	0.0374
CWF19L2	-0.82	4.78E-03	0.0164
SNORD13E	-0.82	2.31E-02	0.0486
UTP18	-0.82	1.01E-02	0.0269
ZCCHC14	-0.82	1.45E-02	0.0349
CLN5	-0.82	6.25E-03	0.0195
ZDHHC17	-0.82	4.29E-03	0.0153
ZNF782	-0.82	2.29E-02	0.0484
SUGT1P3	-0.82	1.29E-02	0.0321
FLI1	-0.82	3.75E-03	0.0140
USP6NL	-0.82	1.17E-02	0.0300
ENST000003526890	-0.82	3.64E-03	0.0137
ENST000003182894	-0.83	2.22E-02	0.0473
KCNQ1	-0.83	1.29E-02	0.0321
AL158152.1	-0.83	7.99E-03	0.0231
HELB	-0.83	9.66E-03	0.0262
ASH1L-AS1	-0.83	1.24E-02	0.0311
ELP4	-0.83	5.32E-03	0.0175
CCNL2	-0.83	5.99E-03	0.0190
GMDS-DT	-0.83	7.12E-03	0.0213
37316	-0.83	1.26E-02	0.0315
SLC12A9	-0.83	7.75E-03	0.0226
NORAD	-0.83	6.47E-03	0.0199
ENST000003569400	-0.83	4.85E-03	0.0165
AC087501.4	-0.83	2.08E-02	0.0451
ARHGAP5	-0.84	9.45E-03	0.0258
AFF1	-0.84	1.64E-02	0.0380
CLN3	-0.84	1.11E-02	0.0288
PDE4D	-0.84	1.84E-02	0.0414
DTNB	-0.84	1.42E-02	0.0344
BTBD8	-0.84	1.12E-02	0.0290

ENST000002880710	-0.84	1.33E-02	0.0328
MED17	-0.84	4.79E-03	0.0164
LINC01184	-0.84	6.78E-03	0.0206
BCO2	-0.84	2.09E-02	0.0453
BBX	-0.84	4.80E-03	0.0164
ENST000003485130	-0.84	8.27E-03	0.0235
TPM1	-0.84	1.71E-02	0.0393
ANKRD18A	-0.84	1.89E-02	0.0421
ENST000002263190	-0.84	1.92E-02	0.0427
C16orf72	-0.84	6.92E-03	0.0209
CSMD3	-0.84	1.38E-02	0.0336
ENST000003543630	-0.84	2.08E-02	0.0452
KLHL42	-0.84	2.38E-02	0.0497
ZNF780B	-0.84	5.01E-03	0.0169
AC118459.2	-0.84	2.18E-02	0.0466
ZNF75D	-0.84	4.75E-03	0.0163
NFRKB	-0.84	1.09E-02	0.0285
DDX3Y	-0.85	2.72E-03	0.0115
CD247	-0.85	5.30E-03	0.0175
NOP53	-0.85	1.55E-02	0.0365
AC093323.1	-0.85	1.09E-02	0.0285
SRSF6	-0.85	7.53E-03	0.0221
NDUFA6-DT	-0.85	8.99E-03	0.0249
KCNJ6	-0.85	2.31E-02	0.0486
TMUB2	-0.85	1.39E-02	0.0337
BRAF	-0.85	1.76E-02	0.0400
SZT2	-0.85	4.85E-03	0.0165
ENST000003365050	-0.85	1.65E-02	0.0382
PIGT	-0.85	9.41E-03	0.0257
ERCC6	-0.86	1.13E-02	0.0292
LRCH4	-0.86	1.54E-02	0.0364
DYRK1A	-0.86	2.86E-03	0.0118
AC027097.1	-0.86	9.77E-03	0.0263
ENST000003063780	-0.86	2.25E-02	0.0477
AP001029.2	-0.86	1.42E-02	0.0344
PIK3R3	-0.86	1.75E-02	0.0399
ZNF805	-0.86	1.99E-02	0.0437
GNPTG	-0.86	9.37E-03	0.0256
FBXL5	-0.86	3.68E-03	0.0138
IFNAR2	-0.86	1.04E-02	0.0275
ARPIN	-0.86	2.34E-02	0.0491
AL162171.3	-0.86	2.13E-02	0.0459
NPIPB5	-0.86	2.03E-02	0.0444
ZNF577	-0.86	1.83E-02	0.0413
CASD1	-0.86	5.48E-03	0.0179
RPS2P44	-0.86	1.90E-02	0.0423
PHC1	-0.86	6.06E-03	0.0191

SNX30	-0.86	4.27E-03	0.0152
APOL3	-0.86	3.58E-03	0.0136
DIS3L2	-0.86	6.02E-03	0.0191
ENST000003387450	-0.86	2.19E-02	0.0469
ANKLE1	-0.87	1.04E-02	0.0276
JPX	-0.87	5.44E-03	0.0178
KBTBD2	-0.87	4.12E-03	0.0149
ATP2A3	-0.87	8.64E-03	0.0243
OTUD1	-0.87	1.95E-02	0.0431
TRG-AS1	-0.87	9.27E-03	0.0254
ARL4A	-0.87	7.20E-03	0.0214
ENST000002568580	-0.87	1.38E-02	0.0337
TBC1D2	-0.87	1.77E-02	0.0403
EID3	-0.87	2.30E-02	0.0484
N4BP2L1	-0.87	1.23E-02	0.0310
RNASEH2B	-0.87	3.91E-03	0.0143
AC093827.5	-0.87	1.94E-02	0.0429
AC005899.1	-0.87	1.95E-02	0.0431
SERINC1	-0.87	4.03E-03	0.0146
HEATR5B	-0.87	4.80E-03	0.0164
SNX13	-0.87	3.09E-03	0.0124
NUBPL	-0.87	1.57E-02	0.0370
LINC00514	-0.87	2.03E-02	0.0444
ENST000003386371	-0.87	2.39E-02	0.0499
HLA-E	-0.87	2.08E-03	0.0098
LINC00652	-0.87	1.89E-02	0.0422
AC245452.1	-0.87	1.45E-02	0.0349
TSGA10	-0.87	1.31E-02	0.0324
CPEB3	-0.87	1.71E-02	0.0392
YAF2	-0.88	3.27E-03	0.0128
TBC1D8B	-0.88	4.59E-03	0.0159
RBM26	-0.88	4.97E-03	0.0168
SUCO	-0.88	5.71E-03	0.0184
ZMAT3	-0.88	4.01E-03	0.0146
ZNF549	-0.88	5.89E-03	0.0188
EML4	-0.88	2.71E-03	0.0114
CCDC69	-0.88	9.35E-03	0.0256
TARSL2	-0.88	9.92E-03	0.0266
AL158064.1	-0.88	2.17E-02	0.0466
KMT5B	-0.88	4.58E-03	0.0159
HEBP2	-0.88	9.03E-03	0.0250
SIVA1	-0.88	6.65E-03	0.0203
AL353622.1	-0.88	1.92E-02	0.0427
PPIEL	-0.88	2.34E-02	0.0492
NPHP3	-0.88	6.26E-03	0.0195
STAG3L1	-0.88	1.89E-02	0.0422
ENST000002981590	-0.88	1.66E-02	0.0384

ENST000003987520	-0.88	7.77E-03	0.0226
LINC00680	-0.88	1.09E-02	0.0285
GSTK1	-0.88	4.86E-03	0.0165
AKAP9	-0.88	2.05E-03	0.0097
MPP7	-0.88	1.12E-02	0.0290
KLHL7-DT	-0.88	6.79E-03	0.0206
PARVG	-0.88	3.58E-03	0.0136
APH1B	-0.88	6.32E-03	0.0196
INO80C	-0.88	1.01E-02	0.0270
ZNF335	-0.89	1.32E-02	0.0325
ENST000002801541	-0.89	3.82E-03	0.0141
MALAT1	-0.89	4.89E-03	0.0166
RTP4	-0.89	1.34E-02	0.0330
ENST000002860630	-0.89	1.80E-02	0.0407
RAB11FIP2	-0.89	6.52E-03	0.0201
NIN	-0.89	6.97E-03	0.0210
MINDY3	-0.89	3.62E-03	0.0137
MBTPS1	-0.89	5.00E-03	0.0168
ENST000003155851	-0.89	1.35E-02	0.0331
AC090114.2	-0.89	8.67E-03	0.0243
LENG8	-0.89	5.41E-03	0.0178
PLOD2	-0.89	1.81E-02	0.0410
ENST000002489010	-0.89	6.50E-03	0.0200
LUC7L	-0.89	3.68E-03	0.0138
STK17B	-0.89	2.78E-03	0.0116
AC010907.2	-0.89	2.11E-02	0.0456
IKZF1	-0.89	1.16E-02	0.0298
LINC01694	-0.89	1.07E-02	0.0280
MRPS30-DT	-0.89	1.42E-02	0.0343
ZNF586	-0.89	3.89E-03	0.0143
EDEM3	-0.89	8.22E-03	0.0234
CCDC191	-0.89	1.32E-02	0.0326
HLA-F-AS1	-0.89	2.19E-02	0.0469
SBDS	-0.89	5.45E-03	0.0178
ENST000003202203	-0.89	1.13E-02	0.0292
LINC00458	-0.89	1.57E-02	0.0369
TOM1L2	-0.89	2.14E-02	0.0460
BCL7A	-0.89	7.80E-03	0.0227
AKAP13	-0.89	5.83E-03	0.0187
AP001505.1	-0.89	1.77E-02	0.0402
ANKRD31	-0.89	1.71E-02	0.0392
ZNF554	-0.89	1.80E-02	0.0408
FBXO24	-0.89	1.88E-02	0.0421
TTC13	-0.89	4.86E-03	0.0165
BCDIN3D-AS1	-0.89	2.33E-02	0.0490
TTC3	-0.89	3.72E-03	0.0139
MON2	-0.90	2.58E-03	0.0111

PIAS1	-0.90	5.19E-03	0.0173
ZNF800	-0.90	3.09E-03	0.0124
HAAO	-0.90	1.91E-02	0.0425
ARSG	-0.90	8.24E-03	0.0235
ZNF611	-0.90	6.10E-03	0.0192
AC019155.2	-0.90	1.99E-02	0.0437
DPY19L1P1	-0.90	1.33E-02	0.0329
WWC3	-0.90	1.27E-02	0.0318
CCNT2	-0.90	2.64E-03	0.0113
SDHA	-0.90	4.02E-03	0.0146
AL590065.1	-0.90	1.37E-02	0.0335
ATRX	-0.90	1.81E-03	0.0090
RPAP2	-0.90	2.37E-03	0.0105
LIPG	-0.90	1.32E-02	0.0326
ZNF783	-0.90	1.03E-02	0.0272
TMEM213	-0.90	1.96E-02	0.0433
FITM2	-0.90	1.59E-02	0.0372
MANBA	-0.90	1.40E-02	0.0341
SUGT1	-0.90	6.52E-03	0.0201
AC087276.1	-0.90	1.95E-02	0.0431
ZNF573	-0.90	4.76E-03	0.0163
TPT1	-0.90	1.78E-03	0.0089
AC022558.2	-0.90	1.57E-02	0.0369
SEMA4C	-0.91	5.64E-03	0.0183
ENST000002908550	-0.91	5.42E-03	0.0178
THEMIS2	-0.91	6.94E-03	0.0209
FOXJ3	-0.91	6.19E-03	0.0194
RABL2A	-0.91	1.18E-02	0.0300
PLK3	-0.91	1.48E-02	0.0354
TOGARAM1	-0.91	4.93E-03	0.0167
TSPYL4	-0.91	5.63E-03	0.0182
HAUS3	-0.91	3.11E-03	0.0124
YY2	-0.91	1.71E-02	0.0392
AL136295.7	-0.91	9.21E-03	0.0254
ANK3	-0.91	2.57E-03	0.0111
PPP3CC	-0.91	3.03E-03	0.0122
TTC28-AS1	-0.91	8.37E-03	0.0237
ENST000002531592	-0.91	9.54E-03	0.0260
SNRPA1	-0.91	5.24E-03	0.0174
DENND1B	-0.91	2.79E-03	0.0116
STXBP4	-0.91	1.50E-02	0.0357
STIM2	-0.92	3.90E-03	0.0143
ZNF213-AS1	-0.92	1.70E-02	0.0391
TNKS	-0.92	7.20E-03	0.0214
PPP1R3E	-0.92	1.91E-02	0.0424
TBC1D10C	-0.92	3.94E-03	0.0144
OR1I1	-0.92	2.22E-02	0.0473

UXS1	-0.92	4.79E-03	0.0164
AL513523.1	-0.92	1.24E-02	0.0311
AL513523.5	-0.92	1.24E-02	0.0311
POLR2J3	-0.92	5.39E-03	0.0177
NBPF11	-0.92	1.97E-02	0.0434
AC027514.1	-0.92	1.74E-02	0.0398
MAN2B2	-0.92	7.97E-03	0.0230
SMAD5	-0.92	5.00E-03	0.0168
SLC27A1	-0.92	8.44E-03	0.0239
NPIPB4	-0.92	8.79E-03	0.0245
SPOCK2	-0.92	2.01E-03	0.0096
AC005838.2	-0.93	1.28E-02	0.0319
EPB41L5	-0.93	6.86E-03	0.0208
CAMKMT	-0.93	9.96E-03	0.0267
LFNG	-0.93	5.09E-03	0.0171
PPP1R12B	-0.93	7.73E-03	0.0226
RUFY2	-0.93	1.23E-02	0.0310
RCOR3	-0.93	9.14E-03	0.0252
CCDC169	-0.93	9.51E-03	0.0259
TMX4	-0.93	1.21E-02	0.0307
FAM47E	-0.93	1.22E-02	0.0308
AMN1	-0.93	1.88E-02	0.0419
NBPF12	-0.93	5.43E-03	0.0178
PGRMC2	-0.93	5.01E-03	0.0169
MOK	-0.93	1.61E-02	0.0375
DMTF1	-0.93	2.86E-03	0.0118
AC083798.2	-0.93	8.37E-03	0.0238
C9orf147	-0.93	1.16E-02	0.0297
ARHGAP4	-0.93	4.63E-03	0.0160
ENST000003430171	-0.93	1.42E-02	0.0345
ZNF862	-0.93	2.23E-02	0.0475
ZNF502	-0.94	1.97E-02	0.0435
AP005131.6	-0.94	2.17E-02	0.0464
COL6A2	-0.94	9.57E-03	0.0260
LINC01226	-0.94	5.35E-03	0.0176
DNAJB9	-0.94	4.44E-03	0.0156
KAT6A	-0.94	2.43E-03	0.0107
AC016831.6	-0.94	1.74E-02	0.0398
AC004812.2	-0.94	1.17E-02	0.0299
TEPSIN	-0.94	8.05E-03	0.0231
ENST000002619731	-0.94	2.46E-03	0.0108
NPEPL1	-0.94	9.48E-03	0.0258
ERBIN	-0.94	1.95E-03	0.0094
AL158154.2	-0.94	1.18E-02	0.0302
PHF21A	-0.94	2.26E-02	0.0479
AKNA	-0.94	1.77E-02	0.0402
LINC01748	-0.94	1.95E-02	0.0431

PSMB8-AS1	-0.95	4.54E-03	0.0158
ENST000003098630	-0.95	6.75E-03	0.0206
WDR26	-0.95	1.50E-02	0.0357
AC010601.1	-0.95	7.55E-03	0.0222
ENST000003582320	-0.95	8.65E-03	0.0243
PHACTR2	-0.95	2.30E-03	0.0104
AC018521.7	-0.95	2.18E-02	0.0467
KLF8	-0.95	1.79E-02	0.0405
AL133245.1	-0.95	1.78E-02	0.0404
PLPP2	-0.95	1.12E-02	0.0290
AC091078.3	-0.95	9.20E-03	0.0253
FYCO1	-0.95	8.39E-03	0.0238
AC084083.1	-0.95	1.02E-02	0.0271
SNORD15B	-0.95	6.19E-03	0.0194
MORC4	-0.95	4.64E-03	0.0161
AF131215.6	-0.95	1.44E-02	0.0348
RGPD6	-0.95	9.30E-03	0.0255
SLFN12L	-0.95	8.85E-03	0.0246
AC137932.3	-0.96	1.23E-02	0.0311
ARL2BP	-0.96	1.58E-02	0.0371
HSPBAP1	-0.96	8.78E-03	0.0245
OR6C75	-0.96	2.05E-02	0.0447
AL390838.1	-0.96	1.50E-02	0.0358
DNAJC27-AS1	-0.96	2.06E-02	0.0448
SLC38A6	-0.96	1.31E-02	0.0325
ZBTB1	-0.96	2.09E-03	0.0098
ZAP70	-0.96	2.18E-03	0.0100
ENST000002889851	-0.96	2.83E-03	0.0117
AC004834.1	-0.96	1.36E-02	0.0333
NIPBL	-0.96	1.86E-03	0.0092
CCSER2	-0.96	1.98E-03	0.0095
HLA-DOA	-0.96	1.40E-02	0.0341
RSBN1	-0.96	3.91E-03	0.0143
PIGS	-0.96	6.08E-03	0.0192
ASH1L	-0.96	4.37E-03	0.0155
AC130466.1	-0.96	1.74E-02	0.0397
AC092326.1	-0.96	1.40E-02	0.0340
AC246789.2	-0.96	1.40E-02	0.0340
AL138889.1	-0.96	1.01E-02	0.0270
ZNF487	-0.96	6.56E-03	0.0201
ENST000002215612	-0.97	8.19E-03	0.0234
AL163051.2	-0.97	1.15E-02	0.0295
LINC02073	-0.97	1.07E-02	0.0281
CLIP4	-0.97	4.81E-03	0.0164
CIRBP	-0.97	9.40E-03	0.0257
TPD52L1	-0.97	1.86E-02	0.0417
MAN2A1	-0.97	1.80E-03	0.0090

TRBV5-1	-0.97	2.01E-02	0.0441
AC117382.1	-0.97	1.00E-02	0.0267
STXBP5	-0.97	2.60E-03	0.0112
TRBV18	-0.97	9.47E-03	0.0258
CRYBG1	-0.97	2.99E-03	0.0121
LINC01902	-0.97	1.56E-02	0.0368
MNT	-0.97	7.58E-03	0.0222
TMEM80	-0.97	8.90E-03	0.0247
PROX1-AS1	-0.97	1.14E-02	0.0293
MAP4K4	-0.97	3.51E-03	0.0134
CDRT4	-0.97	1.18E-02	0.0301
ZNF655	-0.97	2.21E-03	0.0101
STAG3L5P	-0.97	2.95E-03	0.0120
CCDC122	-0.97	7.22E-03	0.0215
TMEM245	-0.97	4.36E-03	0.0154
BCL2L15	-0.97	1.31E-02	0.0325
ENST000003438820	-0.97	7.78E-03	0.0226
HIP1R	-0.98	3.75E-03	0.0140
GCSAM	-0.98	4.42E-03	0.0155
PTS	-0.98	3.01E-03	0.0122
ARID2	-0.98	5.63E-03	0.0182
PPP1R13B	-0.98	9.76E-03	0.0263
AC138409.2	-0.98	6.03E-03	0.0191
EIF5A2	-0.98	1.51E-02	0.0358
LINC02274	-0.98	1.44E-02	0.0348
CNKSR2	-0.98	6.25E-03	0.0195
AC087623.2	-0.98	2.03E-02	0.0444
ENST000003084880	-0.98	2.83E-03	0.0117
MICAL1	-0.98	5.12E-03	0.0171
HELZ	-0.98	9.30E-03	0.0255
PRMT2	-0.98	1.39E-02	0.0338
GLG1	-0.98	2.07E-03	0.0097
ENST000002633980	-0.98	2.19E-03	0.0100
EIF5	-0.98	3.76E-03	0.0140
USHBP1	-0.98	1.62E-02	0.0377
DIP2A	-0.98	4.52E-03	0.0158
TRAJ25	-0.98	2.19E-02	0.0468
ZNF609	-0.98	3.89E-03	0.0143
H6PD	-0.98	9.91E-03	0.0266
TSIX	-0.98	7.15E-03	0.0213
STK33	-0.98	7.62E-03	0.0223
RASA2	-0.99	1.53E-03	0.0082
JMJD1C	-0.99	4.19E-03	0.0151
AL805961.1	-0.99	2.28E-02	0.0482
AL627230.2	-0.99	2.19E-02	0.0468
TLL2	-0.99	2.12E-02	0.0457
ENST00000623111	-0.99	6.14E-03	0.0193

IL6R	-0.99	8.99E-03	0.0249
MTERF4	-0.99	7.02E-03	0.0211
CEP95	-0.99	2.32E-03	0.0104
CLIP2	-0.99	1.14E-02	0.0293
CTNNA2	-0.99	1.64E-02	0.0380
BCAS4	-0.99	4.10E-03	0.0149
TBCEL	-0.99	5.59E-03	0.0181
C3orf62	-0.99	6.40E-03	0.0198
LEF1-AS1	-0.99	5.58E-03	0.0181
HSPA1L	-0.99	8.84E-03	0.0246
PLEKHA5	-0.99	2.50E-03	0.0109
PRKCQ-AS1	-0.99	3.05E-03	0.0123
TMC7	-0.99	1.62E-02	0.0377
AC005304.2	-0.99	6.41E-03	0.0198
NXPE3	-0.99	3.43E-03	0.0132
ENAH	-0.99	2.12E-02	0.0457
TRPM3	-0.99	1.02E-02	0.0272
ENST000002861860	-0.99	8.35E-03	0.0237
LINC00852	-0.99	2.32E-02	0.0488
ZBTB25	-0.99	1.66E-03	0.0086
LINC00923	-1.00	2.07E-02	0.0450
TTC32	-1.00	6.04E-03	0.0191
CFAP70	-1.00	8.21E-03	0.0234
PCDHA4	-1.00	4.49E-03	0.0157
FCHSD2	-1.00	2.31E-02	0.0487
TDRD3	-1.00	2.28E-03	0.0103
ITGA9-AS1	-1.00	8.15E-03	0.0233
TMEM131	-1.00	2.42E-03	0.0107
TFCP2L1	-1.00	1.30E-02	0.0323
ENST000003173361	-1.00	5.94E-03	0.0189
IRF1	-1.00	1.81E-03	0.0090
AC011337.1	-1.00	2.04E-02	0.0445
THEM4	-1.00	2.77E-03	0.0116
AL132656.2	-1.00	7.96E-03	0.0230
MRTFB	-1.00	2.73E-03	0.0115
CFAP69	-1.00	9.90E-03	0.0266
ENST000003108621	-1.00	1.12E-02	0.0291
AL157392.3	-1.00	2.06E-02	0.0449
ATG14	-1.00	3.06E-03	0.0123
VGLL3	-1.00	2.10E-02	0.0455
UBXN4	-1.00	3.76E-03	0.0140
PIK3CB	-1.00	6.68E-03	0.0204
AC007620.2	-1.00	1.57E-02	0.0369
ENST000003008230	-1.00	1.37E-02	0.0336
ZNF19	-1.00	1.53E-02	0.0363
ABCB9	-1.00	8.49E-03	0.0240
SLC2A1-AS1	-1.01	1.27E-02	0.0318

AL117378.1	-1.01	9.06E-03	0.0251
AC009120.2	-1.01	7.14E-03	0.0213
FRMD3	-1.01	1.69E-02	0.0388
ADGRF1	-1.01	8.20E-03	0.0234
ADAMTSL4-AS1	-1.01	9.53E-03	0.0259
GNAQ	-1.01	1.91E-03	0.0093
SNHG14	-1.01	1.01E-02	0.0269
AC008938.1	-1.01	1.50E-02	0.0357
LAPTM5	-1.01	1.37E-02	0.0335
TMEM45B	-1.01	9.11E-03	0.0252
MORC3	-1.01	1.46E-03	0.0080
CDC37L1-DT	-1.01	1.52E-02	0.0361
AC079316.1	-1.01	2.05E-02	0.0447
SERPINA10	-1.01	5.46E-03	0.0179
C14orf28	-1.01	9.25E-03	0.0254
RELA	-1.01	1.07E-02	0.0282
ANKRD49	-1.01	1.77E-03	0.0089
ETFRF1	-1.01	7.51E-03	0.0221
CNST	-1.01	1.97E-03	0.0095
TGFB1	-1.01	4.50E-03	0.0157
AKTIP	-1.01	4.26E-03	0.0152
DENND2A	-1.01	1.63E-02	0.0379
ZNF407	-1.01	1.90E-03	0.0093
CARF	-1.01	6.96E-03	0.0210
BLMH	-1.02	4.28E-03	0.0153
EVI2A	-1.02	3.23E-03	0.0127
LINC01609	-1.02	1.13E-02	0.0292
ASPH	-1.02	4.35E-03	0.0154
SLC2A11	-1.02	8.32E-03	0.0236
DDR1-DT	-1.02	4.33E-03	0.0154
ARL17B	-1.02	4.48E-03	0.0157
UGCG	-1.02	3.01E-03	0.0122
CCDC88C	-1.02	4.66E-03	0.0161
TESPA1	-1.02	1.70E-02	0.0392
IGIP	-1.02	4.98E-03	0.0168
SCARNA13	-1.02	3.72E-03	0.0139
TSPYL1	-1.02	2.15E-03	0.0099
SUGT1P4-STRA6LP	-1.02	5.36E-03	0.0176
ATP8B2	-1.02	6.35E-03	0.0197
HMGB1P3	-1.02	1.21E-02	0.0307
BTBD9	-1.02	7.01E-03	0.0211
ELF2	-1.02	2.45E-03	0.0108
AC009318.1	-1.02	1.99E-02	0.0437
SLC35D2	-1.03	1.07E-02	0.0280
AC011474.1	-1.03	2.24E-02	0.0476
IFT80	-1.03	1.77E-03	0.0089
SERTAD2	-1.03	6.68E-03	0.0204

APLF	-1.03	1.86E-02	0.0417
FKBP11	-1.03	4.05E-03	0.0147
NSUN5P2	-1.03	4.23E-03	0.0152
AL359955.1	-1.03	1.48E-02	0.0355
RUNX1	-1.03	3.90E-03	0.0143
OSBPL7	-1.03	1.93E-02	0.0428
NKD1	-1.03	1.72E-02	0.0394
ZNF280D	-1.03	3.08E-03	0.0123
ATF7IP	-1.03	3.81E-03	0.0141
CASC15	-1.03	1.41E-02	0.0341
MIR100HG	-1.03	7.52E-03	0.0221
TRAJ12	-1.03	7.11E-03	0.0213
AC100774.1	-1.03	1.11E-02	0.0288
AC011511.1	-1.03	1.46E-02	0.0351
MTMR10	-1.03	2.65E-03	0.0113
TAS2R20	-1.03	1.54E-02	0.0365
TUG1	-1.03	1.49E-03	0.0081
AC116351.1	-1.03	8.40E-03	0.0238
AC116351.3	-1.03	8.40E-03	0.0238
C9orf139	-1.04	2.26E-02	0.0478
YLPM1	-1.04	2.11E-03	0.0098
PKN2	-1.04	2.58E-03	0.0111
ENST000003601561	-1.04	5.04E-03	0.0169
MAP2K5	-1.04	6.14E-03	0.0193
FAR2P2	-1.04	1.49E-02	0.0355
MBTD1	-1.04	1.97E-03	0.0095
ZFAND6	-1.04	1.35E-03	0.0076
SYNRG	-1.04	2.23E-03	0.0101
CERKL	-1.04	1.71E-02	0.0393
SLC25A53	-1.04	4.57E-03	0.0159
PLEKHF2	-1.04	1.58E-03	0.0083
FAM66E	-1.04	1.84E-02	0.0414
TEX9	-1.04	1.43E-02	0.0346
CPEB1	-1.04	1.56E-02	0.0368
MATN1-AS1	-1.04	1.55E-02	0.0365
ENST000002678071	-1.04	2.81E-03	0.0117
TLK1	-1.04	2.16E-03	0.0099
SPIB	-1.04	1.35E-02	0.0332
SMURF2	-1.04	4.25E-03	0.0152
PCMTD2	-1.04	9.86E-03	0.0265
ENST000003142611	-1.04	4.19E-03	0.0151
RNASEL	-1.04	2.50E-03	0.0109
FAM160B1	-1.04	2.97E-03	0.0121
RGL4	-1.04	1.87E-02	0.0418
HERC2P3	-1.04	1.30E-02	0.0323
AC092017.4	-1.04	9.99E-03	0.0267
AC002558.3	-1.05	9.62E-03	0.0261

ASAH1	-1.05	4.75E-03	0.0163
NPIPB13	-1.05	2.12E-02	0.0457
ENST00000641520	-1.05	5.31E-03	0.0175
AC018647.2	-1.05	5.93E-03	0.0189
SAMMSON	-1.05	1.62E-02	0.0378
LINC01579	-1.05	1.93E-02	0.0428
LINC01761	-1.05	6.98E-03	0.0210
PAXIP1-AS2	-1.05	5.20E-03	0.0173
CACNA1A	-1.05	6.75E-03	0.0206
FNDC3B	-1.05	2.58E-03	0.0111
RBM48	-1.05	2.50E-03	0.0109
XAF1	-1.05	8.03E-03	0.0231
CHD6	-1.05	1.82E-03	0.0090
SRP54-AS1	-1.05	6.06E-03	0.0191
HGSNAT	-1.05	6.37E-03	0.0197
TRBV12-3	-1.05	1.92E-02	0.0427
ILKAP	-1.05	3.08E-03	0.0123
MOB2	-1.05	4.05E-03	0.0147
IL16	-1.05	2.97E-03	0.0121
TTTY15	-1.05	1.43E-03	0.0079
AC011726.2	-1.05	9.52E-03	0.0259
ZBTB8B	-1.05	9.12E-03	0.0252
AC012435.1	-1.05	1.19E-02	0.0304
DAGLB	-1.05	1.20E-02	0.0305
AC069222.1	-1.05	1.21E-02	0.0308
CHIC1	-1.05	6.88E-03	0.0208
WTIP	-1.05	5.24E-03	0.0174
ZNF92	-1.05	4.19E-03	0.0151
CLSTN1	-1.05	8.32E-03	0.0236
AC245078.1	-1.05	1.15E-02	0.0295
SRGAP1	-1.05	8.98E-03	0.0249
PLCD1	-1.05	4.60E-03	0.0160
GRTP1	-1.06	1.90E-02	0.0423
ENST000002971090	-1.06	6.26E-03	0.0195
KLHDC2	-1.06	2.33E-03	0.0104
ENST000003443370	-1.06	2.82E-03	0.0117
OR2G6	-1.06	7.22E-03	0.0215
CROCCP3	-1.06	5.15E-03	0.0172
LRRC39	-1.06	1.58E-02	0.0370
RIC3	-1.06	6.18E-03	0.0194
AL080317.1	-1.06	2.15E-02	0.0461
WAC-AS1	-1.06	2.13E-03	0.0099
AC012213.2	-1.06	1.40E-02	0.0341
SMC2-AS1	-1.06	5.78E-03	0.0186
NAV1	-1.06	4.11E-03	0.0149
ZNF792	-1.06	1.02E-02	0.0272
AL355312.2	-1.06	2.29E-02	0.0483

SCARNA2	-1.06	4.39E-03	0.0155
MBNL3	-1.06	2.64E-03	0.0113
CCNL2P1	-1.06	1.85E-02	0.0416
HCG27	-1.06	1.22E-02	0.0309
ARHGAP45	-1.06	1.77E-03	0.0089
ECHDC2	-1.07	4.56E-03	0.0159
BICD1	-1.07	1.62E-02	0.0378
RREB1	-1.07	7.11E-03	0.0213
PCIF1	-1.07	5.93E-03	0.0189
YBX1	-1.07	1.75E-02	0.0399
ARRDC3-AS1	-1.07	2.01E-02	0.0440
LDLRAD4	-1.07	1.98E-03	0.0095
ENST000003357273	-1.07	2.13E-02	0.0459
SUCLG2-AS1	-1.07	1.58E-02	0.0370
BTD	-1.07	3.83E-03	0.0142
TMC4	-1.07	2.16E-02	0.0463
CCDC186	-1.07	1.13E-03	0.0069
POPDC2	-1.07	1.14E-02	0.0293
FRS2	-1.07	3.94E-03	0.0144
NMT2	-1.07	2.84E-03	0.0117
SRRM1	-1.07	2.58E-03	0.0111
AC009120.1	-1.07	1.43E-02	0.0346
AC092447.5	-1.07	1.15E-02	0.0295
KANK1	-1.07	3.01E-03	0.0122
ORMDL1	-1.07	2.40E-03	0.0107
OR2A5	-1.07	2.02E-02	0.0442
AC105429.1	-1.07	7.37E-03	0.0218
AC108676.1	-1.07	5.86E-03	0.0187
BAALC-AS1	-1.08	1.44E-02	0.0347
B4GALNT2	-1.08	4.83E-03	0.0165
AC015971.1	-1.08	1.47E-02	0.0352
CAPRIN2	-1.08	2.55E-03	0.0111
VPS8	-1.08	1.90E-03	0.0093
ENST000003006191	-1.08	1.37E-03	0.0077
TECPR1	-1.08	1.66E-03	0.0086
GCC2	-1.08	3.15E-03	0.0125
OR52K1	-1.08	6.28E-03	0.0195
AC004918.1	-1.08	8.32E-03	0.0236
AC004918.2	-1.08	8.32E-03	0.0236
AGO4	-1.08	1.41E-03	0.0078
AC034213.1	-1.08	5.98E-03	0.0190
ETS1	-1.08	1.02E-03	0.0065
LIPT1	-1.08	5.21E-03	0.0173
NECAP1	-1.08	1.83E-03	0.0091
HSBP1L1	-1.08	1.23E-02	0.0311
SNX18P3	-1.08	4.34E-03	0.0154
HSD17B11	-1.08	3.29E-03	0.0129

GUSBP2	-1.08	5.52E-03	0.0180
ENST000002646580	-1.08	8.21E-03	0.0234
ENST000002950950	-1.08	9.86E-04	0.0064
LINC01695	-1.08	6.83E-03	0.0207
P2RY10	-1.08	1.24E-03	0.0073
OR7D4	-1.08	1.84E-02	0.0414
FBXO44	-1.08	1.27E-02	0.0317
B3GALT4	-1.08	1.51E-02	0.0358
CEMIP2	-1.08	4.26E-03	0.0152
ESYT2	-1.08	1.07E-03	0.0067
AC008982.2	-1.08	1.72E-02	0.0394
AC008982.4	-1.08	1.72E-02	0.0394
FAM120C	-1.08	1.53E-02	0.0362
TIGD1	-1.08	2.01E-03	0.0096
CELF2	-1.08	1.12E-03	0.0068
PINK1	-1.08	5.41E-03	0.0178
WDPCP	-1.09	2.44E-03	0.0107
RTKN2	-1.09	3.46E-03	0.0133
DIRC2	-1.09	8.30E-03	0.0236
ZNF493	-1.09	6.94E-03	0.0209
AL022323.4	-1.09	3.78E-03	0.0141
TAS2R15P	-1.09	8.09E-03	0.0232
BEX4	-1.09	3.46E-03	0.0133
ZFYVE28	-1.09	4.64E-03	0.0161
RHOH	-1.09	1.98E-03	0.0095
AC099811.1	-1.09	1.33E-02	0.0328
AC091491.1	-1.09	2.25E-02	0.0477
ENST000002640651	-1.09	2.34E-03	0.0105
PRORSD1P	-1.09	6.35E-03	0.0197
ZSWIM7	-1.09	1.86E-02	0.0417
JAZF1	-1.09	1.40E-02	0.0340
AC124312.5	-1.09	2.52E-03	0.0110
DDX59	-1.09	3.90E-03	0.0143
ZFP3	-1.09	5.54E-03	0.0181
ENST000003558390	-1.09	1.63E-02	0.0379
AC139494.1	-1.09	3.83E-03	0.0142
L3HYPDH	-1.09	3.32E-03	0.0129
PATL2	-1.09	3.37E-03	0.0131
MICU3	-1.09	6.44E-03	0.0199
BTN3A2	-1.10	1.84E-02	0.0414
ZNF14	-1.10	7.07E-03	0.0212
AC139769.1	-1.10	1.58E-02	0.0371
AC015969.1	-1.10	1.92E-02	0.0426
FMR1	-1.10	2.49E-03	0.0109
CEACAM22P	-1.10	1.12E-02	0.0290
TRIM23	-1.10	1.97E-02	0.0435
LINC01238	-1.10	8.12E-03	0.0233

CMTM8	-1.10	6.87E-03	0.0208
SLC4A8	-1.10	9.56E-03	0.0260
AC105760.2	-1.10	8.04E-03	0.0231
PNISR	-1.10	1.45E-03	0.0080
AC092656.1	-1.10	8.25E-03	0.0235
AP002387.1	-1.10	8.03E-03	0.0231
AL353743.1	-1.10	1.20E-02	0.0305
WASL	-1.10	1.05E-02	0.0278
SWT1	-1.10	6.04E-03	0.0191
TMEM131L	-1.10	2.89E-03	0.0119
OR4F17	-1.10	8.86E-03	0.0246
AC104109.4	-1.10	9.38E-03	0.0257
SHISA9	-1.10	1.02E-02	0.0272
AC108866.1	-1.10	2.32E-02	0.0488
UBE2Q2	-1.10	2.10E-03	0.0098
AL035071.1	-1.10	6.99E-03	0.0210
DMAP1	-1.10	3.16E-03	0.0125
PSTK	-1.10	3.70E-03	0.0139
ATP8A1	-1.10	8.71E-03	0.0244
ZNF83	-1.10	1.27E-03	0.0074
AL360270.2	-1.10	1.87E-03	0.0092
AC004817.4	-1.10	3.76E-03	0.0140
DNMBP-AS1	-1.10	1.27E-02	0.0318
RBMS1	-1.10	8.19E-03	0.0234
RBM43	-1.10	8.75E-03	0.0245
CCDC66	-1.10	2.44E-03	0.0107
ZNF362	-1.10	7.04E-03	0.0211
RN7SKP95	-1.11	1.59E-02	0.0373
DNASE1	-1.11	3.87E-03	0.0143
SIK3	-1.11	2.59E-03	0.0112
RNF216	-1.11	2.65E-03	0.0113
CLK4	-1.11	1.48E-03	0.0080
IQSEC1	-1.11	1.51E-02	0.0359
PTCHD1	-1.11	7.98E-03	0.0230
AP001059.2	-1.11	1.41E-02	0.0342
NAP1L3	-1.11	8.23E-03	0.0235
AC021086.1	-1.11	2.25E-02	0.0477
CR383656.13	-1.11	1.35E-02	0.0332
ENST000002621890	-1.11	6.97E-03	0.0210
ERVK9-11	-1.11	9.51E-03	0.0259
TEN1-CDK3	-1.11	3.75E-03	0.0140
FCAR	-1.11	1.81E-02	0.0408
FLT3LG	-1.11	9.09E-03	0.0251
ATP10B	-1.11	8.80E-03	0.0246
TSPAN6	-1.11	7.61E-03	0.0223
SH3BGRL	-1.11	1.21E-03	0.0072
NRBP2	-1.11	1.82E-02	0.0410

SBDSP1	-1.11	5.04E-03	0.0169
AP000866.6	-1.11	9.19E-03	0.0253
CBX4	-1.11	4.81E-03	0.0164
KATNAL2	-1.11	1.25E-02	0.0314
AC099518.5	-1.12	2.13E-02	0.0458
PELI1	-1.12	1.69E-03	0.0087
AC002519.1	-1.12	7.20E-03	0.0214
LINC02193	-1.12	1.64E-02	0.0381
OSER1-DT	-1.12	3.96E-03	0.0145
AC007000.1	-1.12	2.22E-02	0.0473
MEF2A	-1.12	3.01E-03	0.0122
LRRC37B	-1.12	4.39E-03	0.0155
LINC02018	-1.12	9.23E-03	0.0254
HERC2P9	-1.12	4.68E-03	0.0162
SMURF2P1	-1.12	9.13E-03	0.0252
AP000866.1	-1.12	4.62E-03	0.0160
NAA16	-1.12	1.58E-03	0.0083
AL356481.3	-1.12	1.11E-02	0.0288
GHET1	-1.12	3.64E-03	0.0137
CCBE1	-1.12	9.39E-03	0.0257
38777	-1.12	1.86E-03	0.0092
SCARNA9	-1.12	1.54E-03	0.0082
AC084855.2	-1.12	1.96E-02	0.0433
AL117379.1	-1.12	1.96E-02	0.0433
NOSIP	-1.12	1.57E-03	0.0083
AL590666.3	-1.12	1.59E-02	0.0372
ZNF571	-1.12	3.68E-03	0.0138
PCDHA9	-1.12	1.32E-02	0.0326
SUSD1	-1.12	3.91E-03	0.0144
ENST000003160051	-1.12	1.45E-02	0.0349
CXorf65	-1.13	5.15E-03	0.0172
ENST000002959000	-1.13	1.15E-02	0.0296
AC005332.6	-1.13	1.11E-02	0.0288
HERPUD2	-1.13	1.39E-03	0.0077
SMG1P4	-1.13	5.75E-03	0.0185
DGKD	-1.13	4.55E-03	0.0158
ZNF251	-1.13	3.28E-03	0.0128
PLEKHM3	-1.13	2.76E-03	0.0116
UGGT2	-1.13	1.05E-02	0.0277
AL358334.2	-1.13	1.05E-02	0.0278
WDFY2	-1.13	1.96E-03	0.0094
KAT2B	-1.13	3.92E-03	0.0144
TRAJ42	-1.13	9.17E-03	0.0253
PLCB2	-1.13	1.87E-02	0.0418
CLDND1	-1.13	9.57E-03	0.0260
BTBD11	-1.13	7.32E-03	0.0217
KCNQ1OT1	-1.13	2.62E-03	0.0112

ATP6V1G1	-1.13	1.76E-03	0.0089
ANKRD12	-1.13	8.95E-04	0.0061
FOCAD	-1.13	2.08E-02	0.0451
TRAJ4	-1.13	2.24E-02	0.0475
TINCR	-1.13	1.94E-02	0.0429
SEPT2	-1.13	2.50E-03	0.0109
GBP4	-1.13	7.60E-03	0.0223
AC124312.4	-1.13	1.56E-02	0.0368
MLXIP	-1.13	2.72E-03	0.0115
ADGRV1	-1.13	8.79E-03	0.0245
CYTH1	-1.13	1.18E-03	0.0070
TAS2R19	-1.13	1.22E-02	0.0308
AC025171.1	-1.13	7.37E-03	0.0218
AC017081.4	-1.13	1.12E-02	0.0290
DBP	-1.13	5.01E-03	0.0169
MBP	-1.13	1.79E-03	0.0090
AL450384.2	-1.14	6.00E-03	0.0190
OR5AU1	-1.14	1.83E-02	0.0413
RNF216P1	-1.14	2.24E-03	0.0102
AL136038.4	-1.14	1.37E-02	0.0336
USP9Y	-1.14	7.34E-04	0.0055
MGC27382	-1.14	3.69E-03	0.0139
ZNF641	-1.14	3.00E-03	0.0121
EFCAB6	-1.14	1.02E-02	0.0272
UBL3	-1.14	2.81E-03	0.0117
KRT18P31	-1.14	6.81E-03	0.0207
HIVEP2	-1.14	1.36E-03	0.0076
CAPN2	-1.14	1.24E-03	0.0073
AC090515.2	-1.14	9.27E-03	0.0254
RAB30	-1.14	1.99E-02	0.0438
CDC42SE2	-1.14	8.29E-04	0.0058
AC004918.3	-1.14	3.79E-03	0.0141
AC007780.1	-1.14	1.34E-02	0.0329
GREM1	-1.14	5.25E-03	0.0174
AL589642.2	-1.15	3.45E-03	0.0132
AL117336.3	-1.15	6.91E-03	0.0209
AP000640.2	-1.15	8.59E-03	0.0242
AC097359.2	-1.15	1.97E-02	0.0435
ZNF345	-1.15	6.82E-03	0.0207
CCNH	-1.15	9.29E-04	0.0062
SEPSECS-AS1	-1.15	1.58E-03	0.0083
AL450384.1	-1.15	2.23E-02	0.0474
SLC25A21	-1.15	2.20E-02	0.0470
KANK2	-1.15	6.49E-03	0.0200
TMEM110-MUSTN1	-1.15	1.07E-02	0.0280
FAM95C	-1.15	5.81E-03	0.0186
AC138393.1	-1.15	1.30E-02	0.0323

CSF2RB	-1.15	6.75E-03	0.0206
AP001469.3	-1.15	4.78E-03	0.0164
GXYLT2	-1.15	1.72E-02	0.0394
SMIM31	-1.15	1.80E-02	0.0408
KIAA0355	-1.15	1.43E-03	0.0079
ZNF224	-1.15	3.82E-03	0.0141
RPL29P14	-1.15	1.90E-02	0.0423
LYSMD3	-1.15	6.02E-03	0.0191
PDE3A	-1.15	9.91E-03	0.0266
COL5A3	-1.15	1.09E-02	0.0285
ATXN7L3B	-1.15	2.10E-03	0.0098
ZNF880	-1.15	9.09E-03	0.0251
FBXL2	-1.16	1.97E-02	0.0434
LMCD1-AS1	-1.16	7.00E-03	0.0211
TUBA3FP	-1.16	2.09E-02	0.0452
MAN1A2	-1.16	1.61E-03	0.0084
PPP1CB	-1.16	1.94E-03	0.0094
AC040162.1	-1.16	2.79E-03	0.0116
AC243960.11	-1.16	1.40E-02	0.0339
CRIP1	-1.16	1.02E-02	0.0272
AC016582.3	-1.16	3.05E-03	0.0123
AC025031.4	-1.16	1.83E-02	0.0413
LINC02068	-1.16	8.70E-03	0.0244
LINC00939	-1.16	8.72E-03	0.0244
PLEKHM1P1	-1.16	1.93E-03	0.0094
TRAJ50	-1.16	7.78E-03	0.0226
LINC01427	-1.16	1.19E-02	0.0303
DICER1-AS1	-1.16	5.57E-03	0.0181
ENST000003152510	-1.16	9.29E-03	0.0255
AC021016.2	-1.16	3.44E-03	0.0132
RBM33	-1.16	2.69E-03	0.0114
RPL7L1P10	-1.16	1.85E-02	0.0416
REPS2	-1.16	4.53E-03	0.0158
MX2	-1.16	1.19E-02	0.0304
CA5A	-1.16	7.63E-03	0.0223
CNBD2	-1.16	1.20E-02	0.0304
CSF2RA	-1.16	4.83E-03	0.0165
ENST000003739540	-1.16	2.41E-03	0.0107
KCNV1	-1.16	7.03E-03	0.0211
CHMP1B	-1.16	9.80E-04	0.0064
TRAPPC3L	-1.16	2.58E-03	0.0111
AC006509.1	-1.16	8.79E-03	0.0245
LINC01362	-1.17	1.94E-02	0.0430
RPS27	-1.17	6.35E-04	0.0051
TUT4	-1.17	3.71E-03	0.0139
ANOS2P	-1.17	1.62E-02	0.0377
NBPF9	-1.17	6.84E-04	0.0053

GARS-DT	-1.17	3.47E-03	0.0133
EFHC1	-1.17	3.38E-03	0.0131
LINC01537	-1.17	4.96E-03	0.0167
FBXW7	-1.17	8.17E-04	0.0058
C9orf72	-1.17	2.07E-03	0.0097
TJP1	-1.17	2.20E-02	0.0470
DHRS1	-1.17	7.49E-03	0.0221
FBXO11	-1.17	2.03E-03	0.0096
ADGRE2	-1.17	5.06E-03	0.0170
LINC01722	-1.17	3.85E-03	0.0142
WNT2B	-1.17	1.19E-02	0.0304
ENST000002621330	-1.17	5.98E-03	0.0190
RPRD2	-1.17	7.48E-03	0.0221
SMCR8	-1.17	2.86E-03	0.0118
ABHD1	-1.17	2.19E-02	0.0468
NEIL1	-1.17	1.76E-02	0.0400
ENTPD4	-1.17	1.58E-03	0.0083
ENST000003159300	-1.17	1.89E-03	0.0092
L3MBTL4	-1.17	6.77E-03	0.0206
RFX3	-1.17	7.18E-03	0.0214
FBXO15	-1.17	2.18E-03	0.0100
EIF4E3	-1.17	9.66E-04	0.0063
ANKRD36B	-1.17	1.04E-03	0.0066
CACNG8	-1.17	1.13E-02	0.0292
ZEB1	-1.17	1.02E-03	0.0065
RPS3P2	-1.17	1.50E-02	0.0358
SMIM14	-1.17	1.80E-03	0.0090
AL137803.1	-1.17	5.85E-03	0.0187
ENST000002925300	-1.17	1.82E-02	0.0411
GAPLINC	-1.17	4.88E-03	0.0166
TSKU	-1.17	8.08E-03	0.0232
ZSWIM5	-1.18	6.32E-03	0.0196
EPB41	-1.18	1.04E-03	0.0066
AC244216.3	-1.18	1.56E-02	0.0366
AC245056.4	-1.18	1.56E-02	0.0366
AC245056.5	-1.18	1.56E-02	0.0366
AC079341.1	-1.18	4.72E-03	0.0163
GADL1	-1.18	3.07E-03	0.0123
TCTA	-1.18	2.64E-03	0.0113
ZBTB40	-1.18	1.26E-03	0.0073
AL662907.3	-1.18	1.16E-02	0.0297
TMEM116	-1.18	6.08E-03	0.0192
ZC3H6	-1.18	6.46E-03	0.0199
RN7SL405P	-1.18	1.99E-02	0.0437
C2	-1.18	6.87E-03	0.0208
ING4	-1.18	4.98E-03	0.0168
NKAIN3-IT1	-1.18	1.51E-02	0.0358

RIN3	-1.18	2.08E-02	0.0452
APPL2	-1.18	9.53E-03	0.0259
PLS3-AS1	-1.18	1.61E-02	0.0376
RUNDC3A-AS1	-1.18	8.85E-03	0.0246
CBFA2T2	-1.18	4.23E-03	0.0152
AC024451.4	-1.18	6.87E-03	0.0208
SLC12A6	-1.18	8.24E-04	0.0058
AC013652.1	-1.18	3.13E-03	0.0125
MIR193BHG	-1.18	2.02E-02	0.0442
AC138904.3	-1.18	9.68E-03	0.0262
CLLU1	-1.19	1.06E-02	0.0280
CASP1	-1.19	9.32E-04	0.0062
MIR29B2CHG	-1.19	6.17E-03	0.0194
AC006254.1	-1.19	1.42E-02	0.0343
RNU4-2	-1.19	3.36E-03	0.0130
KMT2C	-1.19	4.42E-03	0.0155
OR10H5	-1.19	9.49E-03	0.0259
AC009120.5	-1.19	1.01E-02	0.0269
ENST000003322200	-1.19	1.00E-02	0.0267
OR6Y1	-1.19	3.79E-03	0.0141
ENST000003284030	-1.19	6.86E-03	0.0208
EPHB6	-1.19	1.53E-02	0.0362
CNN2	-1.19	4.76E-03	0.0163
TNFRSF25	-1.19	1.04E-03	0.0066
AC010976.1	-1.19	1.54E-02	0.0364
VPS13C	-1.19	6.59E-04	0.0052
AGO3	-1.19	1.64E-03	0.0085
KREMEN1	-1.19	8.56E-03	0.0241
AC007998.4	-1.19	2.34E-03	0.0105
N4BP2L2	-1.19	1.87E-03	0.0092
AL450992.1	-1.19	5.14E-03	0.0172
WDR7	-1.19	7.56E-04	0.0055
OR5AN1	-1.19	1.08E-02	0.0283
AL133243.2	-1.19	2.12E-03	0.0099
DLC1	-1.19	7.08E-03	0.0212
AL033543.1	-1.20	2.13E-03	0.0099
GNB5	-1.20	1.26E-03	0.0073
PACS1	-1.20	4.11E-03	0.0149
ENST000003208760	-1.20	6.94E-04	0.0053
AGPAT4	-1.20	2.08E-02	0.0452
AL031772.1	-1.20	5.07E-03	0.0170
C8orf31	-1.20	6.80E-03	0.0206
PLEKHA8P1	-1.20	4.04E-03	0.0147
AC027373.1	-1.20	7.36E-03	0.0218
AL604028.2	-1.20	1.17E-02	0.0300
ZNF91	-1.20	6.48E-03	0.0200
ENST000002361921	-1.20	2.37E-03	0.0105

TRMT9B	-1.20	1.51E-02	0.0359
HELLPAR	-1.20	3.03E-03	0.0122
ARNTL	-1.20	1.10E-03	0.0068
RASSF8-AS1	-1.20	2.36E-02	0.0495
LINC00635	-1.20	5.33E-03	0.0176
AC015911.3	-1.20	1.64E-02	0.0381
ITGA6-AS1	-1.20	1.64E-02	0.0381
IGFL2	-1.20	1.71E-02	0.0392
GLIPR2	-1.20	1.51E-03	0.0081
GNRHR2	-1.20	3.17E-03	0.0126
AP002498.1	-1.20	5.26E-03	0.0174
FCHO2	-1.20	9.47E-03	0.0258
AC091132.4	-1.20	9.82E-03	0.0264
AC243807.3	-1.20	9.82E-03	0.0264
CASR	-1.20	7.81E-03	0.0227
DOCK9	-1.20	6.96E-04	0.0053
ENST000000546660	-1.20	1.21E-03	0.0072
PI4KA	-1.20	5.70E-03	0.0184
ENST000002531443	-1.20	5.74E-03	0.0185
AC008760.1	-1.21	1.44E-02	0.0348
TNFSF4	-1.21	8.76E-03	0.0245
PSMD6-AS1	-1.21	2.14E-02	0.0460
GOLGA8N	-1.21	1.94E-02	0.0429
AC104653.1	-1.21	6.63E-03	0.0203
AC008040.5	-1.21	1.35E-02	0.0331
NAP1L2	-1.21	3.20E-03	0.0126
STX17	-1.21	4.66E-03	0.0161
AC011477.1	-1.21	9.12E-03	0.0252
RDM1P5	-1.21	4.92E-03	0.0167
TAS2R31	-1.21	2.31E-02	0.0487
Z97192.2	-1.21	8.21E-03	0.0234
LRRC74B	-1.21	1.49E-02	0.0355
BAIAP2-DT	-1.21	5.94E-03	0.0189
RPS4XP5	-1.21	4.55E-03	0.0158
ZNF326	-1.21	2.54E-03	0.0110
LINS1	-1.21	1.15E-03	0.0070
AC090114.3	-1.21	6.15E-03	0.0193
IQCM	-1.21	1.12E-02	0.0289
BMPR2	-1.21	1.18E-03	0.0071
WARS2-IT1	-1.21	8.01E-03	0.0231
LINC01278	-1.21	2.85E-03	0.0118
OR8G1	-1.21	1.47E-02	0.0352
FCRL5	-1.21	2.80E-03	0.0116
AC016949.1	-1.21	3.27E-03	0.0128
DISP2	-1.21	4.57E-03	0.0159
JMY	-1.22	1.73E-03	0.0088
SLC25A45	-1.22	2.48E-03	0.0109

INAVA	-1.22	1.80E-02	0.0408
CASC19	-1.22	3.16E-03	0.0125
RIPK2	-1.22	2.11E-03	0.0098
RABGAP1L	-1.22	1.17E-03	0.0070
AC005005.4	-1.22	2.55E-03	0.0111
MECP2	-1.22	1.68E-03	0.0086
RN7SL321P	-1.22	1.18E-02	0.0301
ARF4	-1.22	5.36E-03	0.0176
USP53	-1.22	1.35E-02	0.0331
TRIM22	-1.22	3.65E-03	0.0138
IPO5P1	-1.22	9.37E-03	0.0256
DENND4C	-1.22	8.30E-04	0.0058
CD47	-1.22	1.21E-03	0.0072
LHFPL5	-1.22	8.76E-03	0.0245
CDH13	-1.22	1.40E-02	0.0340
AL080317.2	-1.22	1.68E-02	0.0387
SF3A1	-1.22	1.44E-02	0.0348
OR2H2	-1.22	4.27E-03	0.0152
SLC43A2	-1.22	1.53E-02	0.0361
AC111182.1	-1.22	1.21E-02	0.0306
ZNF708	-1.22	1.08E-03	0.0067
SPTLC3	-1.22	3.08E-03	0.0123
VPS13D	-1.22	1.22E-03	0.0072
AC022893.1	-1.22	7.98E-03	0.0230
SPSB3	-1.23	1.36E-03	0.0076
AC006946.2	-1.23	9.35E-03	0.0256
SKI	-1.23	4.46E-03	0.0156
EIF2AK3-DT	-1.23	4.46E-03	0.0156
STEAP4	-1.23	8.93E-03	0.0248
ZDHHC15	-1.23	1.75E-03	0.0089
FAM129C	-1.23	3.68E-03	0.0138
ACP7	-1.23	1.42E-02	0.0345
PIBF1	-1.23	9.82E-03	0.0264
SDCBP	-1.23	1.00E-03	0.0065
NFYC-AS1	-1.23	5.70E-03	0.0184
ENST000003147422	-1.23	1.74E-03	0.0088
CASP8	-1.23	8.82E-04	0.0060
AL049646.1	-1.23	7.12E-03	0.0213
RPS3AP12	-1.23	9.43E-03	0.0257
TMC2	-1.23	3.49E-03	0.0134
GKAP1	-1.23	5.11E-03	0.0171
LCOR	-1.23	2.17E-03	0.0100
RNF212	-1.23	1.92E-02	0.0426
PHC3	-1.23	1.49E-03	0.0081
AC007878.1	-1.23	1.51E-03	0.0081
LINC00863	-1.23	4.11E-03	0.0149
USP15	-1.23	4.87E-04	0.0044

ADI1	-1.23	1.50E-03	0.0081
BX571818.1	-1.23	6.26E-03	0.0195
LINC01359	-1.24	3.33E-03	0.0130
ASTN2	-1.24	5.26E-03	0.0174
CASK	-1.24	5.59E-03	0.0181
GABPB2	-1.24	1.25E-03	0.0073
LINC01814	-1.24	2.76E-03	0.0116
CDC14B	-1.24	4.76E-03	0.0163
AC114956.3	-1.24	2.20E-02	0.0470
F2RL3	-1.24	6.23E-03	0.0195
KANSL1	-1.24	5.58E-03	0.0181
SIDT1	-1.24	1.58E-03	0.0083
RAPGEF6	-1.24	5.74E-04	0.0048
ENST000003349760	-1.24	5.45E-03	0.0178
CELP	-1.24	1.60E-02	0.0374
KYNU	-1.24	2.32E-03	0.0104
TRAJ36	-1.24	1.60E-02	0.0374
PLEKHG5	-1.24	5.78E-03	0.0186
KCTD7	-1.24	3.29E-03	0.0129
AP000542.2	-1.24	2.65E-03	0.0113
KIFC2	-1.24	6.64E-03	0.0203
UTS2B	-1.24	1.89E-02	0.0421
TMEM191C	-1.24	1.40E-02	0.0340
TNRC6A	-1.24	1.66E-03	0.0086
SNPH	-1.24	1.36E-02	0.0333
AC079193.2	-1.25	6.41E-03	0.0198
PTPN13	-1.25	1.17E-03	0.0070
AP4S1	-1.25	7.19E-03	0.0214
AC092329.4	-1.25	1.83E-03	0.0091
DAZAP2	-1.25	6.01E-04	0.0049
AC124276.1	-1.25	1.62E-02	0.0377
AC006299.1	-1.25	1.63E-02	0.0379
ABHD12B	-1.25	8.45E-03	0.0239
DCHS2	-1.25	8.80E-03	0.0246
ATP5F1AP10	-1.25	8.74E-03	0.0245
ELF1	-1.25	6.90E-04	0.0053
AC092809.2	-1.25	2.22E-02	0.0473
FAM117B	-1.25	1.08E-02	0.0283
PIK3CG	-1.25	1.56E-03	0.0083
GPLD1	-1.25	3.45E-03	0.0132
ARHGAP28	-1.25	3.94E-03	0.0144
AKAP7	-1.25	1.85E-03	0.0091
SYT12	-1.25	9.38E-03	0.0256
ENST000003273071	-1.25	1.50E-02	0.0356
SNORD11	-1.25	1.12E-02	0.0290
ENST000002993550	-1.25	1.04E-02	0.0275
AC119044.1	-1.25	1.83E-02	0.0413

ABCA9	-1.25	3.82E-03	0.0141
SMG6	-1.25	1.10E-02	0.0287
ENST000002530542	-1.25	3.08E-03	0.0123
GALT	-1.25	1.08E-03	0.0067
AC036214.2	-1.25	2.20E-03	0.0101
DOCK2	-1.25	4.11E-03	0.0149
SH3GL1P1	-1.26	9.91E-03	0.0266
AC104118.1	-1.26	5.05E-03	0.0170
AC009318.3	-1.26	8.99E-03	0.0249
RNF125	-1.26	1.14E-02	0.0293
ZNF705E	-1.26	9.87E-03	0.0265
IREB2	-1.26	1.50E-03	0.0081
AL021368.2	-1.26	2.92E-03	0.0120
AC005832.1	-1.26	1.15E-02	0.0296
FOXP1	-1.26	1.17E-03	0.0070
ERN1	-1.26	3.30E-03	0.0129
HOMER3	-1.26	2.04E-02	0.0445
YAP1	-1.26	1.20E-02	0.0304
AL592437.2	-1.26	1.53E-02	0.0362
AL592148.3	-1.26	3.27E-03	0.0128
AC005776.2	-1.26	5.61E-03	0.0182
ENST000003013991	-1.26	1.87E-02	0.0418
LINC01002	-1.26	1.83E-03	0.0091
FAM185BP	-1.26	6.42E-03	0.0198
RNU6-1016P	-1.26	3.17E-03	0.0126
AL645568.1	-1.26	6.13E-03	0.0193
APBB1IP	-1.26	1.37E-02	0.0335
KIAA1328	-1.26	8.59E-04	0.0059
AC068620.3	-1.26	7.96E-03	0.0230
XKR9	-1.26	6.87E-03	0.0208
RELCH	-1.26	7.27E-04	0.0055
LIFR-AS1	-1.26	1.25E-02	0.0314
AC044810.9	-1.26	4.65E-03	0.0161
ZNRF2	-1.26	9.44E-04	0.0063
EMP2	-1.26	8.57E-03	0.0241
ENST000002620962	-1.26	2.86E-03	0.0118
OPRM1	-1.27	6.24E-03	0.0195
MAP3K3	-1.27	1.04E-03	0.0066
OR5H1	-1.27	1.92E-02	0.0427
MPV17L	-1.27	6.25E-03	0.0195
ENST000003082341	-1.27	4.36E-03	0.0154
LINC01562	-1.27	1.70E-03	0.0087
ZNF235	-1.27	1.53E-03	0.0082
RF00017.127	-1.27	1.30E-02	0.0323
RPL21P135	-1.27	1.30E-02	0.0323
MAP3K4	-1.27	1.89E-03	0.0093
NEDD4L	-1.27	3.55E-03	0.0135

ZBTB4	-1.27	4.42E-03	0.0155
PHF12	-1.27	2.34E-03	0.0105
OR2V2	-1.27	1.20E-02	0.0304
AC073332.1	-1.27	1.42E-02	0.0344
AL157838.1	-1.27	1.85E-02	0.0416
TPH1	-1.27	1.11E-02	0.0288
Z93930.3	-1.27	2.33E-02	0.0490
ENST000003707100	-1.27	9.04E-03	0.0250
CCDC84	-1.27	1.43E-03	0.0079
RNPC3	-1.27	1.37E-03	0.0077
Z95114.1	-1.27	2.02E-03	0.0096
SBK1	-1.27	1.78E-02	0.0404
ENST000002160191	-1.27	2.88E-03	0.0118
AC124804.1	-1.27	2.15E-02	0.0463
PARP4	-1.27	5.11E-04	0.0045
CD69	-1.28	8.30E-04	0.0058
ABCC2	-1.28	3.43E-03	0.0132
IQCF3	-1.28	6.01E-03	0.0190
ATG16L1	-1.28	1.46E-03	0.0080
ENST000002778170	-1.28	1.47E-02	0.0352
CYLD	-1.28	8.14E-04	0.0057
AC018809.2	-1.28	6.20E-03	0.0194
AC037459.3	-1.28	2.34E-02	0.0491
RDH16	-1.28	4.80E-03	0.0164
BORCS7	-1.28	9.33E-03	0.0256
AC011379.1	-1.28	1.10E-02	0.0286
VPS26A	-1.28	8.76E-03	0.0245
NUDT16P1	-1.28	1.12E-02	0.0291
LEKR1	-1.28	4.42E-03	0.0155
AC078906.1	-1.28	1.88E-02	0.0420
LINC02473	-1.28	6.83E-03	0.0207
AP000911.1	-1.28	1.73E-02	0.0395
ZMYND11	-1.28	6.10E-03	0.0192
SGSM2	-1.28	1.33E-03	0.0075
AC008115.4	-1.28	4.76E-03	0.0163
ENST000003300221	-1.28	1.35E-02	0.0331
LINC00342	-1.28	7.51E-04	0.0055
AC012368.2	-1.28	1.08E-02	0.0283
PTEN	-1.28	6.17E-04	0.0050
AC092431.2	-1.28	1.36E-02	0.0333
BRWD1	-1.29	6.37E-04	0.0051
CEP85L	-1.29	5.75E-04	0.0048
NUTM2B-AS1	-1.29	1.80E-03	0.0090
FP325330.3	-1.29	6.85E-03	0.0208
ANKRD44-IT1	-1.29	5.19E-03	0.0173
CDKL5	-1.29	3.58E-03	0.0136
SNORD11B	-1.29	3.08E-03	0.0123

TTC14	-1.29	1.43E-03	0.0079
UNC80	-1.29	2.18E-03	0.0100
GRAP	-1.29	1.07E-03	0.0067
CATSPERG	-1.29	6.56E-03	0.0201
SMAD3	-1.29	9.54E-03	0.0260
PCAT14	-1.29	2.97E-03	0.0121
AMOT	-1.29	3.61E-03	0.0137
LINC01016	-1.29	7.52E-03	0.0221
RNF149	-1.29	1.19E-03	0.0071
C8orf34-AS1	-1.29	3.76E-03	0.0140
LDHAL6CP	-1.29	8.51E-03	0.0240
LINC00882	-1.29	7.20E-03	0.0214
PARP12	-1.29	3.93E-03	0.0144
LINC01684	-1.29	1.36E-03	0.0076
SCD5	-1.29	2.57E-03	0.0111
ALS2CR12	-1.29	1.12E-02	0.0290
AC073073.2	-1.29	2.98E-03	0.0121
AC003681.1	-1.29	1.19E-02	0.0304
AP001486.2	-1.29	1.20E-03	0.0071
TBCC	-1.29	7.36E-04	0.0055
ZNF528	-1.29	9.65E-04	0.0063
PRAM1	-1.29	1.77E-02	0.0403
CACNB4	-1.29	2.04E-02	0.0445
TMEM232	-1.30	1.62E-02	0.0377
CHIC2	-1.30	2.56E-03	0.0111
AC040169.3	-1.30	6.04E-03	0.0191
CNNM2	-1.30	2.92E-03	0.0119
CTSS	-1.30	5.34E-04	0.0046
CD36	-1.30	1.51E-02	0.0359
CYSLTR2	-1.30	6.50E-03	0.0200
ZNF662	-1.30	3.49E-03	0.0133
AL136379.1	-1.30	1.23E-02	0.0311
BORCS5	-1.30	2.83E-03	0.0117
VDAC1P8	-1.30	4.26E-03	0.0152
ENST000003100150	-1.30	6.87E-04	0.0053
CTSK	-1.30	1.45E-02	0.0349
HNRNPA1P54	-1.30	2.16E-03	0.0099
AL133342.1	-1.30	1.18E-02	0.0301
CECR7	-1.30	2.02E-02	0.0442
TPRG1L	-1.30	4.77E-03	0.0164
DGKA	-1.30	1.68E-03	0.0086
AC127070.2	-1.30	3.90E-03	0.0143
CCDC30	-1.30	4.41E-03	0.0155
AL133387.2	-1.30	1.50E-02	0.0357
OR2M3	-1.30	3.34E-03	0.0130
ENST000003290821	-1.30	4.85E-03	0.0165
CD6	-1.30	7.79E-04	0.0056

SLC1A6	-1.30	3.54E-03	0.0135
AL031595.2	-1.31	7.82E-03	0.0227
ENST00000641104	-1.31	1.96E-02	0.0433
LINC00667	-1.31	7.66E-03	0.0224
HERC1	-1.31	7.97E-04	0.0057
UBE2Q2P1	-1.31	1.26E-02	0.0316
VWA5B1	-1.31	1.56E-02	0.0366
AC008870.2	-1.31	1.09E-02	0.0285
ENST000002532701	-1.31	1.16E-03	0.0070
AL137025.1	-1.31	1.70E-02	0.0391
AC016907.2	-1.31	9.10E-03	0.0252
WASHC3	-1.31	9.87E-04	0.0064
AC090181.2	-1.31	1.29E-02	0.0322
SELPLG	-1.31	5.13E-03	0.0171
AC011475.1	-1.31	1.55E-02	0.0366
LINC00547	-1.31	1.25E-02	0.0314
AL021707.2	-1.31	2.22E-02	0.0473
GRAP2	-1.31	7.82E-04	0.0056
ENST000002982742	-1.31	5.50E-03	0.0179
IL10RA	-1.31	1.23E-03	0.0073
LINC00346	-1.31	1.33E-03	0.0075
CRTC3	-1.31	8.60E-03	0.0242
ENST000003177750	-1.31	9.48E-03	0.0258
AL627309.1	-1.31	5.98E-03	0.0190
AC121320.1	-1.31	1.65E-02	0.0383
AC024075.2	-1.31	3.20E-03	0.0126
AP001178.3	-1.31	6.39E-03	0.0198
ZNF891	-1.31	1.89E-03	0.0092
ADAM32	-1.31	3.01E-03	0.0122
TNRC6B	-1.31	1.42E-03	0.0079
ICOSLG	-1.32	1.03E-02	0.0273
SLC35F6	-1.32	2.80E-03	0.0116
NSMCE3	-1.32	8.29E-04	0.0058
AC244669.1	-1.32	5.86E-03	0.0187
AC243922.3	-1.32	1.12E-03	0.0068
KLHL32	-1.32	3.37E-03	0.0131
HLA-DQA1	-1.32	9.14E-04	0.0061
RNA5SP281	-1.32	1.86E-02	0.0417
ZNF556	-1.32	2.76E-03	0.0116
RALGAPA1	-1.32	5.22E-04	0.0045
SEC14L2	-1.32	1.25E-02	0.0313
AL022344.1	-1.32	1.14E-02	0.0293
RPS29P21	-1.32	2.28E-02	0.0482
BTN3A3	-1.32	7.44E-04	0.0055
TRIM73	-1.32	8.40E-03	0.0238
CRY2	-1.32	1.76E-03	0.0089
AC126768.3	-1.32	5.46E-03	0.0179

RN7SL615P	-1.32	1.76E-02	0.0400
AC007038.1	-1.32	6.59E-03	0.0202
DHRS12	-1.32	2.66E-03	0.0113
AC006141.1	-1.32	1.60E-03	0.0084
AC007390.2	-1.32	2.12E-03	0.0098
AC244517.1	-1.32	2.24E-02	0.0476
ENST000002804670	-1.32	2.97E-03	0.0121
ZNF517	-1.32	1.09E-02	0.0285
PLIN5	-1.32	7.89E-03	0.0228
AC133473.1	-1.32	2.08E-02	0.0451
MT-RNR2	-1.32	1.28E-02	0.0319
SCHIP1	-1.32	2.71E-03	0.0115
PRDM2	-1.32	5.22E-04	0.0045
AC087752.4	-1.33	2.00E-02	0.0440
CYP4A22-AS1	-1.33	1.53E-03	0.0082
LINC01091	-1.33	1.09E-02	0.0284
GAB3	-1.33	1.03E-03	0.0066
PNPLA5	-1.33	1.68E-02	0.0387
GPR155	-1.33	5.07E-04	0.0045
LINC00670	-1.33	3.54E-03	0.0135
ZNF471	-1.33	1.22E-03	0.0072
ENST000002503771	-1.33	1.13E-03	0.0069
HEXD-IT1	-1.33	4.40E-03	0.0155
ENST000003352510	-1.33	1.55E-03	0.0083
C1orf158	-1.33	1.57E-02	0.0370
KLRD1	-1.33	3.43E-03	0.0132
AC096644.4	-1.33	7.68E-03	0.0224
AC011476.3	-1.33	1.07E-02	0.0282
ITFG2	-1.33	7.45E-04	0.0055
AC092279.1	-1.33	2.31E-03	0.0104
AC009318.2	-1.33	5.36E-03	0.0176
AC009961.1	-1.33	1.88E-03	0.0092
OLMALINC	-1.33	1.01E-03	0.0065
AC087894.1	-1.33	1.59E-03	0.0084
CDC42SE1	-1.33	7.80E-04	0.0056
MRNIP	-1.33	1.40E-03	0.0078
KCNE1	-1.33	1.53E-02	0.0361
AL354892.3	-1.33	1.25E-02	0.0313
AL354892.6	-1.33	1.25E-02	0.0313
ACSM2B	-1.33	4.18E-03	0.0150
RF00017.100	-1.33	2.24E-02	0.0476
OR10H1	-1.33	1.58E-02	0.0370
ZNF548	-1.33	2.74E-03	0.0115
AL031430.1	-1.33	2.24E-02	0.0476
NSUN5P1	-1.33	2.43E-03	0.0107
NEGR1	-1.33	3.14E-03	0.0125
C5orf64	-1.33	5.60E-03	0.0182

LINC02447	-1.33	8.59E-03	0.0242
MEF2D	-1.33	1.91E-02	0.0426
AL136985.3	-1.33	1.19E-02	0.0303
HERC3	-1.33	1.60E-03	0.0084
AL355816.2	-1.34	5.31E-03	0.0175
VIM-AS1	-1.34	1.50E-03	0.0081
AC007881.3	-1.34	1.88E-02	0.0420
CD44	-1.34	2.28E-03	0.0103
TRIM3	-1.34	3.96E-03	0.0145
AL352979.2	-1.34	6.93E-03	0.0209
ANKAR	-1.34	1.24E-03	0.0073
AC244216.4	-1.34	2.00E-02	0.0439
AC244216.5	-1.34	2.00E-02	0.0439
AC245034.1	-1.34	2.00E-02	0.0439
AC097461.1	-1.34	2.13E-03	0.0099
VPS13B	-1.34	3.55E-04	0.0038
KCNJ5	-1.34	5.52E-03	0.0180
PCDHB16	-1.34	1.46E-02	0.0351
OR7D2	-1.34	7.49E-03	0.0221
WDR81	-1.34	1.06E-03	0.0066
SNORD10	-1.34	3.76E-03	0.0140
WISP2	-1.34	1.65E-02	0.0382
SENP7	-1.34	5.97E-04	0.0049
ZBTB37	-1.34	1.00E-03	0.0065
SESN1	-1.34	8.05E-04	0.0057
GALR1	-1.34	6.96E-03	0.0210
TMOD2	-1.34	2.60E-03	0.0112
HRAT17	-1.34	3.31E-03	0.0129
SCN1A	-1.34	9.74E-03	0.0263
BCL2L11	-1.34	1.99E-02	0.0438
HS3ST3B1	-1.34	1.76E-03	0.0089
AC135050.6	-1.34	1.87E-03	0.0092
AL122035.2	-1.35	3.39E-03	0.0131
SYTL4	-1.35	8.03E-03	0.0231
AC103957.1	-1.35	3.06E-03	0.0123
ASAP3	-1.35	1.15E-02	0.0295
C8orf49	-1.35	7.06E-03	0.0212
TSPAN15	-1.35	1.31E-02	0.0324
EFCAB10	-1.35	1.13E-02	0.0293
GLCCI1	-1.35	2.88E-03	0.0118
ENST000003400960	-1.35	1.92E-03	0.0093
AC239809.3	-1.35	6.63E-03	0.0203
AL596325.2	-1.35	1.42E-02	0.0344
AC010148.1	-1.35	4.30E-03	0.0153
AL138820.1	-1.35	2.12E-03	0.0099
ZNF211	-1.35	1.54E-03	0.0082
CHP2	-1.35	2.03E-03	0.0096

SSH2	-1.35	3.30E-03	0.0129
ENST000003361281	-1.35	2.32E-03	0.0104
SNORA7B	-1.36	1.02E-02	0.0271
TMEM45A	-1.36	3.98E-03	0.0145
AC093520.1	-1.36	1.08E-02	0.0284
OR56A1	-1.36	5.89E-03	0.0188
GPR52	-1.36	1.24E-02	0.0311
AL513548.3	-1.36	1.63E-02	0.0378
RN7SL582P	-1.36	1.63E-02	0.0378
ABHD13	-1.36	5.71E-04	0.0048
C12orf50	-1.36	3.34E-03	0.0130
MAP3K5	-1.36	4.75E-04	0.0044
ENST000002694450	-1.36	1.78E-03	0.0089
FXYD5	-1.36	3.74E-03	0.0140
SPINK9	-1.36	3.35E-03	0.0130
NPIPB9	-1.36	2.38E-03	0.0106
AC011978.2	-1.36	1.05E-02	0.0277
FAM117A	-1.36	1.48E-03	0.0080
SNAI3	-1.36	4.19E-03	0.0150
CORO7	-1.36	1.20E-03	0.0071
TSPYL2	-1.36	4.04E-04	0.0040
CBX3P2	-1.36	1.93E-03	0.0094
AL035555.1	-1.36	3.88E-03	0.0143
OR10K1	-1.36	4.38E-03	0.0155
CFAP97D1	-1.36	5.93E-03	0.0189
RNU6-97P	-1.36	2.24E-02	0.0476
AC007099.1	-1.37	1.30E-02	0.0323
POU6F1	-1.37	9.23E-03	0.0254
KIAA2026	-1.37	3.90E-04	0.0040
OR7C1	-1.37	2.02E-03	0.0096
AL354718.1	-1.37	1.19E-02	0.0303
MIPOL1	-1.37	6.68E-04	0.0052
AC107918.4	-1.37	9.13E-03	0.0252
NBPF15	-1.37	4.92E-04	0.0044
ADCY2	-1.37	6.14E-03	0.0193
AC098936.1	-1.37	1.51E-02	0.0359
COLCA1	-1.37	1.84E-02	0.0414
C2orf15	-1.37	6.19E-03	0.0194
LINC01843	-1.37	1.81E-02	0.0410
LRP1	-1.37	2.37E-02	0.0496
TRBV12-4	-1.37	1.99E-03	0.0095
DNASE1L3	-1.37	1.90E-02	0.0423
ENST000003193630	-1.37	9.85E-03	0.0265
XPC	-1.37	7.36E-03	0.0218
TXLNGY	-1.37	3.35E-04	0.0037
CERS6-AS1	-1.37	1.78E-02	0.0404
ENST000003022190	-1.37	1.12E-02	0.0290

PITPNC1	-1.37	5.39E-04	0.0046
MCF2L2	-1.37	1.22E-03	0.0072
ATAD2B	-1.37	3.85E-04	0.0039
SIPA1L3	-1.37	2.05E-02	0.0446
AC139718.2	-1.38	1.83E-02	0.0413
ENST000003234921	-1.38	7.05E-04	0.0054
AC019117.1	-1.38	7.87E-03	0.0228
SELL	-1.38	6.01E-03	0.0190
NEK11	-1.38	2.80E-03	0.0116
ZIM2-AS1	-1.38	2.24E-02	0.0476
GUSBP11	-1.38	5.16E-03	0.0172
AC008026.3	-1.38	7.33E-03	0.0217
AC093426.1	-1.38	1.44E-02	0.0347
NBPF1	-1.38	7.98E-04	0.0057
SNORA53	-1.38	6.49E-04	0.0051
PLEKHG3	-1.38	2.88E-03	0.0118
AL356317.1	-1.38	3.64E-03	0.0137
AC010913.1	-1.38	4.21E-03	0.0151
LINC02551	-1.38	4.83E-03	0.0165
RF00017.8	-1.38	7.89E-03	0.0228
OR7A17	-1.38	9.63E-03	0.0261
AC009502.1	-1.38	3.10E-03	0.0124
AC079610.1	-1.38	2.71E-03	0.0114
AL450345.1	-1.38	8.25E-03	0.0235
TADA2B	-1.38	9.13E-04	0.0061
AC126763.1	-1.38	2.16E-02	0.0464
ENST000002562550	-1.38	1.17E-03	0.0070
ENST000002026771	-1.38	7.55E-03	0.0222
AJ009632.2	-1.38	3.27E-03	0.0128
SFTPD-AS1	-1.38	7.50E-03	0.0221
ENST000003219451	-1.38	2.16E-03	0.0099
PRKY	-1.38	3.42E-03	0.0132
AC008609.1	-1.38	1.25E-02	0.0313
AL008638.6	-1.39	6.21E-03	0.0194
AP000542.3	-1.39	4.79E-03	0.0164
MIR573	-1.39	4.17E-03	0.0150
ENST000003369760	-1.39	7.17E-04	0.0054
ENST000003183080	-1.39	1.16E-02	0.0298
IKBKE	-1.39	2.20E-03	0.0101
TTC39C	-1.39	3.64E-03	0.0137
AL122125.1	-1.39	1.35E-02	0.0332
LRP2BP	-1.39	3.31E-03	0.0129
OR5H14	-1.39	6.25E-03	0.0195
CCDC80	-1.39	3.50E-03	0.0134
FLJ31104	-1.39	6.41E-03	0.0198
CREBBP	-1.39	2.27E-03	0.0103
AC245100.4	-1.39	7.04E-03	0.0211

ENST00000550837	-1.39	4.51E-03	0.0157
PCMTD1	-1.39	6.05E-04	0.0049
RF00139.3	-1.39	5.67E-03	0.0183
SNX18	-1.39	2.14E-03	0.0099
LRRC8C-DT	-1.39	2.67E-03	0.0113
ACPP	-1.39	7.83E-03	0.0227
PVRIG	-1.39	4.56E-03	0.0159
SUN2	-1.39	3.80E-04	0.0039
CYP3A5	-1.40	5.10E-03	0.0171
AC026124.2	-1.40	1.98E-03	0.0095
AC027097.2	-1.40	2.36E-03	0.0105
CFAP221	-1.40	1.37E-02	0.0336
HDAC4	-1.40	4.88E-03	0.0166
BCL9L	-1.40	1.70E-02	0.0391
ENST000002482110	-1.40	2.88E-03	0.0118
TEX12	-1.40	2.17E-02	0.0466
RNF166	-1.40	9.35E-04	0.0062
PAN3	-1.40	7.41E-04	0.0055
AL121944.1	-1.40	1.28E-02	0.0319
AP003119.3	-1.40	1.52E-02	0.0361
AC084824.3	-1.40	2.39E-02	0.0499
ENST000003006824	-1.40	1.60E-03	0.0084
TRBV11-2	-1.40	1.93E-02	0.0428
LINC01762	-1.40	6.67E-03	0.0204
NPTXR	-1.40	2.24E-02	0.0476
LINC01933	-1.40	7.02E-03	0.0211
AC021660.3	-1.40	1.45E-02	0.0349
CHRM3-AS2	-1.40	1.22E-02	0.0308
SLFN5	-1.40	3.11E-03	0.0124
CHRM5	-1.40	1.31E-02	0.0324
AC010168.2	-1.40	6.51E-04	0.0051
AC025539.1	-1.40	9.75E-04	0.0064
ENST000003412490	-1.40	2.11E-02	0.0456
AC007686.3	-1.40	6.56E-03	0.0201
LINC01473	-1.40	4.69E-03	0.0162
AL132657.1	-1.40	2.22E-02	0.0473
CYP46A1	-1.40	7.97E-04	0.0057
ENST000003898570	-1.41	5.57E-04	0.0047
AC020978.7	-1.41	3.80E-03	0.0141
SH3BP5	-1.41	1.02E-02	0.0272
C16orf54	-1.41	1.28E-03	0.0074
ENST000002925791	-1.41	8.31E-04	0.0058
CD44-AS1	-1.41	1.34E-02	0.0329
LIX1L-AS1	-1.41	8.97E-03	0.0249
DDX5	-1.41	1.39E-03	0.0078
LINC00511	-1.41	2.69E-03	0.0114
TRBV14	-1.41	4.82E-03	0.0165

AC130895.1	-1.41	1.27E-02	0.0317
AC108108.1	-1.41	2.02E-03	0.0096
PLEKHM1	-1.41	4.36E-03	0.0154
LINC02334	-1.41	1.58E-03	0.0083
LINC00276	-1.41	4.57E-03	0.0159
Z98885.3	-1.41	1.43E-02	0.0345
ENST000003126350	-1.41	2.35E-03	0.0105
MGAT4A	-1.41	2.46E-04	0.0032
TMC6	-1.41	4.20E-04	0.0041
OR5A1	-1.41	1.88E-03	0.0092
LMBR1L	-1.41	3.94E-03	0.0144
LINC00672	-1.41	3.82E-03	0.0141
RANBP17	-1.41	6.33E-03	0.0196
SDHAP3	-1.41	1.83E-02	0.0413
ACSS1	-1.41	8.61E-04	0.0059
SNORD94	-1.41	1.88E-02	0.0419
WWP1	-1.41	5.39E-04	0.0046
TMEM105	-1.42	2.14E-02	0.0460
AP000347.2	-1.42	1.53E-03	0.0082
CCDC17	-1.42	1.30E-02	0.0323
FAM228B	-1.42	5.95E-04	0.0049
CFAP206	-1.42	1.76E-02	0.0401
ABCA10	-1.42	1.98E-03	0.0095
ANO9	-1.42	5.76E-04	0.0048
ZNF252P-AS1	-1.42	1.58E-02	0.0371
HEXD	-1.42	7.80E-03	0.0227
ZC3H15	-1.42	6.39E-04	0.0051
TSPAN14	-1.42	8.62E-03	0.0242
LINC00506	-1.42	2.83E-03	0.0117
ENST00000623130	-1.42	9.78E-04	0.0064
PDE6A	-1.42	6.03E-03	0.0191
AC025187.1	-1.42	1.46E-02	0.0351
TAGAP	-1.42	1.74E-03	0.0088
SGTB	-1.42	4.96E-04	0.0044
AC108718.1	-1.42	9.88E-03	0.0265
SVIL	-1.42	6.44E-04	0.0051
MPPED2	-1.42	9.71E-03	0.0262
ZNF844	-1.42	3.22E-03	0.0127
AC015849.5	-1.42	8.03E-03	0.0231
AC015849.9	-1.42	8.03E-03	0.0231
RAD21-AS1	-1.42	1.71E-03	0.0087
AL137782.1	-1.42	5.26E-03	0.0174
AC005670.2	-1.42	1.76E-02	0.0400
AC125613.1	-1.42	2.34E-02	0.0492
PTP4A1	-1.42	2.11E-03	0.0098
AC007342.4	-1.42	4.67E-03	0.0161
KIAA1109	-1.42	3.94E-04	0.0040

ULK4	-1.43	1.46E-03	0.0080
ZBTB16	-1.43	1.50E-02	0.0356
LINC02615	-1.43	7.66E-03	0.0224
OR2F1	-1.43	5.75E-03	0.0185
SNORA75	-1.43	3.64E-03	0.0137
TRIM2	-1.43	6.78E-03	0.0206
PARD3B	-1.43	1.16E-02	0.0297
SNORA2B	-1.43	4.36E-03	0.0154
AL133372.2	-1.43	2.98E-03	0.0121
AC021683.3	-1.43	3.17E-03	0.0126
FAM124B	-1.43	4.07E-03	0.0148
ATCAY	-1.43	6.50E-03	0.0200
IKZF2	-1.43	2.96E-03	0.0121
BVES	-1.43	3.84E-03	0.0142
AC078880.1	-1.43	7.45E-03	0.0220
CD302	-1.43	4.10E-03	0.0148
AC008870.1	-1.43	5.33E-03	0.0176
ARL15	-1.43	1.65E-03	0.0085
RN7SL258P	-1.43	1.95E-02	0.0431
FP236383.1	-1.43	9.87E-03	0.0265
FP671120.1	-1.43	9.87E-03	0.0265
RETREG1	-1.43	7.14E-04	0.0054
TFPI	-1.43	3.18E-03	0.0126
AL162615.1	-1.43	3.77E-03	0.0140
ZBTB10	-1.43	5.66E-04	0.0048
OR5AS1	-1.43	5.23E-04	0.0045
AL008729.1	-1.43	4.27E-03	0.0152
SCRG1	-1.43	2.56E-03	0.0111
RICTOR	-1.43	5.79E-04	0.0048
RNF157	-1.43	1.98E-03	0.0095
OR10G4	-1.43	2.11E-02	0.0455
OR7E122P	-1.43	1.14E-03	0.0069
OR2AT4	-1.44	1.19E-03	0.0071
TEX35	-1.44	1.80E-03	0.0090
SPPL3	-1.44	5.43E-03	0.0178
AC000120.1	-1.44	1.66E-03	0.0086
AC078795.2	-1.44	9.83E-03	0.0265
TRBV10-3	-1.44	2.25E-03	0.0102
SLC39A12-AS1	-1.44	2.98E-03	0.0121
AC103760.1	-1.44	3.33E-03	0.0130
LINC01297	-1.44	5.25E-03	0.0174
MAPK10	-1.44	7.58E-04	0.0055
ADH4	-1.44	4.28E-03	0.0153
FRRS1L	-1.44	1.01E-02	0.0269
ENST000003221531	-1.44	2.08E-03	0.0098
BRD1	-1.44	3.88E-04	0.0040
ZNF44	-1.44	1.07E-03	0.0067

HFE	-1.44	4.87E-03	0.0166
TLR10	-1.44	9.60E-03	0.0261
AL133284.1	-1.44	2.11E-02	0.0455
SRSF5	-1.44	2.64E-04	0.0034
AC092802.1	-1.44	2.19E-03	0.0100
AC074032.1	-1.44	6.47E-03	0.0200
OR4D9	-1.44	3.02E-03	0.0122
AC092611.1	-1.44	1.31E-02	0.0324
YWHABP2	-1.44	1.31E-02	0.0324
CBR3-AS1	-1.45	4.09E-03	0.0148
AC011405.1	-1.45	8.68E-03	0.0243
ENST000002541821	-1.45	2.00E-02	0.0438
GSTM2	-1.45	6.17E-03	0.0194
SNORA31	-1.45	1.88E-02	0.0420
LINC00507	-1.45	9.38E-03	0.0257
GNG12-AS1	-1.45	2.01E-02	0.0440
AC109460.2	-1.45	1.49E-02	0.0355
AC020978.6	-1.45	2.32E-03	0.0104
PLG	-1.45	1.50E-02	0.0357
NCOA5	-1.45	1.55E-02	0.0365
ENST000002584840	-1.45	3.42E-04	0.0038
AC245060.6	-1.45	1.80E-02	0.0408
COL8A1	-1.45	1.48E-02	0.0355
EPB41L4A	-1.45	6.41E-03	0.0198
AC012349.1	-1.45	2.65E-03	0.0113
AC116535.1	-1.45	2.07E-02	0.0449
ADPRM	-1.45	2.46E-03	0.0108
SRSF8	-1.45	4.63E-03	0.0160
ENST000002541081	-1.45	4.56E-03	0.0159
EZH1	-1.45	7.71E-04	0.0056
AC046158.2	-1.45	2.07E-02	0.0450
RBFOX3	-1.45	1.76E-02	0.0400
AP000350.10	-1.45	2.17E-03	0.0100
AP000350.7	-1.45	2.17E-03	0.0100
DNAH2	-1.45	2.68E-03	0.0114
AC008669.1	-1.45	4.70E-03	0.0162
AP005131.1	-1.45	3.60E-03	0.0136
OSTM1	-1.45	2.49E-03	0.0109
AC108693.1	-1.45	1.89E-03	0.0093
C13orf46	-1.45	1.26E-02	0.0315
AP003175.1	-1.45	1.83E-03	0.0091
AC027338.1	-1.45	2.06E-02	0.0448
OR52A1	-1.45	3.84E-03	0.0142
LINC01358	-1.46	4.74E-03	0.0163
SNORD14A	-1.46	3.30E-03	0.0129
ACYP2	-1.46	1.58E-03	0.0083
AC244197.3	-1.46	2.97E-03	0.0121

ZXDB	-1.46	1.13E-03	0.0069
AC010186.3	-1.46	1.92E-03	0.0094
NBPF25P	-1.46	8.15E-03	0.0233
SNN	-1.46	1.02E-03	0.0065
AL157938.3	-1.46	1.36E-02	0.0333
COLEC11	-1.46	8.85E-03	0.0246
KCNIP4-IT1	-1.46	5.65E-03	0.0183
LEF1	-1.46	7.95E-04	0.0057
AC010485.1	-1.46	1.09E-02	0.0284
FTX	-1.46	8.56E-04	0.0059
AC010186.2	-1.46	1.14E-03	0.0069
AC005089.1	-1.46	1.16E-02	0.0298
C1orf54	-1.46	1.37E-02	0.0335
SH3YL1	-1.46	2.79E-03	0.0116
CNOT6L	-1.46	4.26E-04	0.0041
N4BP2L2-IT2	-1.46	7.52E-03	0.0221
FRK	-1.46	2.08E-03	0.0097
LRRC37A3	-1.46	8.71E-03	0.0244
GABPB1-IT1	-1.47	4.77E-04	0.0044
INPP5D	-1.47	2.34E-03	0.0105
ART4	-1.47	2.23E-02	0.0475
ABCA7	-1.47	2.04E-02	0.0445
TRAV8-3	-1.47	2.11E-03	0.0098
FAM160A1	-1.47	2.32E-02	0.0488
HNMT	-1.47	1.73E-02	0.0396
GVINP1	-1.47	3.26E-04	0.0037
UPF3AP3	-1.47	1.34E-02	0.0329
RASEF	-1.47	7.59E-03	0.0222
C8orf34	-1.47	1.33E-03	0.0075
UTY	-1.47	7.01E-04	0.0054
GPR75	-1.47	6.70E-03	0.0204
AC087164.1	-1.47	3.04E-03	0.0122
SUGCT	-1.47	1.56E-02	0.0367
TEX41	-1.47	2.16E-03	0.0100
ENST000003392821	-1.47	7.68E-04	0.0056
KLK10	-1.47	1.72E-02	0.0394
FAM180B	-1.47	2.24E-02	0.0476
BLCAP	-1.47	1.04E-03	0.0066
NSFP1	-1.47	9.04E-04	0.0061
MTSS1	-1.47	2.15E-03	0.0099
ABCA6	-1.47	5.77E-03	0.0185
ENST000003585110	-1.48	2.34E-02	0.0490
RNF103	-1.48	1.20E-03	0.0071
OVGP1	-1.48	1.33E-03	0.0075
AL353708.3	-1.48	6.01E-03	0.0190
AC096711.2	-1.48	1.89E-03	0.0093
CCNL1	-1.48	1.60E-04	0.0027

AC010329.1	-1.48	1.14E-02	0.0294
AC010329.4	-1.48	1.14E-02	0.0294
SNORA28	-1.48	1.24E-03	0.0073
RSRP1	-1.48	3.80E-04	0.0039
AC136475.1	-1.48	1.11E-02	0.0289
CASP14	-1.48	7.81E-03	0.0227
ADCY4	-1.48	8.99E-03	0.0249
FBXO10	-1.48	7.10E-03	0.0213
POT1-AS1	-1.48	1.43E-03	0.0079
ENST000002825162	-1.48	1.51E-02	0.0359
EFCAB5	-1.48	1.11E-02	0.0288
AC092612.1	-1.48	4.22E-03	0.0151
NKAPP1	-1.48	2.18E-03	0.0100
AF165147.1	-1.48	2.71E-03	0.0115
UBE4B	-1.48	1.07E-02	0.0280
ENST000003296231	-1.49	3.85E-03	0.0142
MACROD2	-1.49	7.27E-03	0.0216
SBF2	-1.49	1.21E-02	0.0307
FUT1	-1.49	6.46E-03	0.0199
VAMP2	-1.49	2.99E-03	0.0121
FAM13B	-1.49	4.07E-04	0.0041
Z98259.3	-1.49	1.86E-03	0.0092
AL049830.3	-1.49	1.17E-02	0.0300
BMP8B	-1.49	4.14E-03	0.0149
NANOG	-1.49	6.37E-03	0.0197
GIMAP2	-1.49	4.15E-04	0.0041
PELI2	-1.49	1.02E-03	0.0065
AC004494.1	-1.49	1.95E-02	0.0431
TM4SF19-AS1	-1.49	5.80E-03	0.0186
GRIA1	-1.49	4.33E-03	0.0154
AC012651.1	-1.49	3.79E-03	0.0141
SECISBP2	-1.49	3.78E-04	0.0039
ZNF350	-1.50	1.22E-03	0.0072
TSNAX-DISC1	-1.50	3.42E-03	0.0132
GOLGA8B	-1.50	8.12E-04	0.0057
Z94721.2	-1.50	1.42E-02	0.0343
TJP3	-1.50	7.28E-03	0.0216
MGAT4C	-1.50	3.08E-03	0.0123
CCNK	-1.50	7.23E-04	0.0054
AC107958.2	-1.50	3.59E-03	0.0136
SLC2A14	-1.50	1.21E-02	0.0307
SPATA6L	-1.50	4.41E-03	0.0155
ASIC4	-1.50	8.00E-03	0.0231
KLF3-AS1	-1.50	1.81E-03	0.0090
SFT2D3	-1.50	1.73E-03	0.0088
AL022323.3	-1.50	3.27E-03	0.0128
RHOA-IT1	-1.50	7.83E-03	0.0227

ZBTB20-AS4	-1.50	1.98E-02	0.0436
AC073052.1	-1.50	2.59E-03	0.0112
TNIK	-1.50	2.04E-03	0.0096
AP003716.1	-1.50	5.62E-03	0.0182
AC241952.1	-1.50	4.48E-03	0.0157
CPAMD8	-1.50	1.52E-02	0.0361
SNX29	-1.50	6.84E-04	0.0053
ZNF41	-1.50	8.80E-03	0.0246
MIR181A1HG	-1.50	3.31E-03	0.0129
ENST000003247652	-1.50	1.62E-03	0.0085
AC083899.2	-1.50	1.90E-02	0.0423
SRP14P3	-1.50	7.63E-03	0.0223
MALINC1	-1.50	1.62E-03	0.0085
ARMC2	-1.50	1.68E-02	0.0387
JUN	-1.51	1.43E-03	0.0079
SHISA2	-1.51	2.08E-02	0.0451
AL121652.1	-1.51	8.00E-03	0.0231
RPGR	-1.51	2.30E-03	0.0103
EPHA1-AS1	-1.51	9.34E-04	0.0062
MAGI2	-1.51	1.67E-03	0.0086
ENST000003199800	-1.51	4.05E-03	0.0147
AP001033.2	-1.51	6.88E-03	0.0208
TSBP1-AS1	-1.51	5.79E-03	0.0186
ENST000003033910	-1.51	1.16E-02	0.0298
OR10A6	-1.51	1.91E-03	0.0093
RHBDD2	-1.51	1.39E-03	0.0077
AL023495.1	-1.51	1.14E-02	0.0294
RPS16P5	-1.51	3.25E-03	0.0128
AC084782.1	-1.51	2.77E-03	0.0116
ZNF497	-1.51	2.45E-03	0.0108
TRAJ2	-1.51	5.45E-03	0.0178
SOX13	-1.51	2.31E-03	0.0104
LINC01204	-1.51	1.21E-02	0.0308
AC079921.2	-1.51	5.56E-03	0.0181
AC026316.3	-1.51	1.69E-02	0.0388
GIMAP7	-1.51	3.04E-03	0.0122
LOH12CR2	-1.51	1.05E-02	0.0276
AL139220.2	-1.51	8.46E-03	0.0239
FRAT2	-1.51	1.48E-03	0.0081
AL139397.1	-1.51	1.80E-02	0.0408
AL356966.1	-1.51	7.24E-03	0.0215
PWAR6	-1.52	6.59E-03	0.0202
HRH4	-1.52	2.20E-02	0.0470
SLC7A14	-1.52	1.42E-02	0.0344
MIRLET7BHG	-1.52	5.33E-03	0.0176
ENST000002294650	-1.52	4.26E-03	0.0152
LO000005.1	-1.52	7.55E-04	0.0055

AC093849.1	-1.52	1.56E-02	0.0368
AC079416.1	-1.52	1.78E-03	0.0089
ZNF836	-1.52	5.69E-04	0.0048
ZFAND4	-1.52	8.61E-04	0.0059
LINC01268	-1.52	2.89E-03	0.0119
LINC00504	-1.52	6.87E-04	0.0053
CELF6	-1.52	4.97E-03	0.0168
AC022540.1	-1.52	2.65E-03	0.0113
ENST000003164851	-1.52	1.03E-03	0.0066
AC015802.3	-1.52	2.31E-02	0.0487
AC103810.1	-1.52	2.27E-02	0.0480
AL139317.3	-1.52	1.04E-02	0.0276
CNR2	-1.52	2.81E-03	0.0117
ZNF233	-1.53	1.55E-02	0.0365
FABP2	-1.53	1.39E-03	0.0077
TIPARP	-1.53	1.85E-03	0.0091
ENST000002603235	-1.53	2.37E-02	0.0496
FGD3	-1.53	3.73E-04	0.0039
METTL25	-1.53	1.03E-03	0.0066
THBS1	-1.53	2.29E-02	0.0483
WDR78	-1.53	1.95E-03	0.0094
RN7SKP181	-1.53	8.81E-03	0.0246
Z83843.1	-1.53	7.24E-04	0.0054
37226	-1.53	9.39E-04	0.0063
LINC01793	-1.53	2.17E-02	0.0466
BICRAL	-1.53	4.70E-04	0.0044
LINC00894	-1.53	8.14E-03	0.0233
TMEM220	-1.53	5.78E-03	0.0186
RAB40B	-1.53	5.22E-03	0.0174
OTX2-AS1	-1.53	1.79E-02	0.0405
ENST000003072160	-1.53	3.43E-03	0.0132
STX17-AS1	-1.54	1.47E-02	0.0352
PDHB	-1.54	1.69E-03	0.0086
DENND6A	-1.54	4.74E-04	0.0044
PAX6	-1.54	3.59E-03	0.0136
MIR548AJ2	-1.54	6.07E-03	0.0192
KPNA5	-1.54	2.14E-04	0.0031
ASPA	-1.54	5.45E-03	0.0178
PPM1L	-1.54	1.58E-02	0.0370
SCARNA4	-1.54	2.70E-03	0.0114
AC145138.1	-1.54	1.02E-02	0.0272
AC078880.4	-1.54	1.67E-03	0.0086
CTSW	-1.54	7.25E-03	0.0215
ENST000003574440	-1.54	7.45E-03	0.0220
SMAGP	-1.54	3.81E-03	0.0141
CLK1	-1.54	8.85E-04	0.0060
SGMS1	-1.54	7.93E-04	0.0057

AVPI1	-1.54	3.09E-03	0.0124
ZNF879	-1.54	1.22E-03	0.0072
ABI3BP	-1.55	6.40E-03	0.0198
TENM3-AS1	-1.55	1.04E-02	0.0275
LCMT1-AS2	-1.55	2.18E-02	0.0466
RF00017.187	-1.55	9.64E-03	0.0261
TMEM212	-1.55	1.21E-03	0.0072
RCAN3	-1.55	2.22E-04	0.0031
ENST000002505593	-1.55	4.93E-04	0.0044
SLC6A16	-1.55	3.25E-03	0.0128
AC027796.5	-1.55	1.63E-02	0.0378
AL355306.2	-1.55	8.60E-03	0.0242
AC078845.1	-1.55	1.33E-02	0.0328
AL357054.1	-1.55	9.41E-03	0.0257
CTCFL	-1.55	1.21E-03	0.0072
NDRG2	-1.55	1.44E-03	0.0079
PPDPF	-1.55	2.03E-02	0.0444
NLRC3	-1.55	7.67E-04	0.0056
KAT6B	-1.55	3.61E-04	0.0038
SYNJ2BP	-1.55	1.34E-03	0.0076
NBPF19	-1.55	2.40E-04	0.0032
HEG1	-1.55	6.07E-03	0.0192
ENST000003114591	-1.55	4.20E-04	0.0041
LRGUK	-1.56	1.58E-02	0.0371
AC121333.1	-1.56	5.11E-04	0.0045
ITIH4	-1.56	1.15E-02	0.0296
AC022706.1	-1.56	1.97E-03	0.0095
ILF3-DT	-1.56	5.41E-04	0.0046
SNORA49	-1.56	2.75E-03	0.0115
AC079203.1	-1.56	1.44E-02	0.0347
OR1C1	-1.56	4.08E-03	0.0148
PLEKHD1	-1.56	1.20E-02	0.0306
AC087359.1	-1.56	5.84E-03	0.0187
AC144450.1	-1.56	4.47E-03	0.0157
KCNJ3	-1.56	1.51E-02	0.0359
AC244207.2	-1.56	2.26E-02	0.0480
ZNF513	-1.56	2.77E-03	0.0116
BEST3	-1.56	2.74E-03	0.0115
HMGN2P28	-1.56	2.22E-02	0.0473
LINC01881	-1.56	1.04E-03	0.0066
FBXL17	-1.56	4.72E-04	0.0044
TMEM30B	-1.56	1.26E-03	0.0073
BIN2	-1.56	3.35E-04	0.0037
AP002884.4	-1.56	1.39E-02	0.0338
AC010632.2	-1.56	5.40E-03	0.0177
NELL2	-1.57	1.32E-02	0.0326
LINC00674	-1.57	4.00E-03	0.0146

S1PR4	-1.57	4.89E-03	0.0166
AC007906.2	-1.57	1.17E-02	0.0299
FMO5	-1.57	4.36E-03	0.0154
ENST000003115953	-1.57	1.04E-03	0.0066
POC1B-AS1	-1.57	1.58E-02	0.0371
DPRXP3	-1.57	4.50E-03	0.0157
AC010531.3	-1.57	2.89E-03	0.0118
EBF1	-1.57	2.13E-02	0.0460
FAM172A	-1.57	2.89E-04	0.0035
ENST000002850130	-1.57	4.27E-03	0.0152
RNU6-341P	-1.57	8.54E-03	0.0241
CCDC26	-1.57	1.40E-03	0.0078
EGFLAM	-1.57	1.43E-02	0.0345
LINC00239	-1.57	1.83E-03	0.0091
TRIM62	-1.57	2.64E-03	0.0113
LRRC70	-1.57	2.63E-03	0.0113
TRDN-AS1	-1.58	1.15E-02	0.0295
NEBL	-1.58	5.70E-03	0.0184
AL138899.1	-1.58	7.75E-04	0.0056
AC068338.2	-1.58	1.54E-02	0.0364
LINC00937	-1.58	1.74E-03	0.0088
PMP2	-1.58	1.49E-02	0.0355
LINC02204	-1.58	2.18E-02	0.0467
RPL5P13	-1.58	1.77E-02	0.0402
AC073065.1	-1.58	1.65E-02	0.0382
ENST000003735040	-1.58	8.80E-04	0.0060
STON2	-1.58	1.71E-02	0.0392
AC006017.1	-1.58	6.11E-03	0.0192
NUPR1	-1.58	3.42E-03	0.0132
AC133644.2	-1.58	1.67E-03	0.0086
ENST000002496360	-1.58	2.87E-04	0.0035
AC005332.3	-1.58	2.88E-03	0.0118
AL049651.1	-1.58	2.22E-02	0.0473
SLC35E2B	-1.58	1.31E-03	0.0075
AC008937.3	-1.58	2.26E-03	0.0102
NKTR	-1.58	1.37E-03	0.0077
DAAM1	-1.58	5.93E-03	0.0189
CC2D2B	-1.58	9.27E-03	0.0254
RNF139	-1.58	2.06E-04	0.0030
CHST12	-1.58	9.17E-04	0.0062
AC126773.4	-1.58	3.27E-03	0.0128
LINC00926	-1.58	6.36E-03	0.0197
CLEC7A	-1.58	1.69E-03	0.0087
ENST000003606690	-1.58	2.09E-03	0.0098
DPP6	-1.58	1.81E-02	0.0408
LINC02362	-1.59	1.05E-02	0.0277
ARHGAP22	-1.59	1.33E-02	0.0329

AL360013.2	-1.59	2.88E-03	0.0118
MBD5	-1.59	2.82E-03	0.0117
SGMS1-AS1	-1.59	1.33E-03	0.0075
ENST000002991672	-1.59	6.81E-04	0.0053
HHAT	-1.59	1.46E-02	0.0351
AP001429.1	-1.59	5.28E-03	0.0174
PTCSC3	-1.59	3.71E-03	0.0139
FRG1JP	-1.59	2.37E-02	0.0496
TEX26-AS1	-1.59	9.03E-03	0.0250
AFAP1L2	-1.59	2.18E-02	0.0467
GPCPD1	-1.59	9.98E-04	0.0064
TTC12	-1.59	1.93E-03	0.0094
MCTP1	-1.59	1.28E-02	0.0319
AC074366.1	-1.59	5.42E-03	0.0178
IYD	-1.59	1.03E-03	0.0066
IKZF5	-1.59	6.81E-04	0.0053
WIPF1	-1.59	3.21E-04	0.0037
PRKXP1	-1.59	2.40E-03	0.0106
CREG2	-1.59	3.32E-03	0.0129
ENST000003382572	-1.60	2.35E-03	0.0105
LRRC37A17P	-1.60	1.12E-02	0.0289
ABCD2	-1.60	7.36E-03	0.0218
KIAA0825	-1.60	8.60E-04	0.0059
AC006145.1	-1.60	5.77E-03	0.0185
AC009090.1	-1.60	4.14E-03	0.0149
ENST000003263511	-1.60	1.66E-02	0.0385
MYO1F	-1.60	4.09E-04	0.0041
AP000347.1	-1.60	9.35E-04	0.0062
CCDC102B	-1.60	1.31E-03	0.0075
BCL11B	-1.60	1.50E-04	0.0026
AC095057.3	-1.60	1.01E-03	0.0065
SPEG	-1.60	4.37E-03	0.0154
AL353804.1	-1.60	3.31E-03	0.0129
CCDC83	-1.60	6.54E-03	0.0201
EMB	-1.60	4.26E-04	0.0041
AC136489.1	-1.60	1.14E-02	0.0293
RNF139-AS1	-1.60	1.03E-03	0.0066
AL590822.2	-1.60	5.11E-03	0.0171
BTN3A1	-1.60	2.70E-04	0.0034
AC092903.2	-1.60	1.41E-02	0.0341
FAM8A1	-1.60	4.81E-04	0.0044
MIR186	-1.60	5.30E-03	0.0175
TSPAN10	-1.60	2.04E-03	0.0096
AC146507.2	-1.61	9.35E-03	0.0256
ENST000003192940	-1.61	2.32E-02	0.0487
HSD17B2	-1.61	9.72E-04	0.0064
AC027455.2	-1.61	2.30E-03	0.0104

OR2A3P	-1.61	3.27E-03	0.0128
AC009754.1	-1.61	1.62E-03	0.0085
SLC16A10	-1.61	4.27E-03	0.0152
RN7SKP70	-1.61	2.80E-03	0.0116
KIZ-AS1	-1.61	1.37E-02	0.0335
AC025164.1	-1.61	2.96E-03	0.0121
PDZD7	-1.61	1.67E-03	0.0086
PPIAP41	-1.61	2.77E-03	0.0116
OR14J1	-1.61	1.57E-03	0.0083
CALCOCO1	-1.61	3.26E-04	0.0037
TRIM52	-1.61	2.31E-04	0.0032
CCDC180	-1.61	5.14E-04	0.0045
SP2	-1.61	2.21E-03	0.0101
USP3	-1.61	6.14E-04	0.0050
OR1A1	-1.61	1.37E-03	0.0077
AC003991.1	-1.61	6.13E-03	0.0193
KCNB1	-1.61	1.00E-03	0.0065
ZNF33B	-1.61	3.09E-04	0.0036
CD84	-1.61	1.91E-03	0.0093
CD52	-1.62	8.49E-04	0.0059
ENST000003190801	-1.62	2.60E-03	0.0112
AC092902.4	-1.62	4.15E-04	0.0041
OR55B1P	-1.62	6.47E-03	0.0199
PCDHGA8	-1.62	2.50E-03	0.0109
ACVR2B-AS1	-1.62	4.83E-03	0.0165
AC000367.1	-1.62	9.32E-04	0.0062
PRKCB	-1.62	6.93E-03	0.0209
NDE1P2	-1.62	2.06E-02	0.0449
GPR85	-1.62	8.85E-03	0.0246
ATF7IP2	-1.62	4.08E-04	0.0041
TPRXL	-1.62	1.69E-03	0.0087
AL138749.1	-1.62	1.94E-02	0.0429
GOLGA8R	-1.62	1.15E-02	0.0295
CFAP44	-1.62	9.66E-04	0.0063
RF00017.25	-1.62	1.54E-02	0.0364
ENST000002779002	-1.62	1.70E-04	0.0028
LINC01949	-1.63	6.85E-04	0.0053
PRSS8	-1.63	1.28E-02	0.0319
ABCC3	-1.63	4.01E-03	0.0146
ARID1B	-1.63	2.90E-04	0.0035
AC009812.3	-1.63	5.02E-03	0.0169
AC006960.3	-1.63	9.64E-03	0.0261
YPEL2	-1.63	2.35E-04	0.0032
ENST000003328840	-1.63	2.79E-03	0.0116
CHMP7	-1.63	3.20E-04	0.0037
PGGHG	-1.63	1.35E-02	0.0332
AC124069.1	-1.63	1.03E-02	0.0273

AC009113.1	-1.63	3.15E-03	0.0125
PDCD4	-1.63	2.99E-04	0.0035
FAM210B	-1.63	1.54E-03	0.0082
ZXDA	-1.63	6.27E-04	0.0050
POTEI	-1.63	1.12E-02	0.0290
VAMP3	-1.63	1.61E-03	0.0084
EHD4-AS1	-1.63	1.02E-02	0.0272
NDEL1	-1.63	7.43E-04	0.0055
AC093752.2	-1.63	1.17E-03	0.0070
ZNF480	-1.64	4.77E-03	0.0164
ENST000002635760	-1.64	8.63E-03	0.0242
HLA-DPB2	-1.64	5.19E-03	0.0173
AL683807.1	-1.64	1.60E-02	0.0374
PCNX1	-1.64	1.68E-04	0.0028
HTR7P1	-1.64	1.46E-03	0.0080
HMGA2	-1.64	1.94E-03	0.0094
AC012103.1	-1.64	1.96E-03	0.0094
AC139494.3	-1.64	4.85E-03	0.0165
AL159169.2	-1.64	8.41E-03	0.0238
VNN1	-1.64	7.02E-03	0.0211
ENST000003133500	-1.64	1.90E-02	0.0423
KALRN	-1.64	2.02E-03	0.0096
ACKR3	-1.64	7.75E-03	0.0226
OR5A2	-1.64	8.26E-03	0.0235
ST6GAL2	-1.64	2.86E-03	0.0118
SMIM10L2A	-1.64	1.91E-02	0.0426
PTPRC	-1.64	6.13E-04	0.0050
ST8SIA1	-1.64	1.14E-03	0.0069
TPRKBP2	-1.64	1.46E-02	0.0350
AC016766.1	-1.64	1.75E-02	0.0399
CDKN2B-AS1	-1.64	1.63E-03	0.0085
LINC01197	-1.64	2.32E-02	0.0487
RN7SL834P	-1.64	2.00E-03	0.0095
AP005717.1	-1.65	7.14E-03	0.0213
AC026992.2	-1.65	2.01E-02	0.0440
AC011632.1	-1.65	7.12E-03	0.0213
ENST000002954000	-1.65	1.05E-02	0.0277
SKAP1	-1.65	3.01E-04	0.0035
NDRG1	-1.65	6.45E-04	0.0051
CRBN	-1.65	1.43E-04	0.0026
ENST00000498351	-1.65	1.05E-03	0.0066
ADD3	-1.65	3.11E-04	0.0036
OR5M2P	-1.65	2.36E-03	0.0105
DYNLT1	-1.65	5.84E-04	0.0048
ENST000003172211	-1.65	1.06E-02	0.0278
LINC01138	-1.65	7.69E-04	0.0056
AC118459.1	-1.65	2.76E-03	0.0116

RN7SL45P	-1.65	1.46E-02	0.0350
HMGN1P8	-1.65	1.32E-02	0.0326
AMT	-1.65	4.73E-03	0.0163
RNU2-2P	-1.65	5.11E-04	0.0045
ERO1B	-1.65	8.16E-04	0.0058
CNDP1	-1.65	8.27E-03	0.0236
UGDH-AS1	-1.65	2.04E-04	0.0030
SLC26A11	-1.65	1.73E-03	0.0088
PYROXD2	-1.65	5.75E-03	0.0185
RF00019.186	-1.65	1.62E-02	0.0377
SLC6A4	-1.65	1.21E-02	0.0307
FAM66C	-1.65	1.76E-03	0.0089
MIR3936HG	-1.66	1.82E-03	0.0091
AL606760.3	-1.66	3.52E-03	0.0134
TRERF1	-1.66	2.03E-03	0.0096
ADAMTSL4	-1.66	6.22E-03	0.0194
RRN3P1	-1.66	5.03E-04	0.0045
AC007743.1	-1.66	8.24E-04	0.0058
AL049796.1	-1.66	2.20E-02	0.0470
EXOC6B	-1.66	1.14E-03	0.0069
PKD1L2	-1.66	1.06E-03	0.0066
AL360091.2	-1.66	1.53E-02	0.0362
AL117336.2	-1.66	8.67E-03	0.0243
AC139530.1	-1.66	1.13E-03	0.0069
RN7SL48P	-1.66	2.17E-03	0.0100
LRRC37A4P	-1.66	3.99E-04	0.0040
UBASH3B	-1.66	3.57E-04	0.0038
MYO3B-AS1	-1.66	7.32E-03	0.0217
ANKRD44	-1.66	1.53E-04	0.0027
RN7SL523P	-1.66	1.80E-02	0.0408
PXN	-1.66	2.05E-02	0.0447
BCRP2	-1.66	2.85E-03	0.0118
OTULINL	-1.66	1.95E-04	0.0030
MPC1	-1.67	1.15E-03	0.0070
PLEKHA3P1	-1.67	2.20E-02	0.0470
AC108727.1	-1.67	2.61E-03	0.0112
CCDC91	-1.67	3.45E-04	0.0038
AC104452.1	-1.67	7.43E-03	0.0219
TAS2R45	-1.67	3.14E-03	0.0125
ENST000003447850	-1.67	2.78E-03	0.0116
AL157871.5	-1.67	1.60E-03	0.0084
RPL7L1P3	-1.67	1.90E-02	0.0423
TMEM50B	-1.67	8.93E-04	0.0061
AC103957.2	-1.67	1.22E-03	0.0072
RIMKLB	-1.67	5.12E-03	0.0171
AL355304.1	-1.67	2.16E-02	0.0464
ENST000002700011	-1.67	8.23E-04	0.0058

AL136146.2	-1.67	5.29E-03	0.0175
AURKAP1	-1.67	1.03E-03	0.0066
HACD4	-1.67	3.75E-04	0.0039
CYP2C9	-1.67	1.90E-03	0.0093
AC087854.1	-1.67	8.53E-03	0.0241
BAZ2B	-1.67	1.53E-04	0.0027
AC095057.2	-1.68	6.92E-03	0.0209
AL139147.1	-1.68	7.46E-04	0.0055
TAS2R14	-1.68	8.64E-04	0.0059
GPR82	-1.68	2.12E-02	0.0458
CHMP4BP1	-1.68	1.27E-03	0.0074
ATP2B1	-1.68	4.57E-04	0.0043
AC242842.1	-1.68	1.01E-03	0.0065
GCSAML	-1.68	1.18E-03	0.0071
HTRA4	-1.68	1.12E-03	0.0069
APOBR	-1.68	5.96E-03	0.0190
ENST000003376300	-1.68	5.44E-03	0.0178
AC114400.1	-1.68	2.15E-02	0.0463
AC092757.3	-1.68	1.21E-02	0.0308
ODF2L	-1.68	2.49E-04	0.0033
VNN3	-1.68	1.38E-03	0.0077
MAN1C1	-1.68	1.32E-03	0.0075
DISC1-IT1	-1.68	1.50E-03	0.0081
AL162457.1	-1.68	3.95E-03	0.0144
GOLGA8M	-1.68	1.21E-02	0.0306
ENST00000640587	-1.68	1.41E-03	0.0078
FBXO16	-1.68	2.09E-02	0.0453
ZNF154	-1.68	9.35E-03	0.0256
RGPD4-AS1	-1.68	8.41E-03	0.0238
NLRP1	-1.68	4.41E-03	0.0155
RN7SL284P	-1.69	1.93E-02	0.0428
TECR	-1.69	7.48E-04	0.0055
GRAMD4P3	-1.69	1.38E-02	0.0336
LINC02019	-1.69	1.24E-02	0.0311
AP000662.1	-1.69	5.07E-03	0.0170
AC073530.1	-1.69	2.20E-02	0.0470
AC023389.2	-1.69	3.47E-03	0.0133
AL591623.1	-1.69	6.46E-04	0.0051
AC091614.1	-1.69	1.74E-03	0.0088
C17orf77	-1.69	1.21E-03	0.0072
DNAH1	-1.69	5.96E-03	0.0189
CREBRF	-1.69	2.37E-04	0.0032
AC008555.2	-1.69	2.38E-02	0.0498
AC011287.2	-1.69	8.29E-03	0.0236
AC138028.1	-1.69	1.95E-02	0.0431
AC024568.1	-1.69	1.16E-02	0.0298
FBLN5	-1.69	2.39E-02	0.0499

MOGAT3	-1.69	2.15E-03	0.0099
SSTR5-AS1	-1.69	1.95E-02	0.0431
LINC01176	-1.69	6.71E-03	0.0205
Z82217.1	-1.69	1.26E-03	0.0073
AF131216.3	-1.69	9.76E-03	0.0263
SSPO	-1.69	1.15E-02	0.0296
LINC01776	-1.69	2.21E-03	0.0101
AL356235.1	-1.69	9.26E-04	0.0062
INPP4A	-1.69	2.11E-04	0.0030
SLC8B1	-1.69	8.73E-04	0.0060
TACC2	-1.69	7.54E-04	0.0055
AC010643.1	-1.70	1.05E-02	0.0277
LINC00205	-1.70	5.93E-04	0.0049
EIF1	-1.70	7.02E-04	0.0054
LINC02199	-1.70	1.10E-02	0.0287
NEB	-1.70	2.08E-03	0.0097
AC099792.1	-1.70	5.71E-03	0.0184
CHST5	-1.70	2.21E-02	0.0472
RNF38	-1.70	2.07E-02	0.0450
AL135999.1	-1.70	7.99E-04	0.0057
AL591485.1	-1.70	1.81E-02	0.0409
RN7SL282P	-1.70	1.99E-02	0.0437
PITPNM2	-1.70	4.42E-03	0.0155
AL160191.1	-1.70	1.10E-03	0.0068
AC104046.1	-1.70	1.07E-02	0.0280
DOP1A	-1.70	1.94E-04	0.0030
MIR4473	-1.70	2.10E-02	0.0455
AL391261.2	-1.70	2.86E-03	0.0118
ERVK-28	-1.70	4.57E-03	0.0159
SNORA74B	-1.70	5.60E-04	0.0047
TRMO	-1.71	5.48E-04	0.0046
MUC20	-1.71	2.11E-03	0.0098
SGO1-AS1	-1.71	6.15E-03	0.0193
AC139494.4	-1.71	6.57E-03	0.0202
AL133520.1	-1.71	1.97E-02	0.0434
ENST000003580750	-1.71	1.94E-03	0.0094
AL133492.1	-1.71	1.12E-02	0.0290
AC099550.1	-1.71	1.33E-03	0.0075
CYYR1-AS1	-1.71	1.29E-03	0.0074
Z98884.2	-1.71	7.10E-03	0.0213
GABBR1	-1.71	5.10E-04	0.0045
OR2A9P	-1.71	1.18E-02	0.0301
TBC1D4	-1.71	1.98E-04	0.0030
KLF11	-1.71	5.25E-03	0.0174
TAL1	-1.71	1.54E-02	0.0364
AL080317.3	-1.71	2.27E-03	0.0103
NPAP1	-1.71	1.98E-03	0.0095

CNTNAP2	-1.71	5.05E-03	0.0170
PBX3	-1.71	4.41E-04	0.0042
L3MBTL4-AS1	-1.71	9.68E-04	0.0064
ENST000003620061	-1.71	8.72E-03	0.0244
AC087286.4	-1.72	4.97E-03	0.0168
AC091170.1	-1.72	5.93E-03	0.0189
SGSM1	-1.72	2.36E-03	0.0105
LINC00426	-1.72	4.56E-04	0.0043
TNFRSF10A	-1.72	1.15E-03	0.0069
SETD1B	-1.72	3.16E-03	0.0126
AL590867.1	-1.72	2.36E-04	0.0032
AC139494.6	-1.72	2.19E-03	0.0100
ENST00000640719	-1.72	5.06E-04	0.0045
AKR1B15	-1.72	2.91E-03	0.0119
TRIM52-AS1	-1.72	1.65E-03	0.0086
AC009185.1	-1.72	2.30E-03	0.0104
AL356599.1	-1.72	3.75E-04	0.0039
AP001107.1	-1.72	1.71E-03	0.0087
SYNPO2	-1.72	1.11E-03	0.0068
AL355482.1	-1.72	1.46E-03	0.0080
CYB561	-1.72	7.52E-03	0.0221
NPM1P37	-1.72	2.24E-02	0.0476
OVCH1-AS1	-1.72	4.78E-04	0.0044
SLC25A25-AS1	-1.72	9.00E-03	0.0250
AC004835.1	-1.72	4.79E-03	0.0164
AC004461.2	-1.72	4.18E-03	0.0150
LINC01653	-1.72	1.05E-03	0.0066
EPHX2	-1.73	2.73E-04	0.0034
DGCR11	-1.73	1.08E-03	0.0067
PPIAL4G	-1.73	2.34E-03	0.0105
AC128685.1	-1.73	2.18E-03	0.0100
TPTE2P5	-1.73	1.26E-03	0.0073
AC138827.1	-1.73	3.75E-03	0.0140
ENST00000640720	-1.73	5.89E-04	0.0049
ST3GAL5	-1.73	4.13E-04	0.0041
AC116562.4	-1.73	1.64E-02	0.0381
CST3	-1.73	1.51E-02	0.0359
AC129510.2	-1.73	1.11E-02	0.0288
AC027544.1	-1.73	8.19E-04	0.0058
MAL	-1.73	4.08E-04	0.0041
BRWD1-IT1	-1.73	7.59E-03	0.0223
TRBV29-1	-1.73	6.92E-04	0.0053
AL512306.3	-1.73	9.79E-03	0.0264
MIR590	-1.73	9.79E-03	0.0264
AL121936.1	-1.73	2.20E-03	0.0101
AL354977.2	-1.73	9.16E-03	0.0253
C5orf56	-1.73	6.28E-03	0.0195

LINC002481	-1.73	1.68E-02	0.0388
CERS6	-1.73	1.78E-03	0.0089
AC004854.2	-1.73	9.39E-03	0.0257
LINC02084	-1.73	1.74E-02	0.0397
39508	-1.73	4.10E-04	0.0041
AL356481.2	-1.73	2.27E-02	0.0481
ZBTB18	-1.73	4.99E-04	0.0045
GRIK2	-1.73	1.88E-02	0.0420
TAS2R64P	-1.74	3.37E-04	0.0037
TNFAIP3	-1.74	1.41E-04	0.0026
PHLDB2	-1.74	1.94E-03	0.0094
AL354993.1	-1.74	2.65E-03	0.0113
ZHX2	-1.74	8.62E-03	0.0242
RN7SL619P	-1.74	1.34E-02	0.0329
AC025171.3	-1.74	2.02E-02	0.0442
AC005911.1	-1.74	9.16E-03	0.0253
SCARNA3	-1.74	4.86E-03	0.0165
SELENOM	-1.74	4.25E-03	0.0152
ENST00000640347	-1.74	1.35E-04	0.0025
LINC00562	-1.74	6.69E-03	0.0204
RF00017.10	-1.74	1.74E-02	0.0398
AC003102.1	-1.74	5.60E-03	0.0182
SCART1	-1.74	7.35E-04	0.0055
AL136526.1	-1.74	4.27E-03	0.0152
KIR3DX1	-1.74	1.06E-03	0.0067
LINC02009	-1.74	8.43E-03	0.0239
ZBTB46-AS1	-1.74	4.49E-03	0.0157
RNASET2	-1.74	1.96E-03	0.0094
TRBJ1-6	-1.74	6.92E-03	0.0209
AC138150.1	-1.74	4.69E-03	0.0162
MED13L	-1.74	2.44E-04	0.0032
AKT3	-1.75	1.53E-04	0.0027
TANC2	-1.75	2.94E-03	0.0120
KLHL3	-1.75	5.79E-04	0.0048
ABO	-1.75	3.20E-03	0.0126
SLC10A2	-1.75	3.45E-03	0.0132
NBPF2P	-1.75	1.04E-03	0.0066
PCNX2	-1.75	9.44E-04	0.0063
AC020978.5	-1.75	1.79E-02	0.0406
FSIP2	-1.75	4.23E-04	0.0041
A1CF	-1.75	1.85E-02	0.0415
RF00017.29	-1.75	1.30E-02	0.0322
MDGA1	-1.75	4.49E-03	0.0157
SERINC5	-1.75	1.89E-04	0.0029
OR56B2P	-1.75	1.21E-02	0.0306
EFCAB3	-1.75	9.25E-04	0.0062
SCARNA7	-1.75	1.31E-04	0.0025

FAM226B	-1.75	1.77E-02	0.0402
AC099568.2	-1.75	4.94E-03	0.0167
DNAH8	-1.75	1.56E-02	0.0367
HNF4A	-1.75	1.56E-03	0.0083
LINC02576	-1.75	1.67E-02	0.0386
AL390195.2	-1.75	1.40E-03	0.0078
AC093726.1	-1.75	5.26E-03	0.0174
ENST00000639970	-1.75	4.55E-04	0.0043
AC008752.3	-1.76	5.07E-03	0.0170
LINC02075	-1.76	3.12E-03	0.0124
DDX43	-1.76	4.86E-04	0.0044
TAS2R30	-1.76	7.49E-04	0.0055
SLC3A1	-1.76	6.54E-03	0.0201
EDA	-1.76	3.03E-03	0.0122
PPP3CA	-1.76	9.48E-04	0.0063
ATP9B	-1.76	1.30E-04	0.0025
NCF1	-1.76	4.15E-03	0.0149
RASA3	-1.76	1.56E-04	0.0027
SNORD38C	-1.76	9.37E-03	0.0256
AL162391.1	-1.76	6.87E-03	0.0208
PPIL6	-1.76	1.08E-02	0.0282
UGT1A7	-1.76	7.33E-03	0.0217
LINC02275	-1.76	3.76E-03	0.0140
LCA10	-1.76	1.47E-02	0.0353
MAML3	-1.76	2.35E-03	0.0105
RPS2P45	-1.76	4.29E-03	0.0153
MLANA	-1.76	1.62E-02	0.0377
AC125603.4	-1.76	4.84E-03	0.0165
AC092747.4	-1.76	5.08E-04	0.0045
SNORA3B	-1.77	1.04E-03	0.0066
TAC4	-1.77	6.52E-04	0.0051
AC006978.2	-1.77	5.21E-04	0.0045
ATG2A	-1.77	3.42E-04	0.0038
AC136475.3	-1.77	3.93E-03	0.0144
AL354793.1	-1.77	1.71E-03	0.0087
FAM102A	-1.77	5.34E-04	0.0046
AC019257.6	-1.77	1.08E-03	0.0067
AL138955.1	-1.77	5.53E-03	0.0180
MYCBP2-AS2	-1.77	8.68E-03	0.0243
AAK1	-1.77	1.77E-03	0.0089
FCRL3	-1.77	3.39E-03	0.0131
AC084816.1	-1.77	7.66E-04	0.0056
PRDM8	-1.77	2.25E-02	0.0478
AC100832.2	-1.77	1.11E-02	0.0288
ENST000003165090	-1.77	1.08E-02	0.0282
CC2D2A	-1.77	3.61E-03	0.0137
MC1R	-1.77	9.95E-03	0.0266

ENST000002399401	-1.78	1.09E-02	0.0284
MYH14	-1.78	2.01E-02	0.0441
AC016727.1	-1.78	9.71E-04	0.0064
AC106865.1	-1.78	9.48E-04	0.0063
LPAR3	-1.78	7.99E-03	0.0231
AC092111.1	-1.78	1.96E-03	0.0094
PATE4	-1.78	2.82E-03	0.0117
CAPN3	-1.78	5.98E-03	0.0190
AC007495.1	-1.78	2.03E-03	0.0096
NHLRC4	-1.78	1.55E-02	0.0365
PLEK	-1.78	1.74E-03	0.0088
LINC01206	-1.78	1.02E-02	0.0271
WFDC11	-1.78	1.93E-02	0.0428
DUSP8	-1.78	9.12E-03	0.0252
RN7SL461P	-1.78	2.29E-02	0.0483
ZFP36	-1.78	2.07E-03	0.0097
AC022826.1	-1.78	1.59E-03	0.0084
KCNH6	-1.78	1.64E-02	0.0381
RF00017.68	-1.78	1.64E-02	0.0381
ST8SIA5	-1.78	1.86E-03	0.0092
LINC01828	-1.78	2.22E-03	0.0101
ENST000003534110	-1.78	1.28E-04	0.0025
AL023755.1	-1.79	9.88E-03	0.0265
EVL	-1.79	1.27E-04	0.0025
PDCL3	-1.79	3.85E-03	0.0142
HBP1	-1.79	6.02E-04	0.0049
HTN1	-1.79	1.64E-03	0.0085
FAM229A	-1.79	2.32E-02	0.0488
LINC02062	-1.79	1.01E-02	0.0269
KCNA3	-1.79	2.99E-04	0.0035
TPM2	-1.79	5.50E-03	0.0180
SNHG22	-1.79	1.41E-04	0.0026
AL021368.1	-1.79	4.59E-03	0.0159
C3orf35	-1.79	2.20E-03	0.0101
BBS9	-1.79	1.99E-03	0.0095
AC008574.1	-1.79	1.43E-03	0.0079
LINC00865	-1.79	1.28E-02	0.0318
SMPD3	-1.79	1.46E-02	0.0351
ENST000002893610	-1.79	4.40E-04	0.0042
AC106037.1	-1.79	1.47E-02	0.0352
GABRB3	-1.80	5.46E-03	0.0179
AC084018.2	-1.80	2.40E-03	0.0106
C2orf91	-1.80	3.82E-03	0.0141
PRH1	-1.80	1.02E-02	0.0272
PABPC1P11	-1.80	6.19E-03	0.0194
ENST000003152491	-1.80	3.75E-04	0.0039
GYG1	-1.80	4.91E-03	0.0166

AC026316.5	-1.80	1.73E-02	0.0395
EPC1	-1.80	1.34E-04	0.0025
AL513285.1	-1.80	1.94E-02	0.0429
EFCAB13	-1.80	6.02E-04	0.0049
RF00017.26	-1.80	5.98E-03	0.0190
ABCC9	-1.80	2.94E-04	0.0035
AC025569.1	-1.80	1.27E-03	0.0074
DYRK2	-1.80	1.53E-04	0.0027
SNURF	-1.80	8.68E-04	0.0060
OR11A1	-1.81	2.98E-03	0.0121
NAIP	-1.81	4.73E-04	0.0044
ENST000003668550	-1.81	1.21E-03	0.0072
AC007389.3	-1.81	7.56E-03	0.0222
DFFBP1	-1.81	1.54E-02	0.0363
IMPG2	-1.81	2.00E-03	0.0095
SMARCE1P1	-1.81	1.29E-03	0.0074
FGFR2	-1.81	1.26E-02	0.0315
AC084706.1	-1.81	1.48E-02	0.0355
USP25	-1.81	1.05E-04	0.0023
IGSF6	-1.81	2.02E-03	0.0096
AC005064.1	-1.81	5.97E-03	0.0190
CATSPERB	-1.81	7.61E-03	0.0223
FCRL2	-1.81	4.93E-03	0.0167
AP003393.1	-1.82	5.25E-03	0.0174
AC132938.2	-1.82	3.11E-03	0.0124
BCAS3	-1.82	3.78E-04	0.0039
AC009831.1	-1.82	6.24E-03	0.0195
AC091078.1	-1.82	2.27E-02	0.0481
RORA	-1.82	3.74E-04	0.0039
SYTL1	-1.82	1.57E-03	0.0083
AC025575.2	-1.82	1.63E-02	0.0378
A1BG	-1.82	1.67E-03	0.0086
AC097376.2	-1.82	2.45E-04	0.0032
AC007256.1	-1.82	1.27E-03	0.0073
PLK2	-1.82	8.89E-04	0.0060
KLK11	-1.82	8.96E-03	0.0249
KLHL6-AS1	-1.82	8.38E-03	0.0238
TAS2R46	-1.82	4.70E-03	0.0162
ENST000002665890	-1.82	2.17E-04	0.0031
SNORD63B	-1.82	5.75E-04	0.0048
PARP15	-1.83	2.67E-04	0.0034
TMC8	-1.83	1.54E-03	0.0082
PLN	-1.83	3.56E-03	0.0135
ABLIM2	-1.83	2.40E-03	0.0106
MGAT4EP	-1.83	8.71E-03	0.0244
RN7SL596P	-1.83	1.76E-02	0.0401
GNPTAB	-1.83	1.88E-03	0.0092

AMZ1	-1.83	8.48E-03	0.0240
AF228730.5	-1.83	8.72E-04	0.0060
AC114760.2	-1.83	1.54E-02	0.0363
AC109992.2	-1.83	2.99E-03	0.0121
DCAF13P3	-1.83	5.03E-03	0.0169
LINC01772	-1.83	8.59E-04	0.0059
CFP	-1.83	1.75E-03	0.0088
ANKRD29	-1.83	2.24E-02	0.0476
FABP7	-1.83	2.05E-02	0.0447
JUNB	-1.83	3.31E-04	0.0037
GOPC	-1.83	1.92E-02	0.0427
AL591848.3	-1.83	1.25E-02	0.0313
USP9YP3	-1.83	1.25E-02	0.0313
PTOV1-AS1	-1.83	1.17E-02	0.0298
LINC02057	-1.83	2.06E-03	0.0097
PNPT1P1	-1.84	4.89E-03	0.0166
SARAF	-1.84	1.90E-04	0.0029
RASSF3	-1.84	5.40E-04	0.0046
AC027117.1	-1.84	2.28E-02	0.0482
OR9H1P	-1.84	7.53E-03	0.0221
PLCL2	-1.84	9.64E-05	0.0022
SORBS2	-1.84	1.37E-02	0.0336
TULP4	-1.84	4.14E-04	0.0041
OCLN	-1.84	6.97E-04	0.0053
AC005332.2	-1.84	4.23E-04	0.0041
ADAMTSL3	-1.84	2.94E-04	0.0035
DPP4	-1.84	4.93E-03	0.0167
AC048382.6	-1.84	7.07E-03	0.0212
ADGRE4P	-1.84	4.64E-03	0.0161
AL589740.1	-1.84	5.03E-03	0.0169
TRAM2	-1.84	9.35E-04	0.0062
GABRG2	-1.84	3.32E-04	0.0037
AC015911.8	-1.84	1.92E-04	0.0029
ARHGEF3	-1.84	9.06E-04	0.0061
SLC2A3	-1.84	3.20E-04	0.0037
ENST000003019233	-1.84	1.71E-03	0.0087
AC012074.1	-1.85	3.35E-03	0.0130
MOAP1	-1.85	2.30E-04	0.0032
REXO1L1P	-1.85	5.46E-04	0.0046
ENST000002618371	-1.85	1.75E-03	0.0089
AC022929.2	-1.85	4.99E-03	0.0168
RNASE6	-1.85	1.97E-03	0.0095
LINC01163	-1.85	9.60E-03	0.0261
CAPS	-1.85	1.24E-03	0.0073
KLHL24	-1.85	1.95E-03	0.0094
AC087473.1	-1.85	2.67E-04	0.0034
LEPROTL1	-1.85	1.71E-04	0.0028

LRRC37A2	-1.85	1.45E-02	0.0350
TRANK1	-1.85	1.40E-02	0.0341
AC009264.1	-1.85	1.45E-03	0.0080
G6PC	-1.85	7.16E-03	0.0214
AL049874.3	-1.86	4.79E-04	0.0044
JMJD7-PLA2G4B	-1.86	1.84E-03	0.0091
NUTM2D	-1.86	8.73E-03	0.0244
AC022535.1	-1.86	1.27E-02	0.0317
AL359641.1	-1.86	2.65E-03	0.0113
KDM5D	-1.86	8.21E-05	0.0021
AC092159.2	-1.86	1.31E-03	0.0075
ACTG1P3	-1.86	1.09E-02	0.0284
PCAT19	-1.86	1.37E-02	0.0335
SCN2A	-1.86	1.04E-03	0.0066
ENST00000517762	-1.86	9.72E-03	0.0263
ENST000003156842	-1.86	5.04E-04	0.0045
IGHV4-34	-1.87	7.70E-03	0.0225
ENST000002669430	-1.87	6.38E-04	0.0051
IL15	-1.87	3.62E-04	0.0038
SLC26A7	-1.87	1.60E-02	0.0374
BEND6	-1.87	6.36E-04	0.0051
LINC01873	-1.87	3.99E-04	0.0040
SLITRK5	-1.87	7.57E-03	0.0222
AL353807.3	-1.87	9.92E-03	0.0266
MIATNB	-1.87	6.24E-04	0.0050
AIDA	-1.87	9.48E-03	0.0258
PNRC1	-1.87	2.80E-04	0.0034
AL023584.2	-1.87	2.65E-03	0.0113
LINC01619	-1.87	5.69E-03	0.0184
LINC02444	-1.88	1.05E-03	0.0066
ASB4	-1.88	1.02E-03	0.0065
ENST000003171182	-1.88	1.76E-03	0.0089
AL021877.2	-1.88	2.30E-03	0.0104
SSBP2	-1.88	9.70E-05	0.0022
LINC02503	-1.88	3.13E-03	0.0125
AC243629.2	-1.88	1.60E-02	0.0375
AF001548.2	-1.88	1.60E-02	0.0375
ROR1-AS1	-1.88	3.51E-04	0.0038
SGK3	-1.88	1.32E-04	0.0025
AL671710.1	-1.88	1.81E-02	0.0410
ITGB1	-1.88	6.48E-04	0.0051
AC021092.1	-1.88	4.69E-04	0.0043
CR589904.1	-1.88	1.39E-02	0.0338
NOL4L	-1.88	7.87E-04	0.0056
TSPAN19	-1.88	9.25E-03	0.0254
SNORA38B	-1.88	1.15E-02	0.0296
MARCH6	-1.88	1.12E-03	0.0068

LNX1-AS2	-1.89	6.85E-03	0.0208
CA5B	-1.89	2.82E-04	0.0035
AC020907.3	-1.89	5.19E-03	0.0173
ENST000002956854	-1.89	1.19E-02	0.0304
LINC01224	-1.89	1.68E-02	0.0388
ENST000003080080	-1.89	1.31E-04	0.0025
SLC46A3	-1.89	3.59E-03	0.0136
NALCN	-1.89	2.10E-02	0.0455
AL359220.1	-1.89	3.49E-04	0.0038
POU5F1	-1.89	9.33E-03	0.0256
AC010889.1	-1.89	7.54E-04	0.0055
CRISPLD2	-1.89	3.44E-03	0.0132
AC022113.1	-1.89	4.55E-03	0.0158
FFAR2	-1.89	3.10E-03	0.0124
ENST000003212502	-1.89	1.87E-04	0.0029
AC024075.3	-1.89	2.13E-02	0.0460
AC096543.1	-1.89	6.09E-03	0.0192
LIMD2	-1.89	1.27E-04	0.0025
LINC01787	-1.89	2.27E-02	0.0481
SIGIRR	-1.89	7.05E-04	0.0054
MIR3140	-1.89	1.20E-03	0.0071
RNFT1-DT	-1.89	5.11E-04	0.0045
AC009135.1	-1.90	6.62E-04	0.0052
RNU6ATAC24P	-1.90	1.15E-02	0.0295
AC009053.2	-1.90	7.55E-03	0.0222
ENST000002730090	-1.90	1.13E-02	0.0292
LRRC37A5P	-1.90	1.51E-02	0.0359
AC090425.2	-1.90	1.55E-02	0.0365
TIAF1	-1.90	1.15E-03	0.0070
PPARGC1A	-1.90	1.74E-02	0.0398
ZFP14	-1.90	1.39E-03	0.0077
TAS2R43	-1.90	1.33E-03	0.0075
AP000959.1	-1.90	2.38E-03	0.0106
GPRIN3	-1.90	1.35E-04	0.0025
RABGAP1L-IT1	-1.90	1.08E-03	0.0067
AC027612.2	-1.90	1.34E-03	0.0076
AP001992.1	-1.90	1.04E-03	0.0066
ARRDC4	-1.90	2.24E-03	0.0102
CTSO	-1.90	2.01E-03	0.0096
CADM2-AS1	-1.90	1.48E-03	0.0080
AC114980.1	-1.90	7.31E-04	0.0055
AC006238.1	-1.90	1.15E-02	0.0295
AC017002.5	-1.90	1.84E-03	0.0091
HLA-V	-1.91	1.73E-03	0.0088
CPED1	-1.91	6.26E-03	0.0195
LTB	-1.91	1.60E-02	0.0374
PDE3B	-1.91	1.15E-04	0.0024

SMCHD1	-1.91	7.09E-05	0.0019
LINC01578	-1.91	8.47E-05	0.0021
AC099063.4	-1.91	3.77E-03	0.0140
BPTFP1	-1.91	4.89E-03	0.0166
PCDHB13	-1.91	5.25E-03	0.0174
ADAM12	-1.91	5.80E-03	0.0186
SLC47A1	-1.91	1.09E-02	0.0285
NWD1	-1.91	9.73E-03	0.0263
BX248409.1	-1.91	2.34E-02	0.0491
AC079807.1	-1.91	8.66E-03	0.0243
AL133243.3	-1.91	1.35E-04	0.0025
KLRK1	-1.91	1.65E-03	0.0085
LINC02035	-1.91	2.61E-03	0.0112
SERPINB9P1	-1.91	7.26E-04	0.0054
AC139494.2	-1.92	6.95E-04	0.0053
AP002026.1	-1.92	1.26E-03	0.0073
SOX2-OT	-1.92	5.62E-03	0.0182
HDC	-1.92	5.85E-03	0.0187
ZNF394	-1.92	1.06E-04	0.0023
MEGF9	-1.92	2.15E-04	0.0031
CXCL16	-1.92	2.02E-03	0.0096
EMX2	-1.92	1.13E-02	0.0291
KCNE4	-1.92	4.73E-03	0.0163
PDE7A	-1.92	6.11E-04	0.0050
LINC01299	-1.92	7.99E-03	0.0231
C5AR2	-1.93	6.69E-04	0.0052
AP001011.1	-1.93	3.08E-03	0.0123
RF00019.227	-1.93	1.59E-02	0.0373
CXXC5	-1.93	9.49E-03	0.0259
LINC01531	-1.93	1.06E-02	0.0279
AC090377.1	-1.93	9.88E-03	0.0265
ENST000002472262	-1.93	1.88E-03	0.0092
SNRK	-1.93	2.26E-04	0.0031
LL22NC03-63E9.3	-1.93	1.67E-03	0.0086
LGR4	-1.93	1.43E-03	0.0079
TRBV19	-1.93	1.74E-03	0.0088
NONOP2	-1.93	2.23E-02	0.0474
AC127002.1	-1.93	1.67E-02	0.0386
AC116158.1	-1.93	9.85E-04	0.0064
PEG13	-1.93	1.07E-03	0.0067
AC048341.1	-1.93	5.45E-04	0.0046
WHAMMP3	-1.93	1.67E-04	0.0028
ACAP2-IT1	-1.94	5.26E-04	0.0046
AC243651.1	-1.94	1.72E-02	0.0394
PDCL3P4	-1.94	1.75E-03	0.0089
AC010168.1	-1.94	3.02E-03	0.0122
AP003486.1	-1.94	8.09E-04	0.0057

ATXN7L1	-1.94	1.63E-04	0.0027
SYCP1	-1.94	7.04E-04	0.0054
AL356801.1	-1.94	2.02E-03	0.0096
AL355336.1	-1.94	1.57E-02	0.0370
IGFL4	-1.94	9.51E-04	0.0063
AL139407.1	-1.94	6.07E-04	0.0049
P2RY14	-1.94	1.10E-03	0.0068
AC096921.2	-1.94	3.59E-04	0.0038
AC020663.3	-1.94	5.71E-04	0.0048
AC127024.4	-1.94	1.41E-02	0.0343
EPC2	-1.94	2.12E-04	0.0031
LAMA2	-1.94	5.41E-03	0.0178
RN7SL670P	-1.95	1.98E-02	0.0435
PRKG1-AS1	-1.95	4.93E-03	0.0167
FHIT	-1.95	3.86E-04	0.0040
AC015819.1	-1.95	2.07E-03	0.0097
AC011297.1	-1.95	9.62E-03	0.0261
AC245078.3	-1.95	1.68E-03	0.0086
EPS15P1	-1.95	2.05E-02	0.0447
AC005332.5	-1.95	7.79E-04	0.0056
OR8A1	-1.95	3.42E-04	0.0038
LINC01483	-1.95	9.30E-03	0.0255
AC009227.1	-1.95	9.39E-04	0.0063
AC004672.2	-1.95	1.12E-02	0.0291
AC087392.3	-1.95	4.53E-03	0.0158
SHANK1	-1.95	1.97E-02	0.0434
AL512306.2	-1.95	2.02E-03	0.0096
VMAC	-1.95	3.97E-04	0.0040
PLXNC1	-1.96	1.32E-03	0.0075
RF00017.146	-1.96	9.49E-03	0.0259
AC022182.1	-1.96	5.02E-03	0.0169
RF00019.655	-1.96	5.42E-03	0.0178
LINC02108	-1.96	8.07E-04	0.0057
CD101	-1.96	5.73E-03	0.0185
COL28A1	-1.96	6.65E-03	0.0203
PHF1	-1.96	5.22E-04	0.0045
AC002451.1	-1.96	1.84E-02	0.0415
AC008869.1	-1.96	1.13E-02	0.0293
AL022310.1	-1.96	8.40E-04	0.0058
CCNB3P1	-1.96	1.76E-02	0.0401
LINC00869	-1.96	1.85E-03	0.0091
AC011825.3	-1.96	2.08E-03	0.0097
SKOR1	-1.96	9.52E-03	0.0259
ARHGAP27P1-BPTFP1-KPN/	-1.96	5.72E-03	0.0184
AL356776.1	-1.96	6.44E-04	0.0051
PDP1	-1.96	1.58E-04	0.0027
CACNA1C-AS2	-1.96	2.92E-03	0.0119

GIMAP1	-1.96	1.16E-03	0.0070
SEC14L6	-1.97	8.54E-04	0.0059
BCORP1	-1.97	3.33E-03	0.0130
LINC01205	-1.97	2.20E-03	0.0101
SOX9-AS1	-1.97	1.78E-03	0.0089
SCARNA21	-1.97	7.41E-05	0.0020
AC110792.3	-1.97	1.28E-02	0.0320
AC135068.8	-1.97	7.86E-03	0.0228
IGHV1OR15-1	-1.97	7.86E-03	0.0228
AC083949.1	-1.97	1.24E-02	0.0312
RF00019.710	-1.97	1.24E-02	0.0312
AC068587.4	-1.97	9.50E-05	0.0022
STK38	-1.97	1.30E-04	0.0025
CYP4F8	-1.97	4.40E-04	0.0042
PLEKHA1	-1.97	1.70E-04	0.0028
XYLT1	-1.97	4.36E-04	0.0042
AL354941.1	-1.98	9.66E-03	0.0262
AC009041.2	-1.98	1.07E-03	0.0067
SLC44A5	-1.98	3.41E-03	0.0131
MECOM	-1.98	3.10E-03	0.0124
ALDH3B1	-1.98	4.17E-04	0.0041
ADCY10P1	-1.98	1.33E-03	0.0075
NEK10	-1.98	2.58E-03	0.0111
TRPM8	-1.98	1.42E-03	0.0078
AL365204.2	-1.98	1.78E-02	0.0404
AC079684.1	-1.98	3.28E-03	0.0128
SKINT1L	-1.98	1.25E-03	0.0073
AC004039.1	-1.98	2.58E-03	0.0111
RF00019.145	-1.98	8.66E-04	0.0060
DCN	-1.98	4.33E-03	0.0154
SYT17	-1.98	1.85E-02	0.0416
RNU6-838P	-1.98	1.26E-02	0.0315
AC018645.2	-1.98	2.10E-02	0.0455
LINC02260	-1.98	1.40E-02	0.0340
AC005070.3	-1.99	3.21E-03	0.0127
LINC00891	-1.99	3.61E-04	0.0038
CASS4	-1.99	3.31E-03	0.0129
LAMC2	-1.99	1.52E-02	0.0361
AHNAK	-1.99	4.50E-05	0.0016
AC006369.1	-1.99	9.56E-04	0.0063
AP000766.1	-1.99	5.47E-04	0.0046
HSPE1P3	-1.99	6.22E-03	0.0194
KIF13A	-1.99	1.27E-02	0.0317
RF01241.1	-1.99	3.79E-03	0.0141
AC007728.2	-1.99	4.46E-03	0.0156
KLB	-1.99	7.15E-03	0.0213
RASGRF2	-2.00	1.56E-03	0.0083

AC008079.1	-2.00	4.97E-04	0.0045
SHISAL2A	-2.00	4.37E-04	0.0042
MYLK	-2.00	2.24E-02	0.0477
SCN9A	-2.00	2.47E-03	0.0108
TRDMT1	-2.00	1.43E-04	0.0026
AL359752.1	-2.00	8.45E-04	0.0059
SULT1B1	-2.00	2.40E-04	0.0032
GYG2P1	-2.00	2.32E-03	0.0104
CDC20B	-2.00	8.83E-03	0.0246
KDM5C-IT1	-2.00	5.79E-03	0.0186
AC009065.5	-2.00	9.19E-03	0.0253
TOB1	-2.00	1.43E-04	0.0026
CGRRF1	-2.00	3.09E-03	0.0124
SNORD59A	-2.01	1.39E-02	0.0339
SNORA54	-2.01	2.75E-03	0.0115
ALDH1L1	-2.01	1.64E-02	0.0381
CCDC7	-2.01	3.74E-04	0.0039
LINC01307	-2.01	1.13E-02	0.0291
SF3B1	-2.01	7.83E-05	0.0020
TXLNB	-2.01	3.46E-03	0.0133
AL513548.1	-2.01	7.30E-03	0.0217
ENST000002609081	-2.01	1.11E-04	0.0023
AP002812.5	-2.01	2.14E-03	0.0099
AC006042.3	-2.01	4.55E-04	0.0043
RC3H1-IT1	-2.01	1.08E-03	0.0067
AC006333.2	-2.01	9.23E-04	0.0062
CYP4V2	-2.01	1.34E-03	0.0076
SNORD17	-2.01	7.66E-05	0.0020
RF00017.74	-2.01	7.50E-03	0.0221
CD55	-2.01	2.04E-03	0.0096
PDXDC2P	-2.01	1.02E-03	0.0065
LINC01410	-2.02	3.02E-03	0.0122
PHYHD1	-2.02	5.76E-03	0.0185
AL356019.2	-2.02	1.80E-03	0.0090
RNU6-118P	-2.02	1.51E-02	0.0359
PATJ	-2.02	3.44E-04	0.0038
PSMA3-AS1	-2.02	1.45E-04	0.0026
LINC00355	-2.03	5.03E-03	0.0169
AC090220.1	-2.03	4.05E-03	0.0147
SCARNA6	-2.03	7.52E-05	0.0020
B3GAT1	-2.03	1.15E-03	0.0070
FAM214A	-2.03	4.19E-04	0.0041
C20orf203	-2.03	4.98E-03	0.0168
VAC14-AS1	-2.03	1.15E-03	0.0069
AC108751.4	-2.03	4.89E-03	0.0166
LINC01145	-2.03	1.14E-04	0.0024
MYLK4	-2.03	1.66E-03	0.0086

PLD5	-2.04	1.14E-02	0.0293
SCARNA10	-2.04	5.22E-05	0.0017
KCNH2	-2.04	8.77E-03	0.0245
MGC16275	-2.04	1.14E-02	0.0294
RF00017.174	-2.04	8.72E-03	0.0244
NFKBIZ	-2.04	1.40E-04	0.0026
AC012085.2	-2.04	5.27E-03	0.0174
AC241520.1	-2.04	1.26E-02	0.0316
AC020659.1	-2.04	5.82E-03	0.0187
AC079781.5	-2.04	3.20E-04	0.0037
SCARB2	-2.04	1.32E-04	0.0025
AC135050.5	-2.04	6.07E-03	0.0192
AC009133.3	-2.04	3.16E-03	0.0125
AC103740.1	-2.05	6.45E-04	0.0051
ACCS	-2.05	6.30E-04	0.0050
TLE4	-2.05	9.96E-05	0.0022
AC001226.1	-2.05	1.14E-02	0.0293
RSF1-IT2	-2.05	2.82E-03	0.0117
MAPK8IP2	-2.05	1.45E-02	0.0349
SLC24A4	-2.05	1.88E-02	0.0420
THSD1	-2.05	3.43E-03	0.0132
AL353803.1	-2.05	3.55E-03	0.0135
AL138479.1	-2.05	2.24E-03	0.0102
ENST000002272140	-2.05	1.27E-03	0.0073
AL669942.1	-2.05	5.91E-03	0.0188
SYTL2	-2.05	6.97E-04	0.0053
ASAP1-IT2	-2.05	4.52E-03	0.0158
AC109927.2	-2.05	2.21E-02	0.0471
ACSS3	-2.05	1.12E-02	0.0291
ZNF10	-2.05	5.83E-04	0.0048
AC006064.2	-2.05	5.03E-03	0.0169
PBX1	-2.05	2.65E-03	0.0113
CADM1	-2.05	1.46E-03	0.0080
AC134349.1	-2.05	2.56E-03	0.0111
AC134349.4	-2.05	2.56E-03	0.0111
AC245078.2	-2.05	2.56E-03	0.0111
AL117692.1	-2.05	4.04E-03	0.0147
CACNG3	-2.06	1.14E-02	0.0294
PTCH1	-2.06	3.17E-03	0.0126
AC007923.2	-2.06	7.90E-03	0.0229
LINC00221	-2.06	2.60E-04	0.0033
IRGM	-2.06	1.96E-03	0.0094
AL031283.1	-2.06	1.93E-03	0.0094
ADGRG2	-2.06	1.39E-02	0.0339
MEOX1	-2.06	1.77E-02	0.0402
AC006262.1	-2.06	3.79E-03	0.0141
AC090398.1	-2.06	1.17E-02	0.0298

RF00017.41	-2.06	9.73E-04	0.0064
AC245297.2	-2.06	2.87E-03	0.0118
LRRK1	-2.06	4.39E-04	0.0042
AL161785.1	-2.06	3.51E-03	0.0134
AC009704.2	-2.06	1.19E-02	0.0303
AC105265.3	-2.06	1.02E-02	0.0271
AP001381.1	-2.06	3.69E-03	0.0139
TP53INP2	-2.06	7.44E-04	0.0055
OR2L2	-2.06	1.79E-03	0.0090
TGFBR3	-2.07	2.64E-03	0.0113
STK31	-2.07	2.88E-03	0.0118
CASTOR3	-2.07	6.29E-04	0.0050
LINC02245	-2.07	6.55E-03	0.0201
SAMD12	-2.07	2.92E-04	0.0035
TRABD2A	-2.07	2.75E-04	0.0034
RF02271.12	-2.07	8.60E-04	0.0059
HLA-DPB1	-2.07	9.78E-05	0.0022
AL133330.1	-2.07	7.43E-04	0.0055
RF02271.32	-2.07	2.18E-03	0.0100
TNRC6C	-2.07	1.22E-04	0.0025
AC245297.3	-2.07	1.28E-04	0.0025
SYNE1	-2.07	1.45E-04	0.0026
LIX1	-2.07	3.33E-03	0.0130
AC027020.2	-2.07	5.08E-04	0.0045
POTEC	-2.08	1.90E-03	0.0093
AP002884.1	-2.08	3.18E-04	0.0037
VAV3	-2.08	8.57E-04	0.0059
AC240274.1	-2.08	2.04E-02	0.0445
LINC00347	-2.08	1.85E-03	0.0091
PDCD4-AS1	-2.08	9.57E-04	0.0063
AC025171.2	-2.08	4.38E-04	0.0042
GPR18	-2.08	6.53E-03	0.0201
RF00019.112	-2.08	4.71E-03	0.0162
AC092653.1	-2.08	7.02E-03	0.0211
ACTA2-AS1	-2.08	4.11E-04	0.0041
AC104964.2	-2.08	2.44E-03	0.0108
AF279873.3	-2.09	2.73E-03	0.0115
FAM209A	-2.09	9.19E-03	0.0253
AC055822.1	-2.09	4.86E-04	0.0044
ENST000003563220	-2.09	1.66E-03	0.0086
FBXO3	-2.09	5.69E-05	0.0017
AKR1C1	-2.09	1.45E-03	0.0079
SPP2	-2.09	4.55E-03	0.0158
FTH1	-2.09	1.40E-04	0.0026
LINC02006	-2.09	4.32E-03	0.0153
ANP32AP1	-2.09	2.06E-02	0.0448
AC008555.5	-2.10	7.85E-04	0.0056

TCEANC	-2.10	8.49E-04	0.0059
STX16-NPEPL1	-2.10	1.59E-03	0.0084
AL049840.1	-2.10	3.63E-03	0.0137
CDH8	-2.10	4.27E-03	0.0152
USP36	-2.10	9.08E-04	0.0061
LINC00476	-2.10	1.46E-03	0.0080
MSX1	-2.10	1.41E-02	0.0341
HAMP	-2.10	1.28E-03	0.0074
ENST00000602493	-2.10	9.23E-03	0.0254
AL031848.2	-2.10	1.15E-02	0.0295
TLR4	-2.10	1.15E-02	0.0295
COL6A1	-2.10	1.13E-02	0.0293
SNX29P1	-2.10	2.75E-03	0.0116
ENST00000471810	-2.10	1.72E-03	0.0087
RF00019.541	-2.11	1.16E-02	0.0297
POU5F2	-2.11	1.97E-04	0.0030
AC073957.3	-2.11	8.70E-04	0.0060
AL162578.1	-2.11	1.89E-03	0.0093
MAP3K1	-2.11	5.60E-05	0.0017
AL035446.1	-2.11	8.78E-03	0.0245
AC002553.2	-2.11	4.35E-04	0.0042
CCDC120	-2.11	5.15E-03	0.0172
AC087239.1	-2.11	1.69E-02	0.0389
AC011939.1	-2.11	1.75E-04	0.0028
AP003086.1	-2.11	1.42E-03	0.0078
ENST000003474660	-2.11	2.01E-03	0.0096
LINC00513	-2.12	1.68E-02	0.0388
ANKRD26P4	-2.12	5.07E-03	0.0170
DLEC1	-2.12	5.23E-04	0.0045
MFGE8	-2.12	5.22E-04	0.0045
AL355997.1	-2.12	2.43E-03	0.0107
ZNF781	-2.12	2.74E-04	0.0034
PAG1	-2.12	9.46E-04	0.0063
PLCB1	-2.12	4.75E-04	0.0044
EYS	-2.12	3.13E-03	0.0125
GAB1	-2.12	5.64E-03	0.0183
RGS17	-2.12	5.04E-04	0.0045
FBLIM1	-2.12	2.59E-03	0.0111
LINC02520	-2.12	5.58E-04	0.0047
ERMN	-2.12	2.44E-04	0.0032
MGC4859	-2.12	8.42E-03	0.0238
RPL21P123	-2.12	2.58E-03	0.0111
IDUA	-2.12	4.38E-03	0.0155
TRGC2	-2.13	5.90E-04	0.0049
ENST000003035210	-2.13	1.36E-03	0.0076
AC022819.1	-2.13	7.21E-04	0.0054
AC007216.3	-2.13	4.76E-04	0.0044

UTRN	-2.13	3.99E-04	0.0040
PBXIP1	-2.13	7.55E-05	0.0020
CPQ	-2.13	2.93E-04	0.0035
RN7SL592P	-2.13	1.86E-02	0.0417
OCLNP1	-2.13	1.22E-02	0.0309
ATP2B1-AS1	-2.13	2.06E-02	0.0449
ENST000003088240	-2.13	2.18E-04	0.0031
AL139393.2	-2.14	9.20E-03	0.0253
RF00017.200	-2.14	1.63E-03	0.0085
ENST000002574970	-2.14	6.35E-05	0.0019
ARHGAP12	-2.14	7.22E-05	0.0020
AC018554.1	-2.14	7.03E-04	0.0054
PRDM5	-2.14	5.43E-04	0.0046
EPHA1	-2.14	3.44E-04	0.0038
RGPD5	-2.14	9.17E-04	0.0062
ENST000003356700	-2.14	2.41E-04	0.0032
AC027288.3	-2.14	1.22E-03	0.0072
GPR183	-2.14	1.24E-03	0.0073
IGHV3-37	-2.14	2.51E-03	0.0110
AL139424.3	-2.15	8.81E-03	0.0246
AC005912.1	-2.15	1.00E-02	0.0267
ADRB2	-2.15	1.53E-03	0.0082
AP001330.1	-2.15	3.42E-04	0.0038
BBS2	-2.15	3.69E-04	0.0039
GAS7	-2.15	4.94E-04	0.0044
HLA-DMB	-2.15	6.59E-04	0.0052
LINC00336	-2.15	6.76E-03	0.0206
ENST000002778740	-2.15	2.81E-03	0.0117
RGS17P1	-2.15	5.10E-03	0.0171
LDLRAP1	-2.15	2.50E-04	0.0033
ENST000002960881	-2.16	4.42E-05	0.0016
ATF4P3	-2.16	5.25E-03	0.0174
PABPC1P3	-2.16	5.73E-04	0.0048
AC127024.6	-2.16	9.68E-03	0.0262
LCTL	-2.16	2.57E-03	0.0111
C1GALT1P1	-2.16	4.10E-03	0.0148
SAMD9	-2.16	2.24E-03	0.0102
AC073569.3	-2.16	4.80E-04	0.0044
USP46	-2.16	7.18E-05	0.0020
CLC	-2.16	9.25E-03	0.0254
RF00413.1	-2.16	2.55E-03	0.0111
ACSM1	-2.16	1.82E-02	0.0411
RF00017.62	-2.16	7.77E-03	0.0226
KIAA1551	-2.17	5.40E-05	0.0017
OMA1	-2.17	7.62E-05	0.0020
KCNMB1	-2.17	8.07E-04	0.0057
ZNF276	-2.17	5.27E-05	0.0017

MIR22HG	-2.17	5.36E-04	0.0046
VPS9D1	-2.17	9.45E-04	0.0063
SLC2A5	-2.17	6.70E-04	0.0052
RNU6-82P	-2.17	3.98E-04	0.0040
ARRDC2	-2.17	1.51E-04	0.0026
RNU6-807P	-2.17	6.21E-03	0.0194
AC017081.3	-2.17	2.09E-03	0.0098
AL513412.1	-2.17	2.19E-03	0.0100
AC048382.5	-2.18	4.01E-03	0.0146
Z94721.1	-2.18	6.20E-03	0.0194
SPAG8	-2.18	6.91E-03	0.0209
ENST000003176333	-2.18	1.43E-03	0.0079
MAGI2-AS3	-2.18	4.34E-03	0.0154
C1QTNF3	-2.18	1.67E-04	0.0028
AL135905.2	-2.18	2.61E-04	0.0033
SULT1C2	-2.18	9.03E-04	0.0061
AC002094.2	-2.18	3.07E-03	0.0123
AC122718.1	-2.19	5.15E-04	0.0045
PNPLA2	-2.19	2.90E-03	0.0119
GABRG3	-2.19	5.02E-03	0.0169
LMO7	-2.19	2.74E-04	0.0034
LINC01252	-2.19	6.14E-03	0.0193
AC016708.1	-2.19	1.54E-04	0.0027
CASP17P	-2.19	3.08E-04	0.0036
ITM2B	-2.19	5.15E-05	0.0017
SNORD89	-2.19	2.46E-04	0.0032
AL591926.7	-2.19	3.24E-04	0.0037
UCKL1-AS1	-2.19	7.76E-03	0.0226
KLF12	-2.19	4.41E-04	0.0042
SNORA19	-2.19	4.99E-04	0.0045
AC118344.1	-2.20	8.66E-03	0.0243
AC025031.3	-2.20	1.76E-02	0.0400
RN7SL50P	-2.20	2.31E-02	0.0487
AC009495.2	-2.20	7.35E-03	0.0218
SPON1	-2.20	9.63E-04	0.0063
ELOCP19	-2.21	3.29E-03	0.0129
AC073111.5	-2.21	7.94E-04	0.0057
AKR1C2	-2.21	1.67E-03	0.0086
KRT17P7	-2.21	8.23E-03	0.0235
RF00017.114	-2.21	1.61E-02	0.0376
NOSTRIN	-2.21	4.87E-03	0.0166
SLC4A4	-2.21	5.75E-03	0.0185
TLR7	-2.21	5.92E-03	0.0189
PDE4B	-2.21	1.95E-04	0.0030
AL360169.2	-2.22	1.23E-03	0.0072
AC011939.2	-2.22	2.53E-03	0.0110
RPL11P3	-2.22	9.72E-04	0.0064

C1DP5	-2.22	3.48E-03	0.0133
AC015911.4	-2.22	2.76E-03	0.0116
ARRDC3	-2.22	4.30E-03	0.0153
AC090589.3	-2.22	1.22E-02	0.0308
AC008555.1	-2.23	1.62E-02	0.0377
SLC4A5	-2.23	5.16E-04	0.0045
RN7SL618P	-2.23	7.26E-03	0.0216
AC034236.2	-2.23	1.56E-03	0.0083
AC126389.1	-2.23	5.19E-03	0.0173
PCSK5	-2.23	1.28E-02	0.0320
AC006305.1	-2.23	7.27E-03	0.0216
ENST000002814710	-2.23	5.82E-04	0.0048
AP000842.1	-2.23	2.37E-02	0.0495
AC089998.3	-2.23	2.20E-02	0.0470
ITGB2-AS1	-2.23	4.17E-04	0.0041
C17orf107	-2.23	1.79E-03	0.0090
ENST000003615440	-2.23	3.40E-03	0.0131
ACVR2A	-2.23	1.07E-03	0.0067
AC105233.5	-2.23	2.09E-04	0.0030
OPCML-IT1	-2.23	4.55E-03	0.0158
AL445531.1	-2.23	2.12E-03	0.0099
AL662844.4	-2.23	1.73E-04	0.0028
AC104564.5	-2.23	2.36E-04	0.0032
RARRES3	-2.24	2.89E-04	0.0035
CDH12	-2.24	2.12E-03	0.0099
AC092683.1	-2.24	6.61E-05	0.0019
GCOM1	-2.24	5.61E-04	0.0047
AC083806.2	-2.24	3.21E-03	0.0127
SH3BP5-AS1	-2.24	1.46E-03	0.0080
AC025034.1	-2.24	9.84E-03	0.0265
MGST1	-2.24	1.24E-03	0.0073
PAXBP1-AS1	-2.25	1.99E-03	0.0095
MDS2	-2.25	1.28E-03	0.0074
TC2N	-2.25	1.71E-04	0.0028
PRKG2	-2.25	2.32E-04	0.0032
DENND6A-DT	-2.25	6.58E-03	0.0202
AC091182.1	-2.25	2.08E-02	0.0452
RN7SKP151	-2.25	4.29E-03	0.0153
GPR150	-2.25	1.19E-02	0.0303
CEP68	-2.25	1.40E-04	0.0026
AP001062.1	-2.25	4.13E-03	0.0149
RPL21P44	-2.25	6.44E-03	0.0199
GPR174	-2.26	1.48E-04	0.0026
LINC00271	-2.26	1.07E-03	0.0067
RALGPS2	-2.26	5.42E-04	0.0046
ALOX5	-2.27	2.37E-04	0.0032
APBA2	-2.27	1.06E-04	0.0023

SUDS3P1	-2.27	5.10E-03	0.0171
AC074367.1	-2.27	1.68E-02	0.0388
KPNA2P3	-2.27	2.83E-03	0.0117
SNORA81	-2.27	1.17E-04	0.0024
GTF2IRD2B	-2.27	2.15E-04	0.0031
AARD	-2.27	1.89E-02	0.0421
AC005740.4	-2.27	1.89E-02	0.0421
AC019131.2	-2.27	6.61E-04	0.0052
AC106795.2	-2.27	1.97E-02	0.0434
AC106795.9	-2.27	1.97E-02	0.0434
ZNF331	-2.27	1.41E-03	0.0078
RNU6-202P	-2.27	2.10E-02	0.0455
FCRL1	-2.27	4.90E-03	0.0166
ENST000003362920	-2.27	3.11E-04	0.0036
COLQ	-2.27	6.99E-04	0.0053
DENND5B	-2.27	3.40E-03	0.0131
P2RY8	-2.28	6.29E-05	0.0019
CD200R1	-2.28	1.47E-03	0.0080
AKT3-IT1	-2.28	7.56E-04	0.0055
ENST000003414550	-2.28	1.97E-04	0.0030
ENST000002957560	-2.28	8.56E-05	0.0021
SNORD63	-2.28	1.71E-02	0.0392
FMR1-IT1	-2.28	1.09E-03	0.0068
AL136979.1	-2.28	1.35E-02	0.0331
DDX60L	-2.28	3.36E-03	0.0130
AL121823.1	-2.28	7.54E-03	0.0222
PKD1L1	-2.28	1.97E-02	0.0434
SPTLC1P1	-2.28	3.42E-03	0.0132
AC020912.1	-2.29	6.82E-03	0.0207
LAMA4	-2.29	3.21E-04	0.0037
PSG5	-2.29	1.09E-02	0.0285
CDK14	-2.29	2.35E-02	0.0492
MCUR1P1	-2.29	1.25E-02	0.0313
AC139768.1	-2.30	2.67E-04	0.0034
FAM238C	-2.30	2.89E-04	0.0035
AC006947.1	-2.30	9.96E-04	0.0064
ADAMTS2	-2.30	8.31E-03	0.0236
AC087667.1	-2.30	1.39E-02	0.0338
BTK	-2.30	6.93E-03	0.0209
KRT17P5	-2.30	2.19E-03	0.0100
ENST000003098620	-2.30	1.29E-03	0.0074
LINC00893	-2.31	2.00E-03	0.0095
AC027544.2	-2.31	2.85E-03	0.0118
ENST000002414161	-2.31	6.30E-04	0.0050
AC090229.1	-2.31	2.56E-03	0.0111
LINC01727	-2.31	8.34E-04	0.0058
AC084036.1	-2.31	1.16E-02	0.0297

LINC00920	-2.31	2.26E-02	0.0478
CAMK4	-2.31	5.27E-05	0.0017
FCMR	-2.31	9.41E-05	0.0022
LINC01967	-2.31	1.35E-02	0.0332
AL449403.1	-2.31	8.08E-04	0.0057
AC084768.1	-2.31	1.76E-02	0.0400
TMEM63A	-2.31	1.23E-04	0.0025
SEMA6A-AS1	-2.31	4.79E-04	0.0044
PTPN4	-2.31	7.57E-05	0.0020
ANXA2R	-2.31	1.96E-04	0.0030
CAP2P1	-2.32	3.54E-03	0.0135
TMCC3	-2.32	4.50E-03	0.0157
RALGPS1	-2.32	2.85E-04	0.0035
RBL2	-2.32	1.26E-03	0.0073
ZNF831	-2.32	1.09E-04	0.0023
MIR194-2HG	-2.32	3.21E-03	0.0127
FANK1	-2.32	9.18E-04	0.0062
UBASH3A	-2.32	3.07E-04	0.0036
LINC01422	-2.32	1.67E-03	0.0086
CNTN6	-2.32	1.75E-02	0.0399
GRM2	-2.32	5.83E-03	0.0187
PDZD4	-2.32	2.84E-04	0.0035
AC122707.1	-2.32	9.89E-03	0.0266
HCP5B	-2.32	9.89E-03	0.0266
LINC02064	-2.32	9.89E-03	0.0266
AC008280.3	-2.33	3.53E-03	0.0134
AC009088.1	-2.33	1.08E-03	0.0067
FAM71F2	-2.33	2.09E-03	0.0098
AC063950.1	-2.33	8.35E-03	0.0237
AC116366.1	-2.33	3.51E-03	0.0134
ST8SIA6	-2.33	6.84E-04	0.0053
PDE5A	-2.33	1.10E-03	0.0068
ENST000003184450	-2.33	2.60E-04	0.0033
LINC01089	-2.33	6.07E-04	0.0049
AL109923.1	-2.33	2.43E-03	0.0107
LINC00550	-2.33	1.56E-03	0.0083
LINC00970	-2.33	5.21E-03	0.0173
AC106782.1	-2.33	7.96E-03	0.0230
AC092140.1	-2.33	1.64E-02	0.0380
AC239800.2	-2.33	3.61E-03	0.0137
ENST000003349840	-2.33	2.35E-03	0.0105
PIK3IP1-AS1	-2.34	3.00E-03	0.0122
PPP3CB-AS1	-2.34	1.28E-04	0.0025
AC005224.3	-2.34	4.96E-04	0.0044
MYLIP	-2.34	4.49E-05	0.0016
AC093283.1	-2.34	1.44E-03	0.0079
RGMB	-2.34	9.57E-03	0.0260

LAIR1	-2.35	1.77E-04	0.0028
LINC00216	-2.35	2.23E-03	0.0101
ARHGEF11	-2.35	1.28E-02	0.0320
AL928711.1	-2.35	4.87E-03	0.0166
RNF222	-2.35	5.33E-03	0.0176
CR382285.1	-2.35	2.21E-02	0.0471
TCEA3	-2.35	4.70E-03	0.0162
ENST000003036351	-2.35	4.40E-03	0.0155
EPPK1	-2.35	4.27E-04	0.0041
TTC16	-2.35	2.20E-03	0.0101
DPYD-IT1	-2.35	9.13E-04	0.0061
PLXDC2	-2.36	3.80E-03	0.0141
AADACL2	-2.36	3.97E-03	0.0145
YPEL1	-2.36	3.46E-04	0.0038
CALB1	-2.36	1.16E-02	0.0298
CABP4	-2.36	2.27E-03	0.0103
TNPO1P1	-2.36	8.96E-04	0.0061
CDKN1B	-2.36	1.45E-04	0.0026
AC012615.1	-2.36	1.41E-04	0.0026
AC244230.2	-2.36	7.58E-03	0.0222
AC139491.5	-2.36	1.40E-03	0.0078
CD96	-2.36	1.43E-04	0.0026
PER1	-2.36	2.13E-04	0.0031
AC008467.1	-2.37	1.41E-04	0.0026
TSC22D1	-2.37	3.01E-04	0.0035
TP53INP1	-2.37	6.86E-04	0.0053
FCRLA	-2.37	3.34E-03	0.0130
AC012645.4	-2.37	9.58E-03	0.0260
AC124312.1	-2.37	6.24E-03	0.0195
C11orf21	-2.37	7.92E-04	0.0057
HTN3	-2.37	7.66E-04	0.0056
ZNF204P	-2.38	4.33E-04	0.0042
GSN-AS1	-2.38	7.44E-03	0.0220
APOA2	-2.38	4.89E-03	0.0166
AMY2B	-2.38	7.75E-05	0.0020
CDH19	-2.38	1.96E-02	0.0433
CYSLTR1	-2.38	1.24E-04	0.0025
AC092794.1	-2.38	5.77E-03	0.0186
AL353729.2	-2.38	9.43E-03	0.0257
CFH	-2.38	2.04E-04	0.0030
OR2L3	-2.39	5.28E-03	0.0174
NLRP6	-2.39	8.69E-03	0.0244
MPP3	-2.39	5.91E-03	0.0188
AL606834.2	-2.39	2.30E-04	0.0032
CCSER1	-2.39	4.47E-04	0.0042
HAS3	-2.39	2.03E-02	0.0443
RF00019.328	-2.39	1.11E-02	0.0289

GDA	-2.39	6.00E-03	0.0190
RN7SL625P	-2.39	1.44E-02	0.0348
Z82206.1	-2.39	1.68E-04	0.0028
AC007333.2	-2.39	5.84E-04	0.0048
LINC00528	-2.39	8.69E-03	0.0244
SF1	-2.39	7.45E-05	0.0020
AL121839.2	-2.40	1.20E-03	0.0071
AL121839.4	-2.40	1.20E-03	0.0071
SYNE2	-2.40	8.63E-05	0.0021
THEMIS	-2.40	3.60E-05	0.0015
RF00322.6	-2.40	1.19E-02	0.0302
TSPAN2	-2.40	7.01E-04	0.0054
TPPP	-2.40	2.51E-03	0.0110
GTF2IRD2	-2.40	2.17E-04	0.0031
AC036176.2	-2.40	2.76E-04	0.0034
L3MBTL3	-2.40	3.91E-05	0.0016
AC013474.1	-2.41	3.06E-04	0.0036
DCHS1	-2.41	2.65E-03	0.0113
RNU6-60P	-2.41	1.89E-02	0.0421
TAS2R13	-2.41	6.95E-03	0.0210
CATSPERE	-2.41	2.66E-03	0.0113
RN7SL173P	-2.41	2.54E-03	0.0110
LINC01964	-2.41	6.71E-03	0.0205
IL11RA	-2.42	8.67E-05	0.0021
ENST000002332020	-2.42	1.01E-02	0.0269
LINC02472	-2.42	3.92E-04	0.0040
AC096887.1	-2.42	2.28E-02	0.0482
ENST000000253990	-2.42	1.91E-03	0.0093
AL035563.1	-2.42	7.86E-04	0.0056
ENST000003466520	-2.42	6.68E-04	0.0052
RF00019.480	-2.42	1.26E-03	0.0073
PYHIN1	-2.42	6.49E-05	0.0019
RF00019.23	-2.42	4.25E-03	0.0152
MS4A7	-2.43	1.80E-02	0.0408
RAPSN	-2.43	5.64E-03	0.0183
SAMHD1	-2.43	1.46E-04	0.0026
SCARNA5	-2.43	1.84E-05	0.0011
GRASP	-2.43	3.00E-04	0.0035
FYB1	-2.43	1.13E-04	0.0024
NOG	-2.43	8.21E-04	0.0058
SCG3	-2.43	1.83E-02	0.0412
AC104695.3	-2.44	4.53E-03	0.0158
AC006030.1	-2.44	7.59E-03	0.0222
RF01169.1	-2.44	4.39E-04	0.0042
ZMAT1	-2.44	7.29E-05	0.0020
PRKACB	-2.44	4.29E-05	0.0016
FRY	-2.44	2.01E-03	0.0096

AC010245.1	-2.45	9.59E-03	0.0260
PNMA3	-2.45	9.63E-05	0.0022
RF00019.229	-2.45	8.13E-04	0.0057
KIT	-2.45	4.76E-04	0.0044
NR1D1	-2.45	9.44E-03	0.0258
RPL21P32	-2.45	1.89E-02	0.0422
ENST000003433381	-2.45	2.88E-03	0.0118
AC125612.1	-2.45	1.50E-03	0.0081
AL359962.1	-2.46	1.33E-04	0.0025
CCDC148	-2.46	2.27E-02	0.0481
TLR5	-2.46	3.38E-03	0.0131
YPEL5	-2.46	9.80E-05	0.0022
AL353597.1	-2.46	9.42E-03	0.0257
AC005674.2	-2.46	2.66E-03	0.0113
RNU4-24P	-2.46	4.55E-03	0.0158
WHAMM	-2.46	5.68E-05	0.0017
NLGN3	-2.46	1.45E-03	0.0079
NYAP2	-2.46	1.46E-02	0.0351
RNU4-51P	-2.46	1.01E-02	0.0269
INSYN1	-2.46	3.42E-03	0.0132
ENST00000389243	-2.46	1.35E-03	0.0076
CA14	-2.47	1.26E-02	0.0316
TRGV10	-2.47	8.75E-04	0.0060
PTGER4P2-CDK2AP2P2	-2.47	8.70E-04	0.0060
SCN3A	-2.47	2.33E-03	0.0104
PAPPA-AS1	-2.47	2.46E-03	0.0108
ENST000002601971	-2.47	2.07E-05	0.0012
DSEL	-2.47	3.03E-04	0.0036
SLC7A11-AS1	-2.47	2.55E-03	0.0111
LINC00954	-2.47	3.88E-04	0.0040
AC008764.8	-2.47	5.72E-04	0.0048
EVI2B	-2.47	4.80E-05	0.0016
PSMD6-AS2	-2.47	9.88E-04	0.0064
S1PR5	-2.47	1.87E-02	0.0419
METTL7A	-2.47	1.78E-03	0.0089
WWTR1-IT1	-2.47	8.84E-03	0.0246
AC124016.2	-2.48	2.12E-03	0.0099
AC090673.1	-2.48	8.74E-03	0.0245
PLXND1	-2.48	1.05E-03	0.0066
AC122129.1	-2.48	1.48E-02	0.0354
AC006978.1	-2.48	6.01E-05	0.0018
AC091551.1	-2.48	3.44E-03	0.0132
AC244260.1	-2.48	5.56E-03	0.0181
SNORD116-24	-2.49	1.15E-02	0.0296
MME	-2.49	1.07E-03	0.0067
AC004492.1	-2.49	3.81E-03	0.0141
AC004492.2	-2.49	3.81E-03	0.0141

AQP3	-2.49	3.72E-04	0.0039
APBA1	-2.49	8.30E-03	0.0236
KLHDC1	-2.49	9.84E-05	0.0022
AC012507.2	-2.49	2.79E-03	0.0116
AC015982.1	-2.49	1.89E-03	0.0093
FBP1	-2.50	3.47E-03	0.0133
KIF19	-2.50	7.91E-03	0.0229
BLZF2P	-2.50	1.57E-02	0.0370
ABTB1	-2.50	1.09E-02	0.0285
LINC01505	-2.50	6.76E-03	0.0206
TET1	-2.50	3.28E-04	0.0037
TMEM191A	-2.50	2.31E-02	0.0486
RAD51-AS1	-2.50	9.67E-05	0.0022
RN7SL430P	-2.50	1.08E-02	0.0283
AL360182.1	-2.50	2.28E-04	0.0032
RF00012.23	-2.50	3.65E-03	0.0138
AC002128.1	-2.51	1.58E-03	0.0083
PLA2G4D	-2.51	2.51E-03	0.0110
GUCY1B2	-2.51	8.04E-03	0.0231
CCR6	-2.51	7.19E-05	0.0020
STRA6LP	-2.51	4.66E-03	0.0161
BTG1	-2.51	2.80E-05	0.0014
AL031429.2	-2.52	2.01E-03	0.0096
AC009118.3	-2.52	7.40E-04	0.0055
C5AR1	-2.52	1.03E-03	0.0066
SLC18A2	-2.52	1.02E-02	0.0272
AC091806.1	-2.52	1.26E-03	0.0073
ENST000003387970	-2.52	1.13E-02	0.0293
LINC00908	-2.52	4.00E-03	0.0146
ZMYM1	-2.52	1.44E-03	0.0079
ENST000002433492	-2.52	6.34E-04	0.0051
RN7SL326P	-2.52	1.35E-02	0.0332
PABPC1L2A	-2.52	8.51E-03	0.0240
CASP1P2	-2.52	3.04E-03	0.0122
LINC02263	-2.52	2.45E-03	0.0108
RNU6-638P	-2.53	1.51E-03	0.0081
ZNF815P	-2.53	1.13E-04	0.0024
PPP2R2B	-2.53	9.97E-05	0.0022
LINC01126	-2.53	2.90E-03	0.0119
SORL1	-2.54	4.07E-05	0.0016
AL591503.1	-2.54	3.13E-03	0.0125
PLXDC1	-2.54	9.53E-04	0.0063
AC233309.1	-2.54	3.49E-03	0.0133
AMN	-2.54	1.02E-02	0.0271
AC004233.4	-2.54	5.96E-04	0.0049
TMEM272	-2.55	2.67E-03	0.0113
FAM19A2	-2.55	5.44E-04	0.0046

TMEM229B	-2.55	4.11E-04	0.0041
PTGDS	-2.55	7.57E-03	0.0222
CCDC146	-2.55	5.90E-04	0.0049
STMN3	-2.55	1.49E-04	0.0026
OXNAD1	-2.55	3.17E-05	0.0014
AL662889.1	-2.55	1.98E-03	0.0095
AP003352.1	-2.56	1.61E-02	0.0376
AK5	-2.56	2.29E-03	0.0103
AC026355.2	-2.56	2.75E-03	0.0116
MTURN	-2.56	2.43E-04	0.0032
MEGF6	-2.57	2.94E-04	0.0035
AL133384.1	-2.57	1.50E-03	0.0081
AC139491.7	-2.57	8.87E-04	0.0060
AC103769.1	-2.57	3.58E-03	0.0136
AC069503.1	-2.57	7.33E-03	0.0217
SNORA61	-2.57	1.11E-02	0.0289
GBP3	-2.57	2.79E-04	0.0034
AFF3	-2.58	4.11E-03	0.0149
ENST000003468320	-2.58	8.09E-03	0.0232
CD180	-2.58	1.06E-02	0.0278
NBEA	-2.58	7.18E-04	0.0054
SPDYE16	-2.58	6.70E-03	0.0204
SLC9A9	-2.59	2.02E-04	0.0030
DPYD	-2.59	1.72E-05	0.0011
DISC1	-2.59	3.06E-04	0.0036
ENST00000480419	-2.59	3.78E-03	0.0141
AC019227.1	-2.59	2.04E-03	0.0096
LOXL2	-2.59	8.81E-03	0.0246
PTPRO	-2.59	2.40E-04	0.0032
ENST00000636259	-2.59	7.59E-03	0.0223
HLA-DRA	-2.59	1.16E-03	0.0070
LY9	-2.59	1.25E-04	0.0025
WHAMMP2	-2.60	3.20E-04	0.0037
OR2M4	-2.60	2.19E-04	0.0031
ENST000003168511	-2.60	6.20E-03	0.0194
RFTN2	-2.60	6.44E-03	0.0199
ITPKB-IT1	-2.60	2.53E-04	0.0033
TXK	-2.61	3.70E-04	0.0039
IQCN	-2.61	2.74E-04	0.0034
ENST000002576261	-2.61	1.48E-03	0.0080
MCOLN3	-2.61	3.56E-04	0.0038
AP000977.1	-2.61	3.59E-04	0.0038
PLAC8	-2.61	1.59E-03	0.0084
AC090198.1	-2.61	1.09E-04	0.0023
PRKN	-2.61	3.72E-04	0.0039
AL162377.1	-2.61	7.90E-03	0.0229
CRAT37	-2.61	1.04E-03	0.0066

AC087878.1	-2.61	6.04E-03	0.0191
MRPL37P1	-2.62	3.19E-04	0.0037
TRGC1	-2.62	1.47E-03	0.0080
AC005154.2	-2.62	3.99E-04	0.0040
EEF1DP7	-2.62	1.85E-03	0.0091
POU5F1B	-2.62	4.91E-04	0.0044
ENST000003074413	-2.62	1.35E-03	0.0076
CLEC2B	-2.62	1.28E-03	0.0074
SOX4	-2.62	3.62E-03	0.0137
KRT73-AS1	-2.62	9.33E-03	0.0255
EMBP1	-2.63	1.41E-04	0.0026
ZNF483	-2.63	1.59E-03	0.0084
ITPKB	-2.63	1.36E-04	0.0025
AC004067.1	-2.63	2.55E-04	0.0033
ENST000003515780	-2.63	1.73E-04	0.0028
LTK	-2.63	6.40E-04	0.0051
C4orf50	-2.63	2.88E-03	0.0118
TDRD12	-2.64	2.16E-03	0.0099
LINC02328	-2.64	1.14E-04	0.0024
TMEM40	-2.64	8.77E-03	0.0245
AL138756.1	-2.64	2.87E-03	0.0118
AUTS2	-2.64	1.11E-03	0.0068
SPATA41	-2.64	1.10E-02	0.0286
ALS2CL	-2.64	1.99E-04	0.0030
AC018442.1	-2.64	5.08E-03	0.0170
TMEM200A	-2.64	2.60E-04	0.0033
Z98755.1	-2.64	1.10E-03	0.0068
AC007529.1	-2.65	2.35E-03	0.0105
AC007342.1	-2.65	7.18E-03	0.0214
AP005263.1	-2.65	1.11E-03	0.0068
CNTN2	-2.65	3.37E-03	0.0131
AL034550.2	-2.65	2.04E-03	0.0096
AC008549.2	-2.65	3.85E-04	0.0039
RNF113B	-2.65	3.52E-04	0.0038
HMGB3P4	-2.66	1.40E-03	0.0078
SLC40A1	-2.66	3.76E-03	0.0140
AC122718.2	-2.66	4.57E-03	0.0159
APBB1	-2.66	5.41E-05	0.0017
AC005291.1	-2.66	9.60E-03	0.0261
TAB3-AS1	-2.66	3.60E-03	0.0136
IL7R	-2.66	1.02E-05	0.0010
AL359962.2	-2.66	3.87E-04	0.0040
AL645933.2	-2.66	1.65E-04	0.0028
CERS3	-2.67	2.92E-03	0.0119
AC092650.1	-2.67	1.96E-03	0.0094
AC006064.5	-2.67	3.91E-05	0.0016
AC090833.1	-2.67	2.05E-04	0.0030

AC093423.2	-2.68	3.19E-04	0.0037
TCF7	-2.68	1.73E-04	0.0028
KANSL1-AS1	-2.68	2.32E-03	0.0104
EEPD1	-2.69	1.00E-02	0.0267
RFX4	-2.69	8.37E-03	0.0238
HGD	-2.69	1.30E-03	0.0074
AC005842.1	-2.69	4.41E-03	0.0155
GCKR	-2.69	4.39E-03	0.0155
PARP8	-2.69	2.51E-05	0.0013
LECT2	-2.69	1.14E-03	0.0069
ZNF890P	-2.70	3.66E-04	0.0039
LINC01242	-2.70	7.03E-03	0.0211
AC010983.1	-2.70	9.78E-03	0.0264
SDR42E2	-2.70	3.72E-04	0.0039
MHENCR	-2.70	8.64E-05	0.0021
AL365361.1	-2.71	1.74E-05	0.0011
AXIN2	-2.71	5.89E-05	0.0018
ENST000002601841	-2.72	3.18E-04	0.0037
DNAJB1	-2.72	2.54E-05	0.0013
ANTXR2	-2.73	1.95E-05	0.0012
LINCR-0001	-2.73	1.04E-02	0.0275
GDF5OS	-2.73	7.58E-03	0.0222
PRNCR1	-2.73	1.10E-04	0.0023
LINC00266-4P	-2.73	5.51E-03	0.0180
AL669831.4	-2.73	9.51E-05	0.0022
RN7SL801P	-2.74	4.16E-03	0.0150
TAS2R63P	-2.74	2.32E-03	0.0104
AP000936.3	-2.74	2.28E-02	0.0482
AL357033.3	-2.75	1.39E-03	0.0077
AL355103.1	-2.75	2.11E-02	0.0457
COX6CP1	-2.75	1.44E-03	0.0079
AC090092.1	-2.76	7.41E-03	0.0219
FRG1FP	-2.76	5.60E-03	0.0182
ERP27	-2.77	5.16E-04	0.0045
IPCEF1	-2.77	5.32E-05	0.0017
CARD16	-2.77	5.12E-05	0.0017
ZNF652	-2.77	5.35E-05	0.0017
PLBD1	-2.77	1.21E-02	0.0307
AC011939.3	-2.77	1.37E-02	0.0334
SNTN	-2.77	4.32E-03	0.0153
BOC	-2.78	7.44E-04	0.0055
RN7SL749P	-2.78	4.17E-03	0.0150
CR2	-2.79	1.63E-02	0.0378
MS4A6A	-2.79	1.02E-03	0.0065
AC027045.3	-2.80	3.37E-03	0.0131
AC093726.2	-2.80	3.37E-03	0.0131
AL391834.2	-2.80	2.09E-03	0.0098

AC022140.1	-2.80	6.03E-03	0.0191
LINC01395	-2.81	5.15E-03	0.0172
BEND2	-2.81	1.67E-03	0.0086
MSRB3	-2.82	9.60E-04	0.0063
MLXIPL	-2.82	4.33E-03	0.0154
KRT26	-2.82	1.04E-04	0.0023
AC103724.4	-2.82	1.25E-03	0.0073
AC058823.1	-2.82	2.00E-02	0.0438
ARHGAP15	-2.82	2.27E-05	0.0012
MIR3679	-2.82	5.23E-03	0.0174
LINC01725	-2.82	1.14E-03	0.0069
AMIGO1	-2.82	3.38E-04	0.0037
AC109454.2	-2.83	7.99E-04	0.0057
ADAM28	-2.83	2.02E-04	0.0030
LPAR6	-2.83	3.51E-04	0.0038
GRK5-IT1	-2.83	4.50E-04	0.0042
TBX4	-2.83	5.18E-03	0.0173
RF00019.470	-2.84	4.74E-04	0.0044
GCNT4	-2.84	2.77E-05	0.0014
CTSF	-2.84	1.29E-04	0.0025
EEF1A1P3	-2.84	4.23E-04	0.0041
LIME1	-2.84	1.03E-02	0.0273
PIK3R5	-2.84	1.99E-04	0.0030
LINC00987	-2.85	2.72E-03	0.0115
CNNM3-DT	-2.85	9.87E-04	0.0064
TENT5A	-2.85	1.96E-03	0.0094
AC016590.3	-2.85	6.50E-05	0.0019
ITGA4	-2.85	1.27E-05	0.0010
TCF4	-2.85	3.53E-04	0.0038
AC005197.1	-2.86	4.28E-03	0.0153
LPAL2	-2.86	2.15E-04	0.0031
AC013264.1	-2.86	8.15E-03	0.0233
AC008686.1	-2.86	1.26E-02	0.0315
IGHM	-2.86	6.16E-03	0.0193
AL354798.1	-2.86	5.15E-03	0.0172
TLE1	-2.87	9.82E-03	0.0264
AC125437.1	-2.87	3.30E-04	0.0037
AC105150.1	-2.87	1.24E-02	0.0311
IZUMO1	-2.87	2.43E-04	0.0032
ZBTB20	-2.88	7.72E-05	0.0020
LILRB2	-2.88	3.87E-03	0.0143
PRDM11	-2.88	2.29E-03	0.0103
RANBP2	-2.89	6.35E-05	0.0019
GABARAPL1	-2.89	2.02E-05	0.0012
SELENOP	-2.89	5.94E-03	0.0189
AC135782.3	-2.89	1.70E-03	0.0087
CDC14A	-2.90	1.21E-05	0.0010

RASA3-IT1	-2.90	3.13E-03	0.0125
AC016405.1	-2.90	2.63E-04	0.0033
LINC02273	-2.90	1.62E-04	0.0027
KLRC4-KLRK1	-2.90	1.33E-02	0.0329
AC093865.1	-2.91	1.57E-03	0.0083
GOLGA5P1	-2.91	7.70E-04	0.0056
AC103724.3	-2.91	2.69E-03	0.0114
LILRB4	-2.92	4.81E-03	0.0164
AP000787.1	-2.92	9.80E-04	0.0064
ENST000000824681	-2.93	9.15E-05	0.0022
AC007342.5	-2.93	7.95E-03	0.0230
PCAT1	-2.93	2.08E-04	0.0030
AL513534.1	-2.94	2.28E-04	0.0032
PTGDR2	-2.94	3.76E-03	0.0140
PGBD5	-2.95	3.96E-03	0.0145
RCBTB2	-2.95	1.40E-03	0.0078
FABP7P1	-2.95	4.03E-03	0.0146
DIP2C	-2.95	4.10E-04	0.0041
LINC01726	-2.96	4.88E-03	0.0166
KDELC1P1	-2.96	2.01E-02	0.0441
RAB27B	-2.96	1.63E-04	0.0027
PDZD2	-2.96	5.10E-03	0.0171
RN7SL824P	-2.96	3.43E-03	0.0132
AC110921.1	-2.97	1.01E-02	0.0269
AC159540.2	-2.97	3.36E-05	0.0015
FAM133DP	-2.97	3.31E-04	0.0037
AL356022.1	-2.97	2.39E-03	0.0106
MYBL1	-2.97	1.19E-05	0.0010
MIR5690	-2.97	9.62E-05	0.0022
TENT5C	-2.97	3.29E-05	0.0015
AC134508.1	-2.97	2.40E-04	0.0032
AL589993.1	-2.97	4.23E-05	0.0016
DRICH1	-2.98	2.52E-03	0.0110
RIPOR2	-2.98	3.10E-05	0.0014
CCT4P2	-2.98	7.13E-04	0.0054
TOX	-2.99	2.67E-04	0.0034
CDHR3	-2.99	5.22E-05	0.0017
TMEM119	-3.00	2.26E-03	0.0102
TCP11L2	-3.00	1.11E-05	0.0010
AL357033.4	-3.00	2.57E-04	0.0033
RNU6-1010P	-3.01	4.67E-03	0.0161
BCAS1	-3.01	8.49E-04	0.0059
AC118658.1	-3.02	1.30E-02	0.0322
ENST000003046210	-3.02	4.57E-05	0.0016
AL360270.1	-3.02	1.32E-04	0.0025
ENST000003023162	-3.03	6.82E-03	0.0207
ATXN7	-3.03	3.58E-05	0.0015

PLA2G2D	-3.03	4.70E-03	0.0162
AC104964.3	-3.03	1.19E-02	0.0302
TIPARP-AS1	-3.03	6.29E-03	0.0196
EPHA4	-3.04	1.35E-04	0.0025
SERPINF1	-3.05	3.01E-04	0.0035
AC008115.3	-3.05	9.96E-05	0.0022
IGHV3-36	-3.05	1.27E-03	0.0073
AC005534.1	-3.05	7.77E-04	0.0056
HPCAL4	-3.05	4.41E-05	0.0016
BAGE2	-3.06	5.43E-04	0.0046
GAPT	-3.06	6.74E-03	0.0205
ENST000002641071	-3.06	4.98E-05	0.0017
AL159972.1	-3.06	3.01E-03	0.0122
LINC02033	-3.07	1.46E-04	0.0026
AC036108.3	-3.07	4.86E-03	0.0165
RASSF6	-3.07	4.13E-04	0.0041
AL359792.1	-3.07	4.57E-03	0.0159
SLCO3A1	-3.08	7.31E-04	0.0055
MAP2K6	-3.09	1.15E-04	0.0024
AL645568.2	-3.09	9.05E-03	0.0251
AC122710.2	-3.10	1.68E-04	0.0028
NDUFV2-AS1	-3.10	4.67E-04	0.0043
UNC45B	-3.10	7.74E-04	0.0056
SYT2	-3.10	2.92E-03	0.0119
CX3CR1	-3.10	3.66E-03	0.0138
LINC01876	-3.11	1.74E-02	0.0398
KRT73	-3.11	6.91E-04	0.0053
VPS37B	-3.11	2.05E-04	0.0030
GPRASP1	-3.11	1.23E-05	0.0010
AC068722.2	-3.12	3.71E-03	0.0139
AC067863.1	-3.12	3.14E-03	0.0125
AC022916.1	-3.13	1.49E-04	0.0026
SLC14A1	-3.13	1.07E-03	0.0067
AC025884.1	-3.14	6.92E-03	0.0209
NR1D2	-3.14	1.64E-05	0.0011
HSPE1P28	-3.14	2.51E-03	0.0109
AC024267.4	-3.14	2.80E-03	0.0116
AL359885.1	-3.14	1.58E-02	0.0371
LINC01934	-3.15	8.99E-04	0.0061
MIR3611	-3.15	4.75E-03	0.0163
KLRG1	-3.15	1.85E-04	0.0029
MAP1LC3B2	-3.15	2.43E-03	0.0107
SPDYA	-3.15	1.25E-05	0.0010
LINC00921	-3.15	1.10E-04	0.0023
TBC1D9	-3.15	3.14E-03	0.0125
GZMA	-3.16	1.60E-03	0.0084
AC009812.1	-3.16	2.04E-02	0.0445

THRIL	-3.16	2.15E-03	0.0099
AC026979.2	-3.17	1.23E-04	0.0025
SEC22B4P	-3.17	3.60E-03	0.0136
ARL4C	-3.17	9.33E-05	0.0022
PI16	-3.17	4.45E-04	0.0042
RF00019.708	-3.18	1.27E-04	0.0025
AC017035.1	-3.18	8.92E-05	0.0021
AC022079.1	-3.18	1.75E-04	0.0028
AL109811.2	-3.19	2.24E-04	0.0031
TSC22D1-AS1	-3.19	1.58E-02	0.0371
C11orf88	-3.19	2.68E-03	0.0114
GLIPR1	-3.20	4.46E-05	0.0016
TLR3	-3.20	9.89E-05	0.0022
FCN1	-3.20	1.63E-03	0.0085
HKDC1	-3.20	1.10E-02	0.0287
MIR8485	-3.22	5.90E-03	0.0188
TTN	-3.22	6.70E-05	0.0019
LINC02119	-3.22	1.36E-03	0.0076
NR4A2	-3.23	1.24E-03	0.0073
LIMCH1	-3.23	1.35E-03	0.0076
NR3C2	-3.24	6.41E-05	0.0019
AC007064.2	-3.24	2.89E-03	0.0119
TNRC6C-AS1	-3.24	8.14E-05	0.0021
AMD1P4	-3.24	7.73E-04	0.0056
AL158210.1	-3.25	1.77E-03	0.0089
AC092375.2	-3.25	1.21E-04	0.0024
AC241988.2	-3.25	1.21E-04	0.0024
TKTL1	-3.25	4.13E-03	0.0149
KRT18P15	-3.26	1.01E-02	0.0269
ATM	-3.26	3.48E-06	0.0009
ENST000003510710	-3.26	1.70E-03	0.0087
AC021242.2	-3.26	2.31E-03	0.0104
AL627402.1	-3.27	3.21E-03	0.0127
LINC01891	-3.27	1.93E-03	0.0094
AL590426.2	-3.28	2.37E-04	0.0032
ADAMTS10	-3.28	1.24E-03	0.0073
AC103703.1	-3.28	1.31E-03	0.0075
AC068134.3	-3.28	1.78E-03	0.0089
CHRNA2	-3.28	1.19E-03	0.0071
AL357060.1	-3.29	1.67E-05	0.0011
RGS1	-3.29	1.27E-04	0.0025
RAB37	-3.29	6.62E-05	0.0019
IFNG-AS1	-3.29	1.18E-04	0.0024
RGS2	-3.29	1.41E-05	0.0011
ENST000002762040	-3.31	4.29E-04	0.0041
AL591895.1	-3.31	2.53E-04	0.0033
LINC02361	-3.31	2.21E-04	0.0031

CLCF1	-3.32	1.65E-03	0.0086
DGKB	-3.32	3.47E-03	0.0133
HAVCR1	-3.33	4.27E-03	0.0152
CDC42BPG	-3.33	5.09E-04	0.0045
CHN2	-3.34	1.62E-03	0.0085
SCML4	-3.35	9.43E-05	0.0022
CCDC141	-3.35	2.88E-04	0.0035
AC100802.1	-3.36	3.01E-04	0.0035
PTGER2	-3.37	1.96E-05	0.0012
FAM221B	-3.37	2.99E-04	0.0035
STMN1P1	-3.38	1.95E-03	0.0094
MXD4	-3.38	5.99E-05	0.0018
AL137222.1	-3.38	1.51E-02	0.0358
HPGDS	-3.38	1.51E-02	0.0358
PPIAP53	-3.38	1.85E-03	0.0092
LINC01801	-3.39	2.57E-04	0.0033
PLXNA4	-3.40	3.93E-04	0.0040
TLR8	-3.40	2.31E-02	0.0486
ENST000002735501	-3.40	1.36E-03	0.0076
LINC02295	-3.41	2.31E-02	0.0487
B3GALT2	-3.42	9.36E-03	0.0256
SPRY3	-3.42	8.81E-05	0.0021
SLC44A3-AS1	-3.43	1.49E-03	0.0081
EFHC2	-3.43	1.54E-04	0.0027
ANTXRLP1	-3.43	1.39E-03	0.0077
ENST000002406180	-3.44	1.96E-02	0.0433
AC016582.2	-3.44	1.07E-03	0.0067
SUSD4	-3.44	4.80E-03	0.0164
AL050343.1	-3.44	2.53E-03	0.0110
AL139415.1	-3.46	4.83E-03	0.0165
C1orf162	-3.46	5.32E-03	0.0175
C12orf40	-3.46	1.49E-02	0.0356
GOLGA7B	-3.46	2.20E-04	0.0031
SEMA3C	-3.46	3.13E-03	0.0125
FRAT1	-3.47	3.09E-05	0.0014
IGHV3-33	-3.47	1.68E-03	0.0086
IL7	-3.47	2.07E-03	0.0097
RNF157-AS1	-3.47	2.10E-02	0.0454
SYK	-3.49	6.75E-04	0.0052
RHBDL2	-3.49	2.07E-02	0.0450
VIPR1	-3.49	9.26E-03	0.0254
ENST000003165490	-3.50	1.53E-05	0.0011
HNF4G	-3.50	2.13E-02	0.0459
IGHV3-7	-3.51	1.12E-03	0.0068
AC069213.1	-3.52	8.93E-04	0.0061
Z92544.2	-3.52	1.25E-02	0.0314
CELA1	-3.52	1.98E-02	0.0436

RNU6-1321P	-3.52	1.98E-02	0.0436
TLR1	-3.52	2.02E-04	0.0030
ITGA6	-3.54	4.70E-05	0.0016
FRMPD3	-3.54	1.98E-02	0.0436
SULF2	-3.54	4.85E-03	0.0165
FGF9	-3.54	3.93E-04	0.0040
CCR5	-3.55	3.08E-04	0.0036
MEIS1	-3.55	1.18E-03	0.0071
ADAMTS17	-3.55	2.74E-04	0.0034
ZFP36L2	-3.55	1.50E-05	0.0011
AC027682.4	-3.56	1.89E-02	0.0422
SEMA3G	-3.56	2.82E-03	0.0117
AC008878.3	-3.56	5.04E-03	0.0169
AL021920.2	-3.56	2.17E-02	0.0465
AC119396.1	-3.56	1.55E-05	0.0011
AC011379.2	-3.58	1.12E-02	0.0291
AL158163.2	-3.58	1.10E-03	0.0068
VCAN	-3.58	1.08E-03	0.0067
FAM13A	-3.59	3.38E-03	0.0131
ENST000003322460	-3.60	4.22E-05	0.0016
OLFM5P	-3.60	1.85E-02	0.0415
SLC22A23	-3.60	7.59E-04	0.0055
DPEP2	-3.61	1.29E-03	0.0074
TXNIP	-3.61	3.50E-05	0.0015
FAM13A-AS1	-3.62	4.56E-05	0.0016
DIP2A-IT1	-3.63	1.02E-03	0.0065
FOS	-3.63	4.90E-05	0.0017
MYO16	-3.64	7.05E-04	0.0054
SUMO3	-3.65	6.36E-03	0.0197
FCRL6	-3.65	1.01E-02	0.0270
OCM	-3.66	3.38E-04	0.0037
ZNF540	-3.66	4.68E-05	0.0016
ENST000002993380	-3.67	9.95E-03	0.0266
TRBV23-1	-3.68	1.23E-04	0.0025
ADHFE1	-3.68	5.53E-03	0.0180
AC068580.4	-3.69	9.72E-03	0.0263
AL354813.1	-3.69	9.72E-03	0.0263
PLCL1	-3.69	9.87E-06	0.0010
SAMD3	-3.69	1.49E-05	0.0011
MIR3136	-3.70	9.49E-03	0.0259
PIK3R1	-3.70	7.19E-06	0.0010
HNRNPA1P70	-3.70	6.52E-04	0.0051
ACER1	-3.71	1.66E-02	0.0384
AL158163.1	-3.71	6.32E-05	0.0019
SESN3	-3.72	1.57E-04	0.0027
CLEC4C	-3.72	9.17E-03	0.0253
AC025171.5	-3.72	1.71E-02	0.0393

AC106882.1	-3.73	9.19E-03	0.0253
SIK1	-3.74	2.14E-02	0.0461
TRAT1	-3.74	4.41E-04	0.0042
IGLV3-19	-3.75	1.97E-03	0.0095
MEF2C	-3.76	2.02E-04	0.0030
AC024084.1	-3.76	9.97E-04	0.0064
AC131391.1	-3.76	1.68E-03	0.0086
ENST00000527515	-3.77	1.47E-02	0.0353
NHSL2	-3.77	3.18E-05	0.0014
ANXA1	-3.77	4.29E-06	0.0009
INSL6	-3.77	9.75E-03	0.0263
FGR	-3.77	3.31E-04	0.0037
ZIK1P1	-3.78	8.80E-03	0.0246
AL109767.1	-3.78	1.89E-04	0.0029
ACE	-3.78	1.61E-04	0.0027
WDR86-AS1	-3.79	1.98E-03	0.0095
KLF3	-3.79	3.78E-06	0.0009
RN7SL517P	-3.80	3.89E-05	0.0016
PPIAP72	-3.80	2.09E-02	0.0453
RF00019.18	-3.80	1.48E-02	0.0355
ZBP1	-3.81	4.86E-05	0.0017
TSPAN32	-3.81	1.43E-05	0.0011
AC008565.1	-3.82	9.47E-03	0.0258
FLT3	-3.82	2.35E-02	0.0493
AP002365.1	-3.82	8.12E-03	0.0233
EPGN	-3.83	1.05E-03	0.0066
AL365203.2	-3.84	8.87E-05	0.0021
AL353662.1	-3.84	2.08E-02	0.0451
COBLL1	-3.84	2.97E-04	0.0035
ENST000003340031	-3.84	1.43E-05	0.0011
TNFRSF19	-3.85	1.44E-02	0.0348
AC074254.1	-3.86	7.54E-03	0.0222
PIK3IP1	-3.86	3.68E-05	0.0015
AC233976.1	-3.86	5.42E-05	0.0017
TSPAN18	-3.88	6.26E-05	0.0018
FLJ40194	-3.90	7.36E-03	0.0218
AC139256.1	-3.90	1.85E-05	0.0011
AC068594.2	-3.90	1.38E-02	0.0336
AC068594.4	-3.90	1.38E-02	0.0336
RASA2-IT1	-3.90	2.07E-04	0.0030
CRIP2	-3.90	1.17E-03	0.0070
CYP2E1	-3.91	7.11E-03	0.0213
RN7SL653P	-3.92	7.28E-04	0.0055
IGLV1-51	-3.92	5.56E-03	0.0181
AC012557.2	-3.92	1.34E-02	0.0329
RF00019.281	-3.93	7.05E-03	0.0212
ENST000003177163	-3.93	1.30E-02	0.0322

RAB31	-3.93	2.50E-04	0.0033
NTN4	-3.94	1.21E-02	0.0308
AC055748.1	-3.94	1.86E-04	0.0029
ENST000003081910	-3.95	7.58E-03	0.0222
AC090948.3	-3.96	3.67E-03	0.0138
AL121845.2	-3.97	9.79E-05	0.0022
IGKV3-20	-3.98	1.52E-03	0.0082
AC008906.1	-3.98	3.24E-04	0.0037
ACSL6	-3.98	6.57E-06	0.0010
AC007952.5	-3.99	1.30E-02	0.0323
LINC01336	-3.99	2.36E-05	0.0012
ENST00000588041	-4.01	8.03E-05	0.0020
IGLV3-25	-4.02	2.23E-03	0.0101
AP000254.1	-4.02	2.01E-02	0.0441
CUBN	-4.02	9.58E-05	0.0022
AC090948.1	-4.02	1.34E-05	0.0011
AC092652.3	-4.02	1.88E-05	0.0011
FAM30A	-4.03	1.32E-03	0.0075
AC127526.1	-4.03	5.86E-03	0.0187
CCAT2	-4.04	5.94E-03	0.0189
FGF5	-4.04	5.94E-03	0.0189
KCNA2	-4.05	2.21E-04	0.0031
AC244034.2	-4.08	2.06E-02	0.0449
IGKV1-5	-4.08	3.07E-03	0.0123
IGLV1-47	-4.09	4.44E-04	0.0042
MMP28	-4.11	5.31E-03	0.0175
C16orf74	-4.12	1.29E-02	0.0321
AL356273.2	-4.12	1.49E-02	0.0356
IRS2	-4.12	5.43E-06	0.0009
ARMH2	-4.13	2.79E-04	0.0034
AC009831.3	-4.13	4.65E-05	0.0016
AC016957.2	-4.14	1.49E-04	0.0026
IGKV4-1	-4.15	1.56E-03	0.0083
AC091180.2	-4.16	4.91E-03	0.0166
FCGR2C	-4.18	4.78E-03	0.0164
DAB1	-4.19	9.95E-03	0.0266
AC090948.2	-4.20	2.53E-03	0.0110
MIR548C	-4.20	6.22E-04	0.0050
PSMD10P1	-4.21	1.39E-02	0.0337
AC006195.1	-4.21	4.66E-03	0.0161
ENST000003219350	-4.21	8.99E-03	0.0249
CSGALNACT1	-4.21	8.30E-06	0.0010
BAIAP3	-4.21	6.53E-03	0.0201
ST13P15	-4.22	4.63E-03	0.0160
AL355581.1	-4.23	7.04E-05	0.0019
IGHV3-74	-4.23	8.31E-03	0.0236
LINC00587	-4.23	8.31E-03	0.0236

AL022328.3	-4.24	9.31E-03	0.0255
AL049779.1	-4.25	4.39E-03	0.0155
PDGFD	-4.25	5.25E-03	0.0174
ENST000002435830	-4.28	9.23E-03	0.0254
TENM1	-4.28	5.72E-05	0.0017
IGHV3-23	-4.29	2.51E-05	0.0013
FAM238A	-4.31	1.21E-02	0.0306
IGFBP3	-4.32	1.24E-05	0.0010
ENST000002932083	-4.32	8.21E-03	0.0234
ANKRD30BP2	-4.35	4.45E-03	0.0156
ENST000002764401	-4.35	4.45E-03	0.0156
AP003717.1	-4.37	2.76E-04	0.0034
RN7SL178P	-4.39	1.08E-02	0.0283
RGS18	-4.39	2.44E-03	0.0107
IGHV5-51	-4.40	8.84E-03	0.0246
PKIA-AS1	-4.41	2.65E-03	0.0113
CXCR4	-4.41	1.06E-06	0.0008
LINC01888	-4.41	4.31E-03	0.0153
IGKV3-11	-4.42	5.78E-03	0.0186
AL135924.2	-4.44	6.68E-03	0.0204
HIGD1AP14	-4.44	7.16E-03	0.0214
ADA2	-4.45	3.84E-06	0.0009
LINC02390	-4.45	1.51E-03	0.0081
NUDT16L1	-4.46	1.57E-02	0.0370
AL117381.1	-4.46	3.24E-03	0.0127
LINC01259	-4.47	3.21E-03	0.0127
ARRDC5	-4.48	2.87E-05	0.0014
CARD17	-4.49	1.49E-04	0.0026
LYZ	-4.50	1.07E-05	0.0010
ENST000003429920	-4.53	1.78E-05	0.0011
SNED1	-4.53	8.21E-03	0.0234
CEACAM4	-4.54	2.21E-03	0.0101
ZNF521	-4.55	3.05E-03	0.0123
TRIB2	-4.56	1.51E-06	0.0008
ZDHHC20P1	-4.56	2.16E-03	0.0099
FREM1	-4.56	1.68E-02	0.0387
S100A9	-4.59	3.22E-03	0.0127
EAF1-AS1	-4.60	1.31E-02	0.0324
KLF17	-4.61	2.13E-03	0.0099
TSC22D3	-4.62	1.12E-06	0.0008
UTS2	-4.63	5.61E-03	0.0182
PPM1H	-4.63	2.10E-03	0.0098
IL12A	-4.64	2.66E-03	0.0113
IGLC3	-4.64	6.83E-04	0.0053
SIK1B	-4.66	2.34E-04	0.0032
BX248123.1	-4.66	1.09E-04	0.0023
RAP1GAP2	-4.66	2.37E-04	0.0032

IGLC2	-4.67	1.74E-03	0.0088
DUSP1	-4.67	8.90E-06	0.0010
NCR1	-4.67	2.00E-03	0.0095
MNDA	-4.67	3.37E-03	0.0131
IGLV2-8	-4.68	9.84E-03	0.0265
CACNB2	-4.68	1.99E-03	0.0095
ESR1	-4.68	7.63E-05	0.0020
ENST000003195841	-4.70	1.76E-03	0.0089
AC112693.1	-4.71	2.49E-03	0.0109
AC025171.4	-4.71	1.78E-03	0.0089
AC079015.1	-4.72	1.48E-04	0.0026
CTD1I18.1	-4.73	2.06E-03	0.0097
FGL2	-4.74	1.24E-03	0.0073
AC046136.1	-4.75	1.62E-03	0.0085
ENC1	-4.75	8.27E-04	0.0058
RNU6-1152P	-4.75	7.63E-03	0.0223
WNT10B	-4.76	1.66E-02	0.0385
CCR2	-4.76	2.04E-04	0.0030
AP005242.3	-4.76	1.70E-03	0.0087
PTGDR	-4.76	2.20E-03	0.0101
CR1	-4.77	1.02E-03	0.0065
GPA33	-4.77	2.55E-03	0.0111
AP006284.1	-4.77	1.87E-03	0.0092
C12orf42	-4.78	6.52E-06	0.0010
GP5	-4.79	8.78E-03	0.0245
IGHA1	-4.79	2.48E-04	0.0032
IGKC	-4.80	8.46E-04	0.0059
IGLV1-40	-4.80	1.54E-02	0.0365
AC007349.3	-4.81	1.63E-03	0.0085
CCR3	-4.81	1.63E-03	0.0085
TTC39C-AS1	-4.82	1.85E-04	0.0029
IGLV1-44	-4.83	2.09E-03	0.0098
MS4A1	-4.83	4.37E-03	0.0155
CACNA1I	-4.85	1.48E-05	0.0011
IGKV1-9	-4.85	1.40E-03	0.0078
LINC00565	-4.86	1.57E-02	0.0370
IGKV2-24	-4.87	7.42E-03	0.0219
ENST00000542310	-4.89	1.27E-03	0.0074
AC009041.1	-4.90	1.42E-03	0.0078
RPL21P75	-4.91	1.34E-03	0.0076
WNT7A	-4.91	1.07E-03	0.0067
ENST000001946721	-4.91	9.23E-03	0.0254
ENST000003399980	-4.92	1.20E-03	0.0071
ENST000003241720	-4.94	1.38E-03	0.0077
DSC1	-4.94	1.95E-04	0.0030
ENST000002861221	-4.96	1.13E-03	0.0069
IGLC1	-4.98	2.03E-05	0.0012

IGHG2	-4.98	1.74E-02	0.0398
AC100827.3	-4.99	1.10E-03	0.0068
COPS8P2	-5.03	1.23E-03	0.0072
IGKV1-33	-5.03	1.69E-02	0.0389
HSD17B13	-5.03	1.19E-03	0.0071
BORCS7-ASMT	-5.03	1.10E-03	0.0068
SNRK-AS1	-5.04	1.50E-03	0.0081
ENST000002646900	-5.05	2.14E-03	0.0099
RF00019.201	-5.06	1.31E-03	0.0075
AC107626.1	-5.09	1.04E-03	0.0066
ISM1	-5.09	1.54E-04	0.0027
MPO	-5.12	1.78E-03	0.0089
VSIG1	-5.13	1.65E-05	0.0011
IGHV3-21	-5.16	3.96E-03	0.0145
AL163932.1	-5.17	8.37E-04	0.0058
IGHV2-5	-5.17	1.13E-03	0.0069
ENST000003172040	-5.17	1.48E-03	0.0080
KLRB1	-5.18	3.06E-05	0.0014
ENST000003817950	-5.18	8.66E-04	0.0060
IGLV2-11	-5.19	6.57E-03	0.0202
PPBP	-5.20	6.67E-03	0.0204
PDLIM1	-5.21	1.25E-03	0.0073
LINC00544	-5.21	1.67E-03	0.0086
IGHA2	-5.22	6.12E-04	0.0050
ENST000003042672	-5.22	8.58E-04	0.0059
EDAR	-5.24	1.88E-04	0.0029
ENST00000638176	-5.24	1.31E-03	0.0075
ENST000003484950	-5.24	9.94E-04	0.0064
KRT72	-5.27	1.69E-03	0.0087
COL24A1	-5.27	7.60E-04	0.0056
AC245519.2	-5.33	1.10E-03	0.0068
ALDH1A1	-5.35	8.17E-04	0.0058
JCHAIN	-5.36	6.69E-04	0.0052
AC146335.1	-5.37	8.77E-04	0.0060
TMEM221	-5.37	2.44E-03	0.0107
ENST000003142561	-5.37	5.73E-04	0.0048
KRT2	-5.39	5.95E-04	0.0049
GSAP	-5.40	7.62E-05	0.0020
IGLV3-21	-5.40	1.06E-02	0.0279
ENST000003252391	-5.40	7.17E-03	0.0214
AP003117.1	-5.40	1.05E-03	0.0066
IGLV3-1	-5.41	1.57E-03	0.0083
MPEG1	-5.43	2.73E-04	0.0034
FCER1A	-5.44	1.02E-04	0.0023
IGLV2-14	-5.48	1.66E-04	0.0028
IGHV3-15	-5.48	4.60E-03	0.0160
LINC00861	-5.48	1.35E-06	0.0008

FXYD7	-5.48	4.79E-04	0.0044
IGKV1D-39	-5.50	1.05E-02	0.0277
BMS1P17	-5.50	4.82E-04	0.0044
SDK2	-5.51	8.04E-04	0.0057
VDAC2P2	-5.51	2.24E-03	0.0102
AC131210.1	-5.55	4.27E-04	0.0041
ENST000003149700	-5.56	6.39E-03	0.0198
IGHV3-49	-5.56	9.29E-04	0.0062
ENST000002632051	-5.57	8.46E-04	0.0059
WDR64	-5.60	4.92E-04	0.0044
KRT17P8	-5.62	6.02E-03	0.0190
LINC00278	-5.64	1.88E-02	0.0421
IGHG1	-5.67	4.84E-04	0.0044
MUC20-OT1-876	-5.67	6.53E-03	0.0201
AC078927.1	-5.68	6.19E-04	0.0050
ENST000002965181	-5.70	3.38E-04	0.0037
AC131160.1	-5.71	5.37E-04	0.0046
TGFBI	-5.72	7.83E-04	0.0056
RASGRP2	-5.73	5.71E-06	0.0009
ENST000003103972	-5.75	3.77E-04	0.0039
ENST000003123521	-5.75	3.77E-04	0.0039
AREG	-5.75	1.25E-05	0.0010
IGHV4-59	-5.75	7.78E-04	0.0056
ENST000002740561	-5.76	3.26E-04	0.0037
IGHV3-11	-5.77	4.08E-04	0.0041
ENST000003540641	-5.85	3.27E-04	0.0037
ENST000003173611	-5.86	5.10E-04	0.0045
GTSCR1	-5.86	4.12E-04	0.0041
CCDC65	-5.86	1.02E-03	0.0065
IGLV2-23	-5.86	3.06E-03	0.0123
ENST000002632850	-5.87	2.31E-02	0.0486
ENST000003410121	-5.88	5.87E-04	0.0048
IGHV1-69D	-5.89	3.25E-04	0.0037
GZMK	-5.93	2.45E-06	0.0008
AC044849.1	-5.98	4.56E-03	0.0159
IGHV3-48	-5.99	9.74E-04	0.0064
ENST000002653753	-6.14	3.67E-04	0.0039
CYBB	-6.18	1.33E-03	0.0075
ENST000003345570	-6.18	1.77E-04	0.0028
ENST000002989101	-6.24	2.35E-04	0.0032
IGKV2-28	-6.29	3.02E-04	0.0036
A2M-AS1	-6.29	9.38E-04	0.0063
AC241640.4	-6.30	3.72E-04	0.0039
ENST000003206942	-6.32	2.23E-02	0.0475
IGKV1-12	-6.37	1.28E-04	0.0025
ENST000003209964	-6.39	1.49E-04	0.0026
BTG1P1	-6.40	3.98E-04	0.0040

IGHG4	-6.51	3.93E-04	0.0040
TNFRSF17	-6.53	1.93E-03	0.0094
IGLL5	-6.53	4.95E-04	0.0044
IGLV7-46	-6.57	1.89E-04	0.0029
IATPR	-6.61	3.21E-04	0.0037
ENST000002617490	-6.62	9.68E-05	0.0022
CCR9	-6.72	1.76E-04	0.0028
LINC00402	-6.86	5.58E-07	0.0008
ENST000002374490	-6.97	5.28E-05	0.0017
CPVL	-7.01	4.89E-05	0.0017
IGKV2-30	-7.17	4.06E-05	0.0016
FAM19A1	-7.25	2.99E-05	0.0014
IGKV3-15	-7.51	1.56E-04	0.0027
SORCS3	-7.69	1.74E-05	0.0011
ENST000002680491	-7.97	2.59E-04	0.0033
AC004832.6	-8.05	7.35E-05	0.0020
ENST000002443640	-8.06	9.59E-06	0.0010
ENST000002625100	-8.36	4.25E-04	0.0041
ENST000003088730	-8.59	7.15E-06	0.0010
ENST000003172572	-8.63	1.76E-05	0.0011
KLF2	-9.13	2.90E-06	0.0009
ENST000003256741	-9.44	7.26E-06	0.0010
ENST000003563380	-10.22	4.17E-07	0.0008
