# Supplementary material for: Human splice factors contribute to latent HIV infection in primary cell models and blood CD4+ T cells from ART-treated individuals
Source: PLoS Pathog. 2020 Nov 30;16(11):e1009060. doi: 10.1371/journal.ppat.1009060 (PMC7728277; doi:10.1371/journal.ppat.1009060)
Supplement: S4 Table — Genes are ranked alphabetically and the average log2 fold change (FC) is shown for each model. (PDF) [file ppat.1009060.s004.pdf]

Table S4. List of statistically significant differentially expressed genes (FDR<0.05) in stimulated vs. unstimulated peripheral CD4+ T cells from the Resting-cell model, the Wild-type model (wtNL4.3) and CD4+ T cells from ART-suppressed individuals. Genes are ranked alphabetically and the average log2 fold change (FC) is shown for each model.

			Fold Change (log2)		
Gene name	Gene description	Gene Family (a)	Resting-cell	Wild-type virus	Ex vivo
AC008555.1	novel transcript		-6.98	-4.21	-2.23
AC022079.1	novel transcript, sense intronic to CCDC91		-7.80	-2.91	-3.18
AC025171.4	novel transcript		-7.32	-5.04	-4.71
AC112721.2	novel protein (LOC728009)		7.02	6.09	4.68
AC130324.3	novel transcript, antisense to		7.67	2.36	2.49
AC241520.1	zinc finger protein pseudogene		-8.51	-1.58	-2.04
ACTG1	actin gamma 1 [Source:HGNC Symbol;Acc:HGNC:144]		2.25	1.70	1.95
AL354793.1	novel transcript		-6.27	-1.87	-1.77
AL391832.3	novel transcript		5.56	6.01	3.24
AL445524.1	novel transcript		7.97	4.46	3.09
AMY2B	amylase alpha 2B (pancreatic) [Source:HGNC		-2.77	-2.35	-2.38
ANKLE2	ankyrin repeat and LEM domain containing 2 [Source:HGNC		1.31	1.09	1.07
AP002365.1	programmed cell death 2, pseudogene		-8.35	-6.23	-3.82
AP003472.1	novel transcript		12.27	10.48	10.78
ARF6	ADP ribosylation factor 6 [Source:HGNC Symbol;Acc:HGNC:659]		1.66	1.19	1.38
ARFGEF3	ARFGEF family member 3 [Source:HGNC Symbol;Acc:HGNC:21213]		9.95	4.00	2.57
ARID5A	AT-rich interaction domain 5A [Source:HGNC Symbol;Acc:HGNC:17361]	Transcription factors	2.60	1.56	1.84
ASAH1	N-acylsphingosine amidohydrolase 1 [Source:HGNC Symbol;Acc:HGNC:735]		-1.43	-1.51	-1.05
ATM	ATM serine/threonine kinase [Source:HGNC Symbol;Acc:HGNC:795]	Protein kinases	-3.72	-2.87	-3.26

B3GALT2	beta-1,3-galactosyltransferase 2 [Source:HGNC Symbol;Acc:HGNC:917]		-9.73	-6.71	-3.42
BATF	basic leucine zipper ATF-like transcription factor [Source:HGNC Symbol;Acc:HGNC:958]	Transcription factors	3.70	3.09	4.10
BATF3	basic leucine zipper ATF-like transcription factor 3 [Source:HGNC Symbol;Acc:HGNC:28915]	Transcription factors	9.65	6.55	7.03
BCO2	beta-carotene oxygenase 2 [Source:HGNC Symbol;Acc:HGNC:18503]		-7.30	-1.38	-0.84
BIN2	bridging integrator 2 [Source:HGNC Symbol;Acc:HGNC:1053]		-1.31	-2.51	-1.56
BRCA1	BRCA1 DNA repair associated [Source:HGNC Symbol;Acc:HGNC:1100]		3.70	4.60	5.12
BRI3BP	BRI3 binding protein [Source:HGNC Symbol;Acc:HGNC:14251]		1.34	2.19	2.16
BRWD1	bromodomain and WD repeat domain containing 1 [Source:HGNC Symbol;Acc:HGNC:12760]		-1.75	1.71	-1.29
BTG1P1	BTG anti-proliferation factor 1 pseudogene 1 [Source:HGNC Symbol;Acc:HGNC:37692]		-6.80	-4.18	-6.40
C12orf42	chromosome 12 open reading frame 42 [Source:HGNC Symbol;Acc:HGNC:24729]		-8.92	-2.84	-4.78
C1GALT1P1	C1GALT1 pseudogene 1 [Source:HGNC Symbol;Acc:HGNC:51614]		-6.89	-3.07	-2.16
C3orf14	chromosome 3 open reading frame 14 [Source:HGNC Symbol;Acc:HGNC:25024]		7.88	2.60	3.21

C4orf47	chromosome 4 open reading frame 47 [Source:HGNC Symbol;Acc:HGNC:34346]		8.64	4.54	3.74
CALM3	calmodulin 3 [Source:HGNC Symbol;Acc:HGNC:1449]		2.26	2.38	2.62
CASP1P2	caspase 1 pseudogene 2 [Source:HGNC Symbol;Acc:HGNC:43776]		-8.21	-3.51	-2.52
CCDC150	coiled-coil domain containing 150 [Source:HGNC Symbol;Acc:HGNC:26834]		9.65	4.17	3.88
CCDC88C	coiled-coil domain containing 88C [Source:HGNC Symbol;Acc:HGNC:19967]		-0.98	-0.89	-1.02
CCNL2	cyclin L2 [Source:HGNC Symbol;Acc:HGNC:20570]		-1.63	-0.65	-1.06
CCNT2	cyclin T2 [Source:HGNC Symbol;Acc:HGNC:1600]	HIV interaction	-1.32	-1.34	-0.90
CD200	CD200 molecule [Source:HGNC Symbol;Acc:HGNC:7203]	Cell differentiation markers	2.60	4.81	4.69
CDC6	cell division cycle 6 [Source:HGNC Symbol;Acc:HGNC:1744]		4.69	5.41	6.05
CDCA2	cell division cycle associated 2 [Source:HGNC Symbol;Acc:HGNC:14623]		8.78	5.24	6.36
CDKN2B-AS1	CDKN2B antisense RNA 1 [Source:HGNC Symbol;Acc:HGNC:34341]		-6.03	-2.49	-1.64
CENPM	centromere protein M [Source:HGNC Symbol;Acc:HGNC:18352]		9.66	4.14	3.49
CENPN	centromere protein N [Source:HGNC Symbol;Acc:HGNC:30873]		3.04	3.91	3.89
CFAP58	cilia and flagella associated protein 58 [Source:HGNC Symbol;Acc:HGNC:26676]		7.34	3.69	5.27

CHAF1B	chromatin assembly factor 1 subunit B [Source:HGNC Symbol;Acc:HGNC:1911]		2.98	2.42	2.51
CHST3	carbohydrate sulfotransferase 3 [Source:HGNC Symbol;Acc:HGNC:1971]		5.85	2.79	1.06
CLIC1	chloride intracellular channel 1 [Source:HGNC Symbol;Acc:HGNC:2062]		2.74	0.92	1.90
CORO1B	coronin 1B [Source:HGNC Symbol;Acc:HGNC:2253]		4.14	0.90	2.65
DCLRE1B	DNA cross-link repair 1B [Source:HGNC Symbol;Acc:HGNC:17641]		3.67	1.98	3.13
DHFR	dihydrofolate reductase [Source:HGNC Symbol;Acc:HGNC:2861]		3.20	2.83	3.80
DOT1L	DOT1 like histone lysine methyltransferase [Source:HGNC Symbol;Acc:HGNC:24948]		1.54	1.36	1.44
DSC1	desmocollin 1 [Source:HGNC Symbol;Acc:HGNC:3035]		-9.70	-5.21	-4.94
DSN1	DSN1 component of MIS12 kinetochore complex [Source:HGNC Symbol;Acc:HGNC:16165]		1.45	1.83	2.12
EDAR	ectodysplasin A receptor [Source:HGNC Symbol;Acc:HGNC:2895]		-4.83	-4.66	-5.24
EIF4H	eukaryotic translation initiation factor 4H [Source:HGNC Symbol;Acc:HGNC:12741]	Transcription factors	1.73	1.76	1.56
EMP1	epithelial membrane protein 1 [Source:HGNC Symbol;Acc:HGNC:3333]		9.03	4.13	4.50
ENO1	enolase 1 [Source:HGNC Symbol;Acc:HGNC:3350]	Transcription factors	4.69	3.28	3.70
EVI2B	ecotropic viral integration site 2B [Source:HGNC Symbol;Acc:HGNC:3500]		-3.52	-3.13	-2.47

FAM19A1	TAFA chemokine like family member 1 [Source:HGNC Symbol;Acc:HGNC:21587]		-9.25	-7.19	-7.25
FAM57A	TLC domain containing 3A [Source:HGNC Symbol;Acc:HGNC:29646]		9.59	3.40	4.23
FAM71B	family with sequence similarity 71 member B [Source:HGNC Symbol;Acc:HGNC:28397]		8.36	6.24	4.70
FANCI	FA complementation group I [Source:HGNC Symbol;Acc:HGNC:25568]		3.10	2.93	3.46
FBXO43	F-box protein 43 [Source:HGNC Symbol;Acc:HGNC:28521]		7.47	5.05	4.61
FMR1	fragile X mental retardation 1 [Source:HGNC Symbol;Acc:HGNC:3775]		-1.31	-0.60	-1.10
FSD1	fibronectin type III and SPRY domain containing 1 [Source:HGNC Symbol;Acc:HGNC:13745]		8.73	1.17	1.39
FYB1	FYN binding protein 1 [Source:HGNC Symbol;Acc:HGNC:4036]		-2.99	-2.92	-2.43
FYCO1	FYVE and coiled-coil domain containing 1 [Source:HGNC Symbol;Acc:HGNC:14673]		-0.91	-1.69	-0.95
GALM	galactose mutarotase [Source:HGNC Symbol;Acc:HGNC:24063]		0.57	1.71	2.66
GALNT18	polypeptide N-acetylgalactosaminyltransferase 18 [Source:HGNC Symbol;Acc:HGNC:30488]		9.61	8.25	6.32
GAPDH	glyceraldehyde-3-phosphate dehydrogenase [Source:HGNC Symbol;Acc:HGNC:4141]		4.90	3.90	3.92

GARS	glycyl-tRNA synthetase [Source:HGNC Symbol;Acc:HGNC:4162]		1.01	1.85	2.84
GBP3	guanylate binding protein 3 [Source:HGNC Symbol;Acc:HGNC:4184]		-3.13	-2.52	-2.57
GIMAP1	GTPase, IMAP family member 1 [Source:HGNC Symbol;Acc:HGNC:23237]		-3.08	-3.06	-1.96
GIMAP7	GTPase, IMAP family member 7 [Source:HGNC Symbol;Acc:HGNC:22404]		-3.12	-3.29	-1.51
GJC1	gap junction protein gamma 1 [Source:HGNC Symbol;Acc:HGNC:4280]		8.41	1.36	0.88
GNG5	G protein subunit gamma 5 [Source:HGNC Symbol;Acc:HGNC:4408]		1.46	1.87	2.30
GNPDA1	glucosamine-6-phosphate deaminase 1 [Source:HGNC Symbol;Acc:HGNC:4417]		3.18	1.87	1.87
GPA33	glycoprotein A33 [Source:HGNC Symbol;Acc:HGNC:4445]		-9.61	-6.32	-4.77
GPN1	GPN-loop GTPase 1 [Source:HGNC Symbol;Acc:HGNC:17030]		1.25	1.37	1.09
HCG27	HLA complex group 27 [Source:HGNC Symbol;Acc:HGNC:27366]		-9.97	-1.27	-1.06
HIPK3	homeodomain interacting protein kinase 3 [Source:HGNC Symbol;Acc:HGNC:4915]	Protein kinases	-0.73	-0.52	-0.68
HIST1H4A	histone cluster 1 H4 family member a [Source:HGNC Symbol;Acc:HGNC:4781]		8.59	4.49	2.75
HIST1H4F	histone cluster 1 H4 family member f [Source:HGNC Symbol;Acc:HGNC:4783]		3.14	3.25	3.29
IFNG	interferon gamma [Source:HGNC Symbol;Acc:HGNC:5438]	Cytokines and growth factors	14.06	5.91	7.13

IGSF6	immunoglobulin superfamily member 6 [Source:HGNC Symbol;Acc:HGNC:5953]		-7.14	-1.09	-1.81
IKBKE	inhibitor of nuclear factor kappa B kinase subunit epsilon [Source:HGNC Symbol;Acc:HGNC:14552]	Protein kinases	-1.53	-1.78	-1.39
IL13	interleukin 13 [Source:HGNC Symbol;Acc:HGNC:5973]	Cytokines and growth factors	9.22	4.05	8.10
IL1A	interleukin 1 alpha [Source:HGNC Symbol;Acc:HGNC:5991]	Cytokines and growth factors	7.01	8.56	8.82
IL21	interleukin 21 [Source:HGNC Symbol;Acc:HGNC:6005]	Cytokines and growth factors	9.14	7.71	8.59
IL23R	interleukin 23 receptor [Source:HGNC Symbol;Acc:HGNC:19100]		2.75	4.98	4.29
IL24	interleukin 24 [Source:HGNC Symbol;Acc:HGNC:11346]	Cytokines and growth factors	2.24	8.33	8.19
IPCEF1	interaction protein for cytohesin exchange factors 1 [Source:HGNC Symbol;Acc:HGNC:21204]		-2.81	-2.75	-2.77
IRS2	insulin receptor substrate 2 [Source:HGNC Symbol;Acc:HGNC:6126]		-2.27	-2.11	-4.12
ITM2B	integral membrane protein 2B [Source:HGNC Symbol;Acc:HGNC:6174]		-2.54	-1.92	-2.19
KCNA2	potassium voltage-gated channel subfamily A member 2 [Source:HGNC Symbol;Acc:HGNC:6220]		-7.46	-2.45	-4.05
KIAA1551	retroelement silencing factor 1 [Source:HGNC Symbol;Acc:HGNC:25559]		-3.04	-2.73	-2.17
KIF11	kinesin family member 11 [Source:HGNC Symbol;Acc:HGNC:6388]		3.32	3.81	4.65

KIF15	kinesin family member 15 [Source:HGNC Symbol;Acc:HGNC:17273]		3.56	5.55	5.80
KLF3	Kruppel like factor 3 [Source:HGNC Symbol;Acc:HGNC:16516]	Transcription factors	-3.03	-4.17	-3.79
KLF3-AS1	KLF3 antisense RNA 1 [Source:HGNC Symbol;Acc:HGNC:25796]		-9.34	-0.81	-1.50
KLHDC1	kelch domain containing 1 [Source:HGNC Symbol;Acc:HGNC:19836]		-4.97	-2.14	-2.49
KLRB1	killer cell lectin like receptor B1 [Source:HGNC Symbol;Acc:HGNC:6373]	Cell differentiation markers	-8.03	-4.57	-5.18
L3HYPDH	trans-L-3-hydroxyproline dehydratase [Source:HGNC Symbol;Acc:HGNC:20488]		-2.21	-1.58	-1.09
L3MBTL3	L3MBTL histone methyl-lysine binding protein 3 [Source:HGNC Symbol;Acc:HGNC:23035]		-2.11	-1.37	-2.40
LCP1	lymphocyte cytosolic protein 1 [Source:HGNC Symbol;Acc:HGNC:6528]		1.39	1.03	1.30
LEMD3	LEM domain containing 3 [Source:HGNC Symbol;Acc:HGNC:28887]		-0.62	-0.59	-0.61
LILRB4	leukocyte immunoglobulin like receptor B4 [Source:HGNC Symbol;Acc:HGNC:6608]	Cell differentiation markers	-7.26	-1.80	-2.92
LIMA1	LIM domain and actin binding 1 [Source:HGNC Symbol;Acc:HGNC:24636]	Transcription factors	2.36	2.74	2.81
LINC00158	long intergenic non-protein coding RNA 158 [Source:HGNC Symbol;Acc:HGNC:1283]	Long-non-coding RNAs	6.83	8.32	4.30
LINC00205	long intergenic non-protein coding RNA 205 [Source:HGNC Symbol;Acc:HGNC:16420]	Long-non-coding RNAs	-6.72	-2.19	-1.70

LINC01138	long intergenic non-protein coding RNA 1138 [Source:HGNC Symbol;Acc:HGNC:49454]	Long-non-coding RNAs	-1.93	-2.57	-1.65
LINC02328	long intergenic non-protein coding RNA 2328 [Source:HGNC Symbol;Acc:HGNC:53248]	Long-non-coding RNAs	-9.12	-2.13	-2.64
LINC02362	long intergenic non-protein coding RNA 2362 [Source:HGNC Symbol;Acc:HGNC:53284]	Long-non-coding RNAs	-9.12	-1.85	-1.59
LINC02416	long intergenic non-protein coding RNA 2416 [Source:HGNC Symbol;Acc:HGNC:53345]	Long-non-coding RNAs	9.77	3.33	1.93
LMAN1	lectin, mannose binding 1 [Source:HGNC Symbol;Acc:HGNC:6631]		1.87	1.73	2.22
LSM12	LSM12 homolog [Source:HGNC Symbol;Acc:HGNC:26407]		1.05	1.89	2.54
MAGOH	mago homolog, exon junction complex subunit [Source:HGNC Symbol;Acc:HGNC:6815]	Metabolism of RNA	1.06	1.35	1.20
MAK	male germ cell associated kinase [Source:HGNC Symbol;Acc:HGNC:6816]	Protein kinases	8.19	4.92	4.33
MAN1C1	mannosidase alpha class 1C member 1 [Source:HGNC Symbol;Acc:HGNC:19080]		-1.71	-3.57	-1.68
MAP3K1	mitogen-activated protein kinase kinase kinase 1 [Source:HGNC Symbol;Acc:HGNC:6848]	Protein kinases	-2.03	-1.36	-2.11
MBTPS1	membrane bound transcription factor peptidase, site 1 [Source:HGNC Symbol;Acc:HGNC:15456]		-1.23	-1.00	-0.89
MDH2	malate dehydrogenase 2 [Source:HGNC Symbol;Acc:HGNC:6971]		2.37	1.67	2.19

MELK	maternal embryonic leucine zipper kinase [Source:HGNC Symbol;Acc:HGNC:16870]	Protein kinases	10.75	5.67	5.98
MFSD10	major facilitator superfamily domain containing 10 [Source:HGNC Symbol;Acc:HGNC:16894]		2.17	1.99	2.14
MGAT4A	alpha-1,3-mannosyl-glycoprotein 4-beta-N-acetylglucosaminyltransferase A [Source:HGNC Symbol;Acc:HGNC:7047]		-2.00	-1.83	-1.41
MIA3	MIA SH3 domain ER export factor 3 [Source:HGNC Symbol;Acc:HGNC:24008]		-1.06	-1.39	-0.61
MND1	meiotic nuclear divisions 1 [Source:HGNC Symbol;Acc:HGNC:24839]		9.80	6.93	5.81
MORC2	MORC family CW-type zinc finger 2 [Source:HGNC Symbol;Acc:HGNC:23573]		-0.81	-1.02	-0.79
MRPL47	mitochondrial ribosomal protein L47 [Source:HGNC Symbol;Acc:HGNC:16652]		1.96	1.71	1.79
MRPS10	mitochondrial ribosomal protein S10 [Source:HGNC Symbol;Acc:HGNC:14502]		-0.58	1.06	1.16
MSL2	MSL complex subunit 2 [Source:HGNC Symbol;Acc:HGNC:25544]		-0.96	-0.92	-0.70
MTHFD2	methylenetetrahydrofolate dehydrogenase (NADP+ dependent) 2, methenyltetrahydrofolate cyclohydrolase [Source:HGNC Symbol;Acc:HGNC:7434]		2.65	2.94	4.21
NAMPT	nicotinamide phosphoribosyltransferase [Source:HGNC Symbol;Acc:HGNC:30092]	Cytokines and growth factors	5.67	4.87	4.36

NCAPD2	non-SMC condensin I complex subunit D2 [Source:HGNC Symbol;Acc:HGNC:24305]		1.98	2.04	3.10
NCS1	neuronal calcium sensor 1 [Source:HGNC Symbol;Acc:HGNC:3953]		7.81	5.63	7.57
NIPAL1	NIPA like domain containing 1 [Source:HGNC Symbol;Acc:HGNC:27194]		9.62	1.64	1.39
NLE1	notchless homolog 1 [Source:HGNC Symbol;Acc:HGNC:19889]		1.34	2.07	1.51
NOB1	NIN1 (RPN12) binding protein 1 homolog [Source:HGNC Symbol;Acc:HGNC:29540]	Metabolism of RNA	1.78	0.93	2.08
NUP155	nucleoporin 155 [Source:HGNC Symbol;Acc:HGNC:8063]	Metabolism of RNA; HIV interaction	1.42	1.99	1.97
OCM	oncomodulin [Source:HGNC Symbol;Acc:HGNC:8105]		-9.64	-5.45	-3.66
ODF2	outer dense fiber of sperm tails 2 [Source:HGNC Symbol;Acc:HGNC:8114]		1.23	1.44	2.02
P4HA2	prolyl 4-hydroxylase subunit alpha 2 [Source:HGNC Symbol;Acc:HGNC:8547]		10.03	5.14	4.88
PALD1	phosphatase domain containing paladin 1 [Source:HGNC Symbol;Acc:HGNC:23530]		8.03	2.32	1.79
PAPOLA	poly(A) polymerase alpha [Source:HGNC Symbol;Acc:HGNC:14981]	Metabolism of RNA	-0.75	-0.54	-0.61
PARP8	poly(ADP-ribose) polymerase family member 8 [Source:HGNC Symbol;Acc:HGNC:26124]		-2.33	-2.19	-2.69
PAXIP1	PAX interacting protein 1 [Source:HGNC Symbol;Acc:HGNC:8624]	Transcription factors	1.15	1.08	1.06

PBK	PDZ binding kinase [Source:HGNC Symbol;Acc:HGNC:18282]	Protein kinases	8.30	7.48	6.90
PDAP1	PDGFA associated protein 1 [Source:HGNC Symbol;Acc:HGNC:14634]		1.38	1.79	2.35
PDCD1	programmed cell death 1 [Source:HGNC Symbol;Acc:HGNC:8760]	Cell differentiation markers	4.19	6.50	3.46
PGK1	phosphoglycerate kinase 1 [Source:HGNC Symbol;Acc:HGNC:8896]		3.41	3.51	2.39
PHF3	PHD finger protein 3 [Source:HGNC Symbol;Acc:HGNC:8921]	Transcription factors	-0.94	-0.56	-0.62
PIKFYVE	phosphoinositide kinase, FYVE-type zinc finger containing [Source:HGNC Symbol;Acc:HGNC:23785]		-1.11	-0.82	-0.57
PIM3	Pim-3 proto-oncogene, serine/threonine kinase [Source:HGNC Symbol;Acc:HGNC:19310]	Protein kinases	2.33	2.94	2.46
PLCL1	phospholipase C like 1 (inactive) [Source:HGNC Symbol;Acc:HGNC:9063]		-2.89	-3.32	-3.69
POT1-AS1	POT1 antisense RNA 1 [Source:HGNC Symbol;Acc:HGNC:49459]		-7.97	-1.74	-1.48
PSMA3-AS1	PSMA3 antisense RNA 1 [Source:HGNC Symbol;Acc:HGNC:26445]		-1.60	-1.24	-2.02
PSMD14	proteasome 26S subunit, non-ATPase 14 [Source:HGNC Symbol;Acc:HGNC:16889]	Metabolism of RNA; HIV interaction	1.39	2.10	2.35
PSRC1	proline and serine rich coiled-coil 1 [Source:HGNC Symbol;Acc:HGNC:24472]		7.56	3.14	5.47
PTPN11	protein tyrosine phosphatase non-receptor type 11 [Source:HGNC Symbol;Acc:HGNC:9644]		1.38	2.02	1.76

RAB1A	RAB1A, member RAS oncogene family [Source:HGNC Symbol;Acc:HGNC:9758]		0.56	0.88	0.78
RAB27A	RAB27A, member RAS oncogene family [Source:HGNC Symbol;Acc:HGNC:9766]		1.97	1.29	1.76
RAC3	Rac family small GTPase 3 [Source:HGNC Symbol;Acc:HGNC:9803]		8.46	4.01	2.42
RAD51AP1	RAD51 associated protein 1 [Source:HGNC Symbol;Acc:HGNC:16956]		3.35	5.51	5.03
RAI14	retinoic acid induced 14 [Source:HGNC Symbol;Acc:HGNC:14873]		7.86	2.75	2.43
RANBP1	RAN binding protein 1 [Source:HGNC Symbol;Acc:HGNC:9847]	HIV interaction	2.44	3.21	3.18
RANGAP1	Ran GTPase activating protein 1 [Source:HGNC Symbol;Acc:HGNC:9854]	HIV interaction	1.25	1.95	1.99
RASSF3	Ras association domain family member 3 [Source:HGNC Symbol;Acc:HGNC:14271]		-2.71	-2.23	-1.84
RBPJ	recombination signal binding protein for immunoglobulin kappa J region [Source:HGNC Symbol;Acc:HGNC:5724]		1.92	1.91	1.81
RHPN2	rhophilin Rho GTPase binding protein 2 [Source:HGNC Symbol;Acc:HGNC:19974]	Transcription factors	6.84	2.88	2.05
RMDN1	regulator of microtubule dynamics 1 [Source:HGNC Symbol;Acc:HGNC:24285]		0.94	0.74	1.09
RN7SL471P	RNA, 7SL, cytoplasmic 471, pseudogene [Source:HGNC Symbol;Acc:HGNC:46487]		9.63	5.20	7.29

RNF216	ring finger protein 216 [Source:HGNC Symbol;Acc:HGNC:21698]		-1.85	-1.05	-1.11
RNVU1-7	RNA, variant U1 small nuclear 7 [Source:HGNC Symbol;Acc:HGNC:37500]	Metabolism of RNA	6.83	1.96	2.19
SAFB2	scaffold attachment factor B2 [Source:HGNC Symbol;Acc:HGNC:21605]		-1.00	-0.61	-0.70
SAMD9	sterile alpha motif domain containing 9 [Source:HGNC Symbol;Acc:HGNC:1348]		-3.02	-2.99	-2.16
SCML4	Scm polycomb group protein like 4 [Source:HGNC Symbol;Acc:HGNC:21397]		-2.84	-2.93	-3.35
SDC4	syndecan 4 [Source:HGNC Symbol;Acc:HGNC:10661]		3.90	6.49	5.04
SEC13	SEC13 homolog, nuclear pore and COPII coat complex component [Source:HGNC Symbol;Acc:HGNC:10697]	Metabolism of RNA; HIV interaction	1.05	1.33	1.67
SGO2	shugoshin 2 [Source:HGNC Symbol;Acc:HGNC:30812]		1.66	3.14	4.51
SHMT2	serine hydroxymethyltransferase 2 [Source:HGNC Symbol;Acc:HGNC:10852]		3.05	2.81	3.83
SLC17A9	solute carrier family 17 member 9 [Source:HGNC Symbol;Acc:HGNC:16192]		7.95	2.10	1.76
SLC22A23	solute carrier family 22 member 23 [Source:HGNC Symbol;Acc:HGNC:21106]		-8.60	-5.24	-3.60
SMCR8	SMCR8-C9orf72 complex subunit [Source:HGNC Symbol;Acc:HGNC:17921]		-2.24	-0.98	-1.17
SNHG14	small nucleolar RNA host gene 14 [Source:HGNC Symbol;Acc:HGNC:37462]		-1.92	-1.18	-1.01
SNHG16	small nucleolar RNA host gene 16 [Source:HGNC Symbol;Acc:HGNC:44352]		1.50	2.01	0.77

SNRNP25	small nuclear ribonucleoprotein U11/U12 subunit 25 [Source:HGNC Symbol;Acc:HGNC:14161]	Metabolism of RNA	3.69	2.48	3.72
SNRPD2	small nuclear ribonucleoprotein D2 polypeptide [Source:HGNC Symbol;Acc:HGNC:11159]	Metabolism of RNA	1.22	1.31	1.54
SORL1	sortilin related receptor 1 [Source:HGNC Symbol;Acc:HGNC:11185]		-2.60	-2.56	-2.54
SPC25	SPC25 component of NDC80 kinetochore complex [Source:HGNC Symbol;Acc:HGNC:24031]		8.46	4.46	4.24
SPR	sepiapterin reductase [Source:HGNC Symbol;Acc:HGNC:11257]		7.59	7.78	6.42
SRGN	serglycin [Source:HGNC Symbol;Acc:HGNC:9361]		3.12	2.60	1.23
SSBP2	single stranded DNA binding protein 2 [Source:HGNC Symbol;Acc:HGNC:15831]		-1.64	-1.60	-1.88
STAP2	signal transducing adaptor family member 2 [Source:HGNC Symbol;Acc:HGNC:30430]		7.72	1.75	2.02
STARD4	StAR related lipid transfer domain containing 4 [Source:HGNC Symbol;Acc:HGNC:18058]		3.06	1.63	2.23
STK38	serine/threonine kinase 38 [Source:HGNC Symbol;Acc:HGNC:17847]	Protein kinases	-2.80	-2.69	-1.97
STXBP5	syntaxin binding protein 5 [Source:HGNC Symbol;Acc:HGNC:19665]		-1.35	-1.17	-0.97
STYX	serine/threonine/tyrosine interacting protein [Source:HGNC Symbol;Acc:HGNC:11447]		-0.98	-1.04	-0.74

SYNGR3	synaptogyrin 3 [Source:HGNC Symbol;Acc:HGNC:11501]		7.47	4.26	2.92
SYNPO2	synaptopodin 2 [Source:HGNC Symbol;Acc:HGNC:17732]		-7.63	-1.69	-1.72
SYTL3	synaptotagmin like 3 [Source:HGNC Symbol;Acc:HGNC:15587]		3.65	2.69	2.07
TBX21	T-box transcription factor 21 [Source:HGNC Symbol;Acc:HGNC:11599]	Transcription factors	3.51	3.83	2.64
TC2N	tandem C2 domains, nuclear [Source:HGNC Symbol;Acc:HGNC:19859]		-2.66	-3.45	-2.25
TFDP1	transcription factor Dp-1 [Source:HGNC Symbol;Acc:HGNC:11749]	Transcription factors	2.72	2.60	2.45
THAP4	THAP domain containing 4 [Source:HGNC Symbol;Acc:HGNC:23187]		1.15	0.77	1.29
TK1	thymidine kinase 1 [Source:HGNC Symbol;Acc:HGNC:11830]		9.63	4.72	5.93
TMEM116	transmembrane protein 116 [Source:HGNC Symbol;Acc:HGNC:25084]		-1.47	-0.97	-1.18
TMEM191A	transmembrane protein 191A (pseudogene) [Source:HGNC Symbol;Acc:HGNC:25317]		-7.65	-3.10	-2.50
TMEM256	transmembrane protein 256 [Source:HGNC Symbol;Acc:HGNC:28618]		5.70	1.06	1.54
TNFRSF4	TNF receptor superfamily member 4 [Source:HGNC Symbol;Acc:HGNC:11918]	Cell differentiation markers	5.49	6.21	4.07
TONSL	tonsoku like, DNA repair protein [Source:HGNC Symbol;Acc:HGNC:7801]	Transcription factors	2.89	2.21	2.95
TPM4	tropomyosin 4 [Source:HGNC Symbol;Acc:HGNC:12013]		3.11	1.82	2.07

TRANK1	tetratricopeptide repeat and ankyrin repeat containing 1 [Source:HGNC Symbol;Acc:HGNC:29011]		-3.17	-3.87	-1.85
TRMT6	tRNA methyltransferase 6 [Source:HGNC Symbol;Acc:HGNC:20900]	Metabolism of RNA	1.54	0.79	1.24
TSC22D1-AS1	TSC22D1 antisense RNA 1 [Source:HGNC Symbol;Acc:HGNC:43684]		-8.01	-1.70	-3.19
TTK	TTK protein kinase [Source:HGNC Symbol;Acc:HGNC:12401]	Protein kinases	4.15	5.19	5.96
TUBA1B	tubulin alpha 1b [Source:HGNC Symbol;Acc:HGNC:18809]		3.89	4.76	4.29
TULP4	TUB like protein 4 [Source:HGNC Symbol;Acc:HGNC:15530]	Transcription factors	-1.85	-1.10	-1.84
TXLNA	taxilin alpha [Source:HGNC Symbol;Acc:HGNC:30685]	Cytokines and growth factors	1.46	1.18	1.91
UTRN	utrophin [Source:HGNC Symbol;Acc:HGNC:12635]		-2.23	-1.60	-2.13
WDR76	WD repeat domain 76 [Source:HGNC Symbol;Acc:HGNC:25773]		2.65	2.35	3.26
WNT7A	Wnt family member 7A [Source:HGNC Symbol;Acc:HGNC:12786]		-8.87	-5.72	-4.91
XRCC3	X-ray repair cross complementing 3 [Source:HGNC Symbol;Acc:HGNC:12830]		2.99	2.80	2.51
YEATS2	YEATS domain containing 2 [Source:HGNC Symbol;Acc:HGNC:25489]		1.86	1.35	1.56
ZBTB32	zinc finger and BTB domain containing 32 [Source:HGNC Symbol;Acc:HGNC:16763]	Transcription factors	12.37	7.32	6.40
ZEB1	zinc finger E-box binding homeobox 1 [Source:HGNC Symbol;Acc:HGNC:11642]	Transcription factors	-0.94	-0.78	-1.17

ZNF181	zinc finger protein 181 [Source:HGNC Symbol;Acc:HGNC:12971]		-3.30	-1.32	-0.69
ZNF235	zinc finger protein 235 [Source:HGNC Symbol;Acc:HGNC:12866]	Transcription factors	-1.86	-2.08	-1.27
ZNF367	zinc finger protein 367 [Source:HGNC Symbol;Acc:HGNC:18320]		1.85	2.89	3.03
ZNF44	zinc finger protein 44 [Source:HGNC Symbol;Acc:HGNC:13110]	Transcription factors	-2.39	-1.54	-1.44
ZNF548	zinc finger protein 548 [Source:HGNC Symbol;Acc:HGNC:26561]		-1.78	-0.97	-1.33
ZNF75D	zinc finger protein 75D [Source:HGNC Symbol;Acc:HGNC:13145]		-2.21	-1.63	-0.84
ZNF792	zinc finger protein 792 [Source:HGNC Symbol;Acc:HGNC:24751]		-1.41	-1.54	-1.06
ZNF879	zinc finger protein 879 [Source:HGNC Symbol;Acc:HGNC:37273]		-2.80	-1.90	-1.54

a. Gene family determinated by Gene Set Enrichment Analysis (GSEA) and Reactome databases
