# Supplementary figures and images for: Human splice factors contribute to latent HIV infection in primary cell models and blood CD4+ T cells from ART-treated individuals
Source: PLoS Pathog. 2020 Nov 30;16(11):e1009060. doi: 10.1371/journal.ppat.1009060 (PMC7728277; doi:10.1371/journal.ppat.1009060)

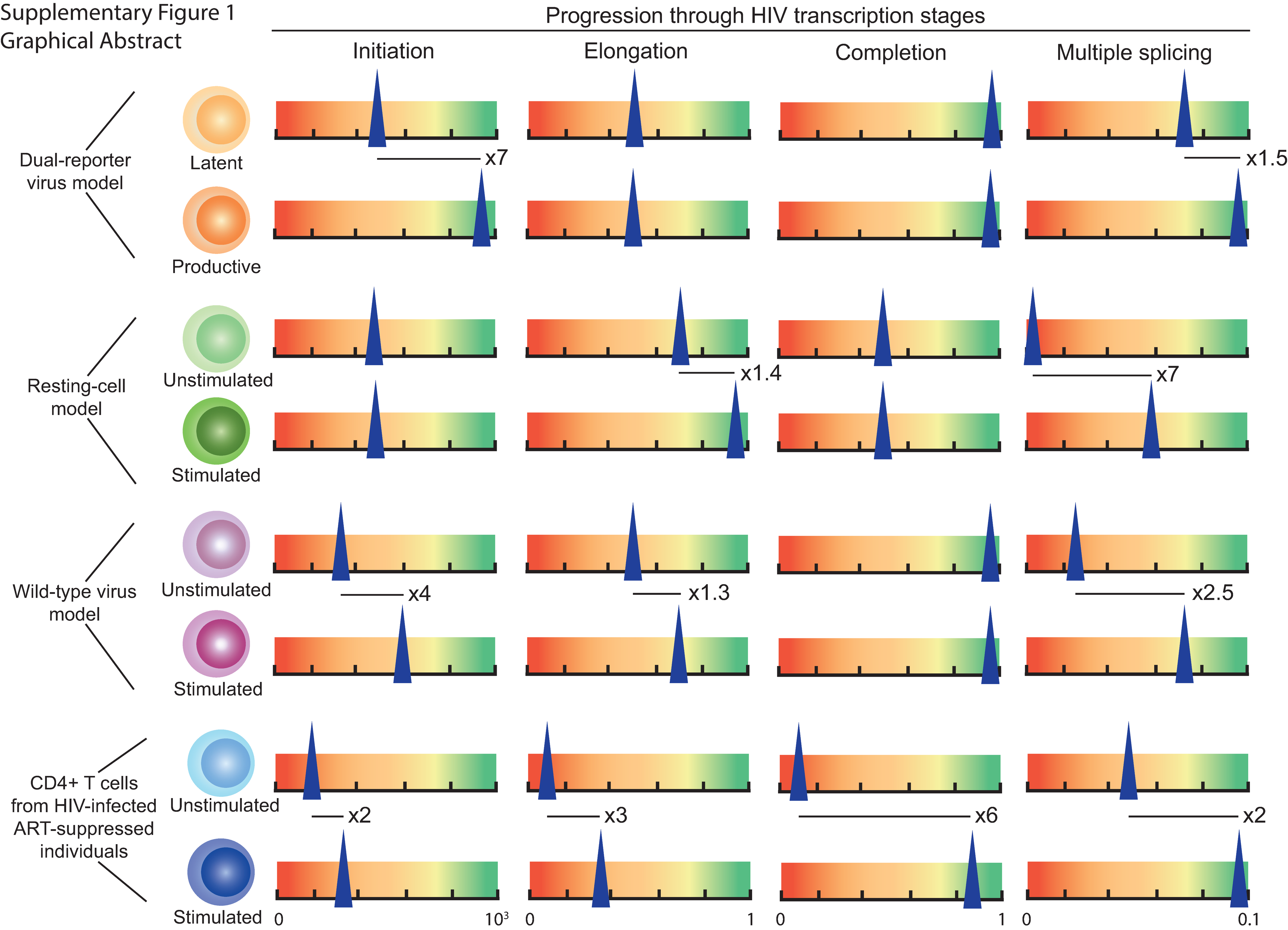

Supplement: S1 Fig — This schematic representation shows relative levels of HIV transcription initiation, elongation, completion, and multiple splicing quantified in latently/unstimulated and productively/stimulated HIV infected cells from the Dual-reporter, Resting-cell, and Wild-type primary cell HIV latency models and from CD4+ T cells from HIV-infected ART-suppressed individuals. The scale depicts the maximal block to transcription (red) to no transcriptional block (green), and the blue arrow indicates the comparative progression through each stage of HIV transcription. (TIF) [file ppat.1009060.s005.tif]

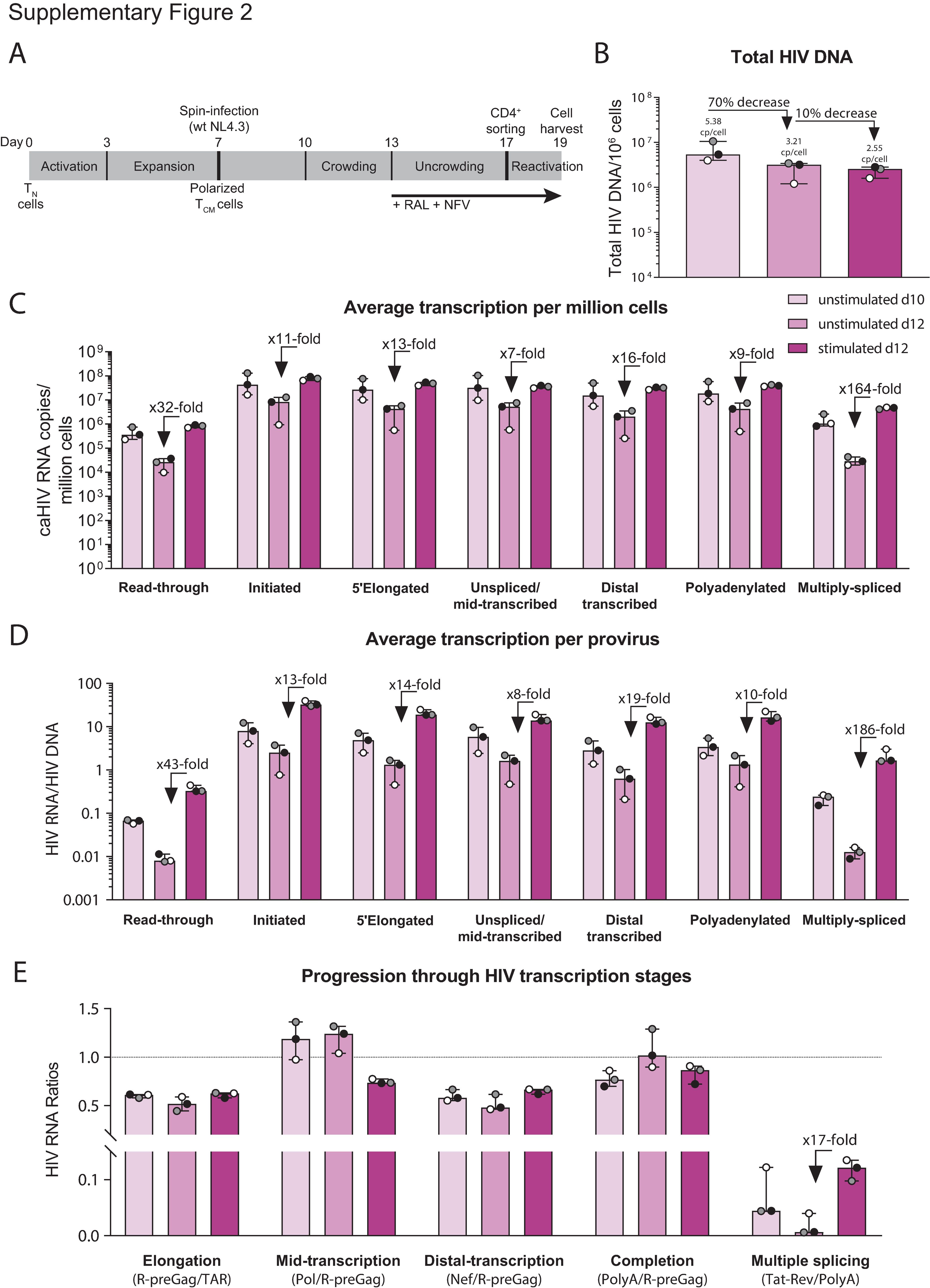

Supplement: S2 Fig — (A) Diagram of the model, (B) total HIV DNA, (C) level of HIV transcripts million cells, (D) level of HIV transcripts per provirus, (E) progression through HIV transcription stages. Individual values per donor (dots), and median and range (bars) are shown. Unstimulated cells at days 10 and 12 post-infection are shown in light colors and stimulated cells at day 12 in dark color. (TIF) [file ppat.1009060.s006.tif]

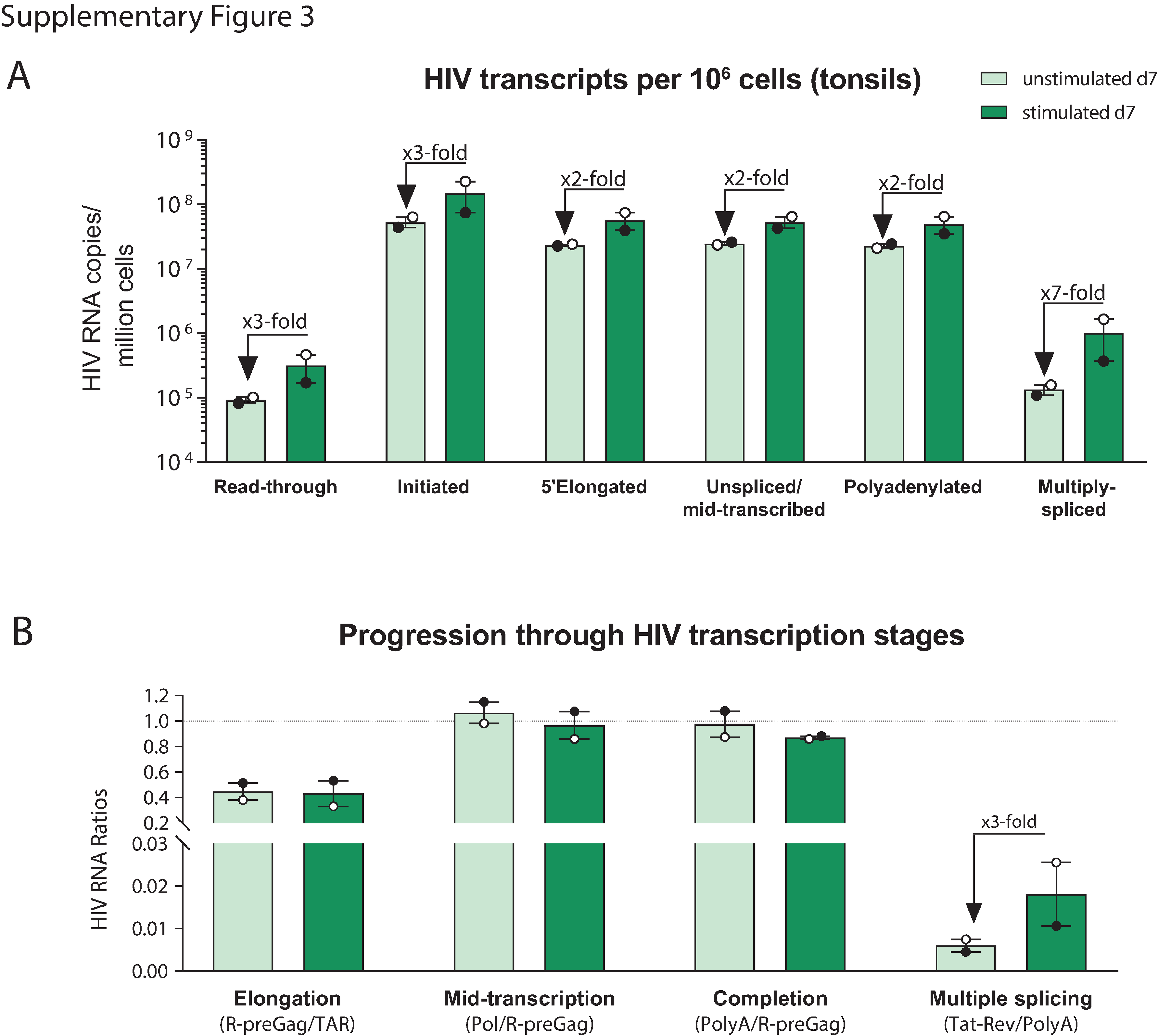

Supplement: S3 Fig — (A) Level of HIV transcripts per million cells, (B) progression through HIV transcription stages. Individual values per donor (dots), and median and range (bars) are shown. Unstimulated cells are shown in light color and stimulated cells in dark color. (TIF) [file ppat.1009060.s007.tif]

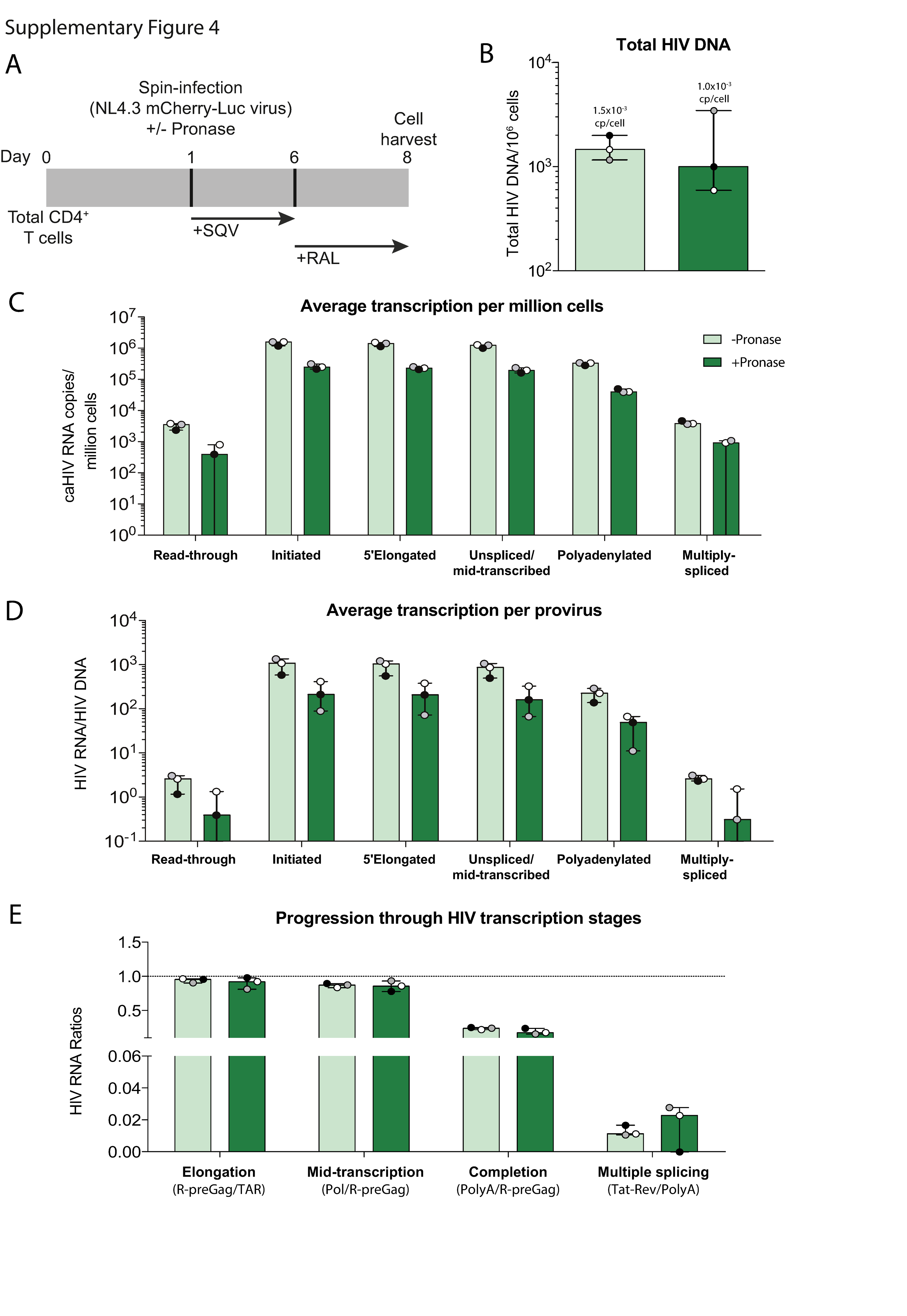

Supplement: S4 Fig — (A) Diagram of the model, (B) total HIV DNA, (C) level of HIV transcripts per million cells, (D) level of HIV transcripts per provirus, (E) progression through HIV transcription stages. Individual values per donor (dots), and median and range (bars) are shown. Non-pronase treated cells are shown in light color and pronase treated cells in dark color. (TIF) [file ppat.1009060.s008.tif]

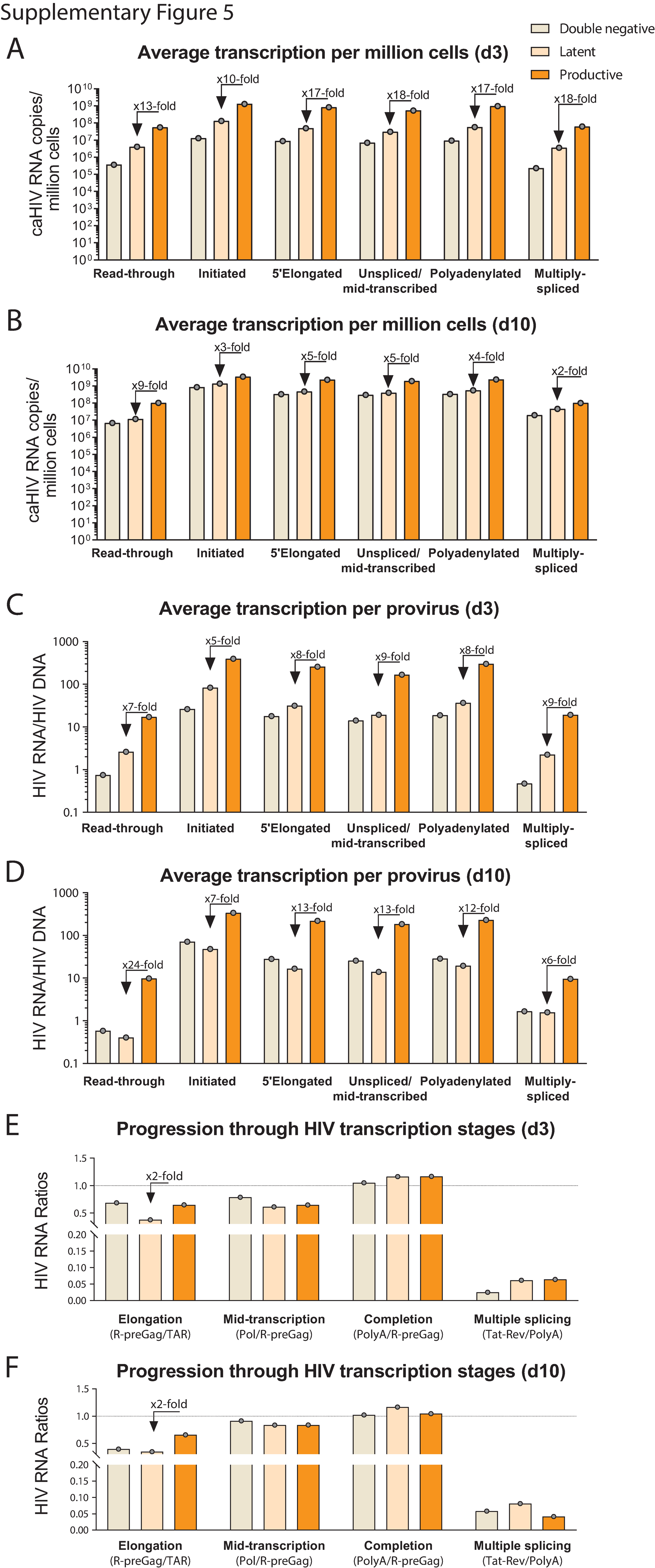

Supplement: S5 Fig — (A-B) Level of HIV transcripts per million cells at days 3 and 10 after infection, (C-D) Level of HIV transcripts per provirus at days 3 and 10 after infection, (E-F) progression through HIV transcription stages at days 3 and 10 after infection. Individual values per donor (dot) are shown. Double negative and latent cells are shown in light colors and productive cells in dark color. (TIF) [file ppat.1009060.s009.tif]

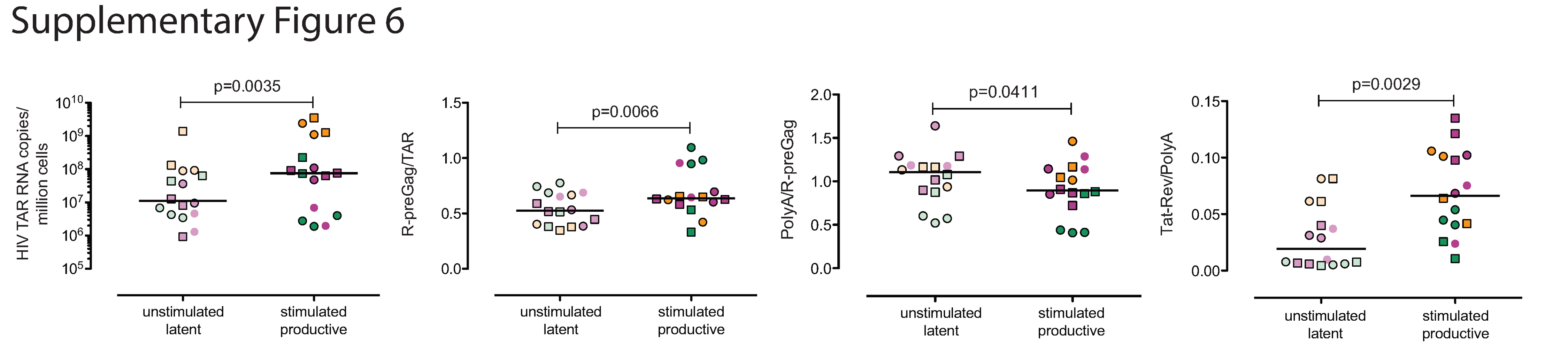

Supplement: S6 Fig — Levels of HIV transcriptional initiation, elongation, completion, and multiple splicing are shown in the latent (unstimulated) and productive (stimulated) populations from all donors used in the three main models (Figs 2–4) plus supplementary experiments (S2–S4 Figs). Bars indicate the median; P-values were calculated using the Wilcoxon signed rank test. (TIF) [file ppat.1009060.s010.tif]
